# Identification of candidate chemosensory genes by transcriptome analysis in *Loxostege sticticalis* Linnaeus

**DOI:** 10.1371/journal.pone.0174036

**Published:** 2017-04-19

**Authors:** Hong-Shuang Wei, Ke-Bin Li, Shuai Zhang, Ya-Zhong Cao, Jiao Yin

**Affiliations:** State Key Laboratory for Biology of Plant Diseases and Insect Pests, Institute of Plant Protection, Chinese Academy of Agricultural Sciences, Beijing, China; Institute of Plant Physiology and Ecology Shanghai Institutes for Biological Sciences, CHINA

## Abstract

*Loxostege stic*ticalis Linnaeus is an economically important agricultural pest, and the larvae cause great damage to crops, especially in Northern China. However, effective and environmentally friendly chemical methods for controlling this pest have not been discovered to date. In the present study, we performed HiSeq2500 sequencing of transcriptomes of the male and female adult antennae, adult legs and third instar larvae, and we identified 54 candidate odorant receptors (ORs), including 1 odorant receptor coreceptor (Orco) and 5 pheromone receptors (PRs), 18 ionotropic receptors (IRs), 13 gustatory receptors (GRs), 34 odorant binding proteins (OBPs), including 1 general odorant binding protein (GOBP1) and 3 pheromone binding proteins (PBPs), 10 chemosensory proteins (CSPs) and 2 sensory neuron membrane proteins (SNMPs). The results of RNA-Seq and RT-qPCR analyses showed the expression levels of most genes in the antennae were higher than that in the legs and larvae. Furthermore, *PR4*, *OR1-4*, *7–11*, *13–15*, *23*, *29–32*, *34*, *41*, *43*, *47*/*IR7d*.*2*/*GR5b*, *45*, *7*/*PBP2-3*, *GOBP1*, *OBP3*, *8* showed female antennae-biased expression, while *PR1*/*OBP2*, *7*/*IR75d*/*CSP2* showed male antennae-biased expression. However, *IR1*, *7d*.*3*, *68a/OBP11*, *20–22*, *28/CSP9* had larvae enriched expression, and *OBP15*, *17*, *25*, *29/CSP5* were mainly expressed in the legs. The results shown above indicated that these genes might play a key role in foraging, seeking mates and host recognition in the *L*. *sticticalis*. Our findings will provide the basic knowledge for further studies on the molecular mechanisms of the olfactory system of *L*. *sticticalis* and potential novel targets for pest control strategies.

## Introduction

The beet webworm, *Loxostege sticticalis* L. (Lepidoptera: Pyralidae), a worldwide distributed and migratory pest in North China, causes serious economic damage every year [1, 2]. *L*. *sticticalis* seems to be polyphagous in its larval stage, but it has been reported to have obvious host-plant selection for crops (sugar beet, potato and soybean) and pastures [3–5]. This has been associated with its highly developed olfactory system to detect and distinguish the host-plant volatiles [5, 6].

Chemical sensing by olfaction can regulate insect behaviors, including seeking food, choosing mates, locating suitable oviposition sites, and avoiding natural enemies [7, 8, 9]. Insects discern chemical signals by olfactory receptor neurons (ORNs) in the olfactory sensilla [8]. The ORNs located at the sensilla root are the primary units of olfaction in the insect antennae which include the odorant binding proteins (OBPs), chemosensory proteins (CSPs), odorant receptors (ORs), ionotropic receptors (IRs), and the sensory neuron membrane proteins (SNMPs) [8, 10]. OBPs dissolved in the sensilla lymph are some kinds of acidic proteins with a pattern of six conserved cysteine residues [11]. Insect OBPs were mainly expressed in the antennae of both sexes, which allows the insect to identify odor molecules in environment and plays an important role in the process of insect host location [12, 13]. Two subfamilies of OBPs, general odorant-binding proteins (GOBPs) and pheromone binding proteins (PBPs), are respectively responsible for recognizing and transporting host-plant volatiles and pheromones to ORs to protect them from odorant-degrading enzymes (ODEs) [14–16]. Same as OBPs, other soluble proteins named CSPs are also secreted in the sensillum lymph [16]. Although the functions of CSPs reported in previous articles are analogous to OBPs, they are still poorly understood. SNMPs with two transmembrane domains, the accepting stations of odorant ligands located in the dendritic membranes of pheromone-sensitive neurons, play a role in capturing pheromone molecules in coordination with ORs [17–19].

There are two types of olfactory receptor (ORs and IRs) proteins and one type of gustatory receptors (GRs) in insects. The conventional ORs binding the ligand molecules released by OBPs are also trans-membrane proteins with seven conservative transmembrane domains [20]. Pheromone sensilla primarily located on the antennae can perceive the pheromone molecules at the periphery of the olfactory system, and pheromone molecules transported to the dendritic membranes of ORNs are recognized by pheromone receptors (PRs), which are a subclass of insect ORs [21]. Beyond that, the odorant receptor coreceptor (Orco) was proved to be heteromeric ligand-gated ion channels and cyclic-nucleotide-activated cation channels with the capacity for transforming chemical signals to electric signals [22–25]. Compared to ligands (esters and alcohols) binding to ORs, IRs are narrowly tuned for amine and acid ones [26, 27, 28]. Furthermore, IRs are more standard ion acceptors compared with the ORs [26, 27]. A family proteins of sense of taste expressed in the antennae, proboscis and palps, GRs, were still exposed that they were adjusted for CO_2_ detection and responsible for selecting brooding spots [29, 30].

In the Lepidoptera, the antenna is a specialized organ for insect sensing, especially for olfaction, and many olfactory genes in some moths have been studied by antennal transcriptome analysis [31, 32]. However, the legs that also have a special olfaction sense though less sensitive than olfaction in the antennae [33, 34], its olfactory gene database seems incomplete for the *L*. *sticticalis*. In this study, we sequenced and analyzed integral transcriptomes of *L*. *sticticalis* adult antennae, adult legs and third instar larvae using Illumina sequencing platform. Our aims were to identify chemosensory genes of *L*. *sticticalis* and report the results including sequencing, gene annotation, GO annotation and specifically, identification and expression pattern of ORs, IRs, GRs, OBPs, CSPs and SNMPs.

## Materials and methods

### Insect rearing and RNA preparation

The beet webworms were acquired from a laboratory population at the Institute of Plant Protection, Chinese Academy of Agricultural Sciences (Beijing, China). The insects were fed an artificial diet at a temperature of 22 ± 1°C with 70% ± 10% relative humidity under a photoperiod of 16L: 8D (Light, Dark). When the larvae grew up to the third instar, 20 third instar larvae were picked and frozen in liquid nitrogen for conservation. Male and female pupae were placed into separate cages for eclosion. The adult moths were fed with a 5% honey solution after emergence. The antennae and legs from the male and female individuals were excised at 1 to 3 days after eclosion, immediately frozen and stored in liquid nitrogen until the RNA extraction.

The total RNAs were isolated from 100 adult male antennae, 100 adult female antennae, 24 adult legs (male: female = 1:1) and 2 third instar larvae respectively. Three biological replicates were prepared for each pilot part. Total RNA was extracted using Trizol reagent (Invitrogen, Shanghai, China), following the manufacturer’s instructions. The integrity of the RNA samples was detected by gel electrophoresis, and a NanoDrop 2000 spectrophotometer (NanoDrop, Wilmington, DE, USA) was used to determine RNA quantity. Before sequencing, the RNA samples were stored at -80°C.

### cDNA library construction, and Illumina sequencing

The cDNA library construction and Illumina sequencing of our RNA samples were performed at Biomarker technologies CO., LTD., Beijing, China. First, the NanoDrop 2000, Qubit 2.0(Invitrogen, Carlsbad, CA, USA) and Agilent 2100 (Agilent Technologies, Santa Clara, CA, USA) methods were used respectively to detect the purity, concentration and integrity of each RNA sample (10ug). Second, Oligo (dT) magnetic beads were used to gather mRNA (poly-A RNA). Using a fragmentation buffer, the mRNA of each sample was broken into short fragments randomly at 94°C for 5 min. Third, The first-strand cDNA were synthesized using N6 random primers and mRNA templates and the second strand cDNA were synthesized using buffer, dNTPs, RNase H and DNA polymerase I. The synthetic cDNA was purified using AMPure XP Beads (Beckman Coulter, Inc.). These dual-strand DNA samples were treated with T4 DNA polymerase and T4 polynucleotide kinase, respectively, for end-repairing and dA-tailing, followed by adaptor ligation to the dA tail of the dsDNA using T4 DNA ligase. Then, suitable fragments were selected with AMPure XP beads (Beckman Coulter, Inc.). Finally, the products were amplified by PCR and purified using the QIAquick PCR Purification Kit (Qiagen, Valencia, CA, USA) to create a cDNA library. The libraries were sequenced on an Illumina HiSeq^™^ 2500 platform, and paired-end reads were generated using a PE125 strategy (paired-end reads of 125 base pairs per read).

### *De novo* assembly and function annotation

High-quality clean reads were obtained from the raw reads by removing reads containing either an adapter or poly-N sequence and reads that were in low-quality. Transcriptome de novo assembly was performed with the short read assembly program Trinity [35]. Then, the Trinity outputs were clustered by TGICL [36]. The consensus cluster sequences and singletons compose the unigene dataset. The annotation of unigenes was performed by NCBI BLASTx against a pooled database of non-redundant (nr) and Swiss-Prot protein sequences with e-values < 1e-5. The Blast results were then imported into the Blast2GO [37] pipeline for GO Annotation. Protein coding region prediction was performed by OrfPredictor [38] according to the blast results.

### Sequence analysis

The sequence analysis methods used in this paper were as previously described [33]. First, the open reading frames (ORFs) of chemosensory genes in *L*. *sticticalis* were predicted online using ORF finder (http://www.ncbi.nlm.nih.gov/gorf/gorf.html). Second, similarity searches were performed with the NCBI-BLAST network server (http://blast.ncbi.nlm.nih.gov/). Then, N-terminal signal peptides of putative LstiOBPs and LstiCSPs were predicted by the SignalP 4.0 server (http://www.cbs.dtu.dk/services/SignalP/). The transmembrane domains of the candidate LstiORs, LstiIRs, LstiGRs and LstiSNMPs were predicted with the TMHMM Server Version 2.0 (http://www.cbs.dtu.dk/services/TMHMM). The nucleotide sequences of all identified olfactory gene are listed in supporting information (S1 Table).

### Phylogenetic tree analysis

Multiple alignments of the *L*. *sticticalis* amino acid sequences of the chemosensory genes were performed by ClustalX 2.0 [39]. The phylogenetic trees were constructed by MEGA 6.0 [40] using the neighbor-joining method [41] with a p-distance model and a pairwise deletion of gaps. Bootstrap support was assessed by a boot strap procedure based on 1000 replicates. The data sets of chemosensory gene sequences, which were chosen from other Lepidopteran species, are listed in supporting information (S2 Table).

### RT-qPCR analysis

Using real-time quantitative PCR (RT-qPCR), we measured the expression profiles of chemosensory genes in different parts (male antennae, female antennae, legs and third instar larvae). The primers used for the RT-qPCR were designed using the Primer Premier 5.0, which are listed in supporting information (S3 Table). The RT-qPCR was performed by ABI 7500 Detection System (Applied Biosystems, Carlsbad, CA, USA). Before transcription, RQ1 RNase-Free DNase (Promega, Madison, USA) was used to remove residual genomic DNA of total RNA. An equal amount of cDNA (150 ng/u l) was synthesized using 1^st^ strand cDNA synthesis kits (TaKaRa, Dalian, China) as the RT-qPCR templates. Each RT-qPCR reaction was conducted in a 25 μ l reaction: 12.5 μ l of 2X SuperReal PreMix Plus (TianGen, Beijing, China), 0.75 μ l of each primer (10 μ M), 2 μ l of sample cDNA, and 9 μ l of sterilized ddH_2_O. The RT-qPCR was run as follows: 94°C for 2 min, followed by 40 cycles of 95°C for 15 s, 60°C for 30 s, 60°C for 1 min, heated to 95°C for 30 s and cooled to 60°C for 15 s to measure the melting curve.

RT-qPCR data analyses were performed using the 2^-ΔΔCT^ method [42]. Data of relative expression levels in various tissue were subjected to one-way analysis of variance (ANOVA), followed by a least significant difference test (Tukey) for mean comparison. The data were analyzed directly by SPSS 9.20 software (SPSS Inc., Chicago, IL, USA). Differences were considered significant at p < 0.05. The RT-qPCR data were analyzed and exported as TIF files by Graphpad Prism 5.0 (GraphPad Software, La Jolla, CA, USA).

## Results

### Transcriptome assembly of *L*. *sticticalis*

Using the Illumina HiSeq^™^ 2500 platform, we performed next-generation sequencing on a cDNA library constructed from *L*. *sticticalis*. A total of 869.3 million clean reads (86.93 Gb) were obtained. Q30 bases were more than 85.01% in all the samples. After *de novo* assembly, we assembled 3,266,885 contigs with a mean length of 68.57 nt and an N50 length of 63 nt, 148,291 transcripts with a mean length of 971.37 nt and an N50 length of 1828 nt and identified 80,761 unigenes with a mean length of 722.82 nt and an N50 length of 1495 nt (Table 1). The size distribution analysis of the unigenes indicated that 14,484 unigenes were larger than 1000 nt in length, which represented 17.93% of all unigenes (S1 Fig). All of the clean data used in this study were uploaded to SRA with the accession number SRS1782539 to SRS1782550 (male antennae: SRS1782539, SRS1782546 and SRS1782548; female antennae: SRS1782540, SRS1782545 and SRS1782550; legs: SRS1782541, SRS1782544 and SRS1782547; larvae: SRS1782542, SRS1782543 and SRS1782549). Most assembled unigene sequences were uploaded to GeneBank with the accession number GFCJ01000001 to GFCJ01079039. The accession numbers of 131 candidate chemosensory genes identified in this study were listed in supporting information (S4 Table).

**Table 1:** Transcriptome assembly summary of *L*. *sticticalis*.

Statistics item	Total Number	Total Length(nt)	Mean Length(nt)	N50	Q30(%)
Clean reads	86,930,000				>85.01
Contigs	3,266,885	224,022,716	69	63	
Unigenes	80,761	58,375,997	723	1495	
Transcripts	148,291	144,044,980	971	1828	

Note: Q30: the percentage of sequences with sequencing error rate lower than 0.1%.

### Nr homology analysis and Gene Ontology (GO) annotation

Of the 80,761 unigenes, the results of annotation by NCBI BLASTx showed that 30,581 (37.87%) unigenes matched to known proteins. The remaining unigenes failed to match any sequence, with an e-value < 1e-5, in neither the Nr nor the Swiss-Prot databases. Among the Nr homology annotated unigenes, 49.62% of the homologous species had best blast match to Lepidopteran sequences. The highest match percentage (28.12%) was to *Bombyx mori* sequences followed by *Danaus plexippus* (20.09%) and *Papilio xuthus* (1.41%) (S2 Fig). Of the Nr annotated unigenes, 62.01% of the unigenes showed strong homology, with an e-value < 1e-45.

**Fig 1:**
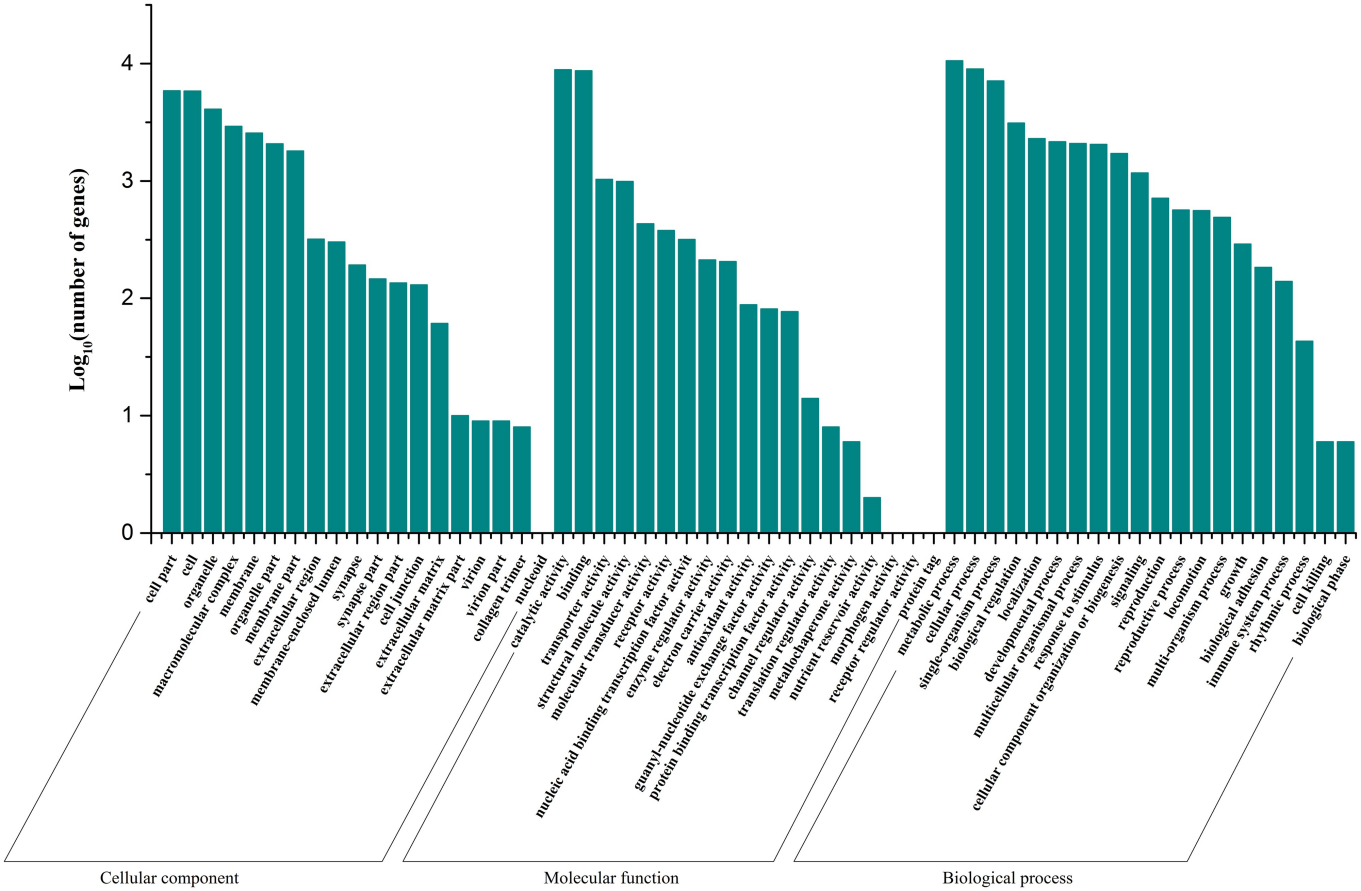
Gene ontology classified annotation of the *L*. *sticticalis* unigenes.

Gene ontology (GO) annotation of the unigenes was acquired using the Blast2GO pipeline according to the BLASTx search against Nr, which was used to classify transcripts into functional groups according to the GO category. Of the 80,761 unigenes, 16,899 (20.92%) unigenes were assigned to the various GO terms. Among the 16,899 GO annotated unigenes, the unigenes were allocated to the biological process terms more than the molecular function terms or the cellular component terms. In the molecular function category, the genes expressed in the antennae were mostly enriched for molecular binding activity (e.g., nucleotide, ion and odorant binding) and catalytic activity (e.g., hydrolase and oxidoreductase). In the biological process category, cellular, metabolic and single-organism processes were the most represented. In the cellular component category, cell, cell part and organelle were the most abundant groups (Fig 1). These results are comparable to the reported *Chilo suppressalis* transcriptional profile [21].

### Identification and expression of candidate ORs of *L*. *sticticalis*

In this study, we identified 54 candidate ORs in *L*. *sticticalis* by bioinformatics analysis. Of these, 38 unigenes had full-length ORFs that encoded 325 to 474 amino acids, and 16 unigenes were partial sequences by the NCBI BLASTp analysis. The 54 OR sequences had a BLASTx best hit to Lepidopteran sequences, with an e-value < 1e-5 (Table 2). Using the TMHMM Server v. 2.0, we also detected 54 candidate OR sequences with 0–8 transmembrane domains (TMDs).

**Table 2:** Unigenes of canidate ORs.

Gene name	Length (nt)	ORF (aa)	Unigene reference	Status	TMD (No.)	Evalue	Ident	BLASTp best hit
LstiOrco	2156	474	c57376_g0	Complete ORF	7	0.00E+00	91%	gi|163845598|gb|ABU45983.2| odorant receptor Or83b [*Helicoverpa assulta*]
LstiPR1	2598	325	c52064_g0	Complete ORF	3	5.00E-162	66%	gi|319918821|dbj|BAJ61939.1| odorant receptor [*Ostrinia nubilalis*]
LstiPR2	2224	420	c53597_g0	Complete ORF	8	0.00E+00	73%	gi|284448851|gb|ADB89183.1| odorant receptor 6 [*Ostrinia nubilalis*]
LstiPR3	2537	374	c55412_g0	5',3'lost	7	3.00E-131	57%	gi|205361596|dbj|BAG71417.1| olfactory receptor-1 [*Diaphania indica*]
LstiPR4	1796	435	c55184_g0	Complete ORF	5	2.00E-161	53%	gi|459958445|gb|AGG91649.1| odorant receptor [*Ostrinia furnacalis*]
LstiPR5	1430	364	c49318_g0	Complete ORF	5	8.00E-162	60%	gi|319918797|dbj|BAJ61929.1| odorant receptor [*Ostrinia nubilalis*]
LstiOR1	1741	342	c52219_g0	Complete ORF	5	0.00E+00	85%	gi|803378049|dbj|BAR43488.1| putative olfactory receptor 46 [*Ostrinia furnacalis*]
LstiOR2	1480	430	c51480_g0	Complete ORF	6	3.00E-173	54%	gi|697993562|gb|AIT69907.1| olfactory receptor 64 [*Ctenopseustis herana*]
LstiOR3	1361	397	c48813_g0	Complete ORF	6	0.00E+00	66%	gi|749692081|gb|AJF23797.1| olfactory receptor OR29 [*Planotortrix octo*]
LstiOR4	1758	255	c50161_g0	3'lost	4	4.00E-109	63%	gi|666916157|gb|AIG51873.1| odorant receptor [*Helicoverpa armigera*]
LstiOR5	2780	375	c57796_g0	Complete ORF	6	6.00E-160	59%	gi|357605671|gb|EHJ64733.1| olfactory receptor 18 [*Danaus plexippus*]
LstiOR6	1343	396	c52421_g0	Complete ORF	6	9.00E-155	56%	gi|803377987|dbj|BAR43474.1| putative olfactory receptor 32 [*Ostrinia furnacalis*]
LstiOR7	1736	408	c54915_g0	Complete ORF	3	0.00E+00	79%	gi|803378017|dbj|BAR43495.1| putative olfactory receptor 53 [*Ostrinia furnacalis*]
LstiOR8	1608	406	c53013_g0	Complete ORF	6	3.00E-160	57%	gi|803377953|dbj|BAR43457.1| putative olfactory receptor 15 [*Ostrinia furnacalis*]
LstiOR9	1638	392	c53531_g0	Complete ORF	6	5.00E-179	59%	gi|803377979|dbj|BAR43470.1| putative olfactory receptor 28 [*Ostrinia furnacalis*]
LstiOR10	1417	401	c48406_g0	Complete ORF	6	0.00E+00	92%	gi|803377977|dbj|BAR43469.1| putative olfactory receptor 27 [*Ostrinia furnacalis*]
LstiOR11	914	304	c55922_g0	3'lost	5	3.00E-72	45%	gi|803377961|dbj|BAR43461.1| putative olfactory receptor 19 [*Ostrinia furnacalis*]
LstiOR12	1413	372	c53849_g0	Complete ORF	5	0.00E+00	85%	gi|803377959|dbj|BAR43460.1| putative olfactory receptor 18 [*Ostrinia furnacalis*]
LstiOR13	2817	296	c52168_g0	5',3'lost	3	6.00E-121	60%	gi|182509192|ref|NP_001116807.1| olfactory receptor 39 [*Bombyx mori*]
LstiOR14	4200	416	c58276_g0	Complete ORF	4	0.00E+00	70%	gi|803377951|dbj|BAR43456.1| putative olfactory receptor 14 [*Ostrinia furnacalis*]
LstiOR15	1891	407	c56008_g0	Complete ORF	5	2.00E-159	52%	gi|698029530|gb|AIT71984.1| olfactory receptor 10 [*Ctenopseustis obliquana*]
LstiOR16	1438	416	c52751_g0	Complete ORF	7	0.00E+00	81%	gi|803377967|dbj|BAR43464.1| putative olfactory receptor 22 [*Ostrinia furnacalis*]
LstiOR17	1028	301	c52003_g0	5',3'lost	5	2.00E-129	64%	gi|803378045|dbj|BAR43486.1| putative olfactory receptor 44 [*Ostrinia furnacalis*]
LstiOR18	1513	437	c53294_g0	Complete ORF	5	0.00E+00	69%	gi|803377991|dbj|BAR43476.1| putative olfactory receptor 34 [*Ostrinia furnacalis*]
LstiOR19	1430	413	c53715_g0	Complete ORF	7	0.00E+00	73%	gi|333408659|gb|AEF32141.1| odorant receptor [*Spodoptera exigua*]
LstiOR20	1688	400	c46193_g0	Complete ORF	4	0.00E+00	79%	gi|803377963|dbj|BAR43462.1| putative olfactory receptor 20 [*Ostrinia furnacalis*]
LstiOR21	1499	365	c49860_g0	5'lost	4	0.00E+00	94%	gi|803377993|dbj|BAR43477.1| putative olfactory receptor 35 [*Ostrinia furnacalis*]
LstiOR22	1321	255	c53072_g0	5'lost	3	4.00E-155	85%	gi|803377949|dbj|BAR43455.1| putative olfactory receptor 13 [*Ostrinia furnacalis*]
LstiOR23	1777	401	c51775_g0	Complete ORF	6	3.00E-145	51%	gi|803378001|dbj|BAR43481.1| putative olfactory receptor 39 [*Ostrinia furnacalis*]
LstiOR24	1147	300	c52154_g0	5'lost	3	1.00E-142	86%	gi|803377975|dbj|BAR43468.1| putative olfactory receptor 26 [*Ostrinia furnacalis*]
LstiOR25	1596	393	c52246_g0	Complete ORF	7	7.00E-97	39%	gi|803377943|dbj|BAR43452.1| putative olfactory receptor 10 [*Ostrinia furnacalis*]
LstiOR26	2121	430	c55854_g1	Complete ORF	6	3.00E-140	48%	gi|749692127|gb|AJF23820.1| olfactory receptor OR64 [*Planotortrix octo*]
LstiOR27	1499	392	c55222_g0	Complete ORF	6	0.00E+00	82%	gi|803377983|dbj|BAR43472.1| putative olfactory receptor 30 [*Ostrinia furnacalis*]
LstiOR28	1904	365	c53069_g0	3'lost	3	3.00E-108	53%	gi|803377955|dbj|BAR43458.1| putative olfactory receptor 16 [*Ostrinia furnacalis*]
LstiOR29	1629	363	c52605_g0	5'lost	6	3.00E-128	52%	gi|697993564|gb|AIT69908.1| olfactory receptor 66 [*Ctenopseustis herana*]
LstiOR30	1421	376	c52897_g0	3'lost	6	1.00E-139	64%	gi|698029528|gb|AIT71983.1| olfactory receptor 9 [*Ctenopseustis obliquana*]
LstiOR31	817	230	c50161_g1	3'lost	3	6.00E-81	54%	gi|666916157|gb|AIG51873.1| odorant receptor [*Helicoverpa armigera*]
LstiOR32	2177	437	c55203_g0	Complete ORF	2	1.00E-150	55%	gi|666916161|gb|AIG51875.1| odorant receptor [*Helicoverpa armigera*]
LstiOR33	1358	388	c50480_g0	Complete ORF	7	4.00E-178	65%	gi|803377985|dbj|BAR43473.1| putative olfactory receptor 31 [*Ostrinia furnacalis*]
LstiOR34	5504	423	c59969_g0	Complete ORF	4	0.00E+00	71%	gi|803377981|dbj|BAR43471.1| putative olfactory receptor 29 [*Ostrinia furnacalis*]
LstiOR35	1467	390	c50674_g0	Complete ORF	7	0.00E+00	83%	gi|803377997|dbj|BAR43479.1| putative olfactory receptor 37 [*Ostrinia furnacalis*]
LstiOR36	1954	382	c55053_g0	Complete ORF	6	0.00E+00	67%	gi|803378047|dbj|BAR43487.1| putative olfactory receptor 45 [*Ostrinia furnacalis*]
LstiOR37	1279	389	c49794_g0	Complete ORF	6	0.00E+00	79%	gi|803377945|dbj|BAR43453.1| putative olfactory receptor 11 [*Ostrinia furnacalis*]
LstiOR38	1169	376	c52410_g0	Complete ORF	6	0.00E+00	76%	gi|803378005|dbj|BAR43483.1| putative olfactory receptor 41 [*Ostrinia furnacalis*]
LstiOR39	1473	392	c50614_g0	Complete ORF	5	2.00E-153	51%	gi|669092476|gb|AII01110.1| odorant receptor [*Dendrolimus kikuchii*]
LstiOR40	2678	448	c49183_g0	Complete ORF	0	0.00E+00	70%	gi|357628941|gb|EHJ78030.1| olfactory receptor 29 [*Danaus plexippus*]
LstiOR41	2931	408	c56510_g0	Complete ORF	6	0.00E+00	69%	gi|803378015|dbj|BAR43494.1| putative olfactory receptor 52 [*Ostrinia furnacalis*]
LstiOR42	1365	400	c51381_g0	Complete ORF	5	0.00E+00	67%	gi|803377999|dbj|BAR43480.1| putative olfactory receptor 38 [*Ostrinia furnacalis*]
LstiOR43	1487	418	c47710_g0	Complete ORF	5	2.00E-178	57%	gi|803377955|dbj|BAR43458.1| putative olfactory receptor 16 [*Ostrinia furnacalis*]
LstiOR44	1695	405	c51607_g0	Complete ORF	7	0.00E+00	82%	gi|803377973|dbj|BAR43467.1| putative olfactory receptor 25 [*Ostrinia furnacalis*]
LstiOR45	657	196	c44707_g0	5'lost	2	6.00E-77	62%	gi|486139804|gb|AGK90015.1| olfactory receptor 7 [*Helicoverpa assulta*]
LstiOR46	652	216	c45601_g1	3'lost	4	8.00E-80	60%	gi|803377985|dbj|BAR43473.1| putative olfactory receptor 31 [*Ostrinia furnacalis*]
LstiOR47	780	223	c42299_g0	5'lost	3	2.00E-56	43%	gi|698029599|gb|AIT72018.1| olfactory receptor 67 [*Ctenopseustis obliquana*]
LstiOR48	459	125	c9294_g0	5'lost	0	2.00E-21	47%	gi|357628292|gb|EHJ77681.1| olfactory receptor 4 [*Danaus plexippus*]

**Fig 2:**
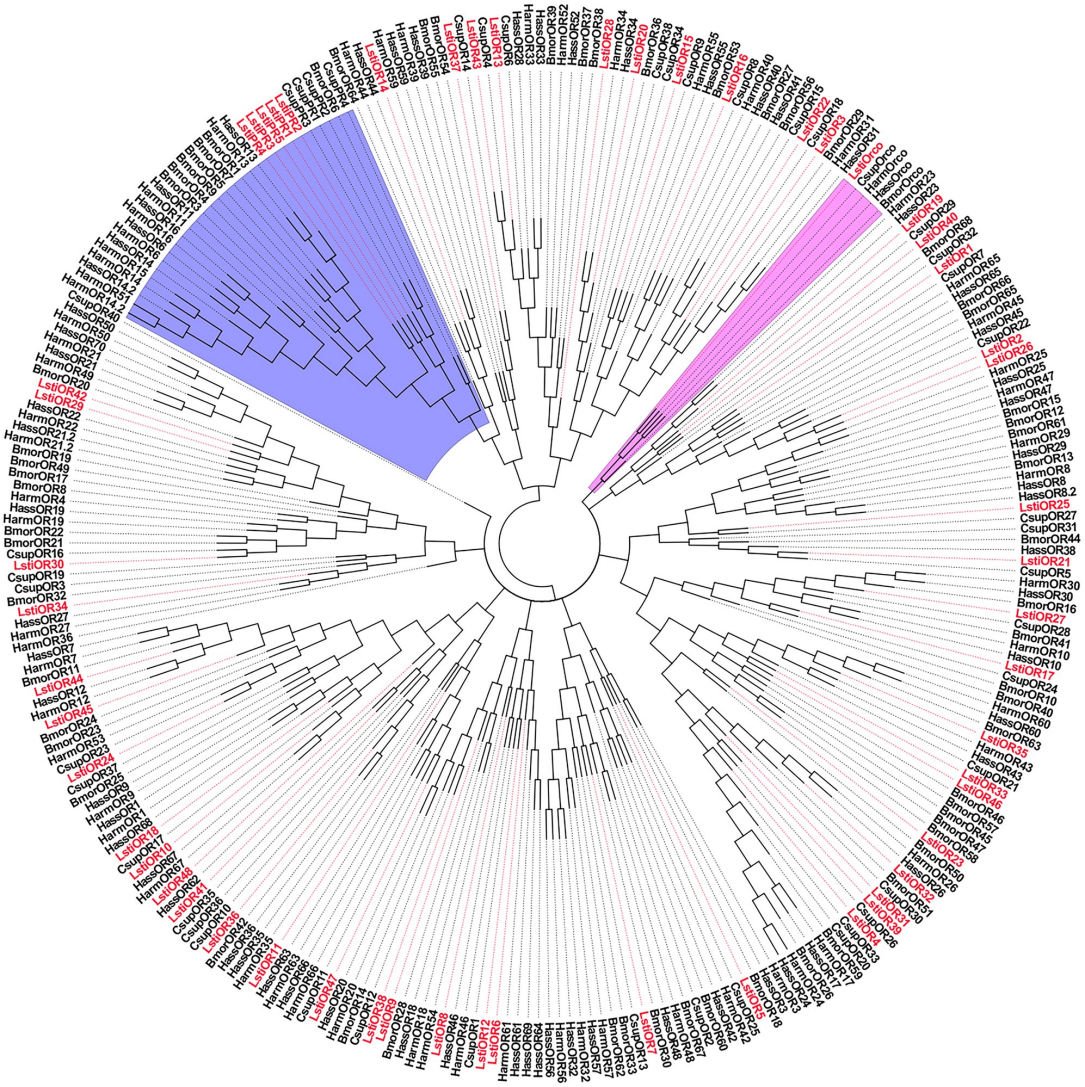
Phylogenetic tree of candidate LstiORs with known lepidopteran ORs. Csup: *C*. *suppressalis*, Bmor: *B*. *mori*, Harm: *H*. *armigera*, Hass: *H*. *assulta*. The clade in blue indicates the PR gene clade; the clade in pink indicates the Orco clade.

**Fig 3:**
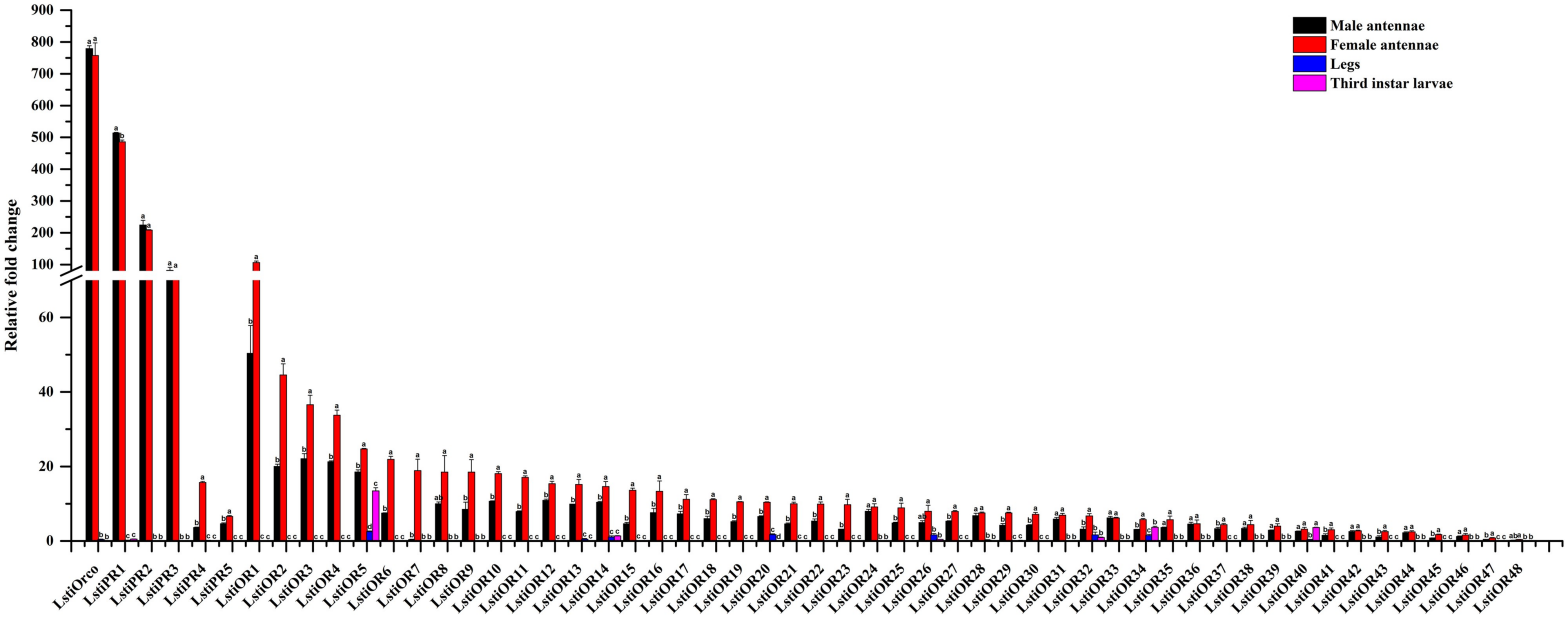
Expression pattern of *L*. *sticticalis* ORs by RT-qPCR. Legs (male: female = 1:1). *β-actin* was used as an internal reference gene to test the integrity of each cDNA template. The standard error is represented by the error bar, and the different letters (a, b, c) above each bar represent significant differences (p < 0.05).

The unigene C57376.g0 was named LstiOrco due to the high level of identity with the conserved Orco proteins of other insect species in Lepidoptera, which was clustered into the Orco clades of Lepidoptera in the phylogenetic tree (Fig 2). Among the 54 candidate LstiORs, *LstiOrco* showed the highest expression levels in the antennae in both RNA-Seq and RT-qPCR analysis (Fig 3).

Five unigenes, named “LstiPRm” (m = 1 to 5), were considered to be pheromone receptors (PRs) because they shared considerable similarity with previously characterized Lepidopteran PRs and were clustered together into one subgroup in the phylogenetic tree (Fig 2). For the relatively conserved PR genes, LstiPR1 and LstiPR2 were clustered together with PR 1, 2, 3 and 4 in *C*. *suppressalis*. LstiPR3, 4 and 5 were not closely grouped with the Pyralidae PRs but clustered with the *B*. *mori*, *H*. *armigera* and *H*. *assulta* PR clade with high bootstrap support (Fig 2). The five LstiPRs showed higher expression in the antennae of both sexes than in the legs and larvae (p < 0.05) (Fig 3).

The remaining 48 LstiOR unigenes were highly divergent, which is common for insect olfactory receptor genes. These unigenes were named “LstiORn” (n = 1 to 48), followed by a numeral, in descending order in accordance with their female antennal expression levels. The RT-qPCR results showed that 47 candidate LstiORs had antennae-enriched expression, and 33 candidate LstiORs (*OR1-23*, *OR25*, *OR27*, *OR29*, *OR30*, *OR32*, *OR34*, *OR41*, *OR43*, *OR45* and *OR47*) had female antennae-biased expression, especially for *LstiOR7* being female specific. But, the putative *LstiOR40* was richly expressed in the antennae and larvae (Fig 3).

### Identification and expression of candidate IRs and GRs of *L*. *sticticalis*

Based on bioinformatic analysis, we identified 18 candidate IR sequences in *L*. *sticticalis*. Ten sequences contained full-length open reading frames (ORFs), and the remaining 8 sequences were marked as incomplete because they lacked a complete 5' or 3' terminus. Seventeen putative IRs in *L*. *sticticalis* were predicted to have 1–4 TMDs by TMHMM Server v. 2.0 (Table 3).

**Table 3:** Unigenes of candidate IRs and GRs.

Gene name	Length (nt)	ORF (aa)	Unigene reference	Status	TMD (No.)	Evalue	Ident	BLASTp best hit
*L*. *sticticalis* IR								
LstiIR1	3082	599	c53104_g0	Complete ORF	1	0.00E+00	55%	gi|666916271|gb|AIG51930.1| ionotropic glutamate receptor [*Helicoverpa armigera*]
LstiIR7d.2	3283	509	c57698_g0	5'lost	3	3.00E-65	50%	gi|666916245|gb|AIG51917.1| ionotropic receptor, partial [*Helicoverpa armigera*]
LstiIR7d.3	3124	908	c56115_g0	Complete ORF	3	0.00E+00	71%	gi|666916269|gb|AIG51929.1| ionotropic glutamate receptor [*Helicoverpa armigera*]
LstiIR7g	579	161	c57960_g1	3'lost	0	2.00E-62	67%	gi|666916261|gb|AIG51925.1| ionotropic glutamate receptor [*Helicoverpa armigera*]
LstiIR8a	5900	907	c60034_g0	5'lost	4	0.00E+00	90%	gi|814544210|dbj|BAR64796.1| ionotropic receptor [*Ostrinia furnacalis*]
LstiIR21a	2890	497	c57834_g0	5'lost	4	0.00E+00	90%	gi|814544212|dbj|BAR64797.1| ionotropic receptor [*Ostrinia furnacalis*]
LstiIR25a	3281	925	c56710_g0	Complete ORF	3	0.00E+00	97%	gi|814544214|dbj|BAR64798.1| ionotropic receptor [*Ostrinia furnacalis*]
LstiIR40a	2726	719	c55259_g0	Complete ORF	3	0.00E+00	93%	gi|814544216|dbj|BAR64799.1| ionotropic receptor [*Ostrinia furnacalis*]
LstiIR41a	1634	433	c56539_g0	3'lost	1	0.00E+00	79%	gi|814544218|dbj|BAR64800.1| ionotropic receptor [*Ostrinia furnacalis*]
LstiIR64a	2034	607	c54099_g0	Complete ORF	3	0.00E+00	80%	gi|814544220|dbj|BAR64801.1| ionotropic receptor [*Ostrinia furnacalis*]
LstiIR68a	3972	701	c57364_g0	Complete ORF	4	0.00E+00	71%	gi|313505776|gb|ADR64682.1| putative chemosensory ionotropic receptor IR68a [*Spodoptera littoralis*]
LstiIR75d	3796	525	c59316_g0	5'lost	3	0.00E+00	56%	gi|313505778|gb|ADR64683.1| putative chemosensory ionotropic receptor IR75d, partial [*Spodoptera littoralis*]
LstiIR75p	1836	245	c57651_g0	5'lost	2	2.00E-125	86%	gi|814544228|dbj|BAR64805.1| ionotropic receptor [*Ostrinia furnacalis*]
LstiIR75p.1	1831	373	c57266_g0	5'lost	3	0.00E+00	93%	gi|814544232|dbj|BAR64807.1| ionotropic receptor [*Ostrinia furnacalis*]
LstiIR75q.2	4322	640	c59586_g0	Complete ORF	3	0.00E+00	88%	gi|814544234|dbj|BAR64808.1| ionotropic receptor [*Ostrinia furnacalis*]
LstiIR76b	2193	547	c56375_g0	Complete ORF	3	0.00E+00	86%	gi|814544236|dbj|BAR64809.1| ionotropic receptor [*Ostrinia furnacalis*]
LstiIR87a	2630	652	c55166_g0	Complete ORF	3	0.00E+00	91%	gi|814544238|dbj|BAR64810.1| ionotropic receptor [*Ostrinia furnacalis*]
LstiIR93a	2808	873	c56170_g0	Complete ORF	3	0.00E+00	89%	gi|814544240|dbj|BAR64811.1| ionotropic receptor [*Ostrinia furnacalis*]
*L*. *sticticalis* GR								
LstiGR1	1512	456	c50908_g0	Complete ORF	7	0.00E+00	74%	gi|486139901|gb|AGK90023.1| gustatory receptor 1 [*Helicoverpa assulta*]
LstiGR4	1584	433	c53093_g0	Complete ORF	6	0.00E+00	72%	gi|486139682|gb|AGK90011.1| gustatory receptor 4 [*Helicoverpa armigera*]
LstiGR5a	1759	403	c51915_g0	3'lost	6	3.00E-148	55%	gi|486139707|gb|AGK90012.1| gustatory receptor 5 [*Helicoverpa armigera*]
LstiGR5b	813	188	c52834_g0	3'lost	2	6.00E-52	50%	gi|486139707|gb|AGK90012.1| gustatory receptor 5 [*Helicoverpa armigera*]
LstiGR6	335	110	c3705_g0	5',3'lost	2	5.00E-05	32%	gi|217416194|tpg|DAA06379.1| gustatory receptor 16 [*Bombyx mori*]
LstiGR7	653	188	c52834_g1	5',3'lost	2	7.00E-26	34%	gi|486139927|gb|AGK90025.1| gustatory receptor 5 [*Helicoverpa assulta*]
LstiGR21a	1590	457	c49914_g0	Complete ORF	6	0.00E+00	86%	gi|666916225|gb|AIG51907.1| gustatory receptor [*Helicoverpa armigera*]
LstiGR21b	1081	305	c41631_g0	5'lost	5	1.00E-101	89%	gi|666916227|gb|AIG51908.1| gustatory receptor [*Helicoverpa armigera*]
LstiGR45	392	119	c21748_g0	5'lost	1	2.00E-19	44%	gi|195963347|ref|NP_001124346.1| gustatory receptor 45 [*Bombyx mori*]
LstiGR51	402	122	c4938_g0	5'lost	2	2.00E-36	53%	gi|217416213|tpg|DAA06388.1| gustatory receptor 51 [*Bombyx mori*]
LstiGR63a	543	127	c28880_g0	5',3'lost	2	1.00E-17	44%	gi|217416227|tpg|DAA06395.1|gustatory receptor 63 [*Bombyx mori*]
LstiGR63a.1	1711	428	c50350_g0	5'lost	7	2.00E-35	33%	gi|217416227|tpg|DAA06395.1| gustatory receptor 63 [*Bombyx mori*]
LstiGR63a.2	1375	435	c47120_g0	3'lost	1	1.00E-49	43%	gi|746873808|gb|AJD81603.1| gustatory receptor 10, partial [*Helicoverpa assulta*]

**Fig 4:**
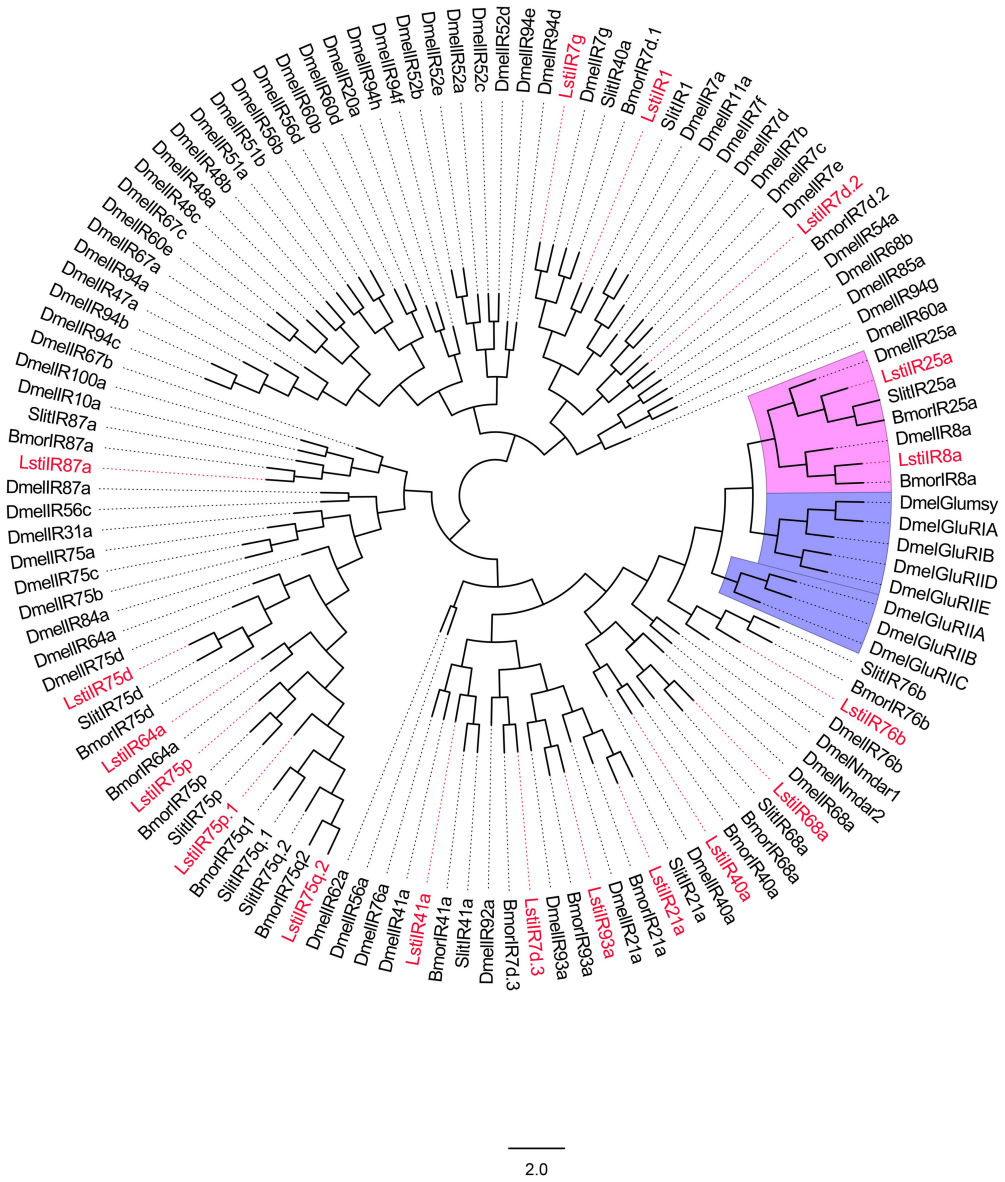
Phylogenetic tree of candidate LstiIRs with known lepidopteran IRs and iGluRs. Dmel: *D*. *melanogaster*, Bmor: *B*. *mori*, Slit: *S*. *littoralis*. The clade in blue indicates the iGluR gene clade; the clade in pink indicates the IR8a and IR25a clade.

A phylogenetic tree of the LstiIRs was constructed based on the amino acid sequences from *L*. *sticticalis*, *Drosophila melanogaster*, *B*. *mori* and *S*. *littoralis* (Fig 4). The neighbor-joining tree analysis showed a clear segregation between Dmel ionotropic glutamate receptors (iGluRs) and insect IRs, and 18 LstiIR candidates were clustered to antennal IRs and the IR25a/IR8a clades, but did not belong to DmeliGluRs. According to their BLASTx best hits to Lepidopteran IRs and their positions in the phylogenetic tree, the 18 candidate IRs were given names consistent with the number and suffix of the Dmel/Bmor/Slit IR orthologs in the same clade (Table 3).

**Fig 5:**
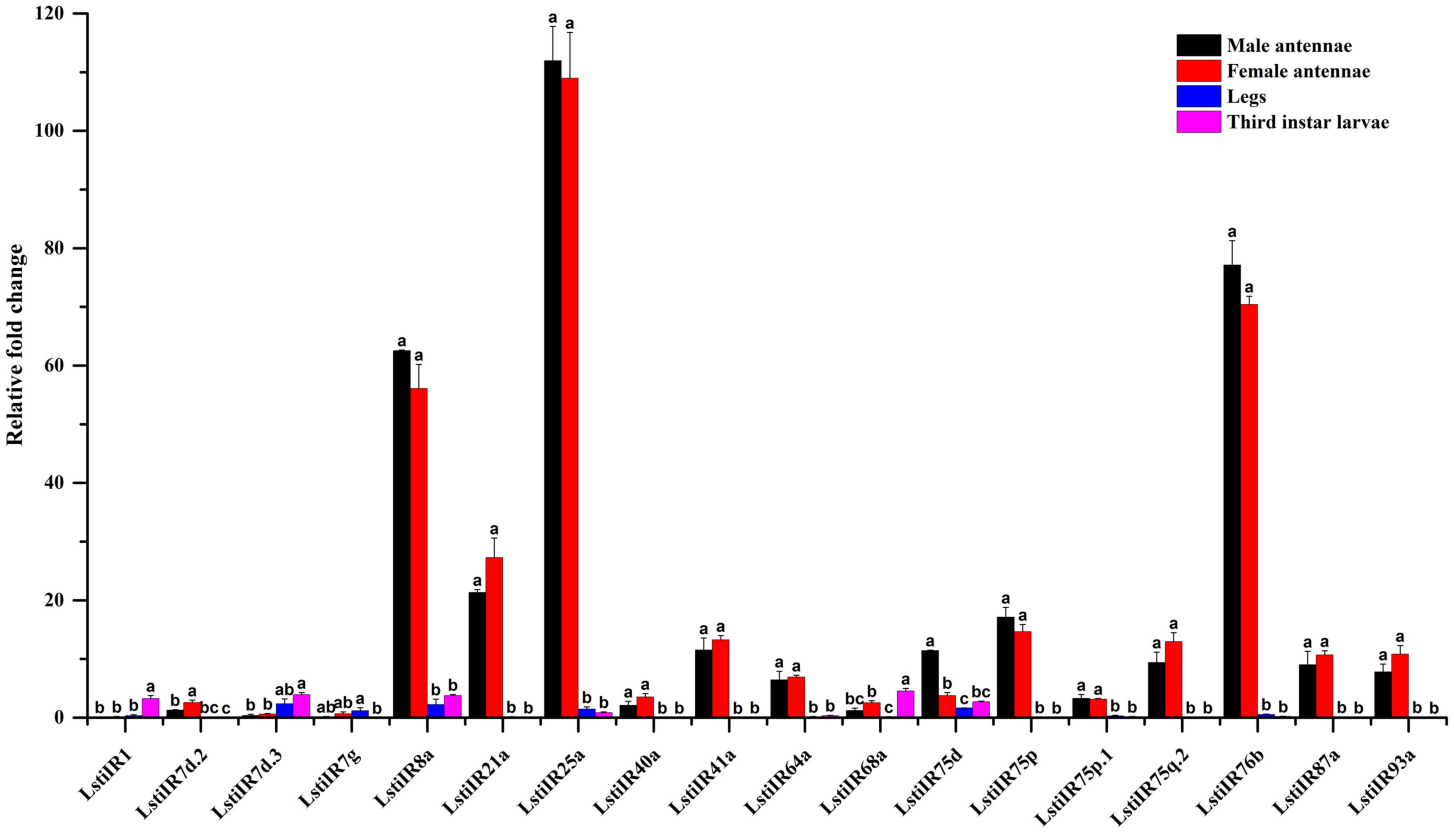
Expression pattern of *L*. *sticticalis* IRs by RT-qPCR. The details were same as mentioned in Fig 3.

Of the 18 named LstiIR candidates, the RT-qPCR results showed 10 putative LstiIRs (*7d*.*2*, *21a*, *40a*, *41a*, *64a*, *75p*, *75p*.*1*, *75q*.*2*, *87a*, and *93a*) showed antennae specific expression, and expression levels of *8a*, *25a*, *75d* and *76b* were higher in the antennae than in the legs and larvae (p < 0.05). But the *LstiIR1* showed larvae specific expression, *LstiIR7d*.*3* and *68a* in the larvae and *LstiIR7g* in the legs had higher expression than in the antennae (Fig 5).

**Fig 6:**
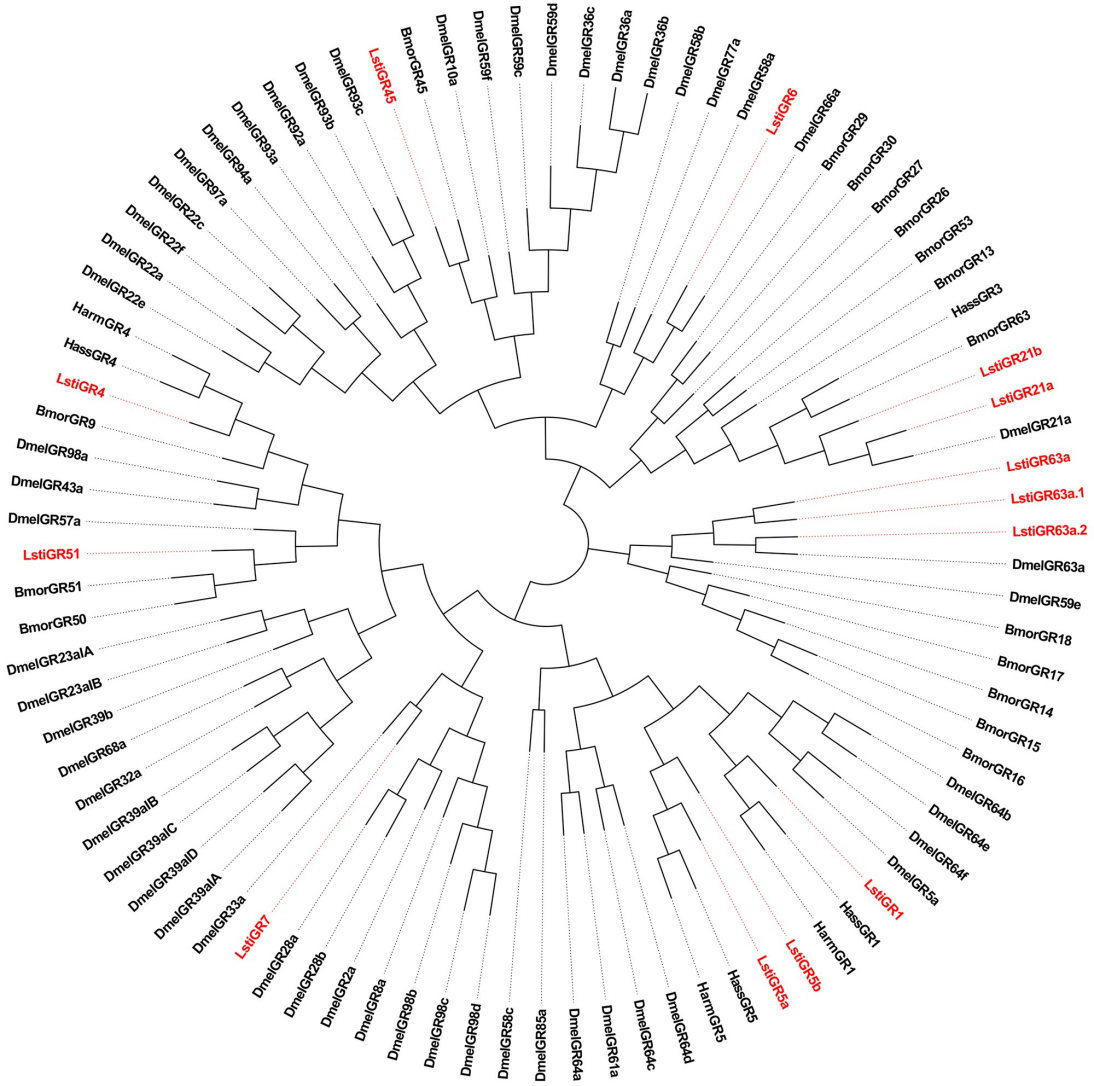
Phylogenetic tree of candidate LstiGRs with known lepidopteran GRs. Dmel: *D*. *melanogaster*, Bmor: *B*. *mori*, Harm: *H*. *armigera* and Hass: *H*. *assulta*.

**Fig 7:**
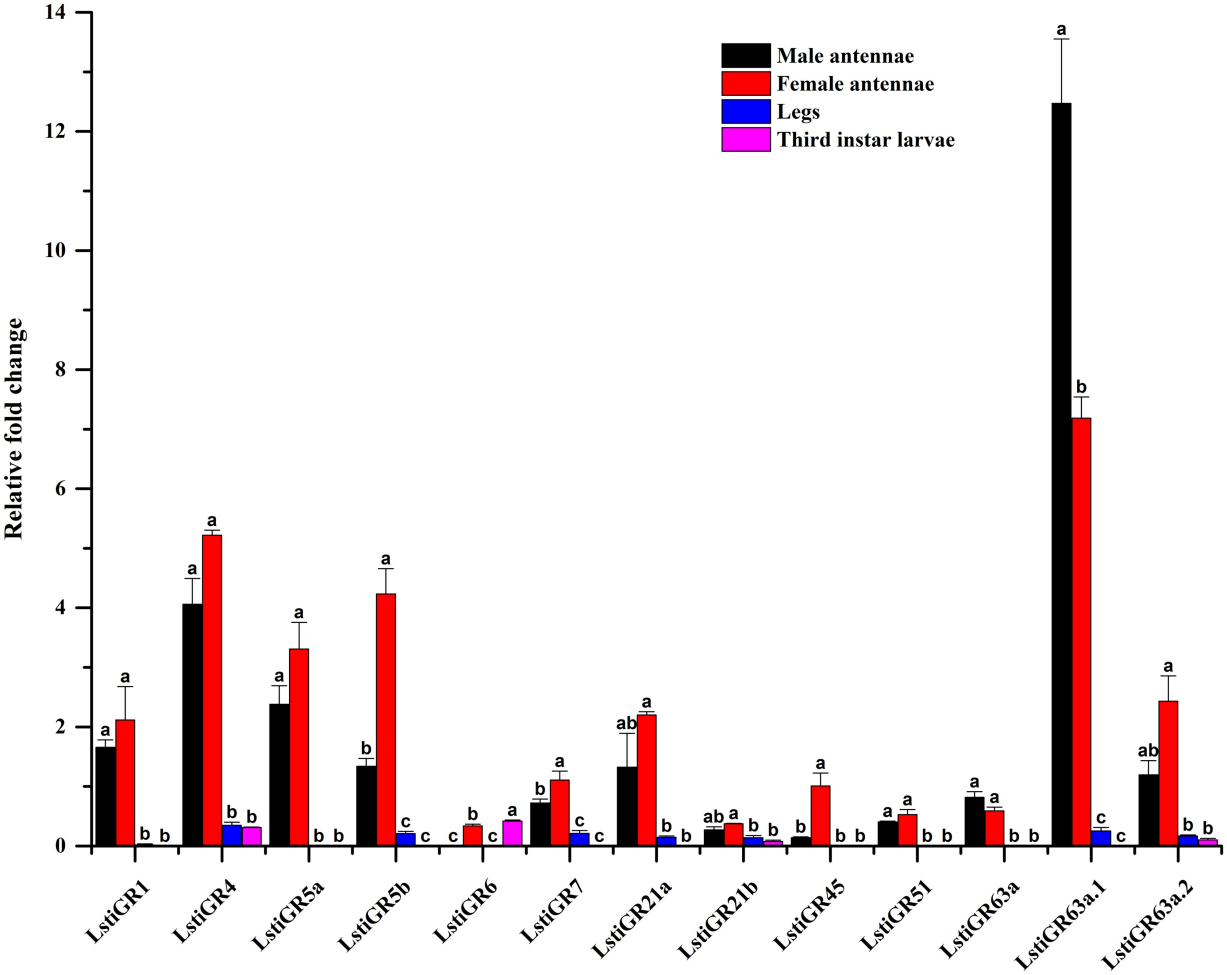
Expression pattern of *L*. *sticticalis* GRs by RT-qPCR. The details were same as mentioned in Fig 3.

In total, we identified 13 GR candidates in *L*. *sticticalis*, including 3 unigenes with full-length ORFs and 10 unigenes with partial sequences. Thirteen putative GRs were predicted to have 1–7 transmembrane domains (Table 3). Of the 13 putative LstiGRs, 11 sequences were named based on their clustering into the clades of Dmel/Bmor/Hass/Harm GRs in the phylogenetic tree (Fig 6). Two unigenes (C52834.g1 and C3705.g0) had low bootstrap values and were unable to be placed on the phylogenetic with confidence and were named LstiGR6 and LstiGR7, respectively. The RT-qPCR results showed that 13 candidate LstiGRs were enriched in the antennae and the expression amounts of *LstiGR63a*.*1* in the male antennae was the highest. Interestingly, the putative *LstiGR6* was sex-specific expressed in the female antennae, but also expressed in the larvae (Fig 7).

### Identification and expression of putative OBPs of *L*. *sticticalis*

In the process of identification of putative OBPs, we used not only keyword searching by PSI-BLAST, but also motif scanning to detect the conserved six cysteine residue pattern, which is C1-X5-39-C2-X3-C3-X21-44-C4-X7-12-C5-X8-C6 [19], in the sequence of OBPs. In all, we identified 34 candidate OBPs in *L*. *sticticalis*, including 3 PBPs and 1 GOBP. The results of the sequence analysis showed 23 unigenes with full–length ORFs and the remaining 11 unigenes corresponding partial sequences. Among the 34 putative LstiOBPs, 22 unigenes were predicted to have signal peptides by SignalP 4.1 Server analysis. These 34 OBP sequences had a BLASTx best hits to Lepidopteran sequences with an e-value < 1e-5 (Table 4).

**Table 4:** Unigenes of candidate OBPs.

Gene name	Length (nt)	ORF (aa)	Unigene reference	Status	Signal Peptide	Evalue	Ident	BLASTp best hit	Group
LstiPBP1	1094	172	c59843_g0	Complete ORF	Y	3.00E-86	72%	gi|315075439|gb|ADT78501.1| pheromone binding protein 2 [*Ostrinia furnacalis*]	Classic
LstiPBP2	1263	83	c52747_g0	5'lost	N	7.00E-37	100%	gi|194320500|gb|ACF48468.1| pheromone binding protein female 2, partial [*Loxostege sticticalis*]	-
LstiPBP3	1116	163	c52060_g0	Complete ORF	Y	2.00E-115	99%	gi|188998306|gb|ACD67881.1| pheromone-binding protein [*Loxostege sticticalis*]	Classic
LstiGOBP1	2187	140	c58964_g0	Complete ORF	N	8.00E-98	99%	gi|172041802|gb|ACB47481.1| general odorant binding protein 1, partial [*Loxostege sticticalis*]	Classic
LstiOBP1	4099	140	c54427_g2	Complete ORF	Y	8.00E-72	83%	gi|507155159|gb|AGM38607.1| odorant binding protein [*Chilo suppressalis*]	Classic
LstiOBP2	837	128	c49708_g0	3'lost	N	6.00E-26	84%	gi|472271932|gb|AGI37366.1| general odorant-binding protein 2 [*Cnaphalocrocis medinalis*]	-
LstiOBP3	4469	122	c60039_g0	Complete ORF	N	1.00E-36	81%	gi|472271924|gb|AGI37362.1| general odorant-binding protein 3 [*Cnaphalocrocis medinalis*]	Classic
LstiOBP4	861	149	c48974_g1	Complete ORF	Y	6.00E-36	48%	gi|469664295|gb|AGH70102.1| odorant binding protein 6 [*Spodoptera exigua*]	Classic
LstiOBP5	1359	166	c56490_g0	3'lost	Y	2.00E-78	70%	gi|290965852|gb|ADD71058.1| odorant-binding protein [*Chilo suppressalis*]	-
LstiOBP6	1053	146	c53701_g0	Complete ORF	Y	5.00E-84	84%	gi|383215092|gb|AFG72998.1| odorant-binding protein 1 [*Cnaphalocrocis medinalis*]	Classic
LstiOBP7	968	133	c51868_g0	Complete ORF	Y	2.00E-74	83%	gi|469664301|gb|AGH70105.1| odorant binding protein 9 [*Spodoptera exigua*]	Minus-C
LstiOBP8	684	106	c49392_g0	3'lost	Y	2.00E-29	54%	gi|614255900|gb|AHX37224.1| odorant binding protein 2 [*Conogethes punctiferalis*]	-
LstiOBP9	4932	243	c59888_g0	Complete ORF	Y	2.00E-80	56%	gi|669092244|gb|AII00994.1| odorant binding protein [*Dendrolimus kikuchii*]	Classic
LstiOBP10	1145	143	c52167_g0	5',3'lost	N	2.00E-11	41%	gi|380085008|gb|AFD34183.1| pheromone binding protein 2 [*Argyresthia conjugella*]	-
LstiOBP11	687	205	c43276_g0	Complete ORF	N	3.00E-58	43%	gi|669092272|gb|AII01008.1| odorant binding protein [*Dendrolimus kikuchii*]	Plus-C
LstiOBP12	1352	330	c48814_g0	Complete ORF	Y	2.00E-78	47%	gi|512911268|ref|XP_004927370.1| PREDICTED: general odorant-binding protein 71 [*Bombyx mori*]	Classic
LstiOBP13	797	136	c47523_g0	Complete ORF	Y	7.00E-54	60%	gi|669092214|gb|AII00979.1| odorant binding protein [*Dendrolimus houi*]	Minus-C
LstiOBP14	638	147	c49381_g0	Complete ORF	Y	9.00E-39	48%	gi|669092242|gb|AII00993.1| odorant binding protein [*Dendrolimus kikuchii*]	Classic
LstiOBP15	1154	185	c51405_g0	Complete ORF	Y	1.00E-122	92%	gi|669092212|gb|AII00978.1| odorant binding protein [*Dendrolimus houi*]	Classic
LstiOBP16	489	122	c45457_g0	Complete ORF	N	1.00E-34	51%	gi|226531141|ref|NP_001140188.1| odorant-binding protein 4 [*Bombyx mori*]	Classic
LstiOBP17	885	259	c47838_g0	Complete ORF	Y	8.00E-69	42%	gi|237648972|ref|NP_001153663.1| odorant binding protein LOC100301495 precursor [*Bombyx mori*]	Minus-C
LstiOBP18	1861	114	c57098_g0	Complete ORF	N	2.00E-27	59%	gi|669092258|gb|AII01001.1| odorant binding protein [*Dendrolimus kikuchii*]	Classic
LstiOBP19	1006	153	c51039_g0	Complete ORF	Y	9.00E-33	37%	gi|237648974|ref|NP_001153664.1| odorant binding protein LOC100301496 precursor [*Bombyx mori*]	Classic
LstiOBP20	2332	128	c57179_g0	5'lost	Y	8.00E-04	28%	gi|909558413|ref|XP_013134219.1| PREDICTED: general odorant-binding protein 68-like [*Papilio polytes*]	-
LstiOBP21	643	144	c45607_g0	Complete ORF	Y	5.00E-35	47%	gi|519767927|gb|AGP03455.1| SexiOBP9 [*Spodoptera exigua*]	Classic
LstiOBP22	556	146	c41600_g0	Complete ORF	Y	1.00E-78	75%	gi|482612754|gb|AGK24580.1| odorant-binding protein 4 [*Chilo suppressalis*]	Plus-C
LstiOBP23	495	68	c23316_g0	5'lost	N	2.00E-14	48%	gi|482612756|gb|AGK24581.1| odorant-binding protein 5 [*Chilo suppressalis*]	-
LstiOBP24	323	93	c65807_g0	5' lost	N	4.00E-27	53%	gi|255652863|ref|NP_001157372.1| odorant binding protein fmxg18C17 precursor [*Bombyx mori*]	-
LstiOBP25	480	122	c38508_g0	5'lost	Y	6.00E-28	46%	gi|255652863|ref|NP_001157372.1| odorant binding protein fmxg18C17 precursor [*Bombyx mori*]	-
LstiOBP26	586	146	c38320_g0	Complete ORF	Y	3.00E-59	66%	gi|324103933|gb|ADY17886.1| odorant binding protein [*Spodoptera exigua*]	Classic
LstiOBP27	439	116	c73123_g0	3'lost	N	6.00E-57	69%	gi|927034300|gb|ALD65894.1| odorant binding protein 20 [*Spodoptera litura*]	-
LstiOBP28	923	157	c48290_g0	Complete ORF	Y	3.00E-17	35%	gi|482612750|gb|AGK24578.1| odorant-binding protein 2 [*Chilo suppressalis*]	Minus-C
LstiOBP29	881	146	c48395_g0	Complete ORF	Y	3.00E-57	62%	gi|324103933|gb|ADY17886.1| odorant binding protein [*Spodoptera exigua*]	Classic
LstiOBP30	213	70	c86797_g0	3'lost	N	1.00E-09	65%	gi|357614207|gb|EHJ68962.1| odorant-binding protein 3 [*Danaus plexippus*]	-

**Fig 8:**
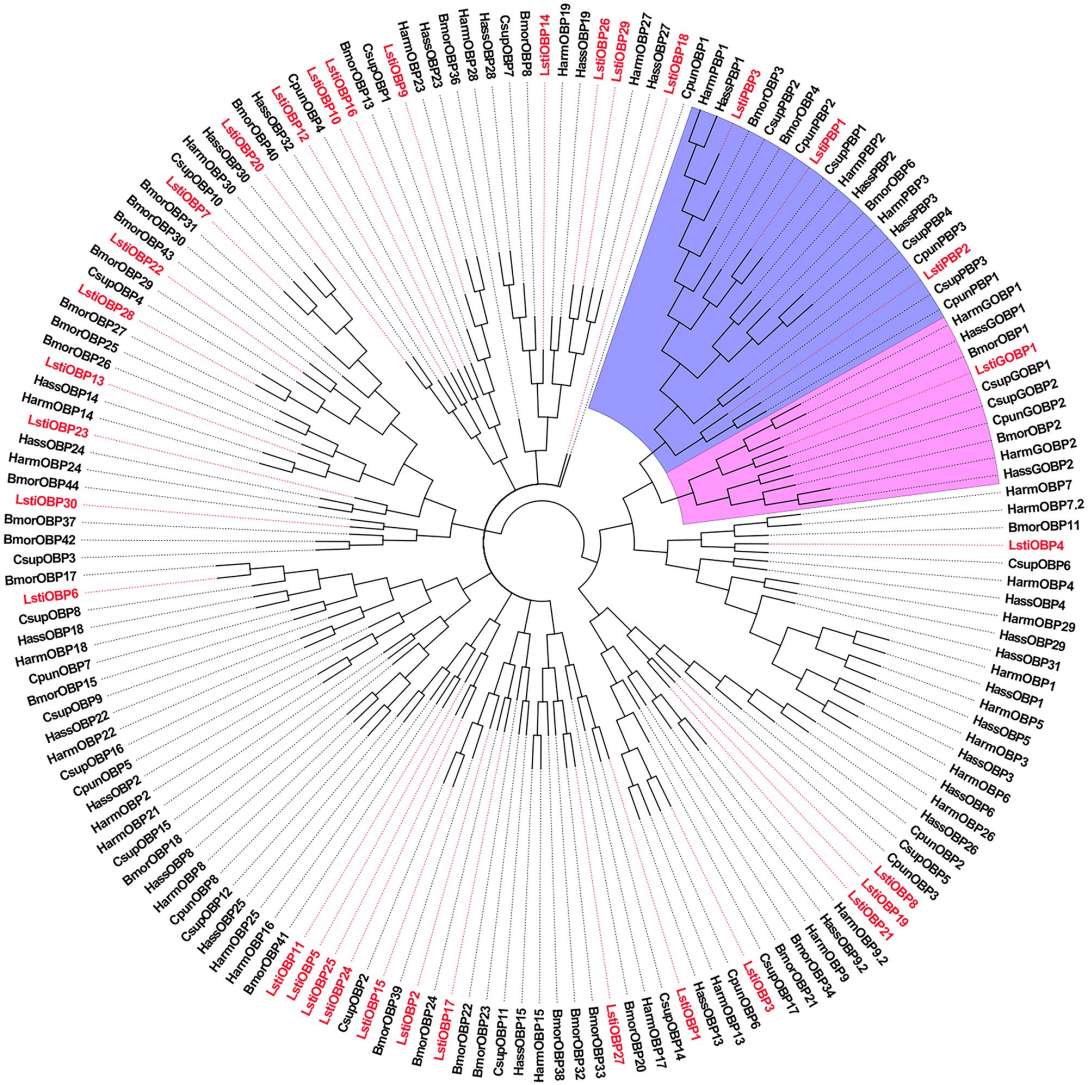
Phylogenetic tree of candidate LstiOBPs with known lepidopteran OBPs. Csup: C. *suppressalis*, Bmor: *B*. *mori*, Harm: *H*. *armigera*, Hass: *H*. *assulta*, Cpun: *C*. *punctiferalis*. The clade in blue indicates the GOBP gene clade; the clade in pink indicates the PBP clade.

Four unigenes (C59843.g0 C52747.g0, C52060.g0 and C58964.g0) were clustered into the PBP and GOBP clades of Lepidoptera in the phylogenetic tree (Fig 8) and were named LstiPBP1, LstiPBP2, LstiPBP3 and LstiGOBP1, respectively. The remaining 30 sequences were named LstiOBP1-30 on the basis of the similarity to known Lepidopteran OBPs and female antennal expression levels. OBPs usually were classified into three phylogenetic families. Classic OBPs, which include the PBP-GOBP group, are characterized by the conserved 6 cysteine residue pattern. The Minus-C class has lost cysteine residues, which are generally C2 and C5, and lysine can replace the position of the lost C2 [15]. In contrast, the Plus-C class has 1–2 extra cysteines and one characteristic proline next to the end of the sixth conserved cysteine residue [5]. The results of our sequence analysis showed that 23 complete ORF OBPs of *L*. *sticticalis* could be divided into three groups: 17 Classic OBPs (LstiPBP1, PBP3, GOBP1, OBP1, OBP3, OBP4, OBP6, OBP9, OBP12, OBP14, OBP15, OBP16, OBP18, OBP19, OBP21, OBP26 and OBP29), 4 Minus-C OBPs (LstiOBP7, OBP13, OBP17 and OBP28) and 2 Plus-C OBPs (LstiOBP11 and OBP22) (Table 4).

**Fig 9:**
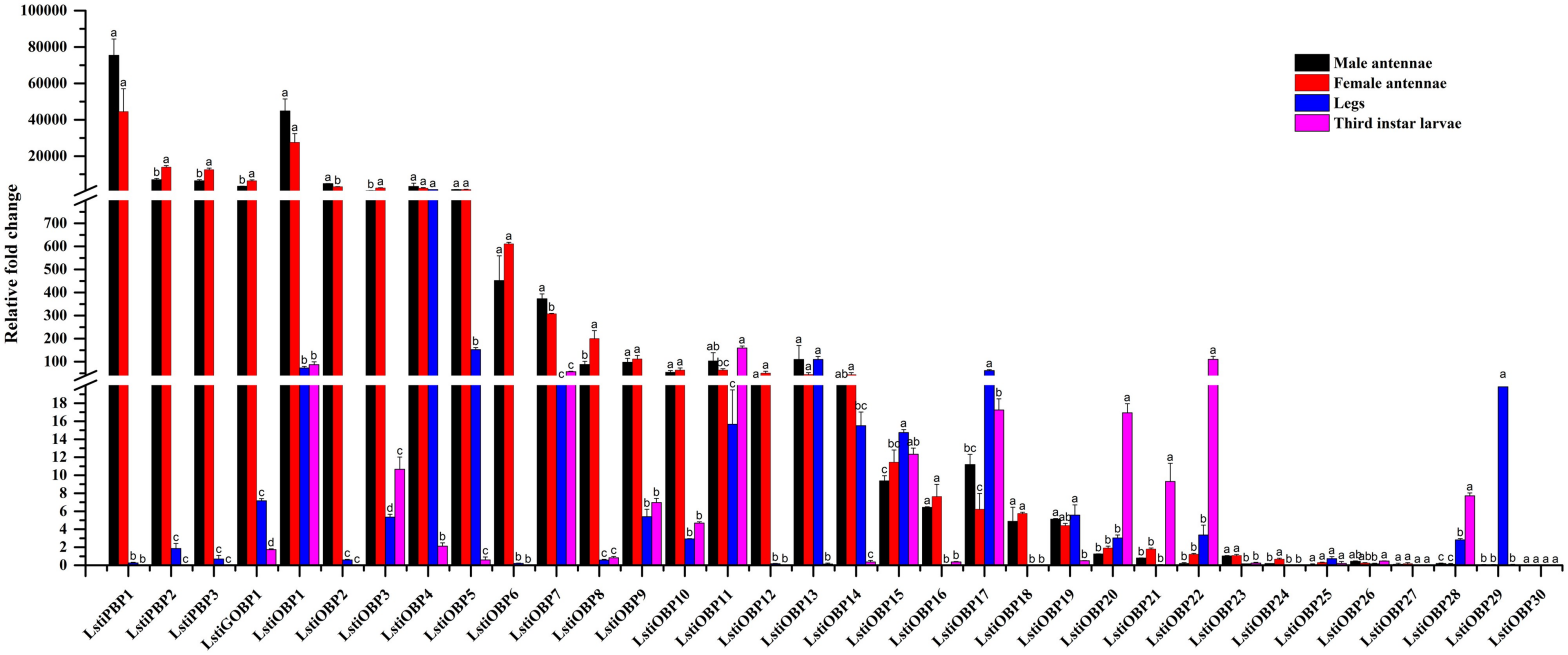
Expression pattern of *L*. *sticticalis* OBPs by RT-qPCR. The details were same as mentioned in Fig 3.

The RT-qPCR results showed that among the 34 candidate LstiOBPs, 22 LstiOBPs were highly expressed in the antennae, 4 LstiOBPs (*OBP15*, *OBP17*, *OBP25*, and *OBP29*) were highly enriched in the legs, and 5 LstiOBPs (*OBP11*, *OBP20*, *OBP21*, *OBP22*, and *OBP28*) were mainly expressed in the larvae. The expression levels of 3 LstiOBPs (*OBP13*, *OBP19*, and *OBP26*) were not significantly different between the antennae and legs (Fig 9).

### Identification and expression of candidate CSPs and SNMPs of *L*. *sticticalis*

**Fig 10:**
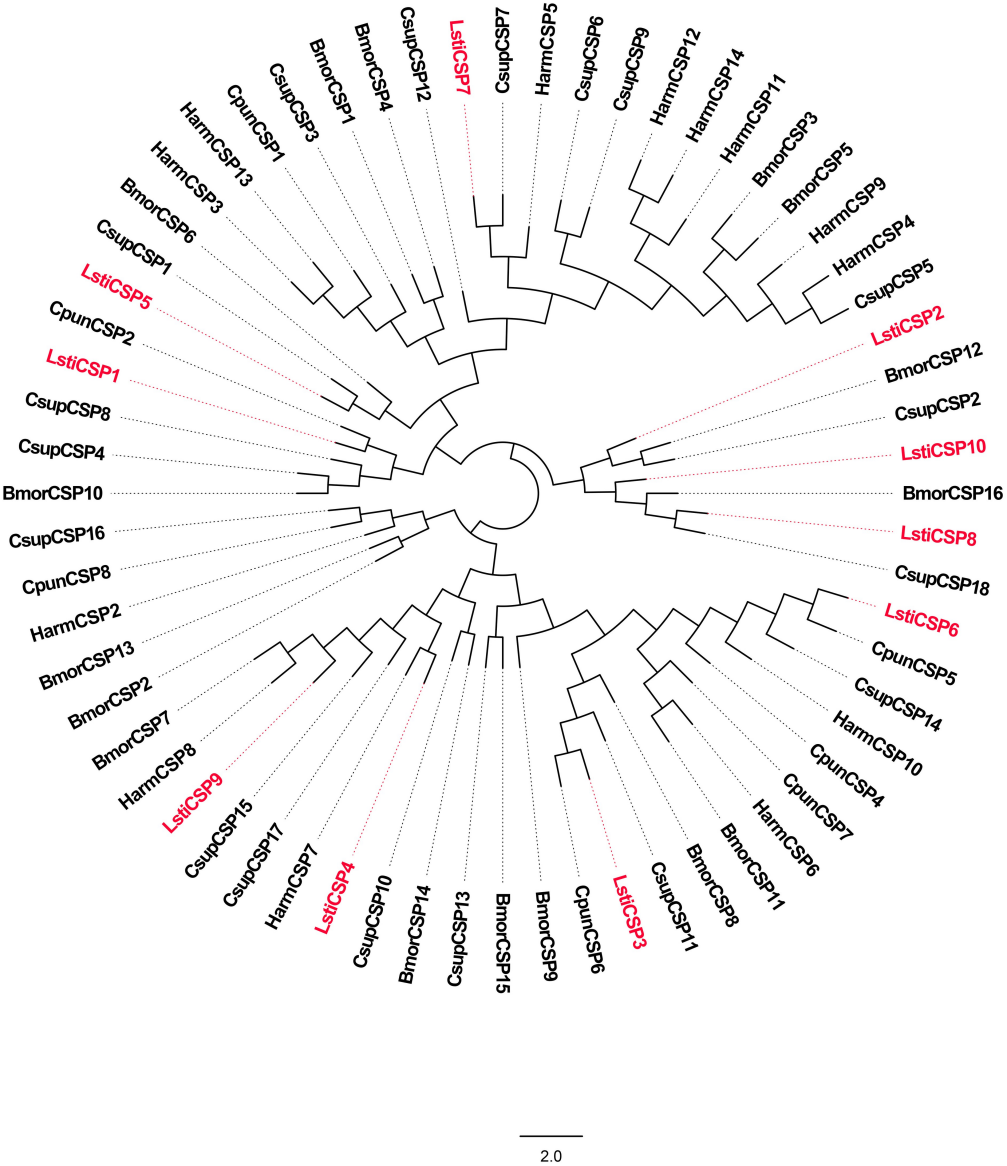
Phylogenetic tree of candidate LstiCSPs with known lepidopteran CSPs. Csup: *C*. *suppressalis*, Cpun: *C*. *punctiferalis*, Bmor: *B*. *mori*, Harm: *H*. *armigera*.

**Fig 11:**
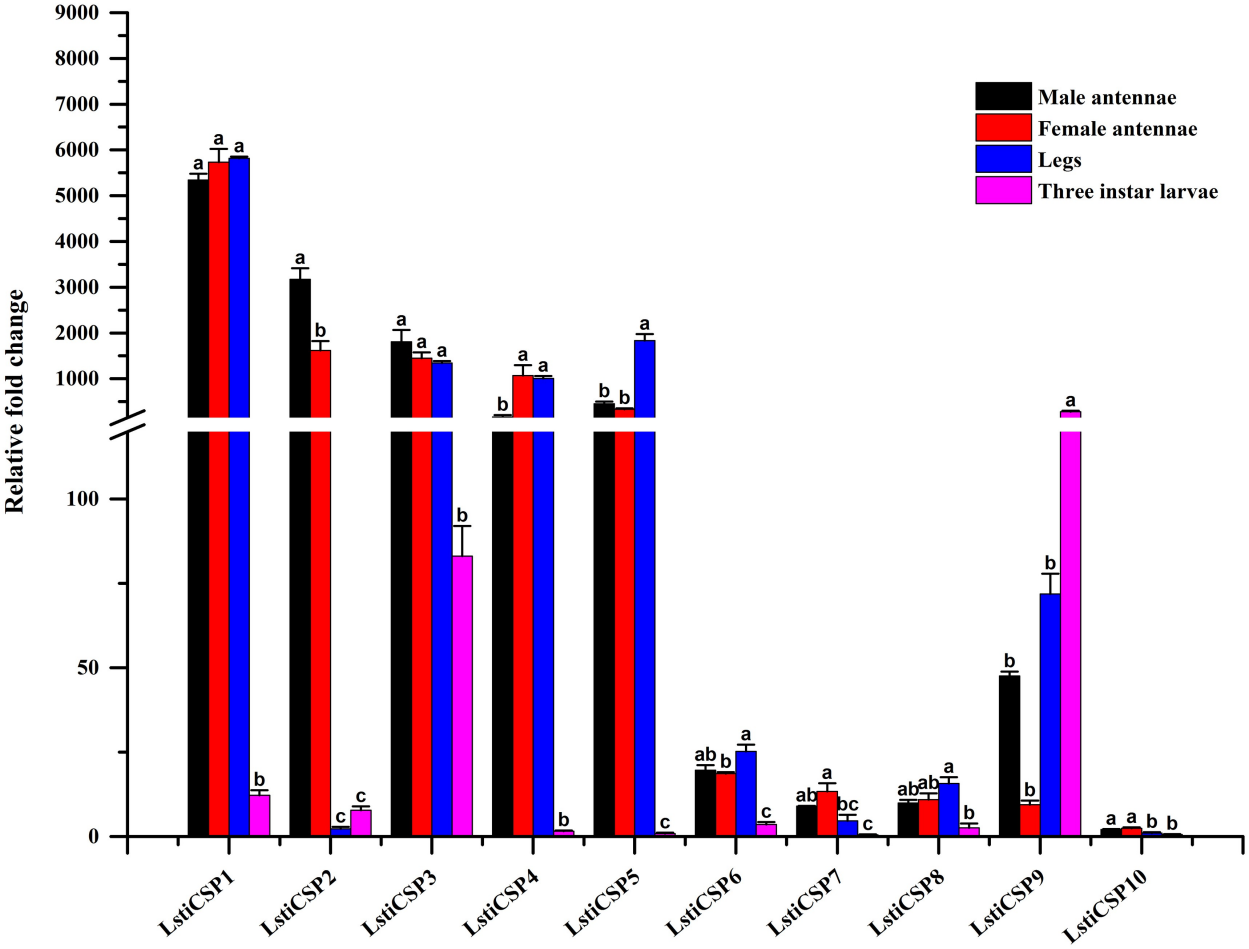
Expression pattern of *L*. *sticticalis* CSPs by RT-qPCR. The details were same as mentioned in Fig 3.

CSPs have a conserved cysteine pattern of C1-X6-C2-X18-C3-X2-C4 [11]. Through bioinformatics analysis, we identified 10 candidate CSPs in *L*. *sticticalis*. Eight sequences had full-length ORFs, but other unigenes were partial sequences. In addition, the unigenes C50444.g0 and C54133.g0 failed in the SignalP tests (Table 5). The 10 candidate CSPs of *L*. *sticticalis* best matched to Lepidopteran sequences, with an e-value < 1e-5 and an identity of more than 55% (Table 5). We named the 10 CSP candidates according to their expression levels in the *L*. *sticticalis* female antenna. The 10 CSP sequences in *L*. *sticticalis* were clustered with Lepidopteran orthologous genes from *L*. *sticticalis*, *C*. *suppressalis*, *C*. *punctiferalis*, *B*. *mori* and *H*. *armigera* in the phylogenetic tree (Fig 10). The RT-qPCR results showed that candidate *LstiCSP2*, *LstiCSP7* and *LstiCSP10* presented higher expression in the antennae, *LstiCSP5* had enriched expression in the legs, and the putative *LstiCSP9* was highly expressed in the larvae. In addition, the other 5 LstiCSP candidates (*CSP1*, *CSP3*, *CSP4*, *CSP6*, and *CSP8*) were mainly expressed in the antennae and legs (Fig 11).

**Table 5:** Unigenes of candidate CSPs.

Gene name	Length (nt)	ORF (aa)	Unigene reference	Status	Signal Peptide	Evalue	Ident	BLASTp best hit
LstiCSP1	2403	129	c52657_g0	Complete ORF	Y	2.00E-76	84%	gi|723592471|gb|AIX97825.1| chemosensory protein [*Cnaphalocrocis medinalis*]
LstiCSP2	1654	100	c50444_g0	5'lost	N	5.00E-32	72%	gi|614255941|gb|AHX37226.1| chemosensory protein 4 [*Conogethes punctiferalis*]
LstiCSP3	1750	124	c55235_g0	Complete ORF	Y	2.00E-68	80%	gi|472271926|gb|AGI37363.1| chemosensory protein 2 [*Cnaphalocrocis medinalis*]
LstiCSP4	2186	108	c56144_g0	Complete ORF	Y	7.00E-38	58%	gi|472271922|gb|AGI37361.1| chemosensory protein 1 [*Cnaphalocrocis medinalis*]
LstiCSP5	678	153	c50283_g0	3'lost	Y	2.00E-66	66%	gi|723592595|gb|AIX97836.1| chemosensory protein [*Cnaphalocrocis medinalis*]
LstiCSP6	1586	135	c54133_g0	Complete ORF	N	6.00E-79	94%	gi|614255951|gb|AHX37227.1| chemosensory protein 5 [*Conogethes punctiferalis*]
LstiCSP7	1105	126	c48206_g0	Complete ORF	Y	3.00E-43	55%	gi|328879844|gb|AEB54579.1| CSP5 [*Helicoverpa armigera*]
LstiCSP8	1208	106	c52695_g0	Complete ORF	Y	1.00E-55	81%	gi|158962519|dbj|BAF91720.1| chemosensory protein [*Papilio xuthus*]
LstiCSP9	556	120	c44870_g0	Complete ORF	Y	6.00E-49	66%	gi|723592481|gb|AIX97826.1| chemosensory protein [*Cnaphalocrocis medinalis*]
LstiCSP10	1281	105	c54763_g0	Complete ORF	Y	9.00E-50	73%	gi|723592536|gb|AIX97831.1| chemosensory protein [*Cnaphalocrocis medinalis*]

**Fig 12:**
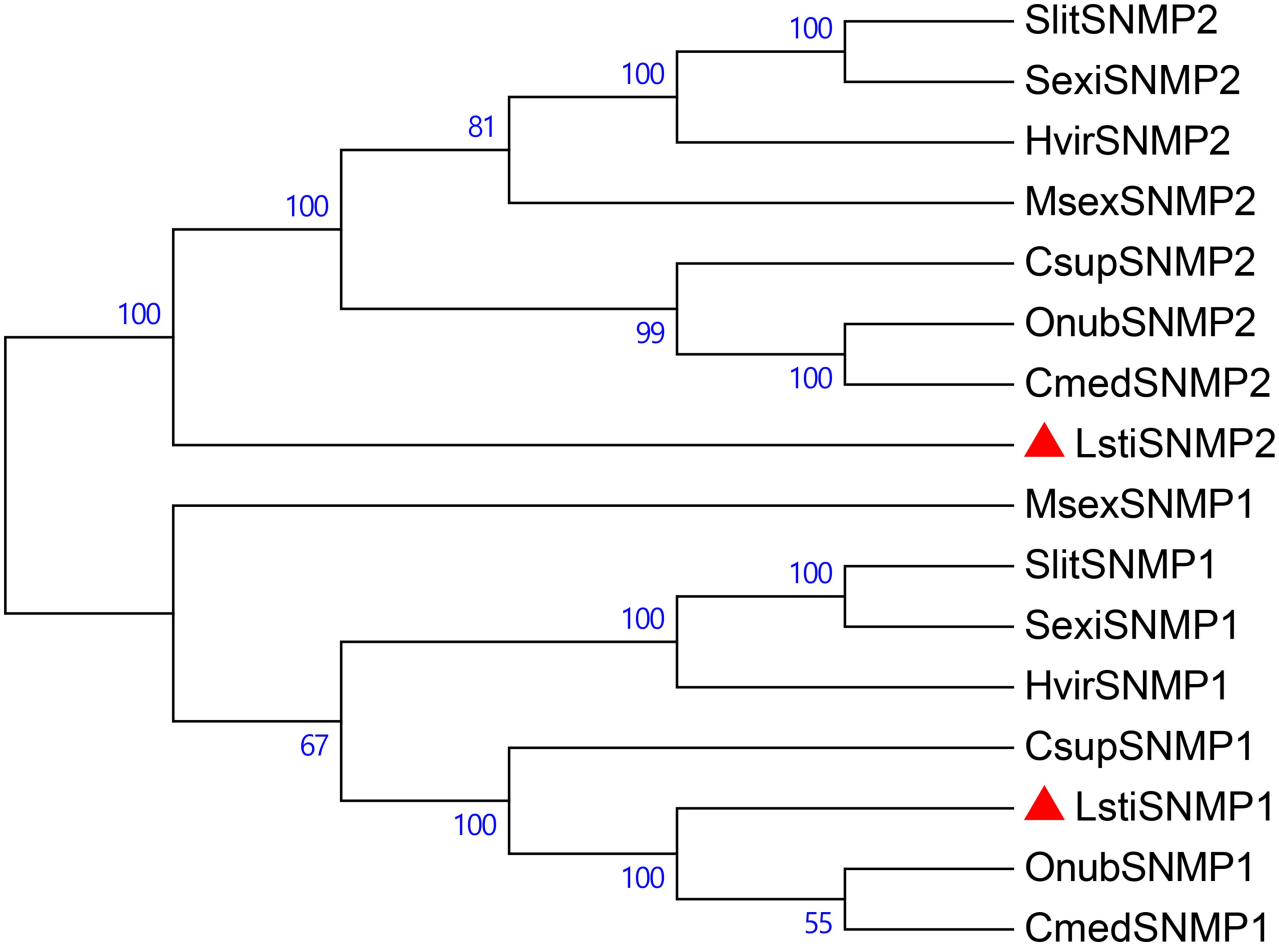
Phylogenetic tree of candidate LstiSNMPs with known lepidopteran SNMPs. Onub: *O*. *nubilalis*, Csup: *C*. *suppressalis*, Cmed: *C*. *medinalis*, Hvir: *Heliothis viresscens*, Sexi: *S*. *exigua*, Slit: *S*. *litura*, Msex: *Manduca sexta*.

In *L*. *sticticalis*, we obtained two SNMPs that were 3'lost and 5'lost sequences, respectively. The two SNMPs separately had a BLASTx best hits to *Ostrinia nubilalis* SNMP1 (similarity 88%) and SNMP2 (similarity 85%) sequences with an e-value < 1e-05 by NCBI BLASTp (Table 6). *LstiSNMP1* and *LstiSNMP2* had significantly higher expression in the antennae than in the legs and larvae validated by RT-qPCR analysis (P < 0.05) (Table 5). According to the phylogenetic analysis, LstiSNMP1 and LstiSNMP2 clustered with the known Lepidopteran SNMP groups (Fig 12).

**Table 6:** Unigenes of candidate SNMPs.

Gene name	Length (nt)	ORF (aa)	Unigene reference	Status	TMD (No.)	Evalue	ident	BLASTp best hit		FPKM	Counts	
									Female antennae	Male antennae	Legs	Larvae
LstiSNMP1	1431	453	c53448_g0	3'lost	1	0	88%	gi|312306076|gb|ADQ73892.1| sensory neuron membrane protein 1 [*Ostrinia nubilalis*]	465.42±45.27 a	415.63±117.75 a	0.39±0.26 b	0.03±0.02 b
LstiSNMP2	2070	300	c55425_g0	5'lost	1	0	85%	gi|312306070|gb|ADQ73889.1| sensory neuron membrane protein 2 [*Ostrinia nubilalis*]	814.19±28.70 a	1030.87±171.75 a	99.75±21.75 b	3.13±0.23 b

Note: data = mean±*SE*. The same letters have no differences, the different letters represent significant differences p < 0.05.

The protein sequences of the candidate chemosensory genes were listed in supporting information (S5 Table).

### Analysis and comparison of RNA-Seq data and RT-qPCR data

**Fig 13:**
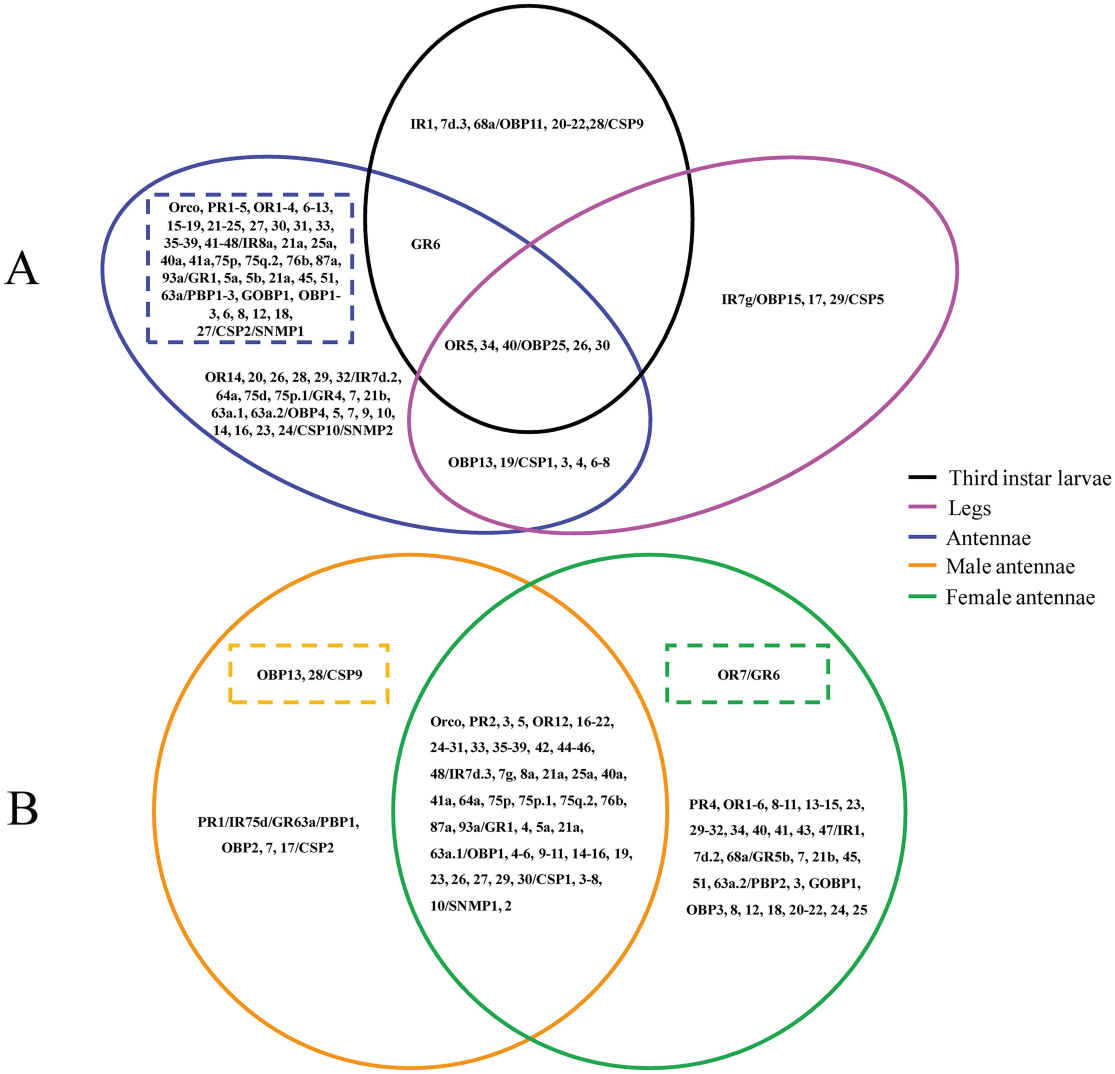
Comparative results of olfactory genes FPKM in the male antennae, female antennae, legs and third instar larvae of *L*. *sticticalis* (Venn diagram). A. comparison among the antennae, legs and larvae. B. comparison between the male and female antennae. Genes in the overlapping intersect show no significant difference among different tissues. Genes outside the intersect show significant difference. Those in the dash-outlined area show specific expression in the tissues.

We obtained 131 candidate chemosensory genes (54 ORs, 18 IRs, 13 GRs, 34 OBPs, 10 CSPs and 2 SNMPs) in *L*. *sticticalis* by Illumina sequencing. The results of RNA-Seq showed that most genes in the antennae had higher FPKM (Fragments per Kb per million reads) than in the legs and larvae (p < 0.05), especially 76 genes with specific expression in the antennae (Fig 13A). Furthermore, the *OR7* showed female antennae-specific expression (Fig 13A and 13B). All results analyzed were based on FPKM.

To test the result of Illumina sequencing, we investigated the expression patterns of 131 *L*. *sticticalis* chemosensory genes with RT-qPCR analyses. The RT-qPCR results showed that the expression levels of these candidate chemosensory genes in different tissues were mostly consistent with the results of RNA-Seq. Most notably, a majority of olfactory genes were predominantly expressed in the antennae. However, the expression levels of several chemosensory genes between the results of RT-qPCR and RNA-Seq have obvious differences. For example, the results of RT-qPCR showed *LstiOR28*, *29*/*IR64a*, *75P*.*1*/*OBP16*, *24* in the antennae, *LstiOBP29* in the legs and *LstiIR1* in the larvae had specific expression (Figs 3, 5 and 9), but these genes in Illumina sequencing analyses only showed higher expression (Fig 13A); on the contrary, the (*IR8a*, *76b*/*PBP1-3*, *GOBP1*, *OBP1-3*, *8*, *16*/CSP2) only showed higher expression levels in the antennae by RT-qPCR (Figs 5, 9 and 11). These differences in the results need further research for confirmation.

## Discussion

At present, the molecular basis of chemoreception in Lepidoptera is well understood compared to other insects, but the research on Pyralidae is relatively scarce. Therefore, we sequenced and analyzed the transcriptome of adult antennae, adult legs and larvae from *L*. *sticticalis* and obtained a dataset of 54 ORs, 18 IRs, 13 GRs, 34 OBPs, 10 CSPs and 2 SNMPs. In this study, comparing to the antennal transcriptome in Lepidoptera from *C*. *suppressalis* (47 ORs, 20 IRs, 26 OBPs, 21 CSPs and 2 NMPs) [21], *C*. *punctiferalis* (62 ORs, 11 IRs, 10 GRs, 15 OBPs, 8 CSP and 2 SNMPs) [43, 44], *O*. *furnacalis* (56 ORs, 21 IRs, 5 GRs, 24 OBP, 19 CSP and 2 SNMPs) [45, 46], *C*. *medinalis* (29 ORs, 15 IRs, 30 OBPs, 26 CSPs and 2 SNMPs) [9], *H*. *armigera* (60 ORs, 19 IRs, 9 GRs, 34 OBPs, 18 CSPs and 2 SNMPs) [33, 47, 48], *B*. *mori* (62 ORs, 17 IRs, 69 GRs, 44 OBPs, 18 CSP and 2 SNMPs) [31, 49–51] and *H*. *assulta* (64 ORs, 19 IRs, 18 GRs, 29 OBPs, 17 CSP and 2 SNMPs) [33, 52], our LstiOR dataset of sequences has no notable difference in the identified gene numbers.

RNA-Seq and RT-qPCR results both showed 54 putative LstiORs were mainly expressed in the antennae, which was similar to the other Lepidopteran results [9, 21, 31, 33, 43, 45]. Studies about *B*. *mori* showed that three female-biased ORs (*OR19*, *OR45* and *OR47*) are capable to respond to host plant volatiles (linalool, benzoic acid, 2-phenylethanol and benzaldehyde) [49, 53]. The 6 female-biased expression LstiORs (*OR4*, *OR23*, *OR29*, *OR30*, *OR32* and *OR34*) that were clustered with the female-biased ORs from *B*. *mori* in the Phylogenetic tree might have similar functions, but further studies were needed. In view of the host selectivity of larvae [3, 4], *LstiOR5*, *OR34* and *OR40* that were richly expressed in larvae might play important roles in host-plant selection. Some reports showed that PRs specific expressed in male antennae detected the sex pheromone components of female moths [54, 55, 57, 58]. However, in our study, 5 candidate PRs of *L*. *sticticalis* were expressed in the antennae of both sexes, which is consistent with the recent reported results of 6 putative PRs identified in *C*. *suppressalis*, 2 PRs (OR6 and OR13) in *H*. *armigera* and 2 PRs in *S*. *littoralis* [21, 56, 57]. Therefore, the recognition mechanism of LstiPRs to the sex pheromone [59] of the female moth requires further research.

As the complement of ORs, ionotropic receptors were first discovered in *D*. *melanogaster* [28] through genomic analyses. Compared to ORs, the IR family is relatively conserved both in sequence and expression pattern. In our study, among the 18 LstiIRs we discovered, 13 sequences have orthologs found in Dmel/Bmor/Slit IRs; the expression levels were not significantly different between male and female antennae, which were similar to the IR expression in *S*. *littoralis* [54], *C*. *suppressalis* [21] and *H*. *armigera* [33]. *Lsti76b*, as well as *LstiIR8a* and *LstiIR25a*, was highly expressed in the antennae, and these genes might also be special subunits of individual odor-specific receptors [60]. The functions of IRs in *L*. *sticticalis* are likely to be conserved as IRs in other Lepidoptera, both in terms of the relatively high sequence conservation and the comparability of expression levels.

Gustatory receptors play a critical role in the detection of chemicals, which ultimately influence the insects’ decisions when looking for food, mates and egg deposition sites [32, 62]. Interestingly, our LstiGR4 shared 72% homology with HarmGR4 which were identified as a sugar receptor [47, 61], so LstiGR4 might be a sugar receptor and participate in sugar detection and consumption. *GR21a*/*GR63a* that were expressed in CO2-sensing neurons could allow the detection of CO2 concentration in *D*. *melanogaster* [62–64]. In our study, 5 LstiGRs (GR21a, GR21b, GR63a, GR63a.1, and GR63a.2) were clustered into the clades of DmelGR21a/BmorGR63a in the phylogenetic tree and might be CO2 receptors. However, annotation of these GRs awaits further demonstration.

Of our 34 LstiOBPs, most LstiOBPs were richly expressed in the antennae of both sexes that was similar to other transcriptome analyses in Lepidoptera [9, 21, 33, 43, 44]. As specific OBPs, PBPs usually were considered to have a connection with male moth perception of the sex pheromone components released by female moths [66–69]. Our 3 LstiPBPs were closely clustered into the PBP clade of other Lepidoptera in the phylogenetic tree, which suggests that our LstiPBPs might have similar function. Currently, studies also show that OBPs specifically expressed in larvae displayed a high recognition capacity to the major sex pheromone component [65]. Thus, one of the LstiOBPs (*OBP11*, *OBP20*, *OBP21*, *OBP22*, and *OBP28*) which specifically expressed in the larvae might play a key role in the perception of female sex pheromone in *L*. *sticticalis*.

CSPs are more highly conserved than OBPs across insect species and are widely expressed in different parts of the insect body [31, 70]. Our 10 LstiCSPs were primarily expressed in the legs and antennae of the adults, which was similar to the results of other Lepidoptera [9, 21, 31, 33, 43, 45]. But *LstiCSP9* was mainly expressed in larvae. The antennal enriched CSPs might be involved in chemoreception [71], and the CSPs expressed in the legs might participate in other physiological processes beyond chemoreception [72]. However, the function of our putative LstiCSPs requires further research.

Because SNMPs were first identified in Lepidopteran pheromone-sensitive neurons [17, 73], these proteins are believed to be involved in the recognition of insect pheromones. In this study, the expression levels of SNMPs in *L*. *sticticalis* were consistent with the reported results that *SNMP1* of *H*. *assulta* was primarily expressed in the antennae, and *SNMP2* of *H*. *assulta* was abundantly expressed in the antennae and legs [33]. Previous studies showed that SNMP1 was crucial for the detection of the volatile pheromone 11-cis-vaccenyl acetate in *D*. *melanogaster* [18]. SNMP2, in contact with pheromone-sensitive sensilla, was expressed in sensilla support cells [74]. According to the similar expression levels and physiological analysis to other Lepidoptera, we can infer that SNMPs in *L*. *sticticalis* might have the same role as in *D*. *melanogaster*. However, the general mechanism of SNMPs’ function in insects remains inadequately understood. Therefore, future studies on the function of SNMP1 and SNMP2 in *L*. *sticticalis* are necessary.

## Conclusion

Our aim of this study was to identify genes potentially involved in olfactory signal detection in *L*. *sticticalis*, and this aim was well met by the identification of a repertoire of 54 ORs, 18 IRs, 13 GRs, 34 OBPs, 10 CSPs and 2 SNMPs. Our results not only establish a means to further elucidate the molecular mechanisms of chemosensation, but also provide potential targets for disrupting the chemical communication system in *L*. *sticticalis* as a means of pest control.

## Supporting information

S1 Fig Unigene length distribution of *L*. *sticticalis*. (TIF)

S2 Fig Distribution of Nr homologous species annotation on *L*. *sticticalis* unigenes. (TIF)

S1 Table Nucleotide sequences of all identified candidate olfactory genes. (DOCX)

S2 Table The sequences used for phylogenetic trees of chemosensory genes in *L*. *sticticalis*. (DOC)

S3 Table Primer used in RT-qPCRs. (DOC)

S4 Table The accession numbers of 131 candidate chemosensory genes in *L*. *sticticalis*. (DOCX)

S5 Table The protein sequences of the chemosensory genes (ORs, IRs, OBPs, CSPs, SNMPs) in *L*. *sticticalis*. (DOC)
