# Supplementary figures and images for: Identification of candidate chemosensory genes by transcriptome analysis in Loxostege sticticalis Linnaeus
Source: PLoS One. 2017 Apr 19;12(4):e0174036. doi: 10.1371/journal.pone.0174036 (PMC5396883; doi:10.1371/journal.pone.0174036)

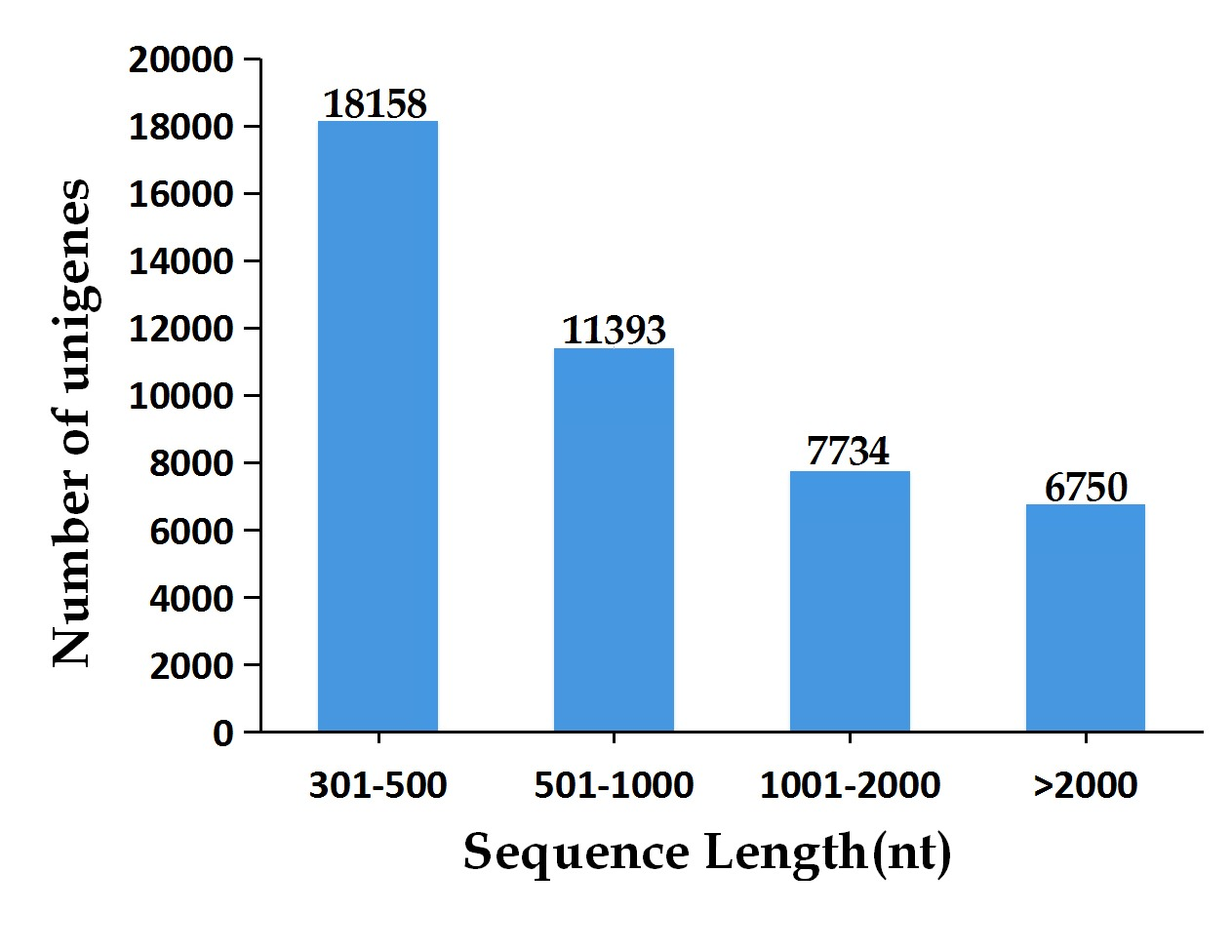

Supplement: S1 Fig — (TIF) [file pone.0174036.s001.tif]

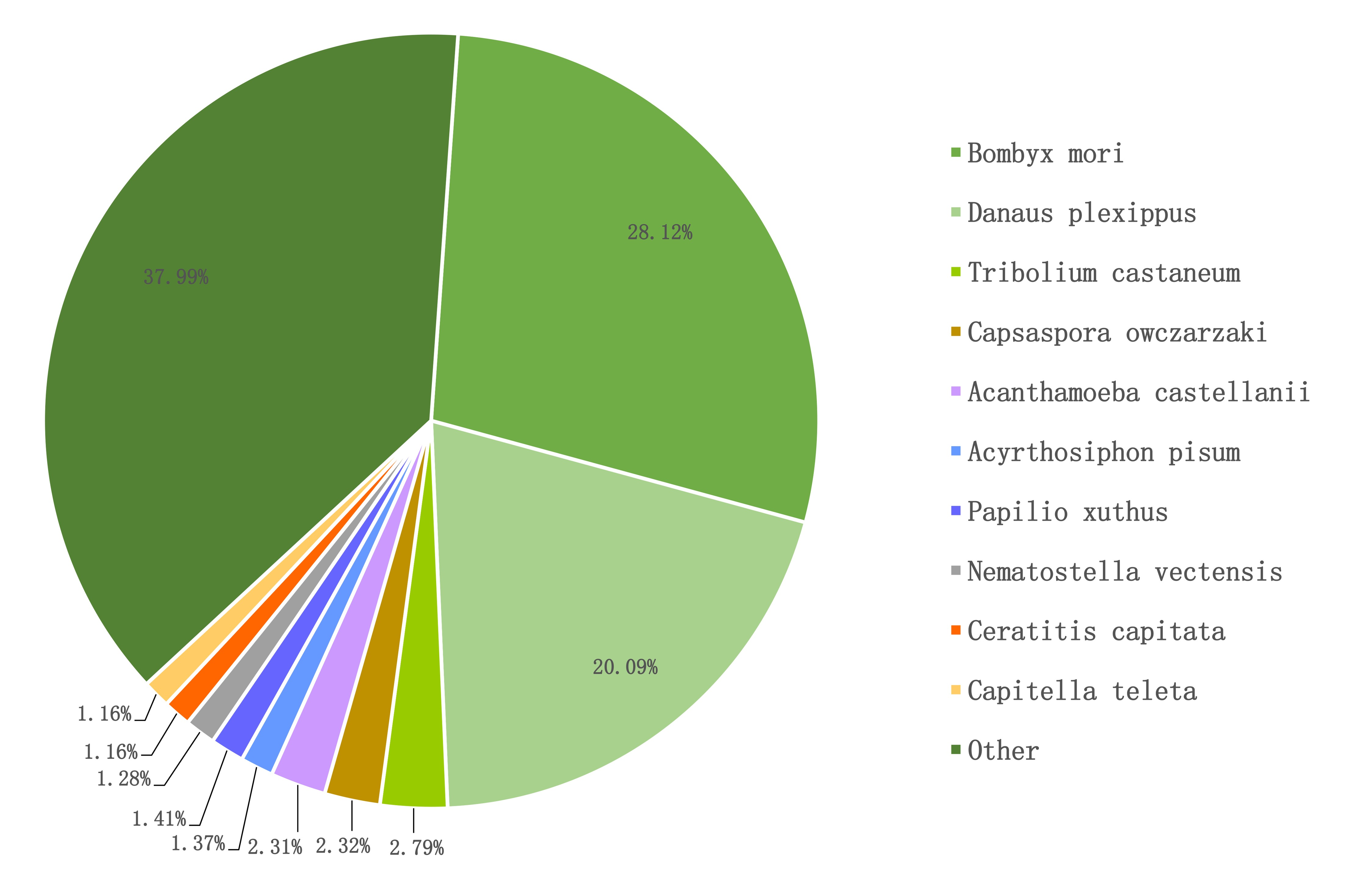

Supplement: S2 Fig — (TIF) [file pone.0174036.s002.tif]
