# Supplementary material for: Identification of candidate chemosensory genes by transcriptome analysis in Loxostege sticticalis Linnaeus
Source: PLoS One. 2017 Apr 19;12(4):e0174036. doi: 10.1371/journal.pone.0174036 (PMC5396883; doi:10.1371/journal.pone.0174036)
Supplement: S1 Table — (DOCX) [file pone.0174036.s003.docx]

**Nucleotide sequences of all identified candidate olfactory genes in the *Loxostege sticticalis***

>PBP1 [Moltype=mRNA] [Organism=Loxostege sticticalis], complete cds

GGAGTGGTGACAGATGACATATCCAAAGATGTGGACGTCGAAGATACTGGTGATGATGGTGGCAGCGTGCGTGATGACTGTGATGGTGGACTCCTCGCAATCGGTGATGACGTCGATGACAAAGAATTTTATAAAGGCTTATGAAGCATGCGCGAAAGAGTACAATCTCCCAGAGTCCACAGGACAGGAACTGATAAACTTCTGGAAGGAAGGTTACACAGTGACGAGCCGTGAAGCCGGCTGCGCCATCCTCTGCCTGTCCTCCAAGCTGGATCTGCTAGATCCTGAGGGGAAACTCCACCATGGAAACACTGTGGAGTTTGCCAAGCAGCATGGATCTGACGATGCAATGGCTCACAAGGTTGTGGAAATTCTCCACTCGTGCGAGAAGGCGGCTGCTCCTAATGAAGACATGTGTCTGGTGGCTCTAGATGTTTCCATGTGCTTCAAGAAGGAAGTCCACAGTCTGAACTGGGCACCTGACAACGAACTGCTGTTTGAGGAGTTGGTGGGCGAAATGAGCAAAACATGAGGACCTCTTAAGAGTCTTAAGGTGGCCATCTAAATGTATGAGATTTAATTATTCAGCCAGAGGAGTAACCATGAGTTCTTGTAACAATAAGTTGTGGGAGTGAGACGCCGTTATGTGCGTGTATCGGGCTATCTCGCTTCCACAGTTGCAAACGATCTTCTATAAGAGCTGATGATTAGGGGTCTGACAATACATTCCGTTTCCCACATTTTTGGTTTCCACCTGGCTTGTATCATAGCTGGCTCAATGATTAAATGGCATCCACTTGGTTAATCCACATGCCGCCAGTACCTTGAATATGCGACGATAACTTCTTGCCATTTTACTTTCATAAGATTTAGTTTGCCTTTTCAATGACGACTTTTGCCAAGAAATAAGGATCGTGATAAAATCTAAGTATAATTGCAATGTTTATTCTTGTACAGTAATGTGTTTTTCGAATTTGGCTAGTCTTATGTTTCTGGAACGTTTTGTCTCTTATCCTTTTAAGGCCACGTGCTCCCGACTTCTTTATTCAGTGTTTGTTTAGTTCAGGACTAAACCATCATCAGTACTTTCCTGA

>PBP2 [Moltype=mRNA] [Organism=Loxostege sticticalis], complete cds

ATGGGACAGCAATCTGACCGAGCGGAGAGATCAGGCGTAGGACCTACGGCTTTTCATGCTTTCCAAGGCACGGGGGTGAAACACCAACAATTTACAAACTCCGGGCCGCCCTGAGCATTTCTGAACAGAAAACCCCAGGAACAAATTTTTGTCCCGACCCGGGTAAGAAACTACTGTAAACCCTAATTAGAAATATTGCGCCTGTGCCCATTAAAAGGGTTAACGTCCATTTAACTTTCAACTACGATCAGCTAAACTAGACCCATAGTATTTCTTCTTACTATCACTCTTCAAAAACATGGTTTAAAGCTTAGACAGGTTCTAGAGCGACGGGTCCAATTCATCACCAAAATGAGTACTGGTTCATGAAAGATCATGGTGCTGTTTAGTTCAAAGCCGTAACATTGCATCGATGTGAACTGAAAGTCTTCAATAAAATTCCGTCACTTTTGTTATTTCCATTTATGAGCTGGTAGATCAGTTATTTGTTTCAATATCAGCCCTAAAATGAACTCTCCTTTAATAGTTTCAATATCTTTTTATTTAATTAACTTTTCAACCTCTTTTCCAGACGAAGCCACTGCTAAGCAGATAATGACCATCGTCCACGAATGCGAGGAGAAGTTCGCGACCAACGAGGACCACTGCGCGCGCGCCATGGAGGTGTCCCGCTGCTTCCGCGACCACATGCACCGGTTGCAGTGGGCGCCGTCGGTGGACGTGCTGGTGGGAGAGATCCTGGTTGAGATGGCTTAGGAGTGCACCACGGTCGGCTTTTGGACTAGTAAACAAGTGAAAGCGTAGGGGGTAACCGCCATCTTTTAAAACAATGTTAAGACAAGATGCAAGTGCTGAAATCGTGCTTTATAACATGGAGGTTTTTCAAATTTACACGCTTTTTAAAATCAATTGAATAATAACGGCCATGAATATGCATAAAATCTAAGGAAGTTTGCATATCAAATTTTAGCTACCAATCAATTATCTAGCTATGAAAGTAGAAATAAGTGAGACTGTGAGATAAAATCGAAAGAGTCAATACTCCACTCCTTTTATCGTTGGGCTTTATTGGGCTAGAAATCTTGTATAAAAACAATTGTTTATTCATAGTTTACTAGTCCATAAGTTAACTTAAACTCCGCAGAAAAATCTACGAATCGAATGTTGAAAAGGATACGATTTATGTGTTTTTTGTTGTAATACGCTTGTGAACACGGCATTTTGTTAGAAATTAGGTATGTTTTTGAATAAATCTGTGGTGGC

>PBP3 [Moltype=mRNA] [Organism=Loxostege sticticalis], complete cds

CGGAGGAGATGGGTTTCTCGGTGAGGCTGCTTGTGGTGTTGGTGGCGGTCACCATTTATGGTGTGAACTCTTCTCAGGATATAATAAAACAAATGACCATCAATTTCGGGAAGGCTTTGGACTCATGCAGGAAAGAGCTAGACCTTCCTGACTCCATAAACGCAGACTTTTACAACTTCTGGAAGGAAGGCTATGAGCTGTCTAACCGGCAGACGGGCTGCGCGATCATGTGCCTCTCCTCCAAGCTGGACCTGGTCGATCCTGAGGGAAAGCTGCACCACGGGAACACTCATGAGTTCGCCAAGAAACATGGCGCTGATGACGCAATGGCGAAGCAGCTCGTAGACCTGATCCACAAATGCGAGAGCGATGTGCCCGACGACCCCGACCCCTGCTTGAAAGTGCTCAACATCGCCAAATGTTTCAAAGCCGAGATCCACAAGCTCAACTGGGCCCCGAGTATGGACCTCATAGTGGCTGAAGTCTTGGCTGAAGTTTAAATAGCCTGGCTTGGATATTTTTAACTATCAACGACACGGCGACCTTCTCTTGTTTTTGTATAACACTTGTCAACATACTCGCACTTAGGTAGAGATTGTTTTAGTTAGAGAAAAGCAGACTTTATTTTGTGATTTTACTTGTAGAATCTTTATTTCGAGCAGTTTTATACATATAGTTAGACATGACTTCTTCTGTATGCTCTATTGATAATATTTTACTTGCAATAGTATTTGAATATATCCTTGAATTATTTTCTAATTTTGATATCAATTACACGCCTATTTTGTATTATTGTACTGAAGTTGATTTTTCATTGTAATAAAATTTTTAAATAATTTTTCAGTAGTTCAAGCTTAAATCATAATCTACAAAAAATCGACGAAGCGATTATAATGGAGTAGGAATCATTCTTAGTAACCAAATATAGTTGTTAAAGGTAGATATTTTTTTATAAATCAGTGTAAGAATATTAGTTAGTTAGTAATTTTTATAGTCTGCCCAATTCGTTCGTGTTGGAATTTAAAATTTGTAGTTTTGGTGTATTTTGGTCCTACTAGATATAAGACAATATTTAATCAGGGTTTTTTTTAATAGTTAAGAGAAATAAAACATGGG

>GOBP1 [Moltype=mRNA] [Organism=Loxostege sticticalis], complete cds

GGCGGTGGTGGTGGCGCTGGCGCAGGTGGAGGTGAAGGCAGACCTTGGGGTCATGACGGACGTCACGCTCGGCTTCGGACAGGCGTTGGAACAGTGCAGAGAAGAGTCGGGTCTGTCGGAAGAGAAGATGGAGGAGTTCTTCCACTTCTGGAGCGAGGACTTCAAGCTGGAGGCGCGCGAGCTGGGCTGCGCGCTGCGCTGCATGAGCAACCACTTCAACCTCATCACCGACTCCAACCGCATGCACCACGCCAACGCAGAGAAGTTCGTCAAGAGCTTCCCCAACGGTGAAGTCCTGGCGAAGCAGCTGGTGGGCATGCTGCACGAGTGCGAGAAGAAGCACCACGACGAGGAGGACAACTGCTTCCGCGTGCTGCACATGGCGGCGTGCTTCCGCGAGGCGTGCCGCGGCGCCAGCCTCGCGCCCACCATGGAGATGCTGCTGGCCGAGTTCATCATGCAGGGTGAGAACTAGCCCTGCCATCTGATAAAAAATCGGACTTAGGGCAAAAACTGGATATTTGGGTGAAATAAAGATTTTAAACCGGACTCCCTCGAAAATTACAGTTCAATAAATAAATAGTTATCATTTTATAGCGCACGGAGCAGGGCGACTAGGGAGGTCACGTCACAACACGAGTGCATGGGAATATTTGTCACACCGTATTTTGAACTATGAAATAATTTATGCTATTTCATTTAATATTTCCCTTCTTTCAAGTTAATCGGATTTTATGCCAGGGAAAATACAAGTTTCCCGGGCACCCTCCAAACTAGGATAAATCCGGGGAAACCCGGCAGAACTAGCTGTTACCAACCACCGTCCTACCTTCTCAGCGACCTGATCAGTGCGCGAACCCAAACAGTCTATTAGGCTGAGTAAGACACAGGCCAAGCATTATCCACCGTGCTTCTACTTTTTGTCTCGCCTCTCAGTTCTCCGGCCTGTAGAATTCATAAAATATGTCTAATAGACAGGCCACGCAGCAGCCACCGTCACATCTGACTTCCCTACGACTTCAGATGGTCCAAACCGGAAAGTTAATCGTGCAGGCCGCTTATTACACGACCTACCTTTAACTTCTGTCATAGCTCTCAGACCTTTAACCCGTAGAATCATGCAGACCTCAAATACCGTCTGTCACTGTCTGTCTCACCTCTCAGTCCTCTAACTCTTCGAGTTCACCACGTAGGCCGCGTAGTAGCGTAGTAGCGTTGATCAGTCCTCCAACCTCCACATACTTTTTAACGTCCAGTAATACCCCAGTGACTAACTCGACTATGGCAGCCCCAATGTTAGTTACATACAACCTTACCTACCTTACATGTATGGAGTAGATATTCATGTAGTAACTCTTTTCATCTAGTTTTAGACCGATAGCTGATCTTTATTGCGAAATACAACTGTATTAAAAACACACATATGTTTAACACCCGAAATATAGACAAGTATAACTTGAGAATCTAGAAATCAAACTTTTGACTTATCTACTATTATTACATACTTACTTTTTTTAATGTAAAGTAAAAATCTTTTCTTAAGTCATTGTTTAAGTTAAAGTAATAAATTTTGCTTGACTATGAATACGGGTGTTAGTCTATCTGCTCAAATGTTTACTAGTACAGGAGAAACGTAGCACGTTTTCCATAGTATGATGTGTAGCATTAATAGTAATAGAATGTAGAGGTATAATAACTACTAACTGGGGAAATGTCGACTTATATTTTTATTATTAATTTGTGTGATAATTTAATATTTATTTGTATGTTATTTAGTTTAATTACGTGAGAAACTAGCAGATATACAAACGACGACCTGGCTACGCGATTTGAATGTTGATATTAATTGTGAAATAGTAAAGGGATGTGGTACAATTATTTGGGATGAGTGTATTATAATAAACTATTCCATCACTGATGTTTCTAGCAGAGGCAAACTGTTTTATTTGGTATGTGTCTTTGTTGCGATTTTACTGATATTTTCACCTGTAGGTATAAGCGGGGATGTAGCTCAGTGGTAGAGCGTTCGCTTTGCATGTGAAAGGCCCCGGGTTCGATCCCCGGCATCTCCAATTTTTGTAGCAGTCTCATTTTTCTTAAAATCTTTTTTTACTTTAATACAAACAACAAGTCAAAGCGTAATTATTTATTTATTTTATTACCGTATTATTAAAATTAAACCAAGCG

>OBP1 [Moltype=mRNA] [Organism=Loxostege sticticalis], complete cds

GTCGGTGTGCACAATTCAATCATGATGGCAGCTATGACAGGGCGGGCGGTGCTGGTGGCAGCCGCGCTGGCGGCGTTGGCGCTGGGAGCGCGCGCCATGGACGACGAGATGGCCGAGCTGGCAAAGATGCTACACGACAACTGCGGCGAGGAAACCGGCGTGGACCTGGGACTGGTGGACAAGGTGAACGCGGGCGCGGACCTGATGCCGGACGGCAAGCTCAAGTGCTACATCAAGTGCATCATGGAGACGGCGGGCATGATGTCGGACGGCGAGATGGACGTGGAGGCGGTGCTGGCGCTGCTGCCCGACGACATGCGCCGCAAGAACGAGCCCTCGCTGCGCGCGTGCGGTACGCAGAAGGGCGCCGACGACTGCGACACCGCCTTCCTCACGCAGGTCTGCTGGCAGAAGGCCAACAAAGCTGACTACTTCCTCATTTAGTCACACTGTTAAAATTCCATATTATAATCAATGTTTAACTAAACTTAATACCTAATAAAATATACCAACTAAAACATTTTAATTTTTTGCTGATAGTGATTAGTGACTGAGTAATATTTATTTAGTAGGAGAACAGTTCAATTGTGTAGCAGAAAATCTAAATTATTTTGAGTTCTTCATAAAGTTAATGACAGTAAGTAGTAAATTCCTTGTGGTCATGTACCCATAGGAAATTGACAAGATTGATTTTATTTATAGAACAAAGGTATTTATTTTAACTGAAAAGATAATCACCAATGTTCTAGTATCTTTTTTTTCATCCTGGTTCATTGAATGTAATCTACAGATAAGCATGAGCATTGAAAGAAAATAAAGGAAATGTGGCCAGCAGTTTTTTTAAATCTTGCCATTACTGTTCACTGCTGGGCAAAAACCTCCCCCAAATCCCTCCAACAATCAGTTTGTGATGATTGAGGTCAATACGCCGCGCATGCGCCTACGACTCGTGCCATATGAACCCATATGTTTTTTTAACTAAAGTGTTGAAATAATTTATAACTGTAAAAAATCCCACAAGTGGCACATGGCCCACCATAACTTTACCCTACCGCCGCTTCAGGACTATTGTAGGCTCGTGCTAAGAAGATACAGCGCAATAAACTCAGACACCATTGCCGCCTCTTTTTTAAGGTTAACAATAAGTTTAATTCTGTATTGGTGAAAGTAATACCTACTTTTCTTATCTTTTTCAAATAACCTTTTCCCATTAAAGTATTCTTAAGGTAAGTCAAGAACCTTATCTATGGTTTAAGAAAAAAATTAAGACATTTTCTGTTTTCTCACACATTGCCTGGTGGCTTTACATCATTTAGTTTGATGAGGCAACTTTTACTTATAGATTAACTTTAGACTGAAAAAAAATTTGGTAAAAGGAGAGCCTGAAATTTTTCAAATCTTCCCAGAACACATCTTTTGTATAAAAGTTTGATGAGCAAGTGGTGTATTAAAGAAGAATCCAAAGAATTGGCATGACATCAAGAATTCACAACCTTAATCTGAGTGGAGCGCCTTTTGGTTTTTAGATACTTATTATATGTGTGTAATTTAATATTAAATATACCAAATAAAGCTTGTTCTTATTTTTATTAAAAAAAATATTTTTAAACATTTTCATGTTGATTTTCAAAATGGGAATCTTTGGTTAAAAAAAATTTTAACTTCATCAACTTTGATTGCTAATCAATAATGATTCATAAAGAATGCCACAAAGTGTACATTAACGTTAAAATGCACCACACCAAACAAATCAAGCAGGAAAATATCAGTACAATGTAGGGGGTCTTATTGCTAAAAGCAATCTCTTTTAAACAATCTTCAGGAACCACCATTAACTACCCATTTTCAAATGGGTAGATTAGATAGGTAAAGAAAGCAAACAATATCAATTTGACGAAAAAAACAGTCTAGAAATAAACTTTTTATTAAAAATCCTAAATCACAGTCTGAACGCAACTCAGAATAAAAAATAACAAAAAAACTATTTACCAACCCTATTTTTACACTTAATAAAGCTTAACATTTTCACAATTTACTTTTTTTCTTTTCTTTTAGCTTTTGCCGCAGCACTCGACGTAAAATCTTTCCGGATGGATTTTTGGGGATAGCGTCGATAAATATCACTCCTCCGTGAAGACGCTTTGCTGGTGACAATCTTGTCGCTGCGAAGTCTATGATCTCCTTCTCAGTCGCCGTGACGCCCGGCTTAAGCACCACGAAGGCGGTGGGCAGCTCGCCCGCGGCCTCGTCGTGCGCGCCCACCACGGCGCTCTCGGCCACCGCCGGGTGCTCCAGCAGCACGCTCTCTACCTCCGCGGGTGGCACCTGATATCCTTTATATTTGATGAGTTCCTTCAAGCGGTCTATGACGAAGAAGCTCCCGTCTTCGTCGTAGTACCCGATGTCGCCAGTCTTGAGGTAGCCCTCGCTGTCTATCATCTCCCTGGTGGCGGCCTCGTTGCCCGCGTACCCCTTCATCATCAGGGGACCTTTCAGGCAAATCTCACCACTCTGTCTAGGGCCTTGTTTCTTACCAGTGTCTAGATCCACAACCTTTGCTTTTAATCCAGGTACAGGGTAACCACCACTTCCCGGTTTACTTTTCTCCATATCTAGATCTGTTGTTGCTAGTAACGATGTCTCCGTCATGCCGTAACCTTGGAATATTCCAAGACAGGTAGGCAAACGTTTCATGGCGTCGTTGATGGTCTCCACACTTAACGGAGCTGCGCCGCACAGCACCACCAGCACCGACGACAGGTCGTACTGGGTGGCCAGCGGGGACTTCGCCAGGAACACCACTATGGGGGGCACTGCGACTAGGACGTTAATCTTATATTCTTGTATGGCATTTAAATATTTTTTGGGGTTGAATCCTGATAAGTACACTAGCTGCTTTCTTACGATGAGATAATTGATGGTTGACATCAATCCGTACGCATGATACCATGGGACTATAGTTAAGAATTTCAAATCCGGTATTGGATTATTTTTATCACCATTTTCAAAGTTAGCTGCCGAGTATAATATGTTTAGATGCGTCAGCATTACGCCCTTCGGCAAGCCCGTGGTTCCCGAAGAGTACAGTATGTAGGCTGTATCAGTCCATCCCTGCACCTCCGTGGCTTCGAAAGCCCACACATCCGTGGCCACCAGCAACTCCCTGTACGGAATGACCCCAGCCACCGTGGGCTCACCGTCCAACTGAATTATTTTCTTTATAGATGGCACTGTTTTAAACGTGGAGTAGTTTAATTTCATAGCCGCCTCTGAACATATTAACATATTTGGCTTCGAAATTTTCATGACGTGTGTCATTTCATCTTTGGTGTACTGTATGTTGAGCGGCGTGATGGTGGCTCCGCAGCAGACCACCGCGAGCGCCGCCGCAATGTACTCCGATTTGTTTTCGCTGCACAGGGCGACAACGTCGCCGCGCTTCACGCCCAACTTCTTCAACCCCGTGCCGACGTTGACGATTTCTTGCAACAATTGACGGTAGGTTGTTTCCTGACCAGTTTCACCATTGGTCATGGCAACTTTGCTTCCTTCCTCGGTGTCAAATACCGCGCGGAACTTGTCCACAACATACTGGCCGAAAGATATGTGGGCTGGAATGTAGAACGCAGGTCCTCCGTGGACAACATTGTTTATTATTGACACCATTATTTACTGTCTAAACCAACCAAATAGACAGCAACACCTATGGAATTAACTTTCTTATCGTACAAAACACTATTGATAAGACAAGGACAACACAAACCACTAATAACTCCCATATCAATGAACGTAAACAAGTATTTCAACTAAAACACGAGCGCTGACGTAAGCGACCATAGACGCAGCTCAACACTGCATGACCATGATCTAGGATCACAGTTCACACACAGAAAAATAAAAGATACCACGACCGTAACCATTTTGTAAGTAGGTAAAAGTAGCACAGCGTTAATAAGCACAGGCCACAGAATAACTGTAGTAAAATTATTACTGTGTGGGCCGCCGTATGTGCCCATTCATGTTCCATTGTTGCTTGCTTGTAAAGTCTTGCTTGCCTCAGCCGGATTTCAATTGATTCGCACTTCAATAAAGATCACCC

>OBP2 [Moltype=mRNA] [Organism=Loxostege sticticalis], complete cds

AAGAGCAACGCTCTAACCACTAAACCAGAGAGGCGTTTAAGTCAGTTCAGGGCAGTTTCATATTCGGCTGAATGTACCAATGACAATATGTCATGTTACAGTAAGTACATACGAGGCGTTACAAATTGCTGCTAAGTGTATAGAATCTTCATCAGGTTCTATGTAAACATGCCTTATAACCCTAAGTGTACCTTTAAGGCATCCATTAGGGCGTCCAGTCTTCTTTATATTTATGGGAAAGAGAGAAAAAAATCTAGTCTTTACTCTACATTAACTATGGCCAAAGGAATGGATGGAAGGTTTTTGTTGCTGCTAGTGTTTATAATTAGTGCCTGTGATGCTATGACAAAGCAGCAACTTAAAAATTCAGGGAAAATGCTGAAAAAGAGCTGTATGGGAAAAAATCAAGTGACAGAAGATCAGATCGGCAGCATTGAGAAGGGCAAGTTTATAGAAGAGAAACCCGTCATGTGCTATATCGCGTGCATTTATCAGATGACGCAAATCGTAAGTATAGTGTAGTATATTGGGAGAAAATGAAGTCTGATAATCTCTTATCTCTCTCTCAAATGAAGTCTTATAGTCTTACATATAAGGCTATTTTCCTTTAAGATGCTACAAATTTTACCTTTTTCTTTAAAATCTTTTTTAATGACAAGGTTATGACAAACGAATGATGAACATTGTGCCAATATTGGTAAGTAACAAAAAATATTGAATTCAAGAAATTACTAATCTTGTAGTCTCGAGTAATTCCGGTAAATAAGATAATTGGATTAAAATATCGAATATTTTAGCATATTTTTTTATATCTTAAGGGAGACCCCACACTAGCGT

>OBP3 [Moltype=mRNA] [Organism=Loxostege sticticalis], complete cds

TATCCGGATCCGATTTAAATCCGTGAAAATTAGAACCAAAAAATCACGGTCCGAACTACAGGTTAGCACTACGTCCGATCCGAATAACGCTCATAAGGTAAAAAAATATGGCCTTAAAGTGAACCGACCGAACCGCGCGGTTAGATAAGATGGCATATTATATTAAAAATTTTTGGTCGTATTGAAATAAGAAGTGCAAAGACGATATTTTCCAAGATTGTATGTATGTGTGAACTCCCTTTTCCGCTCTTAGACGCAGGGTCGGTCCTTTGTGCGTTCCTTGCTTAGACATGCTCTGCAGAGAGGGCTGTCCGTGTTGCCTAGGGTGTATAGGTGTTCGTTAGGAGAGCAGTGTCCGGTCACAGTACCTACGATGATGCGTAAGTTTTGGCTCAGGCTAAGGCTAAGAAGGCTCACTCCTGAGAGAACCAGTTTGGATTGCCTGCAAGCGGTATTCAGCTCCCATCGTTCGTTTTGTTCTGTGAGTGAACGATGTCTTATCCAACCACGCAGTTGGGAGTAGGGCAGAAGTAGGATCAGAAGTGGTAGAGCTGAAATTGATGTGTCCCGTCCCGGCACAAACAAGTTACTGAAATCATGTCTACGCTGCAATGAATATGGCAACGTGACGCGCGACACGCGTCGTGTGTCAGTGGAGCCACGTACGTACGCGGCAAGTGCATTACGAAGACATTGAGCAATAGTGTCGCTGTGCCCCGCCGGCACTCATCCATCAACCGCCAGCCGCATACCACGCGGCCCACTGACACGTTCATAAACTTACACGTGACAGGCCAAGTTTTTCACGACACTAGCGAATGTTTTGGCCATAGCGGTATTACTCTATGTTTTTGATGCTGATTTTGCAAAGTACGAGTAAAAAAAACTACCCGTGATTCAGTTTCAGTGCACCTCATGGGCTTTAATTTTCACCATCATATCACTTAAATACAACCTAAATAAATAACTTGCTTTAGATACAAATTTGCTATTTTAAATATTTTGGAAGCAAATTACTCTCGAGTCGAACTGTTCGTCCATCATATGGTAGCTCTTGATTTACCTACCTTCGGCCCGTGTCGGTAAATCTTAGTGGATTTAAAGGACACCCTGTCTACTTTAAGTGACATTCTTGACACAAAATATACAGGGCATAAAGTTATGAAGTTAGTAAAACTAAATTAGTTTGCGTTTTCCACCAAATTATTATTAAAATAAAATTTTATACCGTGTAAAAGTGGCTTATCATACGCGATGTAAAATCGAGAGCACTTTCACGGCGTAGCCGTCGCGTACCTTTTTGCAAAACGAAATAATTTGAATGGTTTTCCTCATGGTCTCAGAGGAGATTTGTTGCGGTGTTTTTCTTGTACTGTATTACTAAATCTCATTTCAGGAACTGAGCGAGGAAATCAAAGAAATAATACAACATGTTCACAACGAGTGCGTCGGTAAGACCGGGGTCGCCGAGGAGGATATAACGAATTGCGAAAACGGCATATTTAAGGAGGATAAGAAATTGAAGTGCTATATGTTTTGCCTGATGGAGGAAGCGAACCTCGTGGAAGATGACGGCTCGGTTGATTACGACATGGTCATCAGTATAATCCCGGAGCAGTACCAGGAAAGAGCTAAGAACATGATTTATTCATGCAACCACCTTGATACTCCCGATAAGGATAAGTGTCAAAGAGCTTTTGATGTCCACAAATGCTCATATGATAAGGACCCCGATTTCTACTTCTTATTCTAAGATGATTCCACTCCATTGAATGAAGATGGCGAGTTGTTGACAAGTGTACATTAATAAACTTTTTCAATATTTTTAAGAGCAAACATTGAGATCTTTCATTTCTTTCTATTACGATTGACAACTTACTATAGGTAAGTCTCGTAAAGTCTACCCCAACACCGAAAGTCATTTCTATGACGTGTAGGTATACTCGTTTGTGGTACTTATGTGAAATAATAATTCTCACTAAATGAAGCATGATTGCTGTAAAATGCAAAGATGTTTATTAAATACTTTGAGGAAATAAAGCCACAAAATTAGATACACAAGTATTCTATCCGACTCCCCAGTGAGAAGAAAATGTTTAGGTAGGTACCTACTTGTAAATAAAGACGTAATTTGGCGTTAGCCGGAGGCCCTCAGCCCGTCTATTTTTTATGAAATCTCGCTCGAATTTATTCAGATCTTGAAAGAAGTATAATGCTAAGGAATTAATTTGCTTCACGAGAAATAATTTGTTCGCGGAAAATAAATGAGTTCGAATCATGTAAAACGCGTAATTGTGATCTTTAGTTAGAATTAAATTCAAAGAAACTCCCATTGCAAGAAGCAATTTGAAAAATCGTGATTGCGGCTACCACTCTACTTGAATGTTATTTTACGGCATAAAAGAAAACGATGCTTCAGGTATTTTTAAGAAACTCATAATACAGAGAATCTCGTACTTACCTGTTATTAATTTATCAAAGAAAATAATATTACTCAAAGCACTTTTGTCATCTTTGTTAACATTGTGTTTCGGATATTTTATTGCTTGTTTAAGACAAAGATGTAGCTTAATATAATATCAGATCTGGGTATCTTCTTGTTTTGTTTTACTAATCAGTAAATATAAGTACTTGAAAAAGTATCAGCATGGAGATTGGCATGGAGTAAGTAATATGGTTATAGTATTTGCTGATTGTCATTTTTATAGATAACATTAATTAATTAAAGATATATGTTTTTGTACCGTACCAAAAAAAACCTGTAATCAATGTAATAAATTATAGTAACTGCTAATCAATAAAAAACGTACTGTAGCACAACCGCATAAATGCTGATTTGATGTGCCGCGACAATGACGGTAACGGCTGCTGATGTGGAAAGTGAACTTCAAACTCCAGTTACAATAAAAGAGCACGTTATTTAACAGCAAAACGACATTCAAAAGACGGAAAAACCGAGGGTCGGAACATCTGCACATAAAGAGGCAATTTGGAGATTGCCGGGGGCGCGCAACCGCCACGGAGAATTGTACTCAAATGTATTCAAAATTAAAAAAACGAAGGAAATATTGCAAGGAAATTCATTGCGGCAAAATAAACAGGAGTGGCTGGCTCTGTGGCTGCGCCGGGACACCTACAGCTTTCAGAGGGCTTGCCCCCGGCCTGCGGTCATTACGGGAGATTAATAAACCGCTTTTATGTGGTTCTGGATTGCAATTATGAAATATTTCTCATGTTGTTGTCATGGTTCGTAAAAAGACCGCTAACTGACTCCCGTCCACTCGTTTCAGTCTTAGGAACTGGCCCCACAGACACGGAACTCTTACGCCAATCACTGAATATGTATGGACAGGACTACATTTATTCAGTGATTGGCACTGGAATGTCACCTTTTTAGGTCCAGCTACTATTGTTGTGTAAACTAGACATGGAACTGTAACATCAGAACAAAATAAATAAACGATTTCTTATACATCAAGAGACTCGCACATGCTAATTGGTAAGATTGAGCAAGTGCGACACTAGTTTCATGCCAGCCAAACCTCTCGATCGCACGTCAGTAGAGCCAGCAGCCTTTTTTTGGGTAACCACTGCCAGTTTATTTGAGTCCATATGAAAAGACAATAAAAGATTAAAAATCAAATCTGCATATAATGACAAAAACATCTGCATATAAACTAGTAGAAACTCCTTACCGAATGGCCACTGGCAAGATTGAGCAAGTATAGTGGTGGGCCAGCGGATACGGCAATTTGCCGGCGCGAAATGGCTCCGGCCGCGGCCGCAGCGACGTGCACTGCCTGCCGCCCGGGTTAGCCGCGCTTTCAATTCTATAATTCTAAAGCTTGGGCTGTAGCAAAGCGACGCCATGGCAATTGCACTACTTACCACTTCCATCTTGCTAAAGTATGCAGGTAACGCCATTTACAAGTTAGTATTTAGATGGAATTCGAAGAGATCACTGATCTTCTGGGTAAACCTAAGTTATTTCATCACATAATCTATAACATTTACCTGTTTTGTTTATCGGATTTAATGGTTATCATCATGTTTGGTTTATGTTTCTCAAATGTAGCAGCACGTAGCAGTATTATTGTACAATTTCTGCTTTCAGAGGCTATTGTTTTTTTAAAATATAGGAGTTAGTAAATTCATTTCATGAAAACGAATAAGCAAAAACTAAACTTTAAAACATGCCACGTGTTGTGTGACTGTAGACGACTACGAGTCGAACGGAGCAACGTGCTACGAAAGCTACGTGTCTACGACGCTACGGGATTGCTACGAATGTGACGTCGAAAATCTGAGGCGAAAATGCTCCTGTAATTAGGTACTACGAACACGTTCGACAAATATAATATTTCAAAAGCGCTTAATTGTATAATCTGTCATAAAGTTCTGTATTTAGGCGTTGATTTTATATTGTACATAAAGTTAACCATGCGATTAATGTTTCATTTCAATTAACC

>OBP4 [Moltype=mRNA] [Organism=Loxostege sticticalis], complete cds

TTTGGCCGCCGGGACAGTGTCTTCAAGTGTGGTACATAGCTTGGATAGCAATATTTATTATTTAGAAAAATATTTTTAAAATAATGGCTAAGTTCACGATTTTGTGCCTAGGTGTTTTGGCCGCCGCCATAAGCAGTGCTAGAGCACTTACCCCAGAGGAGCTGACGAAAATCGAAGGCGACATGTTGGTCCACGTCCAAGATTGTGCCAAGAAGTTTGACGTAGATGAATCTGACCTGAAGAAAGCCAAAGAAGAGGAGAACATTGACGGTGTCGACCCTTGCCTTATAGGCTGCGTCTTCAAGAACATTAAGCTGGTTAACGACAAGGGTTTATACGACCCTGATGTAGCCATCGAGAGCAGCAAGAGCTACCTGTCCGATGATGCGGACAAGGCCAAGTTCGCAGAAATCGCCAAGGACTGTGCTTCAGTGAACGACGAGTCGGTGTCTGACGGAGAAGAAGGCTGTGAGCGCTCCAAACTCCTTCTGGTTTGCTTCGGCAAGCACAAGCATCTCTTGATGAAGGAATAAGGAATGTTGAAGCTGTGATGACAACTACACCGATTAGGACAATCCGCGAACTCTCCGAATTTACCTAATACGGAACTCTAAACTAATGTTTGCTCATATATTTGTTTATTACATGATTAATGTTTAACTGAGTCTTTCATTCTAATTATTATAATCTCTCAGATAATGCTTTTTTTAATTTATCCATTATAAATTTCATAAATAAAAATTGTATATAATGACAATAGCAAAAAAAATTAGCACTACCAACTAACCAATATTAAGTCATCGCATTCGATGCATTTATAACTTCCTCATAACGACGATAAAGTATTTTTTTAGGGTTCCG

>OBP5 [Moltype=mRNA] [Organism=Loxostege sticticalis], complete cds

GCTAAAGCTGACCGCTCAGGTAGTCAGCACACGAAGGCAGCCTCTTGGTCACGCTACTCGTGTTGTAAATAATAAATAAATAAAAATCAATCTTAAGTATTGAGAATTAATTATGAAGTTTATCATTAAATATGTTCTTGTTTTGGCAATCACCCTCGTCTGTGATGGACTTGTGGACATAGAGAAATACCTAAAAATATGTGATCGAAATTCCGTAGACGTGAACGACTGCTTATTGGAAGCCGCGCAAGAAGGCCTTGCTGTCCTGGCCAATGGCATCCAAGATCTAGATGTGCCGTCAATAGACCCTTACAACCAAAAAGATTTACGTATAGAATATAAAAATAACCAGATTTATGCAAAGTTTATAGCCAAAAACATTTATGTAGAGGGATTAAAGGAATCGACAGTGCATGATGCAAGGCTCCGTGCGGACGAAGACCGATTCCACCTAGAGTTGGATCTGACTACTCCAAAAATCAATGTTCGAGGACAGTATGCTGGCGAGGGCCGGTATAATTCCCTGCAGATTCAAGCCAACGGCGAATTTATAACAAACATGACGGATTTGGTTTACACATGGAAACTCGACGGCGTTCCCGAAAAAAACTGAAAATGGAACTTTTGTACGCATCACTGATTTCTACATGCGGCCTGACGTCGGAGACATGAAATCAACCCTTACAAACGAAAACCCAGACAGCAAAGAATTGACTGAGCTGGGCAATAGGTTCACCAACCAAAACTGGAGACTTCTCTACAGGGAGCTGCTTCCATACGTCCAGACTAACTGGAACAAAATTGGGAGCCGCGTCGCTAACAAAATATTCCTCAAAGTACCTTATGATCAACTGTTTCCAGCCAAGTAGTAATTTTCGTTTAACTTAAACATAGTTTTTTTATTTGTTTTTTATTTTAAAAGGGTTAGATAATTATGTTAGTACCTGCGGTCGTCAGCCATTATGATGGCTTTGTCTGTGATAAATTGAACAATTCAGTACCTATGTATTTTAATTAGTAATAATAATATTTGATACCATTATACTGTTTCTTATTTTGACTTGTTTTTCATTATTTATCAAATTAGTAAAAATGTTAGCGGCTTGATGGAGGCAAAAGCGAGGTTGTACTTATTTTTACGGGTGGCGCCTCACGGAAGTACAAATGCACGTTAATATGCTCGTTACTATCAACACGAAGCACGCCAAAGGTATGTATATAATAGCAGGGAAAATACAATTACATAAAATTCATTGATTTCGGTAATTTAAATCTGAACAAAACAAATACAATGGCTATGGATGGAAAGATTGAAGCGTGGACAGGACATACATACCTACCACAGTACAGTTTTGACAA

>OBP6 [Moltype=mRNA] [Organism=Loxostege sticticalis], complete cds

TAGTTTCTTTAGCACTCGCGTGTAAATCATTCCATTATGATTGTGAAAAAACATCACTGTGTTTTCGTTAATTTTTTCGTCCTAATTTTATTAATTGATGTGAGCTTTGGGATGACAAGGCAGCAGCTCAAAAATTCTGGAAAACTCATGAAAAAGTCTTGCATGCCCAAAAACGACGTGACTGAAGAGCAGGTTGGCGAGATAGAGCAAGGCAAGTTCATAGAAGAGCGCAATGTGATGTGCTACATTGCCTGTGTCTACTCCATGACTCAAGTTGTAAAGAATAACAAACTGAGCTACGACGCAGTCATCAAACAGGTGGACATGATGTTCCCCCCCGAAATGAAAGACGCTGTTAAAGCTTCGGCTGCGCATTGCAAAGATATTTCTAAGAAGTATAAGGATATATGCGAGGCATCTTACTGGACAGCAAAATGTATGTACGACTTCGACCCAAAAAACTTTGTGTTTCCATAGAAGACAGAAAAAAAACGTTTATAAATACCTAATGTTGATTACAAATAGAAATATAATTAAAAAAGTATACAAGTTCCATAGACAAAATGCAAAAATATTTTGCGTGTTGAATACTGCGCAATGCTTAGCCTTGTAATCGAACCAACCGTTTATAATATTATGATACTTGATTAAATTACAAAATTACAGCTTTTCACTGACTTTATACATAACCCTAGCATTACCAAAATTGAGTCTAACAATAACTCAATCGAAAATGAATGACAAACGTTTTAACCCGTTTATTAAGTTAAGAAAATTACAGGTTTCATAAAAAGCCGTTTGCGAAAACATCCATATATTTTTGGCAAATCGTTTTACCGGGTTAGTCGGATTTTATTTCTAGAGTTTTGTAGTCCATCCCCATGATGATATTCAGAATGAAAACTCAAATAGTGTTTTTATTAGTATTGCTTTACACTGAAGATGTATTTGGGGTGAGTATTATTAATATTAGGTACTATAATTACAAGATTTTTAGGTGATGTATTCCTTTTTTTAGTGGAATTTAACAATAGGGATGTGTGTGTTGCCACC

>OBP7 [Moltype=mRNA] [Organism=Loxostege sticticalis], complete cds

AAAGTACTCTGTGATACAAACATGAAGACTTTTATCGTGCTCGCCGTTTGCTTTGTGGTCGCTCAGGCCTTCACCGATGAACAGAAGGAGAAGCTCAAGAAACACAAGACAGAATGCCTCTCAGAAACCAAGGCGGACGAGCAGCTGGTGAGCAAGTTGGCGACTGGCGACTACAAGGCAGAAAACGACGCCCTGAAGAAATACGCGCTCTGCATGATGATCAAGTCTGAGCTGATGACCAAGGACGGGAAGTTCAAGAAGGACGTCGCTCTGGCCAAAGTGCCTAACCCAGCTGACAAGCCTCAAGTAGAGAAGGTGATCGACGCCTGCCTAGCCAACAAGGGCAACACGCCCCACCAGACCGCCTGGAACTACGTCAAGTGCTACTACGAGAAGGACCCTAAACACGCCATCTTGCAATAAACCGGCCAAATCTAGAATCCAGGCAGAAGATCTTTTTTGTTTTAGATCTGTGGCGTTGAGAATTTTAATTCATTTTCTTTAGTGTGTTTCCTTAATTCTTATTTGCTGAGACTGGTCAATATTGATTGTTCACGTTAATTCTGTAGCCTGCTAAATTGGTTAAAAATATGAAGTGGGAACATGCAGTTAAGACATTAGTAGATAGGTAGTAATGTTGAGTATTTATGCCAGGAGCCCAAACTCAAGCCACATTTAAGCAATTTAGATTACCTGCGATTGTTTTGAGAAAATTCAAATCAACTGTACCAAAAAAAGAATTTCTAAAATATGGCTTCCTATTGCAGCCCACAAACAAACTTTATCAAAACTAAACTCGTTACCTATAAATTAATCTTTTAATTCATCATAACTTTGCAAAAATCATGTTAACTTATGAAAATGTGACTCTTAATACAAGGAAAATTTGCTTATAATATTCTGAAATACTTACCATGACTGCCTGAACGAATTTAATTAGTTAGAAGAAATAAAAAATGAAATTGCAA

>OBP8 [Moltype=mRNA] [Organism=Loxostege sticticalis], complete cds

GAAGCAAAAATCAACAAGTATTTTAAACTGTTCAGTAGTTAGTTTAGGATTCCAAAATGTTTTGTCGTGTTATTTTGCTGTCTAGTGTCTATTTTTTGGCTTTGACCCCTTATTCTATAAATGCCATGACTGAAGCACAGAAAGAAATGATCAAGCAGCACTTCGAACAACTTGGCATGGAGTGTATAGGAGACAATCCAATCACTGAGCAGGACATCAACGACTTAAGAGCGAAGAAGGCGCCTTCAGGACCTGGTGGTCCCTGTTTCCTAGCTTGTATCCAGAGAAAGATTGGTGTGATGGACGAACACGGTATGATGCAAAACGAGAACGCGTTGGAACTTGCAAAAAAAAGTATTCCAGGATGAAGAAGAGCTGAAAATTATTTCCGACTACCTACATTCGTGTAAGAGCGTGAACGATGTCTCTGTCAGTGATGGAGAAAAGGGCTGTGAGCGTGCAATGGCCGCCTTCAAATGTATGATCACAAATGCACCAGCGTTCGGCATCGAAGTCTAGAACACGAGAAGACTGCAAGTGATTAAATAACAAAACTGTTAAATTCGATTTGATTCTAAAAAAATACTATGAAAATTGTATGGTGTACAATGTACTTATTGGCTTGTCAAATGGAAAATGTGATTAATTTAGTGTAAAGAAATAAATTTAAGTGCAAGTTTAAAA

>OBP9 [Moltype=mRNA] [Organism=Loxostege sticticalis], complete cds

GTGCTACGAGTGACGTCACCGCCACCGCTACGGACGCTACGCGTCTACGAAGGCCCGACCGCTACTGCAATTATTGCAAGAGGTATGGGCATACTCGAGACGTGTGCCGAAAACTTATTGGTGAGTCTTTGAACAGTTTAAACTTTAACTCAATAGACCCAAAAGTGCTACGGTGTTATAATTGTGGACATGAAAACGTTACGCGCAGTCAGTGCCCGCGGTGTAATCGAAATAGTGATGAGAATGCGGAATCTTTCGATTCATTGAATGTAAATAATGTTAAATCTTCTCAAACTTCGCAAAATGTTGACAATGATGATTCTTGTAATAATTATGTATTTGGAACCATGTGTACCACGAGCGACTGCGAGAGGCCTGTAACCCAACCGCGATACCATAGGGCGCGACCAATTTTAAATATTGAGATTTTGGGAGTACGGGGCTCCGCTCTTTTAGATACGGCTGCAAAACATTGCATCGCCGGACACTCACTTTACTCTATCTTATTGCGCCGTGGTCACCACATGTACCCCTCAGTACGTAAGATTAAACTGGCTGACGGAGTCGTTCGCGAGATGAAAGTTTTGACTTGCGAACTGAACGTAAAAATAATTAATAAGACAGTGACGATTCCATTCATGATTTTCCCGGAATCGAATAATAATGAAACACTGTTAGGTATTGATTTTATCAAGGCCACGAAATTAATACTAGACTTCGCAACGGACAAATGGTATTTTGCGAATGATGTGCATAACAAACACAGTTTGCGGTACGAGTCTTGTGGAGACCATCATTCGTGCTCATCTGCAAACGTCCTGGGGGATGATGAAGGTAAGTATTCCACAAATTTGAAATACGAGAGCGTTTCCCGTGTAGGGGGAGGGCCCACGCCTTATGCAGAGCACCGAATTGAAGTTGGTAAGCATCAGCCCATTTCTGTACCTCCAAAAACACGAGCCCCCGCCAAGAAGGTGATGAAGGAAAAGCAGATAAAGCGTACCGGACCGTATAGTGTAGCGAAACAGGTAAGTCCTGCCACATACAAATCTACTCGCATGCCGTACGCTCATGACATCTCCGTACAATACCATGTCCGCGATATAAAGCCATACCATGATGGAAAGGGCCAGGAATTTAGTCAACAGCCAGCCCATCCACATAGGAGAATAAATTGGCCTATTCATGCAAGTCCAGGAGCGAGGTCGTTCTCCTAAACTTGAGGGGGAGTATATAGCCAACCCTAAATTTTAACGCATAGAACAGATAGAATAAACTAGAAACTTCGGCCCCTTAAGATTTCCTGAAAAGGAGATTCCGACAGTTTGATCTAGAATATTCTAGCCAATAGGCAACATTTTCGTTATCGGCCCCTCGCAGCCGCCGACCAATCGCGTCGCTTTCCAACGGAGTTGCGGACGCGCGGCCATGAATAGCCGCGAGTCAGTCGAGCCGAGTCCTTTCCGCGAGAGATACGTTCCGTCCGCGCGACCGCTGCTCCGCCGCCCGCGGTGATATATTTTCCTTGCCCGCGAGGCCCCTATTGACTTTATTTCTTTTGTTACAACTTTAATTGCCAAACCAACAGACGCTTATAAGTGAATGGTGTTGTGTTTGTGAACATTTTGAAACTGTGAACTTTCTTTTAATTATAAACAATTATCGTACCTAACTAACTTGTTTCTTTTATTTAATTAATCCAACCTAGCGAAGGACCCTTGACTATAGTATTCATTTGTACCACACAATAAATGATTCCCATTAACTGTTGAGACCTCATCAAAGTATTACTTATAACGTTTAAATCAATCAAACAATCACATATGTTTGTGATGTGTTTAGTTTGACATAATGTAACTTTATGAATGGAAATAACAAATTAACTTTGATTGATTAATAGTGGGCAAATAAATAATAATATCGATGTTTATGCCTTTATTCATATAGACTTTCCTTATGAAATGTGCTTTTTGTGCAGGTTTTATTATTATTTTGCGGCCTCATTTAGCGTTAAAAAACAAATTACATTGATACCAACTTCGCAATTCCCAGTTGGTGAATGCATTTATTGTGATACAATGCAGGACACGTCATTATCTTTTCTCTGCAGAGCAATTTCGACCTTGCAGTCAATCAATCTGTCGCGCCTAATAACCTGGTTTCCTGTGCAGGTTCCATTTATATTGCCAGCTGAAATAGTATTCCTTTAGATCGGCCAGGTGAGATGAGTATAGTTGTGGTCTTTCTGTCGCTGGTCCCAGCGCTGGTCAAATGCTCAGGGGAGGGCAACATTCGACTGCTGGAGGAGGAGGTGGCGACGGCGATGAAGGCGTGTGCTGTGCCTTCTGAAGACCCTAAAGATGGTTCAGCGGCGAACCAGCGCCAGCGCAGATCGGAAGACTACCCCAGCGTAGACAACAATGATAACAACACTGGTCAAAACGTGTACAGCTACGAGAGACGTGTCCTCAACCTGACTGACATCAGAGACCAGATGTACATCCTGAACGCCACCGATTATGACTACGGAGGTTACGGAGCCGGCAGTGCGGGCGAGAAGTACCTCTTGACAGTCCCTAGACCTGCATCAGGAAGAAGCTATTATGGAAACAGCAGTGACAACGCGAACAGGACCAGGCGAAGTGAACCTCTTTTGAAGCCAGAATCCAATCAGTGTCTCAGCCAGTGCATATTTGCCAATCTGCAAGTGGTGGACTCTAAGGGAATCCCGCGCGAGGCTGAGCTGTGGGGCAAGGTGCAGTCGTCGGTGACTTCTCAGCAGTCGCGAGCCGCGCTAAGAGACCAGATAAGGGCCTGCTTCCAGGAGTTGCAGTCGGATGCCGAAGACAACGGCTGTTCCTATTCAAACAAACTGGAACGATGTTTGATGCTGCGCTTCTCCGACCGCCTGAAGGCAGATAGAAGCAAGACGCAAGCAAACAACCAGAAAACTTAATAACTTTATCAAAAATTCAAATGTTACTCTTGTTATGTTACTTACCATAAATAACAGTGAGATAAATTAATCCTAAATAGATTTTAATATTTAGTCAATAGGTAGTCGATTTTGTTGCAAATCAGAATAAAGTCTGAACAAACCATTGCCTTTATGTTTTCACAAGGAATCGAATGATCAGTTTATCATATTATCAACAACAAAGCTTCCACAACCGAATTATTCGTAATATCAAATTATGCAACAGAATTCGAAACATTTTGTTTTCTATCAATCAAAATATTAACAACAATCGTCTTCTTATCTTGTAGACTTCTTAACCCCTTTTATCACAATGAGAAATCTCATAATAAAGGCGGTACAGTGGGATTTTTTTTCCGAGCTCTATCGAATCTTCGAATTCTTTACATAAACATATTTAGAGTTCTTTGCATGTGGTTGCCAATACAACTCTGGGATCACGGTGCCATGGTCAGCGGTCTTTTGAAAAAAAAACGCATCTGATACCTAGTACCTAGTAGTAGTAGGCCAAGTTCCATTCAGGATTAAAATAAATCACAACTTTTTGTTATTTATTCATAAGTACAAAGTAACTTACTTGTTCGTGTATAACATCTCATAAGTACACAATAACTGGATAGCCAGGAAATGAGAACGGTTGAGGATGAAACACAATAGGGCGGTCCAGGCTTGTATTTTTTAATTCATGAGGATATAACTGTTTAGGCAAAAGATCTATGGCCTTGGCATCCTGAAAAATAAACTAATATTACTCATAACCCGGAAAATAACTCAAGTCTTACTATTACTTTTTTACGTCGCAACAGCTAAACAGATTATGATGATTTTGCTAAAGAAAGATCTGGCGATAGAGGACGTAGGCTACTTAAATTGGCAAAAATAAAACTTGAAGAGCCCCCTCTTTTAAGAAAGTTACTCAGTTAAAGAAATTGTGTGACAAAACAAATCCCACGCAGATAAAGCCGTGGGTAGAAGCAAGTTACATTGTGTATATTTAGCATTAAAAATAAATTAACTTACACAATCAGCAGCAAAAAACAATAACAAGAAGTAAAAAAGCATTTTAAGTTAACATTCACAAAACAAAATCCAAATCCATACTTTCATGAATTTTATTTTACTGCCAATAAAATGCACACTCAATAAAAACATTCCATCAATGTTTTTAATTAAATTCTCATGCCAGAGTGAGTTTTGAGATAGACCTTAAAGTAATAAAAGTAGTTTTATCTGTATAAAATGTAGAGATTTTCTTGAACTAGTAAAAGATTGTCATACATTCTATGTAAAAATTATTTAATAAGTTAATGTCACATAATTATGAATAATTTATAAAATATGAAATAATATTCAATGCACAGCTGGTGCAAGCAAAGACAGAGTCAATGTATTCAACGGTAGAGGGGGTTTGCTCGCACACACTCTTGGTTGAGCTTGTATGTGTTTAGACCACCGACACACATGCAGGATTGATTCTCGCTCCAACACTGATAAACGTTCAATTATTGAATACAATACATAATATGCTGCAATATCTGCTACTGTTAGGAACTGACCAGTCAGGAATGTATTTTTTTCTAGAGCTTTATTAATGTCCTGTAAGAAGTTTTTAGCGAACGTGGGATTCGTCGCCGCTTGATTCGAATACATAGCAATGTGCTCTAGCCACTGGTAGCAGAGCAGTGTCTGCTCCTGGCTCATTGTACTACCACTCTTCGATGCCAGTCTTAGAACTATACTTGCAAAGCCTTCTACATTCTGCTTATCGAGAACAGTTGTCAACACCTTATCGGTGTTATAACATATTGGGCCCACTGGTGTATTTAGATACTTTCCCACCAGTTTTACTACTTCGACGTTACAAGCACACATTTTTTTACAAATGAACAATGTCTGGAAATAAATATTTCACTGAATTTTGCTTTTTAGATTGCAATTTGCAAGCGTGCCCTCAT

>OBP10 [Moltype=mRNA] [Organism=Loxostege sticticalis], complete cds

GCAATAAAGTTTTAAATAAAAAAAAAAATAGATTTCTGAAATAATTAAAAAAAATCGGTATTACAAAGTTCGGTCAATCTCTGTGCTAGCTAGTAAAAGTAAATAATAAAAGTACTTTTTATAACATTTATTACCCATTTCGATTGAATATTGATTGTGCTAAAATCTGCTCTGACTCTGTACGTTCGCATGACATGTTTTTGGATCTGCCTGTACGTTTGCAGTTGAACCTTGGACAGGATGTATTGACAGACTTCCTATACTACTGGCAAGAGGACCGTCAGTTCACCAACAAACAGGTGGGCTGTACCGTCATCTGCGTCTCGAAGAAGCTCAACCTGTTGGACAAGGCCGGGAGGCTCTCTCAGCCCGACGCTGAAGCCTATGTGAAAACCGCTGGTGGTGGTAATTCACATTTTTTTTATTTATCTTCTACTTCTCAGCCAACGTCTACTGCTGGACAAAGGCCTCCACTACTATTCCTGCGCTGCCCGCATCCATTCACTTACCAGCGACCTTAGTTTTTTTTACATTCATTTTAATATTTTTGATACCTTTAGTAAATAAAATCAAATGAGATCGCCCTGTTCCGCAAACTCCTTCTTTAACTTCTACTTAATTTTAGCCTTATTCTTTAGTTTGCTGCTTGACTTAAACTCATGTTATTTTCCATCTATCTCTTATTTACACCTACCTAACTTAAGGTTTTGATGATTTCCTGAAAATAGTACCTACTACTTTAACCCTTATTCCCTTTTATTCTTTTGAAAATAGTTGGATTCTTGGCATGATAAAAGCAAGGGAAAACGATTTCTAAGAAATTGCCAATGAGCCCTTGCTCAAGGCATAACGCAATAAAAAGAAAGCCTTCCATATCCGCCAATTTTTCTTAGCCTCTTTTAACCAATTCCAGACGATAATTTAGCAAAGTACCTGGTATACCTCTACCAGAGCTGCCAAAAGGTGGTGAATGAGACGGAACCGTGTGCAAACGCCTTGGAGACGACTAACTGCTTTAGGAAGGCAGTGCAAAGAGCAAAATATTCCCCCGATGTGCCCGTAGAGGTGAGGCAGAGAAGCGATGGTTAATATTTTGTCTATGGTCGGTTTGAATGAATAAAATAATAAATGAGTATCTAATCCAC

>OBP11 [Moltype=mRNA] [Organism=Loxostege sticticalis], complete cds

TGCGCAGACAGTGGCGCGGTTTGTTGGCGATTGGACCTTAATTTGGTGCAAATATCGTGACCATGTTCGGAGTGATTGGGTTATTCGTGCTTATGTTTGCGACCTGTCGCGCAAATGTTGCTGTTTCTCGGTCGGATACCCCACAAGTTTTATGTGGACTAATCCCGGACAAGCTGAATTCCTGCGGTCATTTGCCTACAATCGTTTCTGCCGAGTCAGCAAAGAAGTGTGGGAGCTCAAGTAACTCATGCCAAAGAATGACTTGTATCTTCCAAGAATCTGGCTGGATGGACGGAAAGAGTGTGAACAACGCTAAGCTGAGCGAGTATCTGGACCACTTCTCGAGCGAGCACCCGGATTGGACGGCGGCCATACAGCATGCGAAGACTACTTGTCTCGTGCCAAACTTGCCTGCTCAGGGCTTCCATCTCAACTGCCCGGCGTATGACGTCGTGACATGTGTTTTTCGGTCTTTCGTATGGAACATCCCGCCGTCCCTCTGGTCCTCATCAAGTGACTGCGAGCCGGTGCGGCAGTACGCGGCGGCGTGCCCAGTGTGCCCGACGGACTGCTTCTCGCCTGCCATACCCGTTGGTTCGTGCAACGCTTGCCGCGCATTGCCGCGCTCGCCTTAGCCTAATTGGATGGTTGAGAAACCTCGACTAAACCGTTAAGAGTAAATAATAA

>OBP12 [Moltype=mRNA] [Organism=Loxostege sticticalis], complete cds

CCCAAAGAGTTTGCGCGGGCGCAGCGAAAGTGAACATGTGTCATAGTCTGCTTTGCATTGTTATATTTGCTGTTATTGTATTGGATTGTAATGCCCTGAACTGCCGATCAGAAGGTGGACCCAAAGAAAATGAGCTCAAATCCGTATACATGACATGCCTGAAGAAGCAAGATGGAAAGAATTCAAGCGACTCACACGGGTACACAGAAGACCAGGATTGGAAGGAAACAAGAGGCCAATCGAAGTTCCACCATCGATCGAAATGGGGCAGCGGAAGCATGGGTGAAATAGATGATAGAATGAGAGATAGAGATGATAGGATGGACGACAGAGATGACAGAACAAATCGAGAGGATAGAACAAGAAGTCGTGATGATAGAATGGGAGGTAGAAATGATAGGATGGGCGGGAGAGACGATATGAATAGTAGGGATAGAATGGGTGGACGTGAAAATATTATGAACGATAGGAATAACATGATGAATAGAGATGAAGATGCGAACAGATATGGAAGGGAACCGTTACGTGGACGACATGATTTTCCGCAAAGTGATGAATACGAAACCGACATGACCCGTTACGGCTACCACTCTACGACCCAGTCAACTCGAAGGTTCAAGAGGAGCAGACGCACAGAGATAAACTCCGGTCAAAGGAGTCAGTACAACCCGAATTCCAGAAAACCTTCACAGTATGAGGAAACTTATAAAGATGAAGAAAGGAACTCCAGTAATTCTAGCAGAGAATCAGACAACAAAGCCTGTGCTCTGCACTGTTTTATGGAACAACTGGAAATGACAGACGATAACGGCATGCCAGATAGATATCTAGTAACACATGCTATCACAAAAGATGTAAAGAACGAGGACTTAAGAGATTTTCTTCAAGAATCCATTGAAGAGTGCTTTCAGATTCTTGACAATGAGAATACTGAAGATAAATGTGAGTTTTCAAAGAACCTAATGATGTGCTTATCAGAAAAAGGACGTGCAAATTGTGATGACTGGAAAGATGATCTTAAATTTTAAAAGAAGATATTGCCTTGCTGTTTCGTCATTTCAATATTTTGTAATCTTGGAGTGCAGGGATAATTTTTAAATAAATAATAAGTATACTGATGTGTTCATTATCGTTATTTAAATAAACACAGTACCAACCTTCTTTTTAAAGTACCCAGGGAATTCCAAGATATAAGTTAGTAATCCTTTTGATATAAGTCTAATCAATAATTATTATGTACTTAATTCGTCAGTTACCATTGGAAACATTTGAATGTTTTCAAAAGGGGGAAATGGTCACATCAGCCGAACCATGATCTTTTTCTTCTATTAAGAAAATATATAACGATGGTT

>OBP13 [Moltype=mRNA] [Organism=Loxostege sticticalis], complete cds

AATTTCGATCACTTACTTGGGACAGACGCAAGCGATTTGTTTATAGTAAACCATATATCACATAATTGGATATAGGTCGACAACTATTGATATCGAGTCCTAAAGTATTGACTTCCGTTATTTTCTGTCTTGATTTCCTTCGGTCCTAGTATAAAAGTGCCTGCTTGTAGGCTGTAGATTGTACTTTTAGTTTAACTCGAAGTAAAACAACAATTAGAAATGAGGCCGTTTTTGTTTCTGTGTTTGGTTATGGCGGTAGCTGGTAATTCTCATCATGCCCAACTTTCTCAAGCCCAAAAGGAGAAAGTCCAACAATACACGATGCAATGCATCAAACAGACTGGGGTCAAACCAGATGTCCTAGCTGAGGCCAAGAAGGGCCACTTCTCTGACGACGAGGCTCTAAAGAAGTTCGCTCTTTGCTTCTTCCAAAAAGCAGGCATCGTGGATAGCAATGGAAAGTTGAACGTTGAGGCAGCCCTGGCTAAGATACCGTCTAGTGTTAACAAGGCTGATGCAATGAAACTTCTAGAAGAATGCAAGAAGAAGAGTGGAAAGGACGCAGCTGACACGGCTTTTGAAGTTTTCAAATGTTACTCTAGGGGCACCAAAACTCATATTTTAGTGTAAAGGTTAACTTGTTTATTTTGCGGCCAAGCTATCTGTTTATTTATTTTGATTTCTGATCGAGTTAAAATTAAACGTAAAAATATGCTCCATGTTTGTTATTCAAGTGATTTATTTATTTAATTTATTACAATGTAAATAGTACTTTATTAAACTTTGTTAAAATTAAA

>OBP14 [Moltype=mRNA] [Organism=Loxostege sticticalis], complete cds

CTTTTTTCATCTCAACACACATTCAGGTCAGAAAACAGATTGGTTTGGTAAAATGTTTCATCGAGTGCTCAGTATTATACTTTTTGGTTTGTTTACTTGCAACGTAAAGGGGGATTTTTCCAACGAGTTGCAGAAGAAGTTTGTAGGGTACCTGGGTGAATGCTGGCAGACGTATGAGCTTACTCCTAAAGACCTGGAGGACCTGAAACTGTTAAAGATGCCTGACAGTGAAAATGTGAAATGCTACTTCGCTTGCGTCTACAAAAAAGCTGAAATGATGAACGATAAAGGCGAATTTTGGGAAGAAGGTGTAAAGAAAACATCTCTGGAGCAATACGGAAATGACGATGCTTTACTCAAAAAGGTCAATGACTTTATTGACATTTGCAAAAAGGTAAACGATGAACCTGTGACCGACGGAGAAAAAGGCTGCGAGCGAGCGGCTCTTATGTTCAAGTGTAGTAACGAACACGCTCCTGAGTTTGGATTTATATAAATCTGTGATACTGTTTGCAAAACGACTGACGAATGGACTTAAAATTTATATTTCATTGGAACAAGGACATTATTTTGTAATACTTCGCAGTCAAGTGATATTTTTACTTAATCGGAAGCGAATAAATCTTAGACAAATACAA

>OBP15 [Moltype=mRNA] [Organism=Loxostege sticticalis], complete cds

TTCCATTCCATACCGCGACCAGTCGCGCACCTTTCGCCGCACGGGCCTTGCAGAAAAAAAAACACGTTCGAAATTTGAATATTTAAAATTTTGTGGTAAAGTTTGAAAAGAGGTTGGTGTTGTGTGAAAACAAAATGGTTCGTAAAATCAGTGCGCTCCTGTGTTGTCTCTGCGTTTTCGGCATCTCGTTGAGTGATAGCGCGATATCAGCTGATAGCGAAAAAAGATGTCGGAACCCTCCCACTGCCCCTCAGAAGATAGAAAGAGTCATCACGCTGTGTCAAGATGAGATCAAGCTGTCCATTTTGAGAGAGGCCCTGGACGTGATCAAAGAGGAGCACACCATGCCCGAAAAGAGGCGGCGGAACAAGAGAGAGGTGCCGTTCACTCATGATGAGAAGAGGATCGCTGGGTGCCTCCTACAATGCGTTTACCGGAAAGTGAAAGCGGTGGACGGCTACGGTTTCCCGACCCTCGAAGGTTTGGTGGGCCTGTACTCGGACGGCGTGAACGAGCGCGGCTACTTCATGGCGGTGCTGGAGGCTTCGAGGGAGTGCCTCATGAGGCACCACGACCACTTCTCGAGGACTGTGCCTATGGATAACGGCCGCAACTGTGACGTGTCCTTCGACATCTTCGAGTGCATCTCCGACCGCATCGGCGAGTACTGCGGCAACTCTGGACTCTGAAACTACAACACAATAATCACTCAACTTCTTAACTTTTTCTCAATCTGAAATTTATAAAATTCATCCCACCATAGCGATGGGCAAATGGTTACAATTGACTATAAAAAACTGAGGCGCTAATCTCCAAGATGATGCCATATTTTTGGCCGATCATAGTCTTTAGGAGTTGGTCCTTTTATGTACATTTTATGTAATCACCCCGTGATTGCCGTTTTTGGGTGTATTCCTTAGAAACACTGTCTCTTTTACTATACAACACAAACTCATATGATATGCAATGCTCCATTTCGAATCTCAACAACACGACATAACGTTTACTAGACCATTTATACTATACTGTGTTTTACGAACCAGGATAAAGAAATTCATTTTCTTTGGCTACATTGATGTTGGTTCATGTTGATACCCATACTGTTAACAAAAGACATCAACGAGAACGTATACACCACGATTTTTTAATAATGT

>OBP16 [Moltype=mRNA] [Organism=Loxostege sticticalis], complete cds

GATTGGTGGAGGGGTGTTGTAAGGCTTCACGGAGTCCAGCAGAGATCAGACAATGGATGCTGACCAAAGCCCTGGACTGTACCAAGGGTAACTTCGTGAGCTCCAAAGAGCTCCAGATGATGATGAATCACCAGCTTCCCGGGACCAAGAACTCAGATTGCTACATAGCATGTGTCTTTAAGAAAGTTGAATGGCTGGATGAGAAAGGCAATTACAACATAGAAGCAACCCATAAAATGGCGGACAAGGAATACGCAGATGACGCGACTAAAATGGAGAACGCCAAGAAATTATTTGATCATTGTAAAACGGTGAACGACGAAGCTGTTACCGACGGAGAGGCGGGCTGCGATAGGGGCCACTACTTAGCCAAGTGCCTCATAGACAATGCACCTAAGATGGGCTTCGACTTGTCAAAATATTAAAAATAATACTCCTCCGCTCCACATAATAAACTTAACGTCAAATAAAACTTTGGTTATGAGCAAT

>OBP17 [Moltype=mRNA] [Organism=Loxostege sticticalis], complete cds

GTTAACAGAAGGATGTATCTAAAAAGGACATTAGTTGTTTTGTGCACGGTTTTAGTGCTAGGCAGTGCAGCGTTTGTTGATAACATCCCAAAATGCGGCGCTAAAGACACTGACTGTCACAGGCAATCTTTCCAATATGTCATCAGAGAGGGCAGTAAGACCGGAATCCCAGAGGCAAATATTGCTCCATTCGACCCCCTAGAGTTGAAGCAGGAATTGAATATACCGATCCGGGATATCGTCCAATTGCATTTTGGCGACGGTGTCGTTAAGGGGTTAAGTAAATGCGTCATCAATGACTTTGTGACGAATGTAGAGCAAGGAAAAGCCTCACTGGATATTACATGCAACTTTACCGTAAAGGGCCATTATAAGGCGAATTCATCTAGCCCTGTCATCAAGTCTTTGCTAGGAGGCGAATCGGTGCATGGAGATGGCAGAGTGAAAATAAAAATCGTAAAACTGAACCTCAAGTTAGATTTCGACTTCATTGTCGACAAAAGGAATGGTGACACCTACTTCAAACGGAAAGGTAATAACATCAAATTCAAGTATGATGTACTTGGACAAGTGATGTTTGCTGCCGACGGCCTTTACCTTGGCGATCGGGATGCTAGTGAACTCCTAACAAATATGCTGAACCAAAACTGGAAACTCGTCATGGCGTCTGTTGGAGATGACATCATGAAAGACTCTATGGGAGCCGTGGAAGAATTCGTGCGTAACTTCTTTGAAAATGTTCCTACAAAGTACTTTATTACAGATGACTTGACGCCTTATGCCTCAAACTAGAAATAATTTTAGACTTAGGCAAATCCAATGAGTAAAATGTTGATGTGATATCCTTGAATTCCTATCTTATTTATAAATAAAGATTGATTTATT

>OBP18 [Moltype=mRNA] [Organism=Loxostege sticticalis], complete cds

TGCCCAAAGCTGGAGTAACAGAAGGTTTGTTATATCACTAGACAATTGCATTTATATTAATGTCAATTTAGTGTTTATTGCACCATCTAATACCACAGCCTATTTATTCGCCAGTACGTGAAGTGACGTTAATATCACAAATAGCATGTTTTTTGACGACGATTGGGCAATGGCCTCTTCTGATCCTTTCCAGTGCTAGACCTGCCTAGTTCGTCCTGTGAAATGCCAACTCGTAGTCCCGTCATAACCTCTCTCTCTATTGCCTCTCGTTTCAGCGATAGCCATTCTTCTAAGGCTATTGTGTCATGATTCGTCGGATCCACCTATCGGCATGATTTCTGCACACGTGGCCCGTACACTTTTACTTAATGTTGCATTAAAACACGTATTCTTTTTCAATTTTTATCTGCATGTCTCAACAGAAACATGAAATTTATGCTCGCAATAAGTTTGGATATTAACAAGCACAATATTGGTGTATTTCATTATGTTTGAAAACTGAAAAAGGGTCATTTAACTAGACACATTTGTAATTCCATATAAATTATCACTCTCGACAGTGATACCCATTATGTAACTTTCAAAATACACATTTCAGATAAAGTAAAAAATATCGAACAAGGAGAGTTTATTGAAGATCGTAAAGTTATGTGTTACATTGCTTGTGTCTACAAAACTATTCAAGTGGTAAAAAATGAAAAGATAGACAGGGATCTAGTATTTAAGCAAGTGGATATTCTGTACCCCGCTGACATGAAGGCAGCTGTCAAAAGTGCTGTCGAACAGTGCTATGGCGTCCAGGCAAAATACAACGATCTATGTGAAGCGGCGTACTACGCGGCGAAGTGCTTGTATGAAACGGACCCTCCCAACTTTGTTTTCCCTTAAAAACATATAAAAAAATCACAAAAGCCAAGATTTTCACGTAACTGCAAATAATTCTTAACTAATTAGTTTTAAGATGAGTATTTTGTTTGTGACTAATTAGCGTTTGACTGGCTTGACCTCAATCTTACCAAAAACCTAACCAAATATTGTAATTTAAGTGCTAAATAACATACCCTTAAAAAAAACCTTAGAAAATTACTAACTAATAATATTTATCGTAAAACCTTAGCGTTAATTTAAAATGAATTTTGTAAGTTACTGTTACACTAATATACGATTACCTACTGAGTTTCAACTCTTTAAACTTTTCTCTGTTCGATCACTGTTTGACTGTAGAGAATACTTCTTTGCATTAAGTTCGTCATTTGTTCTTGTCTTATTGTGCAATAAATCATATTAACATAAATGAGTAAGTTGCCACATAACTTGCCCTTAACTTTTTTGACATCATTTCAAGCAGGTCATAAGTATTTTCGGTAAGTCCCCAAACATTATGTGTATTTAATTCTGAAACAACAGTTGGTCCCATTCATTTAGTTGTTGTAATCGAGCAGTAGAAGCAGCCCTTACAAGGTTTTTTATAAACAAAAAAATATTTTGTATAAATAGGCTGTGGTATTTACAATGTACAAAACGTAAATATAACGGAATCCAAAGTAAATCCTATGAATGATGCCGTTTTCAAAGGATATGATGTTTCGAGTTTAATTTTTTTTTATAGTATCGTTAGAGCGCTTACGCAGTACGTCCGATCCGTATCAAATCCGAATCTGTAAAAATAAGAACCAAGAAATCTCGGACTGAATTTGGATACAAATAATACTATCGCTACTACGGTTTGTCAAAATATTATCCGGGATTAAAATTTACGGATGACGGAGAGAAATTGAGACCCACGGTAAATTAAGGCTGACTTTGACAATTTGGGTGAAATTAAATGTCAAATAAGTTAGGCCGGCCATACACGGACTGC

>OBP19 [Moltype=mRNA] [Organism=Loxostege sticticalis], complete cds

AAATAATATCTCGACAAAAAATACTAGTTTATATCCGACTAGCTAAAGATCACTAGTCTTCACAGTAACGTCTTAAAACAAAGTCCTGCCTTTGTCTACGCTTCGCTACACCACCGTGACTGATCAAGACAAATGATCATCACCAATTTTGGGTTTCCACGAGTTTAAAGCGTACTTCCCACTAATCGAAGGTCAGCTCTTCGGAAGTGTAGTTGTTCACACGATTTAGACCAGATCATGCGTGGTTAGGATTAGTATAAAATAGTATAGTGAACGAAAATAGAACCAGTATCTATTATTCGCTCGTAGAAGTGTAAAAGTGGTTTACAATGTTTAAGTTGATTTTAAGTTGTATCGCTGTGGCAGTTTGCATGAAGAGTGTTAATTCTTTGACACCAGATCAGAAGGCAGCAGTCCAGGCGAAGCTGCTGACCAGCGGGCTCCACTGCATCAGGGACCACCCCCTCAACCTGGATGAGATAAAGATGCTGAGAGACAAGAAACTCCCAGAAGGGGAGAACGCGAAGTGCTTTACGGCTTGTTTGTTTAAGCAGATTGGTATTATGGATGACATGGGTAAACTCAACGCAGCGAATGCCGTAAAAAGTGCCGAAGAAGTTTTCAAAAGCAGCGACAAACATTTGGAGAAATCCAAACAAATAATACAAGAGTGCATTTCTGTGAACGACGCTCCAACCTCAGACGGTGCCAAAGGATGTGACCGTGCAAAATTAGCGTTTAGCTGTTTAATCGAAGGCGCCGATAAACATGGTCTCCACATCACCTTCTGAAAGACGCTGAAAATCACCTTTGTTTAAATGAATAAGATATTTTATGTAAGGGTATTGTGTTCTCATGACAAAGCTACAAAATAAAAACAACCATTTCGATGTATTATAGTTAGACTAGTTATTTAAGGTAAATAACAATAAGTATTATTATGGCATACGGAAACTTTAATAGTTTATTAAAAATAAAGTTTGTTAATTAATGTATATTGCTTTAA

>OBP20 [Moltype=mRNA] [Organism=Loxostege sticticalis], complete cds

CGACGGGTCTGATAGTCAACATGCGGTTTCTGTGCCTTTGTTTGTTATTGCAATCGGTGTTATACTCTGAAGCAACTTTTGGAACACCCTATTTGGTCAACTCTCGCTTATGTCAAAACTGGACCTGTGTTAATTCCAAACTTGGACTTCCAAATTCTTTGCCACCTCGTGACCAGTACACCCAGATTTTGAAGACATTGCTGCCTTCGGGGGCATGGCAGGATGTGGTTGAACGAGTTCTAGATTCTTGCTATGGTACTCGGCCTAGAAATTATGTAGGCACTTGCCCAGGACAGGCATTGCTTCTCTGCACAGTAGATAATTTGATAGAGAACTGTCCAGAAGAAAGTTGGAGAAAAGATGATGGCTGCTACCCAGTTACATCTTTGGCTGGCACAAAGAACATGTTCACCCAGAGTCGGTATCAGAACCTCGAGAAGAATTTACCAACCGAACGTCGACCAGCCTGGTTTATGAGAAATTATTTCAACTCGAAGTGCTGCAACGTACCTCAACTAGTAAATTCGACAGTTCTATCAGAGTGTGGCTTTAATCATTTTATGAACTATTTCGAACATAAGCCAAGGACAGAAAATATTGGTGAAAAAACGCATTTTATGAAGCGAGTTACTTCTGCGCCGCCAAAACACAACGCCAGGATTTTTGATTTCAATAGTATTCCAGTAACCGAGAAAGGTCCAGATGGATTTGGAGATAAATTACAAAATGCTGATCTCTATCCAGAGAACAACAATCTCGACGCCCTCTCATGCTGTGATATGACAGAGTTCATCGCCCCATCCTGGCAGTCGGAATGCGGCTTTGCACTCAACTGGAACAAACAAACCCGACTGACGGTCGCCGAGAACTTGGCCCCGACCACTACCGTGCCCCCCACGACCACCGCCGCTCCGCTGAACAAGGATGTCAGGATCGTGCCATTGTCTTGCGAGAAAGAAACATGCGTCTTCCAAAAGCTGAACATCGTATCGGAATCAGGTTCCGTAGACATGGAAGCTTATTCGAAGCTTCTAGACAACTTCACCAGCCTCCACCCCTCGTGGGCCAGCGCCAGGGCCAGGGTCATCACGATGTGCATCAGCAGGCCCAACCACGGGTACCAAGCTGATTGTGAGATCAACAAACTGCTTGCTTGCACTTTGGATATACTTTCTGAAAACTGTCCCTATGCAAACAAAAATGATTCGTGCAGGCATACGAGACAAGACAATACCATCTGCCAGATCAGTACATCTAGATATAGACCTAAAAACCGTCGAGCCGTCTGCGGACTACCGAATCTAGTGAGCAGCGAAGTGTTGTCGGAGTGCGGGCTCACGTCAGTCAGCCGCACGGAGTACGTGCCCGAGCACAAGCCGAAACTGCTCCAAGGGTGGAGGAACTCTAAGTATGCGTGCAAAGACTCCACACCGCCCACCGCCTGCATAATGACAAAGATGGGTGTCTTAAACAAGTACGGCTTCATGGACTACTTCAGGATGAAGGACCGGATCAGATCCTTCACTACGAACCATCCGGAGTGGTCGGCATTGGTAGACGTATACATTAACGCGTTCAGCGGCATGCCGATGTACAGGGAATACTGCAACTCACCGAAGAAACTGCTCAATGTTGTCGATGCTATGCTGATGACTTGTCCCATCACAAAAAGAAGAAACACACCACAATGCACAGAACTCTTTGCCAACATGACAAACACAGCACCAGTCAACAACAATCAAAACGTGACCAAAGAGAAACTAAATGAAATCCTTCAACATTTCAACCACATGTTCCTACCCTCGTCTAAACTAGCGAAAAAGACGGTATACAAACGAACGAACCCGCTTTTTGATTTTGGAATGTTCGATTCTACCAACGTTCCGCCAGTCGAAACGTTAGATTTAAAGACAACGAAAAAAATACCAGTTATTTTACCAGTGTACATGAGATCGAATCCGTTACTCCGAGCTAATACTGCATCTACAGGGCCTTATAAAACTGGCGTTTTCCAAACTTCGTCGTTTAAGCTACATGGCAAAATACCTTCGACGCCTTTGCCTTTTCCTAATATGCCTTAATTTTAAAAAAAGTTCCTTTGAAGACTAGAGTCCGATCAAAGATAGATGCAAAGGTGTCCAATGCAACGAATAAATAAGTAAAAATAAATTTCGACAGTGTACTTTCTATAAATGAGGCACATACATAAGATCTTATTTTGTATTTCTTTTTAATTTTATGTAGGTAGTGCGAGTTTTTTTACGTGTGCACGACCGAAACGTGCCATGCTACAGGCCGCGTCATCTAAAAAAAAACGCTGGAAGAAAGAGAG

>OBP21 [Moltype=mRNA] [Organism=Loxostege sticticalis], complete cds

GTGCAAAAGAGAACAGTTAACTACTGGTCTTTGGTCGTTCTTAGCAGAAGTAAAAACATTAGCAAAATGTTCAAATCTGGTGTCTACATTGCTTTGTTTGCTTGCCTTTTGGAAATGGCTATGTCCTTAACAGAAGAGCATAAATTAAAGCTTCAAGCAACGTTTGAGACGGTTGGCGAGAAGTGTGCGAAAGAGAACAATATAACTGAAGAGGACATCGCAGCCTTCAAGGAGAGGAAGTTCCCTGACGGTCAAGAGGCAGCCTGCTTCAGTGCCTGCGTGCTGAAGAATATTGGACTTATAGACGACGAAGGGCAGTTGTCACATGATTTAGCAGTAGAAAACGCCAAAGCAGTGTTTGGTGAAGGAGACGAAATCAAAGCCATCGAAGAGTTCATTGAAACTTGCAAAGATTCAGTTGCTGATGGAGCTGACGCCTGCGAACGGGCAAAACTTGTCTTCAAGTGCTTCGTGGAACATTCAGAAAAGTTCAACTTCTAAATAGAGAAAAAAGACGCTGATTGAGGTATTTAAGGAAATTGTTATAACCTTTGTATTTTACTTATCTGGCATCATAAATAGGATTGTGGACATTAAATATAAGGCAGTTAAACATAATGTTAAGTAACTAATAAATAAGGTG

>OBP22 [Moltype=mRNA] [Organism=Loxostege sticticalis], complete cds

TTGGTTAATCAGTATTTGAAGTGACAAGTTGGTTTGGTTTAACTAAGCGGAGTGAAAATGCGGAAATTCTTTTGGATAATTATTGTTTTTGTATCTGGGGTCAAAACTGATCTTTTACATCAAGATCGAAGCAAGGGTGCTACGCTGAAACCTATATCTGCATGCTGTGATATCCCAGAACTGGGAGATGAAAAGCCACTTTCAGAATGTTCGAATCCAAAACTACCCGGTCCATGCAACGACGTACACTGCGTATTTGAAAAGTCAGGTTTCTTAGTAGATAAAAATACTCTTAACAAGGATGCCTACCGGCGCCACCTGCGGCAGTGGGCGGAGAATCACAAGGACTGGTCAGATGCCATAGAAAGGGCCATCACAGACTGTGTGGATAAAGACCTAAGGCAGTATTTGGACTACCCTTGTAGGGCGTATGACGTCTTCACTTGTACTGGGATTGCTATGTTAAAGAAATGCCCAAAAGATGCATGGAAATGCTGAACACTCAAGACTGATGGCCTCTAAGAAATATACTATATTAATTATATATACAAGCGCC

>OBP23 [Moltype=mRNA] [Organism=Loxostege sticticalis], complete cds

TTCCATTACTGTTTTATCTTGATCAGGAACTTGGTTCAGTGCGTTGTCGAGATAAAACAGTAATGGAAGACTTCATAGACAATTGTTTGCCAAAGAAAGCTAGCGCCCCCCATGAAATTGCTTGGAACTACACAAAATGCTACCACACACAGGCTAAAGAGCCTCATAATAAGAAGAAGAAACTAGATTACCTTAATATATTTTATTAAAGCTAGTTTCATAATAATATAAAATTCTACTTTGAGATGTCAAATTCTACATAAAATTTTATTATTCACTTACAACAGCGTCAGCGCCATCTGTTATTTTTTAGTTGAACGATTGTACCACTCTCTGGGCTCTGCTATCAGAACTGTTTTCACACAATAGTTCACTTCTGAGATGATAGATGGCGCAAAATAGCATTGGGGCTTACACAACTGTAGAAATTACCTTGTTAAGCAGGAAGTCCCAAATTTTTTTTTGTTATTTTAAATAAAATAAAATGTATGTACT

>OBP24 [Moltype=mRNA] [Organism=Loxostege sticticalis], complete cds

GTTTGTAAGCAATGTACAAGGAGAAGACGGGAAAAATCATTGGAATATTAAAAACTGGACGTATACATATGATCTCAAAGGCAAATCCAATGTTTATTTTGAAAACTTGTTTAATAAAGAAAGTTTTTTGGGTCAAACTGCCCAGGAAATGGTAGCATCCAACGGCAACGCAATTATCCACGATATTGGCAAACCAATCATAACATCAATAGTTACTGAAATCGTACACAATGTCCAAAGATTCTTCAAAGCAGTGCCATCTGAAGACTTGAGCCTTGATTAATCTATAATAAATATAAATGTAACCAAATGTATTACGAAGC

>OBP25 [Moltype=mRNA] [Organism=Loxostege sticticalis], complete cds

CTTGCGTATAATATCATCACATGCTTTAACGAATGCGTTCTCTATACAGTACCATTCATAAAGAAATGTGAACCGTCCGACTCAAAATGCGTACTGTTCAACACTGTCAACACAATTCCCCTGTTTGGTGCTGGGATACCTGAACTGGATGTCGAACCGTTAGACCCACTATACATAGGCAAAGTTGATGCCAGCACACCCAACCTGAAACTTGTAGTCGACGATTTGAAAGTAGAAGGATTAAAAGATTGCCAGGTGCTCAGTATCGAACATGACAAGGCAAACCTAAAAATATATCTGAATATTGAGTGTACGGTCGATCTCTACGGAAAATATGATATGGATGGCCAACTTCTCGTTCTGAAACTCCAAGGAAATGGCAATGCACACTTCAAGCTTAGAAACACACAATTTAACGTAATATGTGAACACAATGAGAAAATTGGCAAAGATGGAAAGTTACATTATAATATCAAGAACACTAAATACACATACGACTTGAAAGGAAAAGTGGATATAGAGTTAGAGAACCTTTTGCAAGGCAATGAAGTTCTGGCTGCTGCAGCTCGTGAGGTCTTTACCACAAACGCCAATATTATAGCTGATGAAATTGCACCGAAATTCATCAAGGCTGTCGTCGACAAGATTGTCAAAAACGTTAACAACTTCTTCCACGCCGCCGCCGTTGAAGACATCGAAATTGTTTAGATGCTGTTGAAACAAATGACACATGAATTAAAAAA

>OBP26 [Moltype=mRNA] [Organism=Loxostege sticticalis], complete cds

CGGAAAAATAGTATAAATATGTAGCGGTTTTGGTAAATATTCACATCAAAAACATATATTGAAGAAATAACATCTAATATGATCCAAATAGTCTTTCTAGTTCTAGCTTTTATAAGCGGGTACAGCCATGCATTGACCGAAGAAGAAATCAAGGCGGAATTCACCAAGCTGGTGATGAAATGCCTAAAGGACCACCCAGTGGACATGTCTGAGCTGACCAACCTTCAGAAGTTGGTGGTGCCCAAGAAAAATGATGTCAAATGTCTGCTTGCCTGCGCTTATAAGCTGGACGGAATAATGAACGCCAAAGGCCTTTACGACCTCGACCACGCTTACAAAGTGGCGGAGCTCACGAAGAACGGAGATGAGAAGAGACTGGAGAATGGGAGGAAGATGGCTGACGAATGTGTCAAAATTAACGACATTGAGGTGAGCGATGGTGAAAAAGGCTGTGAAAGAGCAGGACTGATGTTCAAATGCGCCATCGAAAATGCACCTAAGTTTGGTTTCAAGCTTTAACATGAAGACGGAGAAGATGATAAATCGAGAAACACCCAAGAATACTACCTAATTTATCTGTAATGCT

>OBP27 [Moltype=mRNA] [Organism=Loxostege sticticalis], complete cds

GGACAGACAGTTTCACTATCTAGCACTGGGCTCGCTTCCAGCTAAAAAAAATAGTAACTCCCAACAAAAAAGCTTCAATCCTGCTCATCATGCTGCTACCATTTGTCACCCACGCTCGTATCTCAGTGATGTACGCTCACGACAAACTGAGCGACATCGTTGCGGAGCAGTGCTTCAACGAAATGTTCCCAAAGACAAAGCACGTCGAGGTTCAGGAGTCGGACGAGCCGTGCCTCATCTTCTGTGTCATGAAGAAGCTGGGGATCATGTCTCCGAATGGGGCTATCAATTTGGAGACGTATAGAAAACGAGTCCTAATGGCCCATCAGCACGACCAGAGAACCCTGGTGAGTGACTTCGGGAGTTCATGCGTGGAGAACGCAGAGGCCACGCAACATAAGCAGGACGTCTGCAAGAAGGCAAAGGTCTTCAATGACTGA

>OBP28 [Moltype=mRNA] [Organism=Loxostege sticticalis], complete cds

AGCTGTGGCACTGCTGGTCTTCGTGTACGCGCTGTATCGGTTGCCGTTGGCTCACGTGCTGCTTTACCTGCCGCTGCCGCTGCGCCTGCCACAGGGAGTGATCATGACGTGGGTACTAGCTGTGGCACTGCTGGCTGTCTTTGGGGCAGTACAATCGGCTTCAACAGGATGCAAGAACTGCATTTCCCTCGGCAAGGAGGAGAAGGCGATGTTCCGTGCGCACTCCGACGCGTGCCTGCCGCAGTCAGAAGTGGATCCGAAGTTGGTGGAGGCGATGCTGAACGGGGAACTGACCGACGACCCGGCGCTGAAGCGGCACGTGTACTGCGTGCTGCTCAAGTGCAAGGTCATCAGCAAGGACGGCAAGCTGCAGAAGACGGCCGTGCTGGGCAAGATGGCCAACAGGGCTGATGGGAAGAACGCTACCAAGGTGTTAGAGGGCTGCGCAGAGCAACACGGAGACACCCCCGAGGAGATCGCATGGAACCTCTTCAGATGCGGCTACGACAAGAAAGCAGTGCTGTTCGAGTACATGCCCACCAACATAGGCAATAGCGAGATAGACAACAATTCCTAATGAAGTATTCAACAAGTGTTGCTCATCGATATCGATGAATGCAATAGTATCGATAGTACATAACACTACCGATACTATTGCTATTGATTACCGATACATTAGCAACACTATTCTCATTATTGCAAAGTATTACGTAGTTCCATATCCCGCCAACAGATGCCTCTGATTAATTATTGAATTTTCCAGTTAGTCCGTACAGATGCCGTTAGTTTCCATAAAAAGTCAATCAAAACTGTCCAGTGAATTATTTAATAATATCCATTGTTCTGGATTTTGTCCGTCAAGTTACCATTTTATAACATCTATTTATTACTGTATCAATATAAATTTAATAAATATTTACCCT

>OBP29 [Moltype=mRNA] [Organism=Loxostege sticticalis], complete cds

AAGGCAGACTTCATCAAGCTGGTGGGCAAACCAAAAAAAAGTTTTCACAAATATAGTTGCGACATGATAAAATTATTATTCATAGTTTTATTGTCAATAAGTGGATCCAGTCATGCAATGACCGAGGCAGAGATCAAGGCAGACTTCATCAAGCTGGTAATGAAATGCCTGAAGGACCACCCGGTAGAAATGACGGAGCTGATCAAACTTCAGAGCCTGGAGGTTCCGAAGAAACCTGAAGTCAAATGCCTGCTGGCCTGCGCTTACAAACTGGACGGATTAATGACTGAAAAAGGTCTCTACAATATAGAGCATGCTTACAAAGTAGCTGAGGTTACTAAAAATGGAGATGAGAAGAGACTAGAGAACGGAAAGAAGATTGCTGACATATGTGTTAAAGTGAACGAGAACGAGGTCAGTGATGGCGAAAAGGGATGTGAGAGGGCAGGAATGGTATTCAAATGCGTTGTAGAAAATGCGCCGAAGTTTGGATTCAAGATCTAGAAAACAAAGAGCCTGTAGACAAGCAAAAGTAACTGAATAATGTATAAATAAATAGAAGTAATGTTCCACAAACACGTAGATACATAAGAAGATTTGAAATAAAATAAATGAAGGATTGGTGAAAATTCTTACTTTTTTGATTTTAATTCAAAATAAAAGAGGAAATAAATAGTTCCAACTTTTTAGAAACCAAATCATGCTCAGCCCGTAGGTACTAAACCCCTGAAGAGTGACATCTGGCGGTAGCTCAATGAATCAAACGATGGGGTGATGTTCTATCCACAGAGTACTTTGGTTCTACCTTGCTAACGGACCGCTTCACGTAATGTTAACTCCATATTAAAACAGATGGCTAGTCACCTTTGGCAAACGATGAG

>OBP30 [Moltype=mRNA] [Organism=Loxostege sticticalis], partial cds

TTTTGTTTTGCCTTTTTGCTTCGATTTGCGCTGTGATTGGTGACCATGAGAAAGACAGTCCAATAATGGCAATGGTCCACAAAACGCTGGTGATCACAGCCCACTCCTGCATGGATCAGATCAACGCAACTGAAACGGATTTGGAGTACTTGCGACATGACCCTCCCTACCCTGAGAAGGCGTCTTGCATCATCAAATGTTTGCTCGAGAAGG

>CSP1 [Moltype=mRNA] [Organism=Loxostege sticticalis], complete cds

TCGGCCGTAGTGCGTAAGCGCTCTTAAGTGATCAAAACTATCGAGTGACAACATCATTATTTCCTCCATACAATAAGACAATGGGGCTGACAATCATTGTAATTAAATTATCCGAAGGCTTCTGTGTGAGAACACGTTTCGAGAAGCATCTCATTCAGTTATTAACGATGTCTTTTTTGTGTATGACTTAGAAATATTTAATTGGCATCTGAGTTTGCTGACTATTATTAGTTGTTCTGTGCTTGTGGTGACAGCTTCAGTGCCATTTCAGGATCAAGGTAATCGAAGTTAACACAACTTTTCCGTAGTGTAATTATTTAGGTACCAACTAATTGCGTTTGTGTGTCTTTATAATTAACGTACTTCGTTTCCTCGAAAGTTACTTTATTTTGTTTATGATAACTCATATTTGAAATCTTGCTAATTAGCTAATTAGAAGGTCATATCTTCGAAATAGGACAAAATACAACCTTACTGGCAGATTTTGTAGGTGTGGTAATTGCAATCACATTTATTTTGAAAAAGAAGTTCGAGCTTTTATTTATCAATGAATATTGCCTAAATCTCAGTAGGCAGATAAAGATTACATACCTAGTGTTCTTATATCATTAAGGTTCATGGGTGACGTTGGAACGGTTATCTGAGCATCTCCACTGATTGTTGGTTGGGTTACAGGAAAACATTTACATAAGACGTAGGTAGTCATTTTTTAATTATGATCATCACTTTGAGGTATTTTCCACGCGTTCTTACCGTACGCGATCTTTGGTAGGTTACTTATAAATTGACTACTATAATAATTGTCTTGGTTTTTATCTACCCAAATGAGCACCCAGGCCAAGGTATCAATTCACAACCCCAAGTAAGTATTGAGTTTAGAGCAGTTTAATGGCTTATATTTTAAATTTGAGTCCACATTATAACTGACAAAACTACAACTTACAGCTACTCTATTGGAATTTTGATAGCAAAATTATGAAACAAATCGAAGCATACCGCTAACAGCAGCTTGGTATCAATGGGGAGGCTAATGTATGACAACAGCAAACGTCTATGATGTAAAAAGAAAATCGTACATGGTGTAGTACCACATGTAATAAGAAATATTAATCCCTTTAGACGGACAGACAACATTAATGTTTAAGTGCTTATATATAAGTAACATCTCATAAAAAACAACAAATAATTAACTTTATAATTTCTCCATTCCAGATCCACAATGAAGTCACTCGTCCTAGTAGCTTTATCTCTCCTGGTGGCGGTTGCCTGGGCCCGTCCCGGCGCCACCTACACTGACAAGTGGGACCACATCAACGTGGACGAGATCCTGGAATCCCAGAGACTCCTCCGAGGATACGTTGACTGCCTCCTCGACAAAGGCCGCTGCACCCCCGACGGAAAAGCCCTGAAGGAAACTCTTCCTGACGCCCTAGAACATGACTGCTCAAAATGCACGGCGAAACAAAAGGAGTCTTCCGAAAAAGTTATCAGGCACCTCATCAACAAACAACCAGATTTCTGGAAGGAGCTTTCCACCAAATACGACCCCGAAAACATCTACCAAGAGAAATACAAGGACAAAATCGAAGAAGTCAAGAGCAAGAACTAATTTATGAGTAGACACTCACTTCTGCGTAAAATGCACTTATACTAAATTGTGATACGGTGGAAAGATGATGTATGGAGACTGTGATTGGTTGTATTTATTAAAATAATATTTTGGAATCAACGAATTTATTTAATTTCCAACTTTATAGTATTAAAAATATGATCACTTAGTTAGGTTCCTACACACATACCTACAACAAAGAAACTAATTAATGAACATACGCTACTAATTATGCTAACAAAAACAAAAGATTGTTCTTTGTGCAAAGGCTATTTTTGAATCAAAGTGTTTACTGTTTATTATTAGGTAGGAATTTCTAATTTTATACCATCATAAATAATAAATAATCTCAGGTACATAAATTTATAATTGTAGAATGGAACATTTTTTGAAGAATCCTCAATATTATTTCTTTTACTGACAGTTAACTTTGACGTAGAATAGTATTTATTGCCGGAAAATTGTTTAGGTAAAATGTTTAATTACATACCACATGCGATCCTATGTCAATTAATGCCAACTTAATGTATTTTTATTAAGAGAAAAAACTAACAGGAAAAATCTCATTTAAAAATATCTTAGGAAAAAAACATGGTTAATCCATGTGGGTTTGACCTTTATTGGGTGAACCAAATTATTACAGTATCGCTATCAATAGGAACATGGAACGTCAGATCATGCTCTATTGTTATGTAATATACAAGATGTTACAAAAAAGTGCGTAATCCTTTAAGGGGGCGATTTTACTGCTCAAATGCAACAACTTCTTTTATGGGACCAACATCGAAATCTCA

>CSP2 [Moltype=mRNA] [Organism=Loxostege sticticalis], complete cds

GACTCGCACTTGGCCGGTTTTTTAGTACCATAAGGGACGGGTTGCGTATCCTTTGATTTTCTTGTCACTTGTGCCATTAATTATCTTGTAGGGTAATCAAAAACAATCTTAGTGGACCAAAACACTTGGCATGTGCCTTGCTTTTAGCATTTACTGTCACTTTTCCTCAAGTGGTTTACAACTTAGGTAATAAAGCTACTTTCGAATTTATTCGAAACGTATCCGCATACAACTTTTGTGTTTTGGGAAAACCACACAATTTTTATTTGTTCTTTAAAAATAAAAACTTAAATCGACAACTTTACGGAAGGACCTAAGGAGCGGCATTAAAATTTTCCAGTAGAGAGTGTCTCGGTCGTAAGAATATGAAAAAAAAGGTCTATTCAGAAGGTCAGGATTGTTGGTATACTGGAATGTAGACCCACAAAATATGAATTGTTTTCCTGGAAAGATTATGCCTATGCTTAATCAATAAGTACGAAGTCATATTATAAGTATTTATTAAAACTACATTTTCTCATATGGTCATTATAAACGTTCCAGCAAACCTACCTGACGCCATCCAGAACGACTGCAAGAAGTGCAGCGACAGACAACGCGAAGGTGCTGACCAAGTGATGGAGTACATCATTGATCACAGACCAGACGATTGGGTGAAACTGGAAAAGATGTACAATTCGGATGGCAGTTACAAGAAAAAATACCTAGATAGGAAAGAAGCAAAAAACAAATCAGCTACCACTTCTGGTGAAAAATCTGAAGAAAATGTAAGCAAATCTAAAGAAAGCCAAGATTAAGTACTAAGTAGACTTCCTTAAAAAAATCCTCTCAGACCTGTGCTACTAATATACCGGAATCCGATAAAAAAAACGAAACAGTCGCTAATAAAACAGTGGGTTAAAGTTATTGATTTTTTTTTTGTAATATCTATTTTAAATTATTATCTCATTCTAGAAGGAACGAGTTTGGCTTTTTCATGTAAAATATAGAGTTTGTCAGCGTTTTATCATGAGAATCTTCCAAAAAGATTTTAAAGGAGTCTGACCAGAAAGATTGCAGTAAGATACGTGTCTTCAACGCCTGTGAGTCAGTGTCAGATGTCTGAGGAAGACGCGAAGGTGATCATTTGCAGTGCCGTATTAAGAGGAATATATACCCTGGAAAAAAAACATACAAGAAAAATTTGTCATTTTCCGGTGTAATTTTCTATGAAAGTTGGCATGCTAATAGCTTCGAAAAAATATATGTTGTTTATGTATCTTTTAAAAAGAGGTATTAGAAGTTTCGGAACATAATAACAATTAGTAACTTATAAAAAAAAATAAACACTAAAGATTAATTTTTAAGTATTTTTAGCTCCTATCTCAGAAGTTATGTGAGTATAACCTTCATTTTTTTTACTAAAATAGGTATTTGTTATTTGAATGGTTATTAGTAATATCAAATGTTTTAGGATACAAATACACTATATACGAATTTTTTACTCTATATCCAGCGATTACGCTAGATTGTTTAGTCTGCACCGCTGGTTGGTGCTTTTTTTTAGCGTACGTAATCCCCTTAAGGCGACTTTATAAAGAGCGTTTATTGTCAGAGGTGGACCCAGACGATTTAAGGGTAAGCCCTGGCGCCGTTAGTGTTGGAAGGCTTTACG

>CSP3 [Moltype=mRNA] [Organism=Loxostege sticticalis], complete cds

CGGCGACCGAGCACCATCAAACGTGAGTATCTGCTTACAGGATATTAAAGAAGATTCTATCTTATAATCATAACTTAATTAGGTTGGGTTAACTGCCGATCTAAAAAAGTTAAATTTAATTTGCAAACTAATTAATGTAGTGTATATACGTTATTTTGGCAGTAGCAATTTATTTTGAAGGTGATGTTTTTTTTGCAACTTGACGAGGCTTGTTGTCATTACTAGCTTTTATAGCAGTTAGTATTGACACTAGTTTCATGTCGTAAATTAAGTTAGTTAATAACTCCTTTACTAGTATCTATAGAGAATTCAATGAAGGTCTCTGCTCTTAAGGTACTGAGTGGTAACCGCCATGTCCTAAAACAAGCATATCTGTCAACCCCCCAGTGATAGGGGATAGCCGCCCTCTCTTAACAATATATTTTCTCATTTAAACCTTGTTTTAATTGAGCTCCTCCACAGGCCCTTAAGATCAGACATAAAACGTTTGATTCTAAAGGTTAAAAAGACTTGGATCCATTCTCTAAATCAGTAACAGATTGGCAGAGCTACAAAGCAAAGAAACATAGAACCACTATCCAAATTACCTATAGTATAACCAACCTGTCGAGTAGAATCCGTTTGCAGTCAATAAAGTTTATACATTTTGTGTAACGTTTCGTTCACGAACAGTGTTCTCTAGACACAATTTGTGTTATATTGTTAATACGCCTTCATCCGTACACTGTCTAAAATGTAAAAATCTCACAACAATACAAAACTTTATATCTTCCTAAGCTATAATCTTGGTATCTTACGAACTAAACATCATTTAACCTATTTAACTAACAAACCATTGCTTTTTTCCAGAAAATGAAAACCATCGTAGCACTGTGCGCACTTATGGCGGTAGCGCTGGCCCGCCCTGAGGAGACCTACAGCGACACGTGGGACAACTTCAACGCGCAGGAGTTGGTCGACAACGTCCGCCTGCTGAAGAACTACGGCAAGTGCTTCCTCGACCAGGGCCCTTGCACGTCTGAGGGTCAGGACTTCAAGAAGAGGATCCCTGAAGCCCTCAAGACCGACTGTGGCAAGTGCTCCCCGAAACAACGCGAGCTGATCAGGACTGTGGTCAAGGGCTTCCAGGCCAAGCTGCCTGAGGTCTGGGCTGAGCTGGTCAAGAAGCACGACCCTGAGGGCACATACAAGGACTCCTTCGAAGCCTTCCTCAACTCCAACAACTAAACCAAAGCTATGATTAGGTGGCGGTTCAGCTAAAAGCGATTCTCTATAACATCTAGAAGAAACTGAGGATATTATCTTTCCTGTGTGATACTATATTTAAGAACACGGCAATAACAACGTTATAAGGCTACCGTGAAGCACCACTATGTAGATGACATCAAAGTGATAGAAGAGAAATCGCATTTTTCTGAACTAGCCCTTTTCGATTTAATAATTTTCGACAATGCAGCAAAATGTACACGTCCTTTTCAATATGGTTGTGATAGTAATATAACTTGTTTACAGTGGAGTTTCAAAGGAAAATGTATGTTATACTTCTTTTTCTTTTGAATACTTTCAGTGAAAAGAAACTAAAATTAGAAAACATACAAGATCTTAAGTATTATTTAGTTAAAAACTATGTACTTGCTCAAAATCATCACATTTTATAAATTATTGATATAATTAAGGTTTTTTTATCTTATTTAAAATAAAGCCGTTTTTAAAAATACACTATTTTATTTTCAACTAGATATATGTTCATT

>CSP4 [Moltype=mRNA] [Organism=Loxostege sticticalis], complete cds

GCAATCAAAGTGTTTGTTAATAATGAATAGAGACTGCAGAGGCCCCAAAATTCTTTAGCTGTTTCTATCTAATTTCGTGAGGAGACATAGCCCCAAGCCCCGTAGAGACTAAAGCCCCAGGATTCTTCAGCTGTACCTAATTTCGTGAGGAGATATATAGCCCTAAGCTCTGACCAATGTCAATACTGAGCTATGTCAGCATTCTTGCTTTCATTTCTAATCTTTAACAGACAAAAATCATGAAGACTTTCATCCTGATCTGCCTCTCCGCACTGGTGGTGGCTGCGGCCGCCGACAAGTTCGATGACCTGATGAATGTGGATTTCGAAAAACTGCTGGGTGACGAAGAAAGCAGGAAGCAACTGGTGGGCTGTCTCATGGATGAACTTCCGTGCGGGGAATACCAGTCGTACAGAGATTTGATTCCCGATCTCATAGCCACGAACTGCGGGAAATGCACCCCAGAACAGAAGAAGAGGCACGAAGAAGTAAATAAGTTCATCCTGGAGAAATACCCCACTGAGTACAACGCAGTTGTTAACAAGTACAGACCTAAGGCTGAATAAGAAATAAATGTACGAAATAAATAATCAGTTTATGCATTATGTTATAGAAGTCACTTTTATTTAATTGATTTATTAAAACGTTTTATTAAATTATTGCTATTGTATATATTTGTATATGAAACACTATAAACACTGTCATACCGTTAAAACGAACAATACAAATACGAAAGCAAATGAGGTGTTTTCATCATGAAAACGAATATAAATTGATATATCTGTCTCATCTATTTTCTGACTGGTAGAGAATACCTTGAAGCATTAATTCTGCAATTGTATTGTACATTATTGTAGTGCAATGAAGAATAAATAAATTAAGAATGTCCATTCCCTAGAATCTGACTCCACTCGGATTTGTCGATTTCAGAATGACGAAAAAAATATAAAGTACTTCGTTTAAAGAAATTATATTGATATCTTATTATTTGCCATTTCTCAATTTGTTACTTTTAAGAAAAATATTTTACTTTTTGTGAGTAGTTTTCCCATTATAAACATTTTCCCACAGACAGTTTTTGAAATTAATGGTCGAAGGTCTACGCAGACGCCAGCTATTAGCAAAATCATTAGAGCCTTGACTGTTTTGACATCATATTTTAAATACCTGCTATATTTATTAGTTCTTTTAAGAAAAGAATACACAAATCAACTTAGTTTTCATTCAAAAACAAACCACTCAAAAAAGCATAGATTTGAAGTTTTTCGTAATAGTTGCGCGTGAGTTTTGTATTGAAATCGGAGGATTTTTTTGTTTTTTTTGTGTCTAATCTACGAAGCCTTTCATATTCTTAAATATGATTTCTCACTTTTGTAAAAAAATGACTATTTATTTTACACCGAATTATTTAAAACTGTTATATAAACGCTTCCCAATGAGCTCACGATTCAGACTCGGCTGCGGCCTTCTAGAGGTGAGACGTGTGTCGCTCGTCGCTCCGTGCCCCCCGCGCTCCGTATCGCCCCGCGCGCAAGTTTATAGCGCGACTAAAACGAGTGGATACTTTGCGTTTTGTTTATTAAGTATTTTTATGCAGTTAATATGATTAAATATACGGCAAATGAAATAAACTCCTTAGTTTATATAAGCAAAACATATTGTTTCTTTTTTCAGATATAAGGCCATGCTTGACTTTACCAGCGTGCGGTGTAGAAAAATGTGATTCTCACCTCAGTGCGGGGGGGTATTTTATGTGTTACTGCATGAGTACGAAAGAAACAAACAGATATTTCCGAATGTATTTTTAAATATGTTCTAAATTCATTGAAAACAGACATGGCAAAATAATGTTCACGACGCACAGAGTTATTTACTTTGTGTTGTGTGCGGAATGTCCAATATATGGTCACGCTCCCGCGCCTCATGCCGCACCGAAGCGGGCGCGTCATTCTCGCCAGACGCACTTAGTGATGTATAACGTCAGCACGTGTCATGTTTAAATTTTTTGACCACCAGCACGTTTCTTACGTAAGTTAAAGTTCCATAATAATTGTGCTTTAAGATACAAGTTACATTTTTATAATGTTATTATTATATAATTTATTCCTTAGATTTAATCTAAAAGTACAAAAATTAAACCAAGTAAAGGTTAAGTAAAGAAAATCCCACCAAAAACATTCTTTAAA

>CSP5 [Moltype=mRNA] [Organism=Loxostege sticticalis], complete cds

CTCGGATATAGACCATTCAGTTTTACGTGATCTTTCAGAGCAAATACATCGAGAAAAACTTAAGGATGCGTCGCTTCATATTACTGTCAGTTTTGGCCCTGGTGGCCTTGTCACTGGCAGAAGAGGAAAAGGAAAAGAAAGAAGAAAAAGCCGAAGAAAAGTATACTGATCGATTCGATGACATCAATTTCGAAGAAATCCTGGCAAACAGGCGACTGCTGGTGCCATACCTCAAGTGTGTGCTGGATAAGGGAAGGTGCACTCCTGAGGGGAAGGAGTTAAAAGTTCACATTCAAGACGCGATGCAGACTGCGTGCGCGAAATGCACCGACAAACAAAAGGTCGGTGCAAGGAGGGTCGTCAACCACATCCGTGAGAAGGAGCAGGAATACTGGGAAGAGTTGCTGAGCAAGTACGACCCTAAGGGCGAATACAAAAGCATCTACGAGCCTTTCCTTGCTGGTAAAGAATAAGGATTGTCAAAAGAGGATACTTAAAAACTCCGGAAATCAATGTTGTGTGGTTTGATGATTGAAATTAGTTTATGGAATTCTTTGTAATACTTTTCTACGATTTTATGTGATTGCTCATTCTTTGTCTTTTTAGTTGTACTTGTATTAAAAAATGTTTGTGTAAATATTTTTCTTCTCTTAAATAAAATTTGAAAAACAGAAAAAA

>CSP6 [Moltype=mRNA] [Organism=Loxostege sticticalis], complete cds

GTCAGGGGGGGGTTAATGAGCGTTTTAAACAGAGGATAAACAAACTGCACGTCATTTCGTATCAGTCTATCAGAAGGTTAATTTCGCTATGCCGAGCGCAAAATGATTGATTCACTCAATTTAAAAGTTGTTGTGTTTTTGCAAATTTGTTTTTTGATATCGTCCGGTTTGTGTCAGGGAACGTACGATCGGAAGTACGATTATTTTGATATCGATACTCTAGTGCAGAATCCTCGGCTGCTGCAGAAATACATGGACTGTTTTCTTGATAAAGGACCTTGTACGCCCATAGGAAGGGTCTTCAAGATTGCCCTTCCTGAAGTGGTAGCCACAGGCTGCAGCAAATGCACTCCGGCGCAACGCAGCTTCGCTAGACGCACGTTCGGCGCTTTCAAACGCTTATTGCCAGCACAACACGAGGAGCTTAAGAAGCTTCTAGACCCTAACAACAAATACTACGACACCTTCGAAAAAGCCATCGCTAATGCATGAACTTTTTAGAGGAACACCATTTGCAAAAAATCTTCATCATGAGGGCCATAGTACTCCTATCTTGCCTGGTCATGGTCTACGCAGCAGACAAGTACAGCTCCAAATACGACAACTTTGACGTGGAGACCCTGATCTCCAACGACAGACTCCTGAAGGCATACATCAACTGCTTCTTGGAAAAAGGGCGGTGCACACCTGAAGGAGCTGATTTCAGAAAGGCACTCCCAGAAGCGGTAGAGACCACCTGCGCGAAATGTACAGAAAAACAAAAGAACAACATCAGAAAGGTCATCAGGGCGATCCAACAGAAACACCCCAAGCAGTGGGACGAGCTGGTAAACAAGACCGACCCCTCTGGCAAGCACCGCGCCGACTTCGACAAGTTCATCCAGGGTAGTAGCTAGACCTGCTGCTTTTCTATTCTTCAACCATGACCGTTATCGTTGAGATATGTCGCTAGGTCACGTGACTTTTCTATTTAGGTCGTGGAGTTTTATTTTCTAGTTTTAAAGTAGTGATCTTTGTTTCAATCAAATAGATTTAAATGTGATAAGGGTAATAGTGTTTGTTGGAGTGTCAACATTTTAAAATTGATTTCTGACAGAAACGGATTCTTGTTCTGTAATTTTGTTTTGTTTGTAAGCGATATTTTAGTTGGGTACAATTACTTTCTAGATATTGCCTTCGAACTACGACAGGTAAGAGAGTTTCAAGAAAAGCATTTTCTATTAGAATGAAACAAATGATCGTTCATACCTATTTGTAGCTTTAAACATCTTAAGTTTGTCTTACTTTTAATCGATAGCTGACCTTTGGTATTTTACTCCACCATCAGTACAAACATTTTTTAGTTAAGTACTTTTCTATATAAAAAAGGGCTTGTAAAGCTCTTTTATTGCTCTCTATAATACTTATTTATTGTAAAACCTCGCCTAGATTTTTTTCTCTGATTTCTTACTACTCGTATTATATTATTTATTCCATATTGATTGGTCTTATAAATAACTTAGTTTTATTAATGCTAATGAAAAAACAGTGATATCATGAAGATCATGAAATTTGACTGATATTAATAAAGAGTTGGAATATTACCA

>CSP7 [Moltype=mRNA] [Organism=Loxostege sticticalis], complete cds

CGGACTTGGATACATTTGGCCAGAGATGACTGTAAAAAGCCAGACACTTGGCCTATTAGGGTTTTACCCTTTGTATACGGAACTCTTACAATTAAGATGACTAACTATGACTGATATAATTCTGTTATTAATTAAACTTATTTATAAACATTATCATTAGGCTTAACGGACACCATTTATTTAACTCCGACAGATGTTAACGACATTGTGACGACATATCATTCCGCGAAAACTAATAAACAAATGATGGTCAAAACAAGTTATTAACCAAATTGATGTTCTATTTCGTATATAAGTCAACGATCGCTGACCAATATTTTAATAGTCAAGTTCGTGCTATCAAGTTGTGTTAGTGACCGGATTAAAAAAGATGAAGTTCCTCCTACTGGCCTGTTTGCTGGTGTGCGCGGGAGTGTCCCTCGCGAGGCGGGTGCCCCGGTACACGGACATCTACGACAGCATCGACATCAAGACCATCGTGGCCAACCGGCGGCTGCTGCTGCCGTACGTGCTCTGCGTGCTGGGAGAGGGCAAGTGCACTGCGCCTGGCAGGGAGCTGAGATCCCACATCAAAGAAGCGCTTGAGACCCGCTGCGCCAAGTGCACCGAGGCCCAGCAGAGGGGCTCGCGTCTCGTCATCGCCCACCTCATCAACCACGAGCCTGAGTACTGGGCCAAACTGACTGCCAAGTACGACCCGGCCGGCAAGTTCGCCAAGATGTACGAGAGGGAGCTTAAAGAACAAGTTTAGGGAAGTTCAAATTGATAGGTCCTTTCGAGAGGCATTCTTAGAGAGTCAGTGTTTTGAAAACTTTTAGGCTACGTGATGTTATAAGAAAAAAAATACTGGCAACTCTCCAGGTTAACCTCGATAACCACGAGAACAAAAAACGTCGAAAAGGAAAAACAGAACTTGTTGTACCATTGTGTGTCATGTTCCTTCTTGAGATAGCCTGATTTTAAAAGTGTCTGCCAACAATAGGTACATAATTTAAATAGTCGGTGTCTCTGATGTTGTGATTATGAGGCTTGTATGTGATACTTATTAGGTCACCTACAGTGTATTTATTTTGATATGATTAAACAGGTTATAACAATAAAATGC

>CSP8 [Moltype=mRNA] [Organism=Loxostege sticticalis], complete cds

GAAAACCGATCGAAATCGCCCAGTGAATAAGGCCCGCGAGTGTTGCTCGAGTGCAACGGAGCCAAAAAGGGGGGGTTTGGTAGAGTTAAGTAAGAGGTTACGGCACACCAGGCCCCAACACTCGCCACCAAGACGCTCGTGTAAACGCTCCACTCCCAACCACTGTGAAATACTTTAGAAATCATAATTCCGAGATCCGGGTCAATCGTTAGGAACCACTCAGTGCAAGGAAAGTAAAAGTGCTACAGTGGGAGAGCACCGCTAACATGCAGATCGCCCTGGTTCTAGTGATGCTCGCAGCATGCGCGTACGCAGCGGAGACGCCGCGGCCGCAGGTGTCCGACACCGCCTTGGAGGATGCCCTCAACGACAAGCGCTTCATCCAGAGGCAGCTCAAGTGCGCGCTGGGCGAGGCGCCTTGCGACCCCATCGGGAAGAGGCTAAAAACTCTTGCGCCACTGGTGCTGCGTGGTGCCTGCCCCCAGTGCTCCCCGCAAGAAACGAAGCAAATCCAGCGCACGTTGTCGTACGTCCAGCGGAATTACCCGCAACAGTGGGCCAAGATCGTGCGCCAGTACGCGGGCTAACTTCCGCTTGCGTTTCCGTTGAAATAAGAACTCAAAAGTGAACTTAAAAGTTCGTGATTATTACAATGTTTGTGTGAAGAGTTTAAATTGCCGTAAGGTACATAGCTGTATATACTTACCTTTTTATCATTATGCAACTTGATTGCACATTAATTCTGTCTGAATTATTTATGAACTCTCAAGAAATCAGAGATTAAAACGATTTACATGAAAAATCAATATTTTGTAAATAGGTACTTGGATTGTTTAGTAAAATAAATCATATTTTTGACTAGGATTCAATTGCAATAACAATAACAAGATTCTTATATCTAGGACAGCAAAGACCTTCAAATAAAATTAGATAATAGATAGTCATGCATTTTATTGAAAAAAATACTTTTATATTTTTGTAGACATGGCTGTCTGTGGACCTGTGGTGCTTTAAGATATTTTTGTCGTCTTATTAGTGTAGATCCACATTTACCTAGATATTTTTAATAGAAATAACACTGTTTTGAGAGGATATTATTTTCCGTTCAAAAATGTTGTGTAGGTTGTATGTTGTTCTAAAATGTGTATTTAAATTATTGAACTATAAATTTTTATTAGGCAATAAAAAAGTGATAAATTAGATTAAAA

>CSP9 [Moltype=mRNA] [Organism=Loxostege sticticalis], complete cds

ATTTAAAGTTGTTTTTCTATTACATTCATACTTACTCCCACAACTGAATAAATTGTCTCCGTTCATAGGTATTCGTTCTAAGTCAGGTTTTTATAACATCTACTAGAGGTTTCGGGATACTGTACCGATCAAACACACAATCAACATGAAGTTCATCGTAGTTCTTGCTGTCATCGTGGGCCTGGCCATGGCTGATGAGAAGTACACCAGTGAGAACGACAACTTCGATGTGGATGCCCTGGTGGCCAACATCGACGAGCTGAAGAAGTTCTCCGGCTGCTTCCTGGACATCAACGACTGTGATGCTGTGGCCGCTGATTTTAAGAAAGACATTCCTGAAGCTTTCCAGCAGGCCTGCGCCAAATGTACCGACGCCCAGAAGCACATATTCAAGAAGTTCATTGCTGGTCTCAAGGAGAAGTTGCCCCATGACTACGAAGCTTTCATGAAGAAGTACGACCCTGACAGCAAATACTACCCGGCTTTGGAAAAGGTTATCAATGTTTAGGAGTAAGGATTTTTTTGGTAGAATTTGAAATAAAATTTAAGAAACAAA

>CSP10 [Moltype=mRNA] [Organism=Loxostege sticticalis], complete cds

GCTAATATGATGATTGCAGCGCCAGTCAGTCCCTATTCGTCGTCGGAACACCCAAATATCACACGCTATGTTAAGCTACGCTACGCTATGTTAACAATTGAAAATTAAATTTTAAAGTGTTCGTTACGAAGTGTGTCTGCCGATATATTACTTTAATAATGCAGAAGCTCATACTGCTGGCTTTGGTGTGCTCGCTGGGATGGGGCGTAGTGATGGCCGCCCCCCAAATGACTGACTCGCAGCTGGACCAAACCCTGGCGGACCGGAAAACGATGGAGAGGCACCTGAAGTGTGCTCTGCAAGAGGGCCCTTGCGACCCAGTCGGCAGAAGACTTAAAACCCTGGCTCCCCTGGTCCTCCGCGGCGCCTGCTGGCAGTGTTCGCCGCAGGAGACGCGCCAGATCCGACGCACCCTCGCCTACGTACAGCGGAACTACCCCTGGGAATGGTCCAAGATCATCCGCCAGTACGGTTGAACGTTGACATTCCACAATGCACCCAATGTACCTGAGGCCCTACTCCGTATGATGATGTCCTTATTTTTGGCTCTATCCAACACTCCCATCTCATTCACCCTGGGATACTATCTTTCCGTGTGATCTTAAGTCGCGCGGAAAGTTAAGGAAGCAGTACTTTTAAGGCTTTAACATAAGCCTTTCCATGTGTTCCCGCGGGATTTCAGCGTTTTTCTTGTTCAAACAGAGAATAAAAGATTTTCGTTCAAAATTTTAAAAAATATCAGTTAGTTTAAACTGACGAAATTAAAAATTTAAAGCAATGCAATATTATAAGCAATTCGGGTGTGACTGTTATTCACTTAGTAAAATACCATAGATAGATAGCCATATATAACGGACATCATCTTGCAAACTTTGCAAAGTAGGGCCTCTGACACCATAGATCTTACCTCAGAACTTGCCATGCCTTAAGTACCAACGATTATTTAATAAGCGGTTTATTTGCCAATTTTATTTATAAGTTTACGTAAACCCGGTTAAGTCTTTGACCCCGTTTAGTCATGCTGCTAAACGGATAAAGAATTATTGACTTTTGAGGAAAAATAAATAACTTTGTATCTATTTTGTTGGCTGCAACACGATTTTAATTGTTGCAATAAAATTATTGACTATGTTACGCACTGACAACGATATTTATTTATGATGCCCTCAATACCTTAATGTAAGTTAATTAAGTTTAATTTATCTAAGTAAATTGTTTGGATTTTTGATACCCTGTCCTAATTATTGCAATACTTAGTCGAAATAAATTTATTATCAAAAA

>SNMP1 [Moltype=mRNA] [Organism=Loxostege sticticalis], partial cds

AGACTGTCTTGTTGGCGGCACCACTGCGCACATCTGTAAGTGAATGGTGGTCCCGGTCATCCTTCAAGATGCACCTTCAGAAGCCTCTCAAGATCGGTCTGGGGATGATGAGCGCTGGTCTCTTCGGCATCATGTTCGGATGGGTGCTATTCCCTGTCATCCTCAAGAGTCAGCTGAAGAAGGAAATGGCGTTGTCAAAGAAAACTGACGTGCGAGGAATGTGGGAGAAGATACCCTTCGCCTTGGATTTCAAGGTGTACCTCTTCAACTACACCAACCCTGAGGAAGTCCAGAAGGGAGGGATCCCTATTGTCAAAGAGGTCGGCCCTTACCACTTTGATGAATGGAAAGAGAAAGTGGAGGTCGAAGACCACGAGGAAGATGACACGATCACCTACAAGAAAAGGGATTACTTTTACTTCAGACCAGACCTATCTGGGCCGGGACTGACAGGAGAAGAGACCATAGTCATACCTCATATTTTGATGCTGAGCATGGCAACCATCGTCCACAACGATAAACCAGCAATGCTGAACATGCTAGGCAAGGCCCTCAACGGTATCTTCGACGAGCCAAAGGATATCTTCCTGAGGGTCAAAGTCCTGGACCTCCTGTTTCGTGGGATGATCGTGAACTGTGCCAGAACGGAGTTCGCACCGAAAGCAGTCTGCACTGCATTGAAGAAGGAAGCTGCCAATGGACTTACGTTTGAACCTAACAACCAGTACCGATTCTCACTGTTTGGATTGCGCAACGGCACAATCGACCCGCACGTGGTCACAGTGCGGAGAGGCATCAAGAATGTGATGGACGTTGGCAAAGTGATCGCAGTAGACGGCAAGCCAAACCAGGACGTCTGGAGGGACAAATGTAATGAGTACCAGGGAACGGATGGGACGGTCTTCCCGCCCTTTCTCACTGAGAAGGATAACCTTGAGTCTTTTTCTGGTGATCTTTGCAGATCGTTCAAACCCTGGTACCAAAAGAAGACCTCGTACAAAGGAATCAAAACCAACCGCTATATTGCCAACATTGGGGACTTTGCCAACGATCCTGATCTGCAATGCTACTGTGACAGCCCTGACAAATGTCCCCCTAAAGGGGTCATGGACCTAATGAAGTGTATGAAGGCACCAATGTATGCAACCCTGCCGCACTTTTTGGATTGTGACCCACAGCTCTTGAAAAACGTGAAAGGACTCAGTCCTGATGTCAATGAGCATGGAATCGTAATCGATTTCGAGCCGATTTCTGGAACCCCAATGGTAGCCATGCAGAGGGTACAATTCAATATGATGCTTCTAAAAGCAGACAAATTGGATCTTATCAAGGAGCTTCCTGGGACTTTGACACCCATATTTTGGATAGAAGAGGGTCTATCTCTCAACAAGACCTTCGTGAAGATGTTGAAGCACCAGCTGTTCATCCCGA

>SNMP2 [Moltype=mRNA] [Organism=Loxostege sticticalis], complete cds

CGGCTAACTGCTGTGTCAATACGTGTTGTCTCACTATACTAGTATTCATTAAGACACCATTTACCATAATCGGAAAAAAATTGACAAGGAAAACTACACACGGACGGAACGCATAATCGTCCGTAAGTATTCTTTAGCGACGGACTTGTGTTAACAGTCAGTCATATCCGAAACAGCCATTAGACGATTAAAAAACAAAATTGTGTGTTCGAAATTTGAATTCAAGTGTTCTTTTTTATTGAGAAGTGTTCGAATTTCAAAGTGATGTTTTAATCGAATAAAGAATCAGTGTTTTTTTTTATTAAAGCAAAGTGAGTTTTGTTTTTAAAAGTGAAGAAAACTTTTTTGAAAATGCTCGGGAAACGTTCGAAAATGTTTTTCGGGATATCCCTGGGAGCGTTGGTAGTTTCAGTGATTTTAGCCGCTTGGGGATTTCCTAAGATTGTCAGCAAACAAATCCAGAAGAACGTCCAAATCGACAACTCGTCGGCGATGTTCGAGAAGTGGCGGAAGATGCCTATGCCCCTCACGTTCAACATATACGTGTTCAACGTGACCAATGCAGAGGATGTGAACAATGGAGCCAAGCCCAAGCTGCAGGAGATTGGGCCTTATTCTTACAAAGAATACCGCGAGAAAACAATTCTAGGGTACGGAGACAACGACACAGTCTCGTACATGCTGCAGAAGACCTTCGTGTTTGACCAGGAGGCGTCGGGAGGGCTCAGTGAAGATGACGAAGTCACTGTCATCCACTTCTCCTATATGGCTGCAATCCTGACGGTGAACGACATGATGCCGAGCATCTCTGGCGTGGTCAACGGGGCGTTGGAGCAGTTCTTCAGCAACCTCACAGACCCATTCCTCAGGGTCAAAGTCAGAGATCTGTTCTTCGACGGCATCTACCTCAACTGCGCGGGCAACCATTCGGCTTTGGGCCTGGTCTGCGGTAAGCTGAAGACTGACTCGCCACCCACCATGCGCCCTGCTGAGGACGGTAAAGGATACTACTGGTCAATGTTCTCGCATATGAACAGAACCCCATCCGGTCCATACGAGATGATCCGAGGCCGGGACAACGTCAAGGAGCTCGGCCACATCGTTTCATACAAGGGGAAACGTTTTATGAAGAACTGGAGCAACGACCAGTACTGCGGAATGATAAACGGATCTGACTCGTCAATTTTCCCTCCGATAGACGAGAACGATGTGCCTGACAAGTTGTACACTTTTGAGCCTGAGGTTTGCAGGTAACGTAATGGACCAAGAGCCCGTAAGTGCCAGATGTAACTTATTTGCTGATGAACAAAACGTAACGGTAGTGCTAGTACCTACTATCAAGGTATACATACCTGCTATCTTGCCCGTGGGCCGATTTGTATGTACAAGGTGTTACAATATTCTTTTCGCGTGCGCGACAGAGGAGCAGCTTTCCTGTTTTTTCTACGCCTGTTTGCAAAACCAGGTATGGAAGGAACAGTTTTGTGATAAGGGCCACCAACAAGCACAATTTAGTTTTTAACCATATTGATATTTTTCACGAGAAGATGGCGAAGTTTCGTCATCTTGTAAAATTTTCGAAATGATAAGTTAAAGAAAAATTTTTAGTTCTTTTGATGTTTTTTTTTTTTTGTTGTTTATTATTTCACCATTTTTCATCTTGCATTTGTCTCCATAACAAAATCTATTGTGTGAACTAGCTTTGGAAATTGTATATGCTGGTGTAATATTAATTGTAATTAAAAAGCTGTTTATTTCCTTAATAAAGATAAATAAATATCAGAAAATAAGTAACGTCTGGCTCTACCAGGCTCTAATTTTATAACTGCCATCATTAGCAATTTATCCTATACAGAGTGAATGAAGTCGTGTATTTCATTCAAAGCATGTTTTAAGCTGCTGATATATACAGCAACTTTACTAAGGAAACAAAATCAAAATCTCTAGAAAATCTTCGAAATAGAGGTTGGGAACAGAGTGCGTATCGAAACGTAGTCTTTTTTTAACTGGCCTGTATTACATGTTTAAAGTTTTTTTAAATGCTTTTTCAGGTCTCTATACGCAAGTCTA

>IR1 [Moltype=mRNA] [Organism=Loxostege sticticalis], complete cds

CGCTTACGAACGGCTCGCCGAGTTTAGTGGAATAACACATGATTTAAAATGCTCTGTGAATATTATATGTGAAATTATGTGGTTTTTGTGGCTCCTTTTAAGTTTTACCATACAACAATGCACCCCTCAATTCATCTTCAGCGAGAAAAAGGATATATCTTACCAAATAGCGGGCATTTTCCTGTCGGATGCGCAAACGCAGATGCTAGCCTTCAACGAAACTATGGCCAGCGCTGGTCTTGACCAGTTCCATCTGAGCCCCGCCATACTGCAGCCGACTAGCAAAGACAGTCTCGCCATTTGGAATGAACTATGCTCCAACAGAAACCTGCGACCGATAGCAATAGTCGGCCCTCAAGATCCCAAATGGGATGGCATAGTACGCGACCAATGTGCTCTCGCTAACATCCCCCACGTCCAAGCCTCATGGCAACCTCTAGACGCTGACCTTGAGCTCAATGATGAAGAAGAAGAAGAAGTCAGTGAGGGTGAGAACGAAGAAGAGCAGAAAGAAGTCAGCTTCAAGAAATTGTCTATTAACTTTTATCCTAATTCCGAAGAGATATCTCTAGCCTATGCAGCGTTATTGAAGTATTACCATTGGGAAAACTTTGCAGTACTTTATGAAGATGAATATGGACTATTGAGAATACAAAAAATCCTTGCTGAACATACAACAAACTTTCCTGTTATTGTACGAAAGCTGGAACCCGAGATGGATAATCGTAATGTGTTCAAAGAGTTATCGAAACATCAACTAAATCGAATCATGCTGGATTGTAATGCAAGTCGTATCTTAGACTACATGAAACAGGCTAGTGATCTAAAGATGGTCAATTATTACCAGCACTACATCCTGGTGACAATGGAGGCGTACATCGTGGCCGAGCAGCTGACCCACTACCACTCGAACATCACGTGGCTCAGCCTCACGGAGTACGACAAGCTCAAAGACGCGCAGCACGTCCTCGCGCCCATGGTCGGCAAGTGGATCACCAGGCAGATGATCCCGCCACCGCCGGTGACGGACTTCCCTAACGAGGCTCTGATAATGAACGACATAGCGAATCATCTCTTAAGAGCAATGCAGACAATTAAAGACAAGATCACATATTTCAAACCAAGGGCACCTATTTGTGATGCAGATGCGCAGCCCTGGCGGTACGGTGCTTTGTTTCAAAATGCTATACTGACGACACCTTCATACGGCGTTACTGGAAACATAGCATTTGATGAAAAAGGAAGAAGGTTCAATTACACTTTGTACGTCAATGAAATCCACGTGAACAAATTACAAACTATTGGCACATGGGCGTCCACTAACGGTACTGAGATACTAGAAGACAGACCGGACTCGGGAACCTTGGATCAGCAGCAGAGCAGCAAACATTTTGTTGTAATTTCCACAAAAGCCAAACCATGGTTTTACGACAAGACTCCATGTGTGGGCGAAGAAGAGTGCGAGAATGACGATGGTCTCAAGTACGAGGGATTCTCAGTGGACCTCATCAAGGAGATCTTCAGGTTCCTGAGAGAAGAGAAGTTCAACTACACGTTTGAGTTTATGGACGGCGGGGACAAAAAAGCGGGAACGGTAGACGAAACCACCAAGAAATGGACTGGGCTTATTGGAGATTTGTTGGATAACGTAGCAGACCTGGCAGTGTGTGATATAACAATAACAGAAGAACGAAAGAAAATGGTGGATTTTTCAGTACCATTCATGTCGCTCGGAATCAGTATACTCTTCACACAGAAGAAGGACCCAGAACTTGAACTGTTTTCATTCCTGAACCCCTACACTTTCGAAGTCTGGATGTACACAGCAACCGCTTACTGCGTTGTATCTGTGATATTATTTATTTGCGCAAGGTGATTAAGATCGCCCGATTAGATTAACAACTCATTAGAAACGTCATTAATTAAACGATTATTTTATCAGGATATCTCCAGCTGATTGGGAAAATCCACAACCATGCGATAAAGACCCGGAGGAGCTGGAAAATATATGGAATTTCAAAAACTGTGCGTGGTTAACAATGGGATCTATTATGACGCAAGGTTGCGATATTTTACCAAAAGCCTTTGGATCGCGTTGGGTGTGTAGTATGTGGTGGTTTTTCGCCATGATCGTCTGTCAAACGTACATCGCCCAGCTGTCAGCTTCTATGACAGCCGCAGCCGAGGAAGAGCCCATAAATAGTGTGGAGGACTTGGCAAAACAGACAAAGATCTTATACGGAGCCATGCGTGGTGGATCAACTCTTGAATTCTTTAAGACATCCAAGGACAAAATGTACAGAAAGATCTACGAGAACATGATGACAAACCCTGTGGTGTTGGTGGGTAGTAATGATGAAGGCGAAAAAAGAGTATTAAATGGTAAAAATCGATACGCTTTTTTCATGGAGTCCACAAGTATTGATTACAAACTGAAAAGAAAATGTGAGCTTACAAAAGTGGGAGGTGAATTAGACTCAAAAGACTATGGAATAGCAATGCCAGCAAACTCTCCCTTTAGGAGTCATATCAACAGAGCCATTTTACGACTAAAGGAACTAACAATCCTGGATGATATAAAAACAAAATGGTGGGATACAAAATATGGAGCTATAAAATGTCCTGAGAAAAATGATGCGGACGAAGTGGAAGGGTCGCTGGGAATGGAAAATTTAATAGGTGCCTTTTTGGTACTCATCGTCGGAATGGTGTTCTGTCTGTTTGTCACCGCGCTTGAATTCTTGAACGAGGTCCGGAACATCGTTGTGCGGGAACAGGTAACCCACAAAGAAGTCATAATAAAAGAGTTGAAGTCGTCCCTCAACTTCTTCCAATTGCAAAAGCCAGTGCTAAGAAACCCGAGCCGAGCGCCCTCCATGGCTTCCTCAATCGACACCGAGTTCAAGCAAGAAAAACAATCTGCCCTCATTGAAAACTTCTTAGAATTTGAAAAGGAACCCCAATAAATAGAATATATAAATATGTAAACGTCAGACAACACACCTAGTGTCATAGTCTCGATGTAAAGAATTAACATGCTTGCCACGAGGAACCAGACAACGTTCTTAAGAGGAAATCACAA

>IR7d.2 [Moltype=mRNA] [Organism=Loxostege sticticalis], complete cds

CGCTTGTACACGAAAGGCCAAATTATAGGAAGAGACATATTTTATCCTTCATTCGCTCAGAAAACACGACAATTCATTTTGTTCGGCACGGACCTAGTGAACATTGAACAAATGTTGGACTGGATGCAAAAACACCAATTCGACAACACAGGTAGATTCATTGTTACCTGCCAGTCTGAAAGGCCTGAGGATTGTGACGAAACTAAAGCAGTCGACATCTTCTGGAACCACAAAATAATAAATGTGGTATTCATGAAGCAGACGCCGGACGAGGAAGTAAATGGGTACACGTATTACCACCCAGACGAAAACTGTCGCAGCAGCCAACCTGTCAAGATAAAGACATTGAGATGTACTAAGTATAATAATACGACGCCATGCCTTGGAATATTTCCTAAGAAGATGAAAAACCTGCATTGGTGTCCGATTATTGTGTCTACTTTCAAGCAAATGCCATATATGAGCATAACGGATGAGGGTGTCCCCTACGGTGCAGATGGTGATTTGCTTCTGCTGATCGCAGAAGGCCTCAATGCAACTTTAAAGGTGATGACGCCGCGACGTGGCTTTGGCTGGGGCAACCTGGATGCCGATGGCGTCTGGGTCGGTTCCCTTGCTGACGTATTTGATGATGTTGCAAATTTTTCCATGACATCGGCAGCCGTCACCCTTTCCAGATTTACATACTTCCAAATGTCCACAGCTTATTACACAAGTAATGTCGTCTGGATCACACGTCCAGCAGAAAAGAAGCCTGCGTCGTTGAAATTGTTTTATCCGTTTCAAACTCTATCGCAGATCGCTTTAGCATTCAGCTTTATATTCGTTGGCTTGTGCGTATGGCTCGTCAACAGCAAATATTGGCCGTGGCATAATTCTACAGAGGAAGCTAGCAAATTGAGCAGCGTACTTTTCTACTCCTGGATGATATGTATGGGATTGCCATCAACGAAGTTACCCGCCAAAAAGGAATTTATATGTTTGGTTTTAATTTGGATATGGTACTGCTTTCTGATAAGAACTTTTTATCAAGTTTATCTGATAAATTCCTTGCAAGGAGAATTTTACTCTGACGAAATTGATACAATTGAAGATGCATACTTTGCCAATTATTCTTTCGGTGGTGGACCCGCTCTTAGAGACTACTTTATCGATCATCCCTTAGTTTATGACAATTGGAAAAATTTGAATGCCAGTGATATAATACCAACTTTAATCAACTTGACAGAAAAAAAGTTCGTGCTTGCCACGAACCAAGCAACTACGGAATCCATCATAAAGGAACATAATTTGATAGTGCACATTCTACCGCAAAAAGTAATAAATAGCCCCTCAGTAATTTTTTCAAAGAAATTTTCTCCTCTAGTTGAACCTTTAAACATAATTTTGCGGCGACTTCTTGAATCGGGTTTCACAGATAAGTTGTATAAAAACTATGCCACTCAGAACACCTGGAAATCTGATAATCCTGAAGAACCCATTACTTTGGAACACTATACTGGATGCTATGTCATACTTGTTGTTGGATGGGTAGTATCTACTTTAATATTTATCATAGAGTTGTTTTATACTAAATTACAGAAATGCAAATATTACATTATAAAGATGAAACCCAGATTGGGCTGCCAGATTATTTAGCCATAGACAGTTGAGGTAACGCTTATCCGCACTTACCGCGCCAAAGGAGGCGAATTTCGGATTATTTGTATGAAATCATACAAAATCTCCCTAACCTGAACGTTGGAAGAAATTGCTAAGAGAAATTCCTTCGGTCCGTTCGGCGCGGATAAGACAAGGGCGGTCACCTTCAGTTTTAAATGTAAAGCAATAACTATATATAGAAGTAAAATATTATCTCTGCTTTCTTAGTTGACAGAGGCAGTTGAAGCGATTCTACTTCCCGCTAACTTGTGTTTTTAGCTATGTAGGTGAGTATTAACAATAATGAAACTAATGTATTTCCTCAAAGATACCAAATAAGGGCACATAAAAAATGCATTTTAGACTCATTTAGAAATCACAAAACTAGGTAGGTAAGTACTATGTCACAGTAATTGATTGTAAAGTCCTAACATTTTATCAAATGGGCTTTTAATATACAGATAGATTAGCGCCCATTTCGTAATTGCTAACCGATAGATAAGAAATCCTAAAGGCACCTAAGTAATTTTTATCACAATGCAACATGTGAAATTGCAATTAGCTGTTGCATTAACACAAACTACAATGTACTAACAGATTATACGGTATTTTACTGTCGGGGATATCAAACATGGATGTGGACGTGTATTCGGATAAAAGTGACACATCGAATAATCCTGACGCTTTGATCGGCGAGCTCGAAGATCCAGCGGTCGTTGTCAAGATAGGCACTACCGCCGCAAAAATAGCCTATTACAATTTCGAATGGCGGTTTGTGACACTCGTCATGTATAATACAATGCTGCATATCGGCGTGGAATCATTTTTACAGGTTTACAATCAATCTGTCGCCTTAAAACTTGGCAAATTCGTCCCCAGACATCGTGTGCACGTTCCTCAATTTGTCATATTTGGCCAAGACATCGTTGAAGTATCAGAAAATGTGAATTGGCTACTACAAAACAGGTACGACAACACTGCTATGTTCATCGTTATTTGCTCATCTGCAAATTACAATAAGTGCAGTGAAATTGAAATGTTTCAAACGTTTTCACGACTTTACATGCCGAATGTAATTTTCCTGAAAGCCCCCGAAGATGGTGATGAGCCTATGGTACTTACCTACTACCCAGTACTACCAGGGGAATGTAGGAACAACAAGCCATCGCTCATAGAACTTTCCAATTGTGACAGAGATATGTGCTTTAAAAATATGTTCCCAGAGAAATATGGTAACTTGCATAAATGTTCTTTCATCGTATCTACGCTGGAGCAACCTCCGTTCATGCACCTGACTAGCGGAGTTAACGGCAGCCTGCATCCATCGGGTGCTGATGGGGACATAATAAAGCTTATCTCTGAAATACTTAATGCGACGTTGGTTGTTACGACTCCAGAAATCCGTGAATGGGGTCATTACAAAGACAATAATTGGACCGGTTCCTTGGGGGACGTTTTCAACAAACGAGCACATGCTTCCTCCTGTTCGGCCCCACTTACTCCACAAATACATGGAAATTTTCAAATATCCTTTAACTACGCCAGTATCGACATAGTGTGGGCAGCAAAATTACCAGCGCTTAAGCCTTCATTTGAAAAATTGCTCTATCCCTTAAAAATGCAGACCAGAATTGCATTATGT

>IR7d.3 [Moltype=mRNA] [Organism=Loxostege sticticalis], complete cds

AACTGACGGTACCGTGGTCGGTCACTCACAACATTACGCCGCTTTTAAACATTGATTAACCCTAAAGTTAATGATTACAATGTTGGCGGCGGAACGTAAACTCATTAACGTTTATTAACGCTAACTCGTCGAGAGCGCAACGTAAATTTAACGGTGATTAGCTGTGTTCGAAAATGTGGTTGTGATCGGTGGGTTTTGAAATGAGCATGAGGACACAATGGTGGTGAAGATTTTGTTTCTGATAGGAGTGCTAGTAGGATGTGGATTTGCGGAAGAGTATCGGATTGGAGGAATCTTCTACGAAGATGCAGAGGACCTGAAAGTGGCGTTTGCCTTGAGTGCAAAGATGTTAAACTTCACTCCTTCTATACGTGAGGTCTCAAAGAGAGGGGAAATTCTTGAAGTGCGACAGCAAGTCTGCTCTCTTGCTGAGGAAAGCGTGATAGGAATAATAGATGGCATTGGTGGCCGAGGGAGCGAGATCATCCAATCACTATGTGATTCGATGGAGCTGCCGCACGTGCTGATTCGGTACGATTTCACGTACTCAAGCGACTGGTCGTTGCTGAACTTATATCCCAGTCCAATAGTTTATAATAAGGTGGTAGAGCAGATGGTCCTTCGCAAGGAATGGAAAAACTTCACAATCATGTACATTCGGGGACGCAGTCTGTTCCGTTTGCACAACTTGTTGCAGATGGGCAACGATACGGACAACTACGCCATCGGGGTCAGGGAACTCAGTGGTAAAGATTATAGAGACGTCCTAATAAACGCAAAAGAGAACGGCTACACAAACTTCGTAGTAGACTGCCCTTCGAGGCACCTGGAGCAGCTACTCCTGCAAGCCCAGCAAGTCGGCCTTATGGCTGATGAGCACTCGTACATTATACTATCACCAGACTTATTTACTCTGGACATGGAGCGATATAGGTACGGAGGGGTGAATATGACTGGTTTCCGACTAATGGATTTGAAGGCGAGCGAGAATCTTTGGGAGTTTACAGAGCACTATAACGCTGAGACCGGCAAGACAATGCTTCCGGAGCAGTTAAAAACCGAACTTCTGTTGGTCTACGATGCAATACAGGTGTTCGCAGGAGCACTACAAAAGCTCAAAGGGGTTGAGGCTCAGCCGTTAAACTGTGAAAACTACGACGCTTGGATGTACGGATCGTCTCTCCTTAACTTTATGAAAACTAATAAAGTGGAAGGTTTAACAAGGACATTGTTGTTTGATGGGTTAGGAGAACGAACCGACGTTATTTTTGATGTGCTAGAGCTGACATCATCTGGAAACCAGACGATTGGAACTTGGAAACATAATAAACTGGATATACAAAGACCATTCGTTCCCGACGCAGAGTTGACCGAAAACACTGCTTTAAAAAACAAGACGCTTAAAGTATTGATAACGACGACAGGTCCATACGGCTACTTAAAACATTCTGATCAGAAACTAGAGGGCAATGACCGTTATGAAGGGTTCTCGATTGATCTAATTGAAAAGATTTCCGAAATATTGGGATTTTCATACGAGTTTGAAGTTGAAGAGGACAATGGAAAGCTCGTTGGAGGGAAATGGACTGGCATGCCAAGGAAACTTATGGACGATGAAGCCGACTTAGCAATCTGCGACTTCACGATAACCGCTCTCCGGCAAAGCGCAATAGACTTTTCTACGCCGTTCATGAGTTTGGGCATAGGAATATTGTACAAGGAGCCAAGCAAGCAGCCTCCTGAGATGTTTTCCTTCATGGCAGTTTTCTCTAAAGAGGTCTGGTACTACATGATGCTTATCCAGATGATGTTAGGCGGAGTCATGATCTTCGTGGGCCGCATCTCCCACAAGGAGTGGCAGAACCCGGTGCCGTGTATAGAACAGCCGGAGGAACTGAGCAACCAGTTCAGTTTCGCCAACTCTGTATGGCTTATTATTGGTTCTGTTATGCAGCAAGGTTCTGAAATCGCGCCTATTGCCGTAGCTCCTCGAATGATAACCAGTATCTGGTGGTTCTTCACCATGGTCATGGTGGCTTCGTACGTCGGTACTCTCGTCGCTTTCCTAACAGTTGAAAAGAACGTTCTACCCTTCCAAAATGTTGAGGAGTTGTTCCGACATAAGTCTATCTCGTATGGGGCTAAGAAAGGAGGTTCAACGCTTGACTTTTTCATGCAATCGAAGAACGAAATTTACCAAAGAATGTATCAGAAAATGACCTCGAAAGGTTGGCTTGTTGGAAACAATGCTGATGGCGTGAATCTAGTCGAAAACAGTACTTATGCATTTTTCATGGAGTCAACATCCATAGAGTATACCATGGAGAGACATTGCGATTTACTACAAATTGGTGGATTGCTAGACCAAAAGAGTTATGGTATCGGGATGAAGAAAAATTCAACGTTGAAGGTTTATATTGACCGTGCGTTGCTAATACTGAAGGAGAGAGGCGAGATACAAAAGCTGAAAGATATTTGGTGGAAAGAGAAGCGAGGTGGAGGGAAATGTGGGGAAAAACATGCAGTGGAAGAAAAACAATTGGGCATGAAGAACATGTTAGGCGCTTTCGTGGTTCTGGGCGTCGGCTGCGGGCTGGGGGTCATCATCTCCACGCTGGACATGTTGTGGGGCGTCTTCAAACGCTCAGTCAAATACGGAACAACCTTCAAATACGAGCTGGTAGAAGAACTAAAATTCGCTCTCAAATTCAGCGGAGACGTCAAACCAGTGAAGCGGCCTCAGAAGACCATGGAAGGAAGTTTCGAAGCTCTGGCTGAAGCAGAAGGGAAAGACGAAGTCAAGTCTTTGAAATCTCACCAATCAGGGCGGTCTACTTCCACTCACAAGACCCACCACTCTCACAGCTCCAGACATTCTTCACGCGGACTTAGCGTTGCTTTTGCAAGACAGAGAGAGTATTCATAATACTTTTTTTTTCCAAAATAAATAAGCCTAAGAGCTTTCGAACTACGTCTGATCCGAATCGTAGAACTATTTCTATGGTAGCTTACTCACTAGATCCGGCGTCCGTACTCCGTTTTCTATCCGGAAAAATCTCTATGACGGAGAAGATTTTGACAACCCGAAGTAGCCATGGTATTACTTGTATCCGAATTCGATCCG

>IR7g [Moltype=mRNA] [Organism=Loxostege sticticalis], partial cds

GGCCCATCAGAATGACTGCAAGAGTAGTTTCGTTATTAGGATATGGTTTTCTGTGATACATTTACTCTTTATAATTTTGGGTGATGTAAGTTCCTGTTTATCATGAAGCCAAAAATAGAAATCTGTTTACAATGGTTATTGATGTGTTGTTGCACGTTTGTTCACGGAGATAGAACCATTGGAGCGATATTTGATGATGGTACATTTCTAATGGAAGCCGCGTTTAGTGTAGCCGTAACTGCAGCTTCCGAAGGCCAAGAAAATCCATTTGAGGCAAAGGTTATTCGCACCTCTCCCGGCGACTTATTGGAAGCAGAACAAGCAATGTGCTCTCTTTTGGAAAATCATATTTTCGGGGTGTTTGGGCCTAAAACAAAAGGACTAATTGATCATATCCAGTCAATAGCTGACGTACTTGAGCTACCTCAAATACTGACAGAACCTGTAGAAACACAAAATCGCAATTGGTCAGCTGTGAATTTATACCCTAACCATATTGCCTATTCACAGGTTTTTGCAGATATAGTCGAAATGAAAGGATGGGAAGAATTCACGATTATTTACGAAAGCTCAGAACTT

>IR8a [Moltype=mRNA] [Organism=Loxostege sticticalis], complete cds

AGACAAGAAAATTAATACGCCTTGAAAATTGACGGTCATTTTGTCTTGAATTTCATGTAAATTCGCGCGCTTTCTTCTTTTTCTTTATGCTTATTGGCAGTCCACGCCCTTCTTCAGTCTGGTTGACCGAGGATGACTTTTAATTCGCGCGCTTTTATTTCTTTTTAAACTGTCAGAGGTCACCGTTTGGGTCAAAACTTTTTGCTCATTTTGTGAATAAAAAAAAAATGGTTTTGTAACTTTTTCCTTATGTTGGAAAAGTTGAGGATTCTTTTTTATGTTTTCTTGACGCTATTCATACCATATGTCCTACAGGAGTATATTTCAGCGATATTAATAAATAAAGTAAGAAGCTGCAACTGCAAGTATTGCGAAAAATAAAAGATGGACGTACCGTTGTTGTTTTTGTTAATATTTCTCATCAACTTGGGCTGCATCGTTTCGGAGTTGTCTCTTCGTTTTGTATTCATCATCGAAGTCCACGATTCGGACCTGGCGCACCAGATCGGGCGGGCGCTGAAGGTGGCTGAGGACCAGAGGACCGGCGTGAGGGTCAGCGATGCCGTGGTGCAGCTGGACAGGGAGAATGAGGAGGAGAGCTATAGGAGACTCTGCTCAGCACTATCCAAAGGCACATCCCTGGTCGTGGACCTGTCCTGGGCGCCGTGGGACATGGCACAACAGCTGTGTTCAGACGCAGGGCTGCCACTCGTCCGCGCGGCGCTGGGCTCGCAGCAGCTGTTGGCTGCCTTGGACCAGTACTTGGAAAGCAGAAACGCTACCGATGCCGCCATTCTAATGGAGAGTGAGGGCGAGGTAGACAAAACCCTCTACGAGCTGCTGGGCAGGTCCAACGTCCGACTGTGGGTCCACGCGGGGCTCACCCGTGACTCAGCCAAGGCCCTCAAGAGTATGAGACCAGAGCCTAGCTTCTACGTCATCGTTGGGGAGAGCGGGTTCGTCATGGACACTTACAGAAGGGCAGTAAAAGAGAAGCTGGTCCGCCGCGACTACCGCTGGAACCTCGTCCTCACAGACTACTCCGGAGACACCATTGACGTCGCCCAGCTGGTCCTCCCGACCATGATACTCCATGTAGACCAGGTGGAGTGCTGCCGGCTGCTGGGGCTGAGGGAGGAGTGCAGTTGCCCTTCGGATTTGAAGAGAAAACAGTACATCCTCAACGCCCTCCTCATCTACATAACCGAAACATTCTCCAAGCTGGAGAGAGAACTGCCTGTGGTGAGCACTAGGGTGGACTGCGACAATGTCCAGGGTTCAGAGATGAACGGCACCAGGGACAGGATCCTGAGGCAGTTCAGCGAGGATACTGAGATGAATAATGATACGATTTTCTTTTGGGATGATGAAAGGTCCGGCCTCTTCCTTCGCTCTAACTTCATACTGTCCACGTACAAGCCTGACGCAGGCTTGGAGACCGTAGCGACCTGGTCTGCCAACGAAGAGTACAAGCTCCTGCCAGGAGCGACTCTGGAACCTCTGAAGCTGTTCTTCAGGATTGGAACATCACCGGCAGTACCATGGACTCTACCGAAGATCGACCCTGAGACAGGAGAGCCGGAAGTGAATGAGGACGGCCAACCGGTTTACGAGGGGTACTGCATCGATCTAATAGCTAAACTGGCTGAGACCATGGAATTCGACTACGAAATTATAACACCAAAATCGGGGAATTTTGGGAAGAAACTGCCCAACGGATCTTGGGATGGCGTCGTGGGAGATCTGATGAGAGGGGAAACCGATCTGGCCGTAGCTGCTCTGACGATGACGGCAGAGAGAGAAGAAGTAATAGACTTCGTAGCTCCGTACTTTGAGCAGACTGGTATTCTTATTGCAATCCGGAAGCCGATCCGCAAGACTTCCCTCTTCAAGTTCATGACGGTCCTCCGTACGGAGGTGTGGCTCAGCATTGTAGCAGCCCTGGTGCTGACAGGGCTTATGATCTGGCTGCTGGATAAGTACTCACCGTACTCCGCGAGGAACAACCCCCAGGCGTACCCCTACCCATGCAGGGAATTTACCCTCAAAGAGAGCTTCTGGTTCGCGCTGACGTCGTTCACCCCCCAAGGGGGTGGAGAAGCACCCAAGGCCTTGTCTGGAAGGACGCTGGTGGCTGCGTACTGGTTGTTCGTGGTGCTGATGCTGGCCACCTTCACGGCCAACTTGGCAGCCTTCCTGACTGTTGAGAGGATGCAGACGCCAGTGTCATCGTTGGAGCAGTTGGCGAGACAGTCCAGGATAAACTACACAGTAGTGGAAGGGTCCACCATCCACCAGTACTTCATCAATATGAAGTTCGCTGAAGACACCTTGTACAGAGTATGGAAGGAGATAACTCTAAACGCAACGTCAGATCAAGCACAATACAGGGTTTGGGACTACCCCATCAGGGAGCAGTATGGCCACATACTATTAGCTATTAACGCTTCAGGCCCAGTACCGGACGCGAAAACCGGTTTCGAGCAAGTGAACGAACACACGGACGCTGATTTTGCGTTCATCCACGACTCTGCTGAAATAAAGTACGAAGTAACCCGGAATTGTAACCTGACAGAAGTGGGCGAGGTCTTCGCGGAGCAGCCCTACGCCATCGCGGTGCAGCAGGGCTCCAGGCTGCAGGAGCACCTGTCCAGGGCGCTGCTGGACTTGCAGAAGGAACGGTTCTTGGAACAGCTGGCTTCTAAGTACTGGAACGAGTCGGCGCGCCAGGCTTGCCCTGACGCTGATGAGTCTGAGGGGATCACTCTAGAAAGTTTGGGCGGAGTCTTCATAGCAACTCTCTTCGGCCTCGGTCTAGCAATGATTACTTTAGCTTGGGAAGTGTTCTACTACAAACGCAAAGAGAAGAACAAGGTGCAGGGTTTCGACTCAAAAATAGAGAAGGCTGCCTTCGTCGACACTAAAAAGAAGTTAGAAAAAGTGGGGGTGAGACTTAGGAAGAAGAATAAGTCGGGAAAAGTTGGCAAGGTGGATGTGATTGGTAAGGGGAAGGGGGTGACTATAGGGGATAGTTTTAAGCCCGCATCTGAGAAGATGGGGGTGTCGTATATTAGTGTGTACCCTAAGGGGGAGTATAGGCCGTAGATGTTGTTTTGAGCACGGTCCAACTGTCAGACCGTAAAGTGTTTTTCAAACTTTTTGGATACGTGATCCCTTACAACTTTACAAGTAAAAATTCTGGCGAACCCCTGGCCGACTATCTTCAAAATTACTTAAATAATCTGACTTTAAATTTTGCGGGGGATGAAAAAGTAGCTATGTATGTATATTTTTTTATATCATGGAGTATCGGCACTTTAAAACTCAGATACCTCGTAGTATGATCTTAGTAAAACATCACTGGAATGTGACTAGCATAAGTTAGCGTGTTACAAAATACAACTATAAAATAAAATTGCCATTAATATATTTTGTCTTTTTTTTATCAAATTTTAAGTTCTTTAGTTACAAATAATGTAAGTTTTAATTCCTACACCCGTTTTTTTATTACATTTTTTAATTAATTTATTAAAATAATACATGGTGGTTAAAAGACTCATACTGGCAATACAGCAAATAATAATCATACTATATTTCTCGATTGTCGTGAGATCGTGATTACAAAGGTAGGTACATAAATATTTGGTTAAAAATTAAACTAGTGTATTAGTGCTTTTCACAAGGACCCTTCCGCCAAAGTCCAAAAGTCTCCGTATGAAGAAAATAGTAGTGTTCACTGCCGCTGCCGCAGCGTGCCGGTGTGCGGGCGCGGCACTGACGTGTGCGGCCGCGTGCCGGGCCGGTTGTTCACGCTGGCGTACGACAGCAGGCGGTCGGAGAGGTCTGGTGGCTCCATATATTTGTTGTTGTTGTTCGCGGGCGGCGGGTGCGCGGTGGTCATGTTGTTGCCGGTGTACGCCACGCACTTGACCTGCCACTCGCCCAGCTGCTCGTTCCAGTGCACGTACTGCTCGATTAGCGCCTGATACTCCTTAGGAATATACGAGTCCAACACCAAGTCAGCGAGTTGTATCTCTCTCTTCAGCGCTCTGACGCTATCCAGTATCCCCTCCATCTCTCTCTGCTGCTCTCTTTGCTGGTCTGCCAATTCTGCCTTGGCCGAGTTGAGGAGTTGTACGGCGCGTTTGAGCTTGCGAGTCTTGGCTGCGTTCTCTTCTTGAAGACTGGAGTAGCGCTCTTCTAGGTCTAGCCTTTCAGCCTCTTTCTTCTGCAGGTCCTTCTGCAGCCGCAGCTCGTTCAGCTTCCTCTGCTCGAGTTCATAAGCGGCTTGCTCGAGCAGTCGCCTCTGGGTGTCCGCTTTGTCCAGAAGATTTTCTCCGCCTACCAGCACCTTGGATTCAAGGCGCTGTAGCTTCTCTTTGAGCTCTTCTTGTTGTTTTTTAGTTCTTTGGAGCTCAGCTTTCTTCTCTTCCAGAACTTTCCGCGCCTGGTGAGCCTTCTCTCTGTTCAATTTCTCCTTCTCTTCTCTTCTCATCTTGCGGCGCATCTTCTTCTCCTCCGGGTCGAGGGAGTCCATCTCGGGCTCCGTGAGCGTGTCGTCCGACAGTTCCCCTTCTGTGATCTCTTCCTCTTCTTCCTCGCCTTCGATGTCATTTGCTGTCTCTTCAAGTTGCTTCTTGAGCTGATCGATTTCTTGTTGGAATCTAGTGAGCAGCGCGTCGCCCGGCTCGCTGTTGACGTGCGTCTTGTTCTCGATGTTCTTGGCGCGGTTGGCGTAGCGCAGCGTGCTGATCGTCTCCACGTAGTTACACTCCGCAGGGCCGATGGTGGCTATCATGACAGTCTTGGAATTTCCACCCAGTGAATCTTGCAAAAGACGAGTCAGCTTAGAATTTCTGTACGGGATATGGGTTGACTTCCCGTCTACGAGAGCTGATATGACGTTTCCCAGTACTGAGAGGGACTGGTTGATCTTGGTTGCTTCTTTGAGACGGGTGCCGGTTGCTTGTGTCTTGCTTTGACGTTCGGAACCCGCCAAGTCAACAAGGTGCAGTTTCCCCATCTTCACGTGCACTTTCCCATCGTTGCCCTTCTTGCTGGATTCCACAGTGATCGAGAAGATGGCGTGCGACCGTGAGCTCTCCGTGTTCATGGCCGTGGCCCCGATGTGGCGGTTCTTGTTGCCAACTGCCATGATCTTTTCTAGCTCATCTGCGTTGTGGACTACATAGCCAGTAAGATCCTTGACGAAGACGCCGATGTCGGGCCGCTCCTTGACCTCGAGGCTCTGGTGCGGGTTGTTCCCTAGCAGGTCTCTGACCTCCTCGTTGTAGATCTCCAGGTACGTCACGCATACTAAGAACTTTTCATCGTCCTTGGCTCGCGCTATATGACTGAATATATGCGCAAAGGAATTAGGTATGATGCCACGCAGTTCAGGTGCACTGTTTGACCCGGCCATGGTGTAAGTCTTGCCGGTTCCCGTCTGTCCGTACGCGAAGATGGTGCCGTTGTACCCCTTCAACACTTCCTCGACTATGGGGTTCGCCGTTTGCACGTAGATGTCCATCTGGCTAACGTTGCTGTCAAAGACTGCATCATAAGCGTAGATCCTCGGTGGCTCAGGTGGTGTCACATTGTTTCTAGTCACAGCCACGGTGTGGTTCACGGGGTCCACGGAGACACAGTTGTACGAGTTCTCCAGCTTCTCCCGTTGGTCCATGGGACGGACACGGACCACCACACGGACATTCTCGACCGTGGGTGCTGCGAGCTCCGCGTCAGGCATTTTAACAGCTCCCCACTGGATGGATCACGAGAACATTAATTCTACTATGAGACACAAAGAGTTACGTCTTTTGTTTGCCGAATATTGTAAAAATCAATACCGACGACCTACTTTTCCGTTTCAATTCTCTTTGCGAGCGAAATAGAAATGA

>IR25a [Moltype=mRNA] [Organism=Loxostege sticticalis], complete cds

GGCCACCAACCCATCGCAGCGACGGACCATGCTCTAACAACACCTCACATGGATTCTCCAACATGGGAAAGAAATATATGCGGCTGTTACTCAAAGTCTTGCTGCTATTTTCGTTTGTTCGCGCGGCCATTTTTCAAACGACTCAGAACATAAACGTCTTGTTGATCAACGAGGAGAACAATGCGCTCGCGGAGAAAGCGTTCGAGGTAGCGAAGGAGTATGTGAGACGGAACCCAAGTTTGGGATTGGCTGTGGACCCGGTCATCGTGGTCGGCAATAGATCTGACGCTAAAGCTTTCCTTGAAAATGTTTGCAGAAAGTACAACGACATGTTATCGGCCAAGAAAACCCCGCACGTTGTCCTAGATTTCACTATGACCGGCGTGGGCTCGGAAACTATAAAGTCGTTTACGGCGGCACTGGCACTGCCGACTATATCGGGCTCATTCGGGCAGGCCGGCGACTTGCGACAGTGGCGGTCACTCGATGCAAACCAAACCAGGTTTCTGCTGCAAGTGATGCCGCCTGCTGATATCCTACCGGAATCTATAAGAGCGATCGTGACCAAGCAGGATATCACCAATGCTGCTATTATTTTCGACGAACTTTTTGTTATGGACCATAAATACAAATCTCTTCTGCAAAACATTCCGACACGCCACGTGATAACCCCTGTTAAGAGTTACAACAAAGACGAAATAAAAACCCAACTGCGAAGCTTGCGAGAGTTGGACATCGTCAACTTCTTCGTCGTAGGCAGTCTCAGAACCATCAAGAACGTGCTTGATGCGGCGGATGAGAATCAGTATTTCGGACGGAAAACTGCTTGGTTCGCCTTGTCGTTAGACAAAGGAGATATCACGTGTGGATGTAAGGACGCCACCATCGTATACATGAGACCGACTCCTGACGCCAAGAGCAGAGATCGTCTCGGAAAGATCAAGACCACCTACAGCATGAACGGGGAACCTGAAATAACATCGGCGTTCTACTTCGACCTGTCTCTAAGAACGTTTTTAGCCGTAAAATCACTGCTGGACTCTGGCAAGTGGCCAAATGACATGAAATATATTACTTGCGATGATTACGACGGCAAGAATACTCCTAACAGGACCTTGGACCTGAAGGCGGCTTTTCAAGAGATAAAAGAAACTCCTGCTTATGCGCCTTTTTATATACCGGAGGACGATCCTTTGAATGGCAGGAGCTACATGGAATTTAATACAGATCTGACGGCTGTGACTGTTAAAGATGGTGCTTCAATTGGGAGTAGAGTGTTGGGGTCTTGGAAGGCTGGGCTCTCCAACCCGTTGTCTCTGACAGACCCGGAGAATATGAGCGATTATTCCGCGCAATTAGTGTACAGAGTTGTGACAGTGGAGCAAGAGCCGTTTATAATAAGAGATGACGAGGCACCCAAAGGCTTCAAAGGGTATTGTATAGACCTGATTGAGGAAATCCGTCAGATCGTCAAATTCGATTACGAGATTGTGGTATCACCTGACGGAAACTTCGGCACGATGGATGAGAATGGAAATTGGAACGGGATTATAAAGGAATTGATAGAGAAAAGGGCGGATATAGGTCTGACCTCTTTATCGGTAATGGCCGAGAGAGAGAACGTCGTAGATTTCACGGTTCCGTACTATGACCTGGTTGGGATAACAATTATGATGAAATTACCGCGGACTGCGACGTCTCTTTTCAAGTTTCTTACGGTTTTGGAAAACGACGTATGGCTGTCTATACTGGCCGCATATTTCTTTACGAGCTTTTTAATGTGGGTATTCGACAAATGGAGCCCTTACAGCTACCAGAACAACAGAGAGAAGTACAAAGATGATGAAGAGAAAAGAGAATTCACTCTGAAGGAGTGTCTATGGTTTTGTATGACGTCACTCACACCACAAGGCGGTGGGGAAGCCCCGAAGAATCTATCTGGGCGGTTGTTGGCTGCTACTTGGTGGTTATTTGGCTTCATCATCATAGCGTCTTATACTGCTAACTTGGCCGCGTTCCTCACTGTATCTCGCCTGGACACTCCCATAGAGTCCCTGGACGACCTCTCCAAGCAGTACAAGATCCAGTACGCACCCCTGAACGGTTCCGCGGCTATGACTTACTTTGAGAGAATGGCGCACATTGAAGTTAAGTTTTATGAAATTTGGAAAGAAATGAGTCTGAACGACAGTTTGAGCGACGTTGAACGTGCAAAGCTAGCTGTATGGGACTACCCAGTCAGCGACAAATACAGCAAGATGTGGCAAGCCATGAAAGAAGCAGGCCTGCCTAACTCCATTGAGGAAGCTTTGCAACGAGTGAGGGATTCCAAGAGTTCCAGCGAAGGGTTCGCTTGGTTAGGAGACGCGACAGATGTCCGATACCACGTGCTGACCAGTTGTGATCTTCAGATGGTTGGCGATGAGTTCTCGAGGAAGCCGTATGCTATTGCCGTTCAGCAAGGTTCCCCATTAAAGGATCAGTTTAACAACGCCATTCTACAATTGTTGAATAAACGCAAACTAGAAAAGTTGAAAGAGAATTGGTGGACTAACAACCCAAAGTCTATGAAATGCGAAAAACAAGATGACCAGTCGGATGGTATTTCCATACAAAACATTGGGGGTGTGTTTATCGTGATATTTATGGGTATCGGCCTAGCCTGCATCACCCTTGGCGTCGAGTACTGGTGGTACAAGATTCGGAAGCGGTCGACCATCGGTGATGTTACTCAGGTGGAACCAGCAAAGTCATCTAGAAACCACACTGATATCAAAGGAGAAGGATTCACATTTAGGTCTCGAAATTTCGGCCTATCGAACTTGAAGCCTAAGTTCTAAATAGGGTTTCTCATTTCTCGACCTTTTTTATTTCCCGGAGCACGAGAATTCTCGACCACTATTTCCCGGGAATTCTCGGGTTTTCGAGAAATATGTTTTTTTTTTTAAATTATGAGTTAATTAAACAATAAAACTACGCTTTTTATGCAATATTCAACAACATTTATCAACCAACAACGTTTATTTTTAATTTCATGTGACTGTAACTTAACGATAGCTTTCTATAGTTAAGTTAAAGTCACATGATACTTTAATTAAGCGAAATAAACTCTAGCGAGATCTTCTTATCATTATTTCAACAAACAAAATTAGGACTAAATAGTCTTGTTCTTTGGAAAAAAACAAACATCTTTTAGTAAATTTAGTCTAAAAAACACTATCAAAACATGCTTTTACAAATGCACGTAGTCAAAGTTCATTACAAAATTTGTCCAAACACAG

>IR21a [Moltype=mRNA] [Organism=Loxostege sticticalis], complete cds

AACAAAACCTGTCACAACATGATACGTTTAAAATGTTTGATCGCAAATACTTTATTTCTTGTTACCATTGTAGTGTGTGACGACGTTGAGTATTATCCTTCTCAGGCTGTTCTCGATAGCTATTCAAATGTTGCAAAAAGGTCAATTAACGAACGTAAAATTGAAAAGCGGTCGTCCATAGAAAATGAGAAATTTAGATATTTCAACCAAATCAAAAATATTTCGGAAACAAGAAAGAACAATAAAAGAGCAGTAGATCCAGTATTTCGTGGGCACCCTAAAACCAGAGAAGAATTGTGGAACGAGCGTTTTATCAACAAAAGCTCAGCTTTTGATCAAACACCTTCATTGATAAATCTTATTCATAACATAACACTAAGATATTTGAACGATTGCATTCCAGTGATTCTATACGACAGCCAAATAAAATCACGTGAAAGTAACTTATTTCAGAATCTTCTCAAAGATTTTCCAGTCTCGTATGTTCACGGGTACATCGACGACAATAACAAATTGAAGGAACCCGACCTACTAATACCAGTAAAACAGTGTCTTCATTTCATAGTATTCTTAACTGAAGTAAAAACCAGTGCTAAAGTTTTAGGAAAACAATCGGAAAGCAAAATTGTGATTGTGGCTAGATCGTCGCAATGGGCTGTTCAAGAATTTCTGGCAAGTTCTTATTCTAGAAATTTTATCAATTTACTTGTTGTTGGGCAAAGTTTCAAAGACGATGACGATAATTCACTGGAGGCACCATACATCCTGTATACACACAAATTGTATACAGATGGACTAGGTGCTAGCCAGCCTATAGTTTTGAGCTCTTGGACGCATGGAAAGTATTCAAGAGATGTCAACTTATTTCCTCCAAAAATGACTGGAGGATATGCAGGACACAGGTTTATAGTCGCCGCTTCCAATCAGCCACCATTTGTTTTCAGGAGAATCAAAACAGATTCAGACGGTGGGAACTCTCGGGTTATTTGGGATGGGATCGAAATCAGGGTGCTTCAAATACTAGCAGAAAGAAATAACTTCTCGATAGAAATCTTGGAACCTCGAGAACCACATTTGGGGTAATGTTAAATAATGATAGTTGAAATCTAGTCACTTTAAAAAAAAAGGTTATTTCAGAAAATGTGTTTTCTAATTTAGGGGAGGAGATGCTGTCAGCAAGGAAGTAGAAATGGGTCGAGCTGATATTGCAGTAGCAGGAATGTATTTAACAAGTGATCGAATTAAAGAGATGGACATGTCTTTTTCTCATTCTCAAGATTGTGCCGTATTTATAACCCTCATGTCAACAGCCTTACCTCGATATCGAGCCATTCTCGGACCATTTCATTGGCATGTCTGGGTAGCCCTAACGTTCACCTATTTAATCGGTATTTTGCCTTTAGCGTTCTCTGACAAACATACTTTACGACATCTACTACACAACAGTGGTGAAATAGAGAACATGTTTTGGTATGTTTTTGGAACTTTTACCAATTGTTTTACGTTTGTGGGGAAAAATTCTTGGAGCAAAACTACGAAGATTACAACACGATTGCTGATTGGTTGGTATTGGATATTCACTATAATCATCACCAGTTGCTATACTGGTTCGATCATTGCATTCGTGACACTGCCAATTTTTCCTGAAACAGTTGATACCATAGAACAATTGATTGCGGGTTTTTACCGCGTTGGGACGTTAGATCATGGAGGCTGGGAGAGATGGTTCTTCAACTCTTCAGACCCGAAAACGAATAAGCTATTTAAAAAGTTGGAGCTGGTTCCAAATGTAGAGTCTGGAATCAGGAATACAACTAAAGCGTTTTTCTGGCCTTATGCTTTCTTGGGGTCTCAGGCTGAATTGGAGTATATTGTCCAAGCTAACTTTACTGTCACAAAGTCCAAACGTGCAATGCTTCATATTTCTCATGAATGTTTTGTGCCGTTTGGAGTTTCAATGGGCTTTCCCACCAATTCCTTGTATTCCGCTAAACTGAGTAGCGATTTAAGGAGAATGTTCCAAAGCGGTATCATTGACAAAATTGTGGATGAGGTTCGTTGGGAGATGCAACGAAGCAGCACAGGAAAGCTGTTATCTGCTGGATCGGAATCTTTAAAAATAACAAACGCTAAAGAGAAAGGTCTTACATTAGAAGATACCCAAGGCATGTTCTTGCTCCTTGGAGCTGGATTCCTCATGGGCGCATCAGCGCTCGTGTCTGAATGGATGGGAGGTATAACAAGGCGATGTCGCATCGGAAAAAAGAAACCTTCTAGCGCCAATTCTAAGCAAGAATTGATATCGACTCCCGAGATTGAAAATGAAGTAAAAGTTATAACTGATGCCACTGAAAGTAGAATCAACTTTGATAATAGGTCATGTTCATCAAGCGCTGGTTCAAGAGACACCCTCGAAAGTCAAGTAATAAACGTGACAGAAGAAAGTATTGAAGTGCACGAGAGTTTGGATGCAGCAAGGTGGGATTCCAGACGATCTAGTTCAGTAGACTTGGACCGAGAAGTTCAGGAGATATTTGAAAAGGATCTGAGGAGGCGGAAAATTGTGACGGATGATATTGACGAAGTAATAGATGAAAAAAGAGAACCAACAGCCTCGCATGGAGCATTTGGAGACCGTCTTAAGTAGTTTGACTAGAAATTACATCTTAGTAATTGTCATGATTTAGTTACAATGAAAATCAAAGGAATAAAAACATAAAAAGTACGAGTTATTAGTATTGTATTTATTTTTGGCCTTTCGTATGAGTTTACGAATAACGGTACGTTCTCTTGCTGCGAGTTTGTTTGAAATCGATATAGACGGAATGATGTTTCATCTTGTACTAAGTTTTCACTGGATAAAGAACTGTGAACAAATTGGACTTTGAAA

>IR40a [Moltype=mRNA] [Organism=Loxostege sticticalis], complete cds

GCCATAACTATTAGTCTGTTTCATTTCCTTACACACCTGAGCAACACTTGTACTTCTATCCCTTCAGCATGTAATGTACAATTTACTTCTTGGAGAAAGTAACGAAATGAAGCTCCTACCATTTTACCTTTTTCTCAACACCGCTCACTGTTTCATCGACATACAAGATATTGTATCGGAAACTATGACCAAACTCCCCAAAGATTTCGCGGTGGCTATCAAGGACATTGCCGAGGGTTTGCCAGCAAAAACTATAACCGTAGTGAGAGGCGAATCCACTAAAATCAGATCTCAAGATATTTTCCAACTCCTCTGTCTGCTAAGCGAGCATAACATCCCAGTTATTAATTTGGACATAACAACGAAACAAAGTAAAGACAAGTATTATAGCTTTGTGAAGAAAGCACTAGACGTGTCAGAGGAACGCACAAGTTTGATCTTATGTGAGCCGTATGAATGTGAAAACATACTGACAGAGTTGACGGACAACAATTTGATCCACCGTACGATCCTGTACATCTTCTACTGGCCTTATGGGAAAGTCAGTGATCAGTTCCTAAACACTATGAAGGAAGCAATGCGAGTTGCTGTCCTCACTAATCCTAGGGAGAGCGTGTTTCGAGTGTACTACAATCAAGCGACTCCAGATCGCCTCCATCACTTATCCCTAGTGAACTGGTGGTCTGGCAGCTTGTACAAGTCGCCAGTTCTGCCACCAGCTGAAAAGGTTTATCAAGACTTCAAGGGAAGGATATTTGATGTGCCGGTGTTGCATGCACCTCCGTGGCATTTTGTAAAATACAACAACGATTCTACTGTGAACGTCACTGGTGGTAGAGACGACAAACTACTTTCCCTGCTTTCCAAAAAACTCAACTTTAGATACCGATACTACGATCCTCCAGATAGAAGCCAGGGCTCCAGCATTTCTGGTAATGGAACGTTTAAAGGAACACTCGGTTTGATTTGGAAACGCAAAGCAGACTTCTTTATCGGTGATGTGACAATGACGTGGGAGAGACTGCAAGCAGTGGAGTTCTCTTTTCTGACCCTGGCCGACTCCGGGGCTTTTCTGACGCATGCTCCAGCGAAGCTTAGCGAGACCCTTGCCATTATCAGACCTTTCAGATGGGAGGTGTGGCCTCTAGTCTGCGCAACAGTCCTGGTGACTGGCCCTGCTTTGTGGGTCGTGATTGCTGCCCCGTCGCTGTGGCAGAAACGTCAACGAGACCAACTCAGACTCCTGAACAACTGCTGCTGGTTCACAACTACACTCTTTCTCAGACAATCCTCAAGCAAAGAGCCGTCTAAAACTCACAAGGCTCGCCTGGTCTCCGTCCTCGTGTCCCTTGGTGCCACCTATGTGATTGGCGACATGTACTCAGCGAACCTCACTAGCTTGCTGGCGAGGCCTGCGAGGGAGAGACCTATAGGGACCCTGCCAGCGTTGGAGGAAGCCATGAGAGAACGGGGCTACGAGCTGGTTGTGGAAAGGCATAGCTCTTCTTTAGCTATTTTGGAAAACGGTACCGGCGTATACGGCCGTCTCGCGCGGCTGATGCGTCGGCAGCGCATCCAACGCGTGCGCAGCGTGGAGGTGGGCGTGCGGCTCGTGCTCACGCGCAGGCACGTCGCCATACTGGGGGGGAGGGAGACGCTGTACTATGACACGGAGAGATTCGGGTCACACAATTTTCATTTGAGTGAAAAACTCTATACGCGATATTCTGCTATAGCCCTTCAGATTGGATGTCCATTCTTGGAAACCTTTAATAATGTTGTGATGACCCTATTTGAAGCTGGTATCTTAGCAAAAATGACAACAGATGAGTACAAGAACCTTCCAGAACAGTCTAGAAGATCGGAGCCGGTTACAGAGAGTGATAAACCAAACAACGAAATTACAGGAGATTCACCTTCGGCATCACAAAGCGGAACGACGCAAGGGGAATCAACAAAAGCGCTGGAGCCTGTCTCGTTGAGGATGCTGCGAGGCGCGTTCTGCTTACTTGGGATTGGACATCTTTTGGCAGCTATAGCACTCGGAGTAGAAATCCAAATCCACCGACGATCTAAGAATTTTATCAAGATAATGGAGCCAAATGGAGGGAAGAACATGCCTCGAATGAGGGCTCTGAAGAAAGCCAGCAAATGCGTCAGGCAAGGAGTCAGGAGGGTTGTCAGGGCTGTCTGCAGATCCATTGATAGGGCTTTAGGGCCTGGTGTGCAGTAGAGTTTGATAACGTGTGTTTGTAGTCATAGGAATTCTGATCACCTGATTGTTATATTTTTGTCGATTTGCGAGATGTTTCTGTCTTATAGCAAATTGGAATGAAGGATATAAGATTAGGCCAGATATTAGAGACAGCTAAGCCTAGCCTAGCTTTAGCTTAAGAGGTCTTAGTAAATTATATGTATAATAGGTGACAAAATAACATAAAATATAATACTACACACATATGGTGAAAGAATAGATAAACAAACTTGAAGATTGGGTACCAAAATAGCATACAGTACAATGGCATTCACATCCATAAACTGGGAAAATAAATAACAAAAGACTATGTATCATGCACACGTATTTTAATCAAGTAACGGTAATTAAATATTCATAAATGTTTTAACATCCGCTGCAATTGAAGTTCAATGTCCTCGCAAAACCAATACAATAAGGTTGGAATGTTTTTATATTGAATACAAATATCTTGTAAAATGTTATATTGTGC

>IR41a [Moltype=mRNA] [Organism=Loxostege sticticalis], partial cds

GGCGGGGATGCTGTTGATATTTACGAGTTAAAGAGGCCGCTCAGAGCCGGCATACAGTTATAGACGATGGACATGTTGGTTCTATTAAATCCATTGCAGTTTTGGAACATTAAAAATAAAGGAAGTTTATTGTAGTTAATTCTACGAAACACAATTTTGCTCTTGATGTTATTTCTATTGGCTCTACAAATATTATGTTAAATAACTCTTGAAGAAGGAATATCTAAACATATTGTTATTTGTCACATTTCTCAAATTTAAAAGAGTTCACTCAACACGGAATACGAGTATAGCCAGACCTCTATCTCATACAGTTTAGATTTTCTTAAAAAAAAATGATTCATGCAGTAAGAATGCTACCAAATCCAATTTCATTTTTGCCTATAGAAATTTTGCTGAGTACTATTTTTCATCAATATTTGAATAGTTCTTATTGTTTATCTTTAGTCTCGGATTCACCGCTTAATATTCCAATCAAAAATAGTTTCACGTATATCTCTCCAGAAGATGGAGAATTATTCGTAAATCAGTTATTGGATGTGTCTGAAATGGGATGCTCAGATTACATTGTACGAATGCAAGATCCAAAGAAATTCATGGCGGCTTTTGAAAAGGTTAATCATTTGGGAAATGTGAGACGCAGTGATCGGAAAATTGTATTTTTACCACCCGAAGAAGACAATAAAACGAGAATCGATTTGTTAGAAGTTCTAGCGCTTAAAGAGAGTGGATTTGTAGCAAATATACTACTAGTTTTGCCTACGACCGAGCTTTCATTATGCAGTTATTATGATTTAGTTACACATAAGTATGTGGGTCCTGATGATCAAGTTGATGAACCTTATTATTTAGATCGTTGGAATTCTTGTTCGTCAAAATTTGAAAAGAATGCAAACTTGTTCCCACACGATATGTCAAATTTACAAGGAAAGACCGTAAAAGTTGCTTGTTTTACTTATAAACCTTACGCGTTGTTAGATCTCGATCCTTCGAAAGAACCTTTAGGTCGTGATGGTACTGAAGTTAGAATAGTTGATGAATTTTGCAGATGGGTTAACTGTACCATTAGAATTGTTCGCGATGACGAACATGAATGGGGTGAGGTATATGAAAATCATACTGGAGTTGGTGTTCTGGGAAATGTGGTTGAGGACCGAGCTGATCTTGGAATAACTGCATTGTATTCCTGGTATGAAGAATTCCTTGTTTTAGATTTTTCGGTATCTGGTATCCGAACCGCCATTACTTGTGTAGCGCCTTCACCAAGGTTACTGGCTAGCTGGGAAATGCCACTTCTCCCTTTTAGTTGGTACATGTGGTTAGCGCTGTTCGTTACTTTTATCTACTCAAGTTTGGCTCTCACTGTAGCGAAGGGGTTCTCAATGGATAAAGTTTTTCTGACTACATTTGGTATGATGATCACACAATCCCAGGCTGACGTCGGTGCTTCATGGCGTGTTAGAAGCATCACCGGTTGGCTGCTGTTAACGGGGCTCGTTCTTGACAACGCTTATGGTGGAGGCCTAGCTTCTGTCTTCACTGTTCCTAAATATGAAAAACCTATCAACACAGTTCAGGATATAGTAGACCGAGGAATAGATTGGGGGGCCACCCATGATGCTTGGGTGTTTTCT

>IR64a [Moltype=mRNA] [Organism=Loxostege sticticalis], complete cds

CATTTGAAACACCATGGATTTCAACTATTTCCTGAATTTTATTTCAATAGCAGAAATATCTCTTATAATTGACTTACTGAAATTCAAAGACATAGGAAATGTGGTAAATATTAATTGTGATACTCAAAAGAGCATTTTTTTGCACAAAATGTTTAATGACAATACTATCCATGCAGCATATTGGAATATAAACTCTGATAAAAATGAAATACCGAATTCTTATCGTAAAACTGGGATAATTCTAAATGCCTCATGTAGTAACTGGGCTCAAGCTTTCAATAACATTTCTGACTACAGTATCTTCAAAAATCAGTTTATATGGTTTGTTCTAACTGAGGATTTTCTGTCAACTGTAAGATCATTGTCTAGTTACCCCATTGAAATTGATTCTGATGTCACAGTGGTTCATAAAACCGATGGTTTTTATAAGTTGTATGAGGTATACTATAGGAATTATTCGAATGGCGTTTTATCAATACGCGAGATAGGATATTGGGACACATTCTTGCGCGTGAATTCGTCTAATCGAAACGATCTACATGGGTTAGTAATGAGATGTCCAGTTGTAGTCACCGACAAGGTTGTGCAAGAAACGTTTGAAGAGTATTTGAGCAAGCCAAAGAAGAATCAAGTGGACTCTCTTCACAAGTTGAAGTTCTTTGCGCTGCTGAATTACATACGGGACATGTACAATATCAGTTACGACATCCAGCGCACTAACTCGTGGGGCTACAACACGCGCAACGGGTCGTTCGACGGCGTGGTGGGCGCCCTGCATAGGAAGGAGGCCGACATCGGCGGCTCGCCGCTGTTCTTCAGAGCCGATCGAGCGGAGCTCATCGACTACATAGCCGAGACTTGGCAGTCAAGACAATGTTTCATCCTGCGTCACCCGAAGCACCCCGGAGGCTTCTATACGATCTACACCCGCCCCCTGACAGCCAGGGTGTGGTGCTGCATCCTGGTCATGCTCGTGTTCTCGGCGGTCATCCTGTGCCTAATGCTCAAGACCAAGATCCCGCCGTCCAGCGATGATAGCGCAGATTCCTCGTTCAGTTTGGCCTTGCTTTTTATTTGGAGTGCTATTTGCCAACAAGGGATGTCAGTGAACAGAAGTTCTACATCGGTGAAAATGGTCGTTCTCGTAACTTTCGTGTACGCTGTGACATTATACCAGTACTACAACGCTACGGTCGTGTCGACATTGCTTCGGGAGTCGCCGAAGAACATCAGAACGCTGGAGGATTTGCTGCAGAGCAACCTCAAGGCTGGCGCTGAGAACGTTCTTTACGCTAAGGATTACTTTAAGCGAACCACAGACCCAGTAGCTCTAAGGATGTACCACAAGAAGATCGCCCCCAGTCACCAATTCAACTTCTACGCCCCAGCACGCGGCATGAGCCTGGTGAAGAAGGGGGGGTTTGCCTTCCACGTGGACAGCGTGGTGGCGTATCGAATCATGCGCAAGACTTTTACGGAGCGGGAGATCTGCGAAGCACATGAGGTGCTGCTGTACCCGCCACAAAAGATGGGCATGGTCATCAGGAAGGGATCGCCTTATAAGGAACATTTCACTTACGGAATCCGCAAGATCTACGAGTCAGGCCTGATGCACCGTCTCAAATCAGTATGGGACGAGCCCAAGCCAGCGTGCGTGCGCACTCCGGACAGCAGCGTCTTCTCCGTCTCAATCATCGAGTTCTCCACTGCTCTCTTTGCGTTGGTAGCTGGGAACGTCTTGGCCATTTTGGTTTTGTTGTGCGAGATTGTCATGCATAGGTGCGGAACGAATAACCACATAGCTTTTTTTACATTAGGTTCGGTGTTAGTTTTGTAGAAAATATCACCATAATTTACAATGGTATTTAAAAAAAGTTTAGCTAGCACGTGTGAACATGCAATGCAGTCTTATTTATCTCTGAATATCTAGGCATAGAAAAGAAAGTTTGCAACAGAAAATAGAAATAATATGGCGGTTTTTCAATTATTCTTGCTTGCTAATTATGACGAATTTAATAAAACAATAAATCATCT

>IR68a [Moltype=mRNA] [Organism=Loxostege sticticalis], complete cds

AAATAACAGGGTGCTTAGGTAACGGAAGCATGTGGTTGACCAGCTAATGAGACATATAAAATATTCATGTACCTAATTTGTGGACTTCGCTTAGGCACATTTAAAATACAACGCCCGGCCGATTGGGTTTGCATGTTACATGAGCGTGTCAATCAGTACCACAAACAGAAACAGTGCAACATAGCTTGATGGTTTGGTGCGGAGTCGCGGAACAAACATGTTCAAAGTAGTCGTTTTGTGTATTTTGTTGGCTAACACTGCTGCAGATATCTCCCCCATCCTCAGAAGCCTGAACGAGCGGAAAGACCTAGAGCTGGTTCTGGTTGACCTGATCAATGGGCTGTCCCGGCGAGAGGACATCACCTGCGTTGCCTTCATCTGTGATGCGGTATACCTCAACGTGTTCGAGGGATACCTCTTTAAGAGGACGGATGCTGCACCGTATGTTATGATCGTCGTGGAAGATTATGAGGATTTACTCTCTCCTAACTTTGACACGCTGGAATCGCTAAGAGAGACAAGGAAAGACGGCTGCAATGTTTACGTTATTCTTCTCGCCAACGGTCTTCAAACGGCCAGATTGCTACGATTTGGAGACAGATATCGAATCCTAGACACCAGAGCAAAATACATACTGCTCCACGACTACAGATTGTTTCACAGCGATCTCCACTACCTTTGGAAGCGAATTATCAATGTAATCTTCCTTCGCTACCACAGCAAAATCAATGGCGTCCGCAAAAGCAAAGCTTGGTTCGACCTCTCCACTGTCCCCTTCCCGAATCCTATCAAAAGCGTATTCGTATCTCGAAGAGTCGATATTTGGAATAACGGCAGATTTCACTACAATAGGACCTTATTTGCCGACAAAACCAGCAATTTGAACAATGAAACGTTGAATGTGGTTTATCTGGATCACGTACCATCTGTGGTAGTAACAAAAACGAATGATACAAGCAAAGTAGGAGGTGTTGAAATTGAAATCCTCAACACCCTCGCCGAGAAAATGAACTTCCTGCCCAAATTGTACCAACCAATCAACGCGGATCTTCACAAGTGGGGTCAAAAGCAGGCCAATGGGTCTTTCTCTGGCCTACTCGGTGAAATGGTCAACGGCCAAGCCGATGTGGCTTTAGGGAACCTTCAATACAACCCTTACCACCTGGAACTGACTGACCTGAGCATTCCTTATACAACACAATGCTTCACGTTTATCACACCAGAAGCTTCGACTGACAACTCTTGGAAAACATTGATATTGCCTTTCAAGTTAAATATGTGGATTGCAGTGCTATTAGTATTGTTATTATCAGGGACCATCTTCTTTGGCTTAGCCAGATATTACATGCACTTGCAAGAATTCAAAAAGACCCATGATAAACGTAAAAACATCACTGATAAGCAAAAAATGCAGATAGAAGAGCTAGACTCTGACGAGAAGCCGGCCGGCCTTTACTTGTTTGGCGAAATAATAAACAGCATACTGTACACGTACGGAATGTTACTTGTCGTATCCCTACCTAGACTACCCACTGGTTGGTCGATAAGGTTGTTGACTGGGTGGTACTGGCTATACTGCATACTCCTTGTCGTGTCATATCGAGCTAGCATGACTGCTATCCTAGCCAATCCTGCTCCGAGAGTAACAATAGACACCTTGAAAGAGCTCGTCGAAAGTAAAATCGCTTGTGGTGGTTGGGGTATGGAAACCAAGAAGTTATTTGAAGACTCTGCTGACGATATTAAGACAATAGGTCAGCGTTTTGAAATCATCAACGACCCGTTTGAAGCAGCGAACAAAGTCGCGAAAGGTGCTTACGCTTATTACGACAATCAGGACTTTTTGAAGTATATTAGGGTGAAGAGGAAGAATGTTGAGATGAGTATAGAATCTGAGATGGTGAATGGCACTTCAAACTTGACTGATGCTTCAAGTGATGTCGGGATCGAAAGGAACTTGCACATAATGTCTGATTGCGTTGTGAATACACCTATTTCCATTGGTTTCCATAAGAATTCGCCTTTGAAACCACTCGCAGACATCTACATGAGGCGGATTGTTGAAGTAGGTCTTGTTGAGAAGTGGTTGAATGACGCCATGCACCCCATAAAATCATTAGAAACGAATGAGGAAGAGATAAAGGCATTGATGAATTTGAAGAAACTGTATGGCGCATTTATTGCTTTGGCGATAGGATATCTCATTAGCTGTATTGGTCTGATAGGAGAATTGATACATTGGCACCTAATTGTTAAACGAGACCCGAAATTCGATAAATACGCCCTAGATTTGTACTACATGAATAAGAATAAAAAACAGTAAGATTAGACAATTCTGTGTTTGACCAGTAGTAAATGAATAATAATAAAAACAATATTTGCACCATAGAATCAATTTATTAGAAATATTTACAGTATAATAACATGATTGTTTAGAATAAGAACCTTGTGGTACTGGAATAAATAAATTTGTTCAGTCATTTTAAGTCTTTACACAGTCCTTGCCAGTCAGTACCATATCATCAAAGTTATTACATAAAACAATAATTAATAGTTATTTTGTGATCACACTCCTAAATGTTTGCCATTGAGTATGTAAGGAATGACAAAAATCCATAATTTCTTCAAGATGACACAATCAATTAAATCATTATAAAGTATTTGAGCGTTTTGTCTCAAGTTTACAACATCAAACTATTAAAATAAAATGTATAGCTAAATGATAAATTGAATGCATAATTAAATTTAAAGTACAAGATTGTGCTAATACTCTTATAAAATATACAATTAGTTAATTAGTGGTGTTGCCCCTGTTCTGGATTGTGAGGCGGTGGCACAGCACCTTCCGGGGCGTTCTCGAGGATCCACGGGTGCTGCAAAACACTGGTCAAAGGCAACCTCTGTTCAGGGTTAATGACTAGTAACTTCCCCATCAAGTCCTTTGCTTTCTCTGAAATAAATTCTGGATATTTAATTATCACATACCTAATCTTCCTATATGTTTGACTAGAGTCTTTAGCATCAAAAGGAGGTAATCCAACTAGCAGTTCGTAGCACAACACTCCCAGACTCCAGATATCAACTGCATAGCTGTGTGGTTTGCCTTCAATCATTTCTGGTGATAAGTAATCAAGTGTCCCGCACAGTGTCATCCTTCTAGACGATGGAGAATGGACTGACCATCCAAAGTCGGCAATTTTCAGCTCCCAGTTGTGTCCAATCAATAAATTCTCAGGCTTTATATCACGGTGAATCACTTTCTTGGCGTGGCAGTATACCAAAGCCCTTGTCAAGTCTCTGACATAGATTGCTGCAGTTTTCTCGTCGAAACGCCCGCGTTCCTTCAGCAGCTTGTAGAGTGCACCATGCTTTGCGTATTCCAGAATTAAATAAATGCGTTTTTCGTCATGAAAGTAGCCGTACATACGCAGAATGTTGGGGTGACGTAGTCTGCACTGTATTTCCACTTCACGACGGACCTGGTGTTCTATCTCAGAGTCAAGGATCTGACTTTTAAAAAGCACTTTCAATGCAACGACGTAGTGCGATTCTTTCTCGCGCGCTAAGTAAACATTGCCGAACTTCCCCTTACCGAGCGGCCGACCCAAATCGAAGTCGGATAGCGACCAAGATTTCTTCTTGTCATCCTTCTTGCCCGGAAGATCACATTTGAGACCAGAAGAGCATGGCTCCTTATTGTCGTTTAAGTTCACTTTCGTACTGGTGTGCAGTGGTCTGCTGATCGGTACAGCTGGTACTTTGAAGCTGTCAGCTGGCATCTTGTGAGGGTTCGTGGAACAACTACTACAACCAGCCCGAACTGAGTTTTGAGGACGATTCTCCTTACATCCACATTTTTGCACATTCATCGTCACAAAATCTTATATTTCATGCAAATAATATAGCCACTTTGTGTTCGCGGAATTTGAAGATTCGAATAACGTATTTTTGCGTCAGAAAATAAACTATTTT

>IR75d [Moltype=mRNA] [Organism=Loxostege sticticalis], complete cds

CTTTGCATGCGTTTGTAGTCTACTGGACTCGTCATCATGGATACAATAAGCTTAATACCAGCATTTTTCCTTTCGAAAAATATATATTTTTTGACAACTTTCCTATGCTGGAATTCTGAGGAGTTGCACAAGCTGTGGCGCTTGGGGCAGCAGCAGGGCTTGCGGGTACGCGCGATGGCTGCGGGCCCAGCCACGCCGCCCCTGCCGCCCGACGACCTGCACCGGGAGGGGGTCGTGCTCGACCTCGCCTGCCCCTACGCCGACCACATCATACAAGCTGCATCAGAGACCCGCGGCTTCAACTACCGATACGCTTGGCTTCTTCTCCACAACTCCTCATTCGACGCTACGTCATTGGACTCCGTGTTGTCTGGCTCCGTGATCCTGCCCGACGCGGACGTCACATTCGCCTCCGATGACAAGTTGCTGGACGTTTACCGAATAAAGGCCGATCAGCCTTTGTTGGCCACCACTTTGGGTGTGGTCAGAAACAGTACCCGGCGGGACCTTGAGCAGATGTGGGGGGTTTTAAAAAGTACTGTCTCGCGGAGAAAGAATTTGAACAACGTGTTTTTGAAAGGTGCCACTATAATAACTCAGCCGCAGAATTTCAAAGGTTGGAATGACTTGACCGTGAGACATATCGATACTTTTCCGAAGTTGATGTACCCACTGCTAATGCATTGCGCGGAAGACTTGAATTTCCGCCTGAACCTGCTCCAAGTGGAGTTGTACGGCGACGAGCGCAACGGGTCGTTCGATGGACTGGCGGGGATGCTGCAGCGGCGCGACATCGAGGTGGGCGTCACCACGCTGTTCATGCGCCACGACCGGCTCAACGTGATGCACTTCTGCTCCGAGACCCTCGAGCTCAAGGGTGCGTTCATATTCCGTCAGCCGCCGCAGTCGTCGGTGAACAACGTGTTCCTGCTGCCGTTCAGCCGCGGCGTGTGGGCGGCGAGCGCGCTTGTGTTCACAGCGGCAGGAGGGCTGCTGGCCGCACTGAGTCGCCCGCGCTGGCTGCGCGACGCGGACCCCGACCTGGTGCAGCTCAGCGCCGCCGAGGCGTTCACCTTCGCCGTTGGAACCATATGCCAGCAAGGTTGCTACGTGAACCCGCACGCGGTGTCAGTGCGCATGCTGATGTTCTTCACGCTGCTGGCGTCGCTGTTCACGTTCACGTCGTACTCGGCCAAGATCGTGGCCATCCTGCAGACGCCCAGCGACGCCATCCAGACCATCGACGACCTCACGCACTCGCCCATGGCGCTCGGCGTCCAGGAGTCCACGTACAAGCGGGTCTACTTCGCCGAAAGTGACGACCCCGCCACGCAGCGGCTGTACCGGCGCAAGCTGCTGCCGCAGGGCGAGCGCGCGTACCTGAGCATCGTGGACGGCATCGCGCGCGTGCGCAACGGCCTCTTTGCCTTCCAGGTGGAAGAGAGTTCGGGCTACGACGTCATCAGCAAGACGTTCACGGAGCAGGAGAAGTGCGGCCTGAAGCAGATCCAGGCCTTCAAGCTACCCATGGTGGCCGTGCCCATCCTGAAGCACTCGGGCTACAGAGAGCTGTTCGCTGCCAGACTGCGCTGGCAGCGGGAGACGGGGATCATGGACCGCGAGCGGCGCGTGTGGATGGCCTCCAAGCCGCGCTGCGACTCCGACGGCGGCGGCTTCCTCAGCGTGCGCCTGTCGGACGTGCTGCCCGCCGTGCAGGTACTGATTTACGGCATGCTGCTGGCTGCCATTCAGCTGTTCGCTGAGATAGCCCTGCATCGCGCTACGGAGCGGATCAAACGTAAAAACAAACTTAGAGGAAAGAGGGAATAAAGCTGCGCTTAATTACTTAGTATCACATTTATTAAAACCATTTTATTTAAGTCACTATCTAAACATTCGGAAGTATTTACAATAGCAGATTTTCACTTAGTAGACCACACTATTTTGCATACCTTGAACCTATATCAACAAGTAGCAATGAAGGCTTGAAACATCACGTTAGCGTCTCAATTTAATTCCAGAAAATTTGAAACACTTAACTAATTACTTAGCACTGAATAGATAATTAATCATAGTTACTTTAAATTCACGTGACCTGTCTCGCGTCTTATTTTTTATGCTTAATAATTTGGAACACAAAACAGATTAGCATTGAACTGATTACCAAGTTACAACGCGTAGGTACACATAGCAAGGTCAGTAAAAAATCGGAGTCACAAACATGCCTACAGACGTAGTTAGTAGTAGGTCTGGATTCTACAAATAGCTTTTATTGTATAGCTTGTATTATGATGAGTTGTCCATGAAACACTTGAGTGTAAATAAAATGTGCATTCACAGAATTTTGACGGGTTTCCTACATCGGGGCAATATCAGCGTTTAATTTATACCTCAATAGAAGAACACTCCGATCATCTGAATCTGAAATACAGCTTAAGGTGTGATTCGAAACATCCAGTGGTTCTTGAAGAATTGGCAAGTCTGCTTCGGAGAAACTTGTTATGAACTATAATATATCCACTATTCTTTTTTATTTAACACTTTCGCGAGCGAGCTGCCTGTCCTCGTGGCTGCACGGGGCTAGAGGTAGACGCAGACGGCGACGAAGACGGCAAGCAGCAGCACCGCGAGCAGCGCGTAGTCCAGCAGCTGCGCCAGCGCCGCCCACGCGCCCACCTCCGCGGTCAGGCACGTGTCCTCTATCGGTGTGAGCCTGCACAACGTAGACAGTGTGGAGGCGAGGGTCCTCAGCAGGCGCGGCGGCTGCGACTTGCAGCGCGCCACCCGCGCCACCAGCGCCGCGCACACGCCGCCCGCGCCGCCCACCACGCACAGCGCGCAGAACACCGACACCGCCGTCGGCGTCGTCGAGGACGACGGCAGCCGCAGTAATGCTGATACGAGCCAAAGGGCTGCAGTAAAAGAGGCACAGGCGCACAACGGGGCCCGGACGAGCGGCGGCAGCAGCGCAGCACTCAGCAGCATCAGCGCCGCGGCCGCCAGCACGGCGCCGGATGCGGCGTCGTGCGCGGCGGCGCGCCGGCGCAGCACCGCCCACCACGCCGCCACGTGCCGCTCGCCCTGCGACGTCAGCTGCCCCGTCACGCGCTCCAGCTCCCACGAGCCTGTCTCGAATATCACTGCAGTCGTCGTCTGGTCGATTTCCAACTCAAGTTCATCAATCGGGTGCGTTCTAGAACCGAACTTGAAGACGGCGGTCTGCTGGTCGCTGGGCCAACCGGCCAAGTCTACGGGCACGGGCACGGCGGCGTCGAAGCGCAGCACCAGCGACACGGCGCCAGCGCTGGTGACGCGCGCGCTTAGCGCCGGCGCCGCGCCGCCGCCCGCGCCGTTCAGCAGCTCCACGTCGGGCACCCACAGCCGGTCGGCCGCCGCCACCAGCGAGCCGCAGCCCCACTCCGACGCGTTCCATTGGATGCGCGGGTCGGACCATGTCATGAGCATGTCGGCAAGCATGTGCACGCTGGAGTCTGCCGTGATCGCGGCGTGGCGCGGCTCGAAGGTGAGGTGCACCACGGCGGGCTGCGCCGGCGCCGCCTCGCGCTCGTACTCCGCCAGGAGCAGGTCTAGCTGCTGGGACAGCGACGTCACGTTCGTGCATCCCTCCACCAGCCCTAGGGCCAGAAGGAGCAGGGTGGTGGACAGCACGGAAGCTGGCGACATGGCGGACAGTGGCGCACTGGACCGACTCGCGACTGTACTCGCCTGCTCCAGGGGTACACAACTCTAGGTAGGTTACTTTTTAACATGTTGTTAAATTTTTCAGTCGTTGATG

>IR75p [Moltype=mRNA] [Organism=Loxostege sticticalis], complete cds

ATGACCAAGTAAAATTGGTAACTAAATTATCCAGCGCTGGTATAAGATCCTCAAAATCATTGGATTATGCTCCAGATCATATTCTTCACAATTTACTGTTTCTTATTGATCTACAGTGTCCAAACAGTACCGAAGTACTTTTAGATGCAACAGCAAAAAAACTTTTCAGCTCTCCTTTTCGATGGTTGGTGCTCTCAGATTCAAATAATACCGACCAAGAATCTCTGGATCTGCTTTTCAAGTGTCCTATACTAACAGACAGTGATTTGGTATTAGCTGAAAGAAATGATTACGGGTTTAAAATGTTTGAATTACATAAACCGTCTTTGAATTGGTCAATGATATCAACACCGAGAGGATTCTTCAACGGTACTTTTTACGATACTAGAACTCATAGAGAGCATTTCCGAAGACGAAGGAATGTTATGGGACACCCACTGACCATGTCGAATGTTATTCAAGACAGCAACACCACGCAGTATCATCTGCCTTTAGAAAATAGACAAGAACTCCAATTCGACACAATCGCGAAGATTTGCTGGATGGACGTGAAGTTAGTATTCCAGATGCTGAACGCCACTCCTCGGTACATCTTCAGCCACCGATGGGGGTACAAGCAGAATGGGCAGTGGTCTGGTATGATCAACGACCTACACACTGGAAGAGCTGACTTGGGTACCAACTGCCTAATAAACAACAAGGAGCGCCTAGACGTGGTGACCTACACAGACATGGTGGCTCCTTTCCGAGTCCGCTTCGTGTTCCGCCAGCCCCCTCTCTCCTACGTGTCCAATATCTTCTCTCTCCCTTTCTCCACCAGCGTGTGGGTGGCCATCTTTGTAATTTCCGTTGTCTCCACCGCCAGTTTGTATTTGGCTAGCAAGTGGGAGGCTAGTCTTGGGACGAGCCCAACGCAACTGGACGGAAGTGTGGGCGATGCTCTGTTATTGACAATGAGCGCTGTCAGCCAACAGGGTTGCTTTTTTTGAGCCAATAAAGTTTTCAGGTCGTGTGATGACGTGGGTGTTCTTTGCGGCGCTCATGCTGCTGTACGCGGCGTACTCGGCCAACATCGTGGTACTGCTGCAGGCGCCCTCCAACTCCATCCGCACGCTGGCGCAGCTGGCCGCCTCCACGGTCACGCTGGCCGCCAACGACGTCGACTACAACCACTTTGTTTTTAGTCTATACAAAGACCCAGACCGAGTAAGAATTTACAAGAGAGTTGATCCCGAGAAGGGGAAAGGCAAGGGGCAGTTTTACGACATCAATGAAGGTGTTGAAAGAATAAGACAGGGTCTCTTCGCGTTCCACTCCATAGTGGAGCCAGTCTACCGCCGCATCGAGGAGACGTTCCTGGAGATGGAGAAATGCGACCTGGCCGAAGTTGACTTCATGAACGGGTTCGACCCCTTCATACCCGTGAAGAAGGACTCACCATATTTGGAGCTGATGAGAGTTGCTTTCAAGCAGATCCGCGAGGCGGGCGTGCAGTCGGCGCTGGTGCGGCGCGTGCACGTGCCCAAGCCGCGCTGCGCCAGCGAGGTGTCCGCCTTCAGCAGCGTCGGCGTGCGCGACCTCAAGCCGGTGTTGCTGTTCATGCTGTATGGGATTGCTGCGTCCGTTGCCATTGCTGTCATTGAGATACTGATATTTAAATTGCACAGGCAAAGGCGATTTCAGTTAAGAAGATAAATCTAATAGTTGCTAAAATATCTGTAATTTAGAGAGTAGGCAGTGGTAGATTTAATATGTATAAAAGTACAAAGTTAATAACTTACTTAATTGATGTTATGTGTTAGATATTAGAAATAAAACTTTGGATTCGCAGTTTTA

>IR75p.1 [Moltype=mRNA] [Organism=Loxostege sticticalis], complete cds

CACACCTGGGGGTATCGTGACAAGAACGGTAACTGGCAGGGCATAGTCGACAACCTTATCAAGAAGAAAGCTGATCTTGGTACCCTGACAATCTTCACTCAGGAGCGCATGCAAGTGGTGGACTACATCGCAATGGTGGGCTCCACTGCGGTCCGCTTCGTCTTCCGCGAACCTCCTCTCTCATACATCTCCAATATCTTCACTCTGCCGTTCACTGGGGCCGTCTGGCTGGCCATAGTCGTCTGCGTCCTTGGCTGCTCCGTGTTCCTGTACATCACTTCCAAATGGGAGGCTACTATGAGCATGCATCAATTCCAACTGGATGGCTCCTGGGCTGATGTGATCATTCTAATCATCGGTGCTGTCCTTCAACAAGGCTGTACACTGGAACCAAGATACGCCGCAGGAAGAAGCGTTACCCTCCTACTATTCCTGGCGCTGACGATTCTGTACGCCGCGTATTCAGCCAACATCGTCGTGCTGCTGAGGGCCCCCAGCTCCTCAGTGAGGACGTTACCTGATCTGCTGAACTCTCCACTCAAGCTTGGAGCTAGCGACTTCGAATACAATAGATATTTCTTTAAGAAACTGAAAGATCCTATACGCAAATCGATTTACGACAAGAAAATTGCTCCAAAAGGGAAGAAACCGAACTACTACTCGATGGAAGAAGGAGTGGAGAAGATCAGAAAAGGTTTGTTCGCTTTCCACATGGAGCTGAACCCTGGCTATCGCCTGATCCAGGAGACGTACCACGAGGATGAGAAGTGCGACCTGGTGGAGATCGACTACATCAACGAGATCGACCCGTGGTTGCCGGGGCAGAAGCGCTCGCCTTTCAAGGACCTCTTCAAAATCAATTTCATCAAAATCCGCGAATCTGGTATCCAAGCATGCATCCACCACCGTCTCCACGTGCCAAAGCCGAAGTGCTCCGGCACCGTTTCCACCTTCAGCAGCGTGGGCATAACGGACATGTATCCTGCGATGCTGGCCACCCTGTACGGCATGTTGCTGGCGCCAGCTGTGCTGCTGCTGGAGATTGCTTACCATCGTTTAACGGTGCTACGGAAAAGGAAAATAAAGACAAGGAAGTGGAAATCAAAACTAGACCATTTCTAATCAATTTTGTAGGGTTCCGTACCCAAAGGGTGCGCTGTTTGTCAGTCTGTCAAACAGCGGGCTGTATCTTATGAACCGTAATAAGTGGACAGTTGAAATTTTCACAGAATGTGTTATTCTGTTGCCACTATAGTAATATACAGATAAAAGTGAAATAAAGGTCATCATTGAAGGGGTTACCCATACAAATTCCTTTTTTTGCTCGATCTTATGTGGATTAATTTAAAAGTTGCTACGGAACCTTTCGTGTGCGGGCCTGACTCGCACTTGCCCGGGTTTTTAACAGCGATTACCCTTTACAAAAACAACTTTTATGAAGGGTTTCTGTATGACTCAAGCAAGTTACAAGAATGCCAATTAAATTGCTTGCAATGATCCATAATTAGTGCGTTTACCTAAACAAACACCTGGCACCTGGCCCTGTGGTTCCCATTCCCTAACGCGGTGTTCAAGGACCAGAGGGAACATTCGTCCGACCTCAGGCAGAAGGTTTGACAGCCGGCTAGTGCTGCCGAGACTGTCGATAGTTTGACGATCGATAACTGGGTAACTTGGTGGTGCTGGTGTCGATAGGGGTGATTTTGGCTGTTTAAAGATTACTATTTATTGCTTCATTTTTAACTGAGTTGTATTTTGACACGTTAAATGTTAGAGAATTAAAATCTCTTTTCTTAGTTAAAAAAATCAACGTTTCATAAAAACATTTTAT

>IR75q.2 [Moltype=mRNA] [Organism=Loxostege sticticalis], complete cds

GCAAGTACACTTTTCAACACGATGAAGGTTTCCATTACTTTGGTCTCCTTATTTTTTTTAAATATCTGCAATGCAAAGACGGATTCTGTAGTGCTCGTTGTTGCCGACGTCATTCGAGCCATGGAGAAGCCTTCAACCGTTGTAGCTACGCTTTGTTGGCAAACCAATAAAAAGGTGGATTTTTATAATGCAGTTACTTCTTCTAATGATCGATCTCGGGTTGCCACAGCGCGGCTTGTTGATATGAAATACGTAAAGAGAGATTACGGTCAAGATCAACATATCGTGTTTGTGGCGGATCTGTCCTGCCCGAATATCTCTGATTATTTTGTTATGAAAAAGGAAGAACAATATTTCCGATCTCCATTTCGTTGGATTCTCATAAGTCGTAATGAAAATGATGACATAGTTCCGAATGAAATATCTCATATTGATCTCTTGCCTGATTCAGAAGTGATTGTTTTGCGACAAGTTGGCGATGATTCCTATGATCTACATTTTATTTACAAAATAAGTCCTGGCAGCTACTGGCGAACTCAATTTTACGGCACCTGGAATCATGAGAAAAGATTTGTAAAATCCAATCAGCAAATAGTAGAGTCTACGGCTCTGCAAAGATTGGACTTGCTTGGCTATGAGATGAGCATTTGCTATGTTCTTACTGACAAAGACAGCATTAACCATCTTACTGATGAAGTAAACGACCACATAGACACGATCACAAAAGTGAATTTCCCGACTACGAATCACTTGCTGGATTTCTTGAACGCGACGAGGAAATTCATCTTCGCTGATACATGGGGCTATCGTGTTAATGGCTCTTGGAATGGAATGACTGGATACCTCTTAAGAGAGGAGGTTGAGATTGGAGGTTCACCGATGTTCTTCACATCGGAGAGAATATCAGTGGTGGATTACATAGCCAGCCCCACCCCCACTCGTTCCAAGTTTGTGTTCCAGCAGCCAAAATTGTCGTATGAGAACAATTTGTTCCTGCTGTCCTTCCGTACGTCTGTGTGGTACAGCAGCGCTGGTTTGATTTTCCTGTTACTATTGGCGCTTTTCGCTGTGGCTGCGTGGGAGTGGAAGAAGAATGCACATGATATCTACAGGAAAGAGGATTCCGGTACGCTAAGGGCCAGCTTCGACGATGTAATAATCTTGATATTTGGCGCCATCTGCCAACAAGGAAGCCCTGTTGAATTGAAAGGTTCCTTAGGTCGTGTGGTGATGCTGATTCTCTTCTTGGCTCTCATGTTCCTCTACACCTCGTATTCTGCCAACATAGTCGCGCTGCTGCAGTCCAGCTCTTCGCAAATAAGGACTTTAGATGATCTCCTACATTCGAGATTAAAGTTTGGCGTCCATGATACCGTTTTCAATAGATATTATTTCTCAACAGCAACAGAGCCTGTCAGAAAGGCTATATACGAAAAGAAAGTTGCTCCGCCTGGCACTACCCCTCGATTTATGTCTATGGAAGAAGGCGTGAAGAAAATGAGAAAGGGCTTATTCGCATTCCACATGGAAACGGGAGTCGGTTACAAGTTTGTAGGAAAATATTTTAACGAAGGTGAAAAATGCGGACTACGCGAGATACAGTACCTTCAAGTGATCGACCCGTGGCTGGCTGTCAGAAAGAATACACCGTATAAGGAGATGTTCAAAATCGGGATGAAACGCATTCAGGAACACGGTCTTCAAGCCAGGGAGAATCGATTGCTGTACGAAAAGCGACCTAAGTGCTCTGGCAGAGAATCCAACTTTGTTTCAGTCAGCATGGTCGATTGCTACCCCGCGCTTTTGATACTGTCATACGGTTGCCTCGTATCCGTGATCTTTTTGGTTTTTGAATTCCTCTTTCATCAAAAGCAAACTATAATCCAGAAGTTAACACATTGCAATAGAGTTAAAACTTCAAGATCTGCGTTTACAAATTAATTTGAATGATCTTATTAGTCAGTTTAGTTCTTGTTGTAACGGCACTTAATGCGTTGCATAAGCGTGTTATTTAAAAATGTATAACGTTTCACTGTCTGCTGTAATTAGAAGAAACCACCCATATGATTAGTGAGAAAACTCGATGCCACTTAAAGCATATGTATCGTATAATTATGCACAGGCTTCACTGCAACCAGTAATTACACGTGCTACCTAGTTAAACACATTAGCAAATAAACACATGACAACAATTAGACTTGAATATAAATGTCGAGCTTTCTGTTTCGCCACCACATGGTGTTAGGTTCACATTAGAACTAATAAAGAAAAGAGATGAATTCTTGTGTTTTGTTTTTTACACTTGTATTGGCTTATTGCGCCTTCGCCATTCCCAAATCCAGCCATGAGATCAATTTGATTGCAGATGTAATCCAATCTTCAGATAAGTTGACTTCGGTTGTGGCCCATGTCTGTTGGAAACCATCCAAGCAAATTCAAATGATGTATCTATTGGGCAACAAGAACAGACCAATGACAGTACGCTTTGTGAATAAAGACTGGGCCAGCATAGTAGAACCACACCACAGAGAACGTGTGCTGATAGTAGTGGATGTAAACTGTCCTACAACACGACAGTTTTTCGAAAAGGCGAATGCTACAAAAAAAGTTCAGCTTTCCGTACCGCTGGATAGTCATCGGCAAGGCAGTTAATGAGAGCATTGCTGTGACTTCGGATTTCAATGACTTGCCTTTATTGCCAGACTCCGATGTTGTTATTGCACAGAAGAATGACAGCGATTCCTATATTTTGTCTATGATTTACAAAGTACACGTAAGTAAAAAATGGAGAGTTGAGCAATTTGGAATATGGACATCTGTTAATGGGCTCAAGAAATTCGAAGCCGTAAAGTCGTCAATATCCACAAGACGGAGGAACTTCAGGAGGCATTCCATTAAGACGGCTATGGTGATTCTTGATAATAGGACAATATCCAATCCGTATGATTTAAGTGACATACTAACGGATACTGTGACAAAGAGTAGCTTTCGACAAACAGATCCAATATACGGATACCTGAACGCTTCTAGAGAATTGATCTACAGCCCAACTTGGGGCTACTATAGAAACGGGAGTTACGGCGGAATGATTGCAGAGATGACGATTGGAGACGCTGATCTTGCAGGAACCGTCCTTATAGCGACCCAGGACCGAATGGAAGTGGTTGACTACCTCTCCTGTCCATCTCCAATATCCATAAAATTCGTATTTCGTGAGCCACCGCTTTCGTACCAGAACAATTTGTTCCTTTTGCCATTCAAAACCACGGTGTGGTACTGTATCGGTGCATTTGTACTGGTCTTAGCCTTTATATTGTACATCAACGCTTTATGGGAAAACAAGAAACTTGAGTCAAGTGAGCAGAATTCCAAAGACCCTACCGCTCTAAAACCTCATGTTGGTGAGATAGCAATACTAGTGATAAGTGCGATATCACAACAAGGTAGCTTTACAGAGCTAAAAGGGACCCTTGGTCGGATTGTTATGTTCATTCTGTTTCTTGCTTTCCTGCTGCTTTATTCTTCTTACTCTGCTAGCATAGTAGCACTGCTGCAGTCCAGCTCGAACCAAATCCGGACTCTGTCAGATTTGCTGAACTCTAAGCTTGAGCTTGGGGTTGAGGATACGCCATATAACCGGTATTTCTTCCCAATTGCGACAGAACCTGTTAGAAAGGCAATCTACCAAACCAAAATAGCTCCCAAAGGAACAAAACCGAATTTCATGAGCCTTGAAGATGGTGTCAAGAAATTGCAAAAGGAACCATTCGCTTTCAACATGAATAAAGGAATAGGCTACAGACTTGTGGAAAGATATTTTCATGAACATGAAAAGTGCGGGCTTCAAGAAATAGCATATCTGTATGCAACAAAAACTTACATTACGTGCAGAAAGAACTCGCCTTTTAAAGAAATATTTAAAATCGGATTATTCAGGATCCAAGAGCATGGTCTTAGCGATCGCGAGAACCGACTGATCTACGCACGGAAGCCGGCATGCCAAGCGCGCGGTGGCAGCTTCGGCTCTGTCAACATGGTGGACTTCCACCCAGTCCTCTTGATGTACCTCTATGGCATTATCTTGGCGTTTTTCTTACTCTTTGTAGAGATACTCGCGCATAGAAAACTGCATCCTCGTAGTACAGTCGGCCATGTTATCACTTTGTAGTAGGAAGGTACTTGTAGATTTTTAACCAAGTATCTTTTACAATACACGGTAGACTTTTCACCATAATGCCTTACTTGTATATTATATATTTATTTCCTTTTCATAAATATGCACACCTATCAGTATTGTAGACAAAATAAACTAATGTAAATTC

>IR76b [Moltype=mRNA] [Organism=Loxostege sticticalis], complete cds

CCGACTTGCTCATCCTCACTTCGTGTCGACATGTCAACCCGTCGACATCGCACTAGGCGACCTCCCTTTGATTTTCCGTCTTTTAACCCCCCAATTATTAAGTCTAAACCGAATCTTGGTTCCGCCAGTCCCGCTAAAATAGCGGCAATTTTTCCATTTTCGCGATATGTTTTCTTTGTCATTCCGCGTATGCTGAATTAAGTTTGCAGTGTCTAAAATGCCATGGCCACTGGAATAGAGCTAATCATATCGTCGATATGCAACGCCACATTTTGCCAACCAGTGTACGACAACCCTTTATTAGAGAAGCAGGCATCATCATCTATCGATCAGTACCGAGATTTGATTAAAGAAATCAATGGAAAACATCTTAAAATTGGGACTTACAATAACCGTCCTATAAGCTGGGTGGAGCGCGGAGAAGATGGCGCTCTCATCGGCCGCGGAGTATCTTTCGTTCTGGTTGACATCCTGCAGAAGAGGTTCAACTTCACTTACGAGGTGGTGGTGCCGGAAAAAAACTTCGAAATAGGCGGGACCAAGCCGGAGGACTCTTTGATAGGCCTTGTGAATAATAGCTTAGTGGACATGGCAGCAGCGTTCATACCGAAATTGACGCGGTTCCACGAGATGGTCAGGTTCTCGTACGACCTGGACGAAGGCGTCTGGGTGATGATGCTGAGCCGACCGAAGGAATCTGCAGCCGGCTCGGGACTTCTGGCTCCGTTTAACAACGCCGTTTGGTACTTGATCCTCGTAGCAGTGTTGTCTTATGGTCCCTGCATCACGCTGCTGACAAAACTACGGTCCAAGTTGGTCCCAGATGGCGAGAAGTACATCCCAATGTCACCAAGCTTCTGGTTTGTTTACGGCGCTTTCATCAAACAGGGGACCAATCTCGCCCCTGAAGCGAACACAACCCGCGTCCTATTTACGACCTGGTGGATCTTCATAATCCTGCTCTCCGCGTTCTACACGGCGAATCTGACCGCCTTCCTGACGCTGTCCAAGTTCACCCTGGACATCGAGAGTCCCCAGGACCTGTTCAAGAAGAACACCAGATGGGTCTCTGCTGAAGGCGGCGCGGTGCAGTACGTCGTTTCTAGTCCGAACGAGGACATCTACTATCTCAGCCGAATGATAGCCACTGGTCGAGCTGAATTCCGTTCAATGAATAGTCTCTACGAATTTCTTCCACTGGTATCCGGAGGTGCAGTCCTAGTTGAAGAACGAATCGGCATCGACGAGCTCATGTATGGTGACTACCAACAAAAGGCACGGGAAGGCGTGGCGGAGGCTGAAAGGTGCACTTACGTGGTAGCACCCAACTTATTCATGAGCAAGCTGCGGGGATTCGCGTACCCTAAGAATAGCCAGCTCGCCCCGCTCTTTGATACCGTCTTGACGTACGTGCTGCAAGCTGGCATAGTAGATTACCTAGAGCACCGCGACTTGCCCAGCACGAAGATATGCCCGCTCGACCTCCAATCCAAGGACCGGCAGCTCCTGAACAGCGATCTCTACATGACCTACATGATCATGGTCACTGGCCTTTCTGCTGCTGTCGCCGTCTTTATTGGAGAGATCATGATAAAACGCTACGTAATCAAAGACTCCAAACCCAAGAAGCCTAAGCGCAAGAAGACTAAATACGAGAAGAACCGTCATATTAACAGCTACGATGACACCCGGCCTCCGCCATACGATTCCATCTTCGGACGGAGCCCCAAGATCAAAGTCAATGATACCACAAAGACGAAGATCATCAACGGACGTGAGTATTTGGTGATCGACGCAGCCAACGGAGACACCAGGCTTATTCCACTAAGGACTCCGTCGGCATTTCTTTTTCGCCTAGATAGATAAATCAATAGGGAGTTTGAACACATGAGAATTACTTGTGATTAGATCAATTTATTTTATGTTTAAAGTAATCGATAATTCCTAGTAAATTAATAGTACAAATTGCATAACATAGATATAAAATGTAAGGATAAAGTATTTTTGAGGTGATGTTTTATGACTTAAGGATTTATCGATATGATTAATAAAGGTACCTACTATTTACTTACATGGTTTTGGAGTTGCAATTTTTATGTAGAATTTTGAAGACAAAATGTTCACAAATTCACTATGCACAAGGGAGCAGAATATATTTTAATAAATGGTTTTTGTGTTACTGGTTACTTGGTT

>IR87a [Moltype=mRNA] [Organism=Loxostege sticticalis], complete cds

CACTACTGACGACAACCGATTCACTTTGGAGCTGGCGGAGCCATAATGTGCACTAAAATATTTCTACAACATTTATTTTTTGCACTCTACGTTTCTGCTGCGATAAATGAGAATCCACTACTGACGACAACTGGAAATTCTGAGCAAACAGCAAAAACCGCAGAATGCGTTTTAAAACTCTCTGCAAAATATTTCGTGGAAAAGAAAGCCTTAAGCGGGAGCATTGTGATAATCAACATCAACTCTTATGTCTCCACAACACAAGTGCTACTATTGCAAACAATTCACGGAGGCATCAAGTATTCTGTCATGGTAAAGGATTCTTTTTATCCACACGCGAACGCATCGCATTTTCCTGAGAAAGCTAAAAATTATATGCTGATTTTAGAAGAGAAATCCGAACTAACGAGAAACATACTGCAGCTAAACAAATTGCCCACTTGGAATCCATTAGCCAAAGCCATTGTGTTTTATCAGCTGAATAAAACTGAGGACGCCGAGGAGACAGCCATTGAATTTATAAACGAACTGCGTCATTACAAACTTTTCAAAAGCATTATATTTATATATTCACCCGAGGAAAAAGAAGTTGTTTCTTACACTTGGGTTCCGTATAGTGACATGAACTGTGGAGGAAAATGTGATTCTGTATACATTTTAGACAAATGTAAGGACAATGTTATTTACCAATTAGCTACTCAAAAGGAAATGTTTCCTTCAGATATGAAAGGCTGTCCATTAGTTGCTTATGCAATTGTCTCCGAGCCGTATGTCTTACCGCCTGAAATGAAACTCACTAACACAAGTTACAACGACGCTTATGTGTTTCAAAAAGGAGGGGAAATAAACCTCGTCAAAATAATAACTCAATTTACAAACATGAGCCTCATAATGAGAACATCGGAGTTACCAGAAAATTGGGGAACAATTTACTGGAACGGTACTGCTACTGGAGCGTATGAGGTGCTACGTAATGATTCTGCGGACTTAGTCATAGGCAACATTGAAGTGACAAGAACTATTAGAAGATGGTTTCATCCTACAGTTAGTTACACGCAAGACGAAATGACTTGGTGCGTGCCCAAAGCTGGACAGGCATCTACTTGGAATAATCTGGTGATTATTTTTCAATGGTCCACATGGGTAGCCACTTTTGGCAGTCTTGTGATTATGGGGTTGGTTTTTCACTACATGTATTATAGAGAAAATGGCAAAAAAGTAACAAAATGGCCGACAAATTCTATGCTGATGACTTTCAGCATGCTGTTGGGCTGGGGAGCATCATTCGAGCCAAAAAGCGCCACGTTTCGGATTCTGATATTCGGTTGGCTCTGTTTCAGTGTAAACATGGGGATTTCCTACGAATCATTCCTGCGGAGTTTCCTAATGCACCCGCGCTTCGAAAAGCAGATAAGCACCGAGACGGATCTGATACAGTCAAGAATTCCTTTGGGAGGCAGAGAGATTTACCGATCCTATTTTGAGACTAATAACGCGAGTTCGTTTTATTTGTATCGCAAATATAACTCGACAACGTTCGCTGAGGGGATAAGACGGGCGGCTAAGGACAGGAATTTCGCAGTTGTTTCGTCGAGAAGACAAGCCGCTTACGCCGACCAGAAATTGGGGAAAGGTAAGCCGTTGATCTATTGCTTTCCTGAGAGCAATAACTTGTACAAGTACGGAGTTGTTCTGTTGGCTCGGAAGTGGTTTCCGATGATAGAAAGGTGCAACACCATCATAAGAAGTGTGTCTGAGAACGGTTTGATTGATAAGTGGAATAGGGAGTTGTTGATACATATTGGCAACGGAGAAGGGACAAGTGAGATAGAACCGCTAAGCATTCAGCATTTGTTAGGGGCATTTATTTTTGTAGGGATCATGTATGCTGCGAGCATATTAGTCTTTATTTTCGAGGTGTCTATTGGAGTCTATGAAAAGTGGAAGATAAGGAAAAATTTGCAGCCCGATAATAGACACGTAAGATTTCATTTCGCTAAATAGAATCTAAGGGAATCCTTTGAAACATGAAACCTAAATGTAGTCACCTAGTAACAGTATCTACCCACCTATCGTATTGTTATTATTAATATAAAAGGAATTTTTATCACTGTCTTTTTTCCTGACCCTATAAAAAAAAAATTGCATTTTTTACGCTAAGTAACTTATTACTTCCTTATTAATATCATAGGGAATATTATTCATAATGCGACGGCTGCAGCAGAGATCCTCGGTACGTTCCCCGAGTCGGGCCAAATATGTGTACCTGTTTATTTAATCTGTTCAGAAATTCTCAGGGCAGCCCGGAATTTGGAAGTTGGCGGAGTTTCACCCCCGAGGCACGAAAACCCATTGGTTCTGCGCGTGATCTGTCTCCGGTCATGTCAGATTGCTGTCTCATGCGGGCATATCGCACAAATTATCTAGCCACAAACTGAGCAGTATATTTCTGGCTTAGTGCCGGATAAAGATTAAATATTAGTTAGACATTCTTATTGAAACAGGTTACAAATTCCCAAATCACACCACCGCGCCTACGCAACTACAACACAGGAAAAATAGTAGAAAACTCAAGAGTTTATTTCAATACACAACTCTATTGGCTAGGAGTAACCATTTTAATTGAGGTA

>IR93a [Moltype=mRNA] [Organism=Loxostege sticticalis], complete cds

GTTAAAATGACCCAGCTGTGTTCGCCGACTTATTAGTTGGTGTTTAATCAGACCTTCAAATATTCAGCCACTTTTTGATTGGAACAGTGTGGGTTTTTTTTACGAATTATGACTGAAAGCTCACTTTGGGGAGGCAGGGCTAGTGGTTCGATAGTGCAGACGCAAAATGCGGATATGGTTGTTGGTTTTCTGTATAGTGCAAGTGTCGGGGGAAGATTTTCCGTCGCTCATAACAGCAAACGCGTCTATAGCGGTAGTGCTAGACCGAGAGTTTCTCGGCGAGCAGTACCAGGCCATCCTGGACGAGCTGAAGGACTACATCAAGGAGTTGGCGAGGGTTGAGCTCAAGCACGGAGGCGTCGTCGTACATTACTATTCGTGGACCTCCATCAGTCTAATGAAAGGTTTCTTAGCCGTCTTCAGCGTCGCATCGTGCGAGGACACGTGGTCGCTGTTCTCCCGAACTGAAAAAGAGGAACTGCTCCTGTTCGCCCTCACAGAAGTCGACTGTCCCCGGTTGCCGACAGACTCGGCCATCACCGTCACCAATGTGGTGCCTGGCGAAGAACTGCCCCAAATTCTCCTGGACATGAGGACCGAGATGGCTTTCAAGTGGAAGTCGGCAGTGATCCTGCACGATGACACGTTAAGTCGTGACATAGTATCCCGTGTGGTGCAGTCCTTGATAATGCAGATTGACAAAGGAGCCTCGACGTCTCCCGTCTCAGTGGTGGTCTACAAGATGAAGCACGAAATAAACGAGTACTTGAGGCGGAAAGAGATACGTCGAGTTCTGTCCAAGTTGCCAGTAAAACACATTGGCGAAAACTTCATGGCAATAGTAACGACTGAGGTGATGACCACGATGTCAGAGATAGCGCGGGACCTGGTCATGACCAACACTCACGCGCAGTGGCTGTACATCATATCGGAGACGGATGCCCAAAACGGGAACCTGTCAAGTCTCATCAACGCCCTGTATGAGGGGGAGAACGTTGCGTTCATTTACAATGTCACTGATAACGGCCCTGAATGTAAGAACGGGTTGATGTGCTACTGCCAGGAGATGATGAACGCGTTCATATCAGCGTTGGATGCGGCCGTGCAGGATGAGTTCGACGTAGCAGCACAGGTGTCTGACGAGGAGTGGGAGGTCATCCGGCCCAATAAGGTCCAGAGGCGGAGTATGCTGCTCAAACATATGCAGCAACACATAGCCACTAAAAGCAGCTGCGGCAACTGCAGCACCTGGCGAGCCCTGGCGGCGGACACCTGGGGAGCCACCTACCGCTCGTACGGAGACGCCGACCTGGTCGCCAAGGACACCACCAATGGCACGATGACGGGGGCTATAGAGCATGTTGACCTGCTACAGGTCGGTTATTGGCGGCCCATAGATGCGTTGCGGCTTGATGACGTCTTGTTTCCTCACGTAGAACATGGGTTCAGAGGGAAGGATTTACCCATTATTACTTATCATAACCCTCCATGGACCATCCTACAAGTGAACGAGTCGGGCTCGGTGGTCAGCTACACTGGTCTGATCTTCGACATCGTCAACCAGCTCGCTAAAAACAAGAATTTCACAGTCAAAGTGATCCTTCCGAGTCACGTGAAACATCTTGTGGCCAATGATACATCGGCTGATATGTCTCACAGTCAAGATGCAATGCTCACGTTATCAGCAGTCGCAAAGGGACAGGTGGCTATTGCCGCGGTGGCGTTCACTGTTCTCTCGGATCCTCCACCTGGTATCAACTACACAGTGGCTGTGAGCACCCAGACTTACTGCTTCATGATAGCGCGGCCGAGGGAACTTAGCAGGGCTCTGTTATTCCTATTGCCTTTCACTACTGATACCTGGCTCTGTCTTGGTCTCGGCGTGATCCTGATGGGTCCTACGCTGTACATCATCCACCGCCTGAGCCCATACTACGAAGCGATGGAGATCACTCGCCAGGGTGGCCTCTCCACCATTCACAACTGCTTGTGGTACGTCTACGGAGCGTTGTTGCAGCAAGGTGGCATGTACCTACCTAGAGCCGATAGTGGTCGCCTCGTGGTCGGCACGTGGTGGCTGGTGGTCCTGGTCGTGGTCACCACCTACTCTGGGAACCTCGTGGCCTTCCTCACGTTCCCTAAGCAGGAGATCCCAGTGACCACAATAGAAGAGCTACTGAAAAACCAACAGATTTATACGTGGTCGATCCAGAAGGGCTCGTATTTGGAGTTAGAATTGAAGAATTCAGACGAGCCGAAGTACACAGCGTTGCTAAAAGGCGCCGAGCTGAGCAACACCGGCGGGACCATGGAGAGCAATTTATCATCATGGAAGAAACAGCTGATCCGAATCCGCGAGCAGAGGCACGTCATCTTCGACTGGAAGCTGCGGCTGAGCTACCTGATGCGCAACGAGCACAAGCTCACGGACAGATGCGACTTCTCGCTCAGCGTGGACGAGTTCATAGACGAGCAGCTGGCTATGGTCTTGCCAGCGGGAAGCCCGTACCTGCCTGTCATCAATAAGGAGATAAATCGTATGCAAAAGGCAGGGCTCATCTCGAAGTGGCTGTACGCGTACCTGCCCAAGCGCGACCGCTGTTGGAAGACCTCCAGCATATCCCAGGAGGTGAACAACCACACCGTCAACCTCAGCGACATGCAAGGCTCATTCTTTGTGCTGTTCCTTGGTTTCTTCTCTGCATCATTCGTGCTATTTTTGGAGTGGTTCTGCAATAGACGCAAGAGACGAAGCGAGGAAGTCATCATCAAGCCTTATGTAGAATAAATAAAAGCTTTAAACATCAA

>GR1 [Moltype=mRNA] [Organism=Loxostege sticticalis], complete cds

GGACGTTGTAACTGAAGACTTTGCTTTGTACAACAGGATAAAATGGGTCAGCTGAAGAAGAATTTGGGCTTCTGGATACCTATTAAAAAAAACAAAGTACACGTAACAAAGTTCCAGACAGACGGAAAGCAGGCAACATTCCAGAAATCGTTACGGATGACGTTGATAATAGGACAAATGTTTTCATTGATACCAGTGACTGGAATTTTCAGTAACAGTGCTTCAAATGTAAGATTTGTTTTAAAATCATGGAAATGCTTGTATTCAACCCTATCTTTTTGTGGTCAAATTTTTATGACAGTAATGTGTGTCCACAAAGTTGTTCATACAACTACTTCACTAAATGGTAACGCACCAGTGATATTCTACGGGACGACCTGCATCACGATGATAATGTTCTTCCAAGTTGGCCGTTCGTGGCCTAGTCTGGTCAGACATATTGCGAGGAATGAAGAACTTGATCCGAACTTCGATCCAGGTTTATCGTATAAGTGTAACGTCACTTGTGCGATTGTGTTGATGCTGGCTTTGCTGGAACATATTTTATCACTGCTGTCAGCATTTGCCGGAGCTATGGTGTGTCATCCGGATAAAGCATTCTATGAAGGTTTCGTAACACATTTCTACCCGTGGGTCTTCAATGTGCTTCCTTACTCCGCAGTTTTGGGTGCTACGACACAGTTTCTACACTTCCAGTCGACGTTTATATGGAACTTCTCTGACTTATTTGTGATATGCATGAGTTACTACCTGACCTCTCGACTTGAACATATCAATGGGAAGCTATTGGCTGCGCAAGGAAAGTACTTACCGGAGATCTTCTGGAAGACGACTCGCGAGGACTACTGCCGCGCCACGCAACTCGTGCGCAGGGTCGACGAGGTCATCAGCGGGATCGTGTTCATCTCCTTCGCTAACAACTTGTTCTTCATCTGTCTTCAGCTTTTCAATACTTTGGAAGATGGAATCAAAGGCACTGGGGAATGTAGCAGTCGCTCGAAATCAACGCCTTCAAATCTTCTGGGAGGATACGAAGCAGCAACTTACTTTTTGTTTTCACTAGTGTACCTCATCTCACGGTCTGTTGCTGTCTCACTGATAGCGTCGCAAGTCAATGCAGCTTCTACTGTACCGGCTCCTGTGCTGTATGATGTGCCTTCACCAGTTTACTGTGTTGAGGTTCAAAGATTCCTGGATCAAGTCAACGGTGAGCACGTCGCATTGAGTGGTCTTCAATTCTTCAGTGTCACAAAAGGGCTACTTCTTACCGTAGCCGGCACGATAGTGACGTATGAACTAGTGATGTTCCAATTCACAACGTCACAGCCCGACGATGTTTCAACAAATACTGGTTTTAATACAACTGGTGCTTTTTCTAACATATCATCTACAATAAGTTATTTTATACAATAATTACGTTTTGAGTTTAAGTTGTTTTGTAATGACGTTTTGTAAAAATTGTTTCTTAGTGTGCAATTAGGAGAGTGATCTATGTTTTTTTAAGAAAACAGC

>GR4 [Moltype=mRNA] [Organism=Loxostege sticticalis], partial cds

TTGTTGCATGAGGTTATGATTATTTCGTTTGACGCAGTGTTGGACTATCATAACAGAGTCCGGTAGTGTTGTCTATGGTCGCGTCGCATCGCTACGTGATTTCTGCTAGCCTAGTACTTTTATAATTTTAATCGAATTTATGTTCTGTGTTTTAAATACCGTAAAAGCTTTTTTTATGAATATCACTTTCATTTTAATTGTACGATGTGACTTTAATTTATTTTAAGTGAAAATAAAATGTGTGCGTTTTTATAAAATGGAATTTGTGATTTAAATTCAAACACATGGGTGTTGAAACGTCAAACGAAGAGAGAGGCGACGTCACCCCGGCGCCGACGCTGCGCTCTACTGAGCCTACGCGCAGCGTGGTGGGGGGTGCGCACGCGTTCATACTCAGGATATCAAGTTTCTTCGGACTAGCCCCTCTGCGGTTCGAGTCTCGTGCCAATGGGTTCACAGTATCAATATCCAGTGTCATGTGCATCTATAGCTTTATTCTAGTCAGTATTTTAATATTACTAACAATCTACGGTCTAGTAGCCGAGATCAATGCGGGCGTGAAGCTCTCCGTGAGGATGTCTTCGAGGATGTCGCAGGTGGTGTCGACGTGTGACGTGCTGGTGGTGGTTGTGACGGCTGCAGTAGGGGTGTACGGCGCGCCTGCCAGGATGAGGAAGATGCTGAAGCTTATGGACAGGATTGCTTCGGTGGACAATACCCTCAGCGGCCAGTACTCCGCGGTGATGGAACGCAAGCTGTCCGCAATATTGCTGGCGCTACTCATCTTCTTCTCGCTTTTGATTGTGGATGACTTCTGCTTCTACGCGATGCAGGCTAAGAAAGTGGATAGAGAATGGGAAATAGTTATGAACTACATCGGGTTCTACCTGCTGTGGTACGTGGTGATGATCCTGGAGCTGCAGTTCGCGTTCACGGCGCTGTCGGTGCGCGCGCGCTTCCGGGCGCTCAACGATGTGCTTGCGCTGACGGCGCGCAGCATTGCTGTTCCAGTGGAAAAAGCCAGAAAGCCCACACCCTTGAACATCTTCGCAATCCGAGTAACGCCGACAGACCTGCAGAGATCAGATGACATCAGTTTGCTGATGACCTCTACTCCTAAGAAACGTGAAACAGTTATTGTTAGGAGGAGTGTGAGTGGAGAGTCTCGCCTCCTCGTGTCTCCGAGTGAAGCGATCTATGGACTGGCATCTCTTCACGGAACCCTATGTGAGCTGGTCCACAGGATCGACGACAGCTACGGCATCCCTCTCGTCGTGATCCTCATATCCACGCTGCTGCATCTGATAGTCACGCCATACTTCCTTATTGTGGAGATTATTGTATCAGCCCATCGAGTGCACTTCCTAGTTCTTCAGTTCCTGTGGTGCGCTACTCATCTGCTCAGGATGTTTGTGGTGGTGGAACCATCACACTACACAATCATGGAGGGTAAAAGAACTGAAGGTCTGGTCTGCAGGCTGATGACGTCAGGACCATCGGCGGGCCCTCTGCCATCAAGGTTGGAGCTGTTCTCTCGGCAGCTGATGCTGCGCTCTGTGTCCTACTCTCCCATGGGCATTTGCA

>GR5a [Moltype=mRNA] [Organism=Loxostege sticticalis], partial cds

GGTGGGAGAGTTTGTTTTTTGGCCGACTGTTAAGAAGGGCTTATTGTGTGTGTTAGTTTTCTCGTATTATCCAAAAGGCTGGATTGATTTGAAAAAGTATATAACTAAGATTGTCCGATTTTAACCCTAATGTTTTTAGATGGGTGACAGCAAATAAATAAATAGCCATAAATGTGTTGAAAGCCAATCGTGTGAGTGATAATAAACATATTTGTATGGATGTTATGATGAATTGCAAAATTGTTACAGGGTTGTAACCAATTGTAATCTGTGAATTTTACAAATTCTATGATAAGGATGTAGAAGACAGTAGCTGTAGTCTGATTTTCTACAATTTCAACCGAGTTCTTTATTCAGCCGTGCCGACAGAGATGTTCTACGTCTAACCGAACGCGAAAAAGTGCGATATTCGCAAATTCACACCAAAATTGCACCTGGAATTATGATGCAGTTACAGATTTCCATGTCTAAACTGGAACTGTGGTGAATGACTGTGACTTTTGCCGGAAGAAACCAAATATTGAAACATGTTGATTCATTGTTTTAAATGGGAAGTTCACGTGTGCATTTCACTGGTGAAGATAAAATGTCCGACAGTAAAACTGTTGGTGCTCAGAATAAACTGATTTTGCCGGATCAACCTTATCATGATGGATTCCTCGAAACTATGTCGAAGACATTTCACTGGGCTCGTTTATTCGGAATCATGGGACGAGAATCTCGGAGATGGAACGTATGGGCTATAGTACTCCTGATCACTTTGCTGGTCATAGAGGTAGCTGCTATCTGGAAGGTGATTAAAGCACTAGCCGGATGGGCCGTAGACACTGCTGCACATCGTAGTGTAACAGCTAGACTTTCAGGCACGTTGTTCTACACAACTGTGATTGCATCACAGATATTATGCTCGCGATTGTCATTGAATTGGCACAACCTATCTTCGTATTGGGTGTCGGTGGAGAGAGCAGTTGCTATAAACATACCAACAGACCAGACGATGAGAAAAAGGATGCTCACTGTTATCATCACAATGGCGATTCTAGCTACGGTTGAACACTTGATGAGTGTAGTGGCATTAATAGGCTTTGATTGCCCGCCACATTTGATATTAAGGCGGTATACGGCCAGATCGCATGGATTTCTGTTTTTAAGAGATGACTATTCAGTATGGTTCGCGATTCCTTTGATCTTCATCAGTAACATTGCGACTATACTCTGGAACTTTCAAGACGCCTTGGTAGTTTTGATTTGTATGGGTCTCACTTCGAGGTATCGCAGACTTAATAATTACGTCTCCAAAATTTGCGAAGAAGAAAAGAAACTAGCCAATAAAAATATGAAAGCCGAAGCCGTTCGAATATACTCTTGGCGAAGGATTAGAGAAGCGTATGTGAAGCAAGCTGCCCTAGTCCGGAAGTTGGATGGAGCGCTCGGGGGAATCATTCTTCTCTCATCTTTCGGCAATTTCTACTTCATTTGCTTGCAACTGTTTTTAGGGATAACGCAAGGTCTGAGCAACACTTCGACGATCAAGCAGATATATTACATAGTGTCTCTCCTGTGGATACTCGGAAGGTTTACTTCGATGGTACTAGCAGCTGCTGATGTCCATGTTCATTCTAAGAAAGCATTGCCAGATCTACATGCTTGCCATTCGAGATGCTATAATGTTGAGATAGACAGGCTTCTGAACCAGTTGAATAAAGATTACGTTGTATTGACTGGAGTGGGATTTTTCACCATTGACAGAAACATTCTC

>GR5b [Moltype=mRNA] [Organism=Loxostege sticticalis], complete cds

CAAATACCTACTCCTTATGGTCAGCCATACTGATTTTCTTCGTGAGCAAGATAGCAACAATCCTTTGGAACTTTCAAGACCTTATAATAATCTTGATAAGCATGGGGCTGACGTCGCGGTACCACAGATTGAATTCATTTGTAAATCTGTGTGTGAAGAATGAAAAAATCAATAGGGATAAAACGAGTGTAACAGAAAAATACGTGCGCACTCACCAATGGCGTCGGATTCGCGAGGCGTACGTGCGCCAGGCGGCACTCGTGCGCATGGTCGACGCGAACATCGGCGCGCTGGTGCTACTATCCAATGTCAACAATTTTTACTTCATCTGCCTTCAATTGTTCTTGGGCTTAACCAAATCCCAAGGATCCCTTGTAAGCTACCTATACTACTTCATCTCTCTCGGCTGGCTACTCTTCAGGGCGTGTAGCGTCGTCTTAGCGGCTGCAGATGTACATATCCATTCGAGACGGGCCCTGGAGTACCTTCAGACATGTCCTGGCACTGGGTTTAATATTGAGATAATGAGACTCAACAACCAGCTAAGCCACGACTTTGTGGCGTTGAGTGGGATGGGATTTTTCTCGTTGAGCAGACAAACGTTATTAGAGGTGGCCGGGAACATAATAAAATATGAGCTCGTGCTGATTCAGTATGACAAATGATCTGAAGGAAGGCAACTGAAAACATAGCTTCCAATATTCGCACATGACTGCCGCGTTCGCGTTTCTAGGGGGTGACTGCCATCTCTCAAAACAGTATTAAAAACAAGCTTCTTGTGTTTAACGCTGTCAATTAGTACTTAATTTTGAC

>GR6 [Moltype=mRNA] [Organism=Loxostege sticticalis], partial cds

CGTAGATGTAGTCTCATGTCAGTATCTCTACAAAAGCGTAGCAGATATGGTTGAGAAAGTGAAGAAGTCGTTTGATGTTGTGCTCATCTTAACTCTTCTGGCTAACACCACGGATGTTATTATTCATGTTTACCTCCCGTTTGCAAAAGAACCATTTAAATCGATCGTCGGATATGACTTGACCTTGGCGTATGTGGTGGTGGTTCAGCAGCTGCTGATACTGTTCTTTCCTGCGCTCACGGCGGGCATGCTGACGGGCCAGGTGGAGAAGCTGAAGCTCGTGCTATGTGACATGTTGATAAAAGACAAATCTCGCAAGAAGGACATCAAACGGC

>GR7 [Moltype=mRNA] [Organism=Loxostege sticticalis], partial cds

GTTTGTTTTCATTTTAGTTGTGTAAAGAAGTGCTGCGGTGTGGTAAATTGTCGTCAAAATAGTCAAATACATAATTATGAGTGAGACGCCGGATTTGTCAAAATTGGAGTCCCGACAGCAAGACGTAAGAAATTTCTTCCCACTCTCACACATATCTTCACTGTAGCCCAGTGGTTTGGTATACCTTCATATGGGAACAAATTTGCAATTTGTTGGGCAATCATTGTTTTATGTATGTTGACGGTCGTCGAAGGCGCTGCTATTTGGATGATGATAAGGCTTTTGGCTGGTATCGCTAAGCATATAGACGATGGACGTGGTCTAACAGCAAGACTTTCTGGGAGCATATTCTATGCGAACGGATTTCTATCCCTTATTCTGTCCTGGAAGTTCATGTACTCGTGGAAAAGGCTCTCCTTCTATTGGAAGAGAGCTGAGTTAGTAGACGTGTCTTTAGCGATTCCTGATGAAGCTATACAGAGAAAGGTCATCGTTGTGACTTGCTTTGTCTCAGTGTGTGCTTTTGCAGAACATTTATTAAGTATGATATTGGCTATTGGTATAGATAGCCCTCCAATGGATTTTTTAGAGAGGTATATTCTAAATTCTCATGCGTTCCTCATAACACCAAATACCTACTCTTTATGGTCAGC

>GR21a [Moltype=mRNA] [Organism=Loxostege sticticalis], complete cds

GGCAAAACGCTCACCTCCACGAAGACAACGATAATCGAACATGGACGAGGAAAAACAAATGTTCCGGATATACAACACAAACCAGATTAACGGGAAACAAAAAAATACTAACGGAATAAGAGAAGAATATGACGCGAAGGATATATACGGTCCTGAGATCACGGACAAAGATGGGGCGTTATTGGATGAGCATGACAGTTTCTATCACACCACCAAGAGTCTACTAGTGCTATTCCAGATCATGGGCGTTATGCCTATCATGAGGGTACCAAAAGATGCTCAAACCACAAATAGAACTACTTTCAACTGGATATCCAAGGCAACTTTATGGGCGTATCTTGTGTGGAGCCTCGAATGTATCATCGTTGTAAGGGTGGGCAAAGAGCGCTTAGCAACCTTCCAGCAGAACACGAACAAACGTTTCGACGAAGTGATCTACAACATAATCTTCTTGAGCATTCTGATACCGCACTTCCTCCTACCTGTTGCTTCGTGGCGGCACGGGCCGCAGGTGGCGATATTTAAGAACATGTGGACCCATTATCAGCTTAAATACCGAAAAATAACCGGGACGCCCATAGTGTTTCCAAACCTGTACATCTTGACGTGGGGGCTGTGCGTGTTCTCCTGGGGCCTCAGCTTCGCAGTGATATTGTCACAACACTACCTGCAAGAGGATTTCGAATTGTGGCATTCGTTCGCCTACTACCACATCATTGCTATGCTTGACGGCTTCTGCTCTTTATGGTACATAAATTGTAACGCGTTCGGGACGGCTTCCCGGGGTCTAGCCATGAACCTGCACAAAGCTCTGAAGGCAGAACACCCGGCCTTGAAACTGGCGCAGTACCGCCATCTGTGGGTCGACTTGTCGCATATGATGCAGCAACTTGGTCGTGCATACTCCAACATGTACGGAATATACTGCATGGTCATCTTCTTTACCACCACCATATCCCTCTATGGAGCCCTATCGGAGATACTGGAGCGAGGTCTAAGCTACAAAGAGATGGGGCTATTCGTAATAGTCGGGTACTGTATGACCCTGCTCTACATTATATGCAATGAGGCATATCACGCTACGAGAAAGGTGGGGTTCGAATTCCAAGTTCGCCTTCTGAACGTGAACCTCGGTGCCATAGACCGCAGCACCCAGCGAGAAGTGGAAATGTTCCTGGTGGCTATCGCGAAGAACCCTCCTATCATGAACCTTGATGGGTTTACTAATATCAACAGGGAGCTTTTTGCTGCTAACATCTCCTTCATGTCAACATACCTCATCGTCTTGATGCAGTTCAAACTCACTTTGTTGCGACAAAGTGCAAGGAAAGCTATCAAGACCGTGGTCAAGGCTATTTTCAATACCACCACTTTGGGTCCTGATGATGAAGATGATGACGTGGAAGAAGAATGAACAAAGACGAATAGGGAAAACCAAAAGATTATTATTTGTAATTTGTTTCCACATTATGAATATCCAATACTAACTACGATTTGGTCCTTAGCCTAGGGTCTATAGAATGTTTTATTTTTGGTGCTTTAATTTACATTTTAAGTATAATTAATTGATTGTAATAAATACATAAGTAC

>GR21b [Moltype=mRNA] [Organism=Loxostege sticticalis], complete cds

GGGATTATGAGCAAGAACAGCGGGATCTGCTCTCGTCTCAAGATGGCGACACTTGCGAGATCCACGACCAGTTCTACCGGGACCACAAGCTGCTGCTCGTATTGTTCCGCGCCCTCGCTGTCATGCCGATCACCAGGTCTAGACCAGGTACAATCACCTTCAGCTGGCGGTCTTCAGCCACGGCCTACGCTATCTGTTTCTACATTGCCAGCACCATTGTCGTCTTGTTCGTCGGCTATGAACGAATCTTGATTCTCCGGTCCATCAGGAAGTTCGATGACTACATCTACGGAGTTCTGTTCATTGTGTTTCTTGTACCTCATTTCTGGATTCCTTTCGTCGGATGGGGTGTTGCCCATCAAGTTGCCATTTATAAAACCAATTGGGGAAAGTTCCAAGATGTGGGAATAGATTGCTCGGCCAAATTGATTTCTCGGTACCGCTACTTATGGTTAAATCTATCCGAACTACTGCAACTTCTCGGCAACGCGTATGCGCGAACTTATTCTACTTATTGTCTTTTCATGTTCACCAACATAACAATCGCAGTGTACGGAGCCCTATCAGAGATAGTGGACCACGGCATCGGCTTCAGCTTCAAGGAAATGGGCTTGTTCGTCGACACCGTCTACTGCTCTACGTTGCTGTTCATCTTCGCTGACTGCTCGCACAAATCCACACAAAAGGTAGCTGATGGCGTCCAAGAAACGCTTTTGACTATAGATGTCCTTGCTGTTGACCGTCCTACCCAGAACGAGATTGATCACTTCATTCAAGCTATAGAAATGAACCCCGCTGTAGTAAGTCTAAAGGGTTACGCAAACGTTAACAGGGAACTACTTACCTCGGCAATAAGCATGATAGCCATCTACCTCATCGTGTTGCTACAATTTAAGATTTCATTGCCAAAGGATCCATAGATGCCTGCAACATAAAAAAACAATAATGTTTGTGATATAAAGCCACATAGATTACTATAAAAATACCACATTGTCAGACGTAAAACGTAAATGTCTGTTAGTTACGTCCGTTACGACCTTAAATAGAAACGTCGTTGCTGTATGAATACCTACATAGGTCG

>GR45 [Moltype=mRNA] [Organism=Loxostege sticticalis], complete cds

GCAGTATATCTCAATATAGTAGTTACAGGGCTACCACTAAATCTGATCGGGAACTTGGAATGGGTAAGCGTAGTAAGTTTTGTAGCTACCAACTTATTTGTTGCGCTATTCATGTCCATACGATGCGAAATTTTCTTAAGGGAAGTCGTGGAAACGAAACAATTGTGCATTACAATTTTGTCCATGTATACAGATGGCCCTATCAGAGAAAAGGCCAGAAAGATGCTGAAGTTGGCAGAGGCCAGCCCCCCTCGTTTCTCCGTGTACGGCATGTGGAACATCGAGGGAAGGTTCCTTCTTTACCTATTCAGTATTATCACAGCTGTGATGCTCACGGAACTTCAATTGCTTCTCTTGTAAATTTTGTGTGATGCGCTGTATTTAAAATATTG

>GR51 [Moltype=mRNA] [Organism=Loxostege sticticalis], complete cds

CTAAGGACCATTTTTAAAGCGTTACAAAACACAAACGAAGTTGTGATGGGCAAAGGCATGATAGCGTACGCCTCAAGTCGCTGCTTGAGGTACACGGTGATGGTCATCATACCGTGCTACTACTCCAGCGTGACCACGACCCAAGTGTCGTACATGCGGACAATGTTGCACGACGCTATGAACCAGGTTAACATTGGTAAGGTGGATCGTCGACGAGTAAAGGCTTTTTTCCAGTTGACAAGGGAAAATGAATTTGCGTACGCCATTTGGGGAGTTATAAGACTCAACATGTCATTGCCACTGAGTTATTTGAGTCTCTGCACAACGTATCTTGTCATCATCATCCAGTTTGCAAAGTTTATTGATTAATAAGAATCATGTTGGAAATGGAATAATAAAAAT

>GR63a [Moltype=mRNA] [Organism=Loxostege sticticalis], complete cds

ATCGTCACTTTCATAAGTGTGGCTATTCGTATCTTCTCTGGTGTTATGTTTCCAGGACTTTCGTCGGACAAGAAAATATTTATCATTACTGCATGTATTTTAATCGCAATTTGTAGTTCAATCAACATTGCTTGGTTGGTTTACCGCTGTGAGCAAAGTTACGGACAAAGAAATACTATTATACGCATCGCCGATCACATGCTCGTTGATAAGAATATAAGTGAATCCATGCGTCGAACTCTATCGGAGTTTCGTAATCTGGTCGACTCCAGGCCAGTCCAGTTCACGGCTATGGATTTCTATCCTCTAAGTTACGGGCTGGTGGTCTCATCTGCTTCTGTTGTGACAACCCTGACTATCATTTTGCTGCAGGGTTTAGAGTAGAGTTGTAAAGAGGCAATTTCATTCTCCTCTCACATAATCCTTTCTAGAATTTCTTTGCTAGGGCAATTTTCTTCGCATTTAACACGCTACGTAGCTCTAAGCGCCAAGTGAAGCTTCTGTTTGTGAATATATCTTGATTTGTAAAATGATACACTGCTA

>GR63a.1 [Moltype=mRNA] [Organism=Loxostege sticticalis], complete cds

CAGAGACTGGTGAGCAACAACACGTGCACTACTCATAATAAATACTTTAAATTTATTTGCAACGGATATAAAGGCACCATTGCCTTTACAAGAGTTATAAAACAACCAAGTGCTATGGGTCATGGGTAATACATAGCTTGTTTGTGTGAAATGGAAGGTCGTTATAAAACGTATTGATTTTTCCGTAACTTATCATACATCATATTTACATTGATTGTGTATCATTATTGTCAGCTTGCCGCGGATGATACGCCACCACCTTTTCACTTGTGCTTGTAGAATATCGTGGTCAACTATAACTGGATATTGGAGCGTATAACCCATGTAAAAAAGATTTTTGTCCCAAATACAAATAAATAATAATAAACAAATGGACGAAACGAAAGTTGGACAAAATAAACAAAATGAATCAGAAGACGCTTTTGACTCTTTGAATTTCATCAATCAGTTATTAAAAATATTTTGTTTATCGATTCTAAGCCGTGAAAACAGGCGCCTAAAAATATCATACTCATGGTTTAAAGTATTTTTTACAATTATGTGTATAATTTGTTTGATAATCTTCCTGACTTACGACATTGTGAAGTTCTATGCCTATGAAATTCAGCATTTTAAATTCAATGATGAAGTACTATTGGTAATCTTGGTTAGAGCCGTTTTATACAGTATTGATCTATGTTATGTTTTCAAATTTGGTGGAAATACAAACCTTCACTATTTTAAGCTATATGAACAAATTGATACAATCCTAGACACAGATAATGCAATGATCAAAACAAAAGTATTGAAAGTAACAGTTTTTATAACTTCGCTATATGGCATACAGACAGTGGTGAATATAATTTGGGCTGCCTTTTATGATCCTACTGAATCATTTACCACAGTAAGAGCAACTGTTGGAATTATTATGATTTACATAAATTCTCTGTCGATATTAGAAATGTTGGTCCATGTGATTCTAATAGAATATAGACTTATTAAAATCAATAATATACTGCAACTTCGCTGTTCATCTACCACCAATAATTTTGGTGCATTATCTGTTCTTGTTGAAAACAACTGGCTTTATTTCTCAAAACACAAAGAAATTACAAGAAACCCACAGGTAGATTGCAACTACTTCTATGACATCAGCTGGCTGAATAAATGTTATTTGTTGCTGATCGAACAAAGTAATTTTATTAACAAATTATTTGGCGTCAGAATCTTGTTGAATAGTGTAATTAACTTATGGGACCTAGTCAACAACATAAATTTTTCCATTCGAATCTCCTTCCGTGTACTGGATCTGGATCCTGAAACGACAATTTTAAACATATTATCATCGACATTGAACATATCTTCAGTCGCTGCAATTCTTATATGCTTGGTTTACCGTTGTGAAAAAACTTACGAACAGAGAAGAAGTATTATAAATGTTATGGATCGCATACTCGTTGAAAAATATATCAATGTATCTATGAGGAGCAGGTTGGCTGAGTTCCGTACACTCGTGTACTTCAGGCCCATTCAATTTACAGCTGCGCATTTCTACCGACTGGACTACGCCCTGCTCGTCACATTTTGTTCTGCTGTTACCACTTATTCTATAATCTTACTGCAATACTTGCAGTGATAAATGTACCTCTACTAATATTCATTTGTTTTAAATTAAAGAAAGTGTTCCTCTTTTTAAAGTGAAATAGTTATAGTTAGATATTAAAACAACAAA

>GR63a.2 [Moltype=mRNA] [Organism=Loxostege sticticalis], partial cds

CATACTTCATGTAGACACAATTTGCAATTAAGTAATTTGTCCTGTCCGTAACACCTCGGCATCGTAAACGATGGACAATCGTCGGAGAGCTTTATTACCGAGTAGACGTTACAGTTCTAAAAACATGATTCAAGTAAGTGATGTTAAAAGCGTCACTAGTGACCAATCCTGGTTCAATAATCCTTTAAGCTTTTTACTACAACTATTTTCCTTGAGCACCAATGCTCACCGCGATAAAAACTTTAACTCGTGCTTATCTCTCATTAGGATGATTATAACGGGAGTGGGTTTCGGTGTATTGCAATTGTTTGATCTGTATTACAAAATTGGCCATGTATACAGTGGTCTAAGCGTTTCAGTAAGGCTGACAGACTCGGTGCAAACTATCTACGATTATTTTCAGTATACTGTGGACCTGTTCTATGTTTATAAATATGGCAGGCATTTTTATCAAGAATACTATAAACAGTACAATACAATTGACCAAATACTAAGGGCAGGCAGTTGTAATGCTATAAGAAAAAAAATAACCAAATTAGTGATTTTGTTTGTTTCAATTTGGTTGATTACTTCGGTGATGGATTTCATTGCTTGGGTGTTAATTTACGGATGGACTATTCCAACTGTATTCTCGTTGGCATACAATTATCTTCTTTTGAAAATTCTTACTAATTTAGACTTGACTTACCAAACTATGCACATTGAAGTCCGTCTGCAAGTCATAAGCGGATTGATGCAAAGTTACTACACGTGTTGTGACTCGTTGCCTGGGGGACCTGGGGAGAAGTGCGGTGACCCAGTCCAAAACAAGAACTGGCTCTATTCAAACTTCTCTGTGCCACCCAAAGACTCGTTAAAGTGGTCTGCGGAGAGCCGGCGACATGGAATCCGGTGGCTCACCAGATGTTACTTGCTGCTGAAAGAACAAAGCGCATTCATAAATCAAATGTTTGGCGTGAGGGTACTACTGAACAGCCTGAGTCTTTTAATTGATATGGTGAGGTTCTCAAATCTAGCCATCAGACTCGTCATGGGGTTACAGCAGGACGGTAATGGGGCAAAGGTGACTAAATATATCGTCAACGGTGTAAAGCGCAACATGCACGACTGGGAATATTTCACTGCAATCTCAACTGTATGTCGCCTGTTAGTTTGTGCTGTAATTCTTACGAACCTGGTGCACCATTGCGAACTGGTATATCGCCAGACAGATCGGATTATCAGCATATCGGATCACTTACTTATCAATAAAAACCCTGATCCAGACCTGCGGGAAGCTGTCACCGAGTTAAGGGATCTAGTGCAGTCTCGGCCCATCGACTTCCACATGGCCAACTTCATCAGGCTCGACTACTCTATGCTCATGTCCACCGCC

>Orco [Moltype=mRNA] [Organism=Loxostege sticticalis], complete cds

CGCCGATCGATGAAGTTATCCACTTCGTTCTAAGTACCTGTGGAGATCAGACGCGTTGGAACAAGCTAAAAAGGCAGAGGAAAATGTAAGCAGAATCAAAGCGAGCATTTCAACAATTAATATTGTTGAAAGTTGTATTTACAGGAAGTGAATTAATTAATCTTTCAAGATGATGACCAAAGTGAAAGCCCAGGGCCTCGTGTCCGATTTGATGCCGAATATCAAATTGATGCAGGCAGCTGGGCACTTCCTGTTCAATTACCATTCAGATAATTCTGGAATGACAACGCTCTTGCGTAAGATGTACTCTAGTGTCCATGCGTTTCTCATCGTCATCAACTATCTGTGTCTGGCTGCCAATATGGCCCAATATTCTGACGAGGTGAATGAGCTGACGGCGAACACAATCACAGTACTGTTCTTTGCCCATACCGTCATCAAGCTGCTGTTCTTTGCCGTCAGCTCCAAGAGCTTCTATAGGACACTGGCAGTATGGAACCAGTCGAACAGCCACCCTCTGTTCACGGAGTCAGATGCGCGATACCACCAGCTTGCTCTCACCAAGATGCGGAGGCTGCTGTACTTTATCTGTGGAGTTACTGTCTTTTCTGTCATGTGCTGGATAACCATAACGTTCTTCGGAGAGTCTGTCCGTTACATCGCTAACAAGGAAACAAACGAAACCCTTACGGAACCCGCTCCGAGGCTGCCTCTTAAAGCCTGGTACCCCTTCGACGCCATGAGTGGCACCATGTACGTCGTTGCTTTTGTTTATCAGGTCTACTGGCTCCTCTTCTCAATGGCCATCGCAAACCTGATGGACGTGATGTTCTGCTCATGGTTGATCTTTGCCTGTGAGCAGCTGCAGCATCTGAAGGCGATCATGAAGCCATTGATGGAACTCAGTGCCTCTTTGGATACTTACAGACCTAATACTGCTGAGCTGTTCCGAGCTTCTTCTACGGAGAAATCAGAAAAAGTCCCAGACCCTGTGGACATGGACATCCGTGGCATCTACTCCACGCAGCAGGACTTCGGAATGACCCTCCGTGGGGCCGGTGGAAGGCTCCAGAACTTCGGCCAGCCTAACCCGAACAACCCCAACGGATTGACTCAGAAGCAAGAGATGCTGGCCAGATCTGCCATCAAGTATTGGGTGGAGAGGCACAAGCATGTCGTCAGATTAGTGGCATCCATAGGGGACACTTACGGTACTGCTCTACTATTTCACATGCTGGTGTCTACTATCACCCTGACTCTTTTGGCTTACCAAGCTACTAAGATCAACGGTTTGAATGTGTACGCATTCAGCACGATTGGCTACCTCAGCTACACTCTCGGACAAGTGTTCCACTTCTGCATATTTGGAAACCGGCTTATTGAAGAGAGTTCATCAGTGATGGAAGCAGCCTACTCCTGCCAGTGGTACGACGGCTCTGAAGAAGCCAAGACCTTCGTCCAGATCGTGTGCCAGCAGTGCCAGAAGGCCATGAGCATCTCTGGGGCCAAGTTCTTCACGGTTTCGTTGGATCTGTTTGCATCTGTTCTTGGTGCGGTGGTGACATACTTCATGGTGTTGGTGCAACTGAAGTAGATGATTTAAAAAATATTTTCATAAATGTATCAATTATTTAGGATAAAATCATCAAAATCCAGTATTATTTAGGATAAACGAATTAAGTAGGTTTTGATACATCAATGGTTGATCTCATATTTGTTGAAAAAATTGTTACATACATAAAAAGACGCAAATTATCTAAGGGGGTGATTCTGCTGTTTGATTAAAACTACGTAAAATACAGAACATTGATCGAAATTTAAAAAGTAACAACAAAACTGAATTTGAACAGAAGAATCACCCCCTAAGAGGGCTGTTATTCATTTATTAAGTCCTTCTTATATTATATTTCTTGTTCTTATAAAATTGTTATATTATGCTACAAAACATCTATTTTAAATTACTAAAACTAGATACCTACATCTAGTACCATCGATAATTTTTACATCAAAAAAGTTTTGATCATCATAATAATATAGTACAAATTAAGTAGCTTTTTTCATTATAATAATATGAAATCGTTCGATTGTTAACTTACATTAATTTAAGATTTTATTCAATACTGTAAGAACAAAATAACAAAAAGTATTATTAATGT

>PR1 [Moltype=mRNA] [Organism=Loxostege sticticalis], complete cds

GCCAGTCGAAAACATTTCTAAAGCAGGTATTGAGTTATTAACTGCTCGGAGCACCGAGCATTTTTTGTGCTTTTTCAATACTCTAATGTAATTTATCTGCAATAATGAAGACTGTCGTTGACGCGCCTGATAGTCGAGATTCTCCATTAGGACTGGAATACATAAAACTACTAAGTAGGTTCTTGGTACCCCCGGGTACATGGCCAGCTGATGCGTTTGGCAAGAAAGTGTTTATCGGTGTGAAAGTGCACCGATACACTTTGCCATACCATACATTTATGATAGTTTTCGGATTATTCTACTACTTACTTGAACACGCCTCCGAAATGAACTTCCTTGACATTGGAGAAAATATACTGACCACATTTTTAGGCACGGTAACAGCGGTAAGTAAACATTTTGTTTTTCTTATGGAAAGTTGGCAACATGGTGGCAACCAGCGAAAGTAAAAATAAAGAAGGTATAGTATGTATTACCTACGTGAAATCTAGAGATGTTGGAACTATTTGTGATAGGGCTTACACAAAGTATCTTTTCCACTATGTTATGTATGTTACATCTTATTTTCATTAACAGAATGAAGTTAAAGTAAAAGTATAAAAAAAAGTTATGTTTAAATAATTTAAATCTACCTACGTCGTAATATTACATCTAATCTAACGTGAGGCCGAGTAGTATGGCTATGACATTGGTTATTTCGGTATGAACTCTCTGTTTATTATCCCTTACTGACTTGGGGCTTAGTTATCGTCCTCTTGTTTTCAAATCAAAAGTCCTCATTCGATGTTAATTTTATACAATAGGTAAGAAGTATTCTACCAACTATGAAATCATACAGAATACTGATTCGGAAATTTGGGAGGGAATTTCATTTGGAACATTATACACATATGGGACAAATTTACGAAGACATGAACAAGAAGATTAACGTAATTTCGGTATATTTTACTAGATTCATGATGTGCCAAATGATTCTGGCTATGATCATGTTCAATATTGCTCCGATGTACAACAACATCACCAACCGTTATATCCGACATACTGAGAACTACACTTTAGAATTCTCCTTATTAATAAGCTATCCTGGATTTAAACCGCTTAATTACTTTGCAACCACAACGGTTTATAATTTCTATCTATCGTACAACTGTGGAGTCATGTTGAGCGGGCTTGACTTGATATTATCTCTGCTGATTTTCCAAACAATTGGACACGTCAAAATTTTGAGGCACAATCTTGAGAACTTTCAGTCACCCAAAAATAAAGTGGTTATCAAACTGGATGAGCCTCATAAATACAAATTTCATGGAAGCTGTCTTTACGAAGTATTTGACGAAGAAGAAAATGAAAAAATCCGAATCAAGCTGGCGGAGTGCGTTGAACACCACAGGCAAATTATAAATTTTACAGATGAACTGTCGGAACTATTCGGTCCTTTTATAGCCTTCAACTATTTGTTTCATCTTGTTGGCTGTTGCTTGCTGCTACTGGAGTGTACAGGAAATGATGGTGGTATGCTTCGCTTCGGGCCTCTTACAACGGTTGTTTTTGGTCAACTTATTCAGATTTCATGCATGTTTGAGTTAATGGGGTCAGAGGCAGAAAAGTTGAAAGACTCTGCCTATATGGTGCCCTGGGAGAGCATGAATGTTAGCAACCAGCGCACGGCCAACATAATACTGCACAAAATGCAGTACAAAATCAGCCTCAAGGCCCTAGGACTGGCAGCCGTCGGTGTTAACACCATGACTGGGATTTTGAAGACTACCTTTTCATATTATGCATTCTTACAAACACTTTAAATTAAAATCACTCCTTTTTAAGTGGCTGAAGAGTTTCAGTTTTAAATAACAAAACAGAGTATTGTCAGTTTTGTACCTAAAGCATTCAAGCTCGCACGTAATGAAGATAACTCGGTCTGTGACCCCAAGAAAAATTGTGAAATTCCAGATTAACAGATTTTTTTAGAGCAGCGATTTCGAAGAGGATGAATGAATTGATCACAAAAAATTAAACCTGATAATCTAATTTTTGTCCCGGAAAGTTCTAGTGCAAAAATTGCATGTGCAAACAAACAGCATTTAATATGCGTAGCATTTTTTTGTGCACAGGCACAAATATGTTTACATTAGCATCATAACAATTTTATATAAATCACAAAGTTTTCGTGCACGTTTTTAATGTATTCTATTCTTTTCCCGTCTTTTATGATTTTCAGCTTAAACTCAAAACCAATTATTGTGAAGGTTTTGTTCTAATAAATCCAAATAATAAATGCATTGCTTTCACTATTTTTATCATAACTAAAGATGAACAATGGTGATGCTCATAGTACCCGTATGATATTAATCCACCTTGTATATCCCTTCGTCTGTTGACCACGCTCCTGGAGATTGACGTCAAAAAACCCTTATCTGTTATTCCATTTCTAATAAACTAACCACTTGTAACTGCCGAAACTTCGTAAGTGATAAGAATGTGAACCTCAAAGCATACCCGAAAGCATAACATAAATTTTAATAAGGTTTTCATACAAAACTTTAGAG AAATCGTGATTTTTTCATACTGTCGCACTGTCTTGCGACTGTCGCGCGATTC

>PR2 [Moltype=mRNA] [Organism=Loxostege sticticalis], complete cds

TTGAAAGTCAATCATATTATTGAAAGCTACATAGACTCCACGACCTAAAATATAAAATCTGTTGAATTCATTAAATCCCTCAAAATGAAGAATAAATCCCCTTTAACACTGAAGTACATAAAAATAATAAGATCATTTTTGAGACCACCAGGCGGATGGCCGTCCGAGGTATTCGGTGAAAAATTGTCGTTAGCTATACGATTTCACAGAGTAACTTTGCCATTTCATACATCTCTCATAGTTATTGGAGGATTTTATCATCTGTATGATAACGTCCATCGGCTGAGCTTTCTCGAGTTTGGGCACATTATTATAACAACTTTGTTGGCAATGGTGACAGTTCTCAGAAGTGTCCTTCCAAACTTACAGAAGTTCAATTCATTGCTTAGTAAATTTATCAATGATTTTCATTTGATGCATTTTACACACAAAGGTGAATATTTCGAAAAGATGAACAAAATGGTAGACTTAATTTCTAATTATTACACTATGGTCAGTACTTGTATGATGTACGTCGGAATGCTGATGTTCAACATAGGTCCGACATTTAACAACGTCAGGAATACCGTATTTCTTAAAACTGAAAACTACTCTATGGAATATTCCGTATATTATAGCTACCCTGGATTCAAGCCGCTTGATTACGTTACGATCGCTTCGATTTATAATTGTTATTTGTCGTATAACTGCTCAACCTTGTTATGCGGATTCGATTTATTGTTATTCCTGATGATTTTTCAAACTATTGGACATGTCTACATTCTGCGACACAATCTTGAGAACTTTCCGTCGCCTAACAATAAGATAATGCTCACATTTTTGGGAGATAAATACCGTAATAAAGAGGGTTGTATTTGTGAAAAGTTTGACCCCGAAGAAAATAAACTAGTTAGTTTAAAGTTGGCCGAATGCATAGAACACCATAAAATAATAATAAGTTTCACAGATGATCTTTCGCAGATATTCGGGCCTATTTTAGCATTCAACTATTTTTTTCATTTGGTCTCCTGTTGCTTGTTACTACTGGAATGCTCAGAAGGTGGTCTTGATGCAGTGATTCGCTTCGGGCCATTAACACTTATTGTTTTCGGTCAACTCGTTCAAATGTCAGTCATATTTGAGTTACTGGGTTTAGAGACGGAAAAGCTGAAAGACTCCGTATACTGTACACCATGGGAGAGCATGAGCGTCAGCAATCAGCGCACTGTTTGCATAATACTGCATAAAATGCAATACAAAATAAACCTGAAAGCATTGGGACTGGCAGCAGTCGGCGTCAGCACCATGACTGGGATTTTAAAGACAACGTTTTCATATTATGCATTTTTGCAAACGATGGGAGAATAGTGCCAGTGCCAGTAGGTACAAACCGAAAAAGTATTGTATCTATTAACATTATTGGCATATACGAGTATTTATGGACCTTCTACACGCTGTTGCCGACATGTGGTAGTGTACGTATTATAATCGTCCTATGTGAAGAATTTGTATTTTCTGCTGAAGGTAAATCAAACACATATGAATAATAAAGGAAAGATCATGGCTCCTGCATAACTTAGTAAAAAGTAAAGTGGGCGAATATTTACTTTGGATGAAAATTATGGATAATGAATAGACCTTCATTTTTATTTCTTAAATTAGTACATTTCACGCATCATTGTTACAAAACGGAAACAGCACAAAGTTAATTAACCGAATGGCGAGTTAACAGAAAGTAATAGTCCTTAAACATTTTAATGTTGATTCCATGAGTTTAACCAACTAGTGAATTGGAACCATAAAATCGTTTTGGCTCATGTCAAGGCTCTGACAATTAGTGACAAAAGTTAAGAAAACACTTCCTGGCTATCTCGCCATACAATATCTCTTAGTTTTGTTTGAGTTATTTTTCTTTGAGTTTTGAAAATGCAAGATGCAATTTTGAATTCTGGCACAATTCAATACCATACATTATGTTAAACGCGTAATTTGTATTGTGTATTTTAAAACTACAAGAAAAGATAGACGTTCGAATGATCTTCAACCATATAGTGGTGGACGACCACCTAGAGTCTAAGTGGTCGTCCACTCTATGTTTTAGACCAATAAGATAGGTATAGTTAGTAGTACCTAAGCATTCCTCGGCATTGTTTTCTGTTGCAATTTGATTAATAACACATTTTTTACTTCTAGGTAGTTAACTCACGTTTTATTGTAATCACAATTAAAGATTTATCACTGGCAA

>PR3 [Moltype=mRNA] [Organism=Loxostege sticticalis], complete cds

GTTAACCATCATTAGTTAGTACGCCGTATTTGAAACTGAATAAAGGAATCTACGAAAATGTTCAAAATTCATAAAAATGATATGTGAGTAATTAAATATGAACAAAATATAGCCGTCTCATAATGTTAATATTTCTTCATTTAGACTCGTATGTGAAAATGAAGATGATCGAATAGTGACCCTGAATGGACTCCAAACAACTTCATAATTGAAGCGAGGATTGGGGACCTCCTACGAAAAACGAAAAGACAAAAGGAAAAAGGGATTCGATTTAAGCATTGGAGAGTTTGAGCAGTAGCTTTATCATCCGACTATCGTTTGTGTTTCGTTTCGACCGAACTATCGTCTTTCGTTTTTTGTAATTGGGCCCTGTGCCAGAACGACATAAATATGCTTACTTTGCAGCAACGCCCGTCATCCTACGGACCTGCACTACATGAAAATGATCCGGTACCAGCTCCGCATGATCTGCAGCTGGCCCCAAAAACTTTTAGGCGAGGAGGTGAAGTCCGTTCCTCTTAGAAACACCCGCTTTCTCTTCATCGAAGGATCCTTAGTCGCTTTCCTCGGCCTCGTGTATATCAAGACCCACCACACCAGAGTGACTTTCTTAGAAATGGGACACACTTATTTGACAGTCTTCTTGGGTATAGTCGCTGCGCAAAGAGTCACTGTTTCATGGTTCAAATCATATGATCAAACGATGAAGAATTTCGTTCTGGAATTGCATTTGTTTCAGCACCGACACAAAAGTGAATTTCATGAACACATGTATCAATACATAAACAAAATCTGCACTGTGTTTGTCGATTTCATTCACGTGGAACTGTTTATGGGGATAATTCTTTTCACCTTGACGCCTGTGTACAACAACTACATGAAAGGCATGTTCAACCGAGCGACGCCTGTTGGGCCTGACAAGTTTTTCGAGCACTCCATAAATTACTCCCTCCCATACATAAACCAATTCATATACGACGAATTGGTCTCATACCTTTTCATAGCCATTCTCAACGTACTCTTTGCTTACGACGCCGGCATATGCTTCGGGTCACTTGACGTCACTTTATCCGTCATCGTGTTCCACATCTGGGGACACCTGAAGATCCTCGACCACAATTTACGGTCAATCCCGAAGCCGGTTAATGAGTTAACGTACACGGCGGAAGAAAATAAAAAAGTTGGCGGTATACTCAAAAATATCGTTGATCATCACAGAATGATAATGAGCTTTATGACCAATACATCAGATGCGTTTGGGCCGATGTTGTGTCTCTACTACATGTTCCATCAAGTCAGCGGCTGCATTCTACTGCTGGAATGTGCTGAACTGGACGCCAAGTCACTCACTCGGTATGGAGCCTTGACAGTAACTATTTTTCAACTTCTGATTCAGATATCTGTCATTGTTGAACTACTCGGCTCTCAGGTAAGAGTCAACAGCATAAGTCAAATGGTTTTTAAAACAAGGACAAATTGTTTAACTAATCAACGAGTACGCTACGTACTATAGCGCACACTTTGCATTTTAATAATAAAGTGCTGCATAGTTGGCTTGTCGTCAATATTGGGCGCTGTGGGAGAAATTCGGCTAGGAGAATTACTATTTGAAAATCCACAAATCATGATGTGTGATCGTGACAATCGTGTGAGACCTTATTCCAGAGTGAGACCCTGAAGGACGCGGTGTACAGCGTCCCTTGGGAATGCATGGACACCAGTAACCGGAAGCTGGTGCTGTTCCTGCTGTGCAACGTGCAGGAGCCCATCCGCCTCAAGCCCATGGGCATCGTGTCAGTCGGAGTCCAGACCATGGCCACTATTTTGAAAACATCATTCTCATACTTCATGTTGCTGCGAACATTCGATTAAAGTATTTGACTAATGCCTAATAATCCAATTACTTTGGACACTATATTTTACCCTTTGCTATAGTCACATAGTTAAGAGAACTTATCCCCTTCTTCACAAAGCGTGCAGTAAATTACAATGCACAATTTATTAACTTTCAATAAACTTGCTGATGTCTCTTAATATAATTTTAATTCAAATGATTCTGTATTGAGGAAATTAGATCATTATAACAGTGGCCTACGTACCTCGTAATTTAATTTCATTGAACGGGATAAAACGCCCTTTTTCAATTTTTGTTAAGCTTTTGATAATGGTTAAAGAACAGTTTTATTTAACGAAACTTATGAATTACGTTGTCCCTTAATAAATCTGTGACGTGTCCGTCTAGAGATTTTGAACATAAGGGTAGAACACAAAAGTTTATATGAATATGAGTGTGCCATAGGGATCAATAGTAGGACCACTATTATTTCTTATTTAATTTAATAAGAACACCATGTTCATGGACACCTAATAGTTTCATTCAAGTTTTTTTTTAAATCTCTCTTCTTTGCACATTTAATTATTCAATAATTGAAAGGTCACCTCTTTCAACCAAGTAATTATTCTGCCTTGAGAATAAACCTTATTCAAAATAATTAATAGGTTAGTAGTGTTGCTAATAAAAAGTCTTGTTCACCGAA

>PR4 [Moltype=mRNA] [Organism=Loxostege sticticalis], complete cds

GGACTATTCCGATTGAATAGGAGACAACACTTTCTTTGTCGCTTTAGTTTATGCATAAACTCAGAATGTTTTTAATCAGTGACGGGAGCGACCTTAAAGACGTGAAACGAGTTGTAGACATAAAGTATATACAGGTGCTGCGTTCTTACTTGCGTATTATAAGCGCATGGCCGGCCAAACATGTTGGCGACACACCAACTAAATGGGACCGAATAAAAGGCAACCCCGTTCTGGTCTTAAGCATCATAAACTTTTTAACTGGACTACTTTACTTGAAGGAAAACATTGGGAAAATTAAATTTTTCGATTTGGGCCAAACTTATATTACTGTGTTGATGAACTTAGTTTCCGTGTCTCGACAACTCATGGTATACCAAAAATCATATACAGAGGTATCCCGGGATTTTGTGACAAAAGTTCATCTGTTCAACTGGAAAGATGACTCGGAATACGCTATGGAGATACACATATTGGTTCATAAAATATCTCACTTCTTTGTAATGTACATCCATGGGCTGATGTTCATCGGACTCAGTATGTTTAACTTGACACCTTTGTACAACAACTATAGTAATGATGCGTTTACCAAGAGACTCCACGGAAACGCTACTTTGGAACATGCTGTGTATTATTCGCTGCCTTTTGACTACACCACTCAAATTCCAGGATACATTGTTGTATTTACCTACAACTGGTTCATATCTTTAGTGTGCTCCATCAACTTTTGCTCGGTGGACACGTATATGTCTCTTTTAGTGTTTCATCTTTGGGGACATTTAAAAATCCTCATTCACAATTTGGAGCACTTACCCAAACCTAGTGGGTTAAAATCTGCCGCAAATATAAACGGTGCAACCCAAACTGAGCGGTACAATGAAGATGAAACTCAGCAAGTATCTGAGCGATTGAGAGACCTGATTAAACATCACTGTCTTATTAGAAATTTTATCAGCATTATGTCCAGTGCCTTCGGATACGTATTATTCGTATATCTGGGATTCCATCAAGTCTGTGGATGTATACTGCTTCTGGAATGCTCTTCATTGGAACCAAGTGCTCTAACCCGCTATGGTGTCCTCACTGTGATAATATTTCAACAATTAATCCAATTATCTCTCATTTTTGAACTACTTGGAGCAATGACCGAAAAGTTGATGAATGCCGTTTACAATTTGCCATGGGAATGTATGGAGGAGCGCAATAGGAGAATGGTTTGTTTAATGCTGAGGCAATCGCAGTTACCGCTGCGCTATAAGGCGCTAAATATGATAGAGGTTGGCAGTGCGACAATGGTCACAATACTGAAAGCATCAATATCATACTTTGTCATGCTGCAAACTTTCGCAACTAAAGATTAAACTTACCTACATACTAAACATGAATGTAGGTACTTGAAATAATTATATTAATCAACTTCGCCTGCACTTCAAAACCTAATGCCGCTCCATTTATCTTTACACAATTTCAGAAACTATGGCATCACAACATGTCTCACAAATAGAGAATCAGATGATCAAAAGCTACTTCTATTTTCTTTAGTTTTAAAGACATACACAATATTCACAATAACATTGATGACGAAAATAAACCATTTTCACAAAATGAATTTTTGCTCTACCTACGACAATTTCATTGCCACAACTTTCGATCGTAACTGTGTCGATTTTGCCCATGATCCTTTTTTGCAAAGAGTCCGTCCTAATCGACAAATAGTTCCGTAGTTCCTCACTCATACAGAATCTGTGTGGCCCATTGAAAGGCATTTACCCTTTAAAAAAACATTGAAGAACCCGAGC

>PR5 [Moltype=mRNA] [Organism=Loxostege sticticalis], partial cds

GAATTTGGCCAGTGCAAGTCAATTCTGTTTTATGTTTAAAAGGCATAATCAAATATGCTGGAAAATCAGAATGAAGGCGACATGTAAGTGAATACGTTATTATAACCTATGAGTTTTCTTTTGTTAACCTATTTCTACTATCTGAATAATGGGAATATGCCTCCCCAAACTCAAATCTGCTTAAGGCGATAGACCATAATTGCTTTCTTCTAAACTGTTAACAAGAGTGCAGTATCTTCCGCGATGTAAATTACCGGCAGCGCCACCTATTAGTGGCGTGGTGAACAGCTATCTAATTTATCCATATGAATCCACACGCAGCAACGCCCGCCATCCCATGGACCTCCGCTACATGAAGCAGCTCCGAAACTTCCTCCACCTGCTTGACTGCTGGCCGCACCGCCTTCTAGGGGAGGACGTCAAGCCCTTCCCGCTGAGGAGCATCCGCGTCCTAGTCACGGAGTGGATCATCATTCTAGTCGGAGGAGTCATTTTCCTGAGAGCCAACATCAACAAAAGAGATTTCATTGAGTTGGGGCAGACTTATTTGACTATATTTTTGACTGCATTCGGTATACAAAGAGTCACCATTTCATTGTCCAAGTCATATCAAGAACTAATGAACGACTTTGTCTTGGAAATACATTTATTTCACCACAGGCAGAAATCAAAATATTCTGAATACATGTATCAACACATACACAAAATCTGCACAGTATTAGTCAGTTTAATGTATGCAGAAGCAATTATTTCTTCAGTCCTTTTTAACGTGACGCCCTTGTATAAGAACTACAAGAAAGGCATGTTCAGTCAAGAGAGGCCGTCGGACAAGAGGTTTGAGCTCTCCGTATATTACTCCCTCCCGTTCGTCAACCAAGAGACCAATTTGTTCGCGTACATCGTCGTATCCATCTTCAACGTGACCCTGACCTTTGACTGCGGACTGATATACTGCGGCTTGGATGCTAATCTGGCTATCATCGTTTTCCACATCTGGGGGCACCTGAAGATCCTCGACAACACCTTGCGGTCGATTCCAACGCCGGTTGAGATGCGAAATCATATTCCTCGATTCGACGATAAATTAAGCTACACGAAGGAAGAAAACGAAAAAGTTGCTGCGATGCTAAAATATATTATCCACCATCACAGATTGATAATGGGATTCATGACCAAAACATCGAGTGCGTTTGGCCCCACGCTGTGTCTCTACCTCCTGTTTCACCAAATCAGCGGCTGTTTTCTGCTACTGGAATGTTCGACAATGGACGCGGAGTCTCTCGGACGCTACGCCGCTTTGACAGTGATTTTCTTTCAACTTTTGATTCAAATCTGTGTTATTGTGGAACTTCTCGGTACTCAGAGCGAGACTCTGAAGGACGCGGTGTACAGCATCCCGTGGGAGAGCATGGACACCAGCAACCGGAAGC

>OR1 [Moltype=mRNA] [Organism=Loxostege sticticalis], complete cds

GTGCCATCAACCGATTGGCATCATGTTGGTGAAAAAGTTTAAGGCGTTTTATAATAAAGAGGGTTTCGATTACTCCAAGGGATATATCGACCCCCGTGATTTTCATCTAACGTTTTTTTTCGTTCAGAGGGCCTTCCAAGTGATCGATGAGCCATTTCAACCATGGACATATGTTTCAAAAACAATCACGGTGATCTGCGGAATCGGTGTTTTGACTGATGCCTGTTTTTCTTTCTACCACGCGATTGACATTTTCGACATGGGCATGATAACGGAGGCCGGAACATATGTTCTCATGTTGATGTACAAGATGATGACACTTACCATCACCAAAATTAATTTGTCCAGTTACATACACCTGATTAAGTGCATGAAGGAGGACTTCGCGTATATTTGCACCAAAAACGACAAGTACAGGAAAGCGTTTTTTGAAACTCACATGGCAACGTGGCAGCTTTGCGTTAAAGTTTGTATGTTTATGTTCTGGCTGGCAACTAGTTTAGTTTTATTTGCAATTGGGTCCCTATTCTTTTACTTGGCCACCCATGAACCGGGCGATGGGACCCACAGACCACTGGTTTTCCCTTTTTGGGCTCCCGGAATTGACTACACGACGTCACCCGCCTACGACATAGCTTTCAATTTTGCCAACATCGGAGTAATTGCTTGCACATACAACTACACATTCGTACTTCAAACAAACATAGTATGGGTCCGACAAATCGCATCAAAGGCCGAAATGATCGGGATGTGTATCTCAGACCTTTTGGAAGGTATTCAACCAGCCAATAATGAAGAAGAAAAGAGGCACTACGCTAGAATGATCAATTTTCGAATGAGAGAAATTGTTTCTCAGCACCAAAAGATGTACAAGTTGCTAGACAGTTATGCTGCTGTGTATAAGAAATGTTTGATGTTCGAACAGTTCGTATCAAGTCCGGTTATCTGTATGCTGGCTTATTGTTCCGCAGAGGTATGTTTTTTTAGTGGCTTTATGAGTTTGCTGTACCTGAAGATAGAGAAGAGAGGGAACGCTTTAACTATTAATTAATAAAAAATCCAAGTTATTGATTACAGATGCGCTGCATATGAGTTCTGTCAAATACTTACAAATAATTCTGTTTAAGATGGTAGACCTACTGCCGAATAGATAGAAAAATAAACGGCGCATCTCTTAAAATTTCCAAATCTCTCGCGTGGTATTGCGATTCTTTTTCAGAAAATTGATGGCGGTGAAATACATGTCGTGATGATGGTGCTCTGTGTGGGTGCTATATTGATTCTGTTCCTTCCGTGTTATCTTTGCACTTATCTCAGAAGTAAGGTCACTCTTATAGTCGATGCGTGTTGGGAAATAAGATTCTGGGACGCTGGACCCAACATACGCACATACTTGATACTGATCATGCAAAGATGCTTGCGACCCTTGCCACTCCAAGCGCCCGGGTTTCAAGAGGTCTCCATTAAAACATTTTCAAGTAAAATGACATCAGCGTACTCATTGTTCAACATGTTACGTCAAGCCGACCTTGATTTGTAAATTGAGAAATAAACACGATGTTCGAGTTTTCATGATCATGGACCGTCACAACAAATAATACTTTATATACACACAGATTTGAGATTCTTAATTTTTTTCTTTAATTTTAATGGACAATATTTATTTTGTTAAACATAGTAAGTAATTACCTACATATCATCTTCACTTAGAGAAAATTAAAATAAATACTTGCACCTGCGA

>OR2 [Moltype=mRNA] [Organism=Loxostege sticticalis], complete cds

TCTCTCTCAAAGTAGTTCATACCTACTTTCTTCTTATTTTTAAATGCGTTAAATTTTATTTGGGGGCCATGACTCTGATTAAAGCTATAAAAGGGATCTTTTTCAAGGACAGTTTTGATTTTTCAGAACCAGACATCGACCTTTACACCTTTCATCCCCAACTGCGCATCTTCATAGCTCCTTTGGGAATATTTTTTAATAACCGAAAATCTTTGTTGAGGTTCCTGTGGCCGTTCATAAACGGTTCGTTGTCTATCGTAGCAATAGTGTTAGAGATGATATTCGTCTACCACGGCATCACAGTCGGGGACTACTCCTTCGCCACCGAGTGCTTCTGCTATTTCGTCATGCTGTCAGTGATACCAGTGCTTTACTGCGCCGTGCTGGCCAACAGTCAGAGCGTGATGGTGTTGTTGGACAAAATGGACAAAGATTTTGCGTATATCTGCAAATTAGGAGCGAAATACAGAGACCATTTCTTGCAAAGGCAGCTATTGATCTGGCAACTCTGCTGGATTTGGCTGGGGTTCCTGTGCTGTGTGGCGGTGCTATACACGCTGATGACCCTGGCTCCACTCACTTACCAGACCCTGATAGCCACTCAGGACGAGAACATGATTCGGCCACTGCTGTTCCCGATGTGGCTGCCAGAAGACGACCCATACAGAACACCAAACTACGAAATATTCTTATTTCTTCAATTGGATTACCTGCTGATATTCATTCAGTCGTTTGGTGTATACGTCTACATCCAGTTCCACGTGCTACTGCACAACTTCACCGTGCTGGAACTGGTGACATTCGACTTCGATGTGATATTCGAGGGTCTGGACAAGTCCGTGGTTGACCTGCCTCGCGACGACCTTCGTCGCCTGACCGTTCAAAGGGTCTTCAATAGGAGGTTGAAGAGGGTAGCCACTTGGCATGATTCCGTCTTTAAGTCGATCGGGACTCTATCGCGCGTGCAAGGCCCAGTCATAGTGTATCAAGTGATGTTCAGCTCGCTATGTATTTGTCTGATGATGTACCAAGTAGCGGATAAGCTTGACAAGGGCACTTTTGATATACTCTTCATCATGCTGACCGTTGCTGGCATTACGCAGCTATGGATACCTTGCTATTTAGGCACATTGCTACGAAATAAAGCATTCGACGTCGCGGACGCGTGCTGGAACTGCGGCTGGCACGAGACGCCGCTGGGCCGCATGATCCGGCCCGACATCATCATAGTGATCATGCGCGCGCAGCATCCCATCTCCATCAAGTTTACCGGCCTGCCCAACCTGTCGCTTGAGACGTTCTCTTCGATCATGAGTTCTGCATACTCTTACTTCAACATGATAAGACAATCCAATAATTAATTCAACAAAAAAGAGATATCTCAGTGTTCGGTTGGCTTCTAGAATGCATAGAAATGCATAGTACGAGTTATTTATTAAACACCATGAGTACCTTAGTAAATCAATGTTATATCATAAAA

>OR3 [Moltype=mRNA] [Organism=Loxostege sticticalis], complete cds

CGTCTTTGAAGTTAACAACGCAGTGCACTATGTTCAAGAATTTTTCCCTAAGATTCGAAGATCCTGAAAAGCCACTTTATGGCCCGAACTTTTGGATATTAAAGAAAATGGGCTTAATATTGCCTGACAATAGAACGGGAAAAGCATTATACATTTTGATGCATGAGATTGTTGCATTCTTCGTTTTTACGCAGTACATTGAACTGTACATAATCCGGTCCAACTTGGATTTGGTACTGACGAATCTCAGGATCTCTATGTTGAGTATCATCTGTATTGTGAAAGCTAATACGTTCGTATTCTGGCAAACAAAATGGAAGGATGTAATTGACTACTTGACTGAAGCAGACCGCTTTGAACGTGAAAGCAATGATCCACAAAGAAAAATCATCATAGATAAATATACCAACTACAGCCGAAGAGTGACTTACAACTACTGGATACTGGTTTTCATCACCTTCTTGACGACGACTGGTTCACCATTCATACACTTTGCGTCTGCAGTTTATCGAGAAAGTATTCGTAATGGAACGGAAATATTTCCTCACATTTTTAGTTCCTGGGTGCCTATTGATAAGTACCATCCGCCTGGTAACTATATCACTGTAGTGTGGCATATCAGTGTATGTGCATACGGAGCTATGATAATGGCATCGTACGACACCAGCATTATGGTCATCATGGTGTTCTTCGGAGGAAAGCTGGACGTTCTACGGGAAAGATGCAAACAAATGTTAGGCACTGGCGAAGTTGCTCTAAGCGATAGTGAGGTAGCTGCCAGGGTACGGGAACTTCACGACACCCATGTCCTTATAATGAAGTATTTGAGATTATTTAATTCAATGTTATCGCCTGTTATGTTCATTTATGTAGTCATGTGCTCTTTGATGTTATGCGCAAGTGCTTACCAACTAACTTCAGCACAAAACACTGCTCAAAAACTTTTAATGGCGGAGTATTTGATATTTGGGATTGCACAGCTATTTATTTTCTGTTGGCACAGCAACGATGTCCTCGTTAAATCCGACAATGTGATGCTTGGTCCTTACGAAAGTGAATGGTGGGCAGTGAATGTTCGCCAAAGGAAGAATATTCTGCTCCTCGCTGGCCAGCTGCGAATCAGCAAAGTGTTCACTGCGGGCCCTTTTACTGACTTAACCCTTTCTACATTCATAACGATTCTGAAGGGAGCCTACAGTTATTACACTTTACTAAGAGATTAAAATACTTTTGGGAATTGAACTAATTGAAATAGACTCGCATAATTAGCTTAGAAGCGTTTCTTTTAATTTATACAGCTTCTTTTCTCACTTATGTACTACTTAGTTAATAGTGAATACAATATAGTAATAAATAGTATA

>OR4 [Moltype=mRNA] [Organism=Loxostege sticticalis], complete cds

CGCGGGTGGCGGCAGATCACAACACTTACGCAACTGGGCCATCACACTCTTCAGCTTGGGACACCTACGGGAACTATGTCACTAGCAGGGAGAAGCGTATCAGCTCACCTGACGTTCCTCCGGCTTTGCGGGTTCTGCCGGTTGGGCAGAGATGGTTCCTCGCCGCTTGCACGCCGCGCGCACGCCTTCTACTGCAGCTTCGCGCTGGCAGTCACTACCGTGTACCTCATGCAGGAGTGCGTCTACGCTTATCAGGAAAGAAATGATATGGATAAACTGGCACGCGTTATGTTCTTGCTGCTGTGCCACGTCACGTCGATCACTAAACAGCTGGTGTTCTATCTTGATGCTGACCGAATCGACGAAATGATCAGCGGCCTTGACGATCCCTTGTACAACCAGCCAGTGTCGTGGCAGAAGAGCCTGTTGACCGAAACAGCGGTGAGCGCCCGGCGCCTCTTGCGCGTCTACTCCGGCACAGCGGTGATCACTTGCACTCTGTGGATCATCTTCCCCATTTTGTATTATTCGCAGGGACTGCCTGTAGAGTTTCCGTTCTGGACTAATCTTGATCATAGGAAGCCAGTCTTTTTTGTGATTTTGTTGATGTATTCTTACTACGTAACCACTCTTGTGGGTATAGCAAACACAACCATGGATGCATTTATGGGCACCGTGCTGTATCAGTGCAAGACCCAGCTGCGAATTTTAAGAATGAACCTAGAAAATCTCACACAAAGGGCAAGTGTGATTGTTAAGAAAGATCCAAATGAAATATTTGACAAAGTTTTGGAAAGATTGTTCTTGGAATGTTTAGTGCATTACAAACAAATATCAGAGTAAGTAAAATGCGTAGGTAATATTGATAATTTCCTTTTCTCTAGTTTTATAATTTTCATTTGATGAAGGACCAACAAGCGTATGCAGGATATTTTTGGCACCGCCATTCTAGTTCAGTTTGGCATCGGTGGTTGGATTCTGTGCATGGCTGCATACAAAATGATTGGACTGAACGTGTTGAGTATAGAGTTTGCATCAATGACTCTCTTCATTACATGCATTCTCACTGAATTATTTCTTTACTGCTATTACGGCAACGAAGTGACCACAGAAAGCGACCGCATGATGGAGTCGATCTACGCGATGGAGTGGCTGCACGCGCCGGTGCGCTTCAAGAAGTCGCTGGTGCTCATGATGGAGCGCGCCAAGCGCCCGCTGCGCCCCGCCGCCGGCCTAATTATCCCGCTCTCACTCAACACGTTTGTCACGATCTTGAGGTCGTCGTACACGTTTTACGCAGTACTCCGTCAAACGAAATAAATAACGCTGTAATGAATTAAAGCTCGTATGTAGCATATTCATTCACACGTAGAGAAGCCAACAAGTGATGTTTTTATGGAGAGGGAAGGGGTAAACAAATGGTGTGTTACATAAACAGCGGCGGCGAGATAACAACAACAATACTGATACTGCGGCGCTCCCTGTGCGGAGGGCGCCGTGTCGGTTGCGTCACGGAACAATATCGCTTCGCCGCCGGCCCGCCCGGGACCCGGGCAATATTGAAAAGTAATGGACGTCGTAAAAATAACAAAAACCGCAGAACGCCGCTACAAACAGCATTGTGTAGCGCCTGTATTCATGGCCGCAGCGATATTAGACATCTGTGTTATTATTGCTCAAATAAGTATACTCTGGACGATGGTATTACATTCCTTGTTTCGTTTTCCTTTCTTAAATAAAACATCACTGAGTAAAAAA

>OR5 [Moltype=mRNA] [Organism=Loxostege sticticalis], complete cds

CGGTGTTCATGGTCAGTACGTCCAATGCACCAGCTGATTTCTTCGACTTCAATTTGAAGTATTTGTTCTACGTTGGACTCTGGCCTAGAGAGGACTGGCCTCCTACCCTCAATTGGCTTTACAGAATCTATGAGGTCACTCTTATGCTTTTCGCCTTCGCCTTTCTCACTAGTACTGGGATTGGCATGTACATGTCCAAAGACGATGTTATCACTTTCCTTACCAACATGGATAAGGCCATCGTGGCCTATAATTTTACCATCAAAATTGTCATTTTCTTCTTCAAGCGTAAGCACATCAGGGTCCTTATTTCCGAGATTCTTCACTCTGGTGATAAAATTGATAAAAGTCGTCAAAAGCTCATGATGATTCATGTTGTTGCCATCTCTGGTATGATTACCACCATCATTGGGTCATTTCAAACTTTTGCTCAAATGAAAGGTGAAATGGTGGTCGATGCGTGGTTACCCTTTGACCCAAGGAAAAATATGTGGACAGTGTTCATTGCTGGACAGATTCTTGGAGTTTTATTTGTGGTGCCAGTTATTTACAGAGCGATTGCTATCCAGGGAATAGTGTGCAGTATAATAATGTATATGTGCGATCAGCTGATTGAGTTGCAGGGCAGATTGAAGGCTTTGACTTACTCTGTGGAGAATGAGAGCTATATGAGGGAGGAATTTAAAGATATTATAAGGAAACATATTCGACTGATGGGATATTCTAAGTCACTAAAATCTGAATTTAAGGAATACTTTCTTGTCCAAAACTTGGCTGTCACTACAGAATTGTGTTTAAATGCTTTAATGGTAACCATAGTTGGCTTAGAACAGAAGAACCATCTCATATCGTTTATGGCTTTCTTGATAGTGGCGCTATTTAATGCTTACATTTTCTGCCACTTGGGCAACGAATTAATGGATCAGAGTGCAGGCATAGCGAATGCTGCGTACGAGTCGACCTGGACGTCCTGGCCGATCGACATGCAGAAGGACCTGCTGATCATCATCACGGTCGCACAGAAGAGCTTTAAGCTCAGCGCTGGGGGGTTGGCTAATATGTCTATGCAGACTTTTGCTGAGGCCCTGTACAACGGTTACTCAATATTTGCTGTTCTTCGGGACGTTGTAGATTAAAGGATATTGACCAAAGCCGGGCCCAGATTGCGTCTTTGTAGTTTATATTTCAAAAATCTTGTTTTGAATTATAATTGCACTGGTATTTTTGAAATAATCTAACGAATGGGGGTTTAAAAGTAACAATTAAATAGTTGCTATTTGCATCTTGTATTGATAACAAGTTTATGCAAATTAATGCATTTCCGGTGTAGTTTTCCGAAATGAGGCAAAATCTAAATTAACTGAATTTGTAAACGATTTAGCGCACGCCAAAATCGATATTGGTTCAACTCGGAACAGAGCGCTATCCCGATATGTCATTGTCATTTACGTGTCAGTCAAGGGAAGCATTATTATAACCTCAAAATGCCTAATTTGCCATTGAATTCCTCGTAAACCCCTGATTATTTATCAATAATATTTTTTGTGTGTATTCTTACAAATATTTATATATTTTTTCCGAACATTTTGTAAAGACGCATGCTGACGCAAAGTGGGCCGGGTTTCTATAGAGTTTTGGTCAAGATCCTTTCAGTCTTAAAATGATTTAACATACATTGTTAAACATAAAAGTAGTTTCTTAGATCTGCAAAGGTAAGTTTTTGATCACGAAATTGCTTTTTATGGGCTAAAATTCTAAAATACCATTCTATACAGTGGTGAGTGCAGAAAATATAACAACAGTTGGTATTCCATAGTCAAAAACATAGTTCTTTTTATATTTAAGTATCTTAAATTGCCAAATTGCTAGCTATTTATATTTACATTGCCAAATATATTTTTTTAATATTATTTAAATTGGTCTAAAACAAAAACATCAATAATTGCACTGACACTGGCAAATGTAAAATAAAAAAGTAAAAAGCAATACCGTACTAACTATAAAGAGTGATAAGCCGGAAAATTTGTTATTTTTTCCAAGGTCTATAGAATGAACTAGAAAATATTTAAATCATTTACATTGTTTTTCGGTACTAAAATTAGGTCCAGTTTCAACTTATTGCATAAAGATTTGTATTTTATGAAGATGCACACTGTATTCAAAGCCCCTGGACGAATGGTCATAGCAATTTTTTTATGTTAGACCGAAAATATGGCACGTTTGTGAGTTTGCCATATTTTTAATGTATTTTTTTGCCATTTTTTGTAATACGTATCTTCGTTGCAGGCCTTGGAGTAATTTTGAAATGTAATCCTTCTTCTTTGTAATTAGCATCTCATATACCTAATAATAATATTGAAAAATATATTTGAAATTGTATTGTAATACATTGAAGCTGATATTGTTTGTAAACACGTATATGTTTTCCATTGATCGCTCCATTTACAAAGCCACAGCGAGTGTTCAAACTGCACAATCTGCACAAACTAATTGTGCAGGAATTTAACAAGTATGACATTTGATTAAATTAGGTGTAGGTTTGCACTATAAGAGCCGACGAAATGTTATTATGATTGAGTTTGCGCTTCCTGGGGCGGCCTTTTCGGAGATTGGCCACCATTGGCTAGATGTGGCAAATCTATATATTTTGCAAATGTTTCTTTTATTCCAAAAACAATCTATTCGTAGGTACAAACAGAAAGATATCAAAAAATGTGTTAATTTTAAAGGTAACAAGTCATTGAATAAACGTATTCTAAGTAGCAGTAAAGATTCCGAAC

>OR6 [Moltype=mRNA] [Organism=Loxostege sticticalis], complete cds

GGCAGACGTATTAGCACGCAAGTCAACGGTTGCAAAATGAAAATTCTACCAAAGGGAATTATTAAAAAATGCACCCAAACCGTCGGTGTTAATAACGAAATTGATGAAATAATGCGCCTTACGCTCTTTTTTACGAGGATTTTCGGTCATCACATTTTAGATCCAAATTGGACGTGGAACATAACTTTTCCATACCAACTGGCAACATTGCTTTTAATATCCTATGTTATTGTCGGTACTTTGGAGATTATTCGCGGTACAAACGACGTCAAACTAATCGCTGAAGCGGCGTACACCTTCATCGTAATCGTGGTGATGCAGACCAGATTCTACTTTTTTCTCTCGACGCGAAAGCATTTTCAGCATCTTTACATTCAGATGAAAACAACTCTGTATAATTCCATCTTAGATGACCCGGAGAAAAATTTGAAAGATGTTTTAAAGAAACTGAGAATGGTGGTCAATTGGATGGCGTTTTTTTGTTTTTTTCCGGTGGTGATTTATATTCTCACGACGCTATGGTGCTATTTTAACGGAGAGAAGAGATTAATGTCGAAAACAACGTCGATTTTGATGCCAATGAGGACGCCGTACTATGAGATAGGTCTACTATTGCACAGCATTTTTATGTTTTCAGCTGCGTTCACAATAGGAGAGGTCGAAATATGGTTTGTGATGATGATGATTTTCTTCCGTACGGCTTGCGATGGTACGGAAAAATATCTAAGCGTTGAAGCTAGACATGAAAACGAGAGCCAACATGATTATGCGATCCGCTTAAAGAACAGTCTGAGGAAGTTCTACAAATCTCACGTAAAAGAAATCGAGTTTCTAAATACGTTAAACGCAATGTTCAAGTGGTTAGGCATGATGCCTCTCATCAGTGTGGCGTTATGCATTTGTCTTATACTTCTACTATTGAGTAAGGGAATAGACATGACGTTTGTATCCAATGTAATACCAGTTATCGTGGAATTGTTCGTATACAACTGGTTTGGAGAGGAAATAAAAATAAAGGCAGAAAAATGGAAATCGGCTATTTTGGAATTCGACTGGTTGAACTTGCTGCCAAAGGATAAAAAATGTTACTGCATATTGGTGTGTTATATGTCGAAGGAATTTGGTATTAAAATTGCGACAGGAACTTTCCTATCACTTCTTACAATGAGCACGTCGCTCAAATTCAGCTACCAAGCATTCACCGTACTGCAGACAATGGATATATAAAATGCAACATGTTGGATAGAAAACCAAGATTTTTCGCTCGTTTTGCTTATTTTTCGTGCCATTTGTAACACGCCTTAATTCTAGTACAATAACATATTACATGTATCACTCACGGC

>OR7 [Moltype=mRNA] [Organism=Loxostege sticticalis], complete cds

TGAAATGGGGTGGGTATCTACATAGTAGTGGAGAAGCCGTCAGTCATACAAAACCTACTCCTTTTCCTACTGTTGCTAGCAAAAAGTGAAAAAATGGCCATATTTAGAAAAGACATTCCAGCATCTAAGATGAATCACCAAAATTTCACTTTCGATAAGATATTCATAATAACTGCGAAGGCTATGATTTTAAACAGATCGCATCCATCCATACCAAGGAATTGGTTTTGGGTTTTCCAGTTTCTCGTCATATTGACCCTTTCAGCAACCACATTCTTGTTTCTTATTAATTCTGTACTGTTCTACGATATTCCAGCCAGAAGATATGCTGAAGCCAGTAAGAATAGTACTATGGCTATCGTTGCCTTCACTGTTACAATTAAATACTTATTTATGTTATATTTTCAAAAATACATGCAGGACTTAATCGATGTTGTCGACAGAGATTTTAAATTGGCTTTGGATTTCGAAGAGGAAGAAAAAGAAATTGTAATCATGTACGCCAAGAAGGGCTCAAAAGCAAGTTGGTACTGGTTGCTGGCTGCTTCATCAACCTCAAGCCTGTTTCCCTTAAAGGCTTTGTTGAAAATGGGGTACTCATATTGGAAAGGAGAATTTGAATTGATTCCCATGTTTGATATGAGGTTTCCTGATAGAGTGGACATAGTAAAAGAAATACCAGCCGTGTTCGCATTCTATTTTGTTTTATGTTTCATGTTTAGCTGCTATGCTGGATCAATGTACATTGGATTTGATCCGTTAGTTCCTATCTTTTTGTTGCACATTTCTGGACAGTTGAATATACTCAGCAAACAAATTATGAGAATTTTTACAGAAAATAATAGTGTGGATGAAATCAATGAAAAGTTGAAACATGTGAATATAAAGCTACAGGATTTATACTGGTTGATTGAAAATATAAAAAGCAAGTTTACTGTATTATTTGAATACAATATGAAGACTACAACTTTCCTATTGCCACTGGCCTTATTTCAAGTTGTGGAGGACTTAAAACGATCACAATTAAATTTGGAATTTATATCATTCTTCATAGCAACGATTTTGCATTTTTACATGCCGTGTTTTTATAGTGATAATCTTTTAGATCAGAGCAACTACCTACGTGAAGCGATATATTCCTGTGGTTGGGAGAAGCATTCAGACACTCGGGCTCGGAAGACTATCTTACTGATGATGACCAGAACCACGAAACCCTTGGTCCTGAGCACTGTCTTCTATCCCATATGTCTCGACACTTTTGCAGAGATGTGCCGTCAGGCGTACGCCATATTTAATATCATGAGCGCAGCCTGCGCTTAAAGCTCTCAAAAATTTGAGTATGTGTATCAATTTTAAATTCCTAATGAAGTCACCTTTGAAGATATTATCATCTATAATACTTACAACACACAGTCGCATATCAACACAAGTAGTACAAGCATTACTTATGATGAATGAATGACACTTTAACATGTATTTATTTAAAGAAATAAAGAAAATTTTGCAGTGTTTCATTTCTAAACTCAAACTGTTTTTAACTTCCAGCTGTTTTGTATAAACAACTTAAACCAAGGCTGTACCGAATACGACTGTATTGACTTGGTATGCCCTTATTATGGTTGCAAACAATAGATTCGAGCGTACAATAAACTCTGGTACCTACATTCAGTAATTGGAATACGAGTTCTGTGTAGTTTACATTTCATTCTCGTACAAAATTGAACCATTGGCCGCAA

>OR8 [Moltype=mRNA] [Organism=Loxostege sticticalis], complete cds

TCACTGTTGTACCTAAGTCATAAACATAGTCACAAGTTACCACCGGATAATCTTTTACTTAAAATGGAAGTATATCATGAAAGCGGTTTGGGAGGCACGGAAAATGCTGAAGATATTTATTTCAACCCATTTGAAAAGACCTTCAAATTTGTGATGTTTTGCATGGTTGTTGGAATGATATATCCTTACCCAAAAATGACTAGGATGTGGCAGATTGCGGCAATTTGCTTTTACTTAATTATTCCAAATCCGTCAAGCTCAATTGTTGTGTATGACGTAATAGAAGCTTACAAGGAAGGTGACATGGACTGCATATTTCGTCACATCATAGTCATGGGCCCGTTTATAACACACTACCTCAAAATGGTATTGATGTACATCTATCGTTCAAACGCCAAATTACTTTTGGAAGAAATGAATGAGTATTTTGAAAAACTAAACTCTAAGCCATTGTCGCATAAGCTCATCGCAAAGAAGTGGCTAACCAAAAGCTTTTTCTTGGAAAAAAGCTGGGCATACTGTGTCTTGGCAGGAAGTTTCTCTTTTCCTATAATGGCGATCTGTAAGAACATCTACAGTGCTTTATTTGATGAGTACCCAAGGAGGTACTGCATCCAAGAACTACGATCACCGTTTAGTGGGTCAAATTTGGACTCTCCATTTTACGAAATAATGTTTATAAATACATGCATGGCCAGTTGCATGTACTATATCAACTTTAATGGATACGATGGCTTTTTCGTTCAACTGATTTTGCATACAGCGTTGAGGATAGCCGTATGCGGCGAATCGTTGAAGGATGCTTTTAACATTGACGACAGCGTGTTGAGGAGAAGAGCAGTTTGTATGGTAATAAAAGAACACATAGCAATTTGCAATTTCATTAATCGTATAAACGTGTTATTTCAACACTGGATGAGCATAATCACTTCCTATATGGTCATCCATTTTTGCGTATGTGTCTTCTTCTTATCAAAGCGTAGCGGGCTTCAAGAATCACAGTTTTTGTGTGCCGCCTTTGCTTCCGTTATGTACCTCTTCATGATTTGTGCTGTTGGTGGGCTGATACAAGACGAATCCGAGAAGCTATCAGACCTTTTTTACGAATGCGGTTGGGAGCGCATCTCGGACCCTGACTGCCGTCGTCTGCTGGTCTTCATGATAGCCCGAGCCCAAAAGCCTTTGCAGGTCCAAACGATTGCAATGTACAACGTTAACCTACAGCTGTTTGTCAAGGTTCTCAAAATGGCGTATTCGTTATTTACTTTCCTTCAACAAACTTAATTTAGTTAATTATATTTCAACCCCACACTTAGCAGATACTTTATTGTATGATTATTCTTGTATATTTCAAGCACTATTACACATAATTATTATTTAACGGCAATAATACACATTGGATGTAAGCGGTTATCTATGGATGATGTTGGTTAGAAACAAATTAATGTGGGGAGATGGGAAGACGGCACCATCATAATTTCAGCCGTTTCAAGTTTATATTAATGAATACTTCTTTGAACCTTCAAACTATATTTAATTTATCGATCTCACTTAATAAAATAACGATAATACACATAATTTTTACTAAATACACATTACAACATAAAA

>OR9 [Moltype=mRNA] [Organism=Loxostege sticticalis], complete cds

GGGACGTATTTATGCTTACTTTACTAGTAAATAATAAAACATTAGTTAACCCTAGGCATAGCCCGCGACTCCGTGTGCGTACAAATACTTTTATTCTCCGTGAGGAACGGTTTTTCAGGATTTTTTTTATTTTATTAGAGTTTATTTTAATCGAAGGCTTACTTTGACATCTTGTGCCGGGAATCGAACTCCGAACTTTAAAATGGAGAATCAATTCAAGCCGTTCCACGAGACCTACAAAGCTGTGATGTACTCTATGGTGTTTGCCATGGTGTATCCCAACCCAGCTACGGATAAGTGGAGACTACTGGCTATCCCCTTGCTTCTCCTCACAATGTTACCAATGTCGGTGATAGCTTTGCTGGATAGCTTACGTTGTTGGAACGAAGCAAACTACTTGGAAGTTTTGCGACATATTGCAATGTTTGGGCCCTTTCTCTGTGGAATCTTAAAGATGTGTTTCATGTACCACAGGAGGGTTGAAGCCAAAGCAATAATAGACACAATTAACGAAGACTACGCCTCATACAACTACTTCCCGGAAAACTACCAAACGTTTATAAGAGCATACATCGAGAATACTAAGATATACCATAGAGTTTGGTACTTTTGTGTTTTATTGATCCTCTCAGCGTTTGTTATGACCACAACTTTATACAACACTTATGAATATATGTTCAGATCCGAGCCAAAACGACACATGATATACGACATACGCTTACCAAATAAAGCAGCGGGGGTCGAACTTGAGACGCCATACTTCGAAATTCTATACATGTACATGTTATATGTCGCTCTTATATTCTACCTAAACTTCACTGGGTACGACTGTTTCATGATTACTGCTGTTAACCACGCATGCTTGAGGATCGAGCTGTTCTGCAAACACCTGGATGATGCTATGGAGTTCAAAGGTGAAGAATTGAGGAGGAGAATGAGAACAGCCATAAGTGAGCAGTGCGAGACGTTCAAATTGATCGACGCCTGTCAATCTACATTTAATGGTAATTTAGGTATGGTATACCTAGCGGTCACCACTGAACTATGCATTAACCTTTTTTTAATGACCGAGGGCTACGAATTTGACTACAAGTTTACAGCTTTCAGCATTGGCACCATTCTGCACGTTTTTGTTCCTTGTCGCATTGCTGAAAAAATGAAAAATGTTTGTGAGGAATCCTCAACAATGATTTACTGCTGTGGATGGGAGGAAATGTATGATCTGTCCGTACGCAGGTACATCCCCTTCATGCTTGCGCGCGCGCAGTATCCTGCCACTTTGAAGGCGTTTGGGATCCTCACTTATGATATGATGCTTTTTGCTTCTACCGTGAAGACAGCATACTCACTGTACACGATACTCAAAAGGCAACAAACATAAAACCATTTTTACAATATCTAAATCTGTTTTAGTATAACTTACCTAACCTAAAGATATCTTTGTGTAAGGCTGCATTTTCCGTGGACAGGGGAACTGCAGAAGCCATTTATTTACTTTCAATATATTTAAAATATTTTTAATGGAATTTCAATAAAGGAATTTTATAATATCTTTGATCTTGTCTGTGTAGCTTTAAAACATTATTAATGGAGTTATAATCTGCAGCACTTCAATAAAGTAAAATAAAAAAAAACCAA

>OR10 [Moltype=mRNA] [Organism=Loxostege sticticalis], complete cds

GTTCTTTCCTATGATAGGTTTATGATGTACAAACATCGCAGAGTCCTCGTGGCGATCAGATTAAGTGCAGTAGGTTTTGGTTTGAACGGACACGCTCTTGCCCCACCCCTTTTGACCACTAGTCTAAAGAAAATTCAGGTCAAAAATGTCTGAAGCAACGCTTGCTGAGGCAAAACGGGAAATAGCAGAATCATTGACACTAAATACCTTTTGCATGCGGCGCATTGGTTTGTCTTTTGAGGAGCCTAAGAATGCGTCGTCATACTTGGCACAGAAATTTATGTTGGTATTGTCGGTGATGAGCATCTGCTACCACGTTTTCAGCGAGATTGTGTATATCGGCCTGACGCTGTCCAACTCGCCTCGGGTGGAAGATGTTGTTCCGTTATTCCACACGTTTGGATATGGTGCTCTTAGCATCGCAAAAGTATTCGTGCTATGGTACAAAAAGGACGTGTTCAAACAACTGATACATGAACTAGCAGGAATCTGGCCCATGCCTCCGCTTGACGACGATGCTACGGCCATAAAATTAAAAAGTCTTACCGCGCTTCGTATTGCGCATCAATGGTACTTCGCAGTGAACGTACTAGGGGTATGGTTCTACAACCTGACTCCTATCATCGTGTATACTTACCGTTTGTGGCAAGGACAAGAAGTGCAAATGGGCTACGTCTGGGTCTCGTGGTACCCCTTCGACAAATACAAGCCGTTTGCACACGTCGTCGTTTACATCTTTGAGATATTTGCTGGACAAACCTGCGTCTGGATCATGGTTGGCACTGACCTGCTGTTTTCCGGAATGGCAAGCCACATTGGTCTTCTGCTACGCCTGCTGCAGCGACGCCTCGAGACGCTGGCAACTCAGGCACAGACTGAAGAGGATGATTATAGAGAGATTTTAAATAATATTAAGCTTCATCAGCGACTCATCACGTACTGCAATGACTTGGAAGTGGCATTCTCTCTCTCTAATCTGGTCAACATTGTCCTTAGCTCCGTCAACATCTGTTGCGTCGTATTCGTCATTGTATTGCTGGAACCGTTTGTAGCCATCAGCAACAAGCTGTTTTTAGGGTCAGCCTTGATTCAAATTGGAATGTTGTGTTGGTACGCGGACGACATCCTTCACGCGAACGCGGACGTCGCGGCCGCAGCTTACAACAGCGGCTGGTACAGAACCAGCGCTCGATGCCGACGCGCCCTGCTTTTCCTTATCCAGAGAGCTCAAAAACCAATTGCATTTACTGCTATGGGTTTTACGGACATATCCTTGGTAACGTATTCATCGATCCTGACTAGATCTTATTCCTACTTCGCACTTCTTTACACAATGTACAACGATAAGTAAATTTATAATAAAACAAGGTACAGAAAACTCTTAGCACTAACCTGCTTCTTCTTAACATTTCTCTTC

>OR11 [Moltype=mRNA] [Organism=Loxostege sticticalis], partial cds

TAGATCTTGCCATCACTACAAAAGTCTGTGCAAGCATTGAAATTAACTTTACTACAATGGAGGGCATATACCCCGAGGAATTCATAAATTCGGTACTTAAAAGTTTGAGCTACTTTAAAAAGTGCAACATTGATGTTTTCGATTCAAAAAATAGCTTGTATCGGAAATTCTGGTGGTTATTCAACATACCGAGTTTTATTTTGAACTACATAACTTTGACGATGTACATTGTAAAAATCTTTACGGAAGGCGTAGACCCATTTGAGAAGATTTACATGATACCGGTGTGGCTAGTCACAACTCAAGAATTTTTTGTGTGTATTATAATAATTCAGAAGGAAAAAGAAATAAGGACAGTCATCGAACATTTAGGTTCCATATGGAGAACCAAAGACCTGACAGAATATCAATCAAACCATAAGAAAACTACAATGAAACAACTAAACTTTGGACAAAAGATTTTCGAAATAATGTCTTTAATAGTAGCCTGGCTGTATATGCTGATGCCTCTTGCTGAGACGTTGTTCAGGAAGTTCATCTTAGACCAAGAAGCAGAGTTGATGTTGCCGTACGCTTCCGTCTACCCTTTTGCAGTCGACAGTTGGGCTACGTATTTGGGGGTTCTTGCTTTCCAAATATATAACATGTTATTTGTGATCTTCATGTACTTGGGGAGTAATTTACTATTAGTTTCTCTGAGCACTGGTCTCAGCATTCAGTTTGATTTACTTCGAGCGGATTTGATCAACATAAAGCCAACAAATAACAGAGAAAATATAGTTTTTGAAATTAATGACGAAAATGTTAAATGGAGTGCGTGTAACATTGAAGAATTTGTCAAGTTTCATCAGGACGTCATACTGTTGACTCAGGAGCTAAACGCAGTATTCGATAAAATTGTATTCCTAAGCTTG

>OR12 [Moltype=mRNA] [Organism=Loxostege sticticalis], complete cds

TGGACACAGAACCAATTAAGAACTACACCCATTTCTTGGAAATACCCTTGAAGATCGTCGGATGTTGGGACTGGTTCGAGTCTCCGAAGAACGAGAAGGAAATCATCATCAACAATGTGTATTTCTGTCTTGTTCTGTTTGTTCTTATCAACGTTCCAGTCACTTTATATGTCCATCTATACACAGAGTGGGTGGACGTCATGACCAGCTTAGACAAATTAGCAGATTGCCTGCCTTTCCTAGTTTCGATCATAATCGTGGTCTACTTCGGCTTGTACAGAAAGGAAATGTATGATCTGACGAAGTTTATGCAGCGCGAATTCAAGTACCGCTCAGCTCACGGCTTGACGAATATGACGATGCTGAACAGCTACAAGACTGCAAGGAATTTCGGCTACTTCTATACAGCTTGCACCATGTTCAGTGTCACTATGTATGTGATACCCGAGATTATCAACCGGTGGAACAGACAACCGTTGCAAAGCTACATATACATGGACGTGGTGCGGTCGCCGTTCTTCGAGTTCACCTTCTTGCGGCAATGCGTTGCGCAGATGTTCGTTGGACTTGCGATGGGGCAGTTTGGCGTATTCTTCGCCTCCAACGCCATCCTGCTCTGCGGCCAGCTAGACCTGGTCTGCTGCAGCCTTCGCAACGCGCGCTACACGGCGCTCCTGCGCTGCGGGATCAAGCACAGCGTCTTGGCTGCAGCCCATGGTGATATACAGGGCGACGAGCTGTATAACTACATTTACAACGCAGCGGAGTTGCAGCCGTCGCGGTACCATTATGATCAGAAAATGAACCATAACATCTTGAATACAAAAACATCATTTGATATCTATAGCCGGGAGTTCGATGAAGCCACGTGCGAAGCGCTGCGCGACTGCGCCCGCGTCTCTGACGTCATTAACGCGTACAAGGCCAAGTTCGAGCGCTTCGCGTCACCGCTGCTGGTGGTGCGCGTGGTGCAGGTCACCATGTACCTGTGCATGCTGCTGTACGCCGCTACTCTGAACCTGGACATGGTGACAGTGGAGTATTTGGTGGCTGTTATGTTGGACACCTTCGTCTACTGCTTCTATGGCAATCAAATTATAATACAGGCTGACAGAGTATCAACGGCGGCGTACCAAAGCGCGTGGCACACGATGGGCGTGCGCCCGCGCCGGCTGCTGCTCAACATCCTGCTCGCCAACCGGCGCCCGGTCGCGGTGCGCGCCGGCTACTTCCTGCCGATGGACCTGCACACTTTCTTAGTGATTATCAAAACTTCGTTTTCTTACTACACTCTGCTAGTGAATGTAAACGAAAAATAGTGTTATTACTTTAACAACCACTTTTATTAGCTATCAGTAAATAAATAGCAGGCACGAGTTATTATTTATTGTCTTTTCAAGATAAGAAGATATC

>OR13 [Moltype=mRNA] [Organism=Loxostege sticticalis], partial cds

GAGCCCCCCCCCGTCCTTGTCACAGGTGGTCACATTTCTGAGACCCCCCCCTCCCTAGTGTGACATCACATGTTTTGCAATTTAACATCGAAAAATTATTAAATTAAAACAGCAGTTCCGAAATTAGTTTTATTTCCCTGCAAATTATATGTTATATTGTAATAAAATTCCAATTAATATTATTATATCGTTACTTTTTATACCGCGGTCTTGAACCGCACCATATTAGAATGACTTGTATAAAGTCCTATGAAACCGTAGGTGTAAAAACAAACACATAATCGTAGTACACGCGGGCAAAACTACGAGCGGAAAGCTAGTCTTAAATAATTATTAGTACCTAATGCGGCGCGTGCGATGCAATTCAAGTCCGCGTCCATCTTTGACTCCTGAGCGCGATAATTAAGGATTGTAATCAAATTCGTAATAACTTACTTGTACTTTTCCCTCCTTTTCCCGCTCTAGTTTTATTCGTGATGTTGACGCCGAAGGAATTTTCGAAAAAAAAATTCGTTAGTGTCGCTTTTTTTTTCGTCATTTGTATCTGCAGCTCTTTGATTTTTAATTTCACATCACCTTCGATATTGTCACTACTTTTCAAAATTGTCTTTAATACTTTTCGTATTGCCCTAAAGATAGAGTTGGACATATTTTTTAGTTTAATTAATATTTCAAATTACGCGATTAGTACCTCTAGATTTTGAAATGTGATGTCACGAAACTTAGGACCCCTCCCACCCCTTGTCACACCATGTCACACTTTGTCGACCCCCTCCCCCCCCCTTTACGTGTGACGTACTTTATGGATGGCCCCTAAGCCAAAACTAATTGAAAACCATTTTTTTCTGAGAAATTAATCAGATTGTTCAGTTATACATTTTCTTTTGAAAAAACTTAATTGCTTCCTTCTCACTGCCTGGAAAATATAAGTCAATAACCTACTGGACTTCTATAGGGCCTCCTCAAGGGTAATAAGACCAAAACATCTTCAACAATGTGGGAGAAACTCCGAAAGTTCGGTCCGGAGTCCTGCGACTTGCCGACCATGCTGTCGAACGTGTGCTTTCTGCTCAGAGCTCTAACCCTCAACTTAGATAGCAGAATCAAGGACAGTAAGTAAAGTCTAAGAGGTTCTGTATCCTTTAGTGCTTTAGTATTTTCATCGCATGGAGAGAATGATCCGCCATCTTTAAACACAAGCGGACGGTGCATGCAGCGGGGCGTTATTTTGAACGTGTAAGTTGGGCTACTGAAGAGCTAGTAGCCTTCCCTTGAGGTGTCTTGATAGTAGCGTTTTCAGCGCGCAACGAGCATTCGAACCAGTGGACGAAGTGTTGTAGTAAGCGTGAGCGCTGGTATTCATATAATGACCAAATAATCACGACGATTTATAACAAAGGGAGAACTAGTAGCATCACGCTGGATGCGGTATTGCAACCTTACGACTCTTGAACGCCCCGCTGTCGTTCAGAATTTGCATCGCAAGCTTTTCTCGATGTTCAATTGTTCCTTGATGGCAGTCGCTATAGATTCTAGACACAGAAGTTAAACGTTGAATAATTGTAGTAAGAATCATCCTGTCGAAAGCATCGTCATAAAACTGGACATGATATCTTTGGATTTTTTAAATTATATACTTTACTGACTTCCAGGGATCCCCATAAACTGCTACGGGTTTACAATCATCATTTCTTCATGCTACTTTTATGTATACCTGGTATCCATGTGCTGGTTCGTCTTCTGCAAGTGTCGGGAGACTGGTGATCTGATCCCAGCGATGATTGTCTTCTCCCTTGGTGTCAGCAGTGAGATTGGACCTTGCAAGCTGTTTTATATGTTTTTGTATGAGTAAGTTGACATTTCCTCCTCTAGTTACATTCCTCTTGTTTGTTCTGCGTTCTCCTCTTACCAGTAATTTTCTTAAATAATATACTTTCAGGAAAGTAACTAGAACTATCGTTGACGGATACTTAGTTTGCGATGCTCTGACCCTGAAAGGCGAACGATTCACTAAGAATTTGCTGAAGACCCTGAAAGATGTCAAGAAACGTGCCCTGATTTTCTGGGTCGTCATTATTGGAAATGGAGTGATCTACTTTGTTAAGCCCATCCTGCTACCAGGGCGGCACCTCATGGAAGATCAATTCATTCTTTTAGGACTAGAACCGACATTCGAGTCCCCCAACTACGAAATAGGATTCGTTCTAATGAGCTGTGGCGTCATCTGCACCGTCTACCTCCCTGCCAACATCACGGCCTTCCTCATCGTTCTAACTGGTTACACAGAAGCCACTATGCTAGCCCTTGGAGAGGAGCTAATCAACCTTTGGGCCGACGCTCAGCGCTTCTACAGAAATAACCACGTGGAAATTGACATCACTGATGAAAATGCACTGGTCAACCTGACAGATGATGCTGAAAAAAACAGAATCATGAACAGATATATCAAACAAAGATTAGAAGAGATAATCAAAATTCACACAAGGAACATCAATCTCATAACCCAAATCGAGAAAGTATTTCGAGGGGCTATAGCTGTAGAATTTCTTCTACTTATTACTGGTCTGATAGCTGAACTTCTAGGCGGATTGGAAAATACCTATATTGAAATGCCTTTCGCACTGATGCAAGTAGGTATGGATTGTCTCACTGGTCAGAGGATGATGGATGCATGCGTCAAGTTTGAGGAGTCCGTGTACGATTGCAAATGGGAGAACTTTAATGTTTCTAATATGAAGACAGTCTTGTTGATGCTTCAAAACTCTCAGAAGACCATGGTCCTGTCAGCTGGGGGGATGGCTACGCTGAGCTTCAGCT

>OR14 [Moltype=mRNA] [Organism=Loxostege sticticalis], complete cds

GCAAGAGTTAGGGGCACGATGATTAATTTCAAGTCTCTCTGGACGAGGCTAACCCATACTAAGGCTCTGGAAAAATCCAGCGGAAAACTGGAAACTCGCTTCTTCGAAACCGTTTATCGGGTTTCTTATTTGACAGGGATATCAGCAGCTGACGATGACATCCCGTACATGATCTACAGCAGCACTGTCAAACTCCTCATAGTGCTGTTAGTCTGTGGGGAGATCTGGTATGCCTTCACCGAGACGTCGAGCCTGGACGAGATCGCTGCCAGCATCAACACCACGGTGATCCAGTTTATCACCATGTACAGATACAGAAACATGATACGTCACAAAGATGTCTACAAAAAACTAGCTATGTCCATGGAGTCGCCGTTTTTCGATATTTCTACTCAGGAGAGGAGAAATTTGGTGGATTACTGGGTCAAAAAGAATGAGAGATACTTAAAGCTGCTTCTATTTTTGGGAAACTGTACATTAGCTGCTTGGTTTCTGTACCCATTAGTGGACGATCTCGAATACAACATGTTCATCGGGATCCGCCTGCCTTTCCAGTACCGAAGCCTGATTCGGTACACCTTCGCCTATCTTGTGGTGGTGATGGCTTTCGCCTACATATCCCACTTTGTGATGGTGAACGACCTGATCATGCAAGCCCATCTGCTGCATTTGGTTTGCCAGTTTGCGGTGCTGAGTGACTGCTTTGAAAATATTTTGGCCGATTGTGAAAAAAAGTTTAAAGGTGCCGACAGAGAACGACTAATAGCAAATAAGAGATTCAGAGAAGCATACAGAGTGAGGCTGGGCGATATGGTTAACCAACATCAATCTATACTTAGCCACGTCATGGACCTGCGTCGCACGCTAAGCGGGCCCATGCTTGGCCAGCTAGCGGCTAGCGGAACTCTCATTTGCTTCATTGGCTATCAACTCACTACGACGGGCGCCGACAACGTAACCAAGTGTCTGATGAGCCTGTTTTTCTTGGGATACAATTTATTTGAATTTTACATCATTTGCCGTTGGTGTGAGGAAATCACAGTTCAGAGTCAAAAAATAGGCGAGGCAGCGTACTGCTCAAACTGGGAGTGTGGTTTGGCCGACATCCCCGGGGTCAAGTCGTGCCTGGTGCTGGTGATCGCCCGCGCCAACAAGCCGCTCGTCCTCACAGCTGGAGGGATGTACAACCTGTCCTTGCTGTCTTATACCTCTCTGGTCAAGACATCGTACAGCGCACTCACGGTCTTACTTAGATTCAGACAAAACTAATTGATGATTGACGAAGTAGAATTCTAGTGAATAAGTGACACACGCACGCAAGTCAATCGGACACGCTTCTTGATCGCACTGGAGTAATTAGAATATCGTTGAGATAAACACGACTGTCAACGCAAGTACCGAAATGGACACTTGAAATATACAGGCATTGTCATTCAAATGGGCGTTTGTCCGAACTTATTATCATACTACAATATGTTTTCATGTCATCTGCTTTCTGGAATTAGTCAACGTCGCCGTGTATACGAATGATAATTATAAATACGAGGAAGTTGTCCGTATGCCCAAAACTGAGTGAACGAAGTGAGCGAACGCACTGAGTTTGTGGAAGCGAAGTTATCAAGTTATCAAGATGAACCTCATGGGATATTTGATCACCCTTGTCAACCTTCAGAGCTTTGACAAACATGCAGGACCTGTGCAAGATATTGCTGTAAAAGTGGCCTCGATAGAAACTCTCGTGAATGACTTGAAAACGGCAGGCGTGAACCTAGAAGGATTCCGATCTACCGGATACTACCGGACTGGTAATGTCGGACAGTATTTGTCGCATAACCTAAGGGACTACATTTACAAAGGTGAAGGGGACCCTAAAAACGAAGGAGTTGTCTTGACCCTCATCTTCAAAGATAAACTTGGCGACCATCCATCCGCTGACGATGAAACCCTGTCCTATTTCCTCCATAAAAATGTTGGAGACGAAGATACTGAGCGAGTGAAAACCTATCTGGATGGACGAAATTATGGCTGAGAAGGTCGGAAACTGTAAAACGATCCCGTCATAAAAGTGCTAGATACATTTTATCTTATCTGAGTCATTCGTCAACTCGCGCCAGTAAAACTTGCGCGCGCTCGCGAGTTTTTCCCGCGCAACTTGACGAATGACTCCCTTAACAAGTAAAAAAAAACTGGCGACCTCCCAGTTTATCTTGAAAACGACGTCAGCGACTTAAAGTGACGTGTTTTATTTGACTTTTAAACCTTACCTTTTTTTTTAATTGGTTAGCTCGCACTCGGCACCCCTGTGAAAACACTGTTAAAAATGAACTGGCGAGTGAACGCCATGTCCTTGCAAAATGGATACTAATGACAGATAAGCTTGTTTTAACACTCTTTTAAGAGATGGCGTTTACCTCTACTTAGACTGAATTGTAGTACACAAAGTTAACAATTAACTTATTTTTGGACCCTAGAAGTAAAAAAATATCTAAATAACTTTGTTCGAAACATACACGATTAACTATTATACAAATTTACACGTTGTTACTCAAAATGCGACGTATAGATTCCTAGATTTTGCGTCATGGCTCATAAGACACAAAAGCTAGAAAAACAATAAAGATATCTAGGGTAGGTAATAGTATATCTATATACAGCTTACGAAATTAAAAACAATTCTATGAGATAGTAAATTAAGCTACATAGACTCTTATAATACTATACAGTCGATTACTACCTACTTAAGTATAGTTTTCTTCAACTAGAGAGCGAAGGGACCATCAAAATCTTAGAAGTTACTCTCCTCTACCACCCTGATCAGGGCGGGTATCTGTCGCTATAGCGGCCCGGTGACCTGCGTTGTGGGCTCCAGCTTGGTCGCGAACTTCGCTGGTTCTTCAGCGGCTGACAGCATTCGGCGCGCAAGACGGGCGAACGTGCAGCGGAACTGCCGGCTCATGCTGCAGTACAGGACGAAGTTGACGGACGACGTGAACAGCGCTAGCACGTCCATCAGGTCGCCGAACATGCTATAACAGTTCTTGAAGAAGTCCGGCGCAAGCGCACTGGCGAGGCCCAGCAGACCCTGCGGCACCTCGGTGGACAGGAACAAACCCAGGACCGCGAGAAGCATGCGCGTCGTCCGGTCAGTTCTGCTGGAGCGGCGCGCGCATGCCTCTTCAGCCAGGCACTGCTTTTCTGGCTCATTCGGTCCAACGGTTGATCGCTTGAGCAACTTTTGCCTCCTTCTCTCCGTCGTTGTTAATTTTGATATTAAACAAGTACTTAATATAGATAGAGCTATACTCGGGACTAGTTTTAATATAACACTGTATATCCACATAATTGCCGTAACCAACTCAACGTTTTTAGACATTTCTAGCACGTACACGGTCCGGTTATGCGGCAAGGCTAGGTCTTCGGCGAATTCGGAATTATTCTGCGGGGTCACTTCACTCGGCACTATAGTCATCGCGAAATAAAAAGGAAGACACAAAAACGGGCATATCACATACGCACTCACAATAGCTAATGTTGTATTCTTTTTACTACACCAAGTTCTGTTTCTCTGCGGATATGCGATAGCCACGTACCGCCATACGGCTAACGTTACAGCCAGCCATATGGAGATCGTATGAAAGGTTTGACTGAAAATGGAATGGAAGTAAACGAATACGGCCCACGCATAAGTGTTTCTGTTGACTTGAGGTCCGATTTTGATGTTCATGTGCAGAGCGTAGGGTATGTATTCGAGCATGACGAGGAGATCGGCGACGGCGAGTCCGGTGAGTATGGAGTTGGTGGAGCTGGTCATTTCGCGTCGGGAGAGGACAGCTATGTTGATGGAGTTGGTTGCGGAGCCGACGATGCAGATGACGAGGGCGATGTAGCCGTGCATGCGGCTGTAGGCCTTGTTGAAGTCGGTGGCGCCGGGCACGCAGTACGCGTCGGTCGCGTTCGACATGCTCGCGGTCCACGCTCGCTTCGCTTAGTGTTGTCATCGGCGCTGGAGCGAGGTCAACATCATCCCGACATCAAGCCATCGCGCGTCACGCGGCATCGTCACAATATTTCGTGAAATCCAATCAGAGTACTGTCCATTGCGTTAATTTGTGGTAAGTGTTATCCGTGCCTGATAATAATTACGCGACTACGAATTTCAGCGGTCGAAAAACGTCGCCACGTGCACTGAACGCCGAGTCG

>OR15 [Moltype=mRNA] [Organism=Loxostege sticticalis], complete cds

CGACATTCGTTGAAAGGGCGCCATGCTTTCTCAGAAGCTAATTTCATTTTTAGAGAGACTTGAAGACCAAAGTCACCCTTTACTGGGGCCGAATGTAAAATGCCTTTACATTTTTGGTCTCTGGCAAACGGTTAAAACAAGAAAAAGAAATTTCATCTACAATATTTTCCACTTCACCACCTTCCTGTTTGTGATGACCCAATTCTTTGACCTTTACAAGCAATTGGACGACTTCAACAAGGCTTTGAATAATCTTTCTATGACTTTCATAGGAGTCATCAGCTGTGCGAAATGTTATTCATATGTATTGTGTCAGCGGCAGTGGCAGAAATTGGCTGCTGATATTTCAGCAGAAGAATTGGCAGCAATGGAAGATGGAGATGAAACTGTTATGATTAAGATGAAAGAATACAAGTTATATTCTCGGGTCATAACATATTTGTTTTGGGTGCTCGTCACTATGACAGATACAGCTCTGATCGTGACTCCGTTGATAAAGTATCTGACGACTCCAATGTACAGAGCGGACATCAGAGAAGGAATCGAAGAGTACCCCCAAATAATGAGTTGCTGGTTCCCTTTCGACTACATGTCGATGCCAGGATACATGTTCTCAACTATGATCCAAATCATTATGTCAATCCAAGGTTCTGGTGTAATAGCTGCCTCTGATGCAAATGCTATAACTATCATGACTTTCATGAAAGGTCAAATGCAAATACTTAGACAGAAATGTATTAAAATATTTGAAAGCAATTCTTACGAGCCCAAAGAAATTTTGAAGAGGATTAAAGAATGTCACAGACATCATACGTTTTTGATTCAGCGTTCCGAGGATTTCGATAAACTATTGTCTCCAGTCATGTTTATTTACGTTTTGATTTGTTCCATGGCTATTTGCTGCAGCGTTGTCCAATTTTTCTCTAGTGGGGCAACAGCTGCTCAAAAGCTTTGGGTAATTCAGTATACATCAGCACAAATTGCTCAACTGTTTCTTTTCTGTTGGCACGGAAATGAAGTTTTTGTCGAGAGCAAAGATGTAGACCAGGGCGTTTACGAAAGTGATTGGTGGAAAGCAGACGTGCGTTTGCGCAAGCAAGTTCTGCTTCTGGCAGGGAAGCTGAACCGTCCGATACTTTACACTGCTGGACCGTTTTCAAGACTTACTATTCCTACTTTTATCAGTATCATCAAAGGCTCTTACAGTTTCTTTACATTGTTTGCACAAATGCAAGAGGAAACCTAAAAAGACAGGATATTTTTTTCTCTATGTGCACACATACACCCCGTAAGGTATTTTATTTACGTAGGTATATTACGCCAATTATGTGACAACCACATGATTAATTTTATGAGCTTCAAAAGAAACACTATCCCCCTGATAATACTGATAATACTTAGTTTCATAATAACATAGGTGTCATACTCTAGTTCTGTAATTGTGTTTGAAAAAATACAAACATTTTCACTGTACACTGTACAGTATACACACCAATAATTTCTTTCTGTTTAATATCTTATTACAATCAAGTTCTACGTGTCAAAACAGTGTGACATAATAAGCACTGTATATTTCTTTTCTAGAAACTTTGCGAAGTGTACAATTCCTTTTGACATGTGTCCACCTCTTGCAAAATGTTTTGCTTTGTAGAAATAAAATTGTTCAGATGTCTGGTCTGTGTGGGTACCTAAAATGCCATTACAATTAAAGGTAGGTACCTAAAATTATAATGGCCTTCTTTAGTTATCTATAAAAGTGGACTTTAGATTTTAAGGCACGTATTTTATAGACTTATTTGATACGTTTTTTATTTAAGTTTGAAATTCCCAAATCAATGATGAACCAAAGTCGTATGCACAAAGTTGTAATTCAATAATAAAAACACGTTGC

>OR16 [Moltype=mRNA] [Organism=Loxostege sticticalis], complete cds

CCTTTGGGGGTTGCAGAAATGGGAGGGTCATTTCAATCTCATTATTCCTTTTGTTTTTTGATCATGTTGCGAAGATTCGTATCAAGTTTAGAACACGAAGATAACCCATTACTTTCACCCACTCTTTGGGGGTTGCAGAAATGGGGGATGTGGCAACCAAAAAATGGTCCCAGCCCGAAAATCAGCAATGCTATACATTTCGCTGCAATTCTCTTTGTGATCAGTCAATACGTGGAATTGTGGCTAATACGTTCCGACCTGAACTTAGCTCTACGTAATCTATCAGTGACGATGCTTAGTACTGTCTGCGTGGTCAAAGCCAGTACTTTTGTGGCTTGGCAAAAGTACTGGCGGAACGTAATTGAAAATGTTTCAAAATTGGAAAAACGCCAGTTGTCTAAAAAGGACAAAATGACCAATACAATCATTGATGAGTACACTAAATACTCCAGACGTGTAACCTATTTTTACTGGACACTGGTAACAGCAACGGTTTTGACTGTCATAGTAGCACCGTTGGTTGGTTTTTTGTCGTCAGCTGAAACGAGACAGCGTATAAGAAACGGTTACGATTCATATCCAGAAATAATGAGTTCTTGGGTGCCTTTTGATAGGTCCCGAGGTTTAGGATATTGGGTGTCGGTACTTGAGCACATTTTGATTTGCTTCTACGGAGGCGGGATTGTGGCGACTTACGACTCTAACGCTGTGGTGTTAATGACGTTTTTCGCTGGGCAGCTGAAGCTTCTGAGTGTGAATTGCTCGAGATTGTTTGAGGGCGAAGAGGAAATGACTTATGAAGAGGAGATCAAAAAAATTAGGGAGTGTCATTATCACCATCTACTCTTGATCAAGTATTCAAAGATTCTTAACGGCTTATTGTCTCCAGTTATGTTTCTTTATGTTATAATCTGTTCTCTGATGATTTGCGCAAGTGCAATTCAAATAACTACGGATGGAACTACAACGATGCAGCGAATCTGGATAGCCGAATATCTCTTGGCGTTGATTGCTCAGTTGTTCCTGTACTGCTGGCACAGTAACGAGGTGTTAGTCATGAGCAATGAGGTAGATGAAGGCGTATACTCCAGCGCGTGGTGGTCGCGCAGCATCCGAGTGCGCAGATGCGTGCTGCTGCTCGCGGGCCAGCTGCGCAGGAGTGTCGTGTTCACTGCGGGGCCGTTCACAGAAATGACTTTACCCACCTTTGTTGCTATATTGAAGGCGTCGTACAGCTATTACACTTTGTTAGTCAACAAAGATGACTAACTTCTAGCACGAATTCTAATAACAAATAGCTTGATTTCACATGAAAAGAAACACAAATTCAATACAAGATATTAGATTGCACCTTCAAGTCACTTACCTACTTACCTTAAGTACTTAGTAAAACATATTTTTCATAAACATTTTAGCACTTGGCTGAAAATAAAGGTTG

>OR17 [Moltype=mRNA] [Organism=Loxostege sticticalis], complete cds

GTTTTGTTTGCGACTTTTGTACTGAACTTCGCATAGTCAAACTTGCCACCCCTAATTTCGCACGTACAGTTTAATAACCGACTTCATACAAATTTTATAACCGTTGGAATCTTTTTTTGACAAAATGACACACATATTTTTAACCCGACAGAAAAAGGCTCTCACACTTCTTGGGCACTGGTTCCCAACGAAAAAGTACAGGATCCCCTACCTCATTTACCGCTGTTTTCTTTTATTCATCCAGTGGAGTTTTCTTTTGTTCAATATCATCTACATGAGACAGGTTTGGGGCGATTTAGAAGAAACTTCTGAGGGATCTTACCTGCTTTTCACGCATGCCACATTAAGTTTGAAGTCAACAATTTTTCTGATGAGCAAAAATAGGTGGTCAAATATTTTGAATTTTATGGAGAGTGATATCTTTGCGGCACAGACTTCGGTGCATGAGAAGATATTATCCGTGGATGCTATGAAAATGTGGAGCGTGTACGCATTTTTCATAACGTCTGCTACGTTCAACTGTATCGAGTGGGCAGTTGTCCCCTTGTTAGACAACCATGGAGAGAGGGTTTTTCCTTTTAAGATATGGATGCCAGCAGACCCGACCAAGTCACCTGAGTATCACATCGGGTACGTGTATCAGGTGATGGCTATCTACATAAACGCAGCTACCTTCCTCACAATGGACTACCTTACTGCCTCCTTGATCATGTTCGCGGCTACCCAGCTTGGGATCATTGAGGAGAAGATTAAACAGATTCCGGCAACGCCTTTGTCCGCGACACTGGAAGAAAAAAACAAATTAATAAAACAGAACAATGAGATTCTAAATGAGTGCATCCAGCACCACCAAGCGGTGATAAGGTTTGTCAGACTGGTTGAAAACATGTTCAACGTAAACGTTTTCTTTCAAATGAGCGGAACTGTTGCTATTATTTGTGTCATTAGTTTTCGAATGACTATTGAGCCTCCAAACAGCATCCACTTCTTTTCGAATGACTATTGAGCCTCCAAACAGCATCCACTTC

>OR18 [Moltype=mRNA] [Organism=Loxostege sticticalis], complete cds

TTGCAATCGTAAAAAGATGACGACTACAAAAGCAGATACTAAGAACCGAGTCTACAGTCATACTGACTACGATGACTCTTACAAAATAATAACTAAAAACATATTATCACTTGTAGGGATTCGAATCGCGCAGAAAGATTCGTCTTTCGCCAGGTTATGCTGGAACATTCTGTATTGGGCTGAATTCGCAAACCTGTTCATTGCCTTGGTTCTGGACGGATACACCGCATGCGATGTGATCAGGAGCAGCGCCTTGAAGGAGGAAAATATCGTGTTCATGATGCTGCCGTGTATGGGGTACTTGGTTATAGCACTGCTGAAGTCCTACAAGACGGTGTACCAGCGCGGAATATACGAGAACTTAGTGTCTGAACTGCGAAGCATGTGGCCCCAAGGATCAGTCACAGAGGAAGAGCATATCATCATCGACAAAGCGTTGAAGGAACTCAACATCGTTGTCAAAGGCTACTACTGGTGCAACCTCGGCCTCTGCTTCAGCGTCATCGGTCCACCCTACGTGGACCTCGTCAGGAGAGCGTTCGGGGAGGACGTCCCTCGCACTCTGCCGTACTTCTACTGGGTGCCGTATGACGAGTTCCAGCCAGTAGCTTACGAGCTTACCCTAGCGCTGCATGCTTGGCAAACAATGTTGACGTTATGGTTCATGCAAGCCGGTGACCTGCTGTTCTGCGCGTTCCTCAGCCACATCACAACTCAGTTCGACCTGCTGTGCCTCAGGATCCAAAGGCTATTCCACGTACCGGTCGACCAGCAGCTCATTGCTGAGTATCCTTTGGGAAAACAAAGTAAAAAACCTTCAGATAATGAAACCTTTTCCCCATTGAATGAAAGTGAAACAAGGTCCAAACAAGAAAAAGAGCTCAAAAAGATTATAGTGCGACATAACGACTTGATCAGATTGTCAAATGACGTCGAGAATTTATTCAGTTTTGCTCTACTTATCAATTTCTTCAATAGTTCTATCGTTATTTGCTTCTGTGGATTTTGTTGCGTCATGGTAGAAAAATGGAATGTATTTATGTATAAGACTTTCCTGGCTACATCCCTTTCGCAGACGTGGCTTTTATGCTGGCATGGACAAAAGTTGCTAGAATCAAGTGCAAGAGTAGCAGATGCTTTGTACAATAGTGGATGGTATATTGCATCTAACCCGATAAAAAAATCTATACTGATAATGATCCACAGGTCTCAGAAAAATGTCTGCGTTACTACTTACGGATTTTCAGTAATTTCTTTATCAAGTTACACTACTATTATCAAAACGGCTTGGTCGTACTTTACACTCCTTCTCAATACGTACAACCAGTAGAATAAACATTCAAATGAGCGGTAGACATGTTAAAATGCACAGGACATTATTTGAAAATATGTAGATCTGCAATTTTACTAAAGCACGAAAAATAATAATACTAAAATAACACTTTTAAGGCAGTTTCACTCACTTTCTATTAATTATACACGTAACTACTTGTAGTACAAAATTTTAGATCAC

>OR19 [Moltype=mRNA] [Organism=Loxostege sticticalis], complete cds

CCACACCGAATCCCAAGATGGATGAGACTTTGAAAGTATTTCACCGAGTCCTATCCTTTGCCGGTATCGCAATTTACGCCAAGGAAAAATGGAATTCCAATCTCTGGCTCAGCTTTCAGATCTTCAACTTCTTAATCGGAACTTTTTGTTTCATCTTCACAACAGGGTTTGTCGTTTCCAACTACTCAGACCTACTCATTTTCATCCAAGCAGCATGTATTTGGACGACTGGAGTCATCATGACGATGTCTCTTGGAGTTTGTCTGATTTTCAGGCAGAAGTTTAGAATGTTCTTATGTGAGATGGTTTTTAAAGACGAGGTTTTAGAGATGCCGCTGATTAGGTATGTCTTGAGGTTGGAGAGTGGAAAGAAACTCTTTGAATTGAAGCAGATGGTGAAGGATTCCCAGGAACAGCTGTTCAGGGTGACCGGAGTGTTGCTGAAGTGTTATGTGACCAGTGTGTGGTTGGTGGCCACTTTGTACCTGTGCAGCCCCATTTATGAGATGTTCTCTAAAGGGGACAAAAGTTTGAGGCTCTTAGCATTTGACATGTGGTTTCCATGGAGTTTAGAGAATTTGAAAGTTTATATAGCATCTTTTATATTCCATGCATATGCTGGATACCTATGTTGTGTTGCTTACCCAGGCTTGCAACTAACAATCATACTGCTGATTGGTCAAGTCATCCGTCAGCTCAAGATCCTGACCTTCATAATGCTGAACCTAAACGATTTGGTGCTGGAAATCACGAAGGAAAAGGATATACGGTGGCAGACTTACTGTACTGCAGTCCTGTCACAGTGCGTGGACCACTATATTAAGTTAAAAAGTTTCAGCAACCGCCTAAACGTGATATGCCGGCCGTTCTACCTCACTCTCATTTTGGTTGCTATAATGCTTGTATGTATGTGCTCCGTCAAAATAGCAGTTTCAGACAAATTATCTCCAGATACAATAAAGTACTATGTTCATGAATTTTGTTTCATACTGGTTGTTCTCATGTTCTGTTTGCTGGGGCAGCAAGTGGATAACGAATGTGAACAACTAGAACGGGCAATTACCGAAAATTGGTACATCTTTGATAAGAAACACAAGACGCACGTGAAAATTTTCAAAATGGCGCTCACTCAGCGAATGCACATTTTCATCTTTGGCACCATCACTCTATCTCTTCCTACTTTTACTTGGTTCATCAAAACTGGTATGTCGTTCTTCACTCTAGTAATGTCAGTCCTTGAAGAAGGAAACTACGAGTGAATTAAAAATAATAAGCTATAAAATGTTTATCCGCATATGTATCTTTGAATTAAAAGAAATCCTTGAAGCTGTTGTGTGATGCATCATTGTCTTTTTAGGTACAGCCATTTTAATTATGTATGTATTACTTTTTAGCAAATTTCTTAGCAAGTATTGTGTAATACACATGAG

>OR20 [Moltype=mRNA] [Organism=Loxostege sticticalis], complete cds

GCGGTCCTAAATAATACTAACTAAAATTGCCAACGTCCAACCCTATTATTATGCATGACAAAATATTCACAACTGCATTTCCACCGCGATTTTCTCACATCATTTATAGTCATTTAGTGGAAATTATATCGTCTATTCAATTGGCTCGCTTGGAACAGTGCGATCTGCATACTTCGAAAACATTTCCAAAATGACGAATACCCGGACTATAGACAAAATAGGTATTCCAACTTTAAATTTCCTCAATTTCCTAGAGGATCCGAGGTACCCTTCGGTCGGCCCGCACCTCCGCCTGCTAGGTCTCACTGGTCTTTGGCATCCGAACCTCAAATCTAGAATAACCCGTTTCAAACAATACCTCTTCTTCGTCACCATCGCTTTCTTCTTGAGCCAATACGTCAAATGTCTCATCAAATTCGATCCCATCTACTTGAAACTCGTACTCCAATACGCGCCCTTTCATCTTGGCATCGTCAAGTCGTGTTTCTTCCAAAAAGACCACAAGAAATGGGAGACTTTGATAGACTACATTTCAGCCGTAGAACGCGAGGAGATATCCGATGGGAAAAGACAGAGCAATGAAATCATCTCGGAGTACATCAAACGTGGTCGGAAAGTAACATATTTCTTCTGGGGGCTAGCTGTCGTTTCGAATATCAGTATTTTTACGGAGCCGTATCAAAAGAATCAGATAAATGTAAATGGTACTAGTGTGTACCTATGTGTATTCGACGGGTACACTCCTTTCGGCGAGGTGCCCCCAGGGTATTATGCGTCCATGTTCATCCAAACAGTTCTCGGCCATATTGTGAGCGCCTACGTAGTGGGGTGGGACACTTTGGTTTGTACCATCATGATATTCTTCGCTGGTCAACTCAAGATTTCGCGGATGAATTGTACAAATGTGATAGACACTGGAAACGCAGACATAAATCATAAGAATATTGTGAAATGTCATAATTTTCATACAACCCTTGTTATGAACCAGAAATTATTTAACTCTTTGATCTCACGACCAATGTTTGTGTATTTAATCGTGATCTCTGTCAACCTTGGAGTGTGCATCATAGAAATCGTACAGCAGCAAAACGATTTGACGACCCTAATCTCAAGCTGCGTGTTCGTCGTGGCCTGTCTGATACAACTTCTTCTTTTCTATTGGCATAGCAATGTAGTTTCTCAAGAAAGTACAGTAGTTAGTTACGGTACCTTCGAAAGCAACTGGGTGGGGCTGGACCAGCGGACCCAGAAGGAGGTGTACTTGCTCGGCCTGACCACCAGCACCAGGCTCGTCTTTAAAGCCGGTCCTTTCAATGAGATGTCACTTACTACTTTTGTTGCGATTCTGCGACTGAGCTACAGTTTGTACACACTGTTAGACAACACTATGTAATTTGTTACCCATCTACGTAGGGTAACGAAAATGCTTATTAATTAAATAAAAAAGAGTCGTTTATTTCTTGACATTTTTCACTTTTCTGTAACTCATCCAAACACAACACAATATAATAAGTAAGTAGGTACGTTATAAAATAATCTCAAAAATTGTAAAGTAATATATCAATGTTAGATAATAGGTAACATATTAAAGCAAAGCAAAAAGTAAAAATTAGCTATATATATTTAAAATTACTACCCTTGAAATATTGTCTTTACCAGCCTTTAATTATAATGTGAGTAATGTCGTA

>OR21 [Moltype=mRNA] [Organism=Loxostege sticticalis], complete cds

AAAGGCGTATGTGATGAGTATTAAGAGGCATTCTTGAATTACGCGTCAAAGGCTTCACTCCGCTGCCGATAACGATGAAAATACTTATAGAAAACGTGAAAAAGAAGCTTGCTGTGTTGCAGCCAGTCCTTCCATACGGGGTCATAGAGTCCTGGGATGACTTGAACCCGCGACTCTACCATGCCGTTCACATTTACTGGCTCAAATTCTACGGGATGTGGTACAATAATTCCTCGCCCAAAAGTATCTTTTTCTGGCTCCAGATAATATACACGGCGATCGTGTTATGGCTCGTATGTTTTTTTGCCGGGCATCGGCGAAGTCGTTTATTTGCTGAAGCGAAGGGAAAATATCGGAGACGTCGCTGAAGGATTGTACTTATTCCTCAGCGAGATGTACACGTATTTCAAGGTGGCTGTTTTTTGGTTAAATAAGGATAAAGTTATAAATCTCCTCGGGTATCTGTCTTGCGAGGAGTTTAAGCCGGTGGAGGCGGAGCACAGGGAGATTATACGGAAAAGTATAAAGGCGGCGCGGTTCGTGATGACGTACTACTCCACCATGTGCGTCGGCGCCGTCTCAGTCGGCATCATTATGCCACTTACGGAGAATTTCGACATTTTGCCGACAAACGTCGAGTACCCGCACTTCGATGTGTACAAGTCGCCAGCCTACGAAACTTTGTATATTCATCATATTTATTACAAGCCAGCAACCTGCATAATCGACGGAGTAATGGATACGATATTAGCCGCCTTCGTGGCTTCAGCAATCGGCCAAATAGAAATATTGGCGTTTAACTTGCGTCATTTCGACTTGGTGGCGGAGCGAAGGCGCAAAAGAGCCGTGGCGGGGAATAAGCCGAGCGCCACTTGGACCAAAGAAAGGCACATTCGGGCTGTGTTGAAAGAATGTATTGTACACCACAACAGCATCATTAAATACGTGTCTATGATCGAAAGTGCATTCAGCCTGGCTTCTGCTCTGCAATTCATGCTAAGCGTTATGGTGCTATGCCTTGTAGGCATCCAGTTTCTTTCGATTGAGAACCCGTCCAGACATCCAATGCAAATATTATGGATGGCGATTTATTTAACTTGCATGTTGATCGAAGTCTTCATTCTGTGCTGGTTTGGTGATGAGCTTATTTGGAAGAGTACTGAGCTCAGGCAGGCAGCTTTCGACGGGCCGTGGCTGGAAACCAATCATAAAACCATGGTTTTCATAGTAATCTTTTTGGAGCGCTGCAAACGTCCTCTCAGAGTGACTGCCGGGAAAATATTTACTCTGTCACTGGACACTTATACAGTACTTATAAATTGGTCTTACAAAGCATTCGCCGTCGTAAGCAACATGAAAAAGTAAACAATGCTAAGAAAATCCATCCATTGCCTTAAAGGAATTCAAGAAAACAACCGTATTAAAATTTTAAATTCCTCGCTGTCTTATTCAAAGAAGCAGAGAAGTGGTAACATTTTGTCACGAGCACAAAACATGCA

>OR22 [Moltype=mRNA] [Organism=Loxostege sticticalis], complete cds

CCGGATTGTTAGTGCTTTTTCCACGTCAAAATGTTTGTGTTGAAGAAGCTCGAAAACCCAAAAAGACCTTTGCTAGGTCCCAATGTCAAAACTCTAAAGTTCTGGGGCCTACTTCTCCCTAAAAATGTGTACATGAAATACTTTTACATAATTATGCATTTGATGGTTACAGCTTTCACTGCAACGGAATATGTCGACGTTTGGTTCATAAGGAACGACTTGAATCTGCTTTTGAACAATTTGAAGATCACCATGCTTGCTACAATGAGCGTTGTCAAGGTTACCACTTTCTTATACTGGCAGAAGCATTGGAAGGACATCATTGAATACGTCACCAGAGCGGATTTGGCTCAGAGAGCAACCGATGACGTCATCAAGAATGATCTGATTAAGAAATTTACTAAATACTGTCGGAAAATAACGTACTTTTACTGGTCTCTGATGTTCACAACAGTAGTTATTGTGATCTTTCATCCGCTACTCAAGTTTTTTTTTGTCGCAATCATACAGAGACAATGTCAAAGAAGGAAATGAAGATTACTTGCAGGTTGTCAGCTCCTGGGTACCTTTCAACAAGAGTACGATTCAAGGGTACTTAGCCGCTTCTATTTGGCAGTCCTACGCGTCGATCTATGGAGGAGGCTGGATAACCTCTTTTGACAGCAATGCCATGGTCATCATGGTTTTCTTCAGAGCAGAGTTGGAAATGTTAAAGATAGATTGCGCAAATATTTTTGGGACGGAATGGAATCCTGTCTCAGATAAAGTAGCTTTTGCAAGGCTGAAAGATTGTCATAGGAGACACGTGGAGTTGGTGAAGTATTCCCGTCTTTTCGACGCTTGCTTATCTCCAATCATGCTTTTGTACATGTTCGTGTGTTCCGTAATGCTTTGCGTTACGGCATATCAAATTACATCCGAGACCAGCGCTATGCAGCGATTTTTGACGACGGAGTATCTTGTCTTTGGTGTAGCGCAACTCTTCATCTACTGCTGGCATAGTAACGACGTGTATTTTGCCAGTCTCCGACTAAGCGAGGGTCCATATGAGAGCACTTGGTGGTGCAGGCACGTCTCTCATCGGAAGAACCTCTTCATCCTGACGGCTCAGTTCAGCAGGGTCGTGGTCTTCTCTGCTGGACCATTTACTAAGCTCACTGTTGCTACTTTTATCAGCATTTTAAAGGGAGCCTACAGCTATTATACTCTTTTGAGTAAATCGCAAACAAAATGAAGACAGAATCATAGTTACGATGACAACTCACAACCTAGTTTTGAATAATAAAGTTTAATAAAACTGATTTGTTTCTAATATTTTGAC

>OR23 [Moltype=mRNA] [Organism=Loxostege sticticalis], complete cds

CGACCATCATGGGAAAATAACACTGAAAAAACCCGGACCAAGCATGTGACCCTTATTACCAAAGATTAATTATGAACAGACACGTTATTTTTCCCTGGAAAAAGTGCGTTGAATAATTAAGGACTACATTTAAACTTACCAATGAATAAACACTGAATTTTATTACAACATTTATAGTGCATTATTTAGTCTCATACAAGACATTACAATGGCTGAAAGTTTCAAAGTTAAAACAAGTCGAATGGATATTATAAGACACAAAGTGTTGGTCTGGGCTGGCATTTATAAGTTACACACAAAAAAATATTATCTGGGTGTATGCCATGATGTGTACAGAGTTTTTGTAATAGTCATGTTAATTTTGGTTAACATTCAGCATATAATTTACATTTATTTGCGTGCGATACGCGGGGAAGATGTACCGTGGGACATTATCCTTTTAGTAATAACAATGATAGATTTGATTATTAAAGTAGTAACAATAAACATTCATTCAAAACAAATTGACGAAATACACGACCTAATAAAAGCTCCTATGTTTGACCCAACATGCACGGAAGATGAAACTATCTTGAAGAAAACCGAAAAGCAGATAAATATGCTGCTAAAAATAGTGTATATAGATGTAACTGTGCTCAATATCACCTGGGACATTTATAAAATTGGTCAAAGAATGACGAACAAATCAGCTGCCATAGAATCATATTTTCCTTTTAATACCAATCCTTGGCCAGGATATTTACTTGCTTTCCTGTATGAATGGTGGATAATAATTATTTGGCTTGGATATGGGCTCTTGTCATTAGACTGCAGTATAGCGATCTACTACACCCGAGCGGCTACGCAGTTAAAGATAGTTAATTATCACTTAGAACACATGTTTGACAATGTTAAAGTTCAGAGCCAAAAACGTTTTCAATACAAAGATCTCGTGGACCGAAGTTTGAACCTTAAATTCATCCACTTTGTTCAACGCTATCAAAATATTCATCGATTGATTAATACCGTGAACATCGCCTTCAGTGAAGGTACAGCCTTCCAGTTCTTTAGTGCGACAGTTGGCATAGGCTTTTGCCTTTATAAAATGACCTACACAGAACTTTTCTCGGTCGAGTTTCAGTTAGCGTTTGGACTTGTATTGATTTATCAAATGCAAAATTTTATGTATTGTTACTTTGGAAACAAGGTTGAAAGTGAGAGCGACCGAGTCTGTACGTCAATGTACTTCAGCGACTGGCCGTCGGCGTCGCCGCGGTTCCGCCGCCAGATGCTGATAGCGATGGCGCGCTGGGCGCGCCCGATCACGCCGCGCGTGTCCATAGTGCCTGTCACGATGGCCACGTTTGAAGCGACCGTACGTCTCTCCTATACATTGTACACTGTATTGAAAAGCAGAAGTATGACTATGAATTAAAATATTGCTATGTCATCTAAGCATAATGATTTATTATTACTATTTGCATGTATGTTACACTAAGCACCAGTGGTTTTGAAGAACACAAATAAATGCATCTTGTTTTAAAAATGAAGGTCATTTTCGTAAATTGCGATCCCAAGGGCAAATACTCTTATTCCCTTTTATTGTAATAACTGTACCTTAAGTATAATGATACTTAATCTTGATGTATGCATCAGGTGACTTTTCAATATGAAGACTGTATGGTGTTCATGTGCACACAGGCACACTCTTACAGTGTTCCACTCATTCCAGTCAGTTGGATTTGTCACCTCTGTGGAGCAATACATTGATTTGAAAACCGACAAGCAAAAATCCTGC

>OR24 [Moltype=mRNA] [Organism=Loxostege sticticalis], complete cds

GGAAGACGACGATTTTGACGAAAAAACAATAACTGAAGTCAACACGGTCAAGATCTTTAACTATGAGCAAACTGATGAAGAGAAAGAAATAGTGAAAGAATCTATAAAATTCCTGAACTTTGTTGTTCGTCTACAATACTACATTTGTGGTGTAGTCATATTCGCGTTCCCACTGATGCCAGTGACTTCGATGGCTTACGACTACTACATGACGGGGACTACGGAGTACAAGTATCCGTACTTGGTTAAGTACTTCTTTGACGTTTACAATATGAAGATGTGGCCGGCTGTGTACTTTCATCATGTCGGGTCAACGGCAATCGTGGGCGCAGCGGTGTTTGGCTCTGACAGCCTGTTCTACACGGTGTGCATCTACATCCAGATGCATTTCCGAACGCTTTGCCTTCGTTGCGAGAGGATTGTTACTTCATCGGCTAAGGAGACGAGAGAGAACCTGGCGAAAGCTGTGAAGAGGCACCAGGAGTTGATTGATTTGGTGGATCAAGTGGAAATCCTGTACTCAAAGTCAACTCTGTTCAACATCGTTACCAGCTCATTCTTGATATGCTTGAGTGGCTTCATCATTACGGTGCTGGAAGACATCAGCGTGGTGGTGACCTTCGCAACGTTTCTGTTCATGAACCTGTCGCAGATATCTCTGCTGTGCTACTTTGGAGACATGCTCATGCGTTCAAGCACGGAAGTCAGCTCGGCAGTGTACAACTCGTTGTGGTATGAGACTGACGAGAGAACTAAGAAAAGCATGCTTGTGATTCTTATGAGGGCACAGAAGCCTTGCAAACTAACCGCATGCAACTTCGCAGACCTCAACTTGACAGCATTCACTACGATTTTAAGCCGGTCATGGTCGTATTTCGCCTTGTTGAAGACTATGTATAAGTAGAATTATAATGTAATCTATTTATTAGGAGAGTCTAAACAATTTTAGTAATTATACCGAGAAAACTCCACTAACAAGGTCATATCTCTTAAATCATAGATGTTGGACTCAACAAAATTATTTATGCTTTGAAAATATTCATGGAAAAAAAAAAGTAAAAGCACATACGGAGATTAGAAATAAATACTGCCCATGAATAAAACCAATTGCAGTGTAAATGATTTCATTAAACCCGATTAATTTCAG

>OR25 [Moltype=mRNA] [Organism=Loxostege sticticalis], complete cds

GTTTTCATCCGAAATCACCAAAGATACGTTCTCTGCTTCAAGCTTCACTCTAATTGTTAAAACTGAATTTTAGGCACCAGCTCTCACCACCAAAAATATGTCAGTCACAATTTAAGACAGAAATATACGTAATGATACTTTACTAAAACGAGACACTGAAAATTACATGGTAACAGTCATACACACTAGGTGAAAAGCATTTAAAACAAAATGGATTCTAACAAGCTGTCTCACTCATTTTACAAGATTACATATGTTTGGAAATTACTCGGAATGTGGTCAGGTAAATCATCAAGTAAAAACTTGAGAATATATTCTTGGCTCTTCGTTACTTTGTATTACATAATGTACAACTTCTTTTACACATTGAGCTTGGTATGCGCACCACGAGCAGTAGATACAATAGGCGTAGGAGTATATTACTTCACTACATTGTGTGGGTTAGCTGAAATTGTCATGATTCTGAGAAATCGGCAGAAAATCATCTCCGTCTTTGAAACAATGGATTGCAAGGAGTTCCAAGGAAATAATCGCCAAACTAATGAATACCTTCGACAATTTAAGATAACCTTTTCAAGATACTTCAAAGTATATGCAGCGTACTGCTTTATCAGCTCACATACATTTCTTATGTTTCTGCCTTTATTCAATTACTTTTTTCAACACAAAGAACTGGAAATGCCGATCTGGGAATATTATTTTTTGACCAATGCAACACGGAACAAGTATTTTTTCTATCTCTACACATACCAAAGTATGGGCATGATAGCAACAATTTTTAATCATATAGTTTACAATGTATTTTTATTCGGAATATTATCCGTGGCTGTTAGTCAATCTAAAGTACTAAATTGGAATATTGCAAATATAAGGCTCGCTGATGAGGATATTTTAAAGAGCACGGAAGAAAAGGAACGGTTGTATTTAAATAAATTATATAACTGTTTGAAACATTATGAAATCATCTTGAAGTATTGTGAAGATGTACAAGATTTAACAAGCTTTTTAATATCTTTGAATTATGGATTGTCAGTTTTCACTTTATGTTTTAGCATGTATATGTTCCTTCTGCCTACGAATAGTAACACTTTGGTTTATATGGGATTTTACCTAAGCGCGATATTGATCAAAAACTTTGTTCCTTCATATTTAGGCAGTGAACTAACAAATGAGAGTAACAATCTACGGTTCGCAGTATACAGTTGCGATTGGGTTCCACGATCTAAGAATTTTAAAACTTGTCTCATGATTTTCGTGGAACGAGCTCGCAGGCCTCTGCTTATTAAGGGCCTCAAAGTAGTGCCACTGTCTTTGGCAACATTCACTTCGATTGTGAAAACAGCGTACTCCTTTTTCACTCTTCTGAGAGGGGCTCAAGATCAATTAATATAAGTTGTTTTTTTGCGAATATTGACTTGTTTTATATTCAAAATTATCACACTTTTATCAACTAGTTTACGACTTAATTATATTTTTGTGGCTTTAGTTTAAACGCACTTATAAATACAAAATTGTATGTTCATGCCTATATAAAGCATGCAATGTAAGTTCAAAATAAGAAATAGAATAAGTCACTTAATAATAAAATGTCAGAGA

>OR26 [Moltype=mRNA] [Organism=Loxostege sticticalis], complete cds

TTGTTCTGAAGTAGTTAACGGTCTATAAATTCCCAGTTTTCATTCAAGAGCCATGGGTATCTTTAAAGGTTTTAAAAGAATTTTTTTCAAAGAAAATTTCGAATTTTCGAGTCCCGATGTTGACCTCGACAACTTTCATCCCCAGCTACGACGTTTGATCGTACCTTTAGGAATATTTTTCAATAACCAAGATTCGTTTTTAAGATTCCTGAACCCAGTCTTAAACAGTTTAATGATTTTCATAGCAGTAGTTATGGAGATGATATGCGTCATCCATGGCATACAGACTTTGGACTACTCCTTCTTCACAGAGTGTTTCTGCTACCTGGTCATGCTACTGTATGTGCCAGTGCTATACTTCAGCGTGCTAGGCAACAAAGACAGCATGCTGGAAATACTGCACCAGATGGAGGGAGACTTCAAGTTCATTTGTAACTTGGGTGATAAACACAGAGATCATTTTCTGAAGAGGCAACTGTTGATCTGGCAGTTCTTCTTGATCTGGATGGCCACCATATCCTCTGTCGCGTTTCTGATATTTCTGCGGACGTTGATCCCCCTCACCTATCAGAGCCTGATAGCCACCCATGACGAGCACACGATCCGACCTTCTCTATTCCCAATGTGGCTGCCAAAGGATGATCCCTACAGGACACCTAACTACGAAATATTCATGTTTTTTCAAATGTATTTCGTCTGCGTATACGTTCAATCTTTTGGTGTGAATGTCTACATTCAGTTCCACATGCTAATACACAATTATACAATATTGGAGTTGGTGATAATCGATTTCGAGATGATATTTGAAGATCTCGATGAGGATGTGGTATACCTATCGCGCTACCACCCGCGTCGAGTGCTAATCCAAAGAATATTGAACAAAAGAATACAGAGGATAGTTGCGTGGCATGATTCCGTCTTTACGTCGTTCGACAATCTTTCTTCTATTCAAGGACCAGTAATCTGTTATCAAGTGCTGTTCACTCCTATAGTTTATTGTTTAATGATGTTTCAAATAGCAGATAAACTGGAGGGTGGACAGCTTGACATATATTTCGCAGGGTTGCTGTCCGTGTTTACGTTCCAGCTTTGGATGCCTTGCTATATAGGCTCATTGTTAAGAAACAAAGGCTTCGACGTCGGCGAGGCCTGCTATAACAGCAGCTGGAACACAACGCCCCTGTCGCGCATGATTCGGAACGACATCGTCATTGTCATGAGCCGCGCGCAGCAGCCACTATCCATGCAGTTCTTCTGTCTGCCCGACCTGTCGCTTGAAACTTTTTCTTCGATTATGAGCTCTGCATACTCTTACTTCAACATGCTAAGGCAATATAATAGATAATTACATTAATTTTTCACTTTCATTAGGCAACTAAAATTTTTTTTTACTATTTATCAATATTCTGTTGATTGATATACACAAATAGTCATCTCAAAAAATTATAATTATACACATATAATTTTAATATTTAATAAATAAAAAGCACTTATCAAGAAAAAAAATGTCTTTACTAGTAATCTAAATAAAAACTTATTATTTATTTACATAAGTATTTACACAATAAATATGTTGATTGATGATCAATAAATTGTTCTAATGCTGGGAACCTTTATGTTCGGGCAACAAAAAATATATTGTAACAGGACCTTTAGTGGTGTGAACAATTTCTGTATGCCTGATCAACTGTGTTGCGGCCAACCCATACAGCAAGCAAGGAATACCTAAATTAACGACTTTAATAGCTCTCATAAACTTGTCAGATGCCTCAAGGTCCTCGGCTGATTTTTTTGAACAATGATACTTGATGTAGGGGAAGCACCCTGTTCTTAGTATCTGGTAATTAGAACTTCCAACTTTCCAATTGAAATTCGACATGCCAAACTGGTCATCTTTTACACTTCTGTACTTAACAAAATAAGAAGTCCAAGGTGGTTCGTTGCACTGCATCAAATATGCAGTTAGCACCTCTGAAGCTGTAGGTCTCGAAGAAAACTGCGCTGCGTATACTGTAAATACACTGTTTTTTAACAGTATTTTCAAGTTTTTTATCATTTTTTAAATGAATTTGTTACGTAAATATTTTGTTTTGATTTGCTCGCCAAGTTTTTTTAACATAG

>OR27 [Moltype=mRNA] [Organism=Loxostege sticticalis], complete cds

TTTCACTCTAAGAGCACTGAGTAAGCGGAAAAGTTGGTTGAAATGGCCACTTTTAATTCTGAGGATCTGTTCTTGAACAGAGCCAAGTTTGTAATGAAATTTTTAGGAGTATGGATACCGCCTGTCGACGAATCCTTACCGCGAAAGTTTCTCAAGCTCTTCATGCTCATGTTGCAATACTTATTTCTGATTTTCCAAACGATTCACATAGTGCAGATTTGGGGGGACTTGGCTGCAGTCTCTCAGCCGTCCTATCTGCTGTTCACGCAAGCTTGCCTGTGTATGAAAATAACAGTATTTCATGTGAATGTGGACAAACTCAGGGAGCTCTTGAAGCAAATGGCTTCCGATACTTTTATGCCGCAATCTATTGTTCATGAAAAGATCCTCAAGGCCCAAGCTGCAAGGATCAAGAAGTATCTTCTGGCGTTCATGATCGGCTCCCAAGGAGTGTGCAGTATGTGGTACTTGCACCCGCTGTTCGAAGGGACGGGCGTCCGCAAGTTTCCCTTTGATATGTGGATGCCAGTGTCTCCTGAGGATTCCCCACAATACGAGATCGGGTATGCGTTTCAGCTGCTGACCATTTGCATGAGCGCTTACATGTACTTTGGAGTGGACAGCGTCGCTCTATCCCTGGTCATCTTCGCATGTGCTCAGATAGAGATTATTAAGGACAAACTTTTGAGCATTGCTCCAGTTCAATACGGATTGAAGGAAAAAGAACGCAAAATTATGAATGAGAAAAATCACAAGATTCTAGTGGAGTGTATTGTTCAACATCAAGCTGTTGTGACGTTTACCCAACTAGTTGAAGACACGTACCACTTGTATTTGCTGTTCCAACTAACTGGCGACGTGGGAGTGACCTGCATGTGTGCTTTGAGGATATTGGTCGAAGAAGTGCGGAGTGTGCCGTTCGCATCAATTTTTTTATATGTTATCGTGATGCTGATCCAACTCTTTATATGCTGCTGGTCGGGACACGAGTTGACTGCAACTAGCGAAGACCTCCACACGGTCCTGCATAAGTGCTCGTGGTATGAACAAGACCTGAAGTTCAAGCGTGACCTGCGCTTCGCCATGATGCGCATGAACCGCCCGCTGGTCCTTCGAGCCGGACACTACATCAGCATGTCGAGGCAGACATTTGTTGCGGTCCTGCGAATGTCGTACTCTTACTTCGCAGTGCTGAATCAAGCCAACAAGAAAGACCAATAGCAATGTTGATAAGTATTATAAATACATTCATGAACTTTTAGTTAGAGCACTATGAGTTGAAATCTCTAGTTAAATGTAGGTCTAGGATCTATGTGTAGAGTCAATGTATAGCATTAAATACTGCAAATGGTTATTAATTTGCAGTAATCATTTATTTATTATATTTATTGACACAGTATATTTTTGAATTAGGTACTTTAACTGTTAATAATTAAAATGACATATAGTACCTAAATTAAACACACTCAAATAAACCGCGACGAGAAACCGCGACGAGA

>OR28 [Moltype=mRNA] [Organism=Loxostege sticticalis], complete cds

CTTCGATGGTAGCCAGCGGCCGGTGTCGTTAAAGTCTATGCAATTTATTGAATTTAAAAGTAATCTTAACATTGTCCATTTAATGAAGCAGGGTTTTGATAGGATCGATTAATCTAAATTAAACATAAAATCCTACTTGCAATACAATGTGGGAGAACCTTCGCAAGTTCGGGCTGGGGCATTGCGACCTGCCCACCATGGTGTGGAACGTGGCCTTCATGCTGCGGGGGTTCACCCTCAACATCGACAGCAGGTTTACTGGACGTATCCCTAAAATATTCTACATCACCACCATCATCATCGCGTTCTGCTACCTCTATTCGTACTTCTTCTCGATGCTCTGGTTCGTATTCTGGCGCTGCATCGAGACCGGTGACGTGACCGCCGCCATGATAGTCTTTCCGCTGGGCATCACCAGCGAGATCGGCATCGCCAAGTTTATCTACACTTGTGTGTACAGGAAGAAAGTTCGCCAGCTCCTCCAACAGTACCTGGAATATGACTCTCAGATCCCCCAGGGCTCTCGTTTATCCAGGCACCTTCTACAAGCCCTTCGAAACGTCAAACGACGCGCACTCATCTACTGGATCTTTATCGTCAGCAATGGCACCCTCTACATCCTCCAGCCACTGGTTATGCCAGGGAGGGTACCGATGGAAGAAGTTTTTGTTCTATATGGTTTGGAGCCAGAACTGGAGACTCCTAATTATGAAATAACATACGTTCTCTGCACTTTTGGGTCCGTCTGTACTTGCTACCTAACCTCCAACGTGGCTGCGTTCCTTATCATCGTCAGCGGCTACGTGGAATCCCAACTATTAGCACTCAGCGAGGAAATCCTGAATGTTTGGGATGACGCCGAACTCGAGTACAAAGTCATAGACAACGCCGATGAAGAAGAATTTGAAAACAAAGAGAAATATGATGCTATAAATGAGTCAGTTAAAACCCGACTTAAAGATATCGTCAAAGGACATACAACTAATATAAACTTATTACTGCAAGTCGAAGACATCTACAGGGGTACATTCAAATATGCTCGTGCTCTTCACTGGACCCGTGTAGAAATCGAAACCGCTGGCTTTGAAGTCGGGAAAGCGAACTATGAGAACAGCTGGCTTCTTTTGTGGAAAATGCAGTCGAAGAATGTCGGAAAAGAAAATGTTAATAAAATTTTATATGTATTTATATCTTTTTCTAACGCACTAGACTACTCTGCCCTGTTGTCACCGATATCATATTGAATAGACTATTTATTAAATTGTAAATATTTATTCACAATTATTCTCTTGACTTGAGTATACCAATTACACAAAACAATTTTGTTTACGAAACAGCTGCTTTATATAGTCTATAGTTGTCAAACTAATAACATTTAAATTTTGGATAAATTACACAATGCTAAACTGATATCGAGTCTTGTTTTATTCCATTCCTTTTAAATTTCTGTATACTTTCAGTCTCTACTTAAATTTCCAAATAGACTCTCCGAAGATTATACACAAGCAGTTTATCCTGCTTAGCTCAGGCGATAGGAGCCTGTGCAATGACACGTGAAATTGACCACTGCCCCCACCGCAGCACCAGCTAACACACATTAGTTTGGATTTAAGAGCTGGGAGCTGCGGATTAAAGAGTTGCAAGTTTAAATCGAATGTCGTGTTTTTTACTCAAATTCTACAACAACAGATATTTTGCGGTCTAAGAATAATGCAATCAATAATGCACATTTAGTTAATTGCTAATGGGGAAGAATTAGAGTGCGCTGTGTGTTTGTTAAATTGATATTTTTTTTTAAATTTTTTGCTATCGTGGATTTTATTGTTGACCTGTAGTTTTAGAAGTAGTCTCAAAATCAGTCTCTTTCAAATTTTTGCTTTATCTCCTAGAAAAGTCACTTCGTT

>OR29 [Moltype=mRNA] [Organism=Loxostege sticticalis], complete cds

CTTCACTTGTGTCAATAAAATTAGTCTGCGCCATTCACCTTTGGAGAGCCTACTGATAATAACACAATGACAACAGTACGAGTACCAAACGAAATGTCCAGATTCTTTATAAACTTAAACAAAATCACCTATCACCTTGGCTTGCCTAACTTGTGGATAGACGATGTAGATGCACCTCTTCCCCTCAAAAAAAATCCACAAGGGTTTGAGATTTGCGATGAACGTCATCATACTGTTGTTTATGATGTCCGAATGGGCAGCGTTTTTGACTCAGAGCAACTTGACCGAGAAGCAAGCAACTGACCGACTCATGTTTGGATTTTCGCACCCCACTTTGTTTTCGTACGTGCTAGCCGTGGAGTATCACCAAGAACGAATAACAAACATGTTGCACAAACTGGCCATGGTTCTTAAGGAGGAGTATAACGACAAAGACATCGAGCGCAAGATGGTCAAGAAAGCTATGTCCAACGCGGCAGGATTCGCTTGCTTATTCATGATGGCGCTCATCTTTTACGGATACGATGGAATCATGCAAGTAATAAGAGGAGATGGGACCTTTACAACAGTTATAACATTCTGGCCGGACGTGACCGAGGTATCTTACGCAGCCTCCATCACCAGGGTAGCTACTTATTTCATCTGGTGTGTGTTCATGACACGGGTGTGCGCTGTGTACTGCCTCGTGATCCCGACGACTGTCTGTCTGAGCCACCAGTTCAAGAACCTGCAGAGCTACTTCTATTCCCTTGAAGATATCTTCGACGAAGACTTGGAACAGACGGAGCTAGAGAAAAAGTATGAAGAAGCATTCAAAGTTGGCATCAAAATGCACTCTAAGACTTTGGAGTGCACAAAAGACTATCAGACGGCTTACAACGTAATCGTCAGCAATCAGGTCCTGACCACTGTTGGAGTTCTCGTCCTGCTCATGTCACAAATGGTGGACTCTGAGCGCACGTTAGAGAATGTTCTGTCGATAGTAACAACCGGTTCGGCGATGTTGATCAGCACAGGATTTTTCATGTGGAACGCAGGGGATGTCACGGTAGAGGCAGCAGAGCTGGCGACGGCGGTGTACTGTTCGGGCTGGGCGCACTGCCAGCACTCTGCGCCGCGCGTGCGTCGGCTGCTCGTCATCACTATGATGCAGGCACAGAAACCTGTGGTCATATGGGCGCTGGGCATAGTGGAACTATCGTACCAGTCGTATGTATCGATCGTGAAATCATCTTATTCTGTTTTCTCCGTGCTCTACTAAGCCTGGTTCGGACTTCGGAGTATAAAAGTAGACGATGGCTTTATCACTAGTACCTGAAGGAGTAGAATACTATAAAGTTTCTAGTAGTTTTGAATAAAAGTAGGTAATAAAGACAGTGTATAAACGTAGTTTTATTCATTAAACATTGCTCCAGCTCAATTACAAACATCACATTTTCTTTATCATCTATAAATCTTTAACAATTTTACGTAATTTGTCAGAAGTAATAGATCAATTATCAAAAAGACACCTCCCACTGTGTACAGTTTGGAAGTCTATACAAAATTATTTATTAAAATCCATGCATCTATAGATAGTAAAGATAGATTTCGTAAAATAAAACATATTATTTAAAATTTCGATTTTTT

>OR30 [Moltype=mRNA] [Organism=Loxostege sticticalis], complete cds

AGCAAGGCGAGGTGTCGTTAAGTTGACTCGTAGTTTACGTTGAAATATAACTGTAATCACTCAGATTTTACTTTTACAAAATCATCTACAATTAATTTGTTATTTTTCCATTTGTAAATTTATTTTGATTATTATTAACAATGAAGAATTACGAGATTTTAAAAAAATACTGTAAGAAAATGTTCCTAGTTGGCTCAGGTAACTTTTGGTACGAGTCAGGAATCGTCGGGGATGACAGCAGCTGGTATTACAAAATATACAGCCGGTCACTTTTTTCTATTTACGGTTTCATGACCATTCTGGAGATAATGGCCGCCATATTTGGCGATTTTCCTGGTGACGAGAAGCGAGACTCGGTAACTTTCGCGGTGAGCCACACGATTGTCATGTTGAAGATATTCTCAGTGGTCTCAAATAAGGGACTGGTGAAGACGATGAATCAGAACATGGTTAAAATTTGTGAAGCCTACGAAGAGCCGACGTTAATGGCAGCCAAGTACAGGATTGTTAGGATCAATGTTCTGGCGTATTTTAGTGTAGTGTACGGCTCTGCTGCATGTTTTGTCTGCGAAGGCATTCGAAAGTTAAACGCTGGATCCCATTTTGTGACTGTGGTTACGTACTATCCAAGTTTTGAGGATGACTCTATTTTTGGTATTACATTCAGAATATTTACCACGGTCATTTTGTTTTTAATGATGATGACCATGATTGTGTCCGTCGATTCCTTCACTATGGCTTATTTAATCATGTATAAATATAAGTTTATAACACTGAGACACTATTTCGAAGGGTTAACTGAAGAGTTTCACAAAATGAATAGTGTCAATCCCAGATTAGCTGCTGATAAACTAACAAATGGGTTAGTGGAAGGAATAATCATGCATAAAGAAATATTGAGGATGGCAAAAGACATAGATCAAGCATTCGGGACAGTAATAGCTCTGCAACTGCTGCAGAGCTCGGGCTCCGCCGTGTCCCTCCTACTTCAAATAGCTCTCTCGGACCAGTTGACGTTCGTCGCGAGTATGAAAATAATCTTCTTCGTCGTCGCACTATTCTTTTTATTGGGCTTATTTTTGTGCAACGCTGGAGAAATCACGTATCAGGCATCTCTCCTGTCAGATGCCATTTTTTTTCTGCGGTTGGCACGTCAGCAAATGGCAGCCGCCTCCGCAGCGCAGTCTCAGGCGACTGGTGCTGCTGGCGGTTACGCAGTCGCAGCGGCCGCTCGTCATGAAGGCCTTCAAGATGATTGAGCTGTCTTACGGCACTTTCTTGCAGGTACTGAGAGGAACGTATTCTGTGTTCGCTTTATTTTATGCACAAAATAAGTGAAATAGTAAAATAATCTAATAAAAACTGTTATCTTTTTCACGGTTGCAGTTATAGCTCTCAATAAAGTTAGTAAGTAGTTCTAA

>OR31 [Moltype=mRNA] [Organism=Loxostege sticticalis], partial cds

CACTGGCCACGAGTTGTCACTAGTGTATTTATTAATATTTAAAATTCTAAATCAGATTTCTGAAAATATTTTTTAATTTTAGTGTTAATTTCGTTCTTTTCTTTTGTTGTTTGTGATCTTGGTAAACGTGTCGATTAGGTAAAATGTCTTTGGCGGGCAGCAGCGTGGCGCCGCACCTGGCCCTGCTGCGGCGGGTGGGGTTTTGCCACCTGCAGGGCGCGGCTGGCGGCGGCAGCGCCCGCTCCGGCCCGAAGCGGCTACACAGCTACTACTGCTTGTTCGCGTTCGGCGTCACCTCGGGCTATGTGCTGCAGCAAGCCATCTATGCCTTTCAGGAGCGGAGTGACCTTGACAAGCTGTCGCGCGTGCTGTTCGTGATGCTGTGCCACTGCACGTGCGTGGCCAAGCAGGCCGTGTTTCACGCCGACGCGCGCCGCATCGACCGCATCATCACCGGCCTCAACCAAACGCTGTTCAACCAGCCGATTGAGTCACACCGCTCGCAACTGCGCGGCACGGCGCTCAGCGCGGCGCGGCTGCTGCGCGTGTACTACAGCACCGCCGTCGCCACGTGCGTGCTGTGGATCATCTTCCCCGTCGTCTACAAGCTGCGCGGGCACCGCATCGAGTTCCCCTTCTGGACCTTCGTCGATTATAATCAGCCGGTCATGTTCGTAGTAGTGCTGTTCCATTCGTTCTACGCGACGAACCTCGTGGCCGTCGGCAACACGACAATGGACGCTTTCATCGCGACCGTGCTGTTTCAGTGCAAAACGCAGCTACGCATATTACGGATGAATTTCGAAACCTTGCCGGA

>OR32 [Moltype=mRNA] [Organism=Loxostege sticticalis], complete cds

TGCTGAATTACATGCTGTATAATGGGATGGGAACTTGTTCATTGTTAAGACATGTGTTACTTTTACAACCAAAGAGGAAAAGTGTCTGGTTATTACTTCATTCAAATATCGCAGCCAAGATGGCGGGTCCTACGACTTACTCTACGTTCAGTGCAGTTACTCCACATTTTAATGCGCTTGCTAGAGTAGGGTACTTCAAAATGGTTATGAAAAACCCTTCTCCTACCCAGTTGATGTTACACAACTGTTACCGATGGCTTATTTGGTTTTCGATTCTGTCGTACAACATTCAGCAGTTGATAAGAGTAATTCAGACCCGGCACAGCACAGACGAGATGGTGGATACCCTGTTCATCTTGCTCACAACTTTCAACACACTGGGCAAGCATGTCGCCTTCAACGCAAGGGTATGGCGCATTGACCGGATCATCAAAGTGATTAATGGGTCCATATTCGCCGCCAAGAATCCCAAGCACGTAGACATTATGAAGCTAAACGAAAAGGCCATGGCACGCCTGCTGTACTTCTTCCAAGGTATGGTGCTCACGGGGTGCGTCATGCATGCCACTTATCCCATGATCAACAGAGCTCTGGGCCAGGATGAAATCTCGTCTTGCTTCTCATCGGAATCAAGCGGGTCGCTCTCCACTCAAATAGCTACATGGTACTTATCAATTTCGCTCACAATACAAGCCTATGGTAACAGCACTATGGATTGTACCATATCAGGCTTTTATGCTATGGCCAAAGTTCAACTTCAAGTTCTAAGGTACAACCTAGAACACCTCGTGGATTCGGAAGACGAGCAAGAAGATATTGATACAAATGATGTCAATATAGGAAAGCTTCGGTATAAAGATAATACAGTTATACAGAGTCGTTTAGTTCACTGCGTGAAACATCAATTGCAGATAAAGTGGTTTGTCAAGGAAGTAGAGTCAATATTTTGCGAGGCAATGACCGTACAATTTTTAATTATGGCATTTGTTATTTGCATGACCGTTTATAAAATTGTTGGACTCACCGTATCTTCGGCAGAATTCTGGATGGTATTTGTGTACTTAAACTGCATGCTCGCGCAGCTGTTTATTTATTGTTATTTTGGAACCCAAGTAAAATATGAGAGCGAGTTTGTAGCCCAGTCAGCCTACTGCGGTGCCTGGACGCGCCTCTCCCCGGCTTTTCGAAGGCAGCTGGGGATCTTAATGCAGTGCGCTCGGCCTATCGTGCCCTGCGCTGCCAAGATCGTGCCCGTGTCGCTGGAGACTTACATTGCGGTGCTAAGAGCTTCATACACGCTGTTTACAATACTCGACAAGCAATAGATACGTTTTTGGAGCAATAAAATGTAGCTGCGAATATGATGATAGATCTATATTTGAGTTTGTACGTTTTATAATAGGAACACCACGCAATCAGTGTAGGAAGTACAATTTCTGAATCCTTTGTTTTACAAAGCTGCAGGTGGTTTTACAAAAATAAAAAGTTTTTGTAAAACGACTGAACGACGTTTAGTGCTTTTACATAATTACTGCATCTAAGCCGCAGAATTGCCTTAACCTAACCTAAACCAACCAAAACAATCAACACATCTAAAACTTCTTTTATGATATTAGCTGCTAAAAATAAGGTAATACCTAATTTTGACCCACTGAAAATAAATAATGAACCTCTTCAAAGATCACAACAAGAAAAATATTTGGGACTGATAATCGATGAAAAGCTGACTTGGAAGCCACACATTGACCATGTCAAATCTAAATTGACATCTGTACTGGGTGCGCTTCGAAAGATAGCACATTGCATACCAAAAAATATTAAGCCGATTCTATATAACTCCTTAGTAAAATCACATCTTGAATACTTAATTGAAATCTGGGGTTCAGCAGCACCAACGAACCTAGAACCATTACAAAGATGTCAAAATAAATTAATAAAGACTTTGTACCACTACCCATTTCTGACACCAACTATTAAATTATATGAAAAAACTAAAATAATGAACCTACGTCAACTATATAAATACAACACATGCATGTTAATTAAGAAAATAATAACAAACAATATCAAATCTAGTATTGAATTAAATGTTAGAGTCAATAAACACAATTTAAGAAATAAAAACAAACTTAAAGTAATTACGCCTAGAACTACGTATGGGAAAAAGACAATTCTGT

>OR33 [Moltype=mRNA] [Organism=Loxostege sticticalis], complete cds

CAACCGCATTGGTAAGCGCATCGAAGCAAGAAGCAATAATCGAAGCAGTAGAAATGAAGTGGTTGGTCAAAAATACTTTCACAGCTTTGAGAATATCTTTGACATGTTTGACGATCACTGGCTTTTATACGAGAAGAAGAGAAGTTAATTTTGTTTTGAGCTATTGCTTTCCTTTTCTCACTTTTATGTTTATGACTGGCATATCCATAATGGCACAATTCGTAGATTTAATTATTATCTGGGGGGACGTGGCCCTAATGACCGGCACCTCTTTCCTGCTGTTGACAAACGTGGTCCTTGGCCTGAAGGTGTTCAACGTTGTGTGGAAGAGGGAGAAAATAAGGGCCGTCATCGAGGAGTCTGATGGACAGCTGCAGGCTGTGGATACAAATTGGGGAAAAGACATTTTGAAAAGCTGCGAACGCCAATCAACAATATTTTTCTACATCTACATGTTTTTCCCCTACCTGACCATTATGGGATGGGCCACAGGTCATGAAAAAGGGGAATTACCTACGCGAGCTTGGTATCCATATGATACTACCACATCACCTGGCTACGAAATCACGTCCTTGCATCAGGTGGTAGCAGTATGCATTGGTGCCGCTGTAAACGTCAGCGTTGACACCGTGGTCACCGCCCTCCTGGCCCAGTGCTGCTGCCGGCTGAAGCTCCTGTCCGCGTGTCTGAAGATGCTGGGAGACGGGATGCTAACTAATAACCAAGGCATGTTCAAACAAGACCAAGAAGTGGCCATCAAGGCCAATATCAGAAGTTGCATTCAACAGCACCAAGCAGTGCTGGAGGCAGCTGACCTCCTTCAGGAACACTTCTCAAGCCCTATCCTGGCGCAGTTCACGGTGTCCATGGTCATCATATGCGTCACTGCGTATCAGTTGGCTTTTGAGAGCAGTAACACAATTGCCCTGCTCGCGATGGCTTGCTACTTCACGTGTATGACGCTGCAAGTATTTCTCTATTGTTACCAAGGCCACGAGCTCTCAGTCGAGAGCGGTAACGTAGGGGCAGCTGTATACGAGTCTCCCTGGTACAAGATGTCTTTACCGCTTAGACGGGACCTGCTGATGCTGATGATGCGTTCCCAGAGGCTGGCCAAGCTGACAGCTGGGGGGTTCACCACACTATCTTTAAACACGTTTATGGCTATAATCAAGACCTCGTACACGCTGTTTACAGTACTTCAGCAGACAGAAGATTGAATTAGAACATTTGAACAAAAGATATGATGTTAGATAAATAATGTGATTGATTTGGAGTAGCAATACATTTTAATGTTTAGTGCCCAATAATTGTTACATATTTGAACTTATATTTTATAATTATAATTTATTTCCTTG

>OR34 [Moltype=mRNA] [Organism=Loxostege sticticalis], complete cds

ATTCTTTTCCGCTTTGCTTTGGAGTCAAATAAGTTCTATTGGGTTACACAAGTGACTATAAAACTATCATGATGGTCAAGAAAATATTTAGTTTTGTCCGTCGCTCTCCTCTCCAAGAAACGCGTGATCTGAAAGTAAATGATTTGGTAGCCATGATAACAAAAAGGATTCAGAACGCTGGACTGAATTATAGAGACGAAAACCTCAAAGTACATTGGCTGGCTATCGCTTCTATCATTTGTTTTATAATCACATACGGGTTGCAAGTGGTAGCGTTGGTCAACGCGAAAGATGACATAGACAGGCTATTCGAATGCCTCAGCGTCATGTCCTTCTGCGGCATGGGAATCCTGAAACTTCTGTCACTGTACCGCAACCACAAGCACTGGAAAATGCTTTTGAACAAAATAACGGAACTGGAAAAAGAACAGGTCATCAATGAGGGAACTTCCAACGAAGAGTACGAGAGCGACAACGAAGATGACACAACATATTTCCCGGACTATATCGCCACGTATACCAAACAGTTTCAAACTCTCTCAGACATTCTAAGCAGAATCTATGGCTCTACTGCTATTATTTACATTCTTTCCCCATATGCAGAGTTTGCCCTATTAAAATTTACAGGCAGTGATGTCTCAAGCTATCCACATATTCTACCGGGATGGACGCCTTTTGATTCAAGTTTTTTCGGTTATTTAGCAACTATCGCAATAGAGCTAGTATCCGCCATTTACTGTGTCTGTGTGCACGTTGCATTTGATTTGACATCGATCGGGATCATGATCTTCATTTGTGGACAGTTCTCTCTGATAAGTGATTACAGCAAAAATATAGGAGGGAACGGAGCGATGTGTTTCTTGTCGAAGAGAAGAGACGATAGAGCACATGGAAGAATAATCAGATGCCACAAAATTCATGTTCAACTCATAAATACGTGCGACGAGTTGAGTAAACTATTACAGAACATACTGGGAGTGTACTTTTCTGTTGCGACCCTAACGTTATGTTCGGTGGCTGTAAGGTTGAATTCGGAATTGAGCAGCATGGAGTTGGTGTCTTTACTGCAGTACATGTGCGCGACTCTGACGCAGCTCTACCTATTCTGCCACTTCGGAGACAACGTTCTGCACCAGAGTGCAGTTGGCATGGGCCAGGGTCCGTTTGGGGCCGCTTATTGGTGCCTAAGTCCACGTATACGCAAGGAACTGGCCATACTGGGTATGGGTATGATGATCCCAAGGAGTTTCAAGGCAGGGCCTTTCATTTCGGTGGATTTGCCCTCGTTTATACAGGTTGTGCGAACGGCATATAGTTACTTCGCTGTAATTAGAAAATAAACTGATAAGACGGGAAAAGATCCAACTAGCGCCACGTCAGACTTCAACACACCGACTCACACATTGAGCACGCATATGAGTAGTGACAATTAAAGTACTTTAATCAGTTTTAAAGACACGATTGATATTTGAATAATACGCTAGTTATATTAACTGAAAATACTTTATTCGATTAGTTTTTGAGCAAACATTTTAAGATTTATTCAAATAGATGTAGTTTCTATTATTATGGGAAAAATAACATTTGTTGAATATAATAATAACCTATCACATAAATTAAAAACATAGCAAACAAATGATTAGTATTTACATTTAAAGTTCATATAAAATATGTTACATATTTACAAATGTTGTTTTTTTTGGTCAATAAGTCACATTTTAAAAGGCAGAATCAGTCTGGTTTTGTTAAAAGAATAATAAAAGTAATCACAGCTAAGCCACTTTCTGGAAAGAATGTTTTTTTTTCTAATTAAACCCTTTGTAGATTTTTTTTATCAACTGGACCTAGTGTATTTTATCTGCACTGCAAGCTAGTCAAGAAGGAAGAAGAGGCCTTCGTTCAAAGTAGAAAAAGATTAATGAGCACTGCATAAAATTGCTTCCCTAAGATTTGAAGAAAAATAATTCGTATGTTTTTGTAAACATCCCACTTCTCAGTTCTAACACCAGGGGGAACCACCATGTTCCAAAACAAGATTATAACCATGGTACAATAATATCATAGACCATGATTATAACAATCTTATCTGACATTTGCTTGCCAAAATTCAGTCTAAACGACAGCGTCAAACACAAACAAGCTTGTCATAATCTTGTTTTGAGACATCGCGGTTCCCTTCTGAAGTGGGAACAATACAACAGATACTAAGTACCGCAGTTATTCGGCGTGCGCTTTCAGCCAGGCGGCGACTGCGCTGTGGTGTGTGGCCGCCCACTTGATGTTCCCGCGCACGGTCTCGAGAGCGCGCCGGCGCGCCGCCTCGCCTGCGCCCGCTTCGGGGTACTGCTCGAAGAACGCTTCCATCTCTTTTAGCTTTAGTGGTGTGTTGAAGGAGCTAGTAATACTTGGTATGAGGTTGCCCAGGTAGCGACTGTTGAGCGTGAACCGCTCAACCAGTTGCGGCCAGCGCTCGCGAACCTCGTCCCACACGAGCCCCTCGCCCGACGGGTTCGAGCTTATGGACTGGAGTACGTTCAGATAGTCCTGACTGCGGATGTTCTTCTCTTCCCTTGCTAGGATTAGGTATTTTTTAAGCAGTTCAGGGTCACGTGGCCCAGATAGAGCGCTCCTCAGTTTGGTTTGCTCTTGCGCATCCTCTTCCTTCAGGTAAATCTGCCACAGCTTGTCCCACTCAGCCTGCGTGGACGATTTCATACCATAATAATAGACGAAGTCCCTAAGATCAGGCTCTATGGGTGCGAAGTTGTCAGTGCCGTGGTTGTTCAGCCAATCCAAAAATAGGCCTCTGACTTTGCTTTCCGCTTCCGGTAAACGACTTCTGGTTGCCAGAGACAAGATTCTTGTTCTGAGTAATCCTTCAATGACACCGATGTTGGATCTCTCCCAGCTCTGCTTATCGTAAAAAGGTTTGACCAGCTTTTGAATATATTTCTGCAGATAATCATGCTCCAGCGTGCCAAGGAGTTTGTCAGACAGTCTCGAGAAGACTGAAGCGGCCGTCTCCCAAGGCACATAGTCTTCCTCCACGGACAAGTATGTGGTGAGATCTAGCGCCAGCCCGTAAGGCACTGCCTTGGTTTCGGCTAGCGCGAACACATCGTCGAGAAGGTTGGAACGGTCTGATATTGTGAGCTGTTTAGATTTCATCTTCAACTGTTCGATCAATTTCTGCCACATGCTAGTGGAGTAATTGACTCTGTAGTATCCCACTTGGTTATTGTTTATTTTAATCCAACTTTCGTCATCTTCTAGGGTGAGTTCCACTTTATCTACGGTGTCTGGAAACCATAGAATATCCTGTTTTGGTCCCTTACTTGTCGTATAAGTGATTGGAATGAACCAACGGTAGTTGAACTCTGAATCATTTGGATAAACAGCTTCTGGGTCTAATAAGAAGCGGGATTGGGTCAGAACAAATTTGTTCGGGGTGTCACCTCTGGTCACAGTCACCAGTGGATAACCCATTTGCTTGGTCCATGTATCCATTACGTACTTAATATTAATATCAGGGTGCTCGCTTTTGCTGTAGGGCTCCAGTGACGACAATAAGTCATTGGTCACAGTGTTGCCAAACTTGTACTTTTGCAGATAGTCAGATACGCCTCTGCGGAAATTCTCTTCACCAATAAAACCTTCAAGCATTCGAAGAACAGATGAACCCTTGTTGTAAGATATAGCATCAAAAATCGCCGTGATCTGGTCCGGGGTAGCCACAGTCTGCACGATGGGGTGGCTCGAAAGTTTGGCGTCCGTCACCAACACAGAGTGTAAGGTTCTCGTCAGGAACTGATCTAGCATAGTCCATGATGGTTCAATAGCATTCAATGCTTTTACTTGCATGTAAGATGCAAAACCTTCATTCAGCCACAGGTCATCCCACCATTTCATAGTCACCAAGTTTCCAAACCACATGTGAGCCAGCTCATGGGCGATTGTGTTGGCAACACTGATCTTATTTTTTTGATGATGCTGTGCTCTCGTCAACCAGGAATGATGTCTCCCTGTAAGTGATCAGTCCCCAGTGTTCTGTTGCCCCAGAGACATAATCTGGGATGGCAATCATATCTAATTTGGGAAGAGGGAAAGGCACTTCGTAGTACTTAATATAAAACTGTGTTGCTCTTCTGCCAATGTCTTGTGCAAAATCAATTTTGTGCATTTGGTTCTTTTGCGCAAAAGATCTGAGAATAAAGTTGTTCCCGATGCCATTGGAATCTATTTCATTTTGCTTGTAGTCAAAGTCACACACGACAAAACAAGCTAAGTAAGTGGACATGGGGACACTTGTTGCAAAAGCAACTACTTCCGATTTGCCATCTGCATCTATTGTTTTAGATATTTCATTCATATTTGACAATGCTATGTATGACATTGGCTTCACTAAAAAGATGTTGAATGTGGCTTTAAAATCTGGCTCGTCAAAGCATGGGAATGTTTGCCGGGCATAGGTTGGCTGGAATTTACTGGCTACCATGGTCCTATTGTCTTGGAGGCGTGAAGAATAGAACCCGACAATATTTCTAGTAAGGCTTCCATTAAAATCAATGTCAATTCTGTAAGTTCCTGAATTAAGAACTTTATCAAAATGTATCAGAAGTTGTTCAATTTGCTTAACTTCCAAAAACTTGGACACCACAATCTCTTCATCACCTCTGAACACTTTCACAGAAGTTATATCTAAGAAGTTTGTGTGTAAAGTGATGTAGCTCTTTTCTTGTTTTACAATCACATCAATTTTCACGGATCCTTTAAATAATCCCGTGTCCATATTTGGGTGTAGGGTGAGATCATAATGAACTGGTCGTACTAAAGAAGACAGTCTTTCAGATAATTTGTACTTTGTTGTTGTTTCCATTGTGGAAACGGAGGAACTATGCACCCGGCGAATTTGTTGAACAAAAGTACTGCTCGACCGCAAATTACGAGTTAGACAAGAAAACTTGATTATATTCATAAAGCTGCTTTTAGATAGACTTATCACACACAGCACGCACAATTTTCATTAAAACTTAAGGTCAAAAGAATCAAAACAAAGATGATTTTGTTGTTTTTGTGGAAAAACAAGACAGACAGCAGACAAAAGGGGAGAAGTGTATGTCAGTTAGGCGTGGGCGTTGCAAAGACGAACCGTTTCGAAAGACCGTTTTATGTTTCGCGACGATGGAACTGTTTTACGTTTCGTTACAAAACAGTCTTCGTCGCACAACTAGTCCTCGGAACGTCTCAAGCGAATTTGTACTTAAAGACTCCTCAGATACAGTCTATAACGCAATGAGAGCATCGCTACGTCATCGTTCGTTCCGTTCGTTCTGATCTTTGATATAATAAAATAACCTTTATTCCTTGAAGCATAGGTGCGATTTTCGTATAAGACTACTAATTATTTCAATCTCATGTCTCGTTCGCAACACGGTGGTCATTGCTGTGTATCGTATCGTGATTGATATAGAGTGCAAGGTTGTTTAGCAATAATAATGTGGGGC

>OR35 [Moltype=mRNA] [Organism=Loxostege sticticalis], complete cds

CTTTGATTCTTCATCGATATCCGTTTCTCTGTAATATCTTGAAAGAAAACTAAGAAATATGATTGTAAAAAATGTCAATACGTCTGTTAGTATATCTTTGACCACTTTAAGATTAGTTGGTTTCTGGGTTCCAGAGCATTTTGAAGGAAATAAAAAGCTTCTGTACGATTGCTATGGTATTTTCAGCTTCATGTTCCTGTTAGGAACATACCTCATCATCCAAACCGTGGACATGTACATGATATGGGGGGACCTGCCCCTCATGACTGGCGTGGCCTTCGTGCTGTTCACCAACCTGGCGCAGACGACCAAGATCGTCTTCATGGTGCGGAGGAGACGACAGGTCCATGCCATCATCAAAGAAGCTGATCGGGAGTTAAGAGCAGTGGACAGTAACGAGGCGAGGGCGATTGTTAAAAGCTGTAACAAAGAGACCATTTTCCTCCAAGTGGTTTTCAATTGCTTGACGTTGGTCACCATGGTCGGCTGGGCGACCAGTGCCGAGAAGAACAAGCTGCCGCTAAGAGCTTGGTACCCATACGACATAACAAGGTCACCAGCCTACGAGTTGACCTACATGCATCAGATCGGAGCACTGTGCGTGGCAGCGTTTCTCAACGTGTGCAAGGACACGCTGGTGACGTCGCTGATCGCCCAGTGCCGCTGCCGGCTGCGGCTGCTGGGGCTGTCTCTGCGGACTCTGTGCAAGGACCTACGCACCACTGAACAGAATCACCTCAGCGCCGACCAGGAGGATATCGTGCGCGCTCGCCTCGCCAAATGCGTGAAGCAGCACCAATCAGCCCTGGAGGCAGCTCTGCAGATCCAGCGCAGCTTCTCGGAGCAGACCTTCGCGCAGTTCAACGTGTCGCTGGTCATCATCTGTGTCACTGCTTTTCAGCTCGTGTCGCAAACGGGAAACCTGGTTCGTTTGATGTCGATGGGAACGTATCTGCTGAACATGATGTTCCAAGTGTTCCTGTACTGCTACCAAGGCAACCAGCTTTCCGAGGAGAGTGCCCAGATAGCTGGTTCAGCATATGAATGTCCCTGGTACCTCATGTCCACCCCGCTGAGGAGGTCTCTCCTGATCGTGATGACCAGGACGCGCCGCATCGCCAAGATTACTGCTGGCGGCTTCACTACGCTTACTCTTGCTTCGTTTATGGCGATTATCAAAGCGTCATATTCACTGTTTACGTTGCTTCAGCAAGTTGAAGAAAAGGATTAATAATAAGACAAAAAAAAAAAACAATTTTAAAGCAGTTTTTGGTAGCGTCATTTGGTACATAATTTTTGTAGGTAGTATCACATGTCATGTAGGTAATCTATTTTAGTAAAAATAAAGTTCTGTTATGAATATAAAGAAATATGGGTATTTCATTACATCAATTAAGTAAATAATAAATTAGTCGTTAATAGCCATTTGTTGGTATAATAATTAATAAAATCCTACTTTGTCAATCA

>OR36 [Moltype=mRNA] [Organism=Loxostege sticticalis], complete cds

CAGTCATCTGCACCACTTAAATTTGACATAAACAAGAACAGAATATTAAGACAAACCAAATTTCTGATTGGGAATCATTTCTCAACGCAATGGATATTCCGGCGTTCGAAGACCTTTTCAAGGAAATAAAAATTAACTTGTGGCTCTTTGGCATTCCGTTCAACTGTTCGAGAATTCGACTCAGATTCTATTTGATGCTGATTGCTATAGTACTTATGATAATAGGGGAGAGTTGCTTTCTCGTATCAAGATACTCCCCTGAAAATTTGCTAGAATTAACGCAGTTGGCTCCATGTCTTTGTCAAGGCCTTTTATCAGCGTTAAAAATTTTGCCCATTGCAGCTAAAAAGGAGAAGATATTTGAACTGACTAAGTGTCTCGATCGACTCTACAGCACTATTCTTATGGATGCACATAAGAAAGCTGTTGTTCAAAGAGAAATGACATTAGTTAAAATCCTAATGAAATACTTCTTCATACTGAATGCAATTCTCATTTCGGTTTACAACTTTTCTACGTTGTTATTCATGTTCTACAGTTACGTTGTGAGGAGTGAAGTTGAGTTTATGTTACCGTATGCTGTGATTGTACCTTTTTCAACTGAAACATGGGTGACGTGGTTCATTGTTTACATTTACTCAATATCGTGCGGTTTCATCTGTGTTTTGTACTTCACAACAGTAGATGCCCTGTACTGTGTGCTCACTTCACACATTTGCAAAAACTTCGCTATTATTAGCAACGAAATACAAGGTATCAATGCTTCAAACGTTGGTAACCTAAAAGACCTGGTCAAGAACCACCAGTATGTACTCAAGCTGTCTGAAGATTTAGAAGAAATATTTAGGTTGCCCAATTTGTTTAATGTCCTGGTGGGATCTCTTGAAATATGTGCGCTCGGATTTAATTTAACGATGGGTTCAATAACCGAAAAACCGAAAAGTTTCTTGTTCCTTTCATCTGTGTTACTTCAAATATTGTTTATGAGTGTTTTTGGGGAAAACTTAATAAGAGAAAGTCGAAAAATTGGCGACGCTGCCTTTTGTAGTAAATGGTATGATATAGATATGAGTTCCAAGAAAACTATTCTCATTATCATGACCAGATGTCGCAAACAACAACAATTAACTGCTTACAAATTTTCTGTAATATCCTACGGCAGTTTCACAAAGATCATTAGTACGTCTTGGTCCTACTTTACCATACTGAAAACGGTGTATAAGCCTCCTGAATAAAAGAAATTGAGCTCTTCACAACGAGCCACCAAAACTTTATATTTTTCATACAATACTCCATATTCGCCTTTTTGTCTCGATGCTTATATTCATTTTATGTAGATAATACTCGTAGTTAGTCGTCCGTTATGCGTAGACACTTGTAATTTTCACACATATTACTTAAACTTGCTAAAACACCAAATATTTAAAATTAGATAAAGTTGTTGTAATTAGTCGATAATGTACGAAGATCGCCGTCAGGAAAACACGAAGACTGACTGAAGCCAAGGGTTTATTCCATTTTCGATACAAAAAATGTGATTGGACCGAGCAGTAGTGACCGAGCAGTAGTAGTTACTACGTAAAAGCACGAGCTCAGTGCCTTACACTTAGTGAAAACTTACCAAGCCTTAAGATTAAGGCCCAAGAAGGTCTCAACAGCTTGAAAGTCTGTTTGGAGCGCTCAAGAATCCAAGGAAATTCCTGTTTTAGTCATCATGGTTTTACCGCGAGCTAAAAGTAGTCAGCGCCATCTCTTGACATATTGCGAAACTAATGTATTGTCTCTGTTTACAACGAATTAATTGTGTCAGTTTACAAAGAAGGTTTGTCACAAGTTAACAATTAAATGGGGTTTCTGCACAAATAAACATGTTTTTTTCGTTATTTTCTACACCAAGCTATTGTATTGCTAATAATATTAAATCTTGTTTATTCAGTTACGAGTCAACTGCAAA

>OR37 [Moltype=mRNA] [Organism=Loxostege sticticalis], complete cds

GGGGACTGTGTGTTTGTTTCCGAAGTTGTTTCGTGGTCTCTTAAGTTTTGCATCGTTTACCATAATATGGATTTGTTCAATTATATCAAAAGGGTCTTTCGCGATGCGAAAAGTCGCTTGCAGGAAAATAGTTACGAAAGTTTGTTGTCGCTCGTAAATTTTGTTCCAAGTGTGGCCGGTTTTTCCATTAGGGGAAACACTATTTTTGTACCATTCTGGATATTGCACTTGTCTCTCCTATTTTACATCTATGGCGTGGGCTGCGCTGTATACCAGATCAAGTACGCAGAGGATGCACGCGACTTCATCAAGAGCTTCGTCAACGTGTCTCTCATCGTGCTCATTGCGAATAACAGCCACTGGTTTTTGCAGAAAAGATCCTTGCTAAAAACAGCTTTGAAAGAGATCAGTGAGAGCGACGTGATGGCCACTGCCAATGAAAGTTTCCGCCAGAAGCATGAGAGATCGGTGCAGAAGATTAAGAGGATCCTGTTTATCTTCTATGGTTTCAATTTATTAAACGCCACGTTCGTGTACTTGCCGCATCGTGCTGATGTGCTGAATAGCTACTCTATGACTCCGTGCTTCGGAATGGAGCCGCTAACTTCATCACCTAATCGAGAGATTTGTATGACACTCCTTTGTATACAAGAGATCACTATCATGGTCGTCGTTCTAAATTACCAGGCACTTCTTTTGCTTCTGATAGCCTACACTGCTCTCATGTATACGTTGCTCGCTGATGAAATTATGACTCTCAACAATTTCGATCGGGAGACTTATTACAATAATCCTACGGTGAAGCTTATCCTACCAGATTTGGTTAAACGTCACGCGATTCTTCTAAGCATCATTGATAAGTTGAAGGCTTTATACAGTGGTTCAATTGGGGTAAACTTCGGGTCGAATGCTGTGTGCATTTCGTTGTTCTTTTATTTGCCTCTTCAGGAATGGCTTCAGTTCATGCCAGTGCTAGTGTACTGTTTCCTGGTGTTCTTTCTTTATTGCTTTTTATGCCAGAGGCTGACAAACGCAGCTGAACTCTTCGAGATGTCTGTGTATGCGTGTGGGTGGGAGAATTTCGAGTTGAAGGAGAAGAAAGCGATATACTTCATGTTAAGACAGGCTCAGAAGCCTGTGGAGATTTTGGCAGCTGATATCATACCAGTGAACATTTCAACGTTTGCCACCACTTTGCAGGCGATGTTCAAGTTCGTCACCGTAGTCAAAGTTTAATAATTGGATAACATACATTTCACTTTTAGATATGTAGAAATGC

>OR38 [Moltype=mRNA] [Organism=Loxostege sticticalis], partial cds

GTACTTATTCAACTTCAGAAGGTACTTGTGTGACGCTCAAAATGAATTTGAAAATGAATTTCTTAAAAAGATACACTGAAGAAGACTTAGTTAATATTGAAGAGCACAACTTCGGGCCGTTCCACAAAGTCTACCAATGGCTAACTTTTACGCTCACTTTGGGTCTTTTGTTCCCAAACCCTGCTTCGGAAAGGTTCCGATTGACATTTATTATCGTCCTCCTTGTGACAATTCAGCCACTAGCTGTGATGATATTTATCGATATGTACAAGTGCTGGCAGGAGAGGGACATCTTCAACATCATCAGACACAGCACTATAATTGGACCGTTTCTTGGGGCTTTCTTTAAGATGTTCCTAATGTACTGGAAAAGGGTCCAAACCAAATCAATAGTGGACCAAATCAACAGCGACCACGAAGCTTTCAACCACCTTCCACGCAAACAGCAAGACATAGCGTTTCTCTACATAAAAGCTGGTGTTCGCAACGTCGAACGTATCTGGGCTCCTTTAGTCTCGGTTGCTATTATGATGTTCCCTGGTATGGCTGTCATCTTGACGCTATACAGTTACACATTCAATGACATACCCAAAAAATACATGATCCACGAATTAAACCCTCCTTTTTCTACGGATCCTGAGGACATGTCAAGATCGCCATATTTCGAGGTTCTTTTCGTATACGAGACTGGGGCTGCCATAATTTGTGTGCTGAACTATACTGCCTATGATGGGTTGTTCGGTGTAGCGACGAACCATGCTTGTATGAAGATGAGCTTGTGCTGTGTGAAGTTGAATGACGCGTTCGCGTGTGAAGATCAGGAGGAAATGTATAAAGGCGTTCTAGCGTTCATTGAAGAGCAGCAGAAGATGTATAAATTCGTCGACCTGATTCAAGAGATTTTCAATATATGGCTGTTTGCTATTTTGATGAGCACTATGATACAAATTGGATCTTTACTTTTCCATATTTCAGCGGGCTACGGGTTCGATCTGCGCTACACGCTGTTCAGCTTCACTTCTGTTGTCCACATCTTCCTGCCTTGCAAACATGCGGCTACACTGAAAAGCATGTCGACAGAAATGGCGACAATGATCTACATATCCGGCTGGGAGCGCTCCCGCGACCGGCGCGTGCTGCGCATGATCCCCTTCATGCTTGCGCGCGCG

>OR39 [Moltype=mRNA] [Organism=Loxostege sticticalis], complete cds

CCAACAGTTGAGTCGTCTTGTACGAGGTCATAAATTGTAAAATTCAATTCAGAAATTAAAGGAAAAGTTCTGAGAACGATTCAAATAGAAATGAATGAACAATTGTCATACAAGAGTCTGGCTCCTCATGTGAAGTACTTGAGGGCTGCAGGACTCTTCAGATTAAGTCCAGATTCGCCGAAACGACATATATTTTTCCACACGATCTATTTGCGGTGGCTCATTGCATTCTTCTCTGTGTACACTGTCCAGCAGATATTGAAGATTTACGAAGTCCGGGATGACGTGAACAAAGTAATGGACACCATGTTTTTATTTATCACCAACACTGACTGTATTTACAAAGCTGCTGTTCTTCAAAAGAAGCCTGAGAAGATCGAGGAACTTCTGAACACTATGAAAGGACCAATATTTAACCTAGGGGTACCAGAACATCGCCCAATATTATTGGCCACGGTCAGAAAAGCTCTCCTGCTCGTCCATATGTTCAACCGTCTGTCTCTGATCACCTGCTTCCTTTGGGCCTTGCATCCGACTATTATGCACATGCAAGGGAATCCTATTGAGTTCGCAGTGTGGTTGCCGTTTGATGCCAATCAGGACCCACAGTTTTATATTGCTGTTGTCTACGTTTGGATCCAGACCTCTTGGCTGGCCTACAGCAATACAACGATGGATATTTTCATCGCTTTCCTACTAGAACAATGTAGGACGCAAGTTTCTATTCTCAGGCTGGACTTAGAGAGCGTGGTACAAAAAAGCAAAGAAGAAGCGGCTAGGACATCGTCCCCTTATTCAGAAATTCTGGAGAGGCGATTCGGCAGAATCTTAATACATCACAATGAAATCGTAAACTCGGCTGACAAAATTCAGGATATATTTGGAGGGGCTGTGTTTTATCAGTTCGTCATAGGCGGATGGATTTTATGCTGTTCTGCCTACAGAATAGTCAATACTCAACCAGCCTCAGTAGAATTCGTCTCAATGCTAATGTACACTACCTGCATTGTAGTAGAAATCTTCGTGTATTGCTTTTTTGGAAATGAATTGTTCTATGAGAGCAACAAGCTTATGGACTCCGCTTACGCGGTAGACTGGCTGGAAATTCCTGTGAAGCAGCGAAGATCCCTCATCATCTTCATGGAGAGGGTCAAGAGACCGATATGCCCCACGGCGGGATCCATGATACCACTCTCTAACAGTACATTTGTTTCGATTCTCCGTAGTGCGTACTCGTGTTACGCATTTTTGAGGAACTCTGAACACTAAGCATATATTCGTAGCTGTAGAAGCAACATTTAGATTTGATAGCATTAGCGGAACGTTATGTTTTTTTTTAAATTGTTTTATGATCAATGTTGTATTGTTCCTACTTACAGGCTTACAGCTTAGCGAACACACAGAAAAAAAGCAATAATGTTTTATAAGGCTTTTTTGAATTGAAATAAAAGTCGATTTTCGTTTAAAAAAAAA

>OR40 [Moltype=mRNA] [Organism=Loxostege sticticalis], complete cds

TCGAGCACCTGAGAAAGAGAGAGGTTGTTTGTTTTTTTTTCTTTCACTTTCTAATTTTGTGCTTTCCTATTTCGTATGTACAAGAATATTTAAATGTAAATAAACAATAAGTCAGCACTGAGAGATAACATTCAGTCTTATAAAAACTTCGGTCGATTCGAGGCACCCGGGAAGAGTTCAATAAACTCGAAACGTCAGGACTGCAATGCAAATGTCATGTTGGTTTTGGTGTTTTTTACTGTGAATGTTTGATGTGTAAGTCGTAAAAAGCTGTGTATTTCCGCGTGGTTTCATCTCGTTTGCTATGGAAAACCAAAACCCTCAGGAGAGGTCTGCTCCAATTCAGTATGTTCGTGGATCTAGGGCGTTCCGTCAGTTCAAGAATCCACCTCAACCGCACATGTGTATTCAGGATACTATAAAGGATACGACGGAGAAACTATTCATAAACGTGTTGGGGTGGCAGAAAATAGCTAATCCTAAGCAGTACAATGATCCTATCCCCTTGTATGGAGGAATGCAGGTGCCTCAAGGATGTGGTCCAAATAGCAACAGACCTCCACTCTTAGTTTTTGCTGTAATGGTGAATCCAGACATTTTGAAAGCAAATGGCAAAAATGCAGCTAATCCAAATAGCAACAGACCTCCACTCTTAGTTTTTGCTGTAATGGTGAATCCAGACATTTTGAAAGCAAATGGCAAAAATGCAGCTAATCCAACTGACAGAGAAGCCTTGGTGAGTCTCCTTTGTGACTTTGTAGAGGCCATGAACCCAGGGCTACTGTTGGCACGGAGTCCTGTTATCTTGAAAGACCGGGACCTTGCTGGTGAACTGAAAGATGTTTGGCTCGCTGTCCAGAACAAAAGGGAAAGAGAAAAGGGGTTGAGTCAAGATGTTATGTATAAGGTGTATGATATTGATGGAATTGGCAACGAGGACAGCAACGAAGAAGATAAAATGAATTCCAAAAATTGCAAGCAAAACGACTGCGACTCATTGAATAGATCGAGTAATGACAAGAAGAATGTGATTAAATCATCTAAGCAAATACTAATGAATGCTGGGCAGAAGTCTGAATTTGATTCTGGAATGAACAATTGCCAAATCAATCAGAACCCGAGTCGAGAATATAGATGTAGCACAACAAAAGCAGACACAACATATTGTACTCCAGTGTATGGTCAGATAGTGTCCAGTAGAGAAAATCACAATCAAATAAATGATATACAACAGAAATTTTCATCCAGTGAGACAACAAAACCCTCAAGCTCCTTTAGAAAAGACTGGAATCCAGTTCATGGGAAAACAACTGAAGGCTGGGACGAATTTTCAAAACGCAACGTATCCTCTATAAGCAACGAAGGTGATGTGAAAAAGCAGAGGTATATCAAAAATGAAAAAGGTAATAAGGAAATGAGTAATGGTAAGTCTCAGTATGATTTCTTTCCTGTCTTTGACAATAAAGCAGTTGATGAGGTTTCCGACTCAAATGAAAAAAGTGGCACACCAGATAAAAGTAAAGAACAAGAATTAGGTAACGAAGACAGTTCTAAAATAATATTAGATGCAGTGCAGAAGTTAGTTCTGCAACCGACTGACAACAAAATATGTGATAATAAGACAAGTGCTCTCAGCTCAATAAGCTCATAATTTTAAAGCTAATGAACAATTTTGTATGGCAGTTAAATTTGTATTGCTTAAATGCTGTTTGTGAGACGTGTTTATTGAATTATAATGGGATGTGACATAGCAATATGAAATGTTCACAAACAACTAGTCAGTCCTTCAAAATATTGAATAAGCAGTTTTGAATTGCTTGTCAAAAAATATAAGTAAATAATCCTTTTTTATATGACACAGATTAAAAAAAACGATTGTTTTCACTGAATATTTGTGTGAATTTTAAATTCAGATATTTACGGTAGGATTAAAACTTTTGCAATGTTTGATATGACTTGACGAATGACGATGGATTTAAGCTATTGTGCATGCAGTGTGAGAGTTGTTTGTTTATATGGTTGTGTATTTTCATAGATGTGCGTGAACACGAACAAACTGACGCGTGACTTGTATTTATTGATGAAGCTATATGATTGGTGAATTTTTATATTGCTATAATAATAAAATATGTTTCATTGTTGCCAATTTGAAATTTAATATTTAAAAGTTGATACATAATATAATTGATCAAAATCTTATAAGTTTTAAAACCTTTTGCTCATTGAAATTCTAAACATTTTGCATTAAGTAAATGGAAGTTGATCTCACTTTTAATAATTACATTAACCTGTTGTGTCTAGATGAACATCGAACACTGCCAAACTATATACCTTTTCACATACCTACTCACATGAATTGTTTTAGATCTTATCTCCGAGTAAATAGTCAGATAGTTTTAGTAACCAAATATTGTTAGGGTAAATTTTATAAGCGTTATTTAGTCAGCTGATAATCCAGTCTAACTTTTAGTTTTTCTTACAATGTGCAATCTCTTGGTTCTCATAACTGTTTATTTTGTTAAAGTTATTTTAATTTGTATCTGCTGTTATAATATTTATTTACAAATAAATTAGTTTTAATAAGTCTATTTTTTTCATGATTTGGTATAGTGTGGTTTAATTAGTTATTTTCAGGGCAGTTTTAACAATAACTACCATTTAACTTTTTTCAATAAAAT

>OR41 [Moltype=mRNA] [Organism=Loxostege sticticalis], complete cds

GTTCAAGCCACAGTTAATTGTGTTCTTTGCAAAAGACTAGTGTAGTAAAAAGTAGTAGTAAAAAGCATAAAGTCAAGAAGTCTCTGAAAATTCATCTCCATCAAAATGCCCGAAAAATCTTACGGTACTGTAAAAAGCAATCTGCGCGAAGAGTTGAATTACATAAATTCTATGGGTTCCAAAATATTTCTTTACCCTTTTAGTGGAAGATCTAAGCTAGTTGACATCTGCTACTTGTTTGTGTGTTTTCTAGTGGTCGTGACAGCTACTCAACTCTTGACTGCGTTGCTTGTGACTGATTTGAAGGAATGGATAGAGATAGTCAACGTCGCACCCAATTTAGGCGTTGTTCTGATGACATTACTGAAATACACTAAAGTGCACAACAATCAGCATGTTTACAAAAAGATATTCAAGCACTTTAGCGACGACCTATGGGATGTTGTTTTTGATTCTTATGATCATAAGAAGATCGTGATACGCTATACTGCTATTGCGAAGTACGCAACAAGATTTTTGTTCTATTACTCTGTGCCTCTTGTCGTTTTCGTCGATTCTTTTCCAAGGATCATAATGTATTTAGAGAATGAAATTATTGGGAATGAAAACCCACAATATTTATACCCATTTGACGGTTGGTATCCGTTCGATAAAGTGAACTGGTATTATACTGCTTATCTTTGGGAGAGTTTCATGACATTTATTGTTGTCTGCGTCTACGCATTCTCAAATATGATCCACGCATCGTATACCTCGTTCATATGTATGGAACTAGAAATTTTGGGAGTAAGCATCAAAGATCTAATAACTCCTGATGATGTCACCAATATAACAAACCATTTAAAGGTTCAGGAAATTCATAGCCATATAAAACGAAAACTGAAAACGATAATTAGAAGACATCAATTTTTGGCTCAGCTTGCTTCAGAGTTAAATATAGTGTTGGGGGACATGATGCTTCTCAACTATATTTTTGGATCTGTTTTTATAACGCTTACAATTTTTACTGCTACGGTTGTGGATAACATGTACAAATCTCTACGGTACTTTTTCATGTTTTGTTCGTTGATTGTGGAAATATTCTTCAATTGTATGATTGGACAAGTCCTTAGCAACCATAGTGAGCAGTTGACAGATGCAATATATTCAGCCGACTGGCCATTTGCCGACAACGAGACGAAAGTGATGCTGCTGATCCTCATGAGGAGAACCCAGAAGCCGTTCGAGTACACCGCCAATGGCTACCTGGCTATGAATCTTAACAGTTTTAGCGGTGTTTGCAGCATGTCATATCAATTATTCAATTTAATCCGCACCGCTTACAGCAAATAAACATTTCTAATTCTAATTTCTAATTCTACAAAAACGAAATAAAAAAATGTTTTAAGTACAAATATTAATTAAGTCTCTTCAATACTAGTTCTAGTAAATATTTCTGAATATGTACCAAGACTAGATGCTTAAACCCCTAGATCACTAGATCTTAGGTGTTATAAAAATGTTTTATCAGATTGAGGGCCCCTGTCGCAGGTTCCTGATGAGTTTATATCAGCATCAGGAACTATAACCAGAAGGCTATCTGACACCTGGAACTTTATCAAGAGCCTGTGAAACGGCCCACACGGGTCATGAATGTTTAATACTATATCCTTTAAAATAAGAGCACACACTACCCACTACAAAACGAACAGCTAATTTACAGTCGAAACAAATAATCGATAGTTCTAGTTAAGCACGTTTTCCCGGACACATCTGCAAAATATACGAAGTAATTTCACGTTTGCCTAAGGAAATAAAAGGAAATATGACACCTATATTTTGCAGCAATAACGGCCCAGTGTCAATTGGACGATATTGAACCGGGACCATAAAATCAAGATGACTACATTCATACTACAAAGAGGTAAACATTCAGCAGGCTCATATATTTTTGGGGAAAAAATGGAGCCTTTCTCTTCCTTCATGACGAGACAGGACTGTAAATTCTGAAGCAAGCTAAAAAACTTACAAAGTCGTTTGATGCAGTTAAATTATAGAAATAAGCTGTGTGAAACTGTCATTTACTGTTGATCAGGCAAACCTAAATGCAAACTTTTAAAATTACGACCCAAACGAAAGAAAAAATGAGACTACCACTTTCACATAGAAAAGTTGCCGCTCTAACTAGTCCGGAGCTAGAGTTAAACTAACATTGATCCGGCAAACCTACTTTTTGTATGTTCTCCAAAAGAAATGGGAAAAAAGTGTAATCTTTGAGCGCAGAGGGCCCAACTCTAGTACTAGTACTTATTTTTAGAGAAATACTTAAAAATATAAATTGCAAACCGCGCGCGCTGTCCAAGTTCCACATCCACACTTGTTACAAATTGTTACATGTAGCCAAAAAATAACAAATAGTTATGTGTGTGTGCTAACAATGTGTACTACCTTGATTTTTGTGTTCAGATACTTTCAGCGACAGCCCGAAGTTTGGAAATTAGTGATTATGATGATGATGACCCCTATCTAGGCCTAGAAAAGTACGAAAAGCTTCTGCGTCTAAAGTCTCTCTGGCCATGTCAGATTGCTATTCCAGTGAGTTATTGTAAGAGTGAAGAAATTGAAACTGTGTTTGCACACACACTTCTGCACTTACATAAGTCCTGCCCAGAAAGCTAGTCGTTAAGATTGGCTGCCATATTTGAAATTCGGTTAGGGGGACTATTGGCCGATTGTACCTATTACATATTCGTTTTTGAGCCTACTGTGTAAGCGTGTAATATGTGTAGCAAGTGTTTTTCACTCTTTGAACAACCAATATTGCTTAGTCCAAATCTTGTATACTCGTATTATGACTAGTAGTACTGTAATCTTAGAACGTTTAAATAGTAATTTATGGACAATAAGATGCATAATTATTAGAAATTAGTTTAGTTTTATGTTAGATATTTC

>OR42 [Moltype=mRNA] [Organism=Loxostege sticticalis], complete cds

TAGATTTCAAAGACTCTAGTCACTCTTGTAATCGTGTAAAATGAACACGACCCTGGGAACTCCAGTCAAGACGAACAAAAGCGCAGGATTCTTCTTAAAAGTCTGTCAACTGTGCTACCTCTTCGGATTCCCAAACTGCTGGATGGAAAGCCTCAAGTTCTCCAAAACGTTCACCAAAATATACGATCCCTTTTCCAAATTAACAAACGTAACGATTTACTTATTCATACTTGCGGAATGGGGGTCGATGTTTACTCAAAATAATTTGACGGAGAAGCAGCGTTCTGACAGAATCATGTTCTGCCTGTCTCACCCTGTACTATGTTCGTACCGGGTAATCTTGGCATATCATCGAGAAAAGCTACAAGAGTTGATGTATAACCTCTGTTTGGTACTTAAGGAGAAGGTTAACGACGAGGAAATAGAAAAGGGGATGGTCAGAAAAGCCCTAGCTTACACTAGTGCATTAATAGGCCTCTGCTCCACATCCCTTTTTCTTTATGGGGCTGATGGGTTCAATCAAATGATGAGATCAGAGGCCACGTTCACAACGGTGATTACCGCATGGCCACTCGTAGAAGACACAAGTATCTCCGCTAGCGCAGCAAGATTCTTTCTTTACTTCATGTGGTGGGTCTTCATGAGCCGTGTCTTCGGAGCCTTCGCGATGCTGATCTCCCTCATAGTAGCCCTCGAGCACCAGTACAAAAACTTAGGGAAATACTTCCGAAACCTTTCCGGGATCTTCGAGCAAGATTTGAGTCAGGCGCAAAAGGAGAAGGAGTATGACCAATCTGTTAAATACGGGATCAAACTACATGCCAAAACTCTACGGTGTACCCGACTAGCCCAAGATTCGTTTAGCTCAATCTTCGGAGCACAAATCCTTTTGAACACATATGTTCTAGTCTTACTCATGTTCCAAATGGTGAGCTCGGAGCGCACTTTGGCCAACGTGTTGGCGGTGATCGCGACCGGAATCGCGATGCTGCTGAGCACCGGCTTCCTCATGTGGACTGGGGGAGACATCACCGTGGAGGCGGCGATCTTGCCCACGGACATGTATTGCTCGGGCTGGCACAACTGCCGCTCAGCCACTGGCACCAGAAAGCTGCTGGCGCTCGCCATGTTGCAAGGACAGAAGCCAGTTATGATAAAAGGTCTCGGATTTATAACGATTTCGTACCCCGCGTATCTTTCGATCGTGAAATCAGCATACTCCGTTTTCTCAGTTTTGTACTGAAATAATATGTATTATTAACATGACAATTAATTATGCAATCGCATGCAAGATTAGTTATTTAAGTCCATTTCTCAATCGTAGGTAGTAAACCAATTAGTTAGCAACAAACCTTAAAATAAAAC

>OR43 [Moltype=mRNA] [Organism=Loxostege sticticalis], complete cds

AAAAAGCAAACGTTTAAAAAAGTACTTAAAAATGTGGGAAAATCTACATAAGTTTGGTCTGTCTCTGGAGTACTGTAACTTGTCCACCATGTTGTGGAACGTGGGGTTTCTGCTCCGGCCGCTCACCCTCAACGTGGACAGCCGGCACAAGGATAGGATACCCATCAGCTCCTACGTGTTTACCATCACCATAGCATCATGCTACTTCTACGTATACCTGTTCAACATGCTGTGGTTCGTGTTTATCAAGTGTCGGGCGACCGGTGACCTGATCACGGCAATGCTGGTGCTTTCTCTTGGGATCAGCAGTGAGATCGGGCCATGCAAGCTGTTCTCTATGCTTTTTTACAAGGAAACAATCAGAACCATCGTTGAAGGCTACCTGATTTGCGATGCTCAGACTCTGAAAAGCGACCGATTCTCCAGAAATTTGCTGAAGACCTTGAGAGACGTCAAGAAGCGGGTGCTGATATTCTGGGTGGTAATAATCGGAAACGGATTATTCTACATAATAAAACCCATCGTCCTTCCCGGGCGACACCTCACAGAAGATCTGTTAATTATTTATGGACTAGAACCGATGTATGAAACCCCTAACTACCAAATTGCGTTCTTTATGATGTGCTGCGGCACCACCTGCTGCTGCTACCTCCCAGCCAACATTGGAGCCTTCCTCATCATCCTCGTTGGCTACACCGAGGCCACCATGCTGGCACTTAGCGAGGAACTCCTAAACCTTTGGACCGACGCTCAGAGCTACTACACAAATAACCACGAGGAAATTGAAACCACAGTTGACAGTGCAATGGTCACCCCAAACGATGCCGAAGCAAACCGAATCATGAACACATATATCAAACAGAGCTTAGAAAATATAGTCAAAATCCACACAAAGAATATCGGTCTGATACAACAAGTGGAACACGTATTCCGAGGGGCAATTGCTGTGGAATTCGTTCTCGTTATATGTGCTATCATATCAGAACTACTAGGAGGTTTGGAAAATACATATCTTGAAATGCCACTGACATTTATGATAGTGGGTATGGATTGTCTGATTGGTCAAAAGATGATGGATGCCTGCGACACGTTTGAAAGTGCCGTGTACGATTGTAAATGGGAAAACTTTAATGTAGCAAATATGAAGACGGTGTTGATGATGCTTCAAAATTCTCAGAAGACCATGGTCTTGTCTGCTGGAGGAATGGCTACACTGAGCTTCAGCTGTCTGATGTCCGTCTTACAATCAACTTATTCGGCCTACACCACTCTGAGATCAACAATGTAGAGCACCTAGAACACCTATATATTTTTTTTATTGTTTAACCGTAAAAAGAAGTGAAATAAACAGTAGACAGAATTGGATTTTAAGTAACAGATCGACTAAAAACAAACAATTAGCTGTAGTAGGGTAGACAGTATTTTAGGTTTCACGATGTAAATAATTTGTAACTAATATTTTGAAAATAAAAACTAAGTATCCTTTT

>OR44 [Moltype=mRNA] [Organism=Loxostege sticticalis], complete cds

CAGTGTTACGGTTTAATAAGCACACTACTTCAGTTATTCTAAAATCGCATATCGCAATCTAAAATCACATAATCGATGCCAAAACCAACGGTTTGAAATCAAGATACTAAGTGACTACAAATTCTCATAGTTTTATCAAGAAATACTATCCTGGCTTTACTGTTAACACATCCAGACATCATGGGGTTGATATGTGAAAAAGGCTTTTGTCGCATTATTCATTAACCTACCGGTCCATTGATAAATCGTAAACATGATAGAAGAATCACCCTTCGATAAATCATTGCAGAAGATACAATTCGCATTTCGGTCAACAGGCTTGAATCTAGGAACGGACGGTAGGAAAAGAAACTTTAAGCAAAACTGCGTCTACCTTTTCAATTTTCTGTGGCTGAATACGGATATCATTGGTGCCTTATCATGGCTCTTAGAAGGCATTATTAGTGGAAAAAACTTTACAGAACTAACTTATGTTGCTCCCTGTCTAACTTTAAGCATACTTGGAGATATAAAAGCTTTCTGTCTCCTTCTGAACGAAAGAAAAGTGCACAACCTTATAGATAACCTCAGAAACTTGGAAGCTAAATCAAAGAACTTTGAGAATTCTGAATGGGACAACATTATGCAACCGGAAATAAAACTTTTTAATATAATCATCAAAGTATTAAATGTTCTAAATTGCCTGATGATTGTAGTCTTCGATGTAAGTCCCCTTATTTTGATAGCAGTGAAGTATTTTACTACCGGAGAGTTGGAACTTTTGCTGCCATTTTTGGACGTTTATCCATTCGATTCGTTCAATCTGAGGTATTGGCCTTTTGCGTACATCCATCAGATTTGGTCAGAATGCATCGTCCTCCTAGAGATTTGTGCCACAGACTACTTCTTCTTCGCTTGCTGCACTCACATCAAGATCCAGTTCAAGCTCCTACAGCATCAGTTTCAGGAGATTATATCTGCTAAAAGTGTCTCAGCAATGGATTCTGAGGACCCAATTGTTGTTCGAGCTAAGTTCCAGGAGTTGGTCAAATGGCACCAGGAAATAATTAGTTGCGCTAACAAGCTCGAAAAAATTTACTCCTTTTCTACTCTCCTTAACTTCTGTACCAGCTCCTTGGTCATTTGTCTCACAGGCTTTAACGTCACGACAATAGACGACAAGGCATTTGTGATAACCTTCATAATTTTCCTTTCCATGAGCTTATTGCAAGTTTTCTTTTTGTGCTTCTTTGGCGATATCCTGATGCGTTCAAGTATGGATGTAACAGATGCTGTGTACAATAGCCGATGGTACCTCAGTGACGTGGCGACAGGCAGAAACGTTCTACTGGTCCAAACCAGAGCTCAAACCCCTTGCAAGCTCACTGCTGCTGGCTTTGCAGACGTCAATCTTAATGCTTACATGACGATCCTCAGCACAGCTTGGTCTTACTTCGCACTGCTACAAACAATCTATGGTTCACGTTCTTGAATTACATACACCGCCGACGGTAGCGGGTAATCCCGCCAGGGGCAAAATGTTTGCGTGATAAGCACGGGCATTTGTACCCATGTAATCATGGATGTTATGTTATGTAATGTTGTAGTCATGGATGTTATATGAACTGAAGTATTTTTGGGAGACGCTCCCGTCAAAACCGTTGTCTAGTTTCTCGTAACCCAGAATATTAATTCCTTTCTTCGAGACCATGAAAA

>OR45 [Moltype=mRNA] [Organism=Loxostege sticticalis], complete cds

CTATTTCTTCTTCACCTGCTGCACCCACATCAGAATCCAGTTCAAACTACTCCAACACCAGTTCCAAGAGATCATAGCCAACAGGAGCATTTCTGCAGTAGTTTCCATGAATCAGATGTCTATTCGGGCCCAATTTAAGGATTTGATCAAGTGGCACCAAAACATTATAAGCTGTGCAAATATGCTGGAAGAGATTTACTCGAAATCAACACTCTTTAATTTCTTGGCTAGTTCCTTAATGATTTGCCTAACTGGTTTTAATGTTACGACAGTAGACGACAAGGCAATTGTGGTGACTTTCATCATTTTTCTTTCGATGAGCATGATGCAAGTTTACTTTTTATGCTTCTTCGGCGACCTCCTAATGTGTTCGAGTGCAGCAGTGGCAGATGCCGTATATAACAGCAGATTTTACCTGGGTGATGTGGTGATGGGAAAAATCGTTCTGCTAGTCCAAACTAGGGCCCAAAAACCTTGTAAACTAACAGCTGCTGGCTTTGCAGACGTCAACCTTAAGGCTTACATGAGGATATTAAGCACATCTTGGTCTTACTTTGCACTTCTACAAACTATCTATAGTTCACGCTGTTGAATAAAATTATTTAAATATAAACATTTTTTTTCACAACAAAATATATGCTACAGCCGTAAATGGAC

>OR46 [Moltype=mRNA] [Organism=Loxostege sticticalis], complete cds

ACCACCTTCAAGAGAACATCAAGACTTTACAGCAAGAAACAAAAATGAAATTTGTCATCAAAAATACTTTCAAAGCTTCGCAAATATCGTTAACATATCTGGGATTCACTGGCTTTTGGACGAGAAGAAGCGAAGTTAATTTTGTGTTGAATTATTGTTACTGTTTTGTAACATTTATGTTTATGACTGGTATATCCATCATGGCACAGTTCGTGGATTTAATAATTATCTGGGGCGACGTGGCTCTTATGACTGGGACCGCTTTTCTTTTGCTGACCAACGTGGTACTCGCCCTGAAGGTACTTAACATGGTATGTAGGAGGGAGGAAATAAGAGCCATCGTTGAGGAGACTGATGGACAGCTGCAGGCTGTAAATACAGATTGGGGGAAGGAGATTGTGAAAAGCTGCGACCGACATCTAACAGTGTTGATCTCCATCTACACATGTCTGTCTTACCTGACCATCATGGGGTGGGCCACTGGCCATGAAGAAGGGGAACTGCCTACACGAGCTTGGTATCCGTATGATACTACTACGTCACCTGGATACGAAATCACAAGCTGGCAGCAGGTGGTAGGAGTGTGTCTGGGTGCTGGTGTGAACATAAGCCTGGACACCGTAGTCGTCTCTCTTATGGCTCAGTGCTGCTGA

>OR47 [Moltype=mRNA] [Organism=Loxostege sticticalis], complete cds

TTCGACCCTTCGCAGAGCTGGTTCATATACTGTCTGGTATACCCATTTGAGATGTATTGTATGTTCCGCTTCATATATGCTTACTTAGGTGCAGAATTCATAATGGAGGCACTGTGCTCTCACTTGGTCACAGAGTTCAGGCTTCTTCGTGAAGACTTGATGCTTATCAAGCCTGTTCCAAATAAACGCTCTAGCGAGGGTATCGATGAGATCGGGGAGTTTGTGAAGAAGCATCAGAAACTGACTTTATTGAGCAAGCAACTGGATGATATCTACAATAAAGTAAACTTCATTGTGTTGCTGTTTGCGACTGTGATTATCGGTTTTTTTGCTTTTGCTGTAAAGGTATCACATGGCTATAAGATGCTCGTCAATTCTTTAGCAGTTTACGGGATGCTCTTGCCAGTATTCATTATGTGCTACTACAGTCAATTATTGGCAGTAGAGAGCGCTGGTATCGCCGTCTCGGCTTACAATAGCCCTTGGTATAAAGGAGGCACTCATCATCAGAAATCTATTTATTTTATAATTAAAAGGGCCCAGTTACCGTGCTATTTGACTTCTTTAAAGTATTCACCAATAACTTTGAAAACTTTTAGCAAGGTATTGAGCACAACTTGGTCATACTTTTCTCTGGTAACAAGAGTATATGAACACGGAAATGAAGGTTAAATACTACCTACCCCTAAAGTTTTAACACACTAATAGGACATTTATATATACATCAACAGAACTGTATGTGCCGGCCCACTGTAGACCTCCACAAAGCCCATACCCGGA

>OR48 [Moltype=mRNA] [Organism=Loxostege sticticalis], complete cds

CGTCACTCCGTACAACTGCATTGCTAATATTTCCGGTGGAAAGATCAACATGCAGCTATTGACAATGCAGCTGCTCTGGTGCTCAATACACTTCGTCAGCCTGATCGTGATGGTGGAGCCGTGCCACATCACACAGCGAGAGATGGGCCGCACCAACTTTCTGGTCAGTCAGTTGATGCTGCAAAACACCGATGAACTGGTAACAAACGAGCTGAATGTCTTCGGCCGGTATCTCTACCTGAACGATGTCGTCTACTCGCCTATGGGTATTTGTGTTCTGAGCAGATCTCTAGTCGCTTCGATCCTCGCCAGCGTCACAACATACCTCGTCATAATGATGCAATTCCAAGCCACAGAGAATATAGTATATCATGGATAAATTGATCAGAATATTTCAACAAAGAAAAATAAGTAGTTATAAGTGACATACTAAGTAGGTACATAATCATCAAATGTTAG
