# Supplementary material for: Identification of candidate chemosensory genes by transcriptome analysis in Loxostege sticticalis Linnaeus
Source: PLoS One. 2017 Apr 19;12(4):e0174036. doi: 10.1371/journal.pone.0174036 (PMC5396883; doi:10.1371/journal.pone.0174036)
Supplement: S2 Table — (DOC) [file pone.0174036.s004.doc]

**Supplementary material 2**

**Protein sequences of all olfactory genes constructed the phylogenetic tree**

**OR:**

>LstiOrco

MMTKVKAQGLVSDLMPNIKLMQAAGHFLFNYHSDNSGMTTLLRKMYSSVHAFLIVINYLCLAANMAQYSDEVNELTANTITVLFFAHTVIKLLFFAVSSKSFYRTLAVWNQSNSHPLFTESDARYHQLALTKMRRLLYFICGVTVFSVMCWITITFFGESVRYIANKETNETLTEPAPRLPLKAWYPFDAMSGTMYVVAFVYQVYWLLFSMAIANLMDVMFCSWLIFACEQLQHLKAIMKPLMELSASLDTYRPNTAELFRASSTEKSEKVPDPVDMDIRGIYSTQQDFGMTLRGAGGRLQNFGQPNPNNPNGLTQKQEMLARSAIKYWVERHKHVVRLVASIGDTYGTALLFHMLVSTITLTLLAYQATKINGLNVYAFSTIGYLSYTLGQVFHFCIFGNRLIEESSSVMEAAYSCQWYDGSEEAKTFVQIVCQQCQKAMSISGAKFFTVSLDLFASVLGAVVTYFMVLVQLK

>LstiPR1

MKSYRILIRKFGREFHLEHYTHMGQIYEDMNKKINVISVYFTRFMMCQMILAMIMFNIAPMYNNITNRYIRHTENYTLEFSLLISYPGFKPLNYFATTTVYNFYLSYNCGVMLSGLDLILSLLIFQTIGHVKILRHNLENFQSPKNKVVIKLDEPHKYKFHGSCLYEVFDEEENEKIRIKLAECVEHHRQIINFTDELSELFGPFIAFNYLFHLVGCCLLLLECTGNDGGMLRFGPLTTVVFGQLIQISCMFELMGSEAEKLKDSAYMVPWESMNVSNQRTANIILHKMQYKISLKALGLAAVGVNTMTGILKTTFSYYAFLQTL

>LstiPR2

MKNKSPLTLKYIKIIRSFLRPPGGWPSEVFGEKLSLAIRFHRVTLPFHTSLIVIGGFYHLYDNVHRLSFLEFGHIIITTLLAMVTVLRSVLPNLQKFNSLLSKFINDFHLMHFTHKGEYFEKMNKMVDLISNYYTMVSTCMMYVGMLMFNIGPTFNNVRNTVFLKTENYSMEYSVYYSYPGFKPLDYVTIASIYNCYLSYNCSTLLCGFDLLLFLMIFQTIGHVYILRHNLENFPSPNNKIMLTFLGDKYRNKEGCICEKFDPEENKLVSLKLAECIEHHKIIISFTDDLSQIFGPILAFNYFFHLVSCCLLLLECSEGGLDAVIRFGPLTLIVFGQLVQMSVIFELLGLETEKLKDSVYCTPWESMSVSNQRTVCIILHKMQYKINLKALGLAAVGVSTMTGILKTTFSYYAFLQTMGE

>LstiPR3

ICLLCSNARHPTDLHYMKMIRYQLRMICSWPQKLLGEEVKSVPLRNTRFLFIEGSLVAFLGLVYIKTHHTRVTFLEMGHTYLTVFLGIVAAQRVTVSWFKSYDQTMKNFVLELHLFQHRHKSEFHEHMYQYINKICTVFVDFIHVELFMGIILFTLTPVYNNYMKGMFNRATPVGPDKFFEHSINYSLPYINQFIYDELVSYLFIAILNVLFAYDAGICFGSLDVTLSVIVFHIWGHLKILDHNLRSIPKPVNELTYTAEENKKVGGILKNIVDHHRMIMSFMTNTSDAFGPMLCLYYMFHQVSGCILLLECAELDAKSLTRYGALTVTIFQLLIQISVIVELLGSQVRVNSISQMVFKTRTNCLTNQRVRYVL

>LstiPR4

MHKLRMFLISDGSDLKDVKRVVDIKYIQVLRSYLRIISAWPAKHVGDTPTKWDRIKGNPVLVLSIINFLTGLLYLKENIGKIKFFDLGQTYITVLMNLVSVSRQLMVYQKSYTEVSRDFVTKVHLFNWKDDSEYAMEIHILVHKISHFFVMYIHGLMFIGLSMFNLTPLYNNYSNDAFTKRLHGNATLEHAVYYSLPFDYTTQIPGYIVVFTYNWFISLVCSINFCSVDTYMSLLVFHLWGHLKILIHNLEHLPKPSGLKSAANINGATQTERYNEDETQQVSERLRDLIKHHCLIRNFISIMSSAFGYVLFVYLGFHQVCGCILLLECSSLEPSALTRYGVLTVIIFQQLIQLSLIFELLGAMTEKLMNAVYNLPWECMEERNRRMVCLMLRQSQLPLRYKALNMIEVGSATMVTILKASISYFVMLQTFATKD

>LstiPR5

MDLRYMKQLRNFLHLLDCWPHRLLGEDVKPFPLRSIRVLVTEWIIILVGGVIFLRANINKRDFIELGQTYLTIFLTAFGIQRVTISLSKSYQELMNDFVLEIHLFHHRQKSKYSEYMYQHIHKICTVLVSLMYAEAIISSVLFNVTPLYKNYKKGMFSQERPSDKRFELSVYYSLPFVNQETNLFAYIVVSIFNVTLTFDCGLIYCGLDANLAIIVFHIWGHLKILDNTLRSIPTPVEMRNHIPRFDDKLSYTKEENEKVAAMLKYIIHHHRLIMGFMTKTSSAFGPTLCLYLLFHQISGCFLLLECSTMDAESLGRYAALTVIFFQLLIQICVIVELLGTQSETLKDAVYSIPWESMDTSNRK

>LstiOR1

MLVKKFKAFYNKEGFDYSKGYIDPRDFHLTFFFVQRAFQVIDEPFQPWTYVSKTITVICGIGVLTDACFSFYHAIDIFDMGMITEAGTYVLMLMYKMMTLTITKINLSSYIHLIKCMKEDFAYICTKNDKYRKAFFETHMATWQLCVKVCMFMFWLATSLVLFAIGSLFFYLATHEPGDGTHRPLVFPFWAPGIDYTTSPAYDIAFNFANIGVIACTYNYTFVLQTNIVWVRQIASKAEMIGMCISDLLEGIQPANNEEEKRHYARMINFRMREIVSQHQKMYKLLDSYAAVYKKCLMFEQFVSSPVICMLAYCSAEVCFFSGFMSLLYLKIEKRGNALTIN

>LstiOR2

MTLIKAIKGIFFKDSFDFSEPDIDLYTFHPQLRIFIAPLGIFFNNRKSLLRFLWPFINGSLSIVAIVLEMIFVYHGITVGDYSFATECFCYFVMLSVIPVLYCAVLANSQSVMVLLDKMDKDFAYICKLGAKYRDHFLQRQLLIWQLCWIWLGFLCCVAVLYTLMTLAPLTYQTLIATQDENMIRPLLFPMWLPEDDPYRTPNYEIFLFLQLDYLLIFIQSFGVYVYIQFHVLLHNFTVLELVTFDFDVIFEGLDKSVVDLPRDDLRRLTVQRVFNRRLKRVATWHDSVFKSIGTLSRVQGPVIVYQVMFSSLCICLMMYQVADKLDKGTFDILFIMLTVAGITQLWIPCYLGTLLRNKAFDVADACWNCGWHETPLGRMIRPDIIIVIMRAQHPISIKFTGLPNLSLETFSSIMSSAYSYFNMIRQSNN

>LstiOR3

MFKNFSLRFEDPEKPLYGPNFWILKKMGLILPDNRTGKALYILMHEIVAFFVFTQYIELYIIRSNLDLVLTNLRISMLSIICIVKANTFVFWQTKWKDVIDYLTEADRFERESNDPQRKIIIDKYTNYSRRVTYNYWILVFITFLTTTGSPFIHFASAVYRESIRNGTEIFPHIFSSWVPIDKYHPPGNYITVVWHISVCAYGAMIMASYDTSIMVIMVFFGGKLDVLRERCKQMLGTGEVALSDSEVAARVRELHDTHVLIMKYLRLFNSMLSPVMFIYVVMCSLMLCASAYQLTSAQNTAQKLLMAEYLIFGIAQLFIFCWHSNDVLVKSDNVMLGPYESEWWAVNVRQRKNILLLAGQLRISKVFTAGPFTDLTLSTFITILKGAYSYYTLLRD

>LstiOR4

MSLAGRSVSAHLTFLRLCGFCRLGRDGSSPLARRAHAFYCSFALAVTTVYLMQECVYAYQERNDMDKLARVMFLLLCHVTSITKQLVFYLDADRIDEMISGLDDPLYNQPVSWQKSLLTETAVSARRLLRVYSGTAVITCTLWIIFPILYYSQGLPVEFPFWTNLDHRKPVFFVILLMYSYYVTTLVGIANTTMDAFMGTVLYQCKTQLRILRMNLENLTQRASVIVKKDPNEIFDKVLERLFLECLVHYKQISE

>LstiOR5

MVSTSNAPADFFDFNLKYLFYVGLWPREDWPPTLNWLYRIYEVTLMLFAFAFLTSTGIGMYMSKDDVITFLTNMDKAIVAYNFTIKIVIFFFKRKHIRVLISEILHSGDKIDKSRQKLMMIHVVAISGMITTIIGSFQTFAQMKGEMVVDAWLPFDPRKNMWTVFIAGQILGVLFVVPVIYRAIAIQGIVCSIIMYMCDQLIELQGRLKALTYSVENESYMREEFKDIIRKHIRLMGYSKSLKSEFKEYFLVQNLAVTTELCLNALMVTIVGLEQKNHLISFMAFLIVALFNAYIFCHLGNELMDQSAGIANAAYESTWTSWPIDMQKDLLIIITVAQKSFKLSAGGLANMSMQTFAEALYNGYSIFAVLRDVVD

>LstiOR6

MKILPKGIIKKCTQTVGVNNEIDEIMRLTLFFTRIFGHHILDPNWTWNITFPYQLATLLLISYVIVGTLEIIRGTNDVKLIAEAAYTFIVIVVMQTRFYFFLSTRKHFQHLYIQMKTTLYNSILDDPEKNLKDVLKKLRMVVNWMAFFCFFPVVIYILTTLWCYFNGEKRLMSKTTSILMPMRTPYYEIGLLLHSIFMFSAAFTIGEVEIWFVMMMIFFRTACDGTEKYLSVEARHENESQHDYAIRLKNSLRKFYKSHVKEIEFLNTLNAMFKWLGMMPLISVALCICLILLLLSKGIDMTFVSNVIPVIVELFVYNWFGEEIKIKAEKWKSAILEFDWLNLLPKDKKCYCILVCYMSKEFGIKIATGTFLSLLTMSTSLKFSYQAFTVLQTMDI

>LstiOR7

MAIFRKDIPASKMNHQNFTFDKIFIITAKAMILNRSHPSIPRNWFWVFQFLVILTLSATTFLFLINSVLFYDIPARRYAEASKNSTMAIVAFTVTIKYLFMLYFQKYMQDLIDVVDRDFKLALDFEEEEKEIVIMYAKKGSKASWYWLLAASSTSSLFPLKALLKMGYSYWKGEFELIPMFDMRFPDRVDIVKEIPAVFAFYFVLCFMFSCYAGSMYIGFDPLVPIFLLHISGQLNILSKQIMRIFTENNSVDEINEKLKHVNIKLQDLYWLIENIKSKFTVLFEYNMKTTTFLLPLALFQVVEDLKRSQLNLEFISFFIATILHFYMPCFYSDNLLDQSNYLREAIYSCGWEKHSDTRARKTILLMMTRTTKPLVLSTVFYPICLDTFAEMCRQAYAIFNIMSAACA

>LstiOR8

MEVYHESGLGGTENAEDIYFNPFEKTFKFVMFCMVVGMIYPYPKMTRMWQIAAICFYLIIPNPSSSIVVYDVIEAYKEGDMDCIFRHIIVMGPFITHYLKMVLMYIYRSNAKLLLEEMNEYFEKLNSKPLSHKLIAKKWLTKSFFLEKSWAYCVLAGSFSFPIMAICKNIYSALFDEYPRRYCIQELRSPFSGSNLDSPFYEIMFINTCMASCMYYINFNGYDGFFVQLILHTALRIAVCGESLKDAFNIDDSVLRRRAVCMVIKEHIAICNFINRINVLFQHWMSIITSYMVIHFCVCVFFLSKRSGLQESQFLCAAFASVMYLFMICAVGGLIQDESEKLSDLFYECGWERISDPDCRRLLVFMIARAQKPLQVQTIAMYNVNLQLFVKVLKMAYSLFTFLQQT

>LstiOR9

MENQFKPFHETYKAVMYSMVFAMVYPNPATDKWRLLAIPLLLLTMLPMSVIALLDSLRCWNEANYLEVLRHIAMFGPFLCGILKMCFMYHRRVEAKAIIDTINEDYASYNYFPENYQTFIRAYIENTKIYHRVWYFCVLLILSAFVMTTTLYNTYEYMFRSEPKRHMIYDIRLPNKAAGVELETPYFEILYMYMLYVALIFYLNFTGYDCFMITAVNHACLRIELFCKHLDDAMEFKGEELRRRMRTAISEQCETFKLIDACQSTFNGNLGMVYLAVTTELCINLFLMTEGYEFDYKFTAFSIGTILHVFVPCRIAEKMKNVCEESSTMIYCCGWEEMYDLSVRRYIPFMLARAQYPATLKAFGILTYDMMLFASTVKTAYSLYTILKRQQT

>LstiOR10

MSEATLAEAKREIAESLTLNTFCMRRIGLSFEEPKNASSYLAQKFMLVLSVMSICYHVFSEIVYIGLTLSNSPRVEDVVPLFHTFGYGALSIAKVFVLWYKKDVFKQLIHELAGIWPMPPLDDDATAIKLKSLTALRIAHQWYFAVNVLGVWFYNLTPIIVYTYRLWQGQEVQMGYVWVSWYPFDKYKPFAHVVVYIFEIFAGQTCVWIMVGTDLLFSGMASHIGLLLRLLQRRLETLATQAQTEEDDYREILNNIKLHQRLITYCNDLEVAFSLSNLVNIVLSSVNICCVVFVIVLLEPFVAISNKLFLGSALIQIGMLCWYADDILHANADVAAAAYNSGWYRTSARCRRALLFLIQRAQKPIAFTAMGFTDISLVTYSSILTRSYSYFALLYTMYNDK

>LstiOR11

DLAITTKVCASIEINFTTMEGIYPEEFINSVLKSLSYFKKCNIDVFDSKNSLYRKFWWLFNIPSFILNYITLTMYIVKIFTEGVDPFEKIYMIPVWLVTTQEFFVCIIIIQKEKEIRTVIEHLGSIWRTKDLTEYQSNHKKTTMKQLNFGQKIFEIMSLIVAWLYMLMPLAETLFRKFILDQEAELMLPYASVYPFAVDSWATYLGVLAFQIYNMLFVIFMYLGSNLLLVSLSTGLSIQFDLLRADLINIKPTNNRENIVFEINDENVKWSACNIEEFVKFHQDVILLTQELNAVFDKIVFLSL

>LstiOR12

MTSLDKLADCLPFLVSIIIVVYFGLYRKEMYDLTKFMQREFKYRSAHGLTNMTMLNSYKTARNFGYFYTACTMFSVTMYVIPEIINRWNRQPLQSYIYMDVVRSPFFEFTFLRQCVAQMFVGLAMGQFGVFFASNAILLCGQLDLVCCSLRNARYTALLRCGIKHSVLAAAHGDIQGDELYNYIYNAAELQPSRYHYDQKMNHNILNTKTSFDIYSREFDEATCEALRDCARVSDVINAYKAKFERFASPLLVVRVVQVTMYLCMLLYAATLNLDMVTVEYLVAVMLDTFVYCFYGNQIIIQADRVSTAAYQSAWHTMGVRPRRLLLNILLANRRPVAVRAGYFLPMDLHTFLVIIKTSFSYYTLLVNVNEK

>LstiOR13

YTFRKVTRTIVDGYLVCDALTLKGERFTKNLLKTLKDVKKRALIFWVVIIGNGVIYFVKPILLPGRHLMEDQFILLGLEPTFESPNYEIGFVLMSCGVICTVYLPANITAFLIVLTGYTEATMLALGEELINLWADAQRFYRNNHVEIDITDENALVNLTDDAEKNRIMNRYIKQRLEEIIKIHTRNINLITQIEKVFRGAIAVEFLLLITGLIAELLGGLENTYIEMPFALMQVGMDCLTGQRMMDACVKFEESVYDCKWENFNVSNMKTVLLMLQNSQKTMVLSAGGMATLSFS

>LstiOR14

MINFKSLWTRLTHTKALEKSSGKLETRFFETVYRVSYLTGISAADDDIPYMIYSSTVKLLIVLLVCGEIWYAFTETSSLDEIAASINTTVIQFITMYRYRNMIRHKDVYKKLAMSMESPFFDISTQERRNLVDYWVKKNERYLKLLLFLGNCTLAAWFLYPLVDDLEYNMFIGIRLPFQYRSLIRYTFAYLVVVMAFAYISHFVMVNDLIMQAHLLHLVCQFAVLSDCFENILADCEKKFKGADRERLIANKRFREAYRVRLGDMVNQHQSILSHVMDLRRTLSGPMLGQLAASGTLICFIGYQLTTTGADNVTKCLMSLFFLGYNLFEFYIICRWCEEITVQSQKIGEAAYCSNWECGLADIPGVKSCLVLVIARANKPLVLTAGGMYNLSLLSYTSLVKTSYSALTVLLRFRQN

>LstiOR15

MLSQKLISFLERLEDQSHPLLGPNVKCLYIFGLWQTVKTRKRNFIYNIFHFTTFLFVMTQFFDLYKQLDDFNKALNNLSMTFIGVISCAKCYSYVLCQRQWQKLAADISAEELAAMEDGDETVMIKMKEYKLYSRVITYLFWVLVTMTDTALIVTPLIKYLTTPMYRADIREGIEEYPQIMSCWFPFDYMSMPGYMFSTMIQIIMSIQGSGVIAASDANAITIMTFMKGQMQILRQKCIKIFESNSYEPKEILKRIKECHRHHTFLIQRSEDFDKLLSPVMFIYVLICSMAICCSVVQFFSSGATAAQKLWVIQYTSAQIAQLFLFCWHGNEVFVESKDVDQGVYESDWWKADVRLRKQVLLLAGKLNRPILYTAGPFSRLTIPTFISIIKGSYSFFTLFAQMQEET

>LstiOR16

MGGSFQSHYSFCFLIMLRRFVSSLEHEDNPLLSPTLWGLQKWGMWQPKNGPSPKISNAIHFAAILFVISQYVELWLIRSDLNLALRNLSVTMLSTVCVVKASTFVAWQKYWRNVIENVSKLEKRQLSKKDKMTNTIIDEYTKYSRRVTYFYWTLVTATVLTVIVAPLVGFLSSAETRQRIRNGYDSYPEIMSSWVPFDRSRGLGYWVSVLEHILICFYGGGIVATYDSNAVVLMTFFAGQLKLLSVNCSRLFEGEEEMTYEEEIKKIRECHYHHLLLIKYSKILNGLLSPVMFLYVIICSLMICASAIQITTDGTTTMQRIWIAEYLLALIAQLFLYCWHSNEVLVMSNEVDEGVYSSAWWSRSIRVRRCVLLLAGQLRRSVVFTAGPFTEMTLPTFVAILKASYSYYTLLVNKDD

>LstiOR17

PLESFFDKMTHIFLTRQKKALTLLGHWFPTKKYRIPYLIYRCFLLFIQWSFLLFNIIYMRQVWGDLEETSEGSYLLFTHATLSLKSTIFLMSKNRWSNILNFMESDIFAAQTSVHEKILSVDAMKMWSVYAFFITSATFNCIEWAVVPLLDNHGERVFPFKIWMPADPTKSPEYHIGYVYQVMAIYINAATFLTMDYLTASLIMFAATQLGIIEEKIKQIPATPLSATLEEKNKLIKQNNEILNECIQHHQAVIRFVRLVENMFNVNVFFQMSGTVAIICVISFRMTIEPPNSIHFFSNDY

>LstiOR18

MTTTKADTKNRVYSHTDYDDSYKIITKNILSLVGIRIAQKDSSFARLCWNILYWAEFANLFIALVLDGYTACDVIRSSALKEENIVFMMLPCMGYLVIALLKSYKTVYQRGIYENLVSELRSMWPQGSVTEEEHIIIDKALKELNIVVKGYYWCNLGLCFSVIGPPYVDLVRRAFGEDVPRTLPYFYWVPYDEFQPVAYELTLALHAWQTMLTLWFMQAGDLLFCAFLSHITTQFDLLCLRIQRLFHVPVDQQLIAEYPLGKQSKKPSDNETFSPLNESETRSKQEKELKKIIVRHNDLIRLSNDVENLFSFALLINFFNSSIVICFCGFCCVMVEKWNVFMYKTFLATSLSQTWLLCWHGQKLLESSARVADALYNSGWYIASNPIKKSILIMIHRSQKNVCVTTYGFSVISLSSYTTIIKTAWSYFTLLLNTYNQ

>LstiOR19

MDETLKVFHRVLSFAGIAIYAKEKWNSNLWLSFQIFNFLIGTFCFIFTTGFVVSNYSDLLIFIQAACIWTTGVIMTMSLGVCLIFRQKFRMFLCEMVFKDEVLEMPLIRYVLRLESGKKLFELKQMVKDSQEQLFRVTGVLLKCYVTSVWLVATLYLCSPIYEMFSKGDKSLRLLAFDMWFPWSLENLKVYIASFIFHAYAGYLCCVAYPGLQLTIILLIGQVIRQLKILTFIMLNLNDLVLEITKEKDIRWQTYCTAVLSQCVDHYIKLKSFSNRLNVICRPFYLTLILVAIMLVCMCSVKIAVSDKLSPDTIKYYVHEFCFILVVLMFCLLGQQVDNECEQLERAITENWYIFDKKHKTHVKIFKMALTQRMHIFIFGTITLSLPTFTWFIKTGMSFFTLVMSVLEEGNYE

>LstiOR20

MTNTRTIDKIGIPTLNFLNFLEDPRYPSVGPHLRLLGLTGLWHPNLKSRITRFKQYLFFVTIAFFLSQYVKCLIKFDPIYLKLVLQYAPFHLGIVKSCFFQKDHKKWETLIDYISAVEREEISDGKRQSNEIISEYIKRGRKVTYFFWGLAVVSNISIFTEPYQKNQINVNGTSVYLCVFDGYTPFGEVPPGYYASMFIQTVLGHIVSAYVVGWDTLVCTIMIFFAGQLKISRMNCTNVIDTGNADINHKNIVKCHNFHTTLVMNQKLFNSLISRPMFVYLIVISVNLGVCIIEIVQQQNDLTTLISSCVFVVACLIQLLLFYWHSNVVSQESTVVSYGTFESNWVGLDQRTQKEVYLLGLTTSTRLVFKAGPFNEMSLTTFVAILRLSYSLYTLLDNTM

>LstiOR21

YTRRSCYGSYVFLPGIGEVVYLLKRRENIGDVAEGLYLFLSEMYTYFKVAVFWLNKDKVINLLGYLSCEEFKPVEAEHREIIRKSIKAARFVMTYYSTMCVGAVSVGIIMPLTENFDILPTNVEYPHFDVYKSPAYETLYIHHIYYKPATCIIDGVMDTILAAFVASAIGQIEILAFNLRHFDLVAERRRKRAVAGNKPSATWTKERHIRAVLKECIVHHNSIIKYVSMIESAFSLASALQFMLSVMVLCLVGIQFLSIENPSRHPMQILWMAIYLTCMLIEVFILCWFGDELIWKSTELRQAAFDGPWLETNHKTMVFIVIFLERCKRPLRVTAGKIFTLSLDTYTVLINWSYKAFAVVSNMKK

>LstiOR22

SFIRYSSFFLSQSYRDNVKEGNEDYLQVVSSWVPFNKSTIQGYLAASIWQSYASIYGGGWITSFDSNAMVIMVFFRAELEMLKIDCANIFGTEWNPVSDKVAFARLKDCHRRHVELVKYSRLFDACLSPIMLLYMFVCSVMLCVTAYQITSETSAMQRFLTTEYLVFGVAQLFIYCWHSNDVYFASLRLSEGPYESTWWCRHVSHRKNLFILTAQFSRVVVFSAGPFTKLTVATFISILKGAYSYYTLLSKSQTK

>LstiOR23

MAESFKVKTSRMDIIRHKVLVWAGIYKLHTKKYYLGVCHDVYRVFVIVMLILVNIQHIIYIYLRAIRGEDVPWDIILLVITMIDLIIKVVTINIHSKQIDEIHDLIKAPMFDPTCTEDETILKKTEKQINMLLKIVYIDVTVLNITWDIYKIGQRMTNKSAAIESYFPFNTNPWPGYLLAFLYEWWIIIIWLGYGLLSLDCSIAIYYTRAATQLKIVNYHLEHMFDNVKVQSQKRFQYKDLVDRSLNLKFIHFVQRYQNIHRLINTVNIAFSEGTAFQFFSATVGIGFCLYKMTYTELFSVEFQLAFGLVLIYQMQNFMYCYFGNKVESESDRVCTSMYFSDWPSASPRFRRQMLIAMARWARPITPRVSIVPVTMATFEATVRLSYTLYTVLKSRSMTMN

>LstiOR24

EDDDFDEKTITEVNTVKIFNYEQTDEEKEIVKESIKFLNFVVRLQYYICGVVIFAFPLMPVTSMAYDYYMTGTTEYKYPYLVKYFFDVYNMKMWPAVYFHHVGSTAIVGAAVFGSDSLFYTVCIYIQMHFRTLCLRCERIVTSSAKETRENLAKAVKRHQELIDLVDQVEILYSKSTLFNIVTSSFLICLSGFIITVLEDISVVVTFATFLFMNLSQISLLCYFGDMLMRSSTEVSSAVYNSLWYETDERTKKSMLVILMRAQKPCKLTACNFADLNLTAFTTILSRSWSYFALLKTMYK

>LstiOR25

MDSNKLSHSFYKITYVWKLLGMWSGKSSSKNLRIYSWLFVTLYYIMYNFFYTLSLVCAPRAVDTIGVGVYYFTTLCGLAEIVMILRNRQKIISVFETMDCKEFQGNNRQTNEYLRQFKITFSRYFKVYAAYCFISSHTFLMFLPLFNYFFQHKELEMPIWEYYFLTNATRNKYFFYLYTYQSMGMIATIFNHIVYNVFLFGILSVAVSQSKVLNWNIANIRLADEDILKSTEEKERLYLNKLYNCLKHYEIILKYCEDVQDLTSFLISLNYGLSVFTLCFSMYMFLLPTNSNTLVYMGFYLSAILIKNFVPSYLGSELTNESNNLRFAVYSCDWVPRSKNFKTCLMIFVERARRPLLIKGLKVVPLSLATFTSIVKTAYSFFTLLRGAQDQLI

>LstiOR26

MGIFKGFKRIFFKENFEFSSPDVDLDNFHPQLRRLIVPLGIFFNNQDSFLRFLNPVLNSLMIFIAVVMEMICVIHGIQTLDYSFFTECFCYLVMLLYVPVLYFSVLGNKDSMLEILHQMEGDFKFICNLGDKHRDHFLKRQLLIWQFFLIWMATISSVAFLIFLRTLIPLTYQSLIATHDEHTIRPSLFPMWLPKDDPYRTPNYEIFMFFQMYFVCVYVQSFGVNVYIQFHMLIHNYTILELVIIDFEMIFEDLDEDVVYLSRYHPRRVLIQRILNKRIQRIVAWHDSVFTSFDNLSSIQGPVICYQVLFTPIVYCLMMFQIADKLEGGQLDIYFAGLLSVFTFQLWMPCYIGSLLRNKGFDVGEACYNSSWNTTPLSRMIRNDIVIVMSRAQQPLSMQFFCLPDLSLETFSSIMSSAYSYFNMLRQYNR

>LstiOR27

MATFNSEDLFLNRAKFVMKFLGVWIPPVDESLPRKFLKLFMLMLQYLFLIFQTIHIVQIWGDLAAVSQPSYLLFTQACLCMKITVFHVNVDKLRELLKQMASDTFMPQSIVHEKILKAQAARIKKYLLAFMIGSQGVCSMWYLHPLFEGTGVRKFPFDMWMPVSPEDSPQYEIGYAFQLLTICMSAYMYFGVDSVALSLVIFACAQIEIIKDKLLSIAPVQYGLKEKERKIMNEKNHKILVECIVQHQAVVTFTQLVEDTYHLYLLFQLTGDVGVTCMCALRILVEEVRSVPFASIFLYVIVMLIQLFICCWSGHELTATSEDLHTVLHKCSWYEQDLKFKRDLRFAMMRMNRPLVLRAGHYISMSRQTFVAVLRMSYSYFAVLNQANKKDQ

>LstiOR28

MWENLRKFGLGHCDLPTMVWNVAFMLRGFTLNIDSRFTGRIPKIFYITTIIIAFCYLYSYFFSMLWFVFWRCIETGDVTAAMIVFPLGITSEIGIAKFIYTCVYRKKVRQLLQQYLEYDSQIPQGSRLSRHLLQALRNVKRRALIYWIFIVSNGTLYILQPLVMPGRVPMEEVFVLYGLEPELETPNYEITYVLCTFGSVCTCYLTSNVAAFLIIVSGYVESQLLALSEEILNVWDDAELEYKVIDNADEEEFENKEKYDAINESVKTRLKDIVKGHTTNINLLLQVEDIYRGTFKYARALHWTRVEIETAGFEVGKANYENSWLLLWKMQSKNVGKENVNKILYVFISFSNALDYSALLSPISY

>LstiOR29

MHLFPSKKIHKGLRFAMNVIILLFMMSEWAAFLTQSNLTEKQATDRLMFGFSHPTLFSYVLAVEYHQERITNMLHKLAMVLKEEYNDKDIERKMVKKAMSNAAGFACLFMMALIFYGYDGIMQVIRGDGTFTTVITFWPDVTEVSYAASITRVATYFIWCVFMTRVCAVYCLVIPTTVCLSHQFKNLQSYFYSLEDIFDEDLEQTELEKKYEEAFKVGIKMHSKTLECTKDYQTAYNVIVSNQVLTTVGVLVLLMSQMVDSERTLENVLSIVTTGSAMLISTGFFMWNAGDVTVEAAELATAVYCSGWAHCQHSAPRVRRLLVITMMQAQKPVVIWALGIVELSYQSYVSIVKSSYSVFSVLY

>LstiOR30

LLLTMKNYEILKKYCKKMFLVGSGNFWYESGIVGDDSSWYYKIYSRSLFSIYGFMTILEIMAAIFGDFPGDEKRDSVTFAVSHTIVMLKIFSVVSNKGLVKTMNQNMVKICEAYEEPTLMAAKYRIVRINVLAYFSVVYGSAACFVCEGIRKLNAGSHFVTVVTYYPSFEDDSIFGITFRIFTTVILFLMMMTMIVSVDSFTMAYLIMYKYKFITLRHYFEGLTEEFHKMNSVNPRLAADKLTNGLVEGIIMHKEILRMAKDIDQAFGTVIALQLLQSSGSAVSLLLQIALSDQLTFVASMKIIFFVVALFFLLGLFLCNAGEITYQASLLSDAIFFLRLARQQMAAASAAQSQATGAAGGYAVAAAARHEGLQDD

>LstiOR31

TCRLGKMSLAGSSVAPHLALLRRVGFCHLQGAAGGGSARSGPKRLHSYYCLFAFGVTSGYVLQQAIYAFQERSDLDKLSRVLFVMLCHCTCVAKQAVFHADARRIDRIITGLNQTLFNQPIESHRSQLRGTALSAARLLRVYYSTAVATCVLWIIFPVVYKLRGHRIEFPFWTFVDYNQPVMFVVVLFHSFYATNLVAVGNTTMDAFIATVLFQCKTQLRILRMNFETLP

>LstiOR32

MLYNGMGTCSLLRHVLLLQPKRKSVWLLLHSNIAAKMAGPTTYSTFSAVTPHFNALARVGYFKMVMKNPSPTQLMLHNCYRWLIWFSILSYNIQQLIRVIQTRHSTDEMVDTLFILLTTFNTLGKHVAFNARVWRIDRIIKVINGSIFAAKNPKHVDIMKLNEKAMARLLYFFQGMVLTGCVMHATYPMINRALGQDEISSCFSSESSGSLSTQIATWYLSISLTIQAYGNSTMDCTISGFYAMAKVQLQVLRYNLEHLVDSEDEQEDIDTNDVNIGKLRYKDNTVIQSRLVHCVKHQLQIKWFVKEVESIFCEAMTVQFLIMAFVICMTVYKIVGLTVSSAEFWMVFVYLNCMLAQLFIYCYFGTQVKYESEFVAQSAYCGAWTRLSPAFRRQLGILMQCARPIVPCAAKIVPVSLETYIAVLRASYTLFTILDKQ

>LstiOR33

MKWLVKNTFTALRISLTCLTITGFYTRRREVNFVLSYCFPFLTFMFMTGISIMAQFVDLIIIWGDVALMTGTSFLLLTNVVLGLKVFNVVWKREKIRAVIEESDGQLQAVDTNWGKDILKSCERQSTIFFYIYMFFPYLTIMGWATGHEKGELPTRAWYPYDTTTSPGYEITSLHQVVAVCIGAAVNVSVDTVVTALLAQCCCRLKLLSACLKMLGDGMLTNNQGMFKQDQEVAIKANIRSCIQQHQAVLEAADLLQEHFSSPILAQFTVSMVIICVTAYQLAFESSNTIALLAMACYFTCMTLQVFLYCYQGHELSVESGNVGAAVYESPWYKMSLPLRRDLLMLMMRSQRLAKLTAGGFTTLSLNTFMAIIKTSYTLFTVLQQTED

>LstiOR34

MMVKKIFSFVRRSPLQETRDLKVNDLVAMITKRIQNAGLNYRDENLKVHWLAIASIICFIITYGLQVVALVNAKDDIDRLFECLSVMSFCGMGILKLLSLYRNHKHWKMLLNKITELEKEQVINEGTSNEEYESDNEDDTTYFPDYIATYTKQFQTLSDILSRIYGSTAIIYILSPYAEFALLKFTGSDVSSYPHILPGWTPFDSSFFGYLATIAIELVSAIYCVCVHVAFDLTSIGIMIFICGQFSLISDYSKNIGGNGAMCFLSKRRDDRAHGRIIRCHKIHVQLINTCDELSKLLQNILGVYFSVATLTLCSVAVRLNSELSSMELVSLLQYMCATLTQLYLFCHFGDNVLHQSAVGMGQGPFGAAYWCLSPRIRKELAILGMGMMIPRSFKAGPFISVDLPSFIQVVRTAYSYFAVIRK

>LstiOR35

MIVKNVNTSVSISLTTLRLVGFWVPEHFEGNKKLLYDCYGIFSFMFLLGTYLIIQTVDMYMIWGDLPLMTGVAFVLFTNLAQTTKIVFMVRRRRQVHAIIKEADRELRAVDSNEARAIVKSCNKETIFLQVVFNCLTLVTMVGWATSAEKNKLPLRAWYPYDITRSPAYELTYMHQIGALCVAAFLNVCKDTLVTSLIAQCRCRLRLLGLSLRTLCKDLRTTEQNHLSADQEDIVRARLAKCVKQHQSALEAALQIQRSFSEQTFAQFNVSLVIICVTAFQLVSQTGNLVRLMSMGTYLLNMMFQVFLYCYQGNQLSEESAQIAGSAYECPWYLMSTPLRRSLLIVMTRTRRIAKITAGGFTTLTLASFMAIIKASYSLFTLLQQVEEKD

>LstiOR36

MDIPAFEDLFKEIKINLWLFGIPFNCSRIRLRFYLMLIAIVLMIIGESCFLVSRYSPENLLELTQLAPCLCQGLLSALKILPIAAKKEKIFELTKCLDRLYSTILMDAHKKAVVQREMTLVKILMKYFFILNAILISVYNFSTLLFMFYSYVVRSEVEFMLPYAVIVPFSTETWVTWFIVYIYSISCGFICVLYFTTVDALYCVLTSHICKNFAIISNEIQGINASNVGNLKDLVKNHQYVLKLSEDLEEIFRLPNLFNVLVGSLEICALGFNLTMGSITEKPKSFLFLSSVLLQILFMSVFGENLIRESRKIGDAAFCSKWYDIDMSSKKTILIIMTRCRKQQQLTAYKFSVISYGSFTKIISTSWSYFTILKTVYKPPE

>LstiOR37

MDLFNYIKRVFRDAKSRLQENSYESLLSLVNFVPSVAGFSIRGNTIFVPFWILHLSLLFYIYGVGCAVYQIKYAEDARDFIKSFVNVSLIVLIANNSHWFLQKRSLLKTALKEISESDVMATANESFRQKHERSVQKIKRILFIFYGFNLLNATFVYLPHRADVLNSYSMTPCFGMEPLTSSPNREICMTLLCIQEITIMVVVLNYQALLLLLIAYTALMYTLLADEIMTLNNFDRETYYNNPTVKLILPDLVKRHAILLSIIDKLKALYSGSIGVNFGSNAVCISLFFYLPLQEWLQFMPVLVYCFLVFFLYCFLCQRLTNAAELFEMSVYACGWENFELKEKKAIYFMLRQAQKPVEILAADIIPVNISTFATTLQAMFKFVTVVKV

>LstiOR38

MNLKMNFLKRYTEEDLVNIEEHNFGPFHKVYQWLTFTLTLGLLFPNPASERFRLTFIIVLLVTIQPLAVMIFIDMYKCWQERDIFNIIRHSTIIGPFLGAFFKMFLMYWKRVQTKSIVDQINSDHEAFNHLPRKQQDIAFLYIKAGVRNVERIWAPLVSVAIMMFPGMAVILTLYSYTFNDIPKKYMIHELNPPFSTDPEDMSRSPYFEVLFVYETGAAIICVLNYTAYDGLFGVATNHACMKMSLCCVKLNDAFACEDQEEMYKGVLAFIEEQQKMYKFVDLIQEIFNIWLFAILMSTMIQIGSLLFHISAGYGFDLRYTLFSFTSVVHIFLPCKHAATLKSMSTEMATMIYISGWERSRDRRVLRMIPFMLARA

>LstiOR39

MNEQLSYKSLAPHVKYLRAAGLFRLSPDSPKRHIFFHTIYLRWLIAFFSVYTVQQILKIYEVRDDVNKVMDTMFLFITNTDCIYKAAVLQKKPEKIEELLNTMKGPIFNLGVPEHRPILLATVRKALLLVHMFNRLSLITCFLWALHPTIMHMQGNPIEFAVWLPFDANQDPQFYIAVVYVWIQTSWLAYSNTTMDIFIAFLLEQCRTQVSILRLDLESVVQKSKEEAARTSSPYSEILERRFGRILIHHNEIVNSADKIQDIFGGAVFYQFVIGGWILCCSAYRIVNTQPASVEFVSMLMYTTCIVVEIFVYCFFGNELFYESNKLMDSAYAVDWLEIPVKQRRSLIIFMERVKRPICPTAGSMIPLSNSTFVSILRSAYSCYAFLRNSEH

>LstiOR40

MENQNPQERSAPIQYVRGSRAFRQFKNPPQPHMCIQDTIKDTTEKLFINVLGWQKIANPKQYNDPIPLYGGMQVPQGCGPNSNRPPLLVFAVMVNPDILKANGKNAANPNSNRPPLLVFAVMVNPDILKANGKNAANPTDREALVSLLCDFVEAMNPGLLLARSPVILKDRDLAGELKDVWLAVQNKREREKGLSQDVMYKVYDIDGIGNEDSNEEDKMNSKNCKQNDCDSLNRSSNDKKNVIKSSKQILMNAGQKSEFDSGMNNCQINQNPSREYRCSTTKADTTYCTPVYGQIVSSRENHNQINDIQQKFSSSETTKPSSSFRKDWNPVHGKTTEGWDEFSKRNVSSISNEGDVKKQRYIKNEKGNKEMSNGKSQYDFFPVFDNKAVDEVSDSNEKSGTPDKSKEQELGNEDSSKIILDAVQKLVLQPTDNKICDNKTSALSSISS

>LstiOR41

MPEKSYGTVKSNLREELNYINSMGSKIFLYPFSGRSKLVDICYLFVCFLVVVTATQLLTALLVTDLKEWIEIVNVAPNLGVVLMTLLKYTKVHNNQHVYKKIFKHFSDDLWDVVFDSYDHKKIVIRYTAIAKYATRFLFYYSVPLVVFVDSFPRIIMYLENEIIGNENPQYLYPFDGWYPFDKVNWYYTAYLWESFMTFIVVCVYAFSNMIHASYTSFICMELEILGVSIKDLITPDDVTNITNHLKVQEIHSHIKRKLKTIIRRHQFLAQLASELNIVLGDMMLLNYIFGSVFITLTIFTATVVDNMYKSLRYFFMFCSLIVEIFFNCMIGQVLSNHSEQLTDAIYSADWPFADNETKVMLLILMRRTQKPFEYTANGYLAMNLNSFSGVCSMSYQLFNLIRTAYSK

>LstiOR42

MNTTLGTPVKTNKSAGFFLKVCQLCYLFGFPNCWMESLKFSKTFTKIYDPFSKLTNVTIYLFILAEWGSMFTQNNLTEKQRSDRIMFCLSHPVLCSYRVILAYHREKLQELMYNLCLVLKEKVNDEEIEKGMVRKALAYTSALIGLCSTSLFLYGADGFNQMMRSEATFTTVITAWPLVEDTSISASAARFFLYFMWWVFMSRVFGAFAMLISLIVALEHQYKNLGKYFRNLSGIFEQDLSQAQKEKEYDQSVKYGIKLHAKTLRCTRLAQDSFSSIFGAQILLNTYVLVLLMFQMVSSERTLANVLAVIATGIAMLLSTGFLMWTGGDITVEAAILPTDMYCSGWHNCRSATGTRKLLALAMLQGQKPVMIKGLGFITISYPAYLSIVKSAYSVFSVLY

>LstiOR43

MWENLHKFGLSLEYCNLSTMLWNVGFLLRPLTLNVDSRHKDRIPISSYVFTITIASCYFYVYLFNMLWFVFIKCRATGDLITAMLVLSLGISSEIGPCKLFSMLFYKETIRTIVEGYLICDAQTLKSDRFSRNLLKTLRDVKKRVLIFWVVIIGNGLFYIIKPIVLPGRHLTEDLLIIYGLEPMYETPNYQIAFFMMCCGTTCCCYLPANIGAFLIILVGYTEATMLALSEELLNLWTDAQSYYTNNHEEIETTVDSAMVTPNDAEANRIMNTYIKQSLENIVKIHTKNIGLIQQVEHVFRGAIAVEFVLVICAIISELLGGLENTYLEMPLTFMIVGMDCLIGQKMMDACDTFESAVYDCKWENFNVANMKTVLMMLQNSQKTMVLSAGGMATLSFSCLMSVLQSTYSAYTTLRSTM

>LstiOR44

MIEESPFDKSLQKIQFAFRSTGLNLGTDGRKRNFKQNCVYLFNFLWLNTDIIGALSWLLEGIISGKNFTELTYVAPCLTLSILGDIKAFCLLLNERKVHNLIDNLRNLEAKSKNFENSEWDNIMQPEIKLFNIIIKVLNVLNCLMIVVFDVSPLILIAVKYFTTGELELLLPFLDVYPFDSFNLRYWPFAYIHQIWSECIVLLEICATDYFFFACCTHIKIQFKLLQHQFQEIISAKSVSAMDSEDPIVVRAKFQELVKWHQEIISCANKLEKIYSFSTLLNFCTSSLVICLTGFNVTTIDDKAFVITFIIFLSMSLLQVFFLCFFGDILMRSSMDVTDAVYNSRWYLSDVATGRNVLLVQTRAQTPCKLTAAGFADVNLNAYMTILSTAWSYFALLQTIYGSRS

>LstiOR45

YFFFTCCTHIRIQFKLLQHQFQEIIANRSISAVVSMNQMSIRAQFKDLIKWHQNIISCANMLEEIYSKSTLFNFLASSLMICLTGFNVTTVDDKAIVVTFIIFLSMSMMQVYFLCFFGDLLMCSSAAVADAVYNSRFYLGDVVMGKIVLLVQTRAQKPCKLTAAGFADVNLKAYMRILSTSWSYFALLQTIYSSRC

>LstiOR46

HLQENIKTLQQETKMKFVIKNTFKASQISLTYLGFTGFWTRRSEVNFVLNYCYCFVTFMFMTGISIMAQFVDLIIIWGDVALMTGTAFLLLTNVVLALKVLNMVCRREEIRAIVEETDGQLQAVNTDWGKEIVKSCDRHLTVLISIYTCLSYLTIMGWATGHEEGELPTRAWYPYDTTTSPGYEITSWQQVVGVCLGAGVNISLDTVVVSLMAQCC

>LstiOR47

FDPSQSWFIYCLVYPFEMYCMFRFIYAYLGAEFIMEALCSHLVTEFRLLREDLMLIKPVPNKRSSEGIDEIGEFVKKHQKLTLLSKQLDDIYNKVNFIVLLFATVIIGFFAFAVKVSHGYKMLVNSLAVYGMLLPVFIMCYYSQLLAVESAGIAVSAYNSPWYKGGTHHQKSIYFIIKRAQLPCYLTSLKYSPITLKTFSKVLSTTWSYFSLVTRVYEHGNEG

>LstiOR48

VTPYNCIANISGGKINMQLLTMQLLWCSIHFVSLIVMVEPCHITQREMGRTNFLVSQLMLQNTDELVTNELNVFGRYLYLNDVVYSPMGICVLSRSLVASILASVTTYLVIMMQFQATENIVYHG

>HarmOrco

MMTKVKAQGLVSDLMPNIKLMQMAGHFLFNYHSENAGMSNLLRKIYASTHAILIFIHYACMGINMAKYSDEVNELTANTITVLFFAHTIIKLAFFALNSKSFYRTLAVWNQSNSHPLFTESDARYHQIALTKMRRLLYFICGMTVLSVISWVTLTFFGESVRMVTNKETNETLTEVVPRLPLKAWYPFNAMSGTMYIVAFAFQVYWLLFSMAIANLMDVMFCSWLIFACEQLQHLKAIMKPLMELSASLDTYRPNTAELFRASSTEKSEKIPDTVDMDIRGIYSTQQDFGMTLRGAGGRLQNFGQQNPNPNGLTPKQEMLARSAIKYWVERHKHVVRLVASIGDTYGTALLFHMLVSTITLTLLAYQATKINGINVYAFSTIGYLSYTLGQVFHFCIFGNRLIEESSSVMEAAYSCQWYDGSEEAKTFVQIVCQQCQKAMSISGAKFFTVSLDLFASVLGAVVTYFMVLVQLK

>HarmOR6

MSFRKFLFENEAVDGINGPADYLYIKILRFTLDVIRSWPRKELGEPESASFTVFMKYFYLVLTIATVVGSILYVVVHVTELSFLEAGLMYLIILMSFLDALTVMSLTFSEKYRVLAKDFLTKIHLFYYKDRSKAAMEIHKKVHLISHLFSLWLLFQMLSGLSLFNLTPMYSNLAAGKYRRGGLGNTTFEHSLYYLYPFNTSTDVIGYIVACILHWIISYLCSTWFCMFNLFISLMVFNLWGHFKILIITLEEFPRPKSIGSLPSAYKYSQEELVEVAEKLKDCINYHRVIKNFTNRMSDVFGPMLFVYYSFHQASGCLLLLECSQMTAQALMRYLPLTIILTQQLIQLSVIFELVGSESEKLKDAVYSVPWECMDTKNRKMVRFFLMNVQEPIHVKAMGIANVGVTTMAAILKTSMSYFTFLRSM

>HarmOR11

MHLAGNAVTGITGPMDYKYMKVLRFVLRIISGWPGKALGEKTLRIEGMGHAYYNTILSLVYLALGIAYLKKNFHRFDFLELGQLYIVLLMNMLSTSRAFTLCLSQKYREVAKIFIQKIHLFYFKEKSDFAMKIHITVHKISFISAVYLSVLLFIAACMFNLIPMYNNYSAGRFASFDNLENTTYEQAISCLYPWNFETNFNGYLAATLSGWYGTILCGSSVSMFDLFLCLMIFNLWGHFKILIHNLEHFPRPASEVVDAEGEERSGRTVGSEMYSQSELEEVAVLLRDCIQYHMLIYNFTNNMSDAFGMALFIYYSFHQITGCLLLLECSQMTAAALTRYLPLTIIMFGELVLLSIIFETIGTMSEKLKDAVYKVPWEYMDTKNRRTVLIFLIKVQEPIHVKAGGLVDVGVTTMASILKTSFSYFAFLRTF

>HarmOR13

MKILSDGSDLEGVEKVEDIFYINLARKSMWILDSWPKTPNESVTYRYFVLALNVATLVGGAVYLRNNTGVLSSFELGHTYITVFMNCITCSRCIMILSREYNEVMLSFVNKIHLFHHRHKSEYAYKTHIFIHKISHFYTVYLLGLALNGLLLFNMIPFYNCYSRGMFRDVIPANATYDHSVFYSVPFDYTTKFKGYIAMTSFNCFISYTCTSYFCVVDLTVSLVIFHLWGHMRLLTYHLANFKKPASVLESNENTDAIKDHSYTQEELKEVFGKLREYIRHHNLILNFSSEMSNAFGPALLAYMVFHQVSGCILLLECSQLDMKTLVRYGPLTVVIFQQLIQISVIFELLGSSNDKLIDAVYLVPWEYMDTKNRKLVFVMLRQSQRSIDLKMMSMLTVGVQTMTAILKTSFSYFVMLKTVAEEEQ

>HarmOR14

MGGIRDFIFNLEAKEGITKPTDYPYMILCRHLLTVITCWPKEPKEGLDTRAKLKARIWVTFQKIFHLNGCFITTIGMAMYIALHKNSMSFFELGHLYISLLMTVVIFSRVTTLCWNPEYQAVATDFLTKIHLFYYKDDSDFSMQTHKQVHKISHLFTLLLTGQMVAGMSLFNLTPMYNNFSTGKYKKGGLKNSTFEHSLYFSYPFNASSDVRGYILSNIFHWIISYLCSTWFCTLDLFLSIMVFHVWGHFKILIHDLNHFPRSLNTISFRLDQSNITLTTEMYSSRELVQVSERLNKCVEYHRRIVSFTDKMSEVFGPMLFVYYGFHQTSGCLLLLECSQMTVEALVRYLPLTIILFQQLIQLSIIFELVGSVSDKLKDAVYGLPWEDMDTKNRKTVAFFLMNVQEPVHVKALGLADVGVTSMTAILKTSMSYFTFLRSK

>HarmOR14.2

MAGLLDFFFNYEANEAITTPKNYPYLIIMRISLSLIKCWPKKTTENLAAGAKMKAKVWGMVQNVLHLAFCVLTIVGTATYVMIHKKNMTFFELGHLYITLMLSCVVFSRLATLTFNEEYQVVANEFLNKIHLFYYKDNSEFSMQTHKQIHRVSHLFTLYVTGQMLGGLSLFNLTPMYNNYSAGKYSKGGLKNSTFDHSLYYSYPFDVSTDVRGYIFSNILHWFFSYIVSTWICTLDLFLSVIVFHIWGHFKILLHDIDNFPKPSKMVSFKLENTNVTISNENYSTEELEQLADKLKKCIDYHREIISFKNKISEVFGPMLLAYYGFHQASGCLLLLECSQMTPEALARYLPLTLILFQQLIQLPIVFELVGTVSSKLNDAVYGLPWEDMDVKNRKTVAFFLLNVQEPVHVKALGLADVGVTSMTKILKTSMSFFTFLRSM

>HarmOR15

MTGFCDFIFNYQPKDGITTPTDYPYMIIARHLLTVITMWPKTSVILQSAKTQKRAKIWLTIQKAFHFWLCVTSFFGGTLYILRHKKSMTFYELGHLYISLLMIVCTFSRITTLCLNDEYRVIAKDFVTKIHLFFYKDRSDYSMETHKKVHMISHIFTLYLSGQMMLGLFLFNVTPIYNNYSAGKYTSGGLKNSTYEHALYFSWPFNASTDFRGYVVSNILHWLLSFSCSSWFCVVDFFLSLMVFHVWGHFKILLHDLDHFPRPANKISFILEDSYVTITDEIYSRNELNQVFDRLNKCIDYHRDIVSFTDKMSEVFGPMLLAYYGFHQASGCLLLLECSQMTVAALVRYLPLTIILFQQLIQLSIIFELVGSVSDKLKDAVYGLPWEAMDTKNRRIVAFFLMNVQEPVHVKALGLADVGVTSMTAILKTSMSYFAFLRSK

>HarmOR16

MGLRQFLFENEAVEGINSASDYLYIKVLRFMLLIVNSWPRKEMGEPESPKFSAFVKYFYLVVTVLASAGFILYLVKHNSELTFLETGHMYIVLLMSFNDVSRVATLTMSTTYREVARDFLTKIHLFYYKDRSKQAMETHRAVHKIAHLFTLWLVSQMLSGLSLFNLIPMYSNYAAGRFSGEVSKNSTFEHSMYYPYPFDTSTDIRGYSIACITHWIISYLCATWFCMFDLFLSLMVFHLWGHFKILNYTLNDFPRPSSEVEAAKYSDEELVEVAARLKDCILYHREIILFTDRMSNVFGPMLFLYYMFHQASGCLLLLECSQMTAQALIRYGPLTIILTQQLIQLSVIFELVGSESDKLKHAVYGVPWECMDVKNRRSVVIFLANTQEPVHVKAMGVANVGVTSMAAILKTSMSYFTFLRSL

>HarmOR1

MTSILRKYGFKRKSKQRFNEKVYTKKDYDESYAPTKKVLGWVAIRMTHSISENATKWWDRFYWFEMFNLFLTGPSEMVSMVSTAYEAKTFLDSIKVFRMMPCFGCVVLSMFKSINMVIHRPVFENLTNELRAMWPQGEVSEEEHEIISGALKQLNIIVKGYYWCNNALLISFLSPPYFLTLARYFGHDSPMGLHFLYWLPFDPYQPVYYEITLVLQTWHACVVIYFNVAWDMLFCLFLCHITTQFDLLARRVRRLFYVTVDQQLVRSYPMALVSEEMLRVEGERVRSQGDNYWQTRHHAEITQIVLRHHALIRLTGDVERMFSLALLINFMNSSIIICFCGFCCVLIEEWNEVAYKSFLVTALTQTWLLCWYGQKLIDSSKRLADALYDCGWYNASKKARSAVLIMLHRAQKGIYVTTHGFSVISLASYSTIIKTAWSYFTLLLNFFKDKSANL

>HarmOR3

MTLSVLDRFYLIDDGFFSFNLKYLFFVGLWPEKTLTRNQKILYKMYEHFISFLTTTFIVLAGIGTYQHKDDLVVVFCNIDKCLVVYNFFFKTIIFFIKRNQLRDLIDEIEMSGDEVTEERKKLMANYVMFITGVTAAVIGAFSLLALFEGTMSIEAWLPFDPMESLMNQILSLEILAFCVFPGLCRAFAMQGLVCSMIMYLCDQLIHLQKELRDLTYLKETEMVMRTKFKNAIRKHIRLMGYSGRMENIFKEYFLVQNLAVTVELCLNAVMMTVVGVQQITLLITFLAYLMLALVNAYIYCYLGNELIIQSQGIALAAYESAWTSWPVDLQKDLLIVILAAQRPLKLSAGGMALLCIQTFSQALYNGYSIFAVLNDAVN

>HarmOR4

MMLPMMSADCMPITHLITMTYKFITLCHHFERIRTEFDEDTKIMNRREAIDKLRAGCLEGIRMHQKLLCLADEIHRVFGIIMSLQVCESSAVAVLLLLRLALSPHLDLTNAFMTYTFVCSLFLLLALNLWNAGEVTYQASLLSNAMFQ

>HarmOR7

MKIKMSKPLIFDQSIEKLGVLFRFSGMNIKNKIVTPLDTIKYRWLYTLNFLVVFSAIIGSVYYVILGIKQGKNFIEVTSVAPCLTFSILSMIKSLYHLMYEEHIQELIDLLTELEIRENNREKCIEKEEIIANETGFLNKVINVLYVLNCSMIVVFDMTPMVMIAVKFYKTNEFEMLLPYLDVFSFIPYELKYWPFAYIHQIWSECVVLLDMAAADYLFFTCCTYIRVQFKLLQYDFERMIPDRSISKGVFYEENELRNKFTELLKWHQDIIYSSTILEIIYSKSTLFNFLSSSLVICLTGFNVTIVDDIVIIITFLTFLSMALMQVFFLCFFADLMMTASLEITNSVYNCKWYSANIKVGKQILFVQTRAQEPCKLTAAGFADVNLNAFMRVLSSAWSYFALLQTVYGAK

>HarmOR8

YMILFAMNFSFLPKQLDIFVEDLLFYFTDCAAISGILTIVFMREKVCELLEMLESDIFQPDDVEGLAIVEGAGKFIKLYWNIFASVSFTSSAVHLSPIIVHFVIGTELKLPVCSYRFLSENFGQMFVVPLYLYQGSGNMFHMMYNVSIDTFFAGLMVLTIAQLDVLDKKLRRVTDKDEHEDADGETFRQRHDKHREAVRKINQCIIHYEEINKFRRLVQDVFSISLFVQFGMGSCIICICLMRFTMPAPLSYFFFLATYMFLMVIQIMVPCWFGQRIIDKSNFLAFSAYNCEWTSETRQFKSNMRFFVERANKPLSITGGKMFRLSLVTFTSIMNSAYSFFTLLQNVKSRK

>HarmOR9

MLEQFDRCLKSVNLYLKFLGLHLESKDTNRTFVERSRSHRLYFAHLFSLNLEVVAQVLWVLEAVITGKSFVEITRLIPCLILCLISNFKTLSLLYYGRHNNEFIVTMRSLLLNQMQVEEKEHRFRKNLIDKHVLILTSISKKISYVIVLGLLMFALAPAFIIIPHYFKTDEVKLEMPFIAYYPFNEFDLRIYPWVYFHQVYSAVIAMIMVYGPDCFFFTCCTFIHIQFSLLNNDMERIVTEET

>HarmOR10

LWAMIPLFDAASKRSFPFRIWMPVTPLKSPDYELGYLYQMVSIYISAFLFISVDSVAVSMIMFGCAQLEIIMDKIQKIKYVFESADSEEGRRNIIKTNNEFLVECIKQHQTVERFIQLCEDTYHANIFFQLTGTVAIICNIGLRISIVEPNSVQFFSMLNYMVTMLSQLFLYCWCGHELTIRSENLREWLYQCPWYEQDTKFKRALFIAMERMKKPIIFKA

>HarmOR12

MDEEPLLIDKTVKKIEFLFRCTGINIKSGTKTRKDMIKSRTVYIINFLWLNIDLAGAVMWFFTGIANSKSFTELTYVAPCITLSFLGNLKSLFLILREKHVDKLIQVLRDLEINEKARPKSEETDAIIKYEHNFVTTVISVLNVLYFVLLVAFALSPVSLVALKYFTTNELELLLPFLIVYPFDPYDIRYWPWVYLRQIWSEVVVIIDICTADYLFYTFCTYIRMQFRLLKHYIERVIPEDDGGGRLTNIEDVRAEFVLLIKWHQDLISSANMLETVYTRSTLFNFVSSSVLICLTGFNVMAISDVAFVATFLSFLFMSLLQIFFLCFFGDLLMTSSTEISEAVYNCRWYLADTSLGKDLLLVQTRAQTPCKLTASDFSEVNLKAFMKILSTAWSYFALLQTLYGAPT

>HarmOR17

MSLRSDSECARSVAPHVRVLRRVGFLRGAALSSRPRAQRLVLRGYHALALAATSLYVLQQAVYAYQERGDMDKLSQVMFLMLCHVTCVVKQIAFHVDADRIDRLIASLDEPLLNQCAGERGALLRGTARGAARLLRTYAGCAVATCVLWIVFPVINRIQGISFEFPFWTGFSYDHNAVFTLVLLQSFYCTNLVAIGNTSMDAFMATILDQCKTQLRILRINFESLPERARALHVESEENYDTILDKLFIDCLVHYNKITEMCTELHDVFAVPLLVQFGVGGWILCMAAYKIVSLDVLSIEFASITLFITCILIELFIFCYYGNEVTVESERVSQSLYSMEWRRARLTFRRSLVLVMERAKRPLRPAAGRVIPLSLDTFVKILKSSYSFYAVLRQTK

>HarmOR18

MEMKVDVLPEKKYKGFNETFKLCAFSLAFAFLYPNRTTALRRCITITLIVTFCGGQLFWFITYTFKCLYTLDIYNFARNMTLAVVLVLFFIKTYYVIYATSKFAPLLDKISDDLLEANNLEEEFQVLYDDHIKIAKVGEISWLLIPTIMSALFPIYAGALMTIESIQTDDYERRMVHDMELLFVEDIQSETPFFQCMFAYNCVQCVVLVPNYCGFDGSFCIATTHLRLKLKLMTLKVNKAFKYSKSRQELRMRLYDSIKDHQDALDFYVQLQNVYGPWLFAVFLLTSFMISFNLYQIYLLQRIDPKYTSFGVVGVLHIYLPCRYASDLTRVSEEIPDDLYLAQWEAWADPSITKLLMFMITRAQKEMIVTGMGLVVFNMEMFKSILQTSYSFFTLITA

>HarmOR19

MLIYLKLIGTSLELTSYFQSVLINTKMKDRDILRNYCKIMFYIGSGNCWYKEDEIGNDRSLLYRAFGASLIFLYGAMTILEIMAFTIGNFPVEEKRECLSFASSHVVVMLKIFLFIFNKPLIKGLNHKIVSICEDYDDSALMAKKYKTMKRNVISYFAIVYGTTLFYITGGLRNMFRGSHFVTVVTYYPSFDDNSPLANFVRVLNTIILSMMMLTMIISLDSCIVMYLIMYRYKFMTLRKYFENLREEFFALINRQEVEMATEKMANGLVERNKNAIAA

>HarmOR20

MDDELEFKPFHETYRLITFSLCIAMIYPNPRTEKWRLFSIPILIATVAPVAIMIFLDMYKCWKNGDIVNIIRHSTVVGPFLGGFFKMILMYHKRVQAKQILDEFDRDHLMFNTVAETYKDIARASIRNCQIYSERLWACLVTTCVMTFPVMAIVLNIYNFMFKSEPTKYMIHDLEKPFSTEPEERFESPYFELLFVYMFYAAILYVVNFTGYDGFFGLCVNHACLKMELYCKALEEAMMADREEVYGRVIAVIREQCRMFRYVDLIQDTFNIWLGIIFIATMIQICTCLYHITEGYGFDIRYMIFVYGAVVHIYLPCRYAAKLKAMSMETSNRFYCSGWERVDDERVRKMIVFMIARAQVPNEITAFNMMAFDMELFLSILQTSYSMFTLLRS

>HarmOR21

MENYSGYYKSSKTTEFLINLNKFVFIFGLPNFWIEKLDFTDTFRKVVGQLNKYGNWSIFGLILAEYGAYFTQKNLNERQSSDLILFMICHTLTTAFRVSVSHKDVQIRNVVYKLGIALKEEFNDSQAEDQMIRRSKFFSWALILNCVLSLLMYTVEAVMRVIRAGATYTTVITAYPDVDDRSTLSHVVRVIAYIIWCIYLTRIFAVYSLVISLTIAMSYQFKNLTSYFCNLSKIFEDERMTQTEKEQEYERAFRVGIKIHSETLKCTEDIQAICRDVFSGQIIFNILMLIVLMHQMVNSARNLTNAVTLVMAALTILLSPGFFMWNAGDITVEAQLLPPAMFSSGWENCGRDSSVRVRKLIVIAMMQAQEPVVLTGFGIIALSYQFYVSIVKSSYSVFSVLY

>HarmOR21.2

PFKENAMTKFLDELNTIFFLVGLTDLWISEVKFSKRFIQIYKKINYVMDFLCLFFVVFLLGSYFTQKDLTEKLANDRLMFSIILPGNLVFYYISVYYKEEIRNLLYHHRVLKEQHNDTRLEREMIRNIRVFSITLNSIAFVVDTSYGFGALYEVVTKGENFNTIVPVWPDVHDNSSLAGAMRVFFYFCWLNPIATRVLTTFSLLLTEMVAVCYQFRNLQSYFYSLDDIFSDDTLSQKEKEIKYEEGFKIGIRMHIMTLWCKKLHQHVNKEILAIEMVLFFAMLMSELTTLLGGERNASQLCMMFLISVSTCISLGFFMWNGGDITIEASKISEAMYSSGWQHCRGHSSVRMRKLVTFAIKQAQDPVVYKTLGVVDLSHTSYVTLVKMPYSAVSVLY

>HarmOR22

MNCFKNKNKVEVLMTPLVYTETDTTRMIEKYNKFWFLCCCPDFWVNKVDFSDTFTKIYRPAMMAIHVIMVIFCFSCTLSLWTQKNLSESQQSDRLAYGASAPIITIFYHFVILCYKDDVRKVLYKLVVVLKVDHNDKQAEREMMEQSRLHNGLFFSSCVCNMVFVGLYNCYLAVTTDATFITCISAWPDIEERSLPAGLTRVVVYFVWFAHVVRNMGVFLIIHTVLLCLTQQYKNLQSYFEDLNKIFDETKLSQEEKELKYEIKFKRGIEQHALTLWCVDETQRVFKITFSSHVLLWCGLLISILPDVMNNDDHTLKMLVSNAPRVAAALVGLGYFMWPAGDMSVEASNLPQAMYGSGWQCCYHRSSRVRKLVVLAMMQAQRQIEMKAFGHLTFSYETYVAIVKMSYSLFSVFY

>HarmOR23

MEETFLAFHRVLSFAGIPIFAKNNWNSKRWLLLQIINFIVGNLCFVFTTGFVATSYTDFLLCIQGACIWTTGVIMTISIWVCLIFRKKFRRFLEEMAFQDGMLGMPLIKHIFLVSSKGERINELRELVNDSQNKLFKYTRVLLKTYVLSVFVCATLYLCTPIYLMIVREDKSLRLLAFDMWFPWSLENYTVYIVSFIFHAYAGYLCCIAYPGLQSTIILLIGQVIRQTRILTFILLHLNELVLEVTGVQDEEWQTYCTLVHSQCVDHYVKIKRFSNRLNVICRPFYLTLILVAIMLVCMCSVKIAISDKLSLDTMKYYVHEFCFILMVLMFCLLGQQVENECEALEKAATEKWYIFKKTHKVNVLIFNMALSQRMPIYIFGTITLSLPTFTWFIKTGMSFFTLVMSVLEEGEY

>HarmOR24

VPAACALPSSNARTHHGLPATGVRLRLPGTARYGQVSPSDVLLLCHITSIAKQLVFHLKAERIDEMLAGLEDPLYNQPEEAHRRLLGATAASASRFVRAYSGCAVVTCTLWITFPVMYRLQGLPVEFPFWITVDYNRPTMFILVLAYSYYVTTLVGIANTTMDAFMATVLNQCKTQLRLLRMNFECLPERAAALSRQLGGSYDAALFALFRECLVHYEKITETAKMLQNIFGTAILIQFGIGGWILCMAAYKIVSLNMLSVEFASMALFISCILTELFLYCYYGNEVTDESERVSQSLYSMEWRRARLTFRRSLVLVMERAKRPLRPAAGRVIPLSLDTFVKIIKSSYTFYAVLRQTK

>HarmOR25

SIMPSDQSRMFDPPLTVLKIFGVWEGRTPSKYYKTFSFLFLFVSWFFYNFLLTLSLVYTPRSVELFLRELMFYFTEISITSKFLTVLLLRNKILEVFSVIDSDEFVGDYENKDGILYRTNKGYSLCWKVYNVLANIDYTCVIIMPVVIDLIQGTKSVLPICNYYFLSEDFRDSHFVILYLYQSIGMYGHMMYNLNMDSLAWGLLAVGIAQIKVLNKNFTDLKLSAEESKLPLEIQDNIQKTRLFKLLRHYEAILNYCDAIQNLLSVTFFFQFSFGALTTCVIMCSLLMPGTMVYRIFLVIYLFAMAGQIAVPGFFGTLLTHESQELVTAAYNCEWIERSQSFKRTLILFRERAGTPIIISGMKMFPLSLVTFVAIMKTTYSFFTLIRNA

>HarmOR26

MGENMKYSTFQGFRPHFDALARVGYFKIVLKPLSPQKRFLHNTYRFPSWTFILTYNLQHVIRVVQVRHSTNLIVDTLFILLTTLNSLGKQVAFNLRTQRIDKLINIINGPVFEATKPYHVEVLKENALVMSKLLTLYHGAIFTCGTMWTVFPIVNRALGEEVQFTGYFPFETSSTISFSLALAYMIILITFQAYGNVTMDCTIVAFYAQAKIQIQMLRHNLEKLVEFDDSAKINTQFNKTGLYSTSYKDEQQERVAIQERLKKCVQHYYQILRFAKEVESIFGEAMVVQFFVMAWVICMTMYKIVGLSIYSAEFVSMAVYLGCMLAQLFIYCYFGTQLKVESELVNQSIYCCDWMKLSPRFRRQLLVMMQCCGRPIAPRTAYVIPMSLDTYIAVLRSSYTLFTFLNR

>HarmOR27

MLSKIKNIIWCLGRQKIRNGEIDSVVTLLDRLILYNSGLASYTTTYKVHWTAHVLLTCFIIACVLQIIALFMGKDDPDRLFECFSVLSFCAMGMLKLLSLRKNHRKWRKLLTQITILENTQLSNRSISCVEYQSDSEDSDNFSEHISIYTKKFRGTSIVLTRIYSFTAFLFILSPFAERIICEIRGVECVGYPHVFPGWTPLDDFSIFGYLVTVLCEVFSAVYCVCVHMAFDLTVIGIMIFVCGQFSLLRDYSSRIGGKGRQCNLSMRRDERARFRIIRCHDINLLLVNSITELDMLLKNIIGVYFFVATLTLCSVAVRLKSEDMGVMQLVSLIQYMCGTLTQLFLFCRYGDAVLHESTMGMGEGPFAAASWCLSPRVRRDLSMLSAGMMSQRHLRAGPFSFIDLPSFIQVVRAAYSYYAVLGKKE

>HarmOR29

MGYQQIDCFDIHLKILRILGVWPHDNPSIYYIYFSRIFVFTFTVLYVVIYTMNFYFLPQQLEVFADELIFYFTNVGALSKALAFIFLRDKVKKMLFMLESEIFQSDDPEEIKLIKEGKEKSNFYWKITAGLSVSANTVNVCLPLLVHIIFSVELEFPVCRYSFIPEKYEAMFAYPAYFYQSIGITTHMLYNVNIDTFLLGVMFLAMTQLDILDRKLRKVTDVCINPDAARGSVDKFIDDQNAVLEIIKCIKHYDAICEYCKLIQDAFSEILFVLFSSGSCKICMCLFRFTMPATTGYFVFLYTYVTVMTLQVMVPCWFGSRLMDKSSQITIAAYDCDWTPRCRRFKSNLRLLVERANRPITIIGGKMFLLSLGTFTAIMNSSYSFFTLMRHMQSR

>HarmOR30

MLKELLQFMDAEVFKPDNEVHKNILKLQAARIKRLLLAFMVSSQITCGLWAMKPLFDDADRKFPFDMWMPVSPEKAVQYYIGYAFQLGTICISAYMYFGVDSVVFSSVIFGCAQIDIIKEKLMSITTVDRKQGTKEALAQNYNKLVDCIKHHQAIVTFTELVENAYHPYLLFQLVGSVGIICMSALRILVVDWRSMQFFSIFTYVSVMISQLFVCCWCGHELTATSEDLHTVLYKCIWYEQDVKFKRELCFAMMRISRPLVLRAGHYIILSRQTFVAILRMSYSYFAVLNQTT

>HarmOR31

MGSSGINAEYMGHFGKIHHIVKMNSILQNLEDPNRPFLGPNYWIIKNMGLLLPKNLLAKILYIILHEIVAFFVITQYMELYVIRTDLDLVLTNMKISMLSVVCIVKVHSFIFWQKHWHDVLDYVTAADKFERQSDDPIKSRIVETYTRYCRRLTYFYWVLVFTTFLTTTGTPLMRYLSSSTFRQNMRNGTEPFPHIFSSWMPIDKYHSPGCWITVLWHTLLCAYGAAIMAAYDTCIVVIMVFFGGKLDLLRERCKQMFGPSTISDRQCEEVVRQLHGIHVMMLKYSRLFNSLLSPVMFFYMVMCSLMLCASAYQLTSAQNAAQKLLMAEYLIFGIAQLFVFCWHSNDVLIKNENMTSGPFESNWFLANYRQRKDVLTLSGQLCIKNIFTAGPFANLTLPTFINILKGAYSYYTLLRK

>HarmOR32

MELDFNKIFIIPTTALRLARAHPYIPRDKKWILQFVTVHSLFTLTFLLILYNIFCHDLKANNFTQTCQNGVLFVVYIVISYQYGVLLTYQNTLVGLIADMNKDFQTSKDLPPKERDSIKKYINQGLWVCKQWLFLTISGCAIFLFKNLGLMLYYYCMNEFRLVPFYEVVLYPPIMEENRDNIFVYLLMYAIMLLFSAYSALMYAAFVPLGPIFILHACGQLVLVKLRIDDLFVECDDEVIRKKLKGIILHLQYVHSFVDRIQQVFKIGYELTLKFTALILPITIYAVLEGFYRGEVNVEFVTFIVGGVMISGSPCYYSDLLMEKGEDVRMSLYTCGWEQHYDRRTRTTLQLMLQNALKPIAIQTVFTVMCLDALTDLFQQSYAIFNLMNCMWN

>HarmOR33

ENLSNIIQIINAVDEMWRYIRKFGLEYCDLPTMLWNVSVLLKVLTVNIYGKNRKAIPLIFYIIVTVGLLTYFYVYVVSMIWFVFSRCPVTGDVLAALIVFSLGVASEISTVKFLYMRIHIKDVRKMVADCLDSYSKVVPGTRFSNNLLRTLREAKRRAMLFWMVIIGNGLMYVVKPLLLPGRHFMDDVVLLYGLEPMFETPNYQISFVLMGSSCVLICYLCANISAFLIIITGYVQAQMLALSEELTHLWEDAEENYRGNELEDITDDGDQNDRNKDAILNDYVTVHLKDIAKSHAENINLLGQIEGTFRGAIAIEFCLLVVALIAELLGGLQNTYMEVPFALMQVGMDCLIGQRVMDAGAVFEDAVYDCKWERFNKKNMKTAMVLLLNAQRPMTISA

>HarmOR34

MSGFQIVQLFKMSAKFSIMQIFKFLEDPAYPSVGPHLKLLGFTGLWHPNRHTLVGRFKHILFYITISFFFSQYIKCFINFNASSLKLILQYAPFHMGIVKSCFFQKDYKTWQQVIDYMSSVELAQLSKSNKEQYKIIYDYIKRNRKVSYFFWALAFFSNFSIFTEPYQKNQINVNGTSIYLNIFDGYTPFQKEPSGYYISMLIQTVLGHIVSAYVVGWDTLVVSIMIFFAGQLKITCLRCKMMIDVTNPMKSHLKIAECHRFHTTLVEYTRIFNALISPVMFVYLVVISVNLGVCIIQIVEIENDVPTLVSSILFVMACLIQLLLFYWYANEVTHESTFVSYSTFESNWPEANNKLQREVALLGLTTEKMLVFKAGPFNQMSLATFIAILRASYSFYTLLNSTN

>HarmOR35

MSYSDLPVDYRQTFGRYTYLFKCIGLDFFDNSTSYVSWNKMKLLLFCITFYPFISTQFSLLSEIRADNFLEGVRVLPVDIMFAHDIVKMILALVRRADLRVMILEVGELWSKNLTPDDQKAVILKAWLRKIRIPLDLYFMFAMMNLGLFELIPFLISCLYLLKGGKVYLFPFQLPKFWEVDSIITYLLTYVWEVVGTVPCQMCLYLPFDLIIVIMTSNVSALLRLLQVDLKNAIKLRDEQHKTKSNLNVSDTHSYEELKRIVDIHQRLLRIADQLSSIFGLVIFIHVACAALEICFFGFLTMVYGGLAETIANMLTVLNAVFTIFLLSLSGQFLCDTSSEVADAAYESYWYESDHKVKKLTLSIIIRAQRPSYLSALGFSQLTLKSFSKIMSSAWTYFSLLIQMYEET

>HarmOR36

LNLILISVYNFVSPVIIMYDYFVNNQLIFTLPYAVLVPFSTDAWIPWSAVYIYSIICGFVCVLFFTTVDGLYFVLTSHVCANFSVISDMIERLDENSVDRLPDIVK

>HarmOR39

MKCFMWLKQCKSRLKLNSFENLIWLLNLLPTLCGFPLFDVKFNYLFWTIHMILLFYIYIVGIVVYQMYSTQGFIDCINSFFNVSAFTLVFVDGWWIYTKRKEFNDLLEVVKKNDDLIIETGRFLHVHEKMLRSIKIIIIMCYIFHFVNEILIFIPFRTLRMDDFSIASCVGLEPVDTSPNHEVCMGLLAVQVVTSVVLVCSYDLSLLFLFSHTTAVFQILFEEMWSLNDIAQTCQNSDEDYDVIVARLRNVIVRHVLALQTVGKLENIFSVSIGICFGLDTISLCLFFVLPLEVCMHFAPMIYHSLFIFFLYCCQGQRLTTASEKFEMAVYCCGWENLRVKERKQVLLMLKRAQKPVIVYAAKVIPIRIYTLASTMQAIYKFVTIFKV

>HarmOR40

MVLKFLDRLEDPNNPLLGPTVLALRYGGLWQKDRVKHFLYNLVHFIAFLFVVSQYVELWIIRKNLEMAMRNLSLTMLSTVCVFKACNLMLWQNSWKELIDYVSELERSQLSKNDPVVNKIISDYVKYARRVTYLYWALVTATVVTVILAPLFIYLSSPNYQESIKNGSAPYPEIMSSWTPFDRSRGLGFCGATLYQMLACFYGGTVVANFDSTAVVIMTFFTGQLKVLSVNCERLFGDGNELVDYDEAVKRITECHLHHYYMVKFSSVLNSLLSPVLFLYVIICSLMICASAVQLTTEGTGNMQRIWIAEYLMALIAQLFLYCWHSNDVLYMSNKVDDGVYSSAWWSQNVRIRRSLLLLGGQLRRPIIFTAGPFTLLNMATFVAILKGSYSYYTLLAKKDD

>HarmOR42

GFVGLETIDPFVGEKEMDRFMFSLSFVITHNLTLIKLYIFFFKNVDIQEIVRTLEIELYDYYQNIEKNRKTVKISKIITGSFIFFGWLTIGNGNVYGTIQDLHWKSLVATLNDTSQIPVRTLPQPIYIPWNYQKDKSYIPTFVLETVGLLWTGHIVMTIDTFIASVILHMGAQFEILNEAITTAYDRTMTSLREGIRPEDSGHQGQSDRQSSILSVEDSNERIVHAFIPKEEIDAALQTTFRNCFRQHQVLINCVEKFSRTYSYGFMTQLLSSMAAICVVMVQVSQDASSFKSVRLITSVAFFFAMITQLGMQCFTGNELTLQAERISDAVMQCKWERIPSRQRRLLLIMMMRAQRPLRLTAAGFTNMDNACFLAIMKAAYSYYAVLSQRQE

>HarmOR43

MGIIRSIKYKLFAIKLQAVMGLMVKNENRSLQYCLTVLKVAGFLTPPRDGRIPRLTRRLYCFGVFMFLVGCIIMAQTGAMFEIWGDLALMTSASFLLFTNLAFATKIINVVVRSREIQEIIDEGDADLLAEDRYLGIEVIKSSNVETTMSMGLYTLLSGVTVFGWAASAEKNQLPLRAWYPYDTSKSPAYELTYIDQSSAVTLAALVNVCLDTLVTSLIAVCRCRLRLVALSLRTLCDGIPLPDKQLISPTEERIVLTRLSQYIIKHEAALKAARQIQRCFSLPILAQFAVSVVIICVTAYQLAMELNNRNWFRCIPMVAYLLCMALEVFLYCYQGNELLEESSEIAGAAYECPWYHCSVRMRRTLLIVMVRTRRALRLTAGGITTLSLACFTSIIKGSYTFFTVLQQAEDRNPK

>HarmOR44

MGYMIYSSSARMLSALVVICEIWHALGNNMSLDELISSVNVIFIHLITLWKLMIMVSNKKVFKKLARALESPSFDISTENRQAIVNHWVLTHKKYLKVLLCLAYLTLAVWVLHPLVDDMDFNLMVDVKLPFAYDSPLRYVISYLFVGTMFSYASSMVIMSEVIMQAHLIPLVCQFNVLANCFENVFEECASEFPDINKHELVKHNMFVEKYRKRLGDLVKQHREILDQTTDLKTILSAPMLGQLACSGLLICFVGYQATATIAENLGKFVMSLFYLGYNMFTLYIICRWCEEITIQSQRIGQSAYFSGWESGVSHVPGARATIILVIARSNKPLVFLAGGMYTLSLTSYTSLVKASYSALNILLTTKHE

>HarmOR45

MRLQILEDFLLKKTFDFDRPDINLYNFHPQLRILLAVKGVFFTNRRSLLRFIWPCICIQLSIVAMTLEEIFIWRGVTVKDYSFATECFCYWVILGCIPMVYVSIVVNTNKIYDIVVTMNEDFIYVCSLGDRYRKPFLEGQLLIWQLCYAWFIFVCFVGGLYVIIPLVGLLYQSLFATIDENTVRPLQFPMWLPNDDPYRTPNYEIFLVIESTLIFCFVQTFCVYIYTLLHILLHYYTIMNMIIIDFSVIFEGLDESVALLPRHDPRRRETQLILNARIAKIVRWHLSVFKAVSTVSSVYGPPLVYQVSFSSLAICLIAYQIAEKLDNGKVDILFCLLGIAACLQLWIPCHLGTMIRNKAFEVGDAGWTCGWHETPLGLMIRNDILIIILRAQKPVTIKFTGLPSVQLETFSSTMSSAYSYFNMLRQYSK

>HarmOR46

MSEAKPSTLNSEKTGRYIHYIEIPLKLVACWEFFPNSTSERLLVINDIYLGIVLFVLTYIPTCLTVHLYTEWQDIMSSLGTIADSLPLLVSLIIVAYYAMYRQDLYDLMEFMERNFKYHSARGLTNTTMEESCKIAQRFARIYTACTMFSVTMYATMPVIVHLWTKEPIQSWMYMDITRSPFYEFVFLVSCLAQMFVGLAMGQFGVFFASNSILICGQLDLLCCSLRNARYTALLQHGVKHASLRVSHATIQDDEKHNYIYNVSEMKESVYHYDKKVSNLYAEAKTQFDIYSSEFDDATVHALRDCASLCQVINKYKEMFENFVSPLLALRVVQVTLYLCTLLYAATLKFDMITVEYLAAVALDIFVYCYYGNQIILQADRVSTAAYQSMWQTMGVRPRRVLLNILLANRRPVIVKAGRFLPMDLHTFVVIIKTSFSYYTLLVNVNEK

>HarmOR47

MGAIRSSSDRIMPSDQSKMFDQTLTALKIFGVWKGRTPSKYYKYYTLVFLFFSWGFYNFLLTLNLVYTPRSLELFLRELMFYFTEMPVTSKFLTVLLLRDEILEIFNFIDCDEFVGDYENKDGMLYKTNMRYRLVWKLYFVLSHVAFTCDIILPIVFDVIRGDKSELPICKYYFLSDEDRDSHFMYLFLYQSIGMYGHMFYNLNVDSFAAGLLAVAIVQMKILNKKFRNLKLSAEERNLPFEIQDNIQIIRMNKLLRHYEIILNYCDAIQNVFSATFFFQFSFGAMTTCVIMCSLLMPATVEYRVFLVIYLLSMAGQILVPSLLGTLLTHESAELVTAAYNTEWIGRSESFKKSLMLFRQRAATPIKITGLKMFPLSLVSFIAVRISFYIKFSYPPTFSPFLRRFTFIVHLGVKGLHSTYNKSVYCFRS

>HarmOR48

MTKSNLKFEKLFKIATMSMRLNGSHPSIIRDTRWFIKFSIIMINTFCCCLFLIYSICCHDIKTGKFSEASKNGTMVIVSITITLKYMVLLYHQASIREIINIMEEDYRRAQDTSKEDLDIVVRYAERGQTVCKFWLVFGFGTSAIFPIKAFILMAYYTWKDKFVLVPLFDLTYPQPIEAYKNVTVVFWILFVVTFVFDVYASSMYVGFDPMLPIFMLHTCGQLDLLNLRISKLFVEAEDRAEIEEGLKKIICKLQDLYKLVDRVKKNYSILYEYNMKATTFLLPLTMFQIVEELRVKRINVEFISFFAASILHFFMPCYYSDLLMETSEKFRQAIYSCRWEKCYDKRIRQIVLFMMTRARIPMGITTVFYVINLDTFAEMCRQSYGIFNLMNAACE

>HarmOR49

ENLDERQTSDLILFIISHTIITGFRVKISHQEVQIRNVMYKLGIALKEVYNDSEAEDQMIKRSKFFSYALVLNCIMSVLMYTVAAVMRVIRAGLTFTTVITVYPTIEDRSVWSNVVRAIFYLIWCIYLTRVFAVYTLVICLTIAMSHQFKNITSYFYSLSNIFEDEQMTQTEKEQEYERSFRAGIQMHSETLNCTADIQRICRDVFSGQIIFNLTLLIVLMYQMVNSPRNLTNALTLVIAGLTILLSTGFFMWNAGDITVEAQLLPTAMFSSGWENCGRDSSVRVRKLIVIAMMQAQEPVVLTGLGIIALSYQSYVSIVKSSYSVFSVLY

>HarmOR50

TTYTRSKTTEFFYKMNFAIYIFGLPNFWIEDLKLSKRFVKIYDKISLFNDLLVYLLLVMEFGAFFTQHNLTDKQKFNLMVFAISHPLLCSFCVMVSKLKKKVRLVMYSQAVALKRDYNDPEVEKQMIARSLTYVLAFMSSCTITMIMFAIEAIWDVIRHGATFTTLITAYPDVQDRSILADVVRVLAFVTWWIFLTKMVAVYMLVIPLTISLRYQFKNLQSYFLSLAELFERSDLSQKEKEEKYEAGLKLGIKLHSETLSCAEDTQDVCRGVFSGQIIFNILLLIVLMAQMVTSERTFVNMFGTVATSCTVITSTGFFMWNAGDVTVEASYLPTAIYFSGWQHCQRDSSMRVRRLVVTCMSHAQQPVIFKGLGYIELSYQSFITIVKSSYSVFSVLY

>HarmOR51

MAGLLDFFFNYEADDAITTPKNYPYLIIMRISLSLIKCWPKKTTENLEAGAKMKAKVWGMIQNVLHLAFCVLTIVGTATYVMIHKKNMTFFELGHLYITLMLSCVVFSRLATLTFNDEYQVVANEFLNKIHLFYYKDNSEFSMQTHKHIHRVSHLFTLYVTGQMLGGLSLFNLTPMYNNYSAGKYSKGGLKNSTFDHSLYYSYPFNVSTDVRGYIFSNILHWFFSYIVSTWFCTLDLFLSVIVFHIWGHFKILLHDIDNFPKPSKMVSFKLENTNVTISNENYTTEELEQLAGKLKKCIDYHRVIISFTNKISEVFGPMLLAYYGFHQASGCLLLLECSQMTPEALARYLPLTLILFQQLIQLSIVFELVGTVSSKLNDAVYGLPWEDMDVKNRKTVAFFLLNVQEPVHVKALGLADVGVTSMTAILKTSMSFFTFLRSM

>HarmOR52

MRTLREIGQEIRKFGLEYCDLPTMFENVAILLRLLTLNIDIKYKGGITFYSYIITIVSGACYYYVFFFSMTWYVFWRSRELGEDIGAMIILSLGITSEIGPLKLFYMSYNKDKTQKIANDFLECDANTIKSTRFYANLLKHCRTVKKRAMLYWIVVAGNGVIYLLKPITMKGRNLPENYFLIFGLEPIFETPNYQIAYCMMVSALFFVCYVPACVTAFLIVVTGYAESQMLALSEEMIQLWPDAIKRAEERTQLDPSKVLDVYNLEVKTIMNQFVEKRLKEIIKRHALVINLLNQVEIVFRQAIAMGFVLLIVGLLAELLGKLENTFLQMPFAFMQVSIDCFAGQRVMDASLVFEKAVYDCRWENFDKANMKLVLVMLQSSQKTLALSAGGISTLSFTALMSIYRGLYSSYTALRSTVK

>HarmOR53

MSEKEFDKTLKLTNYALIMSGIKTSENDMNKALEYFINHYLFYCNAIALHTVIFGEVYWIVDGIRTNHPFVELSLVSPCATISILSTIKCGFIFSNKGILMRVVHKLKEIHTSFDDNELSKESSARTKIVTDSLKLLQFVQISFATIYIFVFFSFCFIPVILAEYNYYRTGEFVVTYPFFVKYPFDFDVHHCPVWQLIYFHQVWATAIVIMSMFGCDSLFYGLCVYIKTHFQLLGLRFENIVGATKSETQRNLAKAVVRHQELIDLVNQMEMLYSKSSLVNIITSSILICLSAFNITVVDKLNVILAFVTFLVMSLSQISLVCYFADLLMAASMEISGSVYRSPWYEADNHSKKILLLVIMRSQKACKLTAWKFADLNLGAFTTILSRSWSYFALLKTVYK

>HarmOR54

MPFLFTVLGNDRYLPTTPGETYGLSPKYETPFFEITFVLTCVATAFSAINQTGYIVLFVTLICHELGHFYAITEALHKIHTILTKEERSRNNSEENEQKSVDKLLIFCVKHHQFLMNFHGKIREMYKVIFGAHFLSMTVVLVTTLQTMNVWDYRNTILTGMSGIMPLFLYCFGGEKLISAGLQMSSAAYSCGWEMMEAKQAKVVLLMLCLVQRPLYLTAADIFIMNRETFGDVAQ

>HarmOR55

KMPGYVGGIIVHVIMGSQGSGVLAVYDMNSVAIMSYLKGQMIILRHKCISLFDDVTSTEDVLERIKECHRHHNAILKHFLLFNSLLSPTMFIYVLMCSITICGSVVQFASKEATASQKLWVIQYTCGLVSQLFLYCWHSNEVTLQSRVVDGGIYNSNWWKSDVRVRKQLILLAGKLNHPLVLDAGPYTTLSVPTFIEIMKGSYSFFTLFSQIQED

>HarmOR56

VTLSYDLKMKNMEVNYDKIFKVNIKAMTLNQCHPDIPRNKKWFFRFFLTHGIFSLVFLIIMYNIIFHDLANNDFSKTCQDGTLSVVYIVITFQYTIMVWKQDLLKDMIHIMRNDFEAIKVSNREDQEVVLKYAQNGAWVGKQWLLISISASSMFPLKTTVTFIYNYIVGEFELIPIYDLVYPPFIEENKDSLFVYFSLNVLLIYYAIYAGLMYTSFVPLGPTFMLHACGRLELLTKRVNRLFEDNKGDQIMVELRNICVELQLIYDLVDHIKDTFRVAYELTLKATTIILPITVYEVLESFGRGEFSLEFITFIFGGTLISSSPCYYSELLMTKGEEFRQAVYSCGWERLYDRRARSSIALILERALR

>HarmOR57

YKSAVKMENNFENSLKLFLIPMRVVGTHPEIPINLNWFILYSLTYGPFTILAIIIIYNSYLNATNDDFSEACKNGILSLTYFGASLNNIIMLWYRDSIKNLLDMMKNDYKMAAGLPRDEQLIFQEYVSKKTLVCKVWLILFTVSCSLFSVKAILLMVYYAIIGEPRLVHLYDLVYPDFIESRKENLSMYLVIYFFIFSYGVYAGFVFMSFLPLGPVLMLHACGHLEITKKRIETLFTSNTKDVNEKLNDIVKLLQYTYNFVETVKECFKVFYEATLKLSALALPVTFYALLDGLQHGEFSLEFSSFILSGIALSSAPCYYSDLLLEKGREVSLALYTCGWEQEYNRRARSTILLLLIRSSRPIAMQTMFATLCLIALTEMFQQAYTIFNLINSVQNSINEIKY

>HarmOR59

NYKTPFRYPLAYIVVMIAFFYISHFVMITDLKMQTHLLHLLCQFTVLVDCFQNLLRDCRIGFEDVAENNLVYEKRFADKYTKRLGDLVEQHKLILSNTMNLRDTLSSPMLGQLAASGILICFIGYQATTTIAESPFQGLMSAFFLGYNLFGFYIICRWGEEITNQSEKIGEAIYCSGWECGLAKLPGVRSTIMYVIARANKPLVLTAGGMYNLSLTSYTSLVKTSYSALTVLLQFRHE

>HarmOR60

MGVLVRNATMSVSISLTALQFVGFWAPEYLGKTQKQLYTCLSVFSFMFLLGTYLIIQVVDLFLIWGDIAMMTSTAFLLFTNMAQAAKIVNIVYRKERIQRIVNDCDAVLSRAQSLEEKEIVKSCNREMIVLQILYFSLTLITSLGWATSAEPHQLPLRAWYPYDTTKSPAYELTYVHQVGALLIAAYLNVAKDTLVAALIAQCRCRLRLLGYALRTLDKGMGNEAYTFTSEQEKTLNLRLGSCVMQHQKALDVGKELQECFSEPTFAQLTVSLIIICATAFQLSMGHSDNMVRLLSMGTYLLNMTFQVFIYCYQGNQLSEESSEIAGAAYECPWYKCSVRVRRGLLIVMVRTRRALRLTAGGFTTLSLASFMAIIKASYSLFTLLQQVIEE

>HarmOR61

MRMIVRIYFGAIAVPVTTYFLAAIWNYIRGLRVNYSKGFTTLMPSRSPYHEIGLMLHSTIASVLGFTYYIMDVWFFILISVYCVTNDSLVNILKLQRNEATEMEFMDQLHNALKTYYKNHVMLIEF

>HarmOR63

MEVLKHFPEDFSEALQTSFFILSFFNIRYLQDELSFIQKNWRFSYLVLIVIIHVVTLGLHLPELVTGDEMSQFAYLIPSILVTVHAIFKCFVLLPKTKEVSTFLRELGSLWRVKFTETQKQEKDKLLWRLHLVNRVSYWISIFGTSQYMLSPLAETLIRKFILRQESKLILPFDSVYPFNPTKNWLIYIVTYIFQFYSLFLLVTVYSGASLIMVTSCGLLTTEFVMLKDDLTRVKPRMKRGRNEVIKNDDDEPTIEQFVARHQKLLRLSRLLDNAFNGMVFIDLLFVGITVCAFGFMGQFTRSTMYKLLSYLGVVSSLLTVLYLCYYGEQLTSASATIGDMAYENLWYKGEKQYKMTIWLIIKIGQNPCRLTSLRYADVSLNMFTSVVSSTWSYFSLMNSVYSEEEV

>HarmOR65

MWKKFKAFYNKENYDFTKGYIDGYEFHKTFYQIMVAFKVADLSNPNPPSYFNQNLVIFISGFTAITFTCTSIVHGFESFDIPLITEAGTYTIVLFYELLIVSCTWRYLPQFHHLLRAIKDDFHYICTTGEKYRVQYFQTQLKTWKICIIMSIFTVAIPAGMITFATLSLVYFLVTYDPEVGGSRPLLFPFWMPNVDFGASPVYEMAFMFANVNVIIYAYNYIFMIQTQIVWIRQITSKVDIVNWSIQDLLVDIRPATSEEESMYYNYLIQTRMRDIVIHNQAMYRLMEDYAIVYKKLLMFEQKCCGPVVCLTSYCLASKFDEGEFQAILLLLCIATIVLHFIPSYLCTFLAFKVSSVCDACWSIPFWNAGPVIRTYMVLIMQRSLRPLPLQAAGFEDISIETFSKKMTNAYSMFNMLRQTNL

>HarmOR66

MEEFHNLPEEYRENIEHSLNLLTNGNILIFHKPNSFFEKRFRPYVVICSVVTYLASLTMYLGKVFRGELQLTELSYVVSVYMVSVQAILKAAIAYLNKEEIGSIILELGRLWRTQDLTEEQINKKNAQLKRLKFCYAVVYWINMIGSWQYILAPLLETVFRKFILHEECGLLLPFSCSFPFDPSGSWGRYIGVYIFETYSMFRIVYFYLGVEFLMISLCSNLATEFTLLQEDLQNVKPERNNIALKAIIANHQKLINLSQQLDNVFDKVIFVNLTSAAVPLCFFGFSAKVAHGVLP

>HarmOR67

MSESTIEQAKREIDESLVLSAFCMRRIGLSFQEPKTGSAYLRQKLMLIVSVCGICYHVFSEIVFIGLTLSNSPRVEDVVPLFHTFGYGALSIAKVFVLWYKKDVFRQLLNELAGIWPMPPLEEEAQTIKNKSLAALRITHRWYFFINVSGVWFYNLTPIVVYLFGLIQNKDDTIGYVWSSWYPFDKHQTLAHIAVYLFEIFAGQTCVWIMVSTDLLFSAMASHISILLRLLKRRLESVGTGDDDHYQEIVGNIKLHQRLITYCNDLENAFSLSNFVNIMLSSVNICCVVFVIVLLEPLMAISNKLFLGSALIQIGMLCWYADDIFHANADVAAAAYNSGWYRTNPRCRRALLFLIKRAQKPIAFTAMGFTNITLVTYSAILTRSYSYFALLYTMYSDD

>HassOrco

MMTKVKAQGLVSDLMPNIKLMQMAGHFLFNYHSENAGMSNLLRKIYASTHAILIVIHYACMGINMAKYSDEVNELTANTITVLFFAHTIIKLAFFALNSKSFYRTLAVWNQSNSHPLFTESDARYHQIALTKMRRLLYFICGMTVLSVISWVTLTFFGESVRMVTNKETNETLTEVVPRLPLKAWYPFNAMSGTMYIVAFAFQVYWLLFSMAIANLMDVMFCSWLIFACEQLQHLKAIMKPLMELSASLDTYRPNTAELFRASSTEKSEKIPDTVDMDIRGIYSTQQDFGMTLRGAGGRLQNFGQQNPNPNGLTPKQEMLARSAIKYWVERHKHVVRLVASIGDTYGTALLFHMLVSTITLTLLAYQATKINGINVYAFSTIGYLSYTLGQVFHFCIFGNRLIEESSSVMEAAYSCQWYDGSEEAKTFVQIVCQQCQKAMSISGAKFFTVSLDLFASVLGAVVTYFMVLVQLK

>HassOR6

MSFRKFLFENEGVDGIKGPSDYLYIKILRFTLGVVRSWPRKELGEPESAAFTVFIKYFYLILTIATVVGSILYVVVHVTELTFLEAGLMYLIILISILDAITVMSLTFSAKYRVLAKDFLTKIHLFYYKDRSKHAMEIHKKVHLISHLFSLWALFQMLSGLSLFNLTPMYSNLAAGKYRRGGLGNRTFEHSLYYLYPFNTSTDVFGYVAACILHWIISYLCSTWFCMFSLFISLMVFNIWGHFKILIITLEEFPRPKSIGTLQTAYKYSQEELVEVAERLKDCINYHREIKNFTDRMSDVFGRMLFVYYLFHQTSGCLLLLECSQMTAQALMRYLPLTIILTQQLIQLSVIFELVGSESEKLKDAVYGVPWEYMDTKNRRMVRFFLMNVQEPIHVKAMGIANVGVTTMAAILKTSMSYFTFLRSM

>HassOR11

MYAGNAVTGITGPMDYKYMKVLRFVLRIISGWPGKALGEKTLRIEGMGHAYYNTILSLVYLALGIAYLKKNFHRFDFLELGQLYIVLLMNMLSTSRAFTLCLSQKYRQVAKIFIKKIHLFYFKEKSDFAMKIHITVHKISFISAVYLSVLLFIAACMFNLIPMYNNYSAGRFASFDNLENTTYEQAISCLYPWNFETNFNGYLAATLSGWYGTILCGSSVSMFDLFLCLMIFNLWGHFKILIYNLEHFPRPASEVVDAEGEERSGRIVGSEMYSQSELEEVAVLLRDCIQYHMLIVDFTNNMSDAFGMALFIYYSFHQITGCLLLLECSQMTAAALTRYLPLTIIMFGELVLLSIIFETIGTMSEKLKDAVYKVPWECMDTKNRRTVLIFLIKVQEPIHVKAGGLVDVGVTTMASILKTSFSYFAFLRTF

>HassOR13

MKILSDGSDLEGVEKVEDIFYIRIARKSMWILDSWPRTPNESVIYRYFVLALNITTLVGGAVYLRNNTGVLSSFELGHTYITVFMNCITCSRCLMILSRKYNEVMFSFVQKIHLFHHRHKSEYAYKTHIFIHKISHFYTVYLLGLALNGLLLFNMIPFYNCYSRGMFRDVIPANATYDHSVFYSVPFDYTTKFKGYIAMTSFNCFISYTCTSYFCVVDLTISLVIFHLWGHMILLTYHLANFKKPASVLESNENTHAIKDHSYTEEELKEVFSKLREYIRHHNLILNFSSEMSSAFGPALLAYMVFHQVSGCILLLECSQLDMKTLVRYGPLTVVIFQQLIQISVIFELLGSSNDKLIDAVYLVPWEYMDTKNRKLVYVMLRQSQRSIDLKMMSMLTVGVQTMTAILKTSFSYFVMLKTVAEEE

>HassOR14

MQTHKQVHKISHLFTLLLTGQMVAGMSLFNLTPMYNNFSTGKYKRGGLKNSTFEHSLYFSYPFNASSDVRGYILSNIFHWIISYLCSTWFCTLDLFLSIMVFHVWGHFKILIHDLNHFPRPLSMISFRLDHSNITLTNEIYSSRELVHVSERLNKCVEYHRRIVSFTDKMSEVFGPMLFVYYGFHQTSGCLLLLECSQMTVEALVRYLPLTIIVFQQLIQLSIIFELVGSVVRHMRIKYCFYFS

>HassOR14.2

MAGLRDFFFNFEANEAITTPTNYPYLIIIRRSLSVIKCWPKKTTENLEAGAKLKAKVWGMIQNVLHLGFCAFTIFGTATYIAIHKKNMTFFELGHLYITLMISCVVVSRLLTLTFQEEYQVVVNEFLNKIHLFYYKDNSEFSMQTHKQIHRISHLFTLYITGQLFTGLSLFNLTPMYNNYSAGKYSKEGLKNSTFEHSLYYSYPFDVSTDVRGYIFSNILHWFFSYIVSTWFCTLDLFLSVIVFHIWGHFKILLHDIDNFPRPSKMVSFRLENTDVTISNENYSTKELEQLAAKLKQCIDYHREIISFTNKISDVFGPMLFAYYGFHQASGCLLLLECSQMTPEALARYLPLTLTLFQQLIQLSIVFELVGTVSSKLNDAVYGLPWEDMDVKNRRTVAFFLLNVQEPVHVKALGLADVGVTSMTAILKTSMSFFTFLRSM

>HassOR16

MGLRQFLFENEAVEGINSASDYLHIKVLRFMLVIVNSWPRKEIGEPESPKFSEFVKYLYLVVTFLLSAGFILYVVKHNSELTFLETGHMYIVLLMSFIDVSRVATLTMSTTYREVARDFLTKIHLFYYKDRSKQAMETHRAVHKIAHLFTLWLVCQMLSGLSLFNLIPMYSNYAAGRFSGEVSKNSTFEHSMYYPYPFDTSTDIRGYSIACILHWIISYLCSTWFCMFDLFLSLMVFHLWGHFKILNYTLNDFPRPSSKVEAAKYSEEELVDVAARLKDCIVYHREIILFTDRISNVFGPMLFLYYMFHQASGCLLLLECSQMTAQALIRYVPLTVILTQQLIQLSVIFELVGSESDKLKHAVYGVPWECMDVKNRRSVAIFLANTQEPVHVKAMGVAKVGVTSMAAILKTSLSYFTFLRSMETGRL

>HassOR1

MTSILRKYCYKRKSKKRLNEKVYTTKDYDESYAPTKKVLGWVAIRMTHSISENATKWWDRFYWFEMFNLFLTGPSEMVSMVSTAYEAKTFLDSIKVFRMMPCFGCVVLSMFKSINMVIHRPVFENLTNELRAMWPQGEVSEEEHEIISGALKQLNIIVKGYYWCNNALLISFLSPPYFLTLARYFGHDSPMGLHFLYWLPFDPYQPVYYEITLVIQTWHACVVIYFNVAWDMLFCLFLCHITTQFDLLARRVKRLFYVTVDQQLVRSYPMALVSEEMLRVEGERVRSQGDNYWQTRHHAEITQIVLRHHALIRLTGDVERMFSLALLINFMNSSIIICFCGFCCVLIEEWNEVAYKSFLVTALTQTWLLCWYGQKLIDSSKRLADALYDCGWYNASKKARSAVLIMLHRAQKGIYVTTHGFSVISLASYSTIIKTAWSYFTLLLNFFKDKSANL

>HassOR3

MANYVMFITGVTAAVIGAFSLLALFEGTMSIEAWLPFDPMESLMNQILSLEILAFCVFPGLCRAFAMQGLVCSMIMYLCDQLIHLQKELRDLTYVKETEMVMRKKFKNAIRKHIRLMGYSGRMENIFKEYFLVQNLAVTVELCLNAVMMTVVGVHQITLLITFLAYLMLALVNAYIYCYLGNELIIQSQGIALAAYESTWTSWPVDLQKDLLIVILAAQRPLKLSAGGMALLCIQTFSQALYNGYSIFAVLNDAVN

>HassOR7

MKIKMSKPLIFDQSIEKLNVLFRFSGMNVKNKIVTPLDTIKYRWLYTLNFLVVFSAIIGSVYYVILGIKQGKNFIEVTSVAPCLTFSILSMIKSLYHLMYEEHIQELIDLLTELEIRENNREKCIEKEEIIANETGFLNKVINVLYVLNCSMIVVFDMTPMVMIAVKYYKTNEFEMLLPYLDVFSFIPYELKYWPFAYIHQIWSECVVLLDMAAADYLFFTCCTYIRVQFKLLQYDFERMIPDRSISKGVFYEENELRNKFTELLKWHQDIIYSSTILEIIYSKSTLFNFLSSSLVICLTGFNVTIVDDIVIIITFLTFLSMALMQVFFLCFFADLMMTASLEITNSVYNCKWYSANIKVGKQILFVQTRAQEPCKLTAAGFADVNLNAFMRVLSSAWSYFALLQTVYGAK

>HassOR8

MVLRQIDCFIINMKFLKYLAAWPGEDSTLRYKYYSRAFITIYIFVYMTLFTINFPFLPKQLDIFIEDMLFYFTDCAVVSKTLTIVFMREKIYDLFDMLESDIFQPDDSEGLAIIEKAKKLYKLYWKILTTVSFTSSAAHLSPLLVHFIVGTELELPVCSYSFLSENFRQIFVVPLYLYQGSGAMFHMMYNVSIDTFFAGLMVLTIAQLDVLDKKLRKVTDKDKHEDANGETCRQHHDKHREAVKKINQCIIHYEEINKFCGLVQDVFGISLFVQFGMGSCIICTCLMRLTMPAPLSYFVCLGTYLFVMVLQFMVPCWFGQRIIDKSNHLAFSAYNCKWTSETRQFKSNMRFFVERANKPLSITGGKMFCLSLVTFTSIMNSAYSFFTLLQNVKSRK

>HassOR8.2

MALRQIDCFKTNMKFWKLLAVWPGEDSTARYKYYSTAFITTYIFVYMILFTINFPFLPKQLDIFIEDLLFYFTDCTVVSKSLTMILKREKIYDLFDMLESDIFQPDDAEGLAIIEKAKKLYKLYWKILTTVSFTSSAAHLSPIIVHFILGTELELPVCSYGFLSEAFREMFVVPLYLYQGSGIMFHMMYNVNIDSFLAGLMVLAIAQLDVLDKKLRKVTDKDKHEDANGETCRQHHDKHREAVRKINQCIIHYEEINKFCRLVQDVFGISLFVQFGMGSCIICTCLMRLTMPAPLSYFVCLGTYLFVMVLQFMVPCWFGQRIIDKSNLLAFSVYNCEWTSETRQFK

>HassOR9

MLEQFDRCLKSVNLYLKFLGLHLESKDTNKTFAQRSRSHRLYFAHLFSLNLEVAAQVLWVLEAVITGKSFVEITRLIPCLILCLISNFKTLSLLYYGRHNDEFIVTMRSLLLNQMQVEEKENRFRKELIDKHVLILTSISKKISYVIVLGLLMFALAPAFIIIPHYFKTDEVKLEVPFIGYYPFNEFDLRIYPWVYLHQVGTAIVAIIMVYGPDCFFFTCCTFIHIQFSLLNNDMERIVTEETPRYDKTKFKKLAVRHIELMRCVNLLEKIFSKSILFNALTSSVIICVTGFNVLVVDNIVMMASFTAFLIFGLMQIFLYCYYGDSIMRSSMEVSISIYNSLWYKIPAADRKGFLIVIIRAQKPCALTANGFFNMNLSAFASILSKSWSYFALLKTMYHPE

>HassOR10

MAVKNTSLFLGRPKKILSAHGVWPHPNNFVILRKLYMLFVMWTQYSFLLFEIIYIADVWGDIDAVSEASYLLFTQASLCYKSTAFMVNKQSLLELLEIMDSEIFEPKSAEHEKILAAQARKIKRLCLFFLTSATTTCTLWAMIPLFDAASKRSFPFRIWMPVTPLKSPDYELGYLYQMVSIYISAFLFISVDSVAVSMIMFGCAQLEIIMDKIQKIKYVFESADSEEGRRNIIKTNNEFLVECIKQHQTVERFIQLCEDTYHANIFFQLTGTVAIICNIGLRISIVEPNSVQFFSMVNYMVTMLSQLFLYCWCGHELTIRSENLREWLYQCPWYEQDTKFKRALFIAMERMKKPIIFKAGHYISLSRPTFVAILRCSYSYFAVLNRVNTE

>HassOR12

MEDEPLLIDKTVKKIEFLFRCTGINIKSGTKTRKDMIKSRTVYIINFLWLNIDLAGAIVWFFTGIANSKSFTELTYVAPCITLSFLGNLKSLFLILREEHVDKLIQVLRDLEINEKSRPKSEETDAIIKYEHNFVTTVISVLNVLYFVLLVAFALSPVSLVALKYFTTNELELLLPFLIVYPFDPYDIRYWPWVYLRQIWSEVVVIIDICTADYLFYTFCTYIRMQFRLLKHYIERVIPEEDDAGRLANIEQVRAEFVLLIKWHQDLISSANMLETVYTRSTLFNFVSSSVLICLTGFNVMAISDVAFVATFLSFLFMSLLQIFFLCFFGDLLMTSSTEISEAVYNCRWYLADTSLGKDLLLVQTRAQTPCKLTASDFSEVNLKAFMKILSTAWSYFALLQTLYGAPT

>HassOR17

MSLRACVRRSECARSVAPHVRVLRRVGFLRGAALSSRGRAERLALRSYHALALAATSLYVLQQAVYAYQERGDMDKLSQVMFLMLCHVTCVVKQIAFHVDADRIDRLIASLDEPLLNQCAGERGALLRGTARGAARLLRTYAGCAVATCVLWIVFPVINRIQGISFEFPFWTGFSYDHNAVFTLVLLQSFYCTNLVAIGNTSMDAFMATILDQCKTQLRILRINFESLPERARALHVESGENYDTILDKLFVDCLVHYNKITEMCTELHDVFAVPLLVQFGVGGWILCMAAYKIVSLDVLSIEFASITLFITCILIELFIFCYYGNEVTVESERVSQSLYSMEWRRARLTFRRSLVLVMERAKRPLRPAAGRVIPLSLDTFVKILKSSYSFYAVLRQTK

>HassOR18

MEMKVDVQPEKKYKGFNETFKLCAFSLAFAFLYPNRTTALRRCITITLIVTFCGGQLFWFITYTFKCLYTLDIYNFARNMTLAVVLVLFFIKTYYVIYATSKFAPLLDKISDDLLEANNLEEEFQVLYDDHIKIAKVGEISWLLIPTIMSALFPIYAGALMTIESIQTDDYERRMVHDMELLFVEDIQSETPFFQCMFAYNCVQCVVLVPNYCGFDGSFCIATTHLRLKLKLMTLKVNKAFKYSKSRQELRMRLYDSIKDHQDALDFYVQLQNVYGPWLFAVFLLTSFMISFNLYQIYLLQRIDPKYTSFGVVGVLHIYLPCRYASDLTRVSEEIPDDLYLAQWEAWADPSITKLLMFMITRAQKEMIVTGMGLVVFNMEMFKSILQTSYSFFTLITA

>HassOR19

MKDRDILRNYCKIMFYIGSGNCWYKEDEIGNDRSLLYRAFGALLIFLYGSMTILEIMAFTIGNFPVEEKRECLSFASSHAVVMLKIFLFIFNKPLIKSLNHKIVSICEDYDDSALMAKKYKTMKRNVIIYFGIVYGTTLFYIAGGLRNMFRGSHFVTVVTYYPSFDDNSPLANFVRVLNTIILSMMMLTMIISLDSCIVIYLIMYRYKFMTLSKYFENLREEFFALINRQEVELATEKMANGLVEGIKMHSSLIRLKPEIDQAFATFMALQVFESSGVAVCLLLQIALSDEHVPLVVTIKIVFFVFALFFLLGLCLCNAGDITHEASKLSNSVFYCGWQSCPPRCKSAPKRNIRKLVLLAIMQTQRPPVMKAFKMLELNYATFIQVVRTTYSVFALFYAQNK

>HassOR20

MDDELEFKPFHETYRLITFSLCIAMIYPNPRTEKWRLISIPILIATVAPVAIMIFLDMYKCWTNGDIVNIIRHSTVVGPFLGGFFKMILMYHKRIQAKQILDEFDRDHHMFNSVAETYKDIARASIRNCQVYSERLWACLVTTCVMTFPVMAIVLNIYNFMFKSEPTKYMIHDLEKPFSKEPEERFESPYFELLFVYMFYAAILYVVNFTGYDGFFGLCVNHACLKMELYCKALEEAMMADREEVYGRVIAVIREQCRMFRYVDLIQDTFNIWLGIIFIATMIQICTCLYHITEGYGFDIRYMIFVYGAVVHIYLPCRYAAKLKAMSMETSNRFYCCGWERVDDERVRKMIVFMIARAQVPNEITAFNMMAFDMELFLSILQTSYSMFTLLRS

>HassOR21

MDNFTGYYKSSNTTKFLINLNKFGFIFGLPNFWIEELDFSDTFLKIIGRLNKYGNWLVFGLILAEYGAYFTQKNLDERQTSDLILFIISHTIITGFRVRISHQEVQIRNVMYKLGIALKEVYNDSEAEDQMIKRSKFFSYALVLNCIMSVLMYTVAAVMRVIRAGVTFTTIITVYPTVEDRSTLSDVVRAIFYIIWCIYLTRVFAVYTLVICLTIAMSHQFKNITSYFYSLSNIFEDEQMTQTEKEQEYERSFRAGIKIHSETLNCTGDIQRICRDVFSGQIIFNLTLLIVLMYQMVNSPRNLTNALTLVIAGLTILLSTGFFMWNAGDITVEAQLLPTAMFSSGWENCGRDSSVRVRKLIVIAMMQAQEPVVLTGLGLIALSYQSYVSIVKSSYSVFSVLY

>HassOR21.2

MSKFIMNRPIPPFKENAMTTFLDELNTIFFLVGLTDLWISEVKFSKRFIKIYKKINYVMDFLCLFFVVFLFGSYFTQKDLTEKLANDRLMFSIILPGNLVLYYISVYYKEEIRNLLYHLTVVLKEQHNDTRLEREMIRKIRVFSITLNSIAFIVDTSYGFGALYEVVTKGENFNTIVPAWPDVHDDSSLAGAMRVFFYFCWLNPIATRILTTFSLLLTEMVAICYQFRNLQSYFYSLDDIFSDDSLSQKEKEIKYEEGFKIGIRMHIMTLWCKKLHQHVNKEILAIEMVLFFAMLMSELTTLLGGERNASQLCMMILISVSTCISLGFFMWNGGDITIEASKISEAMYSSGWQHCRGTSSVRMRKLVTFAIKQAQDPVVYKTLGVVDLSHTSYVTLVKMPYSAVSVLY

>HassOR22

MNCFKNKNKVEVLMTPLVYTETDTTRMIEKYNKFWFLCCCPDFWVNKVDFSDTFVKIYRPAMMAIHVIMVIFCFSCTLSLWTQKNLSESQQSDRLAYGASAPIITIFYHFVILCYKDDVRKVLYKLVVVLKVDHNDKQAEKEMMEQSRLHNGLFFSSCVCNMVFVGLYNYYVAVTTDATFITCISAWPDIEERSLLAGLTRVVVYFVWFAHVVRNMGVFLIIHTVLLCLTQQYKNLQSYFEDLNKIFDETKLSQEEKELKYEVKFKRGIEQHALTLWCVDETQRVFKITFSSHVLLWCGLLISILPDVMNSDDHTLKMLVSNAPRVAAALVGLGYFMWPAGDMSVEASNLPQAMYGSGWQCCYDRSSRVRKLVVLAMMQAQRQIEMKAFGHLTFSYETYVAIVKMSYSLFSVFY

>HassOR23

MEETFLAFHRVLSFAGISIFAKNNWNSKWWLFLQIMNFIVGNLCFVFTTGFVATSYTDFLLCIQGACIWTTGVIMTISIWVCLIFRKKFRRFLEEMVFQDGMLGMPLIRHIFLVSTKGERINELRELVNDSQNKLFKYTRVLLKTYVLSVFVCATLYLCTPIYLMIVREDKSLRLLAFDMWFPWSLENYTVYIVSFIFHAYAGYLCCIAYPGLQSTIILLIGQVIRQTRILTFILLHLNELVLEVTGVQDEEWQTYCTLVHSQCVDHYVKIKRFSNRLNVICRPFYLTLILVAIMLVCMCSVKIAISDKLSLDTMKYYVHEFCFILMVLMFCLLGQQVENECEALEKAATEKWYIFNKTHKVNVLIFNMALSQRMPIYIFGTITLSLPTFTWFIKTGMSFFTLVMSVLEEGEY

>HassOR24

MDSKMSLSSASLATHLRLLRWCGYCRLAGGARLSRLHALYRALTLALTTVYLLQECVYAYQVQQDMDKLARVMFLLLCHITSIAKQLVFHLKAERIDEMLAGLEDPLYNQPEEAHRRLLGATAASASRFVRAYSGCAVVTCTLWITFPVMYRLQGLPVEFPFWITVDYNRPTMFILVLAYSYYVTTLVGIANTTMDAFMATVLNQCKTQLRLLRMNFECLPERAAALSRQLGGSYDAALFALFRECLVHYEKITETAKMLQNIFGTAILIQFGIGGWILCMAAYKIVSLNMLSVEFASMALFISCILTELFLYCYYGNEVTDESERVSQSLYSMEWRRARLTFRRSLVLVMERAKRPLRPAAGRVIPLSLDTFVKIIKSSYTFYAVLRQTK

>HassOR25

MPSDQSRMFDPPLTVLKIFGVWEGRTPSKYYKTLSFLFLFVSWFFYNFLLTLNLVYTPRSVELFLRELIFYFTEISIASKFLTVLLLRDKILEVFSIIDSDEFVGDYENKDGILYRTNKGYSFCWKVYNVLANIDYTCVIIMPVVIDLIRGTKSVLPICNYYFLSEDFRDSHFVILYLYQSIGMYGHMMYNLNMDSLAWGLLAVGIAQIKVLNKNFTDLKLTAEESELPLEIQDNIQKTRLFKLLRHYEAILNYCDAIQNVLSVTFFFQFSFGALTTCDIMCSLLMPGTMVYRIFLVIYFFVMAGQIAVPGFFGTLLTHESEELVTAAYNCEWIERSQSFKRTLILFRERAGTPIIIWGMKMFPLSLVTFVAIMKTTYSFFTLIRNAQET

>HassOR26

MEENMKYSTFQGFRPHFDALARVGYFKIVLKPLSLQKQFLHNTYRFLSWTFILTYNLQHVIRVVQVRHSTNLIVDTLFILLTTLNSLGKQVAFNLRTQRIDKLIDIINGPVFEASKPYHVKVLKENALVMSKLLTLYHGAIFTCGTMWTVFPIVNRALGEEVQFTGYFPFETSSTITFSLALAYMIILITFQAYGNVTMDCTIVAFYAQAKIQIQMLRHNLEQLVEFDHSFRINTQLNKRTDLYSTSYKDEQQERVAIQERLKKCVQHYYQILRFAKEVESIFGEAMVVQFFVMAWVICMTMYKIVGLSIYSAEFVSMAVYLGCMLAQLFIYCYFGTQLKVESELVNQSIYCCDWMKLSPRFRRQLLVMMQCCGRPIAPRTAYVIPMSLDTYIAVLRSSYTLFTFLNR

>HassOR27

MLSKIKNIIWCLGRQKIRNGEIDSVVTLLDRLILYNSGLASYTTTYKVHWTAHVLLTCFIIACILQIIALFMGKDDPDRLFECFSVLSFCAMGMLKLLSLRKNHRKWRKLLSQITILENTQLSNRSISYVEYQSDSEDSDNFSEHISIYTKKFRGTSIVLTRIYSFTAFIFILSPFAERIICEIRGVECVGYPHVFPGWTPLDDFSIFGYLVTVLCEIFSAVYCVCVHMAFDLTVIGIMIFVCGQFSLLRDYSSRIGGKGRQCNLSLRRDERARFRIIRCHDINLLLVNSITELDMLLKNIIGVYFFVATLTLCSVAVRLKSEDIGVMQLVSLIQYMCGTLTQLFLFCRYGDAVLHESTMGMGEGPFAAASWCLSPRVRRDLSMLSAGMMSQRHLRAGPFSFIDLPSFIQVVRAAYSYYAVLGKKE

>HassOR28

METIRKFGLQYCDVETMLWNVSVLSRTLLLNIDERNKKPIPIISYILGISISLGYFYVYLVSMTWFVFHRCAANGDLLAAIVVFSLGVSSEIGTVKLIFMFLHIGTVKQIVTECLACQATVVPGSRFSNNLLATLRDVKKRAMVFWIVIIANGIVYVAKPIILPGRHFSEDLFIFYGLEPMQENPNYQIASLLQAAGVIFTCYPPATITAFLIVVSGYVEAQMLSLTEELLHLWEDAEAHYYKTNQVISVIDHNKERNLKSKAVNEYVEKHLKDIIQSHGRNINLLHKLEHVFSGAIALEFLLLVIGLIAELLGGLENTYLEIPFALMQVGMDCFTGQRVMDASVKFERAVYDCKWENYNLSNMKIVQTMLQSSQKTMKLSAGGIIMLSSSC

>HassOR29

MGYQQIDCFDIHLKILRILGVWPHDNPSIYYIYFSRIFVLTFTVLYVVIYTMNFYFLPQQLEVFADELIFYFTNVGALSKALAFIFLRDKVKKMLFMLESEIFQSDDPEEIKLIKEGKEKSNFYWKITAGLSVSANTVNVCLPLLVHIIFSVELEFPVCRYSFIPEKYEAMFAYPAYFYQSIGITTHMLYNVNIDTFLLGVMFLAMTQLDILDRKLRKVTDVCINPDAARGSVDKFIDDQNAVLEIIKCIKHYDAICEYCKLIQDAFSEILFVLFSSGSCKICMCLFRFTMPATTGYFVFLTLYVTVMTLQVMVPCWFGSRLMDKSSQITIAAYDCDWTPRCRRFKSNLRLLVERANRPITIIGGKMFLLSLATFTAIMNSSYSFFTLMRHMQSR

>HassOR30

MISSEDLFLNRAKFVMKHLGVWIPAENGSILNRAYRAFMMTLQYLFLIFQMIYIVQVWGDLDAVSQASYLLFTQACLCLKVTIFQINIDMLKELLQFMDADIFKPDNEVHENILKLQAARIKRLLLAFMVSSQITCGLWAMKPLFDDADRKFPFDMWMPVSPEKAVQYYIGYAFQLGTICISAYMYFGVDSVVFSSVIFGCAQIDIIKEKIMSITSVDRKHGTKEALSQNYKKLVDCIKHHQAIVTFTELVENAYHPYLLFQLVGSVGIICMSALRILVVDWRSMQFFSILTYVSVMISQLFVCCWCGHELTATSEDLHTVLYKCIWYEQDVKFKRELCFAMMRISRPLVLRAGHYIILSRQTFVAILRMSYSYFAVLNQTT

>HassOR31

MNSILQNLEDPDRPFLGPNYWIIKNMGLLLPKNLLAKILYIILHEIVAFFVITQYMELYVIRSDLDLVLTNMKISMLSVVCIVKVHSFIFWQKHWREVLDYVTAADKFERQSDDPIKSRIVETYTRYCRRLTYFYWVLVFTTFLTTTGTPLMRYLSSSSFRQNMRNGTEPFPHIFSSWMPIDKYHSPGCWITVLWHTLLCAYGAAIMAAYDTCIVVIMVFFGGKLDLLRERCKQMFGPSTISDRQCEEVVRQLHGIHVMMLKYSRLFNSLLSPVMFFYMVMCSLMLCASAYQLTSAQNAAQKLLMAEYLIFGIAQLFVFCWHSNDVLIKNENMTSGPFESNWFLANYRQRKDVLTLSGQLCIKNIFTAGPFANLTLPTFINILKGAYSYYTLLRK

>HassOR32

MDLDFNKIFIIPTTALRLARAHPYIPRDKKWILQFVTVHSLFTLTFLLILYNIFCHDLKANNFTQTCQNGVLFVVYIVISYQYAVLLTHQDTLVGLIADMNKDFQTSKDLPPKEQDSIKKYMNQGLWVCKQWLFLTISGCAIFLLKNLTLMLYYYCINEFRLVPFYEVVLYPPIMEENKDNIFVYLLMYAIMLLFSAYSALMYTAFVPLGPIFILHACGQLVLVKLRIDNLFVECDDEVIRKKLKGIILHLQYVHSFVNRIQQVFKIGYELTLKFTALILPITIYAVLEGFYRGEVNLEFVTFIVGGVMISGSPCYYSDLLMEKGEDVRMSLYTCGWEQHYDRRTRTTLQLMLQNALKPIAIHTVFTVMCLDALTDLFQQSYAIFNLMNCMWN

>HassOR33

MSGIRKFGLQHCDLPTSMENVAFFMKAVTLKIDERDEGAIPIIFYFLTLFCIACYLYVYVVSMIWFVFSRCPVTGDVLAALIVFSLGVASEISTVKFLYMRIHIKDVRKMVADCLDSYSKVVPGTRFSNNLLRTLREAKRRAMLFWIVIIGNGLMYVLKPMLLPGRHFMDDVVLLYGLEPMFETPNYQISFVLMGSSCVLICYLCANISAFLIIVTGYVQAQMLALSEELTHLWEDAEENYRGTELEDITDDGDQNNRNKDAILNDYVTVHLKDIAKSHAENINLLGQIEGTFRGAIAIEFCLLVIALIAELLGGLQNTYMEVPFALMQVGMDCLIGQRVMDAGAVFEDAVYDCKWERFNKKNMKTAMVLLLNAQRPMTISAGGVTTLSYVSFMTIIKSIYSTYTTLRSTMDEP

>HassOR34

MSAKFSIMQIFKFLEDPAYPSVGPHLKLLGFTGLWHPNRHTLVGRFKHILFYITISFFFSQYIKCFINFNASSLKLILQYAPFHMGIVKSCFFQKDYKTWQQVIDYMSSVELAQLSKSNKEQYKIIYDYIKRNRKVSYFFWALAFFSNFSIFTEPYQKNEINVNGTSIYLNIFDGYTPFQKEPPGYYISMLIQTVLGHIVSAYVVGWDTLVVSIMIFFAGQLKITCLRCKMMIDVTNPMKSHLKIAECHRFHTTLVEYTRIFNALISPVMFVYLVVISVNLGVCIIQIVEIENDVPTLVSSIVFVMACLIQLLLFYWYANEVTHESTFVSYSTFESNWPEANNKLQREVALLGLTTEKMLVFKAGPFNQMSLATFIAILRASYSFYTLLNSTN

>HassOR35

MLYTDLPVDYKQTFGRYTYLLKCIGLDFFDNSTSFVSWNNIKLLLYCIAFYSFAYTQFSLISEIRADNFLEGVRVLPVDIMIAHDVVKMMFALVRRADLRDMILEVGELWPKNLAPDDQKAVILKAWLRKFKIPLDLYFTFALVNLSLFELIPFFITCFYLVKGDKVYLFPFQLPKFWEVDSFITYLLTYVWEMASTVSCHMCLYLPFDLIIVIMTSNVSALLRLLQVDLKNAIKLRDEQHKTKSNLFFSDTQSYEELKRIVEIHQRLIRIADQLSSFFGLVIFIHVACAAFEICFFGFLTMVYGGLATFSNMLAVLNVVFTVFVLSLSGQFLCDTSSEVADAAYESYWYESDHKVKKLILLIIIRAQRPCYLSALGFSELTLKSFSKIMSSAWTYFSLLIQMYEET

>HassOR36

MENIPPYEEVLKQIKINLWLAGIPYGDPKMKFRYYLGNLHLLVMIICEISFFVSRISAENFLELTQLAPCLGTGILTYLKIVVIARKRMKIYDLSECLGKLYENILNDDNKRRLVKKNLVLVNFLMKYYFVLNVILISVYNFVSPVIIMYDYFVNNQLIFTLPYAVLVPFSTDAWIPWSAVYIYSIISGFVCVLFFTTVDGLYFVLTSHVCANFSVISDMIERLDENSVDRLPDIVKEHQYILKLSEDLEDIFTAPNLFNVLVGSLEICALGFNLTNGSWEKIPGCLLFLLSVLLQILMMSVFGENMIRESTNIGDAAFLCKWYKMDEKSKKTIMTIMIRSKKPQRLTAYKFSSISYASFTKIISTSWSYFTILKTVYTPPELSSSQ

>HassOR38

MIILSENIKQKLAFLSPYLPYGVIESWEDLNPRLYHAVHIYWLKFYGMWFNNYSPNNIKFWLHMAYTLTVLWLVCFFPGIGEVVYLLKQRENIGDIADGLYLFLSEMYTYVKIAVFWMNRDKVISLLEYLHCKEFKPKEPEHRDIITKSIKSARFVMTYYSTMCVGAVSVGIIMPLTENFDILPTNVEYPFFNVYRSPAYEAVYFHHIYYKPATCIIDGVMDTILAAFVASAIGQIEILAFNLRNFNLVAERQRKRDLAQNKYMEEYPAQHYVRSVLKECIRHHNCIIRYVSMIESAFSLASALQFMLSVMVLCLIGIQFLSIENPSAHPMQIAWMGIYLTCMLIEVFILCWFGDELIWKSMDLAKAAFEGPWMNSDRQTNMFIIILLERCKRPLRLSAGKIFTLSLDTYTVLINWSYKAFAVMRNMKK

>HassOR39

MKCIEWLKQCKRRLGQNSFENLIWLLDLLPALLGSPLLTEKFNYLFWTMHAIVVFYLYVVGIAVYQKYSAEGFIDSMNSLFNFFALILIAVTVWWVHSKRKEFNDLLKVAQKNDDLIIETGRFLHVHEKMLRSIKIIIILCYVFHFINEVVVYIPFRILRMEDFSIASCVGLEPLNVSPNREVCMGLMTANILISIMVICCYDISLLFLFSHTTAVFQILFEEMMSLHDITQTCRNSDEDYAVIVARLKNVIVRHVLALQTVGKVEDIFSVSIGICFGLDGISLCLFFVLPLEVILHFAPLIYHSLFILFLYCFQGQRLTTASEKFEMAVYCCGWENLRVKERKQVLLMLKQAQKPVIVYAAKVIPIRLSTFATTMQGIYKFVTVFK

>HassOR40

MVLKFLDRLEDPNNPLLGPTVLALRYGGLWQKDRVKHFLYNLVHFIAFLFVVSQYVELWIIRKNLEMAMRNLSFTMLSTVCVVKACNLMLWQNSWKELIDYVSELERSQLSKNDAVVNKIISDYVKYARRVTYLYWALVTATVVTVILAPLFIYLSSPNYQESIKNGSAPYPEIMSSWTPFDRSRGLGFCGATLYQMLACFYGGTVVANFDSTAVVIMTFFTGQLKVLSVNCERLFGDGNELVDYDEAVKRIKECHLHHYYMVKFSSVLNSLLSPVLFLYVIICSLMICASAVQLTTEGTGNMQRIWIAEYLMALIAQLFLYCWHSNDVLYMSNKVDDGVYSSAWWSQNVRIRRSLLLLGGQLRRPIIFTAGPFTLLNMATFVAILKGSYSYYTLLAKKDD

>HassOR41

MLKNILQKLENPKRPLLGPNVKALKFWGLLLPKNVLMKYFYLLMHVLVTIFTATEYIDVWFVKSDMNLLLNNLKITMLATVSVIKVTTFLCWQKYWVSIIEYVTRADLNQRLTTDKQKLELLATFTKYCRKITYLYWSLMYTTVIIVMVQPIFKYISSENYRTSVKNGTDTYLQVVSSWVPFDKNTMVGYLLASVFQSYAAIYGGGWITSFDTNSMVIMVFFRVELELLRLDAKDIFGMESSKVQHEVALKRLKNCHRRHVELVKFARLFDSCLSPIMLLYMFVCSVMLCVTAYQITIETNPMQRFLTTEYLVFGVAQLFIYCWHSNDVLFASADLMRGPYESIWWTRSVRYRKDLYLQAAQFNKTVVFSAGPFTKLTVATFISILKGAYSYYTLLSQSQMK

>HassOR42

MTKSRPRHYFGFHYRILRFLGLGWWHHPEEGKTTNFPGWYLYYSIVTQVVWVAGFVGLETIDPFVGEKEMDRFMFSLSFVITHNLTLIKLYIFFFKNVDIQEIVRTLEIELYDYYQNIEKNRKTVKISKIITGSFIFFGWLTIGNGNVYGTIQDLHWKSLVATLNDTSQIPVRTLPQPIYIPWNYQKDKSYIPTFVLETVGLLWTGHIVMTIDTFIASVILHMGAQFEILNEAITTAYDRTMTSLREGIRHEDSGHQGQSDRQTSILSVEDSNERIVHAFIPKEEIDAALQTTFRNCFRQHQVLINCVEKFSRTYSYGFMTQLLSSMAAICVVMVQVSQDASSFKSVRLITSVAFFFAMITQLGMQCFTGNELTLQAERISDAVMQCKWERIPTRQRRLLLMMMMRAQRPLRLTAAGFTNMDNACFLAIMKAAYSYYAVLSQRQE

>HassOR43

MSLMVKNENRSLQCCLTVLKVAGFLTPPRDGRIPRFTRRLYCFAVFMFLVGCIIIAQTGAMFEIWGDLALMTSASFLLFTNLAFATKIINVVVRSREIQEIIDEGDADLLAEDRYMGIEVIKSCNMETTTSMGLYTLLSGVTVFGWAASAEKNQLPLRAWYPYDTSKSPAYELTYIDQSSAVTLAALVNVCLDTLVTSLIAVCRCRLRLVALSLRTLCDGIPLPDKQLISPTEERIVLTRLSQCIIKHEAALKAAREIQRCFSLPILAQFAVSVVIICVTAYQLAMELNNRNWFRCIPMVAYLLCMALEVFLYCYQGNELLEESSEIAGAAYECPWYQCSVRMRRTLLIVMVRTRRQLRLTASGITTLSLACFTSIIKGSYTFFTVLQQAEDRDPK

>HassOR44

MWILHSVWRKLTQSRAVDSAGPLERAFFESVYRLAFVTGLSTSDNYMAYMIYSSSVRMLSALVVICEIWHALGNNMSLDELISSVNVIFIHLITLWKLMIMVSNQKVFKKLAKALESPSFDISTENRQAIVNHWVLTHKKYLKVLLLLAYLTLAVWQLYPLVDDMDFNLMVDVKLPFAYDSPLRYVISYLFVGTMFSYASSMVIMSEVIMQAHLIPLVCQFNVLANCFENVFEECASEFPDINKHELVKHNRFVEKYRKRLGDLVKQHREILDQTTDLKTILSAPMLGQLACSGLLICFVGYQATATIAENLGKFVMSLFYLGYNMFTLYIICRWCEEITIQSQRIGQSAYFSGWESGVSHVPGARATIILVIARSNKPLVFLAGGMYTLSLTSYTSLVKASYSALNILLTTKHE

>HassOR45

MRLQILEDFLLKKTFDFDRPDINLYNFHPQLRILLAVKGVFFTNRRSLLRFIWPCICIQLSIVAMTLEEIFIWRGVTVKDYSFATECFCYWVILGCIPMVYISIVVNTNKIYDIVVTMNEDFIYVCSLGDRYRKPFLEGQLLIWQLCYAWFIFVCFVGGLYVIIPLVGLLYQSLFATIDENTVRPLQFPMWLPNDDPYRTPNYEIFLVIESTLIFCFVQTFCVYIYTLLHILLHYYTIMNMIIIDFSVIFEGLDESVALLPRNDQRRRDTQLILNARIAKIVRWHLSVFKAVSTVSSVYGPPLVYQVSFSSLAICLIAYQIAEKLDNGKVDILFCLLGIAACLQLWIPCHLGTMIRNKAFEVGDAGWTCGWHETPLGLMIRNDILIIILRAQKPVTIKFTGLPSVQLETFSSTMSSAYSYFNMLRQYSK

>HassOR46

MSEAKPSTLNSEKTGRYIHYIEIPLKLVACWEFFPNSTSERLLVINDIYLGIVLFVLTYIPTCLTVHLYTEWQDIMSSLGTIADSLPLLVSLIIVAYYAMYRQDLYDLMEFMEKNFKYHSARGLMNMTMEESCKTAQRFARIYTACTMFSVTMYATMPVLIHLWTKEPIQSWMYMDITRSPFYEFVFLVSCLAQMFVGLAMGQFGVFFASNSILICGQLDLLCCSLRNVRYTALLQHGVKHASLRVSHATIQDDEKHNYIYNVSEMKESVYHYDKKVSNLYAEAKTQFDIYSSAFDDATVNALRDCASLCQVINKYKEMFENFVSPLLALRVVQVTLYLCTLLYAATLKFDMITVEYLAAVALDIFVYCYYGNQIILQADRVSTAAYQSMWQTMGVRPRRVLLNILLANRRPVIVKAGRFLPMDLHTFVVIIKTSFSYYTLLVNVNEK

>HassOR47

MPVTSKFLTVLLLRDEILEIFNFIDCDEFVGDYEDKDGILYKTNMGYRLVWKLYFVLSHVAFTCDIILPIVFDVIRGDKSELPICKYYFLSDEDRDSHFMYLFLYQSIGMYGHMFYNLNVDLFSAGLLAVAIVQMKILNKKFRSLKLLAEDRELPFEIQDKIQIIRINKLLRHYELILK

>HassOR48

MVIVSITITLKYMVLLYHQASIREIINIMEEDYRRARDTSKEDLDIVVRYAERGQTVCKFWLVFGFGTSAIFPIKAFILMAYYTWKDKFVFVPLFDLTYPQPIEASKNVTVVFWILFVVTFVFDVYAASMYVGFDTMLPIFMLHTCGQLDLLNLRISKLFVEAEDCAEIEEGLKKIICKLQDLYKLVDRVKKNYSVLYEYNMKATTFLLPLTMFQIVEELRVKRINVEFISFFAASALHFFMPCYYSDLLMETSEKFRQAIYSCRWEKCYNKRIRQIVLFMMTRARIPLGITTVFYVINLDTFAEMCRHSYGIFNLMNAACE

>HassOR50

MNFAIYIFGLPNFWIEDLKLSKRFVKIYDKISLFNDLLVYLLLVMEFGAFFTQKNLTDKQKFNLMVFAISHPLLCSFCVMVSKLKKKVRLVMYSQAVALKRDYNDPEVEKQMIARSLTYVLAFMSSCTITMIMFAIEAIWDVIRHGATFTTLITAYPDVQNRSILADVVRVLAFVTWWIFLTKMVAVYMLVIPLTISLRYQFKNLQSYFLSLAHLFERSDLSQKEKEEKYEAGLKFGIKLHSETLSCAEDTQDVCRGVFSGQIIFNILLLIVLMAQMVTSERTFVNMFGTVATSCTVITSTGFFMWNAGDVTVEASYLPTAIYFSGWQHCQRESSVRVRPLVVTCMSHAQQPVIFKGLGYIELSYQSFITIVKSSYSVFSVLY

>HassOR52

MRTLREIGQEIRKFGLEYCDLPTMFENVAILLRLLTLNIDIKYKGGITFYSYIITIVSGACYYYVFFFSMTWYVFWRSRELGEDIGAMIILSLGITSEIGPLKLFYMSYKKDKTQKIANDFLECDANTIKSTRFYANLLRHCRTVKKRAMLYWIVVAGNGVIYLLKPITMKGRNLPENYFLIFGLEPIFETPNYQIAYCMMVCAVFFVCYVPACVTAFLIVVTGYAESQMLALSEEMIQLWPDAIKRAEERTQLDPTKVLDVYNLEVKTIMNQFVEKRLKEIIKRHALVINLLNQVEIVFRQAIAMGFVLLIVGLLAELLGKLENTFLQMPFAFMQVSIDCFAGQRVMDASLVFEKAVYDCRWENFDKANMKLVLVMLQSSQKTLALSAGGISTLSFTALMSIYRGLYSSYTALRSTVK

>HassOR55

MMLLKKIVKYTKELEDPKNPLLGPTLKGLYLFGLWPIGNKFRSTVYNLFHFSTLLFVISEFTDLYVVRHDSNKVLNNMSLTVLSVICFAKCYSCVMWHSDWKELVRGISEEELRHIQKDDPVTRKHMEDYTRYTRIITFMFWVMVFITNFLLVLTPLLKYASSQSYREEIRMGIEPLPQILCSWFPFDNTKMPGYLGAIIVHVLMGSQGSGVLAVYDMNSVAIMSYLKGQMIILRHKCISLFDDVTSTEDVLERIKECHRHHNAILKHFLLFNSLLSPTMFLYVLMCSITICGSVVQFASKEATASQKLWVIQYTCGLVSQLFLYCWHSNEVTLQSRVVDGGIYNSDWWKSDVRVRKQLILLAGKLNHPLVLDAGPYTTLSVPTFIEIMKGSYSFFTLFSQIQED

>HassOR56

YFYSLVVTISQDHKMKDMEVNYDKIFKVNIKAMTLNQCHPDIPRNKKWFFRFFITHGIFSLVFLIIMYNIIFHDLANNDFSKTCQDGTLSVVYIVITFQYTIMVWKQDLLKDMIHIMRNDFEAIKISNREDQEVVLKYAQNGAWVGKQWLLISISASSMFPLKTFVTFIYNYVIGEFELIPIYDLVYPPFIEERKVSLFVYFSLNVLLIYYAIYAGLMYTSFVPLGPTFMLHACGRLELLTKRVNRLFEDNKGDEIMVELRNICVELQLIYDLVDHIKDTFRVAYELTLKATTIILPITVYEVLESFGRGEFSLEFITFIFGGTLISSSPCYYSELLMTKGEEFRQAVYSCGWERLYDRRARSSIALILERALRPTAIRTMFRTVCLDALADLFHQSYAIFNLMNAMWN

>HassOR57

MENNFENSLKLFLIPMRVVGTHPEIPINLNWFILYSLTYGPFTILAIIIIYNSYLNATNDDFSEACKNGILSLTYFGASLNNIIMLWYRDSIKNLLEMMKNDYKMVAGLPRDEQIIFQEYVRKKTLVCKVWLVLFTISCSLFSVKAILLMVYYAIIGEPRLVHLYDLVYPDFIESRKENLSMYLVIYFFIFSYGVYAGFVFMSFLPFGPVLMLHACGHLEITKKRIETLFTSNTKDVNEKLNDIVKLLQYTYNFVETVKECFKVFYEATLKLSALALPVTFYALLDGLQHGEFSLEFSSFIISGIALSSAPCYYSDLLLEKGREVSLALYTCGWEQEYNRRARSTILLLLIRSSRPIAMQTMFATLCLIALTEMFQQAYTIFNLINAVLT

>HassOR59

MGFVRNFWKKLTHTKALDQSSGRLETVFFESIYRITYVAGMSSTDHDMFYLMYSNTVKLAIVLLVCGEIWYGFTEASGLDEVAASINVTVIQYIAIYRFMNMMSHKDFYKKLATSMESPYFDITTEERKKLVDYWWRTNERYLKLLLALGNCTLAFWFIFPLVDDVDYNLIVGIRLPVNYKTPFRYPLAYIVVMIAFFYISHFVMITDLKMQTHLLHLLCQFTVLVDCFQNLLRDCRIGFEDVAESNLVYEKRFADKYTKRLGDLVEQHKLILSHTMNLRDTLSSPMLGQLAASGILICFIGYQATTTIAESLFQGLMSAFFLGYNLFGFYIICRWGEEITNQSEKIGEAIYSSGWECGLAKLPGVRSTIMYVIARANKPLVLTAGGMYNLSLTSYTSLVKTSYSALTVLLQFRHE

>HassOR60

MGVLVRNATMSVSISLTALQFVGFWAPEYLGKMQKQLYTCLSVFSFMFLLGTYLIIQVVDLFLIWGDIAMMTSTAFLLFTNMAQAAKIVNIVYRKERIQKIVNDCDAVLSQAQSLEEKEIVKSCNREMIVLQILYFSLTLITSLGWATSAEPHQLPLRAWYPYDTTKSPAYELTYVHQVGALLIAAYLNVAKDTLVAALIAQCRCRLRLLGYALRTLDKGMGNEAYTFTSDQEKTLNLRLGSCVVQHQQALDVGKELQECFSEPTFAQLTVSLIIICATAFQLSMGHSDNMVRLLSMGTYLLNMTFQVFIYCYQGNQLSEESSEIAGAAYECPWYKCSLRVRRGLLIMMVRTRRALRLTAGGFTTLSLASFMAIIKASYSLFTLLQQVIEE

>HassOR61

MRKQIIFVVSTVYVIIGIANYIRDAKDIALISEALYGMLVTSTVLIKYLLFMNKRKSLQRLYLTAKTEILEIIKTSGDKSKELLSKMRMIVKIYFGSIAVPVTTYFLAAIWNYMRGLRVNYSKGFTTLMPSRSPYHEIGLMLHSTIASVLGFTYYIMDVWFFILISFYCLTNDSLVNILKLERNEATEMVFMDQLRDALKTYYRNHVMLIEFLNTLSDMYKWLTIIPLVSVIITFCLMMLSMTVQTQWIFLTNAVAPVLQTFAYNWFGEQVKVKKSQLDMALLKFDWVSMRQKDKRNYLIIMSYMNKEFAIKTALGYDLSLVTMTAILKASYQAYTLLRTTET

>HassOR62

MADKSYDSLKENFLHEMDFISDLGVKMFIYPFIGRSRLATYCYHITYGLLFFTMTQLIITLTLICINDIDVFEIINVAPNIGVCLMILIKYGKIHDNKLLYDQINKHFRFDIWEAILDTPAHKEILHNSTWMTKIILRFEFYYTIGLVIVIDLFPRLIMVYQNNILGKEKQYLYPFDGWYPFDKIQWYDAAYIWESFMTTVVIFVFVYVNMLHMSYTRFICLELKILGSSMEGLISNEEVVKIKKRKDVDRIHGNIRQKLKFIICKHQFLDRIVSDLDEVLGDGMFLTYLFGSVFICLTIFTATVVDDLYKSMRYFSFFCSLLVEVFIQCIIGQLLIDHSNKLERAIYFADWVYADSDTKKMLLIFLMRSQKPFGLSAKGYLTMNLDTFSGVCSLSYQFFNLLRTAYSE

>HassOR63

MEVLKHFPEDFSQELQTSFFILSFFNIRYLQDELSFIQRNWRFSYLVLIVIIHVVTLGLHLPELVTGDEMSQFAYLIPSILVTVHAIFKCFVLLPKTKEVSIFLRELGSLWRVKFTVTQKQEKDKLLWRLHLVNRVSFWISIFGTSQYMLSPLAETLIRKFILQQDSKLILPFDSVYPFNPTKNWLIYIFTYIFQFYSLFLLVTVYSGASLIMVTSCGLLTTEFIMLKDDLTRVKPRMKRGRNKESKNDDHEPTIEQFVERHQKLLRLSRLLDNAFNGMVFIDLLFVGITVCAFGFMGQFTRSAMYKLVSYLGVVSSLLTVLYLCYYGEQLTSASSTIGKMAYENLWYKGEKQYKMTIWLIIKIGQNPCRLTSLRYADVSLNMFTSVVSSTWSYFSLMNSVYSEEEV

>HassOR64

MRTLFYDTIKRRSNTIGERNEFDEAVSLAIFIQDIFGQNILHPKWSLKTKIVYQFFVVFLVTHTVLGTMERLEDNPSNICECYFVICIISLYLFKYLLFINSRRNFQKCYLLSKTHLLEIIKLQSMEISKEMMKRIKMMRDILFFCTFVPLTAYFLTEMVYYFLGQRQGLSSSTSTLMPMTSPYYELGMALQLSYFIVASYTTYVLDMWYVVLIFVFCTACDSLVAMLKVKQDEDETELEYKDRLNNTLKTFYENHVKLMEFFNIFKELYLWPTLVPLLSGLVLFCFVMISISGAQNIEWKFVSLVVPATFQIYGYNWYGEQVKNKGSNFSMALLEYDWMIMHHKDIKNYIIILGYLNKDFSIKTAFGMEMSLVTMTSVLKASYQAFTLLRTVES

>HassOR65

MWKKFKAFYNKENYDFTKGNIDGYEFHKTFYQIMVAFKVADLSNPNPPSYFNQNLVIFISGFSAITFTCTSMFHGFESFDIPLITEAGTYTIVLFYELLIVSCTWRYLPQFHHLLRAIKDDFHYICTTGEKYRVQYFQAQLKTWKICITMCIFTVTISVGMITFATLSLVYFLVTYDPEVGGSRPLLFPFWMPNVDFGASPVYEMAFMFANVNVIIYAYNYIFMIQTQIVWIRQITSKVDIVNWSIQDLLVDIRPATSEDESMYYNYLIQTRMRDIVIHNQAMYRLMEDYAIVYKKLLMFEQKCCGPVVCLTSYCLASKFDEGEFQAILLLLCIATIVLHFIPSYLCTFLAFKVSSVCDACWSIPFWNAGPVIRTYMVLIMQRSLRPLPLQAAGFEDISIETFSKKMTNAYSMFNMLRQTNL

>HassOR66

MEEFHNLPEEYRENIEHSLNLLTNGNFLIFHKPNTFFEKRFRPYVVICSVVTYLASLTMYLGKVFQGELQLTELAYVVSVYMVSIQAILKAAIAVLNKDEIRSIILELGRLWRTQDLTEEQINKKNAQLKRVKFCYAVVYWINMIGSWQYILAPLLETVFRKFILHEECGLLLPFSCSFPFDPSGSWGRYIGVYIFETYSMFRIVYFYLGVEFLMISLCSNLATEFTLLQDDLRSVKPEHNNIALKDIIATHQKLISLSQQLDNVFDKVIFVNLTSTSIPLCFFGFSAKVAHGVLQMVNNFAAVISLILPLFNMCYFGEQIREASAGISDSVYHNLWYRGDVRFQKILCFVQRRSQKPCCMTSYKFSPIALTTFTTVLSTTWSYFSLASSLYEGEN

>HassOR68

MAQTARGGSFQDAIEIFCMMPCVGYLLLAMAKSYKIVYHRSVYENLIEELRSMWPRGEVSDEEHQILSTALRHLNYVIQGYYWCNNALLVIFLSPPFVEIIKISLGHKVPLIFPFFYWFPFDPFQRGYYEVILAAQTWHGLITIWFMLCGDLLFCIFLSHITTQFDLLAVRVRRLVYVPVDKQLVDSYPLGEYCKDYASRNKEIINIFDDNDWEVKHQRELSDIIVRHRALIRLSGDVEDMFSFALLVNFFNSSIIICFCGFCCVIVEKWNEFVYKSFLTTALSQTWLLCWYGQRLLESSEGLSDALYESGWYRASKSVKSSILIMLHRAQKDVHVTTYGFSIISLASYTTIIKTSWSYFTLLLNIYKK

>HassOR69

MKTLNEICNRHSNTLGRRNELDEIMAVPIVCQDILGQNTLNPKWSLRATIVKQMIFFCLCIYTVLCVIDFLEEKNASSKSQAYYAVTFICLIVIKNLIFIKNRKYFQKSYLIAKTDLLEIVRSTSLSNFLELLRRIELMINILKACIILPLAIYLLKASWHYILGSRVNMSSSSSTLMPMTSPYYEFGLILQTTIYFIMTITCFVIDLWFIVFVVIFCKASDSLVSILKVGHEESVETEMEYMERLNNTLRIFYGHHIKLVEFLNVLNNMYKWLAVIPQLSVFVTFCVILFSMAEDNQWHFLLTHAMQAMFQLFSYNWFGEQVMFEVSELTRALLEFDWASMRGKDRKNYLIIIGYMNKEFRIKTALGNDLSLLTMSSILKASYQACALLRTMDI

>HassOR70

METTTYTRSKTTDFFYKINFMIYLFGLPNFWIEDLKLSKTFVKIYDKFSAFSNGLIYLMILSEIGSFFTQTNLTEKQMSNLMVFVISHPMLCSFNVMMSRLKTKIRLVMYSLAVSLKREYNDPELEKHMIAHSLTYVSGLSVSYVMTMIMFAVNAFLEMIQNGSTFTTIITAYPLVDDRSGLADVARAFFFVTWWIFVTRFLAVYMLVVPLTTSLGYQFKNLQSYFSSLADIFDSKELSQTEKEEKYEAGFKVGIKVHTETLRCVKDTQAVCRSVFSGQIILNIVLLIILMAQMVTSERTLVNIFGTVITIGAVLVTTGLYMWNAGDVTVEASRLPTAIYFSGWYNCRGQSLVRLRNLIVITMASAQRPVVLKGLGYIDLSYQSYITIVKSSYSVFSVLY

>HassOR67

MSESTIEQAKREIDESLVLSAFCMRRIGLSFQEPKTGSAYLRQKLMLIVSVCGICYHVFSEIVFIGLTLSNSPRVEDVVPLFHTFGYGALSIAKVFVLWYKKDVFRQLLNELAGIWPMPPLEEEAQTIKNKSLAALRITHRWYFFINVSGVWFYNLTPIVVYLFGLIQNKDDTIGYVWSSWYPFDKHQTLAHIAVYLFEIFAGQTCVWIMVSTDLLFSAMASHISILLRLLKRRLESVGTGDDDHYQEIVGNIKLHQRLITYCNDLENAFSLSNFVNIMLSSVNICCVVFVIVLLEPLMAISNKLFLGSALIQIGMLCWYADDIFHANADVAAAAYNSGWYRTNPRCRRALLFLIKRAQKPIAFTAMGFTNITLVTYSAILTRSYSYFALLYTMYSDD

>BmorOR1

MLLSFKDDSRSPDIQKPQNFQYMKILRFNLKIICAWPEKQLNEIRSLGHSIHRVILPIQSVVCLACGILYIHFHFNEIPFFILASTFITVMMNLVTCSRTALVMLFERYLVLTGRFITVMHLFNFQKNSDYAYKLCTFVNRMSHFYTLYVLFSMFMGLGLFNLLPLYNNYVSGAFSDPYGPNVTFFHSVYFAFPFDYSHNFRGYIIMALFNSYVSVTCSIGLVMFDLLMCLMVMHVWGHLKILSHNLINFPRPKASHVITTPNGPTNVETYTEEESKEVFARLRECIKHYGTVDDFANDMSETFGVILLVYYGFHQVSLCMLLLECSDLSTKAMLRYGPLTLIMIQQLIQISIIFELLGSVADRIPDAVYQLPWECMDVKNRRVVYGFLRRTQNPVRFKAMGMLDVGVQTMASILKTSISYFVMLRTVAT

>BmorOrco

MMTKVKTQGLVTDLMPCIRLLQAAGHFLFNYHADTSGMNMLLRKIYSSAHAVLIVVHYICMGINMAQYKDEVNELTANTITVLFFAHSIIKLAFFAFNSKSFYRTLAVWNQSNSHPLFTESDARYHQISLSKMRRLLYFICGMTVFSVISWVTLTFFGESVRMIASKETNETLTEPAPRLPLKAWYPFKTMSGGGYVFAFIYQIYFLLFSMALANLLDVIFCSWLIFACEQLQHLKAIMKPLMELSAALDTYRPNTAELFRVSSTDKTEKVPDAVDMDIRGIYSTQQDFGMTLRGAGGKLQNFNAENNPNGLTAKQEMLARSAIKYWVERHKHVVRLVASIGDTYGTALLFHMLVSTITLTLLAYQATKINGINVYAFSTIGYLVYTLGQVFHFCIFGNRLIEESSSVMEAAYSCQWYDGSEEAKTFVQIVCQQCQKAMTISGAKFFNVSLDLFASVLGAVVTYFMVLIQLK

>BmorOR3

MIFVDDAVIGIKDPREYRHLRVLRTSLRLLGAWPGHYLGEETGSKYECAPMFLLMFIKIACLYLTIVYLRNNADVLGFFELGHVYLTIFMTFVTLSRGFSLTWNPNYHKVVKKFITEMHLLYFKDNSEYAMKTHRRVHKISHFYTVFLKVQMIAGLTLFNVIPMYNNYRQGNYASDRPANITYDLSIYYETFDILNTPNGYIFICVFNWFASYICCSFFCSFDLILSLMISTVSGHFRILIHNLLTFPLPEAITASKKFVDKHRCNGNRSEFVLEEAKLYSPAEMWQVTDRLRQCIDYHRKLVEFTGDISEAFGPMLFVYYLFHQVSGCLLLLECSQLNTAALVRYGVLTVVLYQQLIQLSVIVESVGTVTGRLKDAVYEVPWEYMDTSNRKTVAIFLMNVQEPLHVNALGLAKVGVQSMAAILKTSFSYFTFLRTVSE

>BmorOR4

MFKIIKNIIVENDALKQVEKPQEFQYMKWVQYHLKYIDGWPNMDMNKKNVSKIRFHKRHLLVVEQTITFLSQMFYIVKNYGKLSFFEIGHSYITALMTIVIFSRSVVTALGRYRKIARYFVSSLHLYHYKDISEYALQTHLLVHRLSHYYTVYLISLVVTGMLLFNITPLYNNISSGVFNSPRPENMTFQHAVYLGLPFDYTTDIKGYFVVFILNWHLSHIAASYFCTFDLFLSLLILHLWGHLRIILNNLKTFPKPYTNNSMYTEEENQVVLLKLQECIRYHNFIISFTVMMSNVYDVVIIVYYLFHQVTGCLLLLQCSTLDWESLSRYGPLTLIIFQQLIQVSMIFEILGFLSDKLPNAVYSIPWEAMNVTNRKLVQVLLQKSQKPIQFKAMNMMSVGVQTMASIIKTSISYFIMLRTIARD

>BmorOR5

MLLYYPNTQVKEKVNNVEEFTYIKFLKSFCKIMDFWPEREEKNSKTRIFRLRYILVLQFCFTLVAGVLYLTNSVGKQTFYDLGHTIITVLMNVVSLSRLILRCFKKYDVVGQQFINKIHLYHYRNDSEYAMKIHTVVHKISHNMTYIFSFCIIFGTVTFNLTPIFNNIGSDAYKNPRPDNVTLQQCVYYALPFDYTGNFKWYLLVAIFNVQKTFFCTSLFILFELSLSLMIICLWGHLRIFIHNLNHIPAPRNSFEYTKEERQEVDDTLKKCIQHHTLIIGFVRIMSETYGLAVLIYYAFQQVVGCLLLLQCSQMELKTVTRFGFLTLVLNQQLIQISVIFELLGYMSDKLQDAVYCVPWEYMDTSHRKMVYMMFRQSQIPLQLKAMNMLSIGVKTMVSILKTSVTYYLILKTVTTD

>BmorOR6

MKEEYYLQHPRTQLFYKVLAHVSTIESTIDLTWWGYTFPKYVGWFYHLQCNVVRLFGKCVVVSQILFIILNYQTIDKSVFIIAITITPLGALVGIKAESAKAECYVNLMKNFMDKVHIHSIYRKNENNEFVKKKVIQIERVSRFTAYFLVILIAINCLSWMLKPTLHNIKHFEEIMNKSMEFQYYIYFWTPLDYKYNLRDYIIIHTLCIYLGATAVTVIVTFDIFNFIAVFHVVAHIQILKNNVKSNWSDDFNESEKKGYLVSILEYHAYIIRIFGEVQSAFGLNVASNYLQNLIEDGLFLYQIMNGEKENVLMYGLMIILYLGGLIFLSIVLEEIRRQNYDLCEYVYALPWEGMSLENQKIFVVFLQRTQPDLEFETVCGMKAGVKPAFSIVKSMFSYYVMINSRF

>BmorOR7

MLLYHPNTQVEEKVNNVEEFTYMKFLKSFCKIMDFWPEREEKNSKTRIFRLRYILVLQFCFTLVAGVLYLKNNFGKKTFYDLGHTIITVVMNVVSVSRLILRCFKKYDVVGQQFINKIHLYHFRNDSEYSMKTYKAVHKISNNMTYIFSFSIFVCVVTFNLNPVFNNIGSGAYKNPRPDNVTLQQCVYYALPFDYTGDFKWYMLVAIFNVQKTFFCTSLFILFDLLLSMMIIHLWGHIRIFIHNLNHIPAPRNSLEYTREERQEVDNTLKKCIQHHTLIIGFVRIMSETYGLAVLIYYAFQQVVGCLLLLQCSRLDLKTITRFGFLTTMVNQQLIQISVIFELLGYMNDKLQEAVYCVPWEYMDTSHRKMVYMMFRQSQIPLQLKAMNMLSIGVKTMASILKTSVTYYLMLKTITANEA

>BmorOR8

MSLSTRCLLKDFCKYVYYAGAGNFWYEDIYKETVPYKMYVVISFFTYTVMIFLENLAALFGKLPEVEKNSAVMFAAIHNIVLTKMFLLLYHKRSISKLNCEMAAVGENLEEASIMRRQFRKMRLGTALYFISVYLSLVAYGVESARRTIVEGAPFYTVVTYLPDYDNTTVLASFLRIFFYITWLYMMLPMMSADCMPIAHLITMTYKFVTLCRHFDQIREKFQINVKIMAKTEATEILKLGFIEGIKMHQKLMYLADEIHRVFGIIMALQVCESSAVAVLLLLRLALSPHLDLTNAFMTYTFVCSLFLLLALNLWNAGELTYQASLLSNAMFYSGWYFCDFEKDWCRDIRRLVLIGCAQAQKPLILKAFGVLDLSYETFVSVARMTYSVFAVFYKRGD

>BmorOR9

MVARRPLQFHQGRNVDNVEDFKYVKWLRNHLKTVDAWPVHSKSKRKIQKRYVLPIFSAACFISQTVYLKNGIGTLSFVVLVHSYICFLINGSCLCRGILIATERYKRLATCYLKTVHLFHHKNRSEHAMKIHVIVHRLSHYYTIYLISLVFVGMVLFNFMPIYNNINSGAFKSPRPENVTFQHAMYLALPFDYTTNIKGYFVVFILNWYISLVTTSHFCTFDLFISLMIIHLWGHIKILMCSLEDIEGFVPGSSFKFTIEQNRKIYLILQECIRHHQFTIDFTNEMSSTFGLVILFYYFFYQVSGCLLLLACSQMDIESLSRFGPMTFILFQQLIQLSIVFELISSLSENLPNAVYNVPWEFMDKNNRKMIQVLLLQSQKLIQFKATSMMNVGVQAMATILKTSVSYFIMLRTMYQEH

>BmorOR10

MRTNAKSFLFVPSKVLTLCGVWPVEKTSIFSLIYRSIMLSSQFCFLVFNGIYIGLMWGDLKAVSDALYMFFTQTTCCSKAIGFYFNFMKIKRIVASMDDVLFTAMSIEDQATIFSHSRTVNKLYKGVLGFTGFTLVQWTVLSLIGSGRTLPFNEMWVPTDISKSPNYEITFVVELWMMVISAALFMSVDTITVATMMFSCAQLDIIMKKTQQIQEIPLSPDLSSRNRSELHEKNNGILIDCIKQHQAIVRFSELCEGTFQVHSFFHLGGIVFMICVIGFRMAGESPVSAQFWAALSYLVIILGQLYLYCWCANELTTKSEQLRDKLYLTPWYDQDVKFKRNLCIAMECMAKALTFRAGSYIPLSRAMFVSILRSSYSYFAFLNQANEQ

>BmorOR11

MDEHSHFETSLNKIKVLFKYSGMNLENTVTNTYEFLNHRWVYILNHAWTLAAVTFICIGISNGQNFIEMTCIAPCVAMTVLAVSKSFFHYINENAVKSLLENLIELERTDFERTKSVQRTEIVATEKQLLNMVINVLYVLNCSMILVFDMTPLIIIAIKYWTTNKFVRLLPYLDIFVFVPYKFEYWVMAYILQIWAECIVLLFIGAADCLFFTCCTYIRIHFRLLQYDFERLTSSRRESDGLRDDEDFRETYTNLVKRHQGLIESSSILEMIYSKSTLSNFVLSSLVICLSAFNVTVVNDVTIVMTYLIFLAMSLMQVYFLCFFDMLMSASEEVGNAVYNCSWYTEKASTGKDLLFTITRAQKPCELTAAHFAYVNLKAFMRVSFTSASITTLPTI

>BmorOR12

MTRITDVFSLNFIFWKFLGLWGKSAPSKYNMAYTVFYLFASLFVYDIFLTLNLIHTPRKLETLVRETMFYFNHLVAVTKILMMFIMRKKILVIFDLLDCEEFKPNDENSQEIMKRKTDFYYIYWRIVAVTSNLSCFMLVIGPLIKMLIWKIELGLPVCKFYFMSDELRNKYFVIWYIYQSFGIYNQMVNNLNLDTFNCGMLWMAVGQLQILKTKFVNLKLNDFENGLDLKSRDDMQIERLRKYLTHYEIILKYCAIVQDILNITIFVQLGMSSIVICVGLCGFVAMPSNTETAIFMFSYLTTMTMQIFVPSWMGTQISFECGELMSAAYSCEWIPRSKLFKRSLILFVERAKTPVRITGLKIFTLSLDTFTSIMKTTYSFFTLIRQLQVDEVN

>BmorOR13

MAPKQIDCFEINWKFWKFLGIWSENKPHRYYKYYSKIFITFFVILYDVLYTINFYFVPRQLDLIIGEMLFYLTELSVLSKVFTFIIMRHKLKIIFEILESDAFQTDTEEELKILHRAKVFIKRYWKIVALVSITANLTHISSPLLKNLIFKVELVLPVCSYSFLSESFLKTFEYPLYFYQIVGIHFHMLYNLNIDTYFLGLMILIIAQLDILNVKFRNLKSGKDHTQLNESIMGLNKNLDHYNEIERFCSLVQNIFSFTLFVQFSMASCIICVCLFSFTLSVPVEYYIFLATYMFIMIIQIMVPCWFGSRIMDKSILLSSAIYNCDWTSNSKDFKINMRLFVERANKPLSITGGKMFSLSLATFTSIMNSAYSFFTLLRYIQTRE

>BmorOR14

MSNYIFKPFHETYRIITFTMIAAMIYPNPATEKRRLIYIGLMLLSVIPLAFMIVTEMYEFFMASDLNNTIRHSTVIGPFIGGFVKVALMYYKRRQANELVSEINRDHLAYNGLKGEDREIAASSIRNCQIYCELGWTLIVMSCGLSFPVIAILLKIHSFTFKLDSTKHMIHDINNPFTDDPEDRFESPFFEIMFVYTFFSSFIYIINYVGYDGFFGLCINHACLKMKLYCRALEDAMRSDSRRHEKIVAVIEEQRRTYEYIALIQDTFNIWLGLIYVATMIQMCTCMYHIVQSFNIDVRYIIFVISIIHIYLPCRYAANLKCMAAETPTLIYCCGWESVSDLRIKRMMPFMVARSQVIVEITAFNMFAFDMELFVWIMKTSYSMFTLMRS

>BmorOR15

MMTLVYQTDIFKPNVFFWKMFGIWADRKSSKTYKYYSFVFLFITLIMYNSLLAINLLYTPLKIELLIREVIFCFTEITVTTKVLMILFKRNKILDAFDLLNKNEFRGNSEESSAIIQKNNSAYKTYWKLYAILSNFAYSSQVLGPLIVKLIWKTKLELPICNYYFLNEELRHDFFSGWYIYQSFGMYGHMMYNVNIDTFISGLLMMAVTQLKIIQTKLLSLKLNPRERKMDRGLMNITEVLKLNEILKHYELVLKYCSTVQSILDVAMFVQFGVASAIICVAMCGLIMVRSSTETLLFMVTYLFAMTLQIFVPAWMGTQLHFQSQELVFAAYNSEWIPRCQSFKRSIIIFVERAKIPITITGLKMFPLSLATFTSIMKTAYSFFTLIRNMQTLQEE

>BmorOR16

MSFNSEDLYLNRAKFVMKYLGVWVPPENENFARKFYKIFMMSLQHLFLFFQIIYIVEVWGDLEAVSQASYLLFTQACLCFKITVFQINMNKLKELLKQMNGYVFQPKNINQQNIIKVQATRIKRLLFAFMISSQLTCGMWALKPLFDDVGSRKFPFDMWMPVSPERSPHYHLGYSFQLVTICMSAYMYFGVDSVAFSSVIFGCAQIGVIKDKIMSIKPLGIYRNHKTYTKISRYNRKTLIECVKHHQAVISFTELVEDTYNSYLLFQLVGSVGIICMSALRILVVDWRSVQFFSILCYLSVMISQLFVCCWCGHELSATSEELHTILYNCAWYDQDVKFKRDLNFMMARARRPILLRAGYYISLSRQSFVSILRMSYSYFAVLDQTNK

>BmorOR17

MREDKMEINNSQKFYTKMIFRYLYSVGLGDWWYQHEDRSDSHRKLYCLWAVISNAYIFLNICNELLANFRKDLTDVEKNDAIQFSFAHPLIFAKIASFFFNRKKIREVFGRLLEENRSVYSCGELEKESMKQIKRYSLAFIGVSYMTLVMSTIDGLRAHFKEGIPIRTEVTYYPSPSNSGVIVNILRFLVEFHWWYIVSVMVAIDSLAVASFVFVTFKFKLLQRYFKDMGLTVRRDQSNMTDEALADKFRRDFIVGVKLHENALWCAENVQKAFGWVYSVQVFETVALLVMCLVKLVTTNHNMIFLLANFAFMLCVIILNGSYMMPAGDVTYEASEVPTSIFLCGWELVRQTDLRFLVVVAIQRSQVPVIMKAFGIMTLSYSNFIAVSLFKFYVQFQINLF

>BmorOR18

MGDRMVTRGHFFDFNIKYLFYVGLWPSNEAKRIEKIAYKIYEYQLHVLSLIFLVTTGIGTYKNHKDIIALLTNLDKTLVAYNFVFKVIVFVYKREELRKLIEQIVQSGDQITEDRKALMAKLVIVLTGISTVIITAFSCLALFEGEMTIDAWMPFDPMKSKMNLFAASQILAATFVVPCGYRAFAMLGIVCSLILYLRDQLVDLQNKIRDLRFATGNVEKLRDDFKLIVKKHVRLLGYSKVIEMIFKEYFFIQNMAVTAELCLNAMMVSVVGLEQKTLAASFLAFLSVALLNAYIYCYLGNELIVQSEGIAMAAYESSWILWPVDMQKDLLIVITAAQKPMKLSAGGMAVLSVQTYSQTLYNGYSIFAVLNDIVN

>BmorOR19

MHEFVINVQNETTKLYDQLNIILYILGLQGIWVDEIKLSRRFHVFFKVVTFILHIMCGMFAGLQFFAIFTQNSLNSQQKSDVIVIGISNPMAYIFCINFIRNRNEIKDLFYHLAVVLKIYYNDVEIEKSMVNKIKSYLSTYVFASITILVSNGIIAFFQTINSDEPFLGIITAWPDKTDTSKTASYARIGFYLFWCIHFFRISTVFAVIVCILISIKYQYKFLCSYFESLNKIFDDETSSHEVKEAEFENAFCNGIKIHTQIIWCVRRCQIMCRTVFSANIMLDTFVLVILMLAMVNSENDFYGLCSQMSSVLVTVVLMAFFMWTAGDINVQASQLPDAIYGSGWYNCRGKSSARIRSLVTISMNKAQQPILMWALGFVELSHKNFVAIIKSAYSVFSVFY

>BmorOR20

MIQASKYPNSKTKELFRKIAHIAYICGLPNFWIEELNLPKSFIRVYDKIVRIFNVATYFFLGIEIAAHFTQHHLTNKQKFDLLLYSISHPILNGYGVIVSRQVGNVKKVLLDLIVNLKVKYNDPVIEEAMIKISMTYSVSFITNCVLSMLTYTFDALLMVYKKGVTFNVIITAWPDVEDTTTEASIGRIGFHIFWWLFVTRPFAVYVLVINLTTCLSHQYMNLQSYFFHLEDIFKENLSQNEKEAKYEAEYKIGVMLHANTLRCTRRCHMVWNGVMSGQIIFNISLIVIIMAQMMNSDRTLVNTFGTVLTASAILISTGFFMWNAGDVTVQASRLATAMYCSGWQNCRGKSSVSIRNMVMNTIAVAQRPLVLRGLGVIDLSYQSYLSIVKASYTVFSVIY

>BmorOR21

MNKNHYILKTYCDKIFLVGSGNFWYQKTESRNDKTLLYKIYSCVLFFTYGFMTVLEIMAAMMGDFPEDEKRDSVTFATSHTVVMIKFISIIKNKELLKTLNRKMMMICEAHEEQTLMDEMYRTVKINVVAYCVAVYGSATFYVFEGLRKFYNGSHFVTIVTYYPSNDDDTLAATIVRIATTLVLLMMLLTMIISVDTYTMAYLIMYKYKFITLRHYFKRLRENVDELVAAGKARLAAEKLAQGLVEGIKMHNELLSLSKDIDKAFGTVMALQLCQSSGSAVSLLLQIALSDQLTFTMGMKIFFFLAAMYLLLALFLCNAGEITYQVCTSIV

>BmorOR22

MNKNHYILKTYCDKIFLVGSGNFWHQKTESRNDKTLLYKIYSCVLFFTYGFMTVLEIMAATMGDFPEDEKRDSVTFATSHTVVMIKFISIIKNKELLKTLNRKMMMICEAHEEQTLMDEMYRTVKINVVAYCVAVYGSATFYVFEGLRKFYNGSHFVTIVTYYPSNDDDTMLASIVRIATTLVLLMMLLSMIISVDTYTMAYLIMYKYKFITLRHYFKRLRENVDELVAAGKARLAAEKLAQGLVEGIKMHNELLSLSKDIHKAFGTVMALQLCQSSGSAVSLLLQIALSDQLTFTMGMKIFFFLAAMYLLLALFLCNAGEITYQASLLSDEIFYCGWHKCNSPVLSTQRNIRDIVLIAILRAQSPLVMKAFKMVELTYATFILVVRSTYSVFALFYAQNK

>BmorOR23

MRAKTEFEKTIKLTKTALFLSGINIFLGEWNHWTRTFVDSIAYYLNIVGLYFVLIGEMYWLIDGTITGKSFVELSLIVPCLTISVLATAKVHYLYHNKESLLDVVDKLREIYPDEIEETANDNDQCLNDKKETVYDNDVTEVGIVNEANELLKFVNFLLSTVSFVVTMTFCTMPLFGMAGEFMETGKFVVLYPFAVKYPFDVYNTSFWVIVYVNQFWATIIVCTNIFGVDTLFYALCSYIGMNFRLLSYKFEHLEIKRNDRIINEIIVLIKRHQELIELVNKTQSLYSLSTLFNIVTSSLLICLSGFNITILSRSWSYFALLKTIYS

>BmorOR24

MPEELFLDRSIKKIESYFRWMGINIRSGDNNNKKDVFKIRCIYFINFVLLNTDVLGAIFWFRSGLEQGKTFTEVTYNAPCLTFSFLANFKMLSLIFYEKTVHELIAALQKLEIKHFLRQNCAEELKMLKDEKNFLHAVFKGSKIVNYASILTFGCSPLVLIASNYYKTGRMDYLLPLIVLYPFDVDNITVWPIIYVRQIWSVITAVIGVCATDYLFYTFCVYISTQFRLLGHSIERVVPNNGLSVRTRLNGNLRMKFVENLKWHQELIRAASLLEQIYTKSTLYNFVTSSVIICLTGFNVAVVEDFAVILSFLFFLFMSLLQIILLCFFGDKLMKSSTNISDAVYNSKWYLTEKNVGKVLLMVQIRSQRACRLTAYGFAEVNLRAFMKILSTAWSYFALLQSLYSSHE

>BmorOR25

MFEKALRSANFYMRVIGIPTDIRDGNRTLMERLRNRWFYCINFLWLNTDVAGEITWFVKGLLSGSSTLIENTYLIPCLTLCILGNVKTFFTIKYANHIIDLVAILKDLEIKNNAARKNETEIVKERLKFLTTSNKFLLFVIGTGIIAFGIGPLMLTASIYFSSGDMKLKLPFLIWYPFDSSDIRYWPFVYVHQVWSACIACCAVYGPDCFYFTSCTFIHIHFIHLQNDITNVIVESSRARRNGLYRGCHQAFLELTNRHKDLIRCVNLLEIIYSKSTLVNVVSSSLLICVTGFNVMAIDFLPLIAPFTSFLALGLVQTYLLCYYGDTIMCSSTEVSDAVYNSTWYGTNISQMRDYLFVMKRAQKPCKLTAYGFSDVNLRTFSRILSTAWSYFALLITIYRGNGQQ

>BmorOR26

SLSGSSVFTHLFLLRCCGFCRLSRSSTARRGLSVAHEVYRALTLTLTVVYLLQECVYAYQERTDMDKLSRVMFLLLCHITSVAKQLVFYLDADRIDYLIATLDDPSYNEMSHQRLLVDASRWASRFVWAYSGCAVVTCTLWIVFPIIYHVQGQTVEFPFWIQIDYTKSSMFVVVLLYSYYVTTLVGIANTTMDAFMATILGQCKTQFTILRIKFETLPTRAKQALRCDSEQNYDEVLMRLFHDCLKHYQKIVSAILIQFGIGAWILCMAAYKIVNLSVLSIEFASMILFISCILTELFLYCYYGNEVSTESERLVTSIYSMEWVGARLGFQRGLLVLLERARRPVRPAAGLVIPLSLQTFLKIIKSSYTFYAVLRQTK

>BmorOR27

MPSSFFLPNLENPDYPSLGPTLKGLKYWGMWQSGGIKRILYNSIHAFATFFVITQYVELWIIRNNVELALRNLSVTMLSTVCVVKAGTFVCWQKYWSGIIGFVSNLEKEQLSKNDAATQAAIVKYIKYSRRVTYFYWSLVTATVFTVILAPLVGFLSSPERELIANGTLPYPEIMSSWVPFDRSRGFGYWVTALVHTLICFYGGGVVANYDSNAVVLMSFFAGQMKLLSINCSRLFDDGNEVISNNEAMKRIKECHYHHVFSTIFNSLMSPVLFLYVIICSLMLCASAVQLTTDGTSNMQRIWISEYLMALIAQLFLYCWHSNQVLYMALEDRLGGLFEACLESGRFPSKWKTGRLVLLRKDGRPADSPAGYRPIVLLDEAGKMLERIVAARIVRHLTETAPDLSAE

>BmorOR28

MHTLALVFALLYPSNCNIIKRAIGITLIIALSGGQLFWCMTYTFNVCVLILNYSGFDGSFCIASIRLCMKLKLVVYKVQKAFAESKSVSELKHQLNDAIKDNLDALKFHEQIQNVFFIALVGRRAYGPPDGEWLPSPMDFSNTRGRTKPLSTVYEPWLFLIFLLTFLII

>BmorOR29

MFDFLQNLEDSERPLLGPNFWLINKTGLLLPKTNFGKLAYILVHEIVTFFVVTQYVELYVIRSDLDLVLTNLKISMLSIVCIVKVNTFVFWQTSWREVLEYVNEADKFERNQTDETRGKMIETYTKYCRRLTYFYWSLVFTTFLTTTNTPLMRYWSSPIFRENLRNGTEDFPHIFSSWMPFDKNHSPGSYCTIVWHVLLCAYGAAIMAAYDTCIVVIMVFFGEKLNLLRERCKKMLANDLYNHAFVIGQLHDIHVQLIKQSRLFNSLLSPVMFLYILMCSLMLCASAYQLTSATSTAQKLLMAEYLIFGIAQLFVFCWHGNDVLFKNANVSLGPYESNWWSSSPRVRADVLLLCGQLRVRHVFTAGPFADLTLSTFIKILKGAYSYYTLLRK

>BmorOR30

MSVSNLKFEVLFKPTTMSLHMNRSHPSIKRNKIWLLQFISLMTLTAFCATGLITSLLFHDLKFGKYMEASKNGTIAMLSFTTTFKYSLLLYLQKSLNRLIAKIDMDYEIAKGLTPQEKVTVLNYAKKGVIVSKFWLFTAFAITFCFPLKAFIIMGYRFIIKDEFRLEPMFDMTYPEPIESYKTSFPVYFILFVVCFLFGCYASSLYVAFDPLVPIFVLHACGQLDLLSVRITKLFSDTKNPRIIAKELKVIIIKLQELYSFVNFIKVNFSILYEYNMKITTISMPLSAFQVVESLRRGEFNIEFTYFFFGCILHFFMPCYYSNLLMERSENFRFAIYSCGWENHNDKNIRQMLLFMLTRAAEPLGIATVFTNISLDTFAEVNTFDTVLLA

>BmorOR32

MTTRHAEPCAEAPRLSPAAGGMVGLAPVPQPSSNEMLVQERGRPPGLEGEYVANRPFRKSLRGSQEEPRANGKSENVRFLINSHILHCGLRFNETNCHTHYIAKVAIFCFIVTYMLQVMELYWSKGDQEKLFECFSILSFCGMGVMKLVILRVYHQRWRFLLNQVSILENRHLDPGPLSYDSDNDNDDNEIVTFITKYTDKFKRTSSILIKMYASTLVIYVLSPFVEYIFRQFRGDLNIAYPHILPAWTPLDEFSVTGYLIMVSFETVACIYCVFVHVAFDLTCVGLMIFACGQFYLLRYRSERIGGKGRICRLLKSTEVRAHYRIVFCHGIHVLLVQLIEELDRLIKHILGVYFFLATLTLCSVAVRLKTEDMSITQLVNLLQYMCGTLTQLFLYCKYGDSVYNEADEPYGLPDACLESGRFPKQWKTGRLVLLRKERRPADSPAGYRPIVLLDEAGKLLERVVAARIVQHLTGVGPDLSAEQFGFREGRSTIDAVMRVRALSDEAVGRGGVALAVSLDIANAFNTLSWSVIAGALQYHGVPAYLRRLIGSYLEDRSVVCTGHGGTVLRFPVQRGVPQGSVL

>BmorOR33

MELNFDKIFKIAIISQKFSGTYPYTKRDKKWATHFILMHGELTIICMLFIYNIIEFDLKAADYSQMCRNMCLSFVYLVITLLYINMLYYQSKLKMLIETMKAEYELAKTMSEEEQNVILEYAKKGRWLCRAWAILTTCGMAQFFLKSIVCTIYSAIQGNFRIVQYYEVICPEVIERHRNNPVIFITLYFCTFFYSLYTSALYTSVLPLGPIFLLHGCAKLEIVRLNIKNLFDNDDYVVQERLKKTVLQMQDIYCYSHEINECFQILYEFLLKATSLVLPITIFAVIQALGRGQFIPEFFAFIFGAFMVGTTPCYYSNMLMEK

>BmorOR36

MVFNSKKNIISLFSLLEDSRHPSVGPHLRLLSLTGIWYPNSKTNITLLKRACFYVIVLFFVSQYLKCIIKFKIDSLQLILEYAPFHMGIVKTCFFQKDYNVWQDLVSFISKTERDQIAKKDPKSIKTIQSYISRNRKITYSFWALAFIANIGVFSKPYQNNQSDVNGTVTYNHLFDGYTPFSEEPPGYYFSMGIETILGHVVSFYVLGWDTLVVSIMIFFAGQMQMSRLQCSRMINGSPERTHKNIIKCHKFHTDLIKYQKQFNSLISPVMFVYLFVSSINLSVCIVQIAEIEDDFATVLSSFIFLLACLIQLLLFYWHSNEVTVQSELVSYSTFESNWTSTQNKLQKEVALLGLTTSKTLVFTAGSFNHMTLATFISIIRASYSFYALLNSTKY

>BmorOR37

MELGCSRHLKLPCSLHPIGISKHGNTLSELLIYFPAIPKITYAILAVLLTVYYYIYLCSITWFVFVRCPQTGDLAAASIVFSLGVSSEIGAIKLFIMYVYRAKLRDITGEYLQCEADMAPGRLRARVGRSLRTVRRRAFVYWLVLVVNAFAYDLMPAFLPGRHLSEDVFVIYGFEPMFESPNFEIASTLMGVSVVFICYTAGSISAFLIVIVGYSEATMLALSDEISCVWDDACASECQQPNDFIRARLGKIVAIHTKQIRLIREVEVVFRGALAGGFACVAFGLIAALLGGLENTFLQLPFCVIQISVDCFVGQRLRDANVAFETAVYNCKWEYFDKSNMKTVLLILQNSQKTMGLTAGGVAALDFTSLMTIFKSVY

>BmorOR38

MVVFSLGISSEIGSTKFFNTIIYIKELRKLFKDYLLYDATCPAQGRLRLHLLTTLRYVKRRAIIYWLVIIGNGFIFAIKPLLVEGRHLAQDDLVLIGLEPMRQSPNYEIAYAIMTMGVCFICYPPAHVTMFLIIIVGYTEAQMLALSEELKHLWNDAIEHYEKHSRTEREADAAMKSKILNSFVNFRLVQIIKSHSTNVNLIGRVENVFRGSLAVGYVFLIVGLIAELLGGLENTYLQVPFALIQVAIDCFIGQRVNDANIDFEKAVYDCKWENFDKRNMKIVLLLLQNAQKTVSLSAGGIAKLNFSCFMSVIKSIYSAYTTLRTTMK

>BmorOR39

MLWSVFSYFTRADDVLAGIVIFSLGVSSEIGLVKLCFMYANIDKIQKITEGYLKSDAASARNSRFSKNILHTMQSVKKRGVIFWLVIISNGVVYLVKPIVTPGRHFMEDQFIILGLEPKYETPNYEIGFFMMAVGVCVTCYLPANITAYLITVAGYSEAQFLALGHELANLWPDAQLHCRAMNLSQSVNEQANEYVKMRLRELVKIHSTNVNLLRDIEGAFRGAIAVEFLLLIVGLIAELLGGLENTYMQVPFALIQVSVDCLTGQRVMDANLALERAVYDCRWEEFDASNRRVVLLLLQNAQKVATLSAGGIATLNFSCLMAVIKSIYSAYTTLRTTMK

>BmorOR40

MTGAGAGTFRTGAGPGRGDGVARRGESGETTTLGRDAFAALGCFGAADGSTARARFFPRVTVLNPSEVPGSGLAADSNSISDSESEPELDAAQDAIDAGAGVGGDIGESRARTVFGIQGHDASDSALRMHNNVAIYAKTTMSGNSQLTFATAATIFLKNASGPNGVAIGTDYAICVVSLSLFFCYRFTELVEDTYNSYLLFQLVGSVGIICMSALRILVVDWRSVQFFSILCYLSVMISQLFVCCWCGHELSATSEELHTILYNCAWYDKDVKFKRDLIFMMARARRPILLRAGYYIGLSRQSFVSVSIPRIRFNAILVI

>BmorOR41

MMGNSTDLFLDRTKSILNFFAMWRSFEKPIPLKVYMAFIMTTQYLFLIFEIIYIVNVWGDMAEVSEASILLFTQASVCYKITSFISKTNNFVILLGLIESEIFSAQTELHEKILILKARKIKRLCMFFLVNAVTTCSLWAVIPLLDISSKMLPFKIWMPASTGESPHYELGYLYQMITIYISAFLFIGVDSVPLSMIMFGCAQLEIIMDKIGKVKSRPLDQQPMQRQAVLNSNYELLVECVRRYQSVVRFIELTEKTYHANIFFQLSGSVLIICNIGFRIAIVDSNSLQFYSMLTYLVTMLSQLFQYCWCGHELTIRGEELRETLYQSPWHEQDIRFRKVLIITMERMKRPIIFKAGHYIPLSRPTFVAILRCSYSYFAVLNRVRNE

>BmorOR42

MDIPKFEELLKQIKMNFWLMGIPFDNPKIQIRYYVLLLPLSLMLIEEIAFFGSRMSSENFLELTQLAPCICIGVLSVLKILALTAKRQKIYELTQNLECLHKIILNDTRKTELVRKNLVLIKFITKYFFVLNAVLIFVYNFSSPVIIAYNYIVSNEVQFVLPYAVLLPFKTDSWIPWLIVYVYSIFCGFTCVLYYATVDVLYCVMTSLVCNNFSLISFKLQKVNRNTAHLLKEVVKEQQYVLKLAEDLENIFTAPNLFNVLIGSVEICALGFNLMIGDLTQIPGCILFLSSVLLQILIMSVFGENLISESSRIAEAAFLCKWYEMDQKSKKTILTIMIRSHKPKKLTAYKFSVISYGSFSKIISTSWSYFTILRTMYTPPGTKFQDDL

>BmorOR44

MYTYFKVLVFWLNKDKVISLQKILHCKEFKPKEPEHKEIIRKSIRKARFVMTSYATMCVGAVSVGIILPLTENFDILPTNVEYPFFDVYKNPTYAYLYLHHIYYKPATCIIDGVMDTILAAFVASAIGQIEILAFNLRNFDVLAERRRKRAISGNKYIGKYTNLYFTKRILKECILLHNSIIRYVSVIESAFSLASALQFMLSVMVLCLIGIQFLSIENPTSHPMQMVWMAIYLTCMLIEVFILCWFGNELIWKSNDLRQAAFDGPWRNLNRKTCMFIIIFMERCKRPMRLSAGKIFTLSLDTYTVLINWAYKAFAVMRNMKK

>BmorOR45

MKVLDNVNHAVKVTMNCCRLYGLFVSDDLTKRQLIIMRAFSLMLYLFFVGFFITTQSALIITMWGDLNLMTNVGLVLGTHLTLSAKVFTLHYKEKEITNVIYKNEVRLRAETREQGKYIISEYPCNTTKSPAHEIILAHQGIAVILTATLEIAIVLLMTSIVAVCRCRLKLVGLSFETICDDLPSNIMNKLTADEQVIVAKRVRENVIEHQAVLECINDIQDCFSSAMLVHIAISTMIICATAYQLAVEKSLDLTQRMTMASFLGGMSTEIFLFCYQGGHLSIDSMEVATAVYSCPWYTFPTSLKRSLLVIMIRAQQPALLTAGGFAPLLLDTFVSIMKASYSFFTVLQNASE

>BmorOR46

MAFFIRNKMLGLTITLNTLSWAGLIMRDQYTKTQRIIVRVYGWLVFLYLFVAATYVQIADLIDIWGDLDLMAETSLLLFMELAVISKILTLIFKYDKIMEIINGTEDILCSENRLEGQKIIASIDKETTRFFQYYTSSVIFTTFFWFLGEHSSTFFIRAKYPFNELKSPGYEFALIHQCMMMVFTGYFEFNINIFFASVVAGCRCRLKLVALSLRNICINIPVNKKNLITPEEEKLITERLHCAISQHKYALDAAEDVKHCLSKVLLVQLTVSIVIICTTAYQMAVNKSTDTIQKLSMAGYLLGASFEVFLFCFQGQSLSNASEDIADAVYECPWYTLTQPLKRTLLIIMMRAQSPAILTAGGFVTLDITEYMAVLTGHGGFGDFLHRTGAEPMAECHHCGCDLDTVQHTLLVCPAWKGWRRDLVVKIGNDLSSVLWHRCSAATSRGRRCLTSASAPSRRRRRGA

>BmorOR47

MKLVFDNFISALKVTLNWSRYIGIFIPDELTGRRQKLLVQAYSVFMYYLFIGFFITTQIISFILVWGDLNLMTDVGLVLGTNLALSAKIAVFFFKREDLANILKKNDDTLRSETRAEGKKIISETSCESARVGTTTLPISAVVTILETLELISQASLLITVDIIMLSMIAVCRCRVKLVGLSLQTICDDLPCNVKNKLTSDEEVIVAKRIREYVIEHQAILDCISELQNHFSPALLVQLLTSVVIICVTAYQLAVEKSSDLLRKFTMASFLFAMSTEMFTFGYQGGHLSHDSMEVATAAYSCPWYTFPTSLKRSLLVIMIRAQQPALLTAGGFTTLSLETFVTIMKASYSFFTVLQEATD

>BmorOR49

MLTCFATIFSAVNQTGYIVLFINLLAHELGHFYVITDVLNGIFEKNDADRDPVFIDRKLKFCAKHYQYLLKFHNEIKNLYKIIFGAHFLMMTIVLVTTLQTMNSWDIRNTVLTAVTGIMPLFIYCFGGELLITAGMDMSTAIYQCGWEKMGVKQAKVVSVILCLSQRPLCLTAANVFVMNRETFGGIAQVVYKIYAVFN

>BmorOR50

PIFAAKTLKDKQHLIQNKKEVTRFARLLLTYVTVGGFIWPMSFCFRRIKDPNTVVPFYVPFTPDNWTKLINEVTDIFNPCLTFQFFTSSVAICMVIYKLSDTYIVSLEFVFLLNFIFVLLTQMFIYCYYGNVVSYESKYINTSLYLSDWSSASPGVRKMFLIVMPRWTRPLVVRIARVVPLSLDSFVSVRKYKCSIEHFIYSHVKQTRARDIFSGASNKP

>BmorOR51

MDCTIVAFYSQAKTQIKMLRYDLEQLGKIDNIETKFTENIFERSSHIWKALKDEKIKIHSKLVFCVEHYRQIVWFVKEVESIFGEAMTVQFFVMAWVICMTVYKIVGLSIYSAEFVSMGVYLGCMLAQLFIYCYYGTQLKVESESVNTSLYCSNWLSSLPKVRRQMLIMMQYCSKPLTPRTAYVIPMSLETYISVLKSSYSLFTLLNQKH

>BmorOR53

MALKKMLALTKGLEDPTHPLLGPTLKALSVFGLWQTGSQKSTVIYNTFHFLTFLFVITEYIDLYTVRKELSKMLNNLSVTVLSTICMIKTLSYVCRQSHLKVLVREISELELELMKTTDKNIVKRLRQYTVYTRAVTYVYWFLVVGINVVLLTSPLLKYASSEIYRSEIKNGTEPPPLILCSWFPFDSARMPGYFWATMVHIIMSIQGCGVVATYDMNAVAVMSYLKGQTSILKDKCKAIFDETASSRDVLNRIRDCHRHHNILLRHYYMFNSLLSPIMFVYMLICSFTICCSIIQLDSSETTISQRIWIIQYSIGQISQLFLYCWHSNEFAAKVKKKHFPLFPINLF

>BmorOR54

MGLNTIKEFFVNVKRRFQDVSIDSLLWIVNIVPSLAGFSIRSDRVSAPFWIVHWSLLVYVYAVGNAVYQWKFANEAIDYITSFINVSLLILIGNNSWWFLANRRLLKSVLHKIEVNDELSRRSEQSRLKHKKLLKIIKRIVLVFYMSNYVNASFIYLPNRVDVLNNYAMTPCVGMEPLTVSPNRELCLTILCMQEFSIMTVVLNFQALLLCFIAHTAVMFQILADEIMALNNYENLEEHQAYVKEMLPIFVKRHSLTLSAVDNYKSLYSVPLGVNFGSNALTILLILYLPVLEWFKFIPIFVFCFMLFFLYCFLCQKLVNASEAFETAIYCCGWENFALREMKMIYVMLHQAQKPVELLAADIVPVNMNTFATTLQAMYKFVTVVKF

>BmorOR55

MCFLKIKQQIIDIQKHFKDYSLNGSLWIVNLLPRLMGFNLRADKVGVFFWTIYILLLVYVFGIGIFVYLWKHVDTMSGLMKSYLNLSLILVIVNNSCWFLSKRSLLNKVLKKIHLIEDLSCESEHALAKYRRVFKIVTHLLLASYVLFYFTEIYFMFLFRNYDLLEDYSLAPCVGLEPLSSSPNSEICLIIVLIHEFISTTVMMSFAALFLVLIAHTAVMFLVLAEDMTKLTDLINLADHRKMIRESLRSLIHRHSLLLQIVYELRLLYSVPLGINFISNAMSILVLLCLPIHEWPSFLHIIGYCFFAFFLYCFLGQNVINASEKFIDAIYCCGWEHFGVAEKKLVHVMLRQAQKPVEIIALGMISVNMNTYVEALQLIYKFVTVLKI

>BmorOR56

MKLLEKLEDPDRPLLGPNVKALKFWGLLLPESRSKKYFYLFMHFAVTVFTATEYIDVWFVKSDLALLLNNLKITMLATVSVLKVTTFLLWQNAWRDLIGYVSRADLEQRATSDSRKLALINGFTGYCRKITYYYWFLMYTTVAIVTVQPIFKFFSSAAYRLDVQSGNGTYLQVVSSWIPWDKNTLPGYLLASIYQTYAAIYGGGWITSFDTNAIVIMVFFRAELELLRIDCAALFDDEKSFGDMAFMRRLKECHRRHTELVKHSRLFDSCLSPIMLLYMFVCSVMLCVTAYQITIETNPMERFLMTEYLVFGVAQLFMYCWHSNDVLYASQDLSRGPYESAWWSRDVKYRKNLYILVAQFNKVIVFSAGPFTKLTVATFIRILKGAYSYYTLLSQSQMNKT

>BmorOR57

MPSLIKNRIFGLTLTLNTLSWAGLILRDDYTKTQRIIMKVYGGLVFLYLFVFTAYVQIADLVVIWGNIDFMTETSLILFMQLAVSAKVLTLMLKSKKIMEVTNEADAILISEKKVEGQRIIASIDKNTTLFLKYYGFFVAFTIICWFMGENTSTFFIRSKYPFNELKSPGREFAFVHQCIVVIFTGSFDFNVDIIIISLVAVCRCRLKLVALSLRNLCLDIPMNKRNLITSDEEKVITERLRNIISQHKRALDAAEAIKHYLSGALLVQLMVSIVVICTTAYQLAVKKSTTMQSLTMAGYLFGTSLEVFLFCYQGEFLRESSEEIADAAYECPWYTLTRPLKKTLLIIMTRAQRPATLTAGGFVTLDITEYMAVSLISNT

>BmorOR58

KLVFDNFIFALKVTLNWCRYFGIFIPDELTGRRQKLLVQAYSVFMFMLFIGFFIITQIILFILVWGDLSLMTDVGLVLGTNLALSAKIAVFFFKREELASILKKNDDTLRFETREEGKKIISEYPCDTKRSPAYEIIMIHQTIAVAVIASLAITADLLMLSMIAVCRCRVKLVGLYLQTICDDLPCNVKNKLTSDEEVIVAKRIREYVIEHQAVLDCISELQNHFSPALLVQLLTSVVIICVTAYQLAVEKSSDMLRKFTMASFLFGMSTEMFMFGYQGGHLSHDSMEVATAAYSCPWYTFPTSLKRSLLVIMIRAQQPALLTAGGFTTLSLETFVTVS

>BmorOR59

MDTNPSAAGDSVAPHLRRLRQVGFCQLDPTSQSRRPILALMHRVYHRLVLAATVLYIFEQLTYAYQARNDMERLSRVLFLMLCHLTCIAKQFVFHSDADKINQLVVGLDDALCNQPVETHRLLLLETSRRAARLLMLYSGCAVSTCILWAVFPLLDQLRGRTVEFAFWIPIDYRHNAFQFAVVLAYAFYSTSLVAVANTTMDAFIATVLYQCTTQLRILRMNFESLPERAYALSRKTRQDYHTVTHELLVDCLLHYKKITETCNLLEQIFGKAILVQFGVGGWILCMAAYQIVDMEILSIEFASTALFMGCILTELFLYCYYGNEVTVQSGLVSESVYAMSWLSLCPRERRALVVVLERARRPLRPAAGRVVPLTLNTYLKILKSSYSFYAVLRQTK

>BmorOR60

MVRPCRYFAIHFILLRFLGLGWWHHPHENETRNYPGLYLYYSILTQLVWVVGLVGLETIDPFVGEKDMDRFMFSLSFVITHDLTLIKLYIFYFRNVEIQDIVRTIEIDLYRYYQNDDKIRATIRISRIFTAAFLFFGWVTIGNANIYGIVQDLRWKDIVKNLNETTSKPLRTLPQPIFIPWPYQEDKHYILTFILETMGLLWTGHIVMTIDTFIASVILHMSTQFAILREAIVTAYDRTMIALSEGALQSGVLCENSNGNEENNQIFLESFYSKEHIESVLESTLLSCIRQHQLLIGCVEKFSKTYSYGFMTQLLSSMAGICVVMVQVSQGASSFKSVRLVTSLAFFFAMVIQLAIQCFTGNELTIQAERIADAVMESKWEKMPVRLRRLLLVTMMRAQRPLHLTAAGFAYIDNTCFLSILKAAYSYYAVLSQKQG

>BmorOR61

MARITDVFRLNFIFWKFLGIWGKSAPSKYNMAYTALYLSASLFVYDIFLTLNLIHTPRKLETLLRETMFYFNHLVAMTKILKMFIRRKKILVIFDLLDCEEFKPSDEDSQEIMKRKNEFYYIYWRIVAVTSNLSCFMQVVGPLIKMLIWKSELGLPVCKYYFMSDEFRNKYFVIWYIYQSFGIYNQMVNNLNLDTFNCGMLWMAVGQLQILKTKFVNFKLNDIENSLDLKTRDDMQTERLRKYLTHYEIILKYCATVQDILNITIFVQLGMSSIVICVGLCGFVAMPSNTETAIFMSSYLITMTMQIFVPSWMGTQISFECGELMSAAYCCEWIPRSKLFKRSLILFVERAKTPVRITGLKIFTLSLDTFTSIMKTTYSFFTLIRQLQVDEVN

>BmorOR62

QNVILEYAKKGRWLCRAWAILTTCGMAQFFLKSIIVCTIYSAIQGNFRIVQYYEVIYPEVIERHRNNPVIFITMYFCTFFYSLYTSALYTSVLPLGPIFLLHGCAKLEIVRLNIKNLFDNDDYVVQERLKKTVLQMQEIYCYSNEINECFQVIYEFLLKSSSLVLPITIFAVIQVSSLHICFIFLIPNTQWNVCSQNKHFLGSLEMSCRNCHSK

>BmorOR63

MKLWIRNANFTISLSLTLLRCLGFWSPDGLAGNKRLLYNCYSFVFFMFLLGIYILIQVVDMIKIWGDLPLMTGTAFLLFTNFAHATKVINIVIRKNRIQRVIQQANAVLMGVQSEEARRIVKSCDFETSIQLCLYFLLTFVTTVGWATSAEKHQLPLRAWYPYDTSKSPAYELTYIHQVAALLIAAYINVAKDSLVSSLIAQCRCRLRLVGLALASLGQDLKIDYQSQLSPAQENILNLRLKTCVLEHQTVLAAVTELQACFSKPTFAQFTVSLIIICVTAFQLVSQTGNLVRLLSMGTYLMNMIFQVFIYCYQGNKLSVESSEIAGSVYFSPWYLGSVKLRRALLIVMVRSRRVAKLTAGGFTTLSLASFMAIIKASYSLFTLLQQVKQKK

>BmorOR64

MGVSNGRGTVKPFLYPLVDELDYNLIVGVHLPFEYKTPSRYPLAYITVVIAFIYVSYFVMVTDLIMQAHLLHLLCQFNVLADCFENMLNDCVKGFEGPLVSLHEYIHPLIDEFEYNLMVGLRLPFSFDTPLRYLFTYVIVLIAFNYTAHYVMVTDLIMQSYLIPLICQYAVLADCFENILIDCSNDYGDHARRNDIVYSRSMELRAILSRPMLGQLASSGLLICFVGYQATTSISVNIVKCLMSLFYLGYNMFTLFVVCRWCEEITNKSLNIGNAVYCSGWESGMTVVPTVRSTILLVILRANKPIVFTAGGMYNLSLTSYTSLVKGSYSALTFLLRIQHE

>BmorOR65

MRLGFEVSISEYLYRNIFYIYTLFHILLHFYYILHMIKLDLEAIFDDIDESVALLPHRDTRRIEVQKILNGRMKRVVTWHISVFKAVEAVSSIYGPPLAYQVMFTSIAICLIAIQITQKLENGILDIRFTMLGVAACLQMWIPCYLGTLLRNKAFGVGEACWNSGWHQTPLGRMIRQDIIIVLLRAQQPVTIKFPGLQSIQLETFSSVIFNLYGYYYFLLLRWVDELTAHLVLSGYWSP

>BmorOR66

MRFGLKVYIYTLFHILLHFYYILHMIKFDLEAIFDDIDESVALLPHRDTRRIEVQKILNGRMKRIVTWHISVFKAVEAVSSIYGPPLAYQVMFTSIAICLIAIQITQKLENGILDIRFTMLGVAACLQMWIPCYLGTLLRNKAFGVGEACWNSGWHQTPLGRMIRQDIIIVLLRAQQPVTIKFPGLQSIQLETFSSVIFNLYGYYYYYCLDG

>BmorOR67

MRFGLKGGAAVVTILETLELISQGGFIETIQVTFGGQLSSMLFISACIICSTAVQILAIESPLDNLTTVGWILVYLSLCILILFVDCYFGNTITVKCAYLPTAVFSIPWLDQPKNIQVSTLLFMAKTQQPVQLIAAKLVPVSLTTFTQVSYCPPLDLKCLQGGVIAHLAKD

>BmorOR68

MFTIDFHDERITSFNKNQTRKIIICVITGGRTSCESARVGTTTLPISASEDVCMTLYSCGWETRFDLNTRKCIILMLCRALRPVSIRTIFRSVSLTTLTGVFQQAYALFNLLNAVWN

>CsupOrco

MMAKVKAQGLVSDLMPNIKLMQAAGHFLFNYHSDNSGMSTLLRKIYSSVHAILIVINFLCMAVNMAQYSDEVNELTANTITVLFFTHTVIKLLFFAVNSKSFYRTLAVWNQSNSHPLFTESDARYHQLALTKMRRLLYFICTVTVLAVVSWVTITFFGESVRLIANKETNETLTEPAPRLPLKAWYPFDAMSGTMYIIAFAYQVYWLLFSMAIANLMDVMFCSWLIFACEQLQHLKAIMKPLMELSASLDTYRPNTAELFRASSTEKSEKVPDPVDLDIRGIYSTQQDFGMTLRGGGGRLQTFGQQNTNNPNGLSQKQEMLARSAIKYWVERHKHVVRLVASIGDTYGTALLFHMLVSTITLTLLAYQATKIGGINVYAFSTVGYLSYTLGQVFHFCIFGNRLIEESSSVMEAAYSCQWYDGSEEAKTFVQIVCQQCQKAMSISGAKFFTVSLDLFASVLGAVVTYFMVLVQLK

>CsupPR1

MDFELKENRFRTIEFLHNRIVRNFLMPLGGWPCEVFQEKTPLFSRFFRRFIPIQGSCMIYGELNYIIQNYSRLNFFLLGHIYVTMFLTGVMIIRAILPNKKEYNDLVNFFYGEFDLEHFKHKGSYYQKASEIVYKFSYYYSLVMAGMMIYGMLLYNALPLYHNYNAGVLHRSNRVANVTIEFSVYYSFPGFMPEDHFWFVTFTNLYLTYSCTVEICIFDLFMALFVFQMIGHIMILINNIKNIEMPKTCHNIEGFKTQTNVTVELYDYEENEILRNKIVEIVNHHRFILRFVSDVSFLLGPALASTYLCHLISCCLLLIECSQLDPDALAQYGPITVIMFYQLFQISVLFELLGAKSEKLIDAVYELPWECMDVRNQRLLCFLLQRVQTPVQVTALGLTKVGVTPMVAILKTTYSLFAFLRSTV

>CsupPR2

MIYGELNYIIQNYSRLNFFLLGHIYITMFLTFVMLVRAVLPNQQLYKDMVEFFYGKFDLEHFKHKGPYYQKASEMVYKISYYYGLVMTGMMICGMFLYNALPLYHNYNAGVLHKRNRVENATLEFSVYFIFPGFMPENHFWSVTFVNLYFTYSCSVEICIIDLFMALFVFHMVGHIMILLNNIENVEMPKTHYNIEGLKAQTSVTVALYNDEENEIMRSKIIEFINHHRFIVSFADDVSSLFGPVLASTYMFHLISCCLLLLECSQLDPDALAQYGPLTVIIFNQLFQISILFEFLGAKSEKLIDAVYGLPWQCMDVRNQRSVCFLLHRVQSPVQVTALGMTNVGVTPMVGILKTTFSFFTFLRSIV

>CsupPR3

MLESTPLKNLETRKFYLLAVFLLSTFQMYETVDLFSRLYTILMINMMVGGMILFNLTPLYSNYKNGVFSKNPPENVTYAYSVTYSVPGFNFYEHFTLTTILNWIMSYDVSVNVCVKDLYLSFLVFQIIGHIQILKYNLEHFPKPKNQATNRFDAEENKQIRKTIAECVDHHRLIVSFADDVSDFYGPMLALNYMYHLISCCLLLLECSQKEPDALARYGPLTVIIFGQLISVSVVFEIVETKVKSTKYGANCLTLTLSMVPFSK

>CsupPR4

YYKEISDRVHSICMLFTTMLVVEITLTVLMFNIPPIVNNYRAGMLSTTRPVNGTFEPTFNLAFPYVDTESKSLSDYIPMTIVQVYLTVDCTVCLLIFDCLSSLILFHVWGHIKILIYNLNNVPVPKTSSQFELNEDPLFYSSEEDIMIKNILKENIEHHI

>CsupOR1

MRFRIFQRKLSLEENKSTLQSEPITNYLHFLEIPLKIVGCWDWYMKPETECQIILNNIYYGMVLFFLINVPATLCVHLSTEWKDVMTTLDEIADCLPYFVSIVIVIYFGVYRQEMYDLIQSMGEQFKYRSANGLTNMTMLNSYITAKKFALFYTICTLFSVSMYVVPELISWWTNKPLQSFMYMDITKSPFFEITFLTLYLSQAFVGLAMGQFGVFFAANSILLCGQLDLLCCSLRNTRYTALLQSGVHYRSLRLSHSDIKSDELHNYIYNVAEMEESSYHYDDKMEAITRPRKTKFDIYSSEYDEATERAVRDCARMCRVVNSYRERFERFVSPLLAMRVVQVTMYLCMLLYSATLKFDMVTVEYLGAVALDIFVYCFYGNQIIIQADRVTTAAYQSAWATMGVRGRRLLLNVLLANRRAVAVRAGYFLPLNLHTFLVIIKTSFSYYTLLVNVNEK

>CsupOR2

MASSTRPRHYFYLHFLLFRVLGLGWWHQPDERDTRNFFGWYLYYSIVTQFVWVVGFVGLETIDPFIGEKDIDRFMFSLSFVITHDLTLIKLYIFYFKNDQIQDIVRTLEIDLYNFYQNNAKNRATIKISRLMTASFVFFGWITIGNTNVYGTIYDIRWKAEVAKLNNTDLAPPRTLPQPIYIPWNYQTEEAYISTFVLETVGLLWTGHIVMGIDTFIGSLILHMSNQFSILREALITAYDRTMIRLYEGVRQDFIAITNSDIDKKEQYTQDNIEEVVKSRYSKEEIEVALTETFKNCFRQHQALIGCVENFSTTYAYGFMTQLLSSMAAICVVMVQVSQDASSLKSTNLVTSLAFFVAMIIQLALQCFTGNELTLQASRIADAVMHSKWEKMSPKLRRLLMITMMRAQRPLRLTAAGFAYINTDCFISIMKAAYSYYAVLSQKQST

>CsupOR3

MCFNIASVRHTTDNSTQISFEKKSLGALFTIVNLKVKDIRKMARSQRLSIIASIRHILSTAGIKFTDTMHVHWMAKVAMICLIFTYVLQASALIQIRHNWEFFFECSGDLFYRGMSLVKFYIFRRNYETWCSLIEQADKIEEDELSNERDRENGNFLFSEHIQAYSVRYEKIKKIITTIFRTCTVMYVSSAFIEYGIKKQTVDGSVDLPHILEIWSPLDVSIVGYIITVSFELISAVYDTSTQIAFDLTSIGAMIFISGQFSLIRHYSEAIGCKEQIYPSKEQDDLAHKQIIICHQIHIQIKHLTEMLKGLLTNILWLYFIMSTVMLCSIVVRLNLETSLVQLMTMFLYMCGITTQLFLFCYFCDDIQNKSAIGMGEGPYGAAYWSLSPRIRKELMILARGMSIPCQLYAGPFIPVTLPSFVQILRTAYSYYAILGNRG

>CsupOR4

MKTVLTPSQFAIFVQFVMLERVRKFGLRYCDLPTMLSNVSCMLRVLTLNVDSRHQKGIPIIFYVLTATAAASYFYIYLVSMVWFVFWRCSDTGDTIPAMIVFSLGIASEIGPCKLIFMFLYRDVIMKIVDGYLSCDATVIKGKRFSENLLKTLRVVKKRALIYWLVIIGNGITYVMKPIILPGRHIMEDQFILLGLEPMFETPNYEIAFTLNTMGVYFTCYLPANITAFFIVIIGYTEGTMLALSKEIRHLWDDAQQFYQETFNNAEVAINGGTIDPTFKKRVINQFIKERLQKIVVIHTTILNLIHQVEYVFRITIAVEFVLLSAGLIVELLGGLENTYIQMPFTLIQVAMDCFIGQRLMDASVVFEEAVYDCKWENFDTKNMKTVLLILQNSQKTMALSAGGVTTLSFSCLMTVIRSIYSAYTALRSTMT

>CsupOR5

MKILKRNIKEKLAILKPILPYGVLESWDDLDPKLYHAVHIYWLKFYGLWYYDFAPGSFMFWIRFLYTMLVMWLVCFLPGIGEIVYLLKRRDDIGDIAEGLYLFLSEMYTYFKMSVFWLNKDKVLNLLQYLTCEHFKPIEAEHREIIKKSIGTARFLMTFYSTICVGAVSVGIIMPLTENFDILPTNVEYPFFDVYQSPAYGILYFHHAYYKPATCIIDGVMDTILAAFIVSALGQIEILTFNLRNFDVIAKRRHKRALDGNKPEASWSNERHIRAVLKDCIIHHKSIIRYVSMIESAFSLASALQFMLSVMVLCLVGLQFLSIENPASHAMQIIWMAIYLTCMLIEVFIICWFGDELIWKSRGLVQAAFDGPWLKIEQKDKIFIVIFLERCKRPLRVTAGKIFTLSLDTYTILINWAYKAFAVFSKVKK

>CsupOR6

MWKTIRKFGLEYCDLPTMIWNVSSMLRVLNLNIDPGNTKPIPTTYYIITAIVTASYFYVYLVSMVWFVFWRCRETGDLTAGMIVLSLGITSEIGTTKLMFMMIFRNKLREIVELYLECDSHVNPDSRFLHNMMKTLRHVKKRAMIFWLVIIGNGVVYIVKPAIQPGKHLMEDVFTLYGLEPSTEWPNYEITFVLMALGVVQTVYLPANITAFIIIIIGYSEAQMLALSEEVLNIWNDGLHHLNDHVIIDACADSNDQLTSLEEIISANRNNYDRINEFIKIRLREIIKVHMTNINLVQQMEQVLRGAIAVEFGLLIIGLIVELLGGLENTYMEVPFALMQVAMDCLTGQRLMDASIIFEKSVYACKWENFNVENMRTVLLMSLISQKTMKLSAGGVTMLSFSSLMMVIRSIYSAYTALRPTMS

>CsupOR7

MFWNKIKAVYNKEDFDYSKRFVDPKIYHRIFFAVQRAYQVIDEPFATWTYITKTITALCGVGVLTDACLSLYHAIDIFDMSLITESGTYVLMLLYKMMTLITTKVNLSDYIHLIHAMKEDFNYIETKKEKYRKVFFETQLGTWKACFVVISFMFSMGTSLVLFASGTLVVYHLTHTPGDGSHRTLVFPFWAPGVDYTTTPAFEIAFTFANIGVMACCYNYAFVIQTNIVWIRQIAAKADLIGMCINDLLEGIYSTDDEEQRQHFASLINFRMKEIVSQHIIMYRLLECYAAVYRKCLMFEQFIASPLVCMLAYCSAEKIDNGEVHVVMMVLCLSAILVLFIPCYLCTYLRTKLSGIHDACWNIRFWDAGPNIRPYLVLIMQRCLRPLPLQMPGFQEVSIKTFSSKMTSAYSLFNMLRQADLDF

>CsupOR8

MERMIKNLCDILISVSYREVTTMLRPFFKRLENNNHPLLGPTLWGLARWGMWQPRLGINTKIYCILHIVATLFVISQYVELWIIRYDFNLALRNLSVTMLSTVCVVKAGTFVIWHDQWQEIIEYVSKCENRQLSKRDKITSEIINNYTVYSRRVTYFYWALVAATVFTVTLAPLAAFWSSKEYRARIRAEQIPYPEIMSSWLPIDRTRGIGYWLSIVEHTLICFYGGGIVATYDSNAVALMSFLAGQLKLLNTNCSRLFEENYESRNNTVAKIREYHHDHLRLIKYSKILNGVLSPVMFLYVIICSLMICASAIQIATNGTTSMQRIWIAEYLMALIVQLFLYCWHSNEVLIMSHKVDDGVYASSWWSQSQSVRRSVLLLGGQLRRPIVFTAGPFTKLNLPTFLAILKGSYSFYTLLINKED

>CsupOR9

KRRRQWLSALIQFQLNQQKSTMLLDRLISFAKRWEDPESPLLGPNLKALHLFGLWKTDIRVRSTLLIIVVVFVITQIIDLYLSREDINKALCNFSLTTLSVICIAKSYSLIVHPVLWKKLVENISQEEATQIKKQNPETLSVIGNYTRYSRFISYTYWIMVAMTNFALIVAPLIRYLTVSKYREDIANGIERYPHIINSWFPFDDNAMPGYVYASAIQIIMSIVGSGSLAAYDTTAFAIMIFMKGQLIILKNNCKELFRWETKENNIEFFAKIKECHRHHDFLKRQFNQFNDLMSPTMCLYVLLCSITLCCSVVQLISKEATASQRLWIVQYSSGQILQLFLFCWHANELFLESQNIDGGVYASDWWKADVRMRKQILLLAGKVNYPMLLRAGPLSVLSLSTFFNIIKRSYSFFTLISRMQE

>CsupOR10

HPSAKSPIQRKSFDIKGVLLERVLVSSISMDLPTYDEIFKGIKNIFWLTGIPLDAPHKKLRFYVACLSLIITLYGEIAFFTSKISSENILELMDLAPCFCIGALSFFKGIFLAWKLNKIIVLKNSLEILYDTIFKSDSKRKLLHREIMKVHKLVKYYFGVNTALITVYNFSAPIFMTYHYLSQGKVKFMVPYAVIYPFAIDNWPAWIVAYTEQVFSGFVCILFITMSDALFCVLTSQICNNFYVISDEIKRLKNGNHIGLGEIVKQHQYILKLSEDLEDIFRLTNLFSYLVGSLEICALGFSITIGDWSHFLGYILFLVSVLLQILMMSVFGENIIRESGRVGEAAFLCEWHEINEKAKRTILIIMIRSHKHQKLTAYKFSVISYGSFTKIISTSWSYFTILKTVYKPSEVNNI

>CsupOR11

MNVISINPAEYRNTLILSLNYLKICGITLDRNDSFWEKYCHLCVVSIIMMLHFVSASLYIVQELVQNILQEANFISLWLITVQVFLRGIIILTNKTSIRGIIEQIGCNWRSSDLNEEQIRIKKDFLNRLLYTQKVIKIIGWCAGSLFLLPPLLETVFRSFVLHQDSAFVLPFPCYYQFTVTGWFTYFIAYFIQIYCSSKLIFMYLGADLLLIVLCAHLSNEFELLQVDLGATIKPTKNENEILEDEITAFGREERSIGDFVRRHQKVILLVQLLNISFNKMIFINLLFAAIAIVFFALGGRASRDPTNVANNYMAILVILINMFVLCYSSEMLCTSSSGIADYAYNNIWYEADMRYRTDIYFIIMRSQKACSLSSLNYLPISLSTFGKVLSTTWSYLSLANTFFEN

>CsupOR12

MKILTTQNENSEKEIRIKPFHETYKKITYGLTLGFMFPNPRTAKIRIVTIVIMLVLFQPIVVTVLIDMYSCWQKSDMFNIIRHSTILGPFLGAFYKMFLMHYKRAEVKRIIDEINDDYLTYNNYNHELKQIALESIKSSVFFVEQLWTYTVTACIMAFPVMGIVLTFISHLTQSEPKKYMVHDLKIPFRPPEDRFETPFFEIMFVYMFLAAIICVLNYVSYDGLFGLACYHACLKMRMFSKKLEYVFQCKDGDAYSRLVQVIEEQKATYEYNALVQNSFDIWLGTIVISTMIQLGSLLFHISAGYGFDFRYMLFSCTSVVHIFLPCTYASKLRNTSVETSTLMYCSGWERSRERRIVRVMPFLLARAQIASRITAFYLFDVDMQLFVTMMRTSYTIFTLLRT

>CsupOR13

MKKNMKEFEYDFEKAFRITTKALHLNRAHPFIERNLFWCFQFLLILTLSVMTFVFTFNSLLFYDIPAGEIAEASKNGTMAIVSLTITFKYTFLLYNQNYIKRYIAIINKDYELSKGFVAEERAIVIDYSRKGAKVSLYWLVATTATSILFPVKALVQMVYYHWEDEFRFVPMFDMRYPTTIEIMKNVPAMFCLLFLLCLMFDVYATTMYVGFDPLVPIFLTHICGQLDILSQRIMDIFSDESNLNSQEVNYKLKCINVTLQDQYNMIKEIKSKFTFLYEFTMKTTTILLPLSMFQIVEDLQRRKLNLEFISFFFATILHFYMPCYYSDMLMDRSQKLRDAVYACGWEKRHNARARKTILLMLTRTTVPLALSTVFYPICLDTFAEMCRQSYAIFNIMNAAEV

>CsupOR14

LHSCVLFRVTKMDILTSIGGVFRRIVTRFREDSFDSLLGIVDTVPSLAGFSLRKDKIFVPFFIFHLSLLTYIYGVGSFEYQAKTAKSAGDFIKSFVNVALLVLIANNSHFFMMKRSLLRSTLTEMQNSDKLARCNPASRLKHKKLCNRIKYIILIFYFVNLTNASCVYLPSRSNVDVNIYGVTPCYGMGSLTSPKREICKAMLFGQEVTVMIVVLNFQALLIFVIGHTSLLYQILSDEIMALNDYDKSMFFNNPVVKDILPVLIRRHAMILSIINKCKVLYSVPIGVNFGSNAVCMSLFFYLPLREWIDFFPILMYCFIVFFLYCFLCQRLTNAAQLFETSVYACGWENFETNEKKAVYFMLRQAQKPVELLAADIIPVNISTFATTLQAMFKFVTVVKV

>CsupOR15

MFYLLKKLEDKNRPLIGPNVKALKFWGILLPKNLYTRYLCILMYLLVVIFVGTEYVDIWFVKADLNLLLNNMKITMLATMSVVKVSTFYRWQQHWLDILNYVTRADLTQRKTNDVNKIEMINKFTTYSRKITYAYWSLVYTTVIFVVGYPIFKYVFFSSYRQNVLNGSEPFFEIVSSWVPFDKSTIWGYILASIYQAYSSIVGGGWITSFDSNAMVIMVFFRAELELLRIDCANIFGTEKAQVSDEVAMVRLKDCQRRHAEVMKYIHLFDECLSPVMLCYTIICSVMLCVTAYQITTEPSFVQRLVFTEYLVFCVTQLFIYCWHSNDVLYASRDLSLAPYESIWWSRGVEHRKNLFILTAQFSKVVEFSVGPFTKLTVATFIQILKGAYSYYTLLSKSDE

>CsupOR16

MKDIFILKTYCQYIYRVGSGNFWYEERIVGNDRSLSYKIYRGLHFFLYGCLTILEIMAAIFGVFPSDEKRDAVTFAVSHTIVMIKLFSVISNKALIKQLNKNMTELCEEHEEQQLMAEKYKIVKINVAVYFIIVYVTAVFFAFEGLRKLFNGVHFVTVVTYYPAYEDNSALANSFRIFTTIILQVMLMSMIVTVDTFTMTYLIIFKYKFITLRHYFDSLRENYLKMSKNGNQEIAAEQLTNGFVKGIIMHQKLLKTAKNIDTAFGLVIALQLCQSSGSAVSLMLQLALTDQLTFLASIKAILFVLALFFLLGMFLCNAGEITYQASLLADAIFYIGWHEFAPQPPPKRSLRRLVLLAIAQAQQPLIMKSFKMIELTYGTFLQVVRGTYSVFALFYAQ

>CsupOR17

MSEEAKREIAESLSLNTFCMERIGISFESPKSNIANVRQKLMFVLSVWGICYHVFSEIAYICLTLTKSPRVEDVVPLFHTFGYGALSITKLFVLWYKKNVFKQLIFELAGIWPLPPLDDDGQSTKNKSLAALRMTHRWYFAVNVLGVWFYNLTPIGIYFYRKWQGLDVEMGYVWVSWYPFDKHMPYAHFAVYIFEMFAGQVSVFIMVSTDLLFSSMASHISLLLRLLHRRLEALATTNKTEHEQFDEISANIKLHQRLIRYCNDLESAFSLSNLVNVVLSSINICCVVFVIVLLEPFLNVSNKLFLGSALIQIGMLCWYADDIFQANLKVSAAAYNSGWYHTSPRCRRAILFVIQRAQKPIAFTAMGFTNITLVTYSAILTRSYSYFALLYTMYNKG

>CsupOR18

MLKRFFNSLEDPDRPLLGPNYWILKKLGLLLHFGKLGNIFTILIHNMGLLFVSTQYVDLYLIRSDLDLVLQNLKISMLSVVCVLKVNTFLLWCSKWKEVINYVTEADKYERNTDNPDNVQIVKKYTKYCRRITYNYWILVFTTAFITVVTPLLQYAYSSTFRESVKNGTEAFPHIFSSWVPINKNDFPFNWVTVAWHTYICVQGALVVMAYDTNVMVTMVFIGGKLDLLRERCKLMVGVNGVTTSNVDLAEKLRELHKTHVLILRYSRLFNSLLSPVMFFYMVMCTLMLCASAYQLTSATDTTQKLVMAEYLTFGIAQLFIFCWLSDDVLTKSEKVMLGPYESQWWLANVKQRKIILLMAGQLRIVPVFTAGPFTKLTLSTFLNILKGSYSYYTLLR

>CsupOR19

MPPAERHFLGSIILQWMSNRGFLMNNNCYTYWLNGVTMICCHVTFVLQCVAVIDARDDPERLMQCFCDASFGGMCVLKEFSLRKHRHCWISLLSKISHLEEEELEFETKSINQDEDAHNIVFSGHIKEINKKVKKLNNILSRLFSVTAIGYMLTPFIEYGIRKLIGAETAGLPHITQYWSPLKTNLLGYILAITLEILFVINNYAVHTTFDFSVFGIMIFISGQFRLLHDYSEGDCGSTLCISETREDLAHAKIKKCHEIHVKLIRITNKLSKLIKNILGVYFTLSTISLCAIAIRLSTETNIIKVMVLALYTLSAFLQLFLFCYFGDNLQNMSSIGMGKGPYAAAYWALSPRVRKELILLAKGMSRPCHLYAGPFTRLNLPSLLQVKKSYISSR

>CsupOR20

MSLASSSVSPHLTLLRRVGYCRLSPGVTGSSSTRYLHEIYRKFALAAILTYTSEQAIYAYQNRRDIEKLAPVLFLFLCHITCIVKKLVFHLNAPRIDQLIAELEDAAYNSQTPPHRAMLRSTASSALRLLRAYVGCAIFTCILWVMFPLVDRLQQQDIEFHFWIPVDYNRPLTFPLVLIYSYYVTTLVAVGNTTMDAFIATMLSQCKTQLSILRMNFEDLPLRAMEMAYNSTSYEAALSKLFLDCIHHYQKISETMNELLRVFGLAVLVQFAVGGWILCMAAYKLVSVNILSFDFVSTTLFLVCILTELFLYCYYGNEVYVESDLMVQSLYSMEWVHTPLAFKRSLLLTMERAKRPLRPAAGHLIPLSLDTFVTILKSSYSFYAVLRQTK

>CsupOR21

MFIKNPNKSVGCSLSAMMMFGFWLPKNLTEIERILYQCYGCFWFILILGCYAATQIVQLYFDLGILHLMISASFLLLTISTEIIKLLNIVYRRRMIKSMIDDFDQVLRSTDTEEARAIVKRCDRETTILLAIYAIVTIITMVGFAAAADKGMLPIRAWFPYDVTKHPNYEITYTYQILALSVDAFLNVSTDTLVSSLMAQVRCRFQLLGLSLRNLCQGIRINEPLLASDQVVIVKDRLRLCVEQHCATLEAAQKLQDYFSFPTFMQLSVSLLIICVTAYQMNAVIGKPMAFIGVAAYLLDMMLQVFLYCYQGSMLSEESIAIADAAYECPWYVMPVPLRRSLLIVMTRTRRVAKFTAEGLTTLSLSCFMGVIKTSYSMFSILQGME

>CsupOR22

MLYLIKKFLVPAICLGLTFMATGMEMMFVLHGIQIKDYSFATECFCYCVMLGIIPVFYATNLHKKKYLLQILEDMAEDFVFICKLDTKLRNHFIKGQLLIWKLYLSWIVFICVMGILYVGMTLLPLTYQSLFATLDEHMVRPLIFPIWLPKDDPHRTPNYEIFLFLQLALVLLYMKAFGFYVYIQFHVLLHNKVLLELIIMDFDTLFDGLDEFVAMLPNNDMRRIAVQHTLNKRLERIVTWHNSVFKSIAALSSVHGAPLMYQVGFSPVAVCLMLYQIADKLDHGSFDIIFTALFFAACIQLWIPCYLGTLLRNKAFDVGDACWNCGWHETSLGRLVRMDIIIIILRSQKPLSIKFIGLPNLSLETFSSVGITNTRLHIIL

>CsupOR23

MLDENGKKCFENSLRRTKLFLSLSGIRISATKWPKALEKLFDTYFYYFQVFWLYADVLGEISWLIEGVLNGSSFLELSLAVPCITVSCLATSKSIFLYLNRDVVVKVIDKLREIYPESDETLKYHSNADLSHDKELDIFEDDSNKSDINTDIERDIKNESVDFLNLVVKVQYYICSAVVVAFPLMPVSTMVFIYYSTGVLEYKYVYMVKYFFDPFKMALWPFVYFHQVMSTVIVAMNVFGSDTLFYAACIYIQMHFRILCHHYENAVSASSIQTRLNLKVAIRRHHELIDLVNRVEILYTKSTLFNIVTSSFLICLSGFIITMVEDIIVMVTFATFLFMNLSQISLLCYFGDMLMSSSTQIVNAIYNSLWYDADERVKK

>CsupOR24

MVHIYFQHPRLALLLTGLWTPPKEKKFRLLYIAYRICVISMQYAFVTCNLVNMVMLWGDLEQISDACYLFFSVATCSLKNTNYLLSQKKFLSLLDFMENEVFVSQSLVQDKIISAYAKKMGRIYLIYIVCGFCNCVEWALVPVFEKEGHKIFPFKIWMPMDAASAEIPEYLLGYVLQLFGIIFSVSTYLTMDIVAISLLMFVPVQLDIITCKIKEVQHVSILFDPKQRRDLIEHNSALLKDCIRRHQALLRYIEGVADIFDIHIFFQLSATVIVVCIIALQMTIEPPNTFHYYSTVNYLMAMLVQLCFYSWSGNEITERNNVLRDGLYECLWYEQDLSFKRTLWIAMEFMSRPLIFKAGNYIPLSRPTFVSVGLQTWL

>CsupOR25

ETGGTWYTRADYLDFNLKCLFYAGLWPNEKWSRNKQKIYKIYEVTLFIMSFTFMFITSIGIYMAKSGDTIFFFADVDKNIVTYNYIFKIIAFFTKRNEVKTLINYIIYSGDRITDQRKKLMVTHVIVVTGMILALTGVFQILALMKGELIIVAWFPFDPMKNQWSLFLAEQLLVILFAVPCVFRAISIQGIVCSIIMYICDQLTELQSRLKNLNYSVETAAETKEELKLIIKKHIRLMGYAQSLSYAFKEYFLIQNLAVTAEFGLNALMVSIVGADQKKHLLSFIAFLMLALVNAYIFCFLGNKLMYESTCIALAAYESSWISWPVSMQKDVLLIITVAQRSFKLTAGGMAYMSMQTFAQALYNGYSMFAVVRDLVN

>CsupOR26

YLRVCGFYRLDQSSSKSVKILHRIYRRLVLSFFILYTIQQLLKIYDARSNVDKVMGTMFLFLTNTDCIYKAVILWKKADHIEGILEVMKGPIFNKGEPGHRLFLQDTIRKTLLVFRIYNYMSLFTCFLWVLHPTVLHMQGKLIDLPIRLPFDPNTKYYTAALYVWIQTSWLAYCNTAADVFISILLEQCRTQVTILRYDLENVVQKSKEEATETHGNYGDILERKFREMLLHHKEIVKTAGEILDIFSGAVFYQFLVSGWILCTSAYKMVNMNPASIEYASMISYIICVSIQLYVYCYYGNEINYESRRLTDSAYAVDWLEIPVRQRKTLIIFMERIKQPIEPMAGTIIPLSNSTYVSILRSSYSFYAFLKNSSN

>CsupOR27

RDEESNKIYQCYRVVLLTLFKFVFFISFTLDLFFTPINVGLIVSQSLLYFSELAGLFKIFMVLFKRNSILEVFEILDSKEFLAEDEASQQIIQKSHIFFRKYRTACTLFYCTGVIIFFVPVIKYWTTTGAQLKLPSFQYYFLNDNVREKYSMYLFLYQHCCLVLVVLSNSASDTLICGLIMMATTQFEVLNWKMSHLALRPSEKHYDPKEEEIIIKDRLNKCLRHYDVILRYCKEVQETTGLSLFAQYTTGAITICISLSSFLIPMTHEDFVILVCYIAGMTVEIFYPAYLGAELTEKSENLIFSVYCGDWISRPESYKRSLRLMLERANKPVVITCLKMIELSLITFTSVMKSAYSFFTLLKCLLERQQ

>CsupOR28

VWIPPTNKSLLHKLYRSLMITLQYLFLIFQIIFIIQVWGDLETVSQAFYLLFTQACLCLKVSVFHVNVDKLRELLKQMNGEIFQPQSDRQKQILSKQASRIKALLLAFMVSSQFTCSLWAMKPLFDDVGSRKFPFDMWMPVSPEASPHYEIGYAIQVLTIGMSAYMYFGVDSVALSMLIFACAQCEIIMDKIMSVTSINYAMKNKERQKIFAKNRKKLIDCVKHHEALYAFTKLSEDAYHSYLFFQISGNVGIFCMTALRLTVVEWKSVQFFSMATYLYVMMGELFVCSWSGHELTSTSEMLHTAMYDCPWYEQDVRFKRDLCFAMMRMSRPLVFRTGHYVSLSRQTFIAILR

>CsupOR29

RGSCIWSSGVPMAITFVLCLIYRFELRGFLEEMAFKDEMQAMPLIQHVNSLTEGNLLYELKELVRMSQMKLANFSRIFLKVYIMSVLVIATLYPWSSIYEMCVTEDDTLRLIGFDMWFPWSLDDISVYVMSFLFNVYLGCLCSIAYPGLQTTIVLFLGQLIRQLRILNFILSNLSDLADEIVGDQNHNDIWQEVCNSLLCQCVDHYVKLKSFSNRINLTFHFYYLALLLMATVLVCMCSVKIAISDKLALDTMKYYMHGFCFIMMVLLLCTLGQQVDNECEKLEESVTNKWYLYNKNLKVNIQIFKMALDQRMPISIFGSATLSFPTFTWFIKTGTSFFTLVMSVLDN

>CsupOR30

FIKGIQDRHSTDELVNTLFLFLTTVTAIVKQVAFTVRMKRIKQLFDTTDGELFSPKNSAHLELVEQNEKYMRRLHFLYICTVLSTCAYMSLYPLGNKAFGQDHQDINYRIYFPFDPRKSPIIEVFACSYFGIALTLQGYVNSIVDCTIVAFYGQCELQLKLLRYNLEHLTDLDDVDLQEGTINENTLSYIDDNLIKKRLVHCVKHHQKIIWFLSETQSISNEFVTLQLSVACWTICMSVYKLVTVDMFSGEFFLTIGYLNCMLMQFFMYCYHGSQVLVESEFIAESAYCSNWVDISPRSRRLLLIFMMCCTRPLVICAAKIVPINLESYLAVLKASYTLFTILHKK

>CsupOR31

LHKMDTSFEVDLHRHTSLHIAMMKFFQIWLYIPHPRTSYKYWLSVTLRFFVGLFIFVIPTTAQFMYLFSIIRSDDFEIQEVASIINLVITEMLTSLKLLALHLRREDFLDLMKQLDGKQFVCHTKNHKKIVERSIHFSRGIYIVLSICTFIDVLVHMVVVPAVHRFQELPLKMDLIFFDVNDPSYFPYVYAFQISYKPAMVTTFVTLNTLCWACMCCAISQLDILINKLENMKKLIKDTKVDWHYDENEAFDKIFGGIVQHHLAIIRFTKVLQSVLGGQLTLSLFMTAIIVCTTAIEILSIESPRKHITELLWMLVFVSIIVGNLFADCYFGNAITDKSV

>CsupOR32

MFVEKPGMIKNGIRYKPECSVSGALGLVLRLSQLVGIGPLSFRKRNRGWFVSLSPSLCLVSYVAATVLNTAALTGILLDLQARPSKSARVSSPTLKFVWVSDYIVVLVIASVAAYGAPRRLTTTILCLARIQKINTGVSSKSSNDWKTSLLLMAFLLYVACVLTADYCIFLRAVFLSDRAFTAACLYSFYYFAYFLLVLLEMQYVFSALEVSKTMGRLNKLIGEVEHMLTMHYASLKKIESNDPIKLPLKYDNLMIDSMDTFKFDSGMTVGLASKSISETIRRLALTYMEVCEVVRQLDSSHGVGVLLLLLSFLLHLVITPYHLIVKITCLY

>CsupOR33

VSCPHTTVELGTREQQQKDLTNTTFFMPMKQEMSLAGRSVAPHLLLLRWCGFCRLRPRHASAGHPSLLQRAGRRLHAAYCTFALAATSIYLMQECVYAYQEHNDMDKLARVMFLLLCHITSIIKQVVFYTDADRIDDMIAALDDRLFNPKESSAQALLQGTARSAKRLVRWYSSTAVATCVMWIIFPIMYYVSGHQVEFAFWITVDHSGPLMFTVLIIYSFYVTTLVGIANTTMDAFMATILYQCKTQLRILRLNFENLVETANKIVAANPQESYENVLMKLFLEYLEHYQQISETNNCLQDIFGTSILVQFGIGGWILCMAA

>CsupOR34

IYRKIINYISHVEQKQIADGDLEINQIVSEYISRSRRFCYLFWALPVFVDPIMFLQVFITSIRFGKSTGTYPKILDIYIPYSDYPPGYYFSLLIQTTIGFTMSAYIVSWDSLVCVTMIFFAAQLKITRLMCSRMIDPQNPQKSHDNIVECLKFYTTLIEKQRIFNKLISPVMFVNLFVTSINFGISIIEIARVEDDFATLASGCTYLGACLIQLLIFYWYSNEVTVESAKVSYGVFASDWPLISNKYQREVALLGVATAKTLVFEAGPFNEMTLSTFLGIIRASYSFYTLLNKTN

>CsupOR35

SRFVTYNSLPLIIVVDSFPRIIMYYEHEILGEELVYLYPFDGWYPFDKINWYYTIYIWESFMTCVVIFIYGFCNIIHAAYTALICMELEILGNHLENLITADDVKNISRGRNAQKTHENIKSKLKNIMDRHQFLARIAKELDNALGDIMLLYYIFGAIIICLAMFTAIAVDDLYKTVRYFFMCCYLLWEVFFQCVNGQILSDHSLELSTAIYTADWIYADKDTKTMLHMLMARAQKPFLYTAKGYTTMNLNSFSGICKISYNLFNLLRTAYS

>CsupOR36

GHRRVTPIVTVRFCSKMDIPAFEELVKQIKINFWLIGIPFNDVKLHFRFYLLLFSLMIMVAEECGFLFVEYSPENLLEITELTPCTCIGILSALKIISITPYRHKIFKLTESLNELYSETLENQAAKKLITKKIILMKNLVTYYFVLNVVLVSVYNFSSVVIMSYTYIKTGKTVFYLSYAILVPFSIDTWPTWSLAFIHAISSGYICVLLFTTIDALYYVLSLHICNNFSLLTEDIRCLNETNSQNIRDIVKKHQHLLKLSEDL

>CsupOR37

GVLYKSSGKLVMQIPFVAWYPFDETDIRYWPIAYFHQLWAGFFDASSVHGSDSFYSLSCVFLQIQFKTLQYDIEQIIPEETNINTPELYKSFRKRFMLIVMRHQELIRCVNVLEVIYSKSNLCIIAVSSIVICISAFNFTTSDDIIWRTIFLGFFIMWLLQVFSLCYYSNLISLSSTEVSNAIYNSYWYKANAEVMKDLLFVLRRGQKPCKLTAWGYSDLNLAVFSKIVSTSWSYFALLQTMYSE

>CsupOR38

HSRHVTYFFWALAFFSNFSIFSEPYQKNINSDNGDPVFKKIFDGYIPYSDYPPGYYISMFIQTVLGNIVSAYVVGWDTLICTIMIFFAGQLKVARLLCSRVINVQNPELCRKYIADCHRFHTTLVKNQKLFENLISPAMFVYLIVISVNLGVCIIEIAKIKNDTPTLISSCLFLLDCFIQLLLFYWHSNEVTEDSVLVSYGVFESDWYQAENKYQREVA

>CsupOR40

MSEETELYETLLKKVDTFLIVTGCYFDEADEKRNVVQRFLSRRYYCFNILMLTSHLICDFAWLALEHKTASLIELTYFIPCVTLSMLACAKSYLLVKNGNHVTDLIKSTKKLKAISGRFE

**IR:**

>LstiIR7g

LFIMKPKIEICLQWLLMCCCTFVHGDRTIGAIFDDGTFLMEAAFSVAVTAASEGQENPFEAKVIRTSPGDLLEAEQAMCSLLENHIFGVFGPKTKGLIDHIQSIADVLELPQILTEPVETQNRNWSAVNLYPNHIAYSQVFADIVEMKGWEEFTIIYESSEL

>LstiIR64a

MDFNYFLNFISIAEISLIIDLLKFKDIGNVVNINCDTQKSIFLHKMFNDNTIHAAYWNINSDKNEIPNSYRKTGIILNASCSNWAQAFNNISDYSIFKNQFIWFVLTEDFLSTVRSLSSYPIEIDSDVTVVHKTDGFYKLYEVYYRNYSNGVLSIREIGYWDTFLRVNSSNRNDLHGLVMRCPVVVTDKVVQETFEEYLSKPKKNQVDSLHKLKFFALLNYIRDMYNISYDIQRTNSWGYNTRNGSFDGVVGALHRKEADIGGSPLFFRADRAELIDYIAETWQSRQCFILRHPKHPGGFYTIYTRPLTARVWCCILVMLVFSAVILCLMLKTKIPPSSDDSADSSFSLALLFIWSAICQQGMSVNRSSTSVKMVVLVTFVYAVTLYQYYNATVVSTLLRESPKNIRTLEDLLQSNLKAGAENVLYAKDYFKRTTDPVALRMYHKKIAPSHQFNFYAPARGMSLVKKGGFAFHVDSVVAYRIMRKTFTEREICEAHEVLLYPPQKMGMVIRKGSPYKEHFTYGIRKIYESGLMHRLKSVWDEPKPACVRTPDSSVFSVSIIEFSTALFALVAGNVLAILVLLCEIVMHRCGTNNHIAFFTLGSVLVL

>LstiIR76b

MATGIELIISSICNATFCQPVYDNPLLEKQASSSIDQYRDLIKEINGKHLKIGTYNNRPISWVERGEDGALIGRGVSFVLVDILQKRFNFTYEVVVPEKNFEIGGTKPEDSLIGLVNNSLVDMAAAFIPKLTRFHEMVRFSYDLDEGVWVMMLSRPKESAAGSGLLAPFNNAVWYLILVAVLSYGPCITLLTKLRSKLVPDGEKYIPMSPSFWFVYGAFIKQGTNLAPEANTTRVLFTTWWIFIILLSAFYTANLTAFLTLSKFTLDIESPQDLFKKNTRWVSAEGGAVQYVVSSPNEDIYYLSRMIATGRAEFRSMNSLYEFLPLVSGGAVLVEERIGIDELMYGDYQQKAREGVAEAERCTYVVAPNLFMSKLRGFAYPKNSQLAPLFDTVLTYVLQAGIVDYLEHRDLPSTKICPLDLQSKDRQLLNSDLYMTYMIMVTGLSAAVAVFIGEIMIKRYVIKDSKPKKPKRKKTKYEKNRHINSYDDTRPPPYDSIFGRSPKIKVNDTTKTKIINGREYLVIDAANGDTRLIPLRTPSAFLFRLDR

>LstiIR75p

ALSANRVAFFEPIKFSGRVMTWVFFAALMLLYAAYSANIVVLLQAPSNSIRTLAQLAASTVTLAANDVDYNHFVFSLYKDPDRVRIYKRVDPEKGKGKGQFYDINEGVERIRQGLFAFHSIVEPVYRRIEETFLEMEKCDLAEVDFMNGFDPFIPVKKDSPYLELMRVAFKQIREAGVQSALVRRVHVPKPRCASEVSAFSSVGVRDLKPVLLFMLYGIAASVAIAVIEILIFKLHRQRRFQLRR

>LstiIR75p.1

HTWGYRDKNGNWQGIVDNLIKKKADLGTLTIFTQERMQVVDYIAMVGSTAVRFVFREPPLSYISNIFTLPFTGAVWLAIVVCVLGCSVFLYITSKWEATMSMHQFQLDGSWADVIILIIGAVLQQGCTLEPRYAAGRSVTLLLFLALTILYAAYSANIVVLLRAPSSSVRTLPDLLNSPLKLGASDFEYNRYFFKKLKDPIRKSIYDKKIAPKGKKPNYYSMEEGVEKIRKGLFAFHMELNPGYRLIQETYHEDEKCDLVEIDYINEIDPWLPGQKRSPFKDLFKINFIKIRESGIQACIHHRLHVPKPKCSGTVSTFSSVGITDMYPAMLATLYGMLLAPAVLLLEIAYHRLTVLRKRKIKTRKWKSKLDHF

>LstiIR68a

MFKVVVLCILLANTAADISPILRSLNERKDLELVLVDLINGLSRREDITCVAFICDAVYLNVFEGYLFKRTDAAPYVMIVVEDYEDLLSPNFDTLESLRETRKDGCNVYVILLANGLQTARLLRFGDRYRILDTRAKYILLHDYRLFHSDLHYLWKRIINVIFLRYHSKINGVRKSKAWFDLSTVPFPNPIKSVFVSRRVDIWNNGRFHYNRTLFADKTSNLNNETLNVVYLDHVPSVVVTKTNDTSKVGGVEIEILNTLAEKMNFLPKLYQPINADLHKWGQKQANGSFSGLLGEMVNGQADVALGNLQYNPYHLELTDLSIPYTTQCFTFITPEASTDNSWKTLILPFKLNMWIAVLLVLLLSGTIFFGLARYYMHLQEFKKTHDKRKNITDKQKMQIEELDSDEKPAGLYLFGEIINSILYTYGMLLVVSLPRLPTGWSIRLLTGWYWLYCILLVVSYRASMTAILANPAPRVTIDTLKELVESKIACGGWGMETKKLFEDSADDIKTIGQRFEIINDPFEAANKVAKGAYAYYDNQDFLKYIRVKRKNVEMSIESEMVNGTSNLTDASSDVGIERNLHIMSDCVVNTPISIGFHKNSPLKPLADIYMRRIVEVGLVEKWLNDAMHPIKSLETNEEEIKALMNLKKLYGAFIALAIGYLISCIGLIGELIHWHLIVKRDPKFDKYALDLYYMNKNKKQ

>LstiIR75q.2

MKVSITLVSLFFLNICNAKTDSVVLVVADVIRAMEKPSTVVATLCWQTNKKVDFYNAVTSSNDRSRVATARLVDMKYVKRDYGQDQHIVFVADLSCPNISDYFVMKKEEQYFRSPFRWILISRNENDDIVPNEISHIDLLPDSEVIVLRQVGDDSYDLHFIYKISPGSYWRTQFYGTWNHEKRFVKSNQQIVESTALQRLDLLGYEMSICYVLTDKDSINHLTDEVNDHIDTITKVNFPTTNHLLDFLNATRKFIFADTWGYRVNGSWNGMTGYLLREEVEIGGSPMFFTSERISVVDYIASPTPTRSKFVFQQPKLSYENNLFLLSFRTSVWYSSAGLIFLLLLALFAVAAWEWKKNAHDIYRKEDSGTLRASFDDVIILIFGAICQQGSPVELKGSLGRVVMLILFLALMFLYTSYSANIVALLQSSSSQIRTLDDLLHSRLKFGVHDTVFNRYYFSTATEPVRKAIYEKKVAPPGTTPRFMSMEEGVKKMRKGLFAFHMETGVGYKFVGKYFNEGEKCGLREIQYLQVIDPWLAVRKNTPYKEMFKIGMKRIQEHGLQARENRLLYEKRPKCSGRESNFVSVSMVDCYPALLILSYGCLVSVIFLVFEFLFHQKQTIIQKLTHCNRVKTSRSAFTN

>LstiIR41a

MIHAVRMLPNPISFLPIEILLSTIFHQYLNSSYCLSLVSDSPLNIPIKNSFTYISPEDGELFVNQLLDVSEMGCSDYIVRMQDPKKFMAAFEKVNHLGNVRRSDRKIVFLPPEEDNKTRIDLLEVLALKESGFVANILLVLPTTELSLCSYYDLVTHKYVGPDDQVDEPYYLDRWNSCSSKFEKNANLFPHDMSNLQGKTVKVACFTYKPYALLDLDPSKEPLGRDGTEVRIVDEFCRWVNCTIRIVRDDEHEWGEVYENHTGVGVLGNVVEDRADLGITALYSWYEEFLVLDFSVSGIRTAITCVAPSPRLLASWEMPLLPFSWYMWLALFVTFIYSSLALTVAKGFSMDKVFLTTFGMMITQSQADVGASWRVRSITGWLLLTGLVLDNAYGGGLASVFTVPKYEKPINTVQDIVDRGIDWGATHDAWVFS

>LstiIR21a

FRGGDAVSKEVEMGRADIAVAGMYLTSDRIKEMDMSFSHSQDCAVFITLMSTALPRYRAILGPFHWHVWVALTFTYLIGILPLAFSDKHTLRHLLHNSGEIENMFWYVFGTFTNCFTFVGKNSWSKTTKITTRLLIGWYWIFTIIITSCYTGSIIAFVTLPIFPETVDTIEQLIAGFYRVGTLDHGGWERWFFNSSDPKTNKLFKKLELVPNVESGIRNTTKAFFWPYAFLGSQAELEYIVQANFTVTKSKRAMLHISHECFVPFGVSMGFPTNSLYSAKLSSDLRRMFQSGIIDKIVDEVRWEMQRSSTGKLLSAGSESLKITNAKEKGLTLEDTQGMFLLLGAGFLMGASALVSEWMGGITRRCRIGKKKPSSANSKQELISTPEIENEVKVITDATESRINFDNRSCSSSAGSRDTLESQVINVTEESIEVHESLDAARWDSRRSSSVDLDREVQEIFEKDLRRRKIVTDDIDEVIDEKREPTASHGAFGDRLK

>LstiIR7d.2

MLDWMQKHQFDNTGRFIVTCQSERPEDCDETKAVDIFWNHKIINVVFMKQTPDEEVNGYTYYHPDENCRSSQPVKIKTLRCTKYNNTTPCLGIFPKKMKNLHWCPIIVSTFKQMPYMSITDEGVPYGADGDLLLLIAEGLNATLKVMTPRRGFGWGNLDADGVWVGSLADVFDDVANFSMTSAAVTLSRFTYFQMSTAYYTSNVVWITRPAEKKPASLKLFYPFQTLSQIALAFSFIFVGLCVWLVNSKYWPWHNSTEEASKLSSVLFYSWMICMGLPSTKLPAKKEFICLVLIWIWYCFLIRTFYQVYLINSLQGEFYSDEIDTIEDAYFANYSFGGGPALRDYFIDHPLVYDNWKNLNASDIIPTLINLTEKKFVLATNQATTESIIKEHNLIVHILPQKVINSPSVIFSKKFSPLVEPLNIILRRLLESGFTDKLYKNYATQNTWKSDNPEEPITLEHYTGCYVILVVGWVVSTLIFIIELFYTKLQKCKYYIIKMKPRLGCQII

>LstiIR93a

MRIWLLVFCIVQVSGEDFPSLITANASIAVVLDREFLGEQYQAILDELKDYIKELARVELKHGGVVVHYYSWTSISLMKGFLAVFSVASCEDTWSLFSRTEKEELLLFALTEVDCPRLPTDSAITVTNVVPGEELPQILLDMRTEMAFKWKSAVILHDDTLSRDIVSRVVQSLIMQIDKGASTSPVSVVVYKMKHEINEYLRRKEIRRVLSKLPVKHIGENFMAIVTTEVMTTMSEIARDLVMTNTHAQWLYIISETDAQNGNLSSLINALYEGENVAFIYNVTDNGPECKNGLMCYCQEMMNAFISALDAAVQDEFDVAAQVSDEEWEVIRPNKVQRRSMLLKHMQQHIATKSSCGNCSTWRALAADTWGATYRSYGDADLVAKDTTNGTMTGAIEHVDLLQVGYWRPIDALRLDDVLFPHVEHGFRGKDLPIITYHNPPWTILQVNESGSVVSYTGLIFDIVNQLAKNKNFTVKVILPSHVKHLVANDTSADMSHSQDAMLTLSAVAKGQVAIAAVAFTVLSDPPPGINYTVAVSTQTYCFMIARPRELSRALLFLLPFTTDTWLCLGLGVILMGPTLYIIHRLSPYYEAMEITRQGGLSTIHNCLWYVYGALLQQGGMYLPRADSGRLVVGTWWLVVLVVVTTYSGNLVAFLTFPKQEIPVTTIEELLKNQQIYTWSIQKGSYLELELKNSDEPKYTALLKGAELSNTGGTMESNLSSWKKQLIRIREQRHVIFDWKLRLSYLMRNEHKLTDRCDFSLSVDEFIDEQLAMVLPAGSPYLPVINKEINRMQKAGLISKWLYAYLPKRDRCWKTSSISQEVNNHTVNLSDMQGSFFVLFLGFFSASFVLFLEWFCNRRKRRSEEVIIKPYVE-

>LstiIR7d.3

NEHEDTMVVKILFLIGVLVGCGFAEEYRIGGIFYEDAEDLKVAFALSAKMLNFTPSIREVSKRGEILEVRQQVCSLAEESVIGIIDGIGGRGSEIIQSLCDSMELPHVLIRYDFTYSSDWSLLNLYPSPIVYNKVVEQMVLRKEWKNFTIMYIRGRSLFRLHNLLQMGNDTDNYAIGVRELSGKDYRDVLINAKENGYTNFVVDCPSRHLEQLLLQAQQVGLMADEHSYIILSPDLFTLDMERYRYGGVNMTGFRLMDLKASENLWEFTEHYNAETGKTMLPEQLKTELLLVYDAIQVFAGALQKLKGVEAQPLNCENYDAWMYGSSLLNFMKTNKVEGLTRTLLFDGLGERTDVIFDVLELTSSGNQTIGTWKHNKLDIQRPFVPDAELTENTALKNKTLKVLITTTGPYGYLKHSDQKLEGNDRYEGFSIDLIEKISEILGFSYEFEVEEDNGKLVGGKWTGMPRKLMDDEADLAICDFTITALRQSAIDFSTPFMSLGIGILYKEPSKQPPEMFSFMAVFSKEVWYYMMLIQMMLGGVMIFVGRISHKEWQNPVPCIEQPEELSNQFSFANSVWLIIGSVMQQGSEIAPIAVAPRMITSIWWFFTMVMVASYVGTLVAFLTVEKNVLPFQNVEELFRHKSISYGAKKGGSTLDFFMQSKNEIYQRMYQKMTSKGWLVGNNADGVNLVENSTYAFFMESTSIEYTMERHCDLLQIGGLLDQKSYGIGMKKNSTLKVYIDRALLILKERGEIQKLKDIWWKEKRGGGKCGEKHAVEEKQLGMKNMLGAFVVLGVGCGLGVIISTLDMLWGVFKRSVKYGTTFKYELVEELKFALKFSGDVKPVKRPQKTMEGSFEALAEAEGKDEVKSLKSHQSGRSTSTHKTHHSHSSRHSSRGLSVAFARQREYS

>LstiIR87a

MCTKIFLQHLFFALYVSAAINENPLLTTTGNSEQTAKTAECVLKLSAKYFVEKKALSGSIVIININSYVSTTQVLLLQTIHGGIKYSVMVKDSFYPHANASHFPEKAKNYMLILEEKSELTRNILQLNKLPTWNPLAKAIVFYQLNKTEDAEETAIEFINELRHYKLFKSIIFIYSPEEKEVVSYTWVPYSDMNCGGKCDSVYILDKCKDNVIYQLATQKEMFPSDMKGCPLVAYAIVSEPYVLPPEMKLTNTSYNDAYVFQKGGEINLVKIITQFTNMSLIMRTSELPENWGTIYWNGTATGAYEVLRNDSADLVIGNIEVTRTIRRWFHPTVSYTQDEMTWCVPKAGQASTWNNLVIIFQWSTWVATFGSLVIMGLVFHYMYYRENGKKVTKWPTNSMLMTFSMLLGWGASFEPKSATFRILIFGWLCFSVNMGISYESFLRSFLMHPRFEKQISTETDLIQSRIPLGGREIYRSYFETNNASSFYLYRKYNSTTFAEGIRRAAKDRNFAVVSSRRQAAYADQKLGKGKPLIYCFPESNNLYKYGVVLLARKWFPMIERCNTIIRSVSENGLIDKWNRELLIHIGNGEGTSEIEPLSIQHLLGAFIFVGIMYAASILVFIFEVSIGVYEKWKIRKNLQPDNRHVRFHFAK

>LstiIR1

MWFLWLLLSFTIQQCTPQFIFSEKKDISYQIAGIFLSDAQTQMLAFNETMASAGLDQFHLSPAILQPTSKDSLAIWNELCSNRNLRPIAIVGPQDPKWDGIVRDQCALANIPHVQASWQPLDADLELNDEEEEEVSEGENEEEQKEVSFKKLSINFYPNSEEISLAYAALLKYYHWENFAVLYEDEYGLLRIQKILAEHTTNFPVIVRKLEPEMDNRNVFKELSKHQLNRIMLDCNASRILDYMKQASDLKMVNYYQHYILVTMEAYIVAEQLTHYHSNITWLSLTEYDKLKDAQHVLAPMVGKWITRQMIPPPPVTDFPNEALIMNDIANHLLRAMQTIKDKITYFKPRAPICDADAQPWRYGALFQNAILTTPSYGVTGNIAFDEKGRRFNYTLYVNEIHVNKLQTIGTWASTNGTEILEDRPDSGTLDQQQSSKHFVVISTKAKPWFYDKTPCVGEEECENDDGLKYEGFSVDLIKEIFRFLREEKFNYTFEFMDGGDKKAGTVDETTKKWTGLIGDLLDNVADLAVCDITITEERKKMVDFSVPFMSLGISILFTQKKDPELELFSFLNPYTFEVWMYTATAYCVVSVILFICAR

>LstiIR40a

MYNLLLGESNEMKLLPFYLFLNTAHCFIDIQDIVSETMTKLPKDFAVAIKDIAEGLPAKTITVVRGESTKIRSQDIFQLLCLLSEHNIPVINLDITTKQSKDKYYSFVKKALDVSEERTSLILCEPYECENILTELTDNNLIHRTILYIFYWPYGKVSDQFLNTMKEAMRVAVLTNPRESVFRVYYNQATPDRLHHLSLVNWWSGSLYKSPVLPPAEKVYQDFKGRIFDVPVLHAPPWHFVKYNNDSTVNVTGGRDDKLLSLLSKKLNFRYRYYDPPDRSQGSSISGNGTFKGTLGLIWKRKADFFIGDVTMTWERLQAVEFSFLTLADSGAFLTHAPAKLSETLAIIRPFRWEVWPLVCATVLVTGPALWVVIAAPSLWQKRQRDQLRLLNNCCWFTTTLFLRQSSSKEPSKTHKARLVSVLVSLGATYVIGDMYSANLTSLLARPARERPIGTLPALEEAMRERGYELVVERHSSSLAILENGTGVYGRLARLMRRQRIQRVRSVEVGVRLVLTRRHVAILGGRETLYYDTERFGSHNFHLSEKLYTRYSAIALQIGCPFLETFNNVVMTLFEAGILAKMTTDEYKNLPEQSRRSEPVTESDKPNNEITGDSPSASQSGTTQGESTKALEPVSLRMLRGAFCLLGIGHLLAAIALGVEIQIHRRSKNFIKIMEPNGGKNMPRMRALKKASKCVRQGVRRVVRAVCRSIDRALGPGVQ

>LstiIR8a

KMDVPLLFLLIFLINLGCIVSELSLRFVFIIEVHDSDLAHQIGRALKVAEDQRTGVRVSDAVVQLDRENEEESYRRLCSALSKGTSLVVDLSWAPWDMAQQLCSDAGLPLVRAALGSQQLLAALDQYLESRNATDAAILMESEGEVDKTLYELLGRSNVRLWVHAGLTRDSAKALKSMRPEPSFYVIVGESGFVMDTYRRAVKEKLVRRDYRWNLVLTDYSGDTIDVAQLVLPTMILHVDQVECCRLLGLREECSCPSDLKRKQYILNALLIYITETFSKLERELPVVSTRVDCDNVQGSEMNGTRDRILRQFSEDTEMNNDTIFFWDDERSGLFLRSNFILSTYKPDAGLETVATWSANEEYKLLPGATLEPLKLFFRIGTSPAVPWTLPKIDPETGEPEVNEDGQPVYEGYCIDLIAKLAETMEFDYEIITPKSGNFGKKLPNGSWDGVVGDLMRGETDLAVAALTMTAEREEVIDFVAPYFEQTGILIAIRKPIRKTSLFKFMTVLRTEVWLSIVAALVLTGLMIWLLDKYSPYSARNNPQAYPYPCREFTLKESFWFALTSFTPQGGGEAPKALSGRTLVAAYWLFVVLMLATFTANLAAFLTVERMQTPVSSLEQLARQSRINYTVVEGSTIHQYFINMKFAEDTLYRVWKEITLNATSDQAQYRVWDYPIREQYGHILLAINASGPVPDAKTGFEQVNEHTDADFAFIHDSAEIKYEVTRNCNLTEVGEVFAEQPYAIAVQQGSRLQEHLSRALLDLQKERFLEQLASKYWNESARQACPDADESEGITLESLGGVFIATLFGLGLAMITLAWEVFYYKRKEKNKVQGFDSKIEKAAFVDTKKKLEKVGVRLRKKNKSGKVGKVDVIGKGKGVTIGDSFKPASEKMGVSYISVYPKGEYRP

>LstiIR25a

MGKKYMRLLLKVLLLFSFVRAAIFQTTQNINVLLINEENNALAEKAFEVAKEYVRRNPSLGLAVDPVIVVGNRSDAKAFLENVCRKYNDMLSAKKTPHVVLDFTMTGVGSETIKSFTAALALPTISGSFGQAGDLRQWRSLDANQTRFLLQVMPPADILPESIRAIVTKQDITNAAIIFDELFVMDHKYKSLLQNIPTRHVITPVKSYNKDEIKTQLRSLRELDIVNFFVVGSLRTIKNVLDAADENQYFGRKTAWFALSLDKGDITCGCKDATIVYMRPTPDAKSRDRLGKIKTTYSMNGEPEITSAFYFDLSLRTFLAVKSLLDSGKWPNDMKYITCDDYDGKNTPNRTLDLKAAFQEIKETPAYAPFYIPEDDPLNGRSYMEFNTDLTAVTVKDGASIGSRVLGSWKAGLSNPLSLTDPENMSDYSAQLVYRVVTVEQEPFIIRDDEAPKGFKGYCIDLIEEIRQIVKFDYEIVVSPDGNFGTMDENGNWNGIIKELIEKRADIGLTSLSVMAERENVVDFTVPYYDLVGITIMMKLPRTATSLFKFLTVLENDVWLSILAAYFFTSFLMWVFDKWSPYSYQNNREKYKDDEEKREFTLKECLWFCMTSLTPQGGGEAPKNLSGRLLAATWWLFGFIIIASYTANLAAFLTVSRLDTPIESLDDLSKQYKIQYAPLNGSAAMTYFERMAHIEVKFYEIWKEMSLNDSLSDVERAKLAVWDYPVSDKYSKMWQAMKEAGLPNSIEEALQRVRDSKSSSEGFAWLGDATDVRYHVLTSCDLQMVGDEFSRKPYAIAVQQGSPLKDQFNNAILQLLNKRKLEKLKENWWTNNPKSMKCEKQDDQSDGISIQNIGGVFIVIFMGIGLACITLGVEYWWYKIRKRSTIGDVTQVEPAKSSRNHTDIKGEGFTFRSRNFGLSNLKPKF

>LstiIR75d

MDTISLIPAFFLSKNIYFLTTFLCWNSEELHKLWRLGQQQGLRVRAMAAGPATPPLPPDDLHREGVVLDLACPYADHIIQAASETRGFNYRYAWLLLHNSSFDATSLDSVLSGSVILPDADVTFASDDKLLDVYRIKADQPLLATTLGVVRNSTRRDLEQMWGVLKSTVSRRKNLNNVFLKGATIITQPQNFKGWNDLTVRHIDTFPKLMYPLLMHCAEDLNFRLNLLQVELYGDERNGSFDGLAGMLQRRDIEVGVTTLFMRHDRLNVMHFCSETLELKGAFIFRQPPQSSVNNVFLLPFSRGVWAASALVFTAAGGLLAALSRPRWLRDADPDLVQLSAAEAFTFAVGTICQQGCYVNPHAVSVRMLMFFTLLASLFTFTSYSAKIVAILQTPSDAIQTIDDLTHSPMALGVQESTYKRVYFAESDDPATQRLYRRKLLPQGERAYLSIVDGIARVRNGLFAFQVEESSGYDVISKTFTEQEKCGLKQIQAFKLPMVAVPILKHSGYRELFAARLRWQRETGIMDRERRVWMASKPRCDSDGGGFLSVRLSDVLPAVQVLIYGMLLAAIQLFAEIALHRATERIKRKNKLRGKRE

>DmelGlumsy

MHSRLKFLAYLHFICASSIFWPEFSSAQQQQQTVSLTEKIPLGAIFEQGTDDVQSAFKYALLNHNLNVSSRRFELQAYVDVINTADAFKLSRLICNQFSRGVYSMLGAVSPDSFDTLHSYSNTFQMPFVTPWFPEKVLAPSSGLLDFAISMRPDYHQAIIDTIQYYGWQSIIYLYDSHDGLLRLQQIYQELKPGNETFRVQMVKRIANVTMAIEFLHTLEDLGRFSKKRIVLDCPAEMAKEIIVQHVRDIKLGRRTYHYLLSGLVMDNHWPSDVVEFGAINITGFRIVDSNRRAVRDFHDNRKRLEPSAKAKARTQGGPNSLPPISAQAALMYDAVFVLVEAFNRILRKKPDQFRSNHLQRRSHGGSSSSSATGTNESSALLDCNTSKGWVTPWEQGEKISRVLRKVEIDGLSGEIRFDEDGRRINYTLHVVEMSVNSTLQQVAEWRDDAGLLPLHSHNYASSSRSASASTGDYDRNHTYIVSSLLEEPYLSLKQYTYGESLVGNDRFEGYCKDLADMLAAQLGIKYEIRLVQDGNYGAENQYAPGGWDGMVGELIRKEADIAISAMTITAERERVIDFSKPFMTLGISIMIKKPVKQTPGVFSFLNPLSQEIWISVILSYVGVSFVLYFVTRFPPYEWRIVRRPQADSTAQQPPGIIGGATLSEPQAHVPPVPPNEFTMLNSFWYSLAAFMQQGCDITPPSIAGRIAAAVWWFFTIILISSYTANLAAFLTVERMVAPIKTPEDLAMQTDVNYGTLLHGSTWEFFRRSQIGLHNKMWEYMNANQHHSVHTYDEGIRRVRQSKGKYALLVESPKNEYVNARPPCDTMKVGRNIDTKGFGVATPIGSPLRKRLNEAVLTLKENGELLRIRNKWWFDKTECNLDQETSTPNELSLSNVAGIYYILIGGLLLAVIVAIVEFFCRNKTPQLKSPGSNGSAGGVPGMLGSSTYQRDSLSDAIMHSQAKLAMQASSEYDERLVGVELASNVRYQYSM

>DmelNmdar1

MAMAEFVFCRPLFGLAIVLLVAPIDAAQRHTASDNPSTYNIGGVLSNSDSEEHFSTTIKHLNFDQQYVPRKVTYYDKTIRMDKNPIKTVFNVCDKLIENRVYAVVVSHEQTSGDLSPAAVSYTSGFYSIPVIGISSRDAAFSDKNIHVSFLRTVPPYYHQADVWLEMLSHFAYTKVIIIHSSDTDGRAILGRFQTTSQTYYDDVDVRATVELIVEFEPKLESFTEHLIDMKTAQSRVYLMYASTEDAQVIFRDAGEYNMTGEGHVWIVTEQALFSNNTPDGVLGLQLEHAHSDKGHIRDSVYVLASAIKEMISNETIAEAPKDCGDSAVNWESGKRLFQYLKSRNITGETGQVAFDDNGDRIYAGYDVINIREQQKKHVVGKFSYDSMRAKMRMRINDSEIIWPGKQRRKPEGIMIPTHLRLLTIEEKPFVYVRRMGDDEFRCEPDERPCPLFNNSDATANEFCCRGYCIDLLIELSKRINFTYDLALSPDGQFGHYILRNNTGAMTLRKEWTGLIGELVNERADMIVAPLTINPERAEYIEFSKPFKYQGITILEKKPSRSSTLVSFLQPFSNTLWILVMVSVHVVALVLYLLDRFSPFGRFKLSHSDSNEEKALNLSSAVWFAWGVLLNSGIGEGTPRSFSARVLGMVWAGFAMIIVASYTANLAAFLVLERPKTKLSGINDARLRNTMENLTCATVKGSSVDMYFRRQVELSNMYRTMEANNYATAEQAIQDVKKGKLMAFIWDSSRLEYEASKDCELVTAGELFGRSGYGIGLQKGSPWTDAVTLAILEFHESGFMEKLDKQWIFHGHVQQNCELFEKTPNTLGLKNMAGVFILVGVGIAGGVGLIIIEVIYKKHQVKKQKRLDIARHAADKWRGTIEKRKTIRASLAMQRQYNVGLNSTHAPGTISLAVDKRRYPRLGQRLGPERAWPGDAADVLRIRRPYELGNPGQSPKVMAANQPGMPMPMLGKTRPQQSVLPPRYSPGYTSDVSHLVV

>DmelNmdar2

MLSILERYKWHQFSVVTSQIAGHDDFVQAVRERVAEMQEHFKFTILNSIVVTRTSDLMELVNSEARVMLLYATQTEAITILRAAEEMKLTGENYVWVVSQSVIEKKDAHSQFPVGMLGVHFDTSSAALMNEISNAIKIYSYGVEAYLTDPANRDRRLTTQSLSCEDEGRGRWDNGEIFFKYLRNVSIEGDLNKPNIEFTADGDLRSAELKIMNLRPSANNKNLVWEEIGVWKSWETQKLDIRDIAWPGNSHAPPQGVPEKFHLKITFLEEAPYINLSPADPVSGKCLMDRGVLCRVAADHEMAADIDVGQAHRNESFYQCCSGFCIDLLEKFAEELGFTYELVRVEDGKWGTLENGKWNGLIADLVNRKTDMVLTSLMINTEREAVVDFSEPFMETGIAIVVAKRTGIISPTAFLEPFDTASWMLVGIVAIQAATFMIFLFEWLSPSGYDMKLYLQNTNVTPYRFSLFRTYWLVWAVLFQAAVHVDSPRGFTSRFMTNVWALFAVVFLAIYTANLAAFMITREEFHEFSGLNDSRLVHPFSHKPSFKFGTIPYSHTDSTIHKYFNVMHNYMRQYNKTSVADGVAAVLNGNLDSFIYDGTVLDYLVAQDEDCRLMTVGSWYAMTGYGLAFSRNSKYVQMFNKRLLEFRANGDLERLRRYWMTGTCRPGKQEHKSSDPLALEQFLSAFLLLMAGILLAALLLLLEHVYFKYIRKRLAKKDGGHCCALISLSMGKSLTFRGAVFEATEILKKHRCNDPICDTHLWKVKHELDMSRLRVRQLEKVMDKHGIKAPQLRLASSSDLLNHHHLKERPPLLGNLSLAASAQDLYRWSYKTEIAEMETVL

>DmelGluRIA

MHSRLKFLAYLHFICASSIFWPEFSSAQQQQQTVSLTEKIPLGAIFEQGTDDVQSAFKYAMLNHNLNVSSRRFELQAYVDVINTADAFKLSRLICNQFSRGVYSMLGAVSPDSFDTLHSYSNTFQMPFVTPWFPEKVLAPSSGLLDFAISMRPDYHQAIIDTIQYYGWQSIIYLYDSHDGLLRLQQIYQELKPGNETFRVQMVKRIANVTMAIEFLHTLEDLGRFSKKRIVLDCPAEMAKEIIVQHVRDIKLGRRTYHYLLSGLVMDNHWPSDVVEFGAINITGFRIVDSNRRAVRDFHDSRKRLEPSGQSQSQNAGGPNSLPAISAQAALMYDAVFVLVEAFNRILRKKPDQFRSNHLQRRSHGGSSSSSATGTNESSALLDCNTSKGWVTPWEQGEKISRVLRKVEIDGLSGEIRFDEDGRRINYTLHVVEMSVNSTLQQVAEWRDDAGLLPLHSHNYASSSRSASASTGDYDRNHTYIVSSLLEEPYLSLKQYTYGESLVGNDRFEGYCKDLADMLAAQLGIKYEIRLVQDGNYGAENQYAPGGWDGMVGELIRKEADIAISAMTITAERERVIDFSKPFMTLGISIMIKKPVKQTPGVFSFLNPLSQEIWISVILSYVGVSFVLYFVTRFPPYEWRIVRRPQADSTAQQPPGIIGGATLSEPQAHVPPVPPNEFTMLNSFWYSLAAFMQQGCDITPPSIAGRIAAAVWWFFTIILISSYTANLAAFLTVERMVAPIKTPEDLTMQTDVNYGTLLYGSTWEFFRRSQIGLHNKMWEYMNANQHHSVHTYDEGIRRVRQSKGKYALLVESPKNEYVNARPPCDTMKVGRNIDTKGFGVATPIGSPLRKRLNEAVLTLKENGELLRIRNKWWFDKTECNLDQETSTPNELSLSNVAGIYYILIGGLLLAVIVAIMEFFCRNKTPQLKSPGSNGSAGGVPGMLASSTYQRDSLSDAIMHSQAKLAMQASSEYDERLVGVELASNVRYQYSM

>DmelGluRIB

MRFGLKLSCLWPSFLLWLTWSSGGGGGSGVGVSAQPSLTEKIPLGAIFEQGTDEVQSAFKYAMLNHNLNVSSRRFELQAYVDVINTADAFKLSRLICNQFSRGVYSMLGAVSPDSFDTLHSYSNTFQMPFVTPWFPEKVLTPSSGFLDFALSMRPDYHQAIIDTIQFYGWRKIIYLYDSHDGLLRLQQIYQGLRPGNESFQVELVKRISNVSMAIEFLHTLEQIGRFENKHIVLDCPTEMAKQILIQHVRDLRLGRRTYHYLLSGLVMDDRWESEIIEFGAINITGFRIVDTNRRLVREFYDSWKRLDPQMSVGAGRESISAQAALMYDAVFVLVEAFNKILRKKPDQFRNNVQRRSQTLMVAQAAASTSSDGYNYSASGGGGGNGGAGGGFAGSDSGGSGGMASRALDCNTAKGWVNAWEHGDKISRYLRKVEIEGLTGDIKFNDDGRRVNYTLHVVEMTVNSAMVKVAEWNDDAGLQPLNAKYVRLRPHVEFEKNRTYIVTTVLEEPYIMLKQVAFGEKLHGNNRFEGYCKDLADLLAKELGINYELRLVKDGNYGSEKSSAHGGWDGMVGELVRKEADIAIAAMTITAERERVIDFSKPFMSLGISIMIKKPVKQTPGVFSFMNPLSQEIWVSVIFSYIGVSIVLFFVSRFSPHEWRLVQQQPQQSQSPDPHAHHEQLANQQPPGIIGGAPLPAPPGPPTPGAQTAAGAAALQAALSAGSPGSGGSSSAVVNEFSVWNSFWFSLAAFMQQGCDLSPRSVSGRIAAASWFFFTLILISSYTANLAAFLTVERMVTPINSPEDLAMQTEVQYGTLLHGSTWDFFRRSQIGLHNKMWEYMNSRKHVFVPTYDEGIKRVRNSKGKYALLVESPKNEYVNAREPCDTMKVGRNLDTKGFGIATPLGSALKDPINLAVLTLKENGELIKLRNKWWYEKAECSTHKDGETSHSELSLSNVAGIFYILIGGLLVSVFVAILEYCFRSRDSRSASSGSGMGLGMGLGGGMSGGSLGKANGSMMLGPSSAVPGGMPSSHQRSTLTDTMHAKAKLTIQASRDYDNGRVGYLNCASLQYYPPAQLSATPPDAGDSLHMNAHGQVXHEHAQESRLXRTPCAPTIRRIMANSRQETEKMMGGGHGGTVTVSVPATCSSSMNGSHEGSLALSPSSGSATLMRHSPDINQHPYGK

>DmelGluRIIA

MRLCPVVIYAFIIIIGFLEGIIALGGDDRNEITVGAIFYENEKEIELSFDQAFREVNNMKFSELRFVTIKRYMPTNDSFLLQQITCELISNGVAAIFGPSSKAASDIVAQIANATGIPHIEYDLKLEATRQEQLNHQMSINVAPSLSVLSRAYFEIIKSNYEWRTFTLIYETPEGLARLQDLMNIQALNSDYVKLRNLADYADDYRILWKETDETFHEQRIILDCEPKTLKELLKVSIDFKLQGPFRNWFLTHLDTHNSGLRDIYNEDFKANITSVRLKVVDANPFERKKTRLTKVDQILGNQTMLPILIYDAVVLFASSARNVIAAMQPFHPPNRHCGSSSPWMLGAFIVNEMKTISEDDVEPHFKTENMKLDEYGQRIHFNLEIYKPTVNEPMMVWTPDNGIKKRLLNLELESAGTTQDFSEQRKVYTVVTHYEEPYFMMKEDHENFRGREKYEGYAVDLISKLSELMEFDYEFMIVNGNGKYNPETKQWDGIIRKLIDHHAQIGVCDLTITQMRRSVVDFTVPFMQLGISILHYKSPPEPKNQFAFLEPFAVEVWIYMIFAQLIMTLAFVFIARLSYREWLPPNPAIQDPDELENIWNVNNSTWLMVGSIMQQGCDILPRGPHMRILTGMWWFFALMMLSTYTANLAAFLTSNKWQSSIKSLQDLIEQDKVHFGSMRGGSTSLFFSESNDTDYQRAWNQMKDFNPSAFTSTNKEGVARVRKEKGGYAFLMETTSLTYNIERNCDLTQIGEQIGEKHYGLAVPLGSDYRTNLSVSILQLSERGELQKMKNKWWKNHNVTCDSYHEVDGDELSIIELGGVFLVLAGGVLIGVILGIFEFLWNVQNVAVEERVTPWQAFKAELIFALKFWVRKKPMRISSSSDKSSSRRSSGSRRSSKEKSRSKTVS

>DmelGluRIIB

MHGLQFLVLLALAIASGANEDTLVIKIGAIFFDTEMKLADAFSAALEEVNAINPALKLDAIKRYVTVDDSIVLQDISCDLIGSGVAAIFGPSSKTNSDIVEVLCNMTGIPHLQFDWHPQQSNRERMNHQLTVNVAPMELFLSAAFSDILASKTFDWKSFTIAYERSSHLIRLQHILAWKQLHKAGIKMQEFERGDDYRILWKRINNAREKFVLLDCPSDILVDVINASIGYNMTGSFNHLFLTNLDTHLSGIDGFYSRDFTVAVAAVRIRTYVPPPVHDEIDVFDNSVDTRFSSLGSQLVYDSIVLFYNALLEISQRPGFYIPNFSCGRGFWQPGPRLVEQMKQITPKMVKPPFKTQRLQINADGQREDFNLEVYNPIIDRVTHIWNKEFQLVDFEKLRENSTQALKQKRLQNKEDFSQKPIRYTVATRVGKPYFSWREEPEGVHYEGNERFEGYAVDLIYMLAQECKFDFNFEPVRDNKYGSYDANTDEWDGIIRQLIDNNAQIGICDLTITQARRSVVDFTVPFMQLGISILSYKEPPPKADIYAFLNPYNAEVWLFVMIAMMITAFALIFTGRIDQYEWDQPVENVNREMERQNIWHLSNALWLVLGSMLNQGCDLLPRGLPMRLLTAFWWIFALLISQTYIAKLAAFITSSKIAGDIGSLHDLVDQNKVQFGTIRGGATSVYFSESNDTDNRMAWNKMLSFKPDAFTKNNEEGVDRVKLSKGTYAFLMETTNLQYYVQRNCELTQIGESFGEKHYGIAVPLNADFRSNLSVGILRLSERGELFKLRNKWFNSNESTCDSNVPTIDDGQFDMDSVGGLFVVLIVGVVVGLVIGVAEFLWHVQRISVKEKIPPMLALKAEFYFVIRFWLTRKPLHTYRQSRDSTSTGYSSLEQITSASSAKKKKKTRRIEK

>DmelGluRIIC

MWQRILLLGCMWSAFFMCRSRGQQINIGAFFYDDELELEKEFMTVVNAINGPESEQTMRFYPLIKRLKPEDGSVTMQEHACDLIDNGVAAIFGPSSKAASDIVALVCNSTGIPHIEFDISDEGIQAEKPNHQMTLNLYPAQAILSKAYADIVQNFGWRKFTIVYDADDARAAARLQDLLQLREVHNDVVRVRKFHKDDDFRVMWKSIRGERRVVLDCEPNMLVELLNSSTEFGLTGQYNHIFLTNLETYTDHLEELAADNETFAVNITAARLLVNPDPPPYSLPYGYVTQRDNIVYESSDPPRTLIHDLIHDALQLFAQSWRNASFFYPDRMVVPRITCDFAASGGRTWAMGRYLARLMKGTSGVNNTNFRTSILQFDEDGQRITFNIEVYDPLDGIGIAIWDPRGQITQLNVDVKAQKKMIYRVATRIGPPYFSYNETARELNLTGNALYQGYAVDLIDAIARHVGFEYVFVPVADQQYGKLDKETKQWNGIIGEIINNDAHMGICDLTITQARKTAVDFTVPFMQLGVSILAYKSPHVEKTLDAYLAPFGGEVWIWILISVFVMTFLKTIVARISKMDWENPHPCNRDPEVLENQWRIHNTGWLTVASIMTAGCDILPRSPQVRMFEATWWIFAIIIANSYTANLAAFLTSSKMEGSIANLKDLSAQKKVKFGTIYGGSTYNLLADSNETVYRLAFNLMNNDDPSAYTKDNLEGVDRVRKNRGDYMFLMETTTLEYHREQNCDLRSVGEKFGEKHYAIAVPFGAEYRSNLSVAILKLSERGELYDLKQKWWKNPNASCFEEPDPDATPDMTFEELRGIFYTLYAGILIAFLIGITEFLVYVQQVALEERLTFKDAFKKEIRFVLCVWNNRKPIVAGTPISSVRTTPRRSLDKSLDRTPKSSRRVVIGRSSEEMREMAQGSGSSSGSNNAGRGEKEARV

>DmelGluRIID

MHFCWISLIILSLSRVQAQFYGGNAYEASSGQSIRLGLITDDATDRIRQTFEHAISVVNNELGVPLVGETEQVAYGNSVQAFAQLCRLMQSGVGAVFGPAARHTASHLLNACDSKDIPFIYPHLSWGSNPDGFNLHPSPEDIANALYDIVNQFEWSRFIFCYESAEYLKILDHLMTRYGIKGPVIKVMRYDLNLNGNYKSVLRRIRKSEDSRIVVVGSTTGVAELLRQAQQVGIMNEDYTYIIGNLNLHTFDLEEYKYSEANITGIRMFSPDQEEVRDLMEKLHQELGESEPVNSGSTFITMEMALTYDAVRVIAETTKHLPYQPQMLNCSERHDNVQPDGSTFRNYMRSLEIKEKTITGRIYFEGNVRKGFTFDVIELQTSGLVKVGTWEEGKDFEFQRPPQAVNFNDIDDGSLVNKTFIVLISVATKPYASLVESIDTLIGNNQFQGYGVDLIKELADKLGFNFTFRDGGNDYGSFNKTTNSTSGMLKEIVEGRADLAITDLTITSEREEVIDFSIPFMNLGIAILYVKPQKAPPALFSFMDPFSSEVWLYLGIAYLGVSLCFFIIGRLSPIEWDNPYPCIEEPEELENQFTINNSLWFTTGALLQQGSEIAPKALSTRTISAIWWFFTLIMVSSYTANLAAFLTIENPTSPINSVKDLADNKDDVQYGAKRTGSTRNFFSTSEEPIYIKMNEYLNAHPEMLMENNQQGVDKVKSGTKYAFLMESTSIEFNTVRECNLTKVGDPLDEKGYGIAMVKNWPYRDKFNKALLELQEQGVLARLKNKWWNEVGAGVCSAKSDDDGPSELGVDNLSGIYVVLVIGSIISIIISILCWCYFVYKKAKNYEVPFCDALAEEFRIVIRFSENERPLKSAQSIYSRSRNSSQSIESLKTDSEENMPVED

>DmelGluRIIE

MFFNHFVILWSLFSIHISVNWAQYENFGGYDNYQSLESVPIGLLTDQNTEQMNIVFDHAIDVANQEVGTSLTSLKEEVNYGDAYQSYGKLCRMLETGIAGVFGPSSRHTAVHLMSICDAMDIPHIYSYMSENAEGFNLHPHPADLAKALYSLITEFNWTRFIFLYESAEYLNILNELTTMLGKSGTVITVLRYDMQLNGNYKQVLRRVRKSVDNRIVVVGSSETMPEFLNQAQQVGIINEDYKYIIGNLDFHSFDLEEYKYSEANITGLRLFSPEKMAVKELLMKLGYPTDQDEFRNGSCPITVEMALTYDAVQLFAQTLKNLPFKPMPQNCSQRTESVRDDGSSFKNYMRTLRLTDRLLTGPIYFEGNVRKGYHLDVIELQPSGIVKVGTWDEDRQYRPQRLAPTTAQFDSVDNSLANKTFIILLSVPNKPYAQLVETYKQLEGNSQYEGYGVDLIKELADKLGFNFTFVNGGNDYGSYNKSTNESTGMLREIMTGRADLAITDLTITSEREQALDFTIPFMNLGIAILYLKPQKATPELFTFMDPFSEEVWWFLGFSFLGVSLSFFILGRLSPSEWDNPYPCIEEPEELENQFTLGNSIWFTTGALLQQGSEIGPKALSTRTVASFWWFFTLIVVSSYTANLAAFLTIEKPQSLINSVDDLADNKDGVVYGAKKTGSTRNFFMTSAEERYKKMNKFMSENPQYLTEDNMEGVNRVKTNTHYAFLMESTSIEYNTKRECNLKKIGDALDEKGYGIAMRKDWPHRGKFNNALLELQEQGVLEKMKNKWWNEVGTGICATKEDAPDATPLDMNNLEGVFFVLLVGSCCALLYGIISWVLFVMKKAHHYRVPLRDALKEEFQFVIDFNNYVRVLKNSASIYSRSRQSSMSVASVAQESQ

>DmelIR7a

MFHHLWLLMGLRSLAMGALHPPQPEAMTPLVAAALEILAEQVSPSQSTLAVMDLTQDAEHRDERQEQLMTIILRSVGSEMALRTFQKPPAEVPASFVVFLVNSAQAFNTLGFHFTDIHSTREFNFLILLTHRMSSRAERLQVLRDISRTCVRFHTSNVILLTEKRDGVVLVYAYRLLNMDCDLSVNLELIDIYKNGLFRHGHEARSFNRVLSLSGCPLQVSWYPLPPFVSFIGNSSDPEERAQIWRLTGIDGELIKLLASIFDFRILLEEPCNKCLSPDIKDDCSGCFDQVIISNSSILIGAMSGSHQHRSHFSFTSSYHQSSLVFIMHMSSQFGAVAQLAVPFTVIVWLALVVSSLLLVLVLWMRNRLVCGRSDLASHALQVLTTLMGNPLEARSLPRSSRLRILYAGWLLLVLVLRVVYQGKLFDSFRLPYHKPLPTEISELIRSNYTLINQEYLDYYPRELTVLTRNGSKDRFDYIQGLGKEGKFTTTSLIATMEYYNMMHWSTSRLTHIKEHIFLYQMVIYLRRHSLLKFAFDRKIKQLLSAGIIGYFVREFDACQYRKPFEEDYEVTPIPLDSFCGLYYISLIWLSAAVVAFILELLSQRIVWLRRIFE

>DmelIR7b

MKYWLYILSCCSLVASTMESSSDWDLAEALAQVVANSEMGRFKTLYIYTHTNSQSTGGHLEELLDQVLMIVPNNLQARRLLLQQSMEYKPYVHAVLALVDGLPSLSAIYARIRATQDLSHTLIYMSMPTDAYGEEMQATLRFLWRLSVLNVGVVLRPPGDHILMVSYFPFSALHGCQVISANVVNRYQVGTKRWASQDYFPSKLGNFYGCLLTCATWEDMPYLVWRPDGSGSFVGIEGALLQFMAENLNFTVGLYWMNKEEVLATFDESGRIFDEIFGHHADFSLGGFHFKPSAGSEIPYSQSTYYFMSHIMLVTNLQSAYSAYEKLSFPFTPLLWRAIGLVLILACLLLMLLVRWRHHHELPRNPYYELLVLTMGGNLEDRWVPQRFPSRLVLLTWLFATLVLRSGYQSGMYQLLRQDTQRNPPQTISEVLAQHFTIQLAEVNEARILASLPELRPEQLVYLEGSELQSFPALAQQSGSSARVAILTPYEYFGYFRKVHPMSRRLHLVRERIYTQQLAFYVRRHSHLVGVLNKQIQHAHTHGFLEHWTRQYVSAVDEKDESVARIASTSYSTLDGIDGDPSLSESEEDQQVAPVRQNVLSMRELAALFWLILWANLGAVVVFVLELLLPRIKLRKILRKMKSDIKKQISKLVRK

>DmelIR7c

MLHSAVHNVSLVYALVWAIDNYYGMATSTPLAVVQFPTSRESRRLHNDLIDAALGRSSGTGRIQFLLEDDRVEMTETDTDPPPPSGLTGRPIAIWFLDSLRSYFRLEMYLNQLGSPYKRNGFFLVIYTGLEDQPMESLKIMFRRLLNMYVLNVNVFLQRDGTVHLYTYYPYGPHHCQSSLPVYYTAFQDLAAPANGFGLTKPLFPRKLTNMHGCEMVVATFEHRPYVIIEDDPKTPGGRSIHGIEGLIFRSLAERMNFTIKLVEQKDKNRGEILPDGNFTGILKMMVDGEVNLTFVCFMYSKARSDLMLPSTSYTSFPIVLVVPSGGSISPMGRLTRPFRYIIWSCILVSLIFGFVLICLLKITALPGLRNLVLGRRNRLPFMGMWASLLGGLALYNPQRNFARYILVMWLLQTLILRAAYTGQLYLLLQDVEMRSPIKSLSEVLAKDYEFRILPALRTIFKDSMPTTNFHAVLSLEESLYRLRDEDDPGITVALLQPTVNQFDFRSGPNKRHLTVLPDPLMTAPLTFYMRPHSYFKRRIDRLIMAMMSSGIVARYRKMYMDRIKRVSKRRNLEPKPLSIWRLSGIFVCCAGLYLVALIVFILEILTTNHRRLRRAFNVINRYAA

>DmelIR7d

MDIRCVVALLLGLCKVQAVVWPHQHLLEEQLASQISATLQKIFINGLAVYNFGVFISTSYEEMDRDRVILVHQVLNRNLYPPNFPVAVVLASKMNRKITAQVFTQLLFVQNAEQAIAIAEGVNRNGLCVIVLLTSQPERPIMTKIFTYFMQERYNINVVILVPRLHGVQAFNVRPYTPTSCSSLEPVEIDIKDGDLWDVFPRRLKNLHGCPLSVIVWDIPPYMRINWKSSDPMDGLDGLDGLLLRIVARKMNFTLKLIPNEPNGLIGGSSFMNGTFTGAYKMLRERRANITIGCAACTPERSTFLEATSPYSQMSYIIVLQARGGYSIYEVMLFPFEKYTWLLLSTILGLHWIVGSRWRMPSPILAGWMLWIFVIRASYEASVFNFIQNSPVKPSPRTLDQALSGGFRFITDHASYRMTLKIPSFQGKTLISAGQPVDVFDALLKAPWKTGAFTSRAFLADHLVRHRKHRNQLVILAEKIVDNMLCMYFPHGSYFAWEINKLLFNMRSFGIFQHHSQILAWDNLPTTTDTDTPGKRIHSSTESVATGFAESMSFVVAALNCLMGALCISIVVFGLELLSRRRHWTGLEWLFERV

>DmelIR7e

MNISALLNSYYDLSGEQMNHINEFVARAVLHVVHHYILSVTPSLVLTLCCRSNHTCNFYNKMMSTLFREWGLAPLQIVNVLRGVPWHPVPGRRHFNVIFTDSFAAFEEIRMEYYSREYNYNEHYFIFLQARDRLLQGEMRLIFDYCWRYRLIHCSIQVQKSNGDILFYSYYPFGEHGCSDMEPQLINRYNGSMLVEPDLFPRKLRNFFGCPLRCALWDVPPFLTLDEDQEEVLRVNGGYEGRLLLALAEKMNFTIAVRKVHVNMRDEALEMLRRDEVDLTLGGIRQTVARGMVATSSHNYHQTREVFGVLASSYELSSFDILFYPYRLQIWMGILGVVALSALIQLIVGRMLRERMGSRFWLNLELVFVGMPLLECPRSHTARLYCVMLMMYTLIIRTIYQGLLYHLIRTHQLNRWPQTIESLVQKNFTVVLTPIVQEVLDEIPSVQHMRFRLLEANSELDPLYFLEANHQLRQHVTASALDIFIHFNRLSADKVHQRGEQGSGAHFEIVPEDIISMQLTMYLAKHSFLIDQLNEEIMWMRSVGLLSVWSRWELSESYLRNEQSFQVLGTMELYAIFLMVLVGLIVGLLVFILELVSMRSIYLRKLFT

>DmelIR7f

MNTTSDSNAGSSLSSGSGYSIYKSYLENSRIDMQGEDANLYVARALRLVIENVLAQLSTTLVVTISTRHLGTAHWFEYMMNILMDSWRMVAVQLLRIRPDLVVNPVPGRKRVSLLMVDSYQGLLDTNITASNANFDDPDYYFIFLQARDHLIPKELQLILDHCLAHFWLHCNVMIQTAQVEVLVYTYYPYTADACQKAYPIPVNTFDGRKWKASQMFPDKLSQMHGCPLTVLTWHQPPFVELVWDPKHNRSRGSGFEIQLVEHLARRMNFSLELVNIALLRPNAYRLAEGSSEGPIEKLLQRNVNISMGYFRKTARRNQLLTTPMSYYSANLVAVLQLERYRIGSLALLVFPFELSVWMLLLLALLIHLGIHLPSARRGNEEDGGGGLQVVALLLGAALARLPRSWRHRFIAAHWLWASIPLRISYQSLLFHLIRLQLYNTPSFSLDQLLAEGFQGICTANTQRLLLEMPQLARDPDSIQSVDTPFDWDVLNVLTRNRNRKIFAVANQDVTLSFLHSSAHPNAFHVVKQPVNVEYAGMYMPKHSFLYEKMDDDIRRLDASGFIHAWRRASFASVHRKEQVHMTSRRYINHAKLSGIYMVMAGLYLLAGLLFAGEVLLRQRN

>DmelIR7g

MNVTSLLNFESMKYIGAQTQAASINHHVAQALRVFIEDFYQRIAPAFIVVLSCRRPSPMNFYRNIMQLLYESVDTMIVQLVLVELGRPRRIAGPRTHNLLLVDSLDALLDIEIHTYTAQSDTSEYYFIFLQQRDALIPHDMQGVFAYCWRHQLINCNVMTQSSGGQVLLHTYFPYAPGQCNDSQPTRINMFLGESWKHRDYFPSKLHNLNGCPLIVLARKVSPFLDLDEGQRELRGLEGRLLQELSRRMNFSIQFSGLQDQLKNRTTWTEKQLLQKLVQERIAHLAIGYVRKRIQYATNLTPVFPHYSNRVVGCLLLNAHNLTSLEIWSFPFQALTWICLLLFASWLIFGLIVRSMYSALLFFILRYHLHQRLPGNLQDLTHGDYAAVMGRTTLQDLREVPSLQDLLGLKSVIVTSEREEEVLRTLDRCTLREGAGSHPLFFGLISQDALLHLTQRGHRAGAYHIIPQDVLEQQLAIYLQKHSHLASHLDHLVMSIRSVGLVHHWAGQMASERYFRSRFLYREKRIRQPDLWAVYILTAGLYLLSLVVFICELLASRRAGL

>DmelIR8a

MELPLLVLLLALRFAGSEVLKITFWIEPVQRAEFDTDIAMVLKELDALRLDVKVDDTTLTLTRSEDGLDMQRFCEILSTVGASAVIDLTYSHWEEGYNLVRSLGIGYVRLERIMRPFLDMFGDFMRQKRANNVAMVFMNARDAVEAMQQMLVGYPFRTLIMDASQTDPGQHFLERIRSLRPAPTYIALFARAAAMNGIFEKVQKAGLFQRPLEWHFVFLDTRDRVFKYRRQAELCTRFTLNPRAICRSMPMPDLYCGSGFTMQRAMLLNVLRSLINAAQVSPGYPMAIYQDCNATASSSEVSDPLEKDDYNWLDMVHWSNFLAYAPPLPHIQDQFQSPVPGLTFAVNISAGYYSSEHEAKTDLAAWSSVGEMRLLNETISPARRFFRIGTAESIPWSYLRREEGTGELIRDRSGLPIWEGYCIDFIIRLSQKLNFEFEIVAPEVGHMGELNELGEWDGVVGDLVRGETDFAIAALKMYSEREEVIDFLPPYYEQTGISIAIRKPVRRTSLFKFMTVLRLEVWLSIVAALVGTAIMIWFMDKYSPYSSRNNRQAYPYACREFTLRESFWFALTSFTPQGGGEAPKAISGRMLVAAYWLFVVLMLATFTANLAAFLTVERMQTPVQSLEQLARQSRINYTVVKDSDTHQYFVNMKFAEDTLYRMWKELALNASKDFKKFRIWDYPIKEQYGHILLAINSSQPVADAKEGFANVDAHENADYAFIHDSAEIKYEITRNCNLTEVGEVFAEQPYAVAVQQGSHLGDELSYAILELQKDRFFEELKAKYWNQSNLPNCPLSEDQEGITLESLGGVFIATLFGLVLAMMILGMEVLYYKKKQNALEITQVRPVNDSSGSGGNSSTAPPTATSTTKQAWHIPVLEAEEKPAKVSPPPSFETATFRGKKLPARITLGDGKFKPRHGLYARRNLGASDSHSGYME

>DmelIR10a

MAVLGTVFLLFMLDLKTLNLTRLNGLLVEPTRDLPQLELWLRAGSDHQDAENPYVQWFLLRTEIPLSIVTYQENRYWMDDPFGRRNLVLVMSLDQLLTNRGAAAPIQKASTFFYILADQDKDLSADEQLRLEGSCRQLWTQHKVYNRFFLTRDGVWIYDPFKRRDSAFGRLVRYYGSETLDKLLFRDMAGYPLRIQMFRSVYTRPEFDKETGLLTRVTGVDFLVAQMLRERLNFTMLLQQPEKKYFGERSANGSYNGAIGSIIKDGLDICLTGFFVKDYLVQQYMDFTVAVYDDELCIYVPKASRIPQSILPIFAVGYDIWLGFVLTAFACALIWLTLRVINLKLRIVSLGNQHIVGQALGIMVDTWVVWVRLNLSHLPASYAERMFIGTLCLVSVIFGAIFESSLATVYIHPLYYKDINTMQELDESGLKVVYKYSSMADDLFFSETSPLFASLNKKLSWNRDLRADVIDEVARFRNKAGVSRYTSLILESSHFTLLRKIWVVPECPKYYTISYVMPRDSPWEDAVNALLLRFLNAGLIVKWIQDEKSWVDIKMRSNILEADAESELVRVLTIGDLQLAFYVVIGGNLLAFLGFLAEHFRWKLQKKGV

>DmelIR11a

MRFAILWLFSGCLLPGIQVGIWVVVRAQPTGRDVLLSRLGNQQNELNTRRLANASSYLTRNYIANRINTLVVREICVECPYELSERQRQLVDQILASLAPELSVLLHKGTAEETTWEYTLFVVNDHTAFTGQVFIFPDELLEREFFCIVVVSEIQSRQFVRQTVGSIVKSNLQMHFVNVVVVAQLEDGTVGTYSYKLFKANCTPGITVRQINHFDRITGKPQQSMPDLYPVRNGHLGDCPFNVGAAHMPPHLIYKRHKDPPPASNVSIPAEDLAGIDWDLLQLLAKALKFRIQLYMPQEPSQIFGEGNVSGCFRQLADGTVSIAIGGLSGSDKRRSLFSKSTVYHQSNFVMVVRRDRYLGRLGPLILPFRGKLWGVIIVILLLAVLSTCWLRSRLGLSHPIEDLLTVIVGNPIPDHRLPGKGFLRYLLASWMLLTLVLRCAYQARLFDVLRLSRHRPLPKDLSGLIKDNYTMVANGYHDFYPLELTCRQPLDFSARFERVQRAAPDERLTTIALISNLAYWNHKHPNISRLTFVRQPIYMYHLVIYFPRRFFLRPAIDRKIKQLLSAGVMAHIERRYMQYENKRKVASNDPVLLRRITKSIMNGAYRIHGLVIVLATGMFILELLAGRSNGRLRRWMEWVHQ

>DmelIR20a

MLASLNRSTGLSAELLDLYGLVVHFLLSGEHTTLVYFNPAGLDCSWGVLWQRNLTAHPQIVWQRNYSYPDLYYQFNAKLLVLACLPMDSRAAIQLEILANSLSHLRTVVRLLIEVAGPDQVTLARQYLSFCLRRSMLHVELYFRDYHHSLILYSFRAFPSFELVMRWISVGQGVKLFLHKLDDLRGHRLRVIPDLSPPNTFFYRDARGDNQVTGYLWDFLATFAGRLNAGLEVVRPSWRAGSASDSSYMLEYSAKGLIDVGLTTTLITKWNLWAIHQYTYPLLVSSWCTMLPVEKPLATPDLFGRIVCPTLAMTLLLIILVTWLVFRQLRCLTRLKNSRPARIVPHLLTLLLLTTCSAQLLSLLIFPPYHVRIASFEDLLRGDQKILGMRNEFYNFDGAFRARYAGVFYLIDDPNELYDLRNHFNTTWAYTMPYIKWLVIKTQQRHFSKPLFRWSKDLCFFDFMPTSVIVAPDSIYWESIKDFTFRIHQAGLMKHWIRKSFYDMIKAGKMSIKDYSDLETLKPLNIGDLEIVWRVCGAAIAVASAIFIMELLYFYINVFFNSL

>DmelIR21a

MSYYWVALVLFTAQAFSIEGDRSASYQEKCISRRLINHYQLNKEIFGVGMCDGNNENEFRQKRRIVPTFQGNPRPRGELLASKFHVNSYNFEQTNSLVGLVNKIAQEYLNKCPPVIYYDSFVEKSDGLILENLFKTIPITFYHGEINADYEAKNKRFTSHIDCNCKSYILFLSDPLMTRKILGPQTESRVVLVSRSTQWRLRDFLSSELSSNIVNLLVIGESLMADPMRERPYVLYTHKLYADGLGSNTPVVLTSWIKGALSRPHINLFPSKFQFGFAGHRFQISAANQPPFIFRIRTLDSSGMGQLRWDGVEFRLLTMISKRLNFSIDITETPTRSNTRGVVDTIQEQIIERTVDIGMSGIYITQERLMDSAMSVGHSPDCAAFITLASKALPKYRAIMGPFQWPVWVALICVYLGGIFPIVFTDRLTLSHLMGNWGEVENMFWYVFGMFTNAFSFTGKYSWSNTRKNSTRLLIGAYWLFTIIITSCYTGSIIAFVTLPAFPDTVDSVLDLLGLFFRVGTLNNGGWETWFQNSTHIPTSRLYKKMEFVGSVDEGIGNVTQSFFWNYAFLGSKAQLEYLVQSNFSDENISRRSALHLSEECFALFQIGFLFPRESVYKIKIDSMILLAQQSGLIAKINNEVSWVMQRSSSGRLLQASSSNSLREIIQEERQLTTADTEGMFLLMALGYFLGATALVSEIVGGITNKCRQIIKRSRKSAASSWSSASSGSMLRTNAEQLSHDKRKANRREAAEVAQKMSFGMRELNLTRATLREIYGSYGAPETDHGQLDIVHTEFPNSSAKLNNIEDEESREALESLQRLDEFMDQMDNDGNPSSHTFRIDN

>DmelIR25a

MILMNPKTSKILWLLGFLSLLSSFSLEIAAQTTQNINVLFINEVDNEPAAKAVEVVLTYLKKNIRYGLSVQLDSIEANKSDAKVLLEAICNKYATSIEKKQTPHLILDTTKSGIASETVKSFTQALGLPTISASYGQQGDLRQWRDLDEAKQKYLLQVMPPADIIPEAIRSIVIHMNITNAAILYDDSFVMDHKYKSLLQNIQTRHVITAIAKDGKREREEQIEKLRNLDINNFFILGTLQSIRMVLESVKPAYFERNFAWHAITQNEGEISSQRDNATIMFMKPMAYTQYRDRLGLLRTTYNLNEEPQLSSAFYFDLALRSFLTIKEMLQSGAWPKDMEYLNCDDFQGGNTPQRNLDLRDYFTKITEPTSYGTFDLVTQSTQPFNGHSFMKFEMDINVLQIRGGSSVNSKSIGKWISGLNSELIVKDEEQMKNLTADTVYRIFTVVQAPFIMRDETAPKGYKGYCIDLINEIAAIVHFDYTIQEVEDGKFGNMDENGQWNGIVKKLMDKQADIGLGSMSVMAEREIVIDFTVPYYDLVGITIMMQRPSSPSSLFKFLTVLETNVWLCILAAYFFTSFLMWIFDRWSPYSYQNNREKYKDDEEKREFNLKECLWFCMTSLTPQGGGEAPKNLSGRLVAATWWLFGFIIIASYTANLAAFLTVSRLDTPVESLDDLAKQYKILYAPLNGSSAMTYFERMSNIEQMFYEIWKDLSLNDSLTAVERSKLAVWDYPVSDKYTKMWQAMQEAKLPATLDEAVARVRNSTAATGFAFLGDATDIRYLQLTNCDLQVVGEEFSRKPYAIAVQQGSHLKDQFNNAILTLLNKRQLEKLKEKWWKNDEALAKCDKPEDQSDGISIQNIGGVFIVIFVGIGMACITLVFEYWWYRYRKNPRIIDVAEANAERSNAADHPGKLVDGVILGHSGEKFEKSKAALRPRFNQYPATFKPRF

>DmelIR31a

MNLLISMFILILAAGEGEIIPSMEESVVTNFVKSLVKTKQAIVFSCLFKDFKEISLALMRINQFVSVVNLNQSYSLTSILTRENYARTSVMVNARCSGSSELLFEASENRYFNKTYQWFLWGVDLEVQSLFPLNLNYVGPNAQITYVNETADGYAYWDIHSKGRHLKSNLEINLIATLINDTLNIARDIFHLQSIDFRGQFNGLTLRGASVIDKEDIISNEQIESILSRPTKDAGVAAFIKYHYELLGLLRERFNFTVNFRNSRGWAGRLGNTTFRLGLLGIVMRNEADIAASGAFNRINRFAEFDTIHQSWKFETAFLYRYTSDLDTHGKSGNFLSPFSDRVWLFCLLTLGAFSIIWVLFEIIDYKILRIRVNSQKLEHLNQKSSVICIKTTCIERILQTFGACCQQGLDPNPVDRSVRFLVMTLFLFSLVMYNYYTSSVVGGLLSSSDQGPSTVDEITASPLKISFEDIGYYKVLFRESQNRSITRLIEKKLSSSRSLNELPIFSHIEDAVPYLKAGGFAFHCEVVDAYPVISEYFDANEICDLREVSGLMEVEILNWILHKNSQYTEIFKTAMCNAQEKGFVERILRRRQIKKPACQSLYTVYPVSLSGVLPGFVILICGFGASLLLLCLEKVYAHFGPRKFCGF

>DmelIR40a

MHKFLALGLLPYLLGLLNSTRLTFIGNDESDTAIALTQIVRGLQQSSLAILALPSLALSDGVCQKERNVYLDDFLQRLHRSNYKSVVFSQTELFFQHIEENLQGANECISLILDEPNQLLNSLHDRHLGHRLSLFIFYWGARWPPSSRVIRFREPLRVVVVTRPRKKAFRIYYNQARPCSDSQLQLVNWYDGDNLGLQRIPLLPTALSVYANFKGRTFRVPVFHSPPWFWVTYCNNSFEEDEEFNSLDSIEKRKVRVTGGRDHRLLMLLSKHMNFRFKYIEAPGRTQGSMRSEDGKDSNDSFTGGIGLLQSGQADFFLGDVGLSWERRKAIEFSFFTLADSGAFATHAPRRLNEALAIMRPFKQDIWPHLILTIIFSGPIFYGIIALPYIWRRRWANSDVEHLGELYIHMTYLKEITPRLLKLKPRTVLSAHQMPHQLFQKCIWFTLRLFLKQSCNELHNGYRAKFLTIVYWIAATYVLADVYSAQLTSQFARPAREPPINTLQRLQAAMIHDGYRLYVEKESSSLEMLENGTELFRQLYALMRQQVINDPQGFFIDSVEAGIKLIAEGGEDKAVLGGRETLFFNVQQYGSNNFQLSQKLYTRYSAVAVQIGCPFLGSLNNVLMQLFESGILDKMTAAEYAKQYQEVEATRIYKGSVQAKNSEAYSRTESYDSTVISPLNLRMLQGAFIALGVGSLAAGVILLLEIVFIKLDQARLWMLCSRLQWIRYDRKV

>DmelIR41a

MFIDLSWSLVLSAIVGKYLNESTICIFWNDKFEFQLLHKSDYISFVGINIKSFDDNGGHYIIDTGLKKKELQNKHLFLDELVIKIIISIEVTHCETFVVFDKDIDRFVNAFNKASVYSIWRSLHNKFVFAHIANESPESRNHFFEDQPNILFVVRDHSSASSFDIKTNKFVGRKAENPSQMILVDRYLASEQRFQFGKSLFADKLNNLQGREVIIAGFDYPPYTVIKHNMSTNAQDMGVSGESDFKNVYIDGTETRIVLNFCEQFNCTIQIDSSAANDWGKVYPNMSGDGALGMLINRKADICIGAMYSWYEDYTYLDLSMYLVRSGITCLVPAPLRLTSWYLPLEPFKETLWAAILLCLCAEATGLVLAYKSEQALYVLPGYREGWWTCTSFGVCTTFKLFISQSGNSKAYSLTVRVLLFACFLNDLIITSIYGGGLASILTIPSMDEAADTVTRLRFHRLQWAANSEAWVSAIRASDEALVKDILYNFHIYSDDELLRLAQDQHMRIGFTVERLPFGHFAIGNYLGPQAIDQLVIMKDDIYFQYTVAFVPRLWPLLDKLNTLIYSWHSSGFDKYWEYRVVADNLNLKIQQQVQETMTGTKDIGPVPLGMSNFAGFIIVWILGSAIATLTFLLELSLTYILKQSNLK

>DmelIR47a

MRQIKLLVWLLVVGVVSSTEQLQFLKNFLEAVHKERSISTILLIQRKVHKNDFLHGLYPIFWPIICLDETKRVELVNNFNKDFLALVYMESEADTLLLSALAADLNHIRDARIMIWLQMSPSENFLDRIVFQASKQKFLNLVVIENTLKTRRFYPFPQPKVQVIDKPFEEKEIYPALWRNFMGKNAIAVPDLVPPRSFNSFDPKTGHRRESGSIYNVFKAFTQRYNITMLLKWPLIRNTTQEEIIGKSVRGEIDLPITGQLISFRHPNGSRSQPLLGMTALSIAVPCGPELPMFDRFFLFYGLATPITITGYYVLLNTIEIILGTLSDRIKRHPRRKKILNLVLNLRVFSCILSLPTPQGNRLRSVKGQLTMVMSITGLILSCIVAAQTSTILTMKPQYRHIKNFQELSDSNITVVCNHLNYLTIKQQMDPKFMAKFMQNIWIVNSIEQMKMIFDLNTSYAYQTFSYKKDPFTLLQMHTTRKAFCRTPGLDLVSGLAYTAVLEKNSIYALALQDYTLKAFSAGLVYYWAEESIRDLISTVGRTQFEKLPIVIGYQSLKLQDYNVCWKILLIGGALAFCVFIVEVVVGLINRRI

>DmelIR48a

MIFLPTFLALMHLLITETNLIIGKTLPDILNELNERLIISTNIIFCKQFDNLIPFEAQTSRFVYSSLEAFNITSLWNHVGNDNKLFVIVGNEPPYELFAKLDLRFQLVDFLLVIGNEIEEDIXKSFIKYAWRLGYVKILIYSTYTEICYHHVLFPSLRIRETNIADYTKFRGTFRNLNGHQVRVAAYNNVPRSFIYSDKNGKRIFAGYYMRFARAFIQNINATFKPVYTPNDSPENCTQFILNNTVDICADALVKNSNAFSVSRELRIAPANVIVPHGKPLLSYRYLSAPFETKVWIALGTYVFLISGFLCLIYWLRSGKWDYSQNLLEVYSSLLFXFTGFHLKAINGIERYILFGVLFISGFVYSTMYLGLLKIMLISETFEKQIQTFEELAESNIPLLIDPXWTAVRTVSSETLLNHRNNFDQNYAYVMFPDRMDMFVYAQQYLRHPKFRRIPIDICFLFAGFPMSKKWFLKHQLSRAWFHAFESGIVNKMAWDAYRESVGQGYLRFPITEHLEAKLLGLYYFMMPTISLILGYSVVLLSFIMEVIACKLNCTLNYRCG

>DmelIR48b

MILQQSSNLLKLLLLLAISSVRTQGLNDIIIELNQRLLISNNFLYCNQSDKLNEYEIKYLQHMPPISLMIFTSIESMNFTQVEYNLGADNKLFLIMGNEEPPYDFLHALNLHFQFAEYIIVIDEPVDLKKSTKWLDFVNHLWQQGYVQLLIYTSYDEKLYHKIIFPETVIEETLVEQYISIRGSFNNLYGYPVRVAAYNNAPRSMLYVNRWGKHIFAGFYMRFLRAFIDARNGSFVPVLTPSNSPGNCTLNLVNETVDVCADALAANPAAFSLTHGFRIASANVLVTHAKPLHSYRYLTAPFQWSVWACLVIYVLLVVNFLSFIGWLRSGKWEFSKYLLEVFSSLLFSGFYLKEIRGRERYILFGVLFIAGFVYSTEYLGLLKSMLISEVFEKQIDTFEALVESNITLMVDPYDKILFAKYNMPEILSPIMELVSFETLLKHRNRFDQDYAYILFSDRMALYDYAQQFLKHPKLLRIPIDFSFLYTGIPMRKRWFLKHHLGRAWYWAFESGLTRKLALDADFEAVRVGYLSFLITEHVEAQPLNVDYFVMPAIALAIGYILALLSFVIEMTAWRIREFLGCRKATMTSTGCSEGGHVDVD

>DmelIR48c

MSLLRIILIIIFLRIVSSIPDTIISHLSAELQIKIQIYFGLGNDLYDFSRLDGNYQKIIISHNISEEFKTYHDEPVLIIIRLERDLNLNLATLDVLRSYLTDRQYNDILLIDNDEENLNSYVDIRKAYWNAGFSQVLIYNSQQRTWSIKPYPYLQIRPTSLKEYIENRNTRNLMGYPLRVLVTNDPPHCFVDKDELPGSPNRYKGSIVTMLKIFADQLNATFQANPFREFRRYSTADCVQMVSDDEIDACGSIFIRTYTYATSQPVRLNRVVIMAPFGNPIEKFYYFFRPFDLYVWIGTGIIVVYIAVMGSLLHRWHFKEWNVGQYLLLAVQTLLNRELSLPQSSSGSKFMLLLLLFAIGFILSNLYVALLSMMLTTKLYQRPIENLADLKAANVNILLQTHNIRPNSVYGSSEELRERFLLVEESQHLEKRNGLDPSYAYVDSEDRMDFYLYQQKFLRRRRMKKLSNPVGYTWAVQVIKQNWVLEKHYNDHVQRFFETGLQNKLVDDVHELAVKAGFLHFFPTQTQTIEPLRLEDIVMAAMVLGGGHALAVICFLVELFA

>DmelIR51a

MYNVLVLLLLLSTHAQMEPYRRGHNITLLRSVLTVIRGRENWKNTPIFIGGHSNSDDLNNLMSWLQNTMEVTCHTVDTSTPAKNEHALGNFNINADNSLGLLFCQSSHEVIWFNMDKRLRRLRGIRLIVILLNKRSSSNKAIMGTFKRLWHFQFLKVLVLHRDQIYSYTPYPVIRFFKLDTNVYPLFPPSVQNFQGYVVSTPVGNDIPRVFFVKDKKTGRKQIRGFGYRTFVEYLHRYNASLHVSNSHQEHAINSSVNMGRIINQIADGQLEISLHPYVDVPENMGDNSYPLLIASNCLIVPVRNEISRYMYLLLPLNQSSWILLLGSVIYISGVLYYIQPGLVHRTWAQRIGLNILDSISRIINICSPSRIYDPSLRYFIVSVHLSILGFVVTNLYSIMLGSFFTTLVVGEQVDSMEQLIQQQQKVLVKYYEVSTFLRHVEPDLVDGVAQLLVGVNASEQVSALLGFNRSYAYPFTLERWEFFSLQQQYAFKPIFRFSSACLGSPIIGYPMRRDCHLQSSLNMFIMRIQAAGLLQHWVVSDFNDAMRAGYVRLLENFLGFHSLDVDSLRLGWAVLLCGWLLSALIFLCER

>DmelIR51b

MCKVLTLLVVILLLALTNAAYNVTLLKSVLSLISTREPWINTPIFVGHNTQGGDLNDLIIWLHQTMGVTSLTMNLFLQPEHIRPLGHFKITRYNGIALFFCHDKHDIMWLTLDRNLRKLRRIRLIIILRNQRSGSQGAIKSIFNALWQYQFLNVLVLQRDQLYSYTPYPAMRFFKLDIHTEPLFPHAARNFHGYVVSTPAENDIPRVFHVHDPLTKSRKVLGYAYRTFVEYLDHYNASLRLTNPDENLDPTTSVNMNHIVQLIIDGQLEISLHPYVFTPPTATKSYPLLIYPNCLIVPMRNEIPRHMYLLRPFQLYSWYILLFAVFYITGILYCISPKLNKSSWPQRLGLNFLDAISKILFISPPITIYRPTWRHLIIFLQLSVLGFMSTSWYNIELDSFFTTIVVGEQVNSMDQLVHQQQRVLVKEYEINTFLRHVEPRLVEKVSRLLVPVNASEQVSALLSFNRSFAYPFTEERWQFFAMQQQYAFKPIFRFSSACLGSPHIGYPMRVDSHLETSLNHFILKIQDTGLLNHWVVSDFNDAMRAGYVRFVDNVLGYQSIDVDTLRLGWCVLGIGWILSALVFSCEYWHLYPWRFIA

>DmelIR52a

MALGWSVIILGCIGQLSAQILNYTQSRDLELLEGRLLRVLSRLNLEEEYNTLLIYGKECVFHSLLRKLEIPAVTVPSGSTDYDWSFSTAILILSCGYDAENEENSYTLLKLQRTRRLIYLEDNSEPESVCMRYSLKEQHNIAMVKSDFDQSDTFYSCRLFQTPNYVEGHFFKDQPIYIENFQNMRAATIRTVADSLVPRTILYRDEKSGETKMMGYLGHMINTYAQKLNAKLHFIDTSKLGAKKPSVLDIMNWVNEDIVDIGTALASSLQFKNMDSVWYPYLLTGYCLMVPIPAKMPYNLVYSMIVDPLVLSIIFVMLCLFSVLIIYTQHLSWKNLTLANILLNDKSLRGLLGQSFPFPPNPSKHLKLIIFVLCFASVMITTMYEAYLQSYFTQPPSEPYIRSFRDIGNTSLKMAISRLEVNVLTSLNNSHFREISEDHLLIFDDISEYLVLRDSFNTSFIFPVSVDRWNGYEEQQKIFAEPAFYLATNLCFNQFMLFSPPLRRYLPHRHLFEDHMMRQHEFGLVTFWKSQSFIEMVRLGLASMEDLSRKRNEEVSLLLDDISWILKLYLGAMFISSFCFILEILRCGERCKRLWRCRW

>DmelIR52b

MTWLVILLCFLGYMAAHIADISVQNQRLMDNELINLLLKLRNEEFYDTLLVYGKDCEFHSVIKNVDVAVVLVSDSMNFEWNFSSLTLILSCGPDIDNGGPNSTSIKLQRNRRLVLLKEDFQPSNICNIYTQKEQYNIALVRENFTKSKSIYTCRYFQDPNVDEVNLSGTKPIFIEQFQNMKGKAIKIVPDLLPPRVMVYQDANDGELKMIGYVANLITNFAQKVNATLQLDFLKPSKSITEISRMAKDDELDMGITLEASLNTSNLETSSYPYLLTSYCLMVQVPAKFPYNLVYALIVDPLVLGIIFVLFLLLSVLLIYSQKMSCRDLSVANILLNDKSLRGLLGQSFPFPLNASKKLRLIFTILCFASIMLTTMYEAYLQSFFTDPPSEPHIRSFKDIGKLRHKTIITAIEANVLISTNNTQFLDIPKKHLCIYENWRDWLAMRDTFNLSYNYLVTEDRWSSYAEQQKLFKTPLFYYSNDLCFSRMIFLSIPLRRHLPYRHLFDEHMMRQQEFGLVNYWKSHSFFDMVRLRLTPLKDLSQPTVYNPSLLMEDISWIMKIYFVAISLSVFCFTMEIGWDKWKRWRD

>DmelIR52c

MVWLIIILFCLGNSSSQILDVTNNSHLDFDYRLFGLLQRLQVEKFYDTLLVYGEDCAIPSLFERLQVPAVLVSSGSTNFDWNFSSLTLILSCNFQDEREENYRTLMKLQTSRRLILLKGHIKPESVCDFYSKKEQHNVAMVKENFYQLEVVYSCRLFQDQNYEKLNLFDGKSIYKDQFRNMHGAPIRTLSDKEPPRTIPYIDSKTGEEKFKGYVGMLISQFVKKVNATMQIREDLIKDDEEVSFVDITNFTSNDILDIGICEARTLEMSNYDAISYPYLMSSYCFMAPLPDSLPFSDVYMAIVAPSILIMFLIIFCICSVLIIYIQERSYRSLTIRSVLMNDICLRGFLAQPFPFPRQYNRKLKLIFMLVCFSSLISTTMYTAYLQAFLWGPPIEPRLTSFDDVKKSRYTMAINIYEREFLEALNVSLEDVEIYDYGKFSKLRSTFNTNYLFPVTALQWFTINEEQKLFKYKIFYYCDAFCLNQFDILSIPLRRHLPYRDIFEEHMLLQKEFGLTKYWIDQSYRDMIRANLTTFKDFSPPLENDYIEVHNLYWVFTMYFVGMGMGLCFFILEILRPLRYWRNCKIKCEYCYAFLKNFAK

>DmelIR52d

MVRIIIILLCLGYTKARILDATNTNHTDLEERLLSLLLRLQQEQFFNTLLIYGEDCAFSSLSRRLQVPTILVSSGSTSFEWNYSSLALILTCEFKAEREENYQTLKKLQMNRRLILLNGNIKPDSVCDFYSKKDQYNIAMVNNNFHQVGIIYACRLFQERNYEKVYLSEGNPIYVDQFRNMQGALLKSITFNLIPGSMAYRDPKTGQEKHIGYVANLLNNFVEKVNATLDMQVKLHKAGKKTSFYNITKWASEDLVDIGMSYAAYFEMTNFDTISYPYLMTSTCFMVPLPDMMPYSEIYMGIVDPPVLVVLIAIFCIFSVMLNYIKQRSWRSLSLVNVLLNDICLRGFLAQPFPFPRQSNRKLKLISMLVCFFSVITTTMYTSYLQSFMWGPPIDPKMCSFADLENSRYKLAIRRYDIEMLRPFNVSMDHVVVFDESSQLEYLRDSFDDNYMYPMSALSWSAFKEQQKLFAFPLFYYSEKLCLKPISFFSFPIRRHLPYRDLFEEHMLQQNEFGLSTYWIDRSFSDMVRLKLATMNDFSPPRLEDYIEVSDLSWVFGMYFTGLGISCCCFGLELLGLPSWTRRLRLTNWIRVRN

>DmelIR52e

MAWLIILLLCAGNSNARYLDYIHKNSPHLEDTVFSLLVRLQLEEFFDTLLLYGEDCVFHSSSRRLNVSTVLVSSGSSNFDWNFSSLTLILSCSHEEENETNYHTLAKLQRNRRLVYLRGDIQPQSVCERYLQKEQYNVATIAEDFDKSKIVYACRHFKDPNIEEISLLDSTPVFIEQFRNMYGKPIRAVADLLSPRSMLYMDPKTGDMKITGYVANLVNTFAERVNATLELDVLAKKFIVKQIFNMIDNEQLDIGISLESSFRMKFIEISSYPYILTSYCLMVQVPAKLPYNLVYAMIIDPIVLGIIFVVFLLLSILLIYSQKMSWQDLSLANILLNDKSLRGLLGQSFPFPLNASKKLRLIITILCFASIMLTTMYEAYLQSFFTNPPSEPEICSFQDVGSYNRRIAMSALEVNGLIKTNNSHFREIRMDDLEIFDNMPECYELRDAFNLSYNYVVTGDRWRSYAEQQTLFKEPVFYFARDLCFSRLIFLSVPLRRHLPYRHLFDEHMMQQHEFGFVNYWMSHSFFDMVRLGLTSLKDLSRPLAYTPSLLMDDISWIMKIYLAAIVLCVFCFLLEIGVDKWKRWMKFRNLQILNTC

>DmelIR54a

MWTVITGIVLWAPVLVAGSAVDFIFRAAAEHSLSVIMIRIDYCPYNWAKDIFENQTIPVVVLSDSETFINIRMFSRPLHVACLPGHELQKDLALLENFTSSLMDFPSQKKIVYISNNFSDPTRMDYIFETCYHRRIWNIVGLLASDEHRYFYRYHLYPSFRTEYRSLESSTIFDKDFPNMHGHPLTVMPDQWLPRSVLYVDRRTGKQILAGSVGRFFHVLSWKLNATLQLSKKVTTGRFLNATALKELSESFSVDVPASLTIMERVEQLASTSYPMEVTHVCLMVPVARRIPIKDIYFILSSASNMFLAIVIVSSYGLALNLLRNMTHRDVRLVDFVLNDKALRGILGQSFNLPLSRSFSTRLIFLMLGIVGLNVSSIFGAGLDTLMAHPPRQFQARSFAGLRRTKIPLVTTEEDFPTWMKLRVPMLVVNVSEYNHLRNGRNTSNAYFASRLYWNLFSEQQKRFTRELFIYSTDDCLWSLALLSFQWPQNSLFTEPVSQLILEVNANGLYDFWVGMHYYDMTAAGLSGLEDPSLQLKEREHPTSLRIVDFQWMWQAYGTFMVIAILVFLLEVSWHRITSLFVSLVY

>DmelIR56a

MGSRFFIRNLILFGLLASSNMQIPFGELEKKFELDVDFLLGVTELVGHIQGLYSITVYADCIDIHPSIQQRIMDKFMVPVNTIGSNLSRPNYHKLDNSRIRIVLFTGLNDTILVNLNKTDVPYSDNFYMLAYASAIKNKCIELDFIEEVFTLLWKMSIQNAILLIRGEFMMEMWSYLYMGKIHKIKLTKPNSYLESLRKYNYRFSLEVINDPPAIFWYNSSEQADVTGGGNLSVSGPLGLIIINFLRHLNVTIDIVPIPGKQTSQYELFQQPDNLRAENGVNMVGSALLKYSPMVTQSRMCLLVSNRRMIPFSRFLDRLVSPGVHKLTFVSSIGIFVIKYFSHRPRSFVDAIFCTIRFFFAIPLPSIILNRLPVVDRFIEVFIIIFVQILLSSNISITTSALTTGFWEPPIINVETMRASGLHILTEDPTILQAFKENILPSSLADLVILVDEDTYFHHVTTLNNSYVYVVQAHNWQIFRLYQQQMTNEPFEIASEELCSKWRILGIPLNPKSPLRFMFKDYFYRILESGLREQWVHSGFKKFCEFNNLKKLPVDSVDSWQPLSIEFYSNVIRAYIIGLVIATLAFVAELLHNGYRRKNVKKT

>DmelIR56b

MLLDTDLASGVIRSPYSFDIPHAFIFNETQFVVPKFCGPYMEIVKHFAEVYHYQLFLDSLESLPKKSVVEQDIISGKYNLSLHGVIIRPEETSDFFNATQHSYPLELMTNCVMVPLAPELPKWMYMVWPLGKYIWTCLFLGTFYVALLLRYVHWREPGNATRSYTRNVLHAMALLMFSANMNMSVKLKHASIRVIIFYTLLYIFGFILTNYHLSHMTAFDMKPVFLRPIDTWSDLIHSRLRIVIHDSLLEELRWLPVYQALLASPSRSYAYVVTQDAWLFFNRQQKVLIQPYFHLSKVCFGGLFNALPMASNASFADSLNKFILNVWQAGLWNYWEELAFRYAEQAGYAKVFLDTYPVEPLNLEFFTTAWIVLSAGIPISSLAFCLELFIHRRKQRRPQYERFECYDY

>DmelIR56c

MQHLLNLLAPFGRMNVFQEIVWFVSPHQRLDQLDEFIMRIDEAFGKSATQTVVNNNTEMRMIYSSARRNHMSFVFTTGAEDPIMKVFSKVLLGRHFYVSMVIYVDKVGDMHPIYDLLTFAYNQQFFNSMVHFESMEGVNQLFGVSKFPVMSFENRTDFLKYMGKIWKQVQNARSDVGGFGFTTPLRQDLPHLFQSQGHYDGSTYRIIETFVRFINGSFKELIMPPDSLGGQVINMKDALQLIRERKMEFCAHAYALFMSDEELEKSYPLLVVQWCLMVPLYNSVSTYFYPLQPFDWNVWFFALGALLALVLLELMWLRMFGGWSGYRGAVLNSFCYIINVPIEGQLQQPCLLRFLLLATVFFHGFFLSAYYTSNLGSILTVNLFHAQINTMNDIVSAQLPVMIIDYEMEFLLNLNKELPQEFLELLRPVDSAVFSEHQTSFNSSFAYFVTEDHWEFLDEQQKHLKQRLFKLSSICFGSYHLAFPLQMDSSLWRDIEYFTFRIHSSGLLNFYARSSFGSALHAGLVQRMPDTQEYTSAGLQHLAIAFILLLVMSFLAGIVFVLETLSR

>DmelIR56d

MDNRAAELILRERNIFPTNGSDNITLLNNMFVLEMFYRITQLYHFKNFIFYISERLDLNNKDSQEFFHNFWTYFPMAPNLIITREHHLGIPMMQFISTPSLVMVFTTGKDDPIMELASHNQQGIHWLKTIFVLFPSLQSRDFETNPESLAQFTAEIKDVYDWVWRKQFINTFLITIKDNVFILDPYPTPSIVNKTGVWQAEEFFHKYAKNMKGYLVRTPILYDMPRVFKSDRPTNRYEKNFIHGTSGNLFLGFLEFVNATLMDTSANVTADYLNMTNLLDLVSQGVYETLIHSFTEITTKFVVSYSYPIGINDCCIMVPYRNQSPADQYMHEALQENVWVLISLFTLYITVAIYLCSPLRPRDLSAAFLQSICTLTYSVPTFIIRTPTLRMRYLYILLAIWGIVTSNLYISRMTSYFTTAPPVRQINTVQDVVEANLRIKMLAIEYERMAKSPLQYPESYLNQVDLVDKHMLDLHRDPFNTSFGYTVSSDRWRFLNLQQLHLRKPIFRLTEICEGPFYHVFPLHKDSHMRSVMTEYIMIAQQAGLMNHWERETFWEAVHLHRIHVHLFDDEPMALSLDFFSSLLRTWTLGLILAGLAFAAEMKWHEHVTFKRRPVIRITRKPRSFLRRFMKL

>DmelIR60a

MWCNNPGLIIIIFLGQILNLCQGIVNLSNETANTVIFMLPEKDLGPDVWKAGVGCLDSFAQIFFFRNPKERFTRAYNLMLVHAFHLSSPADQIQEGFSKLINEAVTNPGPPDREELFQMRVASDYNITNGTEDKGELILADNYVIVVDSVDRLKELMKKKIVEMRSWNPGARFLVLFHNATCRNRPLGVASNIFKDLMEMFYVHRVALLYANSTMNYNLLVNDYYSNVNCRILNVQSVGQCHDGKLYPNNAVVKASMQDYVSGFSPRNCTFFACSSISAPFVEADCILGLEMRILGFMKNRLKFDVNQTCSLESRGEMDGPANWTGLLGKVQNNECDFVFGGYYPDNEVADHFWGSDTYLQDAHTWYIKMADRRPAWQALVGIFEAYTWIGFILILIISWLFWFTLVMILPEPKYYQQLSLTAINALAVTISIAVQERPICETTRLFFMALTLYGLNVVATYTSKMIATFQDPGYLHQLDELTEVVAAGIPFGGHEESRDWFENDDDMWIFNGYNISPEFIPQSKNLEAVKWGQRCILSNRMYTMQSPLADVIYAFPNNVFSSPVQMIMKAGFPFLFEMNSIIRLMRDVGIFQKIDADFRYNNTYLNRINKMRPQFPETAIVLTTEHLKGPFFILVVGSCWAALTFIGELIIHRWRTQLVSTSEQQDRRSDKRRRRRRRRKPEKDNRWQRQVQVAPVVRFTPVKRRKVFQGQTSQK

>DmelIR60b

MRRSLYLIIAIGLVDVHCVSLRYILNALENELQYRAILLVESASEIESCWEQKYIQGAVPILNFNANQSLYLKDALNTNILALVCLNENVESTMQALYENLEDMRDTPTILFVLSDSKVQDVFLECLRRKMLNVLAFKGLDRGFVYSFRAFPTFRVIERNVMDILQYFEQQLEDLGGHTLTTLPDNIIPRTVVYKSPDGSRQLAGYLYPFLRNYVSTINATLKVCWHLVPEDGMIQLGEVVRLSEIHDVDFPLGMHGIEHGSTSQNVPLEVSSWFLMLPMEPSLSRAQFFIMLGFEKVTPVLLLLTILLSTAHRIEMGLRPSWRCYVLGDRVLQGTLGQAFFLPRRLSVKLMLVYSLILLNGFTFSNYSITSLETWLVHPPSGHPIHSWEQMRTLNLKVLIVPSELDSMTKALGKQFTESNSDLFELSKSGNFQDKRLAMDQSYAYPVTCTLWPLLEHAQIRLPKPEFRRSREMVLIPLLIMAMPLPKNSMFHKSLNRYRALTHQSGLYEFWFKRSFNELVALRKIHYKVNGDHQIYRDFEWQDFSYVWLGFVGGTIASILVLLAEIGYHRWQLNQN

>DmelIR60d

MRLAIYVAFLSSIGNRSGFLSSLLMSLGKELHYKTILLVGGSSTCWSLEPFETGVPILNLRGENNAYPQDTFNSQMLALACLQTESEDAVKLLYRSLKDMRDTPTLLFASSEEHIHDTLFLGCFRENMLNVLALTASSKEFIYSYQAFPTFRVIKRKLVEIHRYFEPQLKDLGGHIVSALPGNIMPRTMCYRNAEGERQLAGYLNTFIRNYVESINGTLRISWGLVPEDDMRHLTISRLSKIQHVDFPLGIIPLYNKTDKQHVYMEISSWFLMLPMETSVPRAHLFVKLGLERLLPIIVVVGAVLGNAHRIEVGLGPSWRCYYLADKVLRGALAQPIVLPRRLSPKLMLIYSLLLLSGFFLSNYYMASLTTWLVHPPASDRILEWDQLRYLHLKVLTIPEEFKYMSLILGTDFMTAYGSIFQLTNSTDFQRRRISMDPSYAYPVTTSLWPFLELSQVRLRRPLFRRSYDMVLQPFQVMSLPLPRNSIFHKSLLRYAALTRETGLYYYWFRRSYYELVALGKISYKEEEGNPYCDLKWNDFRIVWLAFLGGTIISCLALLLEVAHYRWHLGNSSL

>DmelIR60e

MVIKMISFLLVSVLLCLVGASDSESMQVQVLQDLNLALQTELNVFIDFECCATSEILHKLDSPRILLSSNSREARDLRIRGNFTESTLIIVSVMDSDLNPLVASLLPRLLDELHELHIVFLSNEEPGFPKQDLYTYCFKEGFVNVILMSGKGLYSYLPYPSIQPISLSNVSEYFDRARIIRNFQGFPVRILRSTLAPRDFEYSNEQGGLVRAGYLFTAVKELTYRYNATIESVPIPDLPEYDVYLAVAEMLHTKKIDIVCYFKDFSLEVAYTAPLSIIREYFMAPHARPISSYLYYSKPFGWTLWAVVISTVLYGTVMLHLAARGARVEIGKCLLYSLSHILYNCHQKIRVAGWRDVAIHGILTIGGFILTNVYLATLSSILTSGLYDEEYNTLEDLARAPYPSLHDEYYRSQMKAKTFLPERLRRNSLSLNATLLKAYRDGLNQSYIYILYEDRLELILMQQYLLKTPRFNMIRQAVGFTLESYCVSNSLPYLAMTSEFMRRLQEHGISIKMKADTFRELIHQGIYTLMRDDEPPAKAFDLDYYFFAFVLWTVGLISSLLVFFAELVSGHL

>DmelIR62a

MYLQFLFALFLSRYQIVATENFDRAFELALFLDRIGRVHRLHAITIVNSLGSVDPSYLDDLHRGLMCNSSNHFYMLPQMTATDKDSSHVHFSSLQDEETIYLVFARDSKDAVIYLQAERARGRRYTRTMFLLRKQESQKDIKYFFELLWKLQFRSALVVVAARNFYQMDPYPTVRVIRMRRLSSYDPHHVFPPANRKNFRGYRMRLPVQQDVPNTFWYKNRRTKAWELAGLGGILINQLMMHLNVTMDLFRFEVNGSSLLNMAALTDLIVKGKVELSPHLYDTLQSNTSVDYSYPTQVAPRCFMIPLDNEISRSLYVFLPFSLTMWLCLLFVLLVVHFVYVRRLIPDGHFWAILGVPGAGQVRYGNRKPVRRFSTFLILFGIFILGQTYSTKLTSSLTVTLIRRPDNSLEELFLLPYRILVLPTDVYAIVDSLGHAEQFSTKFSCTDAENFSQKRISMHPEYIYPISTIRWRFFDMQQRFLRKKRFYFSKICHGSFPYQYQLRVDSHLKDALHRFLLHVQQAGLHDLWLDTCYRKAHRMGYLKDFSTLAELEEKLRLRPLALNLLVPAFSLFLCGMLGSGIAFLVEIRHSFGCRQKPPSINRNPGD

>DmelIR64a

MHWWLLVFLPLSCQGLPEHELLELELDYGLAEPQRTSLLQSSLILQFSQDYKHIPRITYFTCQKPHLQTPNQIPNAAEHRDAFAAKNFQLIKSLYESELFVRIVLLDVLAQSPSSGRPNRPGSGPTGGFSQTPSQAQSNSEWLEGVLRMEALRQIAVVDLACGAVSRRFLELASAKMLYSEKFHWLLIEDFAWHGRTQTAEGSGKRDDGEMEEEEPPGQQIQATDDEDLPSIESFLGGMNLYMNTELTLAKRMSEAAHYTLFDVWNPGLNYGGHVNLTEIGSFTPTEGIQLHTWFRTTSTVRRRMDMQHARVRCMVVVTNKNMTGTLMYYLTHTVSGHIDTMNRFNFNLLMAVRDMFNWTFVLSRTTSWGYVKNGRFDGMIGALIRNETDIGGAPIFYWLERHKWIDVAGRSWLSRPCFIFRHPRSTQKDRIVFLQPFTNDVWILIVGCGVLTVFILWFLTTIEWKLVPHDGSALIKPKGGAPPRHHYQQQQQQEQVEAPVRPITAVSVVVSKEKVEEKQEEYEDSTPIDAGTLWQRCYQKLNKYIKDRKAKQKKAPERVGLFLESVLFFVGIICQQGLGFSTSFVSGRCIVITSLLFSFCIYQFYSASIVGTLLMEKPKTIKTLSDLVHSSLKVGMEDILYNRDYFLHTKDPVSMELYAKKITSVPTTKENEADEDEPVDPNPASTDPAKSYRDIVHSHETGAHAKDNAASNWLDPETGLLRVKHERFAFHVDVAAAYKIIAETFSEQDICDLTEVSMFPPQKTVSIMQKNSPMRKVISYGLRRVTETGILTYHFNVWHSRKPPCVKKIETSDLHVDMDTVSSALLILLFSYAITLMILGTEILYSKWHNRIQLKWVGAT

>DmelIR67a

MLPILVPVLLLFNETSWINPILTSIYKDRHHETVLLLQHSQHGNASGLERFPWPVFSFNEQMDFYVRGKYNSEMLVLIWQTGNSDWDLDLWQALDRSLLNMRKVRVLLLRKWEKIPTADVAATAEHLLFLHVAVIGQGNRIYRLQPYAPQSWLQVDPIESPIFIKIRNYFGRYIVTLPDQFPPRSIVYRNPKTDEIQMTGYVYKFLLEFIRIYNFTFRWQRPIVQGERMNLILLRNMTLNGTINLAISLCGFETPSELGVFSDVYDMEEWYIMVPRAQEISIADVYVVMVSGNFLIVLIIFYFIFTILDTCFGPLLLKERVDWSNLMLNERMISGIMGQSFNMSARNTISSKVTNATLFLLGLVLSTLYAAHLKTLLTKRPTSQQISNFKQLRDSPVTVFFEEAERFYLKHAWDRPIRYIKDQLNFRETIEYNALRMGLNRSNAFSALTSEWMIVAKRQELFKQPIFTVQPELRVIQTSVLLSLVMQSNSIYEDHINDLIHRVQSAGIVEYWKHQTLREMITMGMISQKDPFPYVAFREFKVGDLFWIWLLWVSFLFMSFVIFLCELLVDCFISKTLIRNKRPH

>DmelIR67b

MELLYLNTLQSLSLLEGNRLVQTVQELNNIYQTELNVFLEFGNGADILESAQGTFVPTLWIKNPQNQKVMKGNFTSCTLTILYLEDEHLDRGLYYLANWLWEYHHLEVLIFFNGGSYDKLIQIFSRCFNEGFVNVLVMLPGSDELYTFMPYQDLKILNLKSIKEFYSLSRKKMDLNGYNITSGLVIAGAPRWFSFRDRQNRLILTGYMLRMIVDFTNHFNGSVRLMNVLTVNDGLELLANRTIDFFPFLIRPLKSFSMSNILYLENCGLIVPTSRPLPNWVYLLRPYAFDTWIAWLIMLIYCSLALRILSKGQISISAAFLKVLRLVMYLSGSRDMGTRPTTRRLFLFVILTTSGFILTNLYVAQLSSNSAAGLYEKQINTWEDLDKSDSIWPLIDVDIKTMEKLIPDRTKLLKKIVPTLEADVDTYRRNLNTSCIHSGFFDRIDFALYQQKFLRFPIFRKFPHLLYQQPLQISAAFGRPYLQLFNWFVRKIFESGIYLKMKDDAYRHGIQSGLLNLAFRDRHLEVKSNDVEYYYLIAGLWFGGLTLATVCFLLELLIGYAKIKVTISCKMNIM

>DmelIR67c

MFCWLIFLNIILLSDRSESWSAREVIHQFNHDQQLQLNIYLDCNDVELQIGQEVSNLFVNSTADKMKILGRFSSHSLIIACFKDSTRNRTLNGVKELLWGLQYLPILFVVDSNMDFYFQQALRHGFIHVLALNFMNGSLYTYKPYPKVEVHQIKDMQKFYKLTKLRNLQGQAVRTTVETMTPRCFRYRNRHGQLVYAGYMYRMVKEFISTYNGTEEHVFGNVDTVPYKEGLAALKNGEIDMMPRIIHALEWYYFYRSHILYNIKTYIMVPWAEPLPKSLYFIQPFRGTVWITIMVSFVYASIVIWWIRYRQQGNSSLTQSFMDVLQLLFQLPLSKIWHFNMGTHQVVSFIVLFVFGFMLTNLYTAQLSSYLTTGLFKSQINTFDDLFREKRTLLVESFDAEVLHNMTKEKIIQKEFESIILITSIEEVFKHRKSLNTSYAYEAYEDRIAFELSQQRYLRVPIFKILKEVYDQRPVFVALRHGLPYVELFNNYLRRIFESGIWIKLQEDSFLEGIASGEISFRKSKSREIKIFDKDFYFFAYILLGMGWCVSTIALFLELWSFKYSVTNVLHEG

>DmelIR68a

MRCLWILIVAFISLAMATSIPIPIANPAPLSGYEMQLKILLQKILWVANVKRCFAVITDDLHYPIYDRIFFESVGRRVIPFFVMRTNESDDLQRPSRQVELFVKAIKSSDCELNVITILNGWQVQRFLGYIYDNRSLNMQKKFVLLHDLRLFESDMIHLWSVFIDAIFLKRQLDNKYTISTIAFPGILSGVLVMKNIANWELGKGLNGRILFADKTSNLFGTSLPVAISEHVPMVLWANATKSFQGVEVEIMNALGKALNFKPVYYKPNQTENMDWTELDGGASVAYGSGNPDGYAQNGTHIDSMLVDEVAAHSARFAIGDLHLFQVYLKLVELSAPHNFECLTFLTPESSTDNSWQTFILPFSAGMWVGVLLSLFVVGTVFYAISFLNAIINGNVSSEFFRCLRPNRNVPMDPKIYRRISFRIAISRYRSSKGDRMPRDLFDGYTNCILLTYSMLLYVALPRMPRNWPLRVLTGWYWIYCILLVATYRASFTAILANPAARVTIDTLEDLLRSHIPPSTGATENRQFFLEANDEVARKVGEKMEVFGYSDDLTSRIAKGQCAYYDNEFYLRYLRVADESGSALHIMKECVLYMPVVLAMEKNSALKPRVDASIQHLAEGLIAKWLKDAIEHLPAEALAQQEALMNIQKFWSSFVALLIGYVISMLTLLAERWHFKHIVMKHPMYDVYNPSLYYNFKRIYPQH

>DmelIR68b

MKFLVGLLLQWYLPGIYALAEIACRIAVEQNVQVTYLYRCASCPASFDADYSALELDLYRCVGSRLPVITRNMEAHELEPFRRTDSLSIFQIPAAEKGDSLVRRILDMLNPHQRRKHMHKYLFVWPNAGRHQLLRLFRGSWAKKLLYGLAITGRENGTFDFDPFAWGGLQVIQRLDGEVPYARKVKDLRGYPLRFSMFTDPLMAMPRSPVETAGYQAVDGVAARVVGEMLNASVTYVFPEDNESYGRCLPNGNYTGVVSDIVGGHTHFAPNSRFVLDCIWPAVEVLYPYTRRNLHLVVPASAIQPEYLIFVRVFRRTVWYLLLVTLLVVVLVFWVMQRLQRRIPRRGVIQFQATWYEILEMFGKTHVGEPAGRLSSFSSMRTFLMGWILFSYVLSTIYFAKLESGFVRPSYEEQVDRVDDLVHLDVHIYAVTTMYDAVRSALTEHQYGLLENRSRQLPLGIATSYYQPVVRRRDRRAAFIMRDFHARDFLAITYDSQAERPAYHIAREYLRSMICTYILPRGSPFLHRLESLYSGFLEHGFFEHWRQMDLITRVGASPDAEEFLEDLGDQTDTDSGSNELAIRNKKVVLTLDILQGAFYLWSVGIGISCLGFAVEHAHWFWRRQTLRNAVEARTS

>DmelIR76a

MENLLVESYYFSNVLSFFAQQFFADSHATCIFWHPAFDFRLETVHPMPLIIMDWHRWANRSHQDVYDYKIKEDEFEGKGIPYNDWTLRLTVAIERSHCETFIAFQEQIPEFARYFYHASIYSIWRSLRNRFMFVYTKEFEDKKDSYLSGYIFQDQPNILVITSQYLNSSTFEVKTNRFVGPRNFNKNPEPVEFYILQRFDAKGTKATWETQSAMSSKMRNLKGREVVIGIFDYKPFMLLDYEKPPLYYDRFMNTTDVTIDGTDIQLMLIFCELYNCTIQVDTSEPYDWGDIYLNASGYGLVGMILDRRNDYGVGGMYLWYEAYEYMDMTHFLGRSGVTCLVPAPNRLISWTLLLRPFQFVLWMCVMLCLLLESLALGITRRWEHSSVAAGNSWISSLRFGCISTLKLFVNQSTNYVTSSYALRTVLVASYMIDIILTTVYSGGLAAILTLPTLEEAADSRQRLFDHKLIWTGTSQAWITTIDERSADPVLLGLMEHYRVYDANLISAFSHTEQMGFVVERLQFGHLGNTELIENDALKRLKLMVDDIYFAFTVAFVPRLWPHLNAYNDFILAWHSSGFDKFWEWKIAAEYMNAHRQNRIVASEKTNLDIGPVKLGIDNFIGLILLWCFGMICSLLTFLGELWRGQG

>DmelIR76b

MATGIELLVAAALCVACPPLNDSPPTNLIQMGENGTLSPVTELPMDVDASEAGFDADAPVETLETINRKKPKLREMLDWIGGKHLRIATLEDFPLSYTEVLENGTRVGHGVSFQIIDFLKKKFNFTYEVVVPQDNIIGSPSDFDRSLIEMVNSSTVDLAAAFIPSLSDQRSFVYYSTTTLDEGEWIMVMQRPRESASGSGLLAPFEFWVWILILVSLLAVGPIIYALIILRNRLTGDGQQTPYSLGHCAWFVYGALMKQGSTLSPIADSTRLLFATWWIFITILTSFYTANLTAFLTLSKFTLPYNTVNDILTKNKHFVSMRGGGVEYAIRTTNESLSMLNRMIQNNYAVFSDETNDTYNLQNYVEKNGYVFVRDRPAINIMLYRDYLYRKTVSFSDEKVHCPFAMAKEPFLKKKRTFAYPIGSNLSQLFDPELLHLVESGIVKHLSKRNLPSAEICPQDLGGTERQLRNGDLMMTYYIMLAGFATALAVFSTELMFRYVNSRQEANKWARHGIGRTPNGQSVAPSRWLRGWRRLNSGHGQLLGASTHGQNVTPPPPYQSIFNGGSHGDPLNRWRRPLANGNALGNGVLLGGDSEGGVRRLINGRDYMVFRNPNGQSQLVPVRSPSAALFQYSYTE

>DmelIR75a

MQLVQLANFVLDNLVQSRIGFIVLFHCWQSDESLKFAQQFMKPIHPILVYHQFVQMRGVLNWSHLELSYMGHTQPTLAIYVDIKCDQTQDLLEEASREQIYNQHYHWLLVGNQSKLEFYDLFGLFNISIDADVSYVKEQIQDNNDSVAYAVHDVYNNGKIIGGQLNVTGSHEMSCDPFVCRRTRHLSSLQKRSKYGNREQLTDVVLRVATVVTQRPLTLSDDELIRFLSQENDTHIDSLARFGFHLTLILRDLLHCKMKFIFSDSWSKSDVVGGSVGAVVDQTADLTATPSLATEGRLKYLSAIIETGFFRSVCIFRTPHNAGLRGDVFLQPFSPLVWYLFGGVLSLIGVLLWITFYMECKRMQKRWRLDYLPSLLSTFLISFGAACIQSSSLIPRSAGGRLIYFALFLISFIMYNYYTSVVVSSLLSSPVKSKIKTMRQLAESSLTVGLEPLPFTKSYLNYSRLPEIHLFIKRKIESQTQNPELWLPAEQGVLRVRDNPGYVYVFETSSGYAYVERYFTAQEICDLNEVLFRPEQLFYTHLHRNSTYKELFRLRFLRILETGVYRKQRSYWVHMKLHCVAQNFVITVGMEYVAPLLLMLICADILVVVILLVELAWKRFFTRHLTFHP

>DmelIR75b

MLQLHNLILHNLIHMAKLSHVLILHCSLSHLALLAQSKNIFTQFQPLHSDIQLNDDFLNHNILKLGVFLDINCDKSGTVLDMASAKRFFSHRYHWLIYDRSMNFSVLESHFKEAQIFVDADVTYVTHDPFSKNFLLYDVYNKGRQLGGELNITADREIFCNKTNCRVERYLSELYTRSALQHRKSFTGLTMRATAVVTALPLNVSIKEIFDFMNSKYRIQLDTYARLGYQARQPLRDMLDCKFKYIFRDRWSDGNATGGMIGDLILDKADLAIAPFIYSFDRALFLQPITKFSVFREICMFRNPRSVSAGLSATEFLQPFSGGVWLTFALLLLLAGCLLWVTFILERRKQWKPSLLTSCLLSFGAGCIQGAWLTPRSMGGRMAFFALMVTSYLMYNYYTSIVVSKLLGQPIKSNIRTLQQLADSNLDVGIEPTVYTRIYVETSEEPDVRDLYRKKVLGSKRSPDKIWIPTEAGVLSVRDQEGFVYITGVATGYEFVRKHFLAHQICELNEIPLRDASHTHTVLAKRSPYAELIKLSELRMLETGVHFKHERSWMETKLHCYQHNHTVAVGLEYAAPLFIILLGAIILCMGILGLEVIWHRHCTLH

>DmelIR75c

MTSWPLYRLIVFNLLEINLSNLMVFHCWSIKEAFPLVEMLNQNGIFSQYIDVQNPDNLANVHKEYLDSDLVRLGVFLDLGCDKAELVTNQSSRARLYNQNLHWLLYDEAGNFTKLTQLFEGANLSLNADVTYVSREDEERFILHDVYNKGSHLGGKLNITVDQTLQCNRSHCQVKEYLSELHLRPRLQHRMDLSSVTFRLAALVSVLPINSSEEELLEFLNSDRDSHMDSISRIGNRLIMHTQEILGFKLHYIWCGTWSVQDAFGGAIGMLTNESAELCTTPFVPSWNRLHYLHPMTEQAQFRAVCMFRTPHNAGIKAAVFLEPFMPSVWFAFAGLLIFAGVLLWMIFHLERHWMQRCLDFIPSLLSSCLISFGAACIQGSYLMPKSAGGRLAFIAVMLTSFLMYNYYTSIVVSTLLGSPVRSNIRTIQQLADSSLDVGFDTVPFTKTYLVSSPRPDIRSLYKQKVESKRDPNSVWLSPEEGVIRVRDQPGFVYTSEASFMYHFVEKHYLPREISDLNEIILRPESAVYGMVHLNSTYRQLLTQLQVRMLETGITSKQSRFFSKTKLHTFSNSFVIQVGMEYAAPLFISLLVAYFLALLILILEICWARYAKKKFSTIIPQNQ

>DmelIR75d

MKVQVAHWLPLIFFLLVSGTPRVAGSWRSEYSRQDPDPKTRWGNQLPDMLVAYYRHHGVHSLMLVVCHTDIADFRLWKLWQHFNLNNFYVQVSTESSLRDLQHVDALDEHKDAPPPKSFHANNSTHWETSFLLPALPYKMGILLLEFSSECALNLLRWSAASEHNYFTTNRFWLLLTEDPGDIDLLEDPEIFIPPDSELRVLHYENVGNFSCSLIDLYKVAAWKPLKRTLVGHNIRNSRHVIHALQHFGSAITYRQDLEGIVFNSAIVIAFPDLFTNIEDLSLRHIDTISKVNHRLMLELANRLNMSYNTYQTVNYGWRQPNGSFDGLMGRFQRYELDLAQLAIFMRLDRIALVDFVAETYRVRAGIMFRQPPLSAVANIFAMPFENDVWVSILMLLIITTVVLVLELFFSPHNHDMSYMDTLNFVWGAMCQQGFYVEVRNRSARIIVFTTFVAALFLFTSFSANIVALLQSPSDAIQSLSDLGQSPLEIGVQDTQYNKIYFTESTDPVTKNLYHKKIASKGENIYMRPLLGMEKMRTGLFAYQVELQAGYQIVSDTFSEPEKCGLMELEPFQLPMLAIPTRKNFPYKELIRRQLRWQREVSLVNREERKWIPQKPKCEGGVGGFVSIGITECRYALGIFGCGAAVSFVLFLFEFIFRHFKQVYRIIKGYREVQR

>DmelIR84a

MIKLQVKVISWPLIILTAFLRVLQIESINTNFLELAAFEDFLRSEHLSHVLVVRGDDADGDWKIECHQKLLANYRVQFYRPEMSANFEDLMFYGSPRTAVLVLNSEHVLVRRQVFGVASEAGYFNNSLAWFILGSGRESLPVEQLIDQLLSGYRMGIDADITVALRGPDNASMLFYDVYRISRQANTPLIIEKKGLWTHSGGYQKFGNFKNTWVIRRRNFLNVTLIGSTVLTEKPPGFGDMEYLADDKQLQQLDPMQRKTYQLFQLVERMFNLSLAISLTDKWGELLDNGSWSGVMGQVTSREADFAVCPIRFVLDRQPYVQYSAVLHTQNIHFLFRHPRRSHIKNIFFEPLSNQVWWCVLALVTGSTILLLFHVRLERMLSNMENRFSFVWFTMLETYLQQGPANEIFRLFSTRLLISLSCIFSFMLMQFYGAFIVGSLLSESARSIVNLQALYDSNLAIGMENISYNFPIFTNTSNQLVRDVYVKKICKSGEHNIMSLQQGAERIIQGRFAFHTAIDRMYRLLLELQMDEAEFCDLQEVMFNLPYDSGSVMPKGSPWREHLAHALLHFRATGLLQYNDKKWMVRRPDCSLFKTSQAEVDLEHFAPALFALALAMVASALVFLLELFLHWLPDFRRRLGTMST

>DmelIR85a

MSIQWLKHILLLAILVNLAGTRENHIPLDLKKSSIVMVKMSQILCKARIKVLFVYFENQTSHEHTGQILKEVTKCDISNQNTPLEAVKDDGILMYMVMITTNISQPLELSLIRKKSAAKHRSHVFLLVRDADTVSDAWMRASFRQFWKIWLLNIVILYWRDGRLNAYRYNPFMDNYLIPVDNKPNEVPTLEQLFPKTIPNMQRKPLRMCIYKDDVRAIFWRQGTILGTDGLLAAYVAERLNATMMITRPHSYNNHNLSSDICFLEVAKEYVDVAMNIRFLVPDTFRKQAESTVSHTRDDLCVIVPKAKTAPTFWNIFRSFGSLVWALILVSVLVANVFCYILKSEVGRVPMQLFAGALTMPMTQIPPNHSIRLFLIFWLYFGLLICSAFKGNLTSMMVFQPYLPDINQLGALARSHYHIIIRPRHVKHIQHFLTLGHKHESRIREQMLEVSDTQMYEMMRNNDIRFAYLEKYHIARFQVNSRVHMHLGRPLFHLMNSCLVPFHAVYIVPYGSPYLGFLDSLIRSSHEFGFERYWDRIMNSAFIKSGVKVVNRRRGSGNDEPVVLKLQHFHAVFALWLVGIGMACIVLAWEHLTHNYNLAVTKRRD

>DmelIR87a

MSTPEQRFWLAALLFLLSQHSEVRGFGINLMKVQTEDKGQEACILALLRKYFDSGDGLSGSVLCINRNYQLPNIEEQLLRGVNNYENYPWSLLITNSREGPSPAKFLMNEKPQCYFLIVDNLEDEDLDEVFEHWKGMVNWNPLAQFVVYLASLEETDEEMNDLMVELLLTFINKKIFNVNVIGQSEENQFYYGKTVFPYHPDNNCGNRVISVELLDACDYPSEETDSEDENDEDEGDGAQEEDDGPQEEGDGEQEEEDGPQEQEDGDQAKGDEGQENDDGGLENKVENEFRIGASDDDELENDLSSNSSEPEAIIEEFFRAKFEDKFPRDLSGCPLTASFRPWEPYIFRNSEEQPVDDYYYGLQGDEDDYNDTSPNYGESDDESYADPGEDGDGAIPDTETQSGGKLKLSGIEYEMVQTIAERLHVSIEMQGENSNLYHLFQQLIDGEIEMIVGGIDEDPSISQFVSSSIPYHQDELTWCVARAKRRHGFFNFVATFNADAGFLIGIFVVTCSLVVWLAQRVSGFQLRNLNGYFPTCLRVLGILLNQAIPAQDFPITLRQLFALSFLMGFFFSNTYQSFLISTLTTPRSSYQIHTLQEIYSNKMTVMGTSEHVRHLNKDGEIFKYIREKFQMCYNLVDCLNDAAQNEHIAVAVSRQHSFYNPRIQRDRLYCFDRRESLYVYLVTMLLPKKYHLLHQINPVIQHIIESGHMQKWARDLDMRRMIHEEITRVREDPFKALTFDQFRGAIAFSGGLLLVASCVFAFELCYVKYVYRTEKRERKTKKITKKVHNIKIQHD

>DmelIR92a

MLLQPLVMHLSQLLRIIVGQYFAEFPSILIVYNNSASTTPLQLEYLSALELVLRELSKPIRLQWINVAFLKDLNDLEDQVMGALNSSVTEGFITILSQTHHFIHARYYATRNANVRLKDKRYLFLCEDESPAELLCMDILQFYPHHLMVRPGTETAPTGPTGPHPDPRRGGGASVSTKNKDDGEGGAGNKTTSPYRDINFELWTQKFVGAVGNLDALLLDAFLPNETFANRVELYPNKLLNLQRRSLLVGSITYVPYTITNYVPAGQGDVDPIHPQWPNRSLTFDGAEANVMKTFCQVHNCHLRVEAYGADNWGGIYDNESSDGMLGDIYEQRVEMAIGCIYNWYDGITETSHTIARSSVTILGPAPAPLPSWRTNIMPFNNRAWLVLISTLVICGTFLYFMKYVSYRLRYSGTQVKFHHSRKLEKSMLDIFALFIQQPSAPLSFDRFAPRFFLATILCATITLENIYSGQLKSMLTFPFYSAPVDTIEKWAQSGWKWSAPSIIWVHTVQSSDLETEQILARNFEVHDYSYLSNVSFMPNYGFGIERLSSGSLSVGDYVSTEALENRIVLHDDLYFDYTRAVSIRGWILMPELNKHIRTCQETGLYFHWELEFIDKYMDKKKQEVLMDLANGHKVKGAPQALDVRNIAGALFVLAFGVAFAGCALVAELLIHRMDLSK

>DmelIR93a

MNPGEMRPSACLLLLAGLQLSILVPTEANDFSSFLSANASLAVVVDHEYMTVHGENILAHFEKILSDVIRENLRNGGINVKYFSWNAVRLKKDFLAAITVTDCENTWNFYKNTQETSILLIAITDSDCPRLPLNRALMVPIVENGDEFPQLILDAKVQQILNWKTAVVFVDQTILEENALLVKSIVHESITNHITPISLILYEINDSLRGQQKRVALRQALSQFAPKKHEEMRQQFLVISAFHEDIIEIAETLNMFHVGNQWMIFVLDMVARDFDAGTVTINLDEGANIAFALNETDPNCQDSLNCTISEISLALVNAISKITVEEESIYGEISDEEWEAIRFTKQEKQAEILEYMKEFLKTNAKCSSCARWRVETAITWGKSQENRKFRSTPQRDAKNRNFEFINIGYWTPVLGFVCQELAFPHIEHHFRNITMDILTVHNPPWQILTKNSNGVIVEHKGIVMEIVKELSRALNFSYYLHEASAWKEEDSLSTSAGGNESDELVGSMTFRIPYRVVEMVQGNQFFIAAVAATVEDPDQKPFNYTQPISVQKYSFITRKPDEVSRIYLFTAPFTVETWFCLMGIILLTAPTLYAINRLAPLKEMRIVGLSTVKSCFWYIFGALLQQGGMYLPTADSGRLVVGFWWIVVIVLVTTYCGNLVAFLTFPKFQPGVDYLNQLEDHKDIVQYGLRNGTFFERYVQSTTREDFKHYLERAKIYGSAQEEDIEAVKRGERINIDWRINLQLIVQRHFEREKECHFALGRESFVDEQIAMIVPAQSAYLHLVNRHIKSMFRMGFIERWHQMNLPSAGKCNGKSAQRQVTNHKVNMDDMQGCFLVLLLGFTLALLIVCGEFWYRRFRASRKRRQFTN

>DmelIR94h

MLSNISFSSAPELVDLYGLVLKFLVSSETTLFYFNPTGQKCSWETLPRTILSNHPQIIWFREETYPGLYKRHSSNLFVMACLSSTSYDGQLQLLAESLTRYRSVRVLIEVQDKEGSFLASQILLLCQQHSMLNVVLYFSRWTRTLNVFSYLAFPYFKLLKQRLSGSLRPKIFINQLKDLQGYKIRVQPDLSPPNSFSYRDRHGECQVGGFLWRIVENFSKSLKGDTQVLYPTWAKAKVSAAEYMIQFTRNGSSDIGVTTTMITFKHEERYRDYSYPMYDISWCTMLPVEKPLSVEILFSHVLSPGSALLLILAFILFFLIVPQLIKCLGITFRGRLIGMASRIFALVMLCSSSAQLLSLLMSPPLHTRIKSFDDLLTSGLKIFGIRSELYFLDGGFRAKYASAFHLTENPNELYDNRNYFNTSWAYTITSVKWNVIEAQQRHFAHPVFRYSTDLCFSSETPWGLLIAPESFYREPLQHFTLKINQAGLITQWMTQSFHEMVRAGRMTIKDYSRTNLMKPLRIQDLRKCWVIFAVGLGTSTVVFTIELLLIYTNVFLNSL

>DmelIR94g

MSTAVNSVHSKLVSLISRGQELTSIFFYAPAKEKCHLEDTISSATWGLPLVIWRTDRTVILNGFIGEGLLVLACLPGFHWRALLGSLARSLKYLRQARILIELMQDRDEFLVSEVLQFCLSQDMINVNAIFDDFPETENLSSFEAYPSFEVVNQTFTPDTQVSDLYPNKMLNLRGGVIRTMPDYSEPNTILYQDKEGNKEILGYLWDLLEAYAHKHNAQLQVVNKYADDRPLNFIELLDAAQSGIIDVGASIQPMSMGSLSRMHEMSYPVNQASWCTMLPVERQLHVSELLTRVIPYPTLALLLLLWIFYEVLRGRWRRHSRLQSIGWLVLATLVSSNYVGKLLNLFTDPPSLPPVNSLAALMESPVRIISIRSEYSAIEFTQRTKYSAAFHLALHASILIGLRNAFNTSYGYTITSEKWKIYEEQQKRSSKPVFRYSKDLCFYEMIPFGLVIPENSPHRAPLHSYTLLLRQAGLHDFWVNRGFSYMVKAGKINFTAVGERYEAKTLTITDLRNVFIIYVSVLLISLILFTCELFVSWVNYWLGF

>DmelIR94f

MWQQVLLAETSNWFRSDVLQRFWTHLRVEIRFRTMLNYRLESCDCWFDNVLGSDNSTALLWNDQTYPHYLRRRQDTDILVVSCLRFHQYQEVLLALSLMLDQMRSMPVVLQLCGDEDSMQELNSARLLLKHSQDLKMPNVVLLSSTFFTSATLYSYEMFPEFNVQKLVYQAYLTLFPYKLGNLKGHPIRTVPDNSEPLTIVRKTLNGSIAIDGLVWQFMIEFAKHINATLQLPIEPHPEKSIKLVQILDLVRNQTVDIAASLRPYSLNVQRSSTHIYGSPMMVGNWCMMLPTERVIGSHEALTRLMKSPWTWLILLLFYSVHRFLAQKTRLRSSLIHLIKLLINLSLICFLQAQLSAYFIGPQKVNHISNMQQVEESGLKIRGMRGEFMEYPIDMRSRYASSFLLHDLFFDLAQYRNSLNTSYGYTVTSVKWELYKEAQRHFRRPLFRYSEEICVQKLSLFSLIQQSNCIYCYRSRIFILRMHEAGLIRLWYRRSYYVMVTAGRFPIGDLSTVHRAQPIRWTEWQNVVLLHGVGLLFSVVVFVIELTVHYANVCLNNL

>DmelIR94e

MDCPKWILSGLCLISLVSGATVIELLGTLKLELDFEYVLLMKNRNFSLSDQVWNGTSLTKDVMDEVQVPVLQFNENVSYFLHNSISRRLVTLGFMSDANLDEHRGLLTALVANLRHMTTSRVIFLVQSKASTDFLYELFRNCWRKKLLNVIVIFQDFETTSTFYSYSNFPILQIEERIYETSLQTLPIFPDRLRNLHGYEMPVILGGTAPRMIAYRNKKGNVVYDGTVGHFMTAFQQKYNVKFVQPLQAKNPLDFAPSMQTVGAVRNETVEISISLTFPTIPPFGFSYPYEQMNWCVMLPVEADVPPFEYYTRVFELAAFLLTLGTLVLISCLLASALSLHGYATNISEFLLHDSCLRGVLGQSFVEVFRAPTLVRGIYLEICVLGILITAWYNSYFSSYVTSAPKQPPFRTYDDILASKLKVVAWKPEYAELVGRLLEFRKYETMFLVEPDFNRYLALRDTLDTRYGYMITTNRWVLINEQQKVFSRPLFQKRDDFCFFNNIPFGFPLHENSVFMEPVQKLIMELAETGLYYHWITTGFSELIDAGEMHFVDLSPHREFRAMQIQDLQYVWYGYAFMVVLSSLVWLLENLAYTVKSKTIFPTHFMQRNKK

>DmelIR94c

MSKVFKLLVLPLIYLSLTKGSKNPQLKFLRELINVIEEGREIRTIMVIKHSRDEYCHLDQWNPRGSPILRTNEMGSIRISGYFNDQAVILACMGENSDYGLLKSLANAMDNMRQERIILWSEREPTKMLMDYISQQADRYNFAQIIIVTMNEDVDAVPSLHQLNPYPTPRFRQITNISNIRRTSFFGCGLSFQGKTAILKESVVSNIRFKVWSPSGPIPLSELKDYEIVQFAVKYNLSLKLYDQNESKSDHFDIQLGPLFITKDFPTQMAFVSPNTACSLIVIVPCSPKWRFMDVLHKLGVLKLIGCLLIAYAVFVLIETLILWLTHRISGREVRLTSLNQLLNPRAFRGILGLPFPEFRRSSISLRQLFLVISVFGLVYSNFVSCTLSALLTKPAQNPQVRNFKELRDSGLITIMDKYTHSFIEKHIDPEFFDHVLPHYLILQKKEALRMIWNFNDSYSYVMYTTTWKSLNTVQKSFDERVFCESESLTIAWNLPRMYVLGNNSVLKWMLSRYITYMPQTGIPDSWTEQLPKVLKLLYNVTSPRRIKEGAVPLSIQHLSWIWHLLFIGESIATLVFIVEILLQKSNQHTSNMRERSSEDDDFV

>DmelIR94d

MGQLHLLLVALVLLSPGGDSFYHSLIHHLNRELKIEYVLLLGNFDTTWLDILWQLPVSVLQIKEHSRETYSLLENPSHNVLTIAFVNDSPEDILEILYRNLRMLNTQPVLLVIRKSTIRVNSLLEWCWHHQLLKVVAIAQDFMESLIVYSYNPFPVLQFIERRLDNSTVIFEKRLENLHGYEVPIALGGSSPRLIVYRDLEGKLIFSGPVGNFMKSFEQRYNCRLVQPYPFDESAISPARDLIASVQNGSVQIALGAIYPQVPYTGYSYPIELMSWCLMMPVPEEVPHSQLYSMVFSPMAFGITIVAMVLISLTLSMALRLHGYRVSFSEYFLHDSCLRGVLSQSFYEVLRAPALIKAMYLVICLLGLLITSWYNSYFSTFVTSAPRFPQLTSYESIRHSNIKIVIWKPEYEMLLFFSENMEKYSSIFQLQEDYKEFLHLRDSFDTRYGYMMPMEKWSLMKEQQRVFSSPLFSLQDDLCVFHTVPIVFPMVKNSIFKEPFDRLILDVTATGLLSRWRDMSFTEMIKAGQLGLEDRGHPKEFRAMKVGDLIQIWRFVGWMLGLATIVFLLELICFWRHKMWQNMKYMFCRNKNI

>DmelIR94a

MALPKQLKFINIFLVLLIIYGSSDGTENQHEIFLNRLLQAVHNERSVETLFLLHHSNLANCSLQDWNPPRIPTIRSNELTVFNVEKTFNHNALALVCLMKNSYREILNTLAKSFDCMRQERIILMIHRKSDSKFIEDITHEVKNLQFLHLIVLIVQEKYNGQVFASTLRLQSFPEPHFKRIRNVFAIQRIFYRPINFHGKVLNAIPNDIPILFVALNEMFTEYARRYNSTLRIQNRTIKEDIEITEDNYDIDMKIQLHNSQNFLHHMNIAMDIGSNSLIILVPCATELRGLDIFKELGVRTLTWLALLFYIIFVLVEMLFVFISNRFNGRNFTMRYTNPLINLRAVRAILGQTSPISNRYSLSIQHFFVFMSLFGTLFGGFFDCKLRSFLTKRPYYSQIENFSELRKSGVTVVVDHTTRQFIEQEINANFFRDEVPNVRTTTIQELINHVYSYDRKFAFVANSIPWRTFREEMKSINQKILCDSKNLTILENVPLTFSIRRNAIFSHHLRNFIINAADSGMITCWFKMAGKVIRKHIKTTLRESEQQPSHLPLSFDHFKWLWAVLCIAYVMSFMVFVMEILWSKYQRRTRSVSIV

>DmelIR94b

MSLIFNLLFILILSQAVSQETEFLQLKYLNNIVRSMIKLHKMETLVIVKHHLDNNCSLQNWNAHGMGIIRTNDQGKLIMKDTFNSRTLAIICIGQNSHITLLRNVFETFGKVQQKKIILWTQMELKEKFFQEISKKSRDLKLLNLLVLKAVTKDKLLIYRLNPFPSPHFKRIENIWTPNDTLFMDTKFNFHGMTAVVKHDYNWTIQMGNIRKFPISRIEDKEVIEFALKYNLTLQFFNDVERFDIELRKRIILKSNSTQPIDSGIPMVFSSLLIVVPCGNYLSIQDVIKVSGIEKWIFYIILVYVIFVLIEITFLGVTILISRQSRHQMIPNTLVNLCAFRAILGLPFPETRRTSLSLRQLFLAIALFGMIFSIFINCKLSSMLTNPCPRPQVNNFEELKTSGLTVVMDHDAENFIEKEIGVDFFNQYMPRKVTLTFTERAKLLFSLKGNHAFTLFSESFAIIESYQRSKGLRAHCTSEDLIVAERVPRIYILENNSILDRPLRRFIRQMQESGITNHWLKNIPSSLEKNLMQITIPYDRERVHPLSIEHLTWLWCILILGYSISMIVFFVEMSLKRRKKNLENRAPNICIC

>DmelIR100a

MATTLQLIMLALVGGTLGQANNTDHKQVLTSIVKQLEGGLELHLRTSEDGGNDLVQFLMQEKSSIIISAKQEEVPSRAKIMRHHFFIFDGVHQMQEIRTSLFNTDGFYILALENNTIEDDVLLMEFAADVWLQHGHSRIYYVQLSKKSVLLFNPFLQRLVVVQDSKTYSRIYKDLEGYHLRIYIFDSVYSSVIGDGENKVLSVTGADAKLAKTVARQLNFTADFVWPDDEFFGGRLANGEYSGGVGRAHRGEVDIIFAGFFIKDYLTTHIQFSAAVYMDELCLYVKKAQRIPQSILPLFAVHMDVWLCFLLVGLLGALVWLILRAVNLILGIEGVPDGSRATRISYFGAARRIFVDTWVIWVRVNVGRFPPFHSERIFVASLCLVSVIFGALLESSLATVYIRPLYYRDVNTLRELDESGQPIYIKHPAFKDDLFYGHNSEVYRRLDAKMMLVAEGEERLIEMVSKRGGFAGVTRSASLQLSDIRYVMTKKVHKIPECPKNYHIAYVLPRPSPYLEEVNRIVLRLVAGGIVGLWTGEAKERAKWSIQRFPEYLAELDVGRWKVLTLSDVQLAFYALTIGCLLSAIVCMAEILLGRQRRLHSPK

>SlitIR1

MLAQKLTKENVRVSVRRLNGDNVDVVRVAHQTTVPVGVLVDGHCDQTQTLMNQASFNKLFDAVHSWLILTDFEDDNCTEYVMQTFQWLNLSVNADVAVVANRGDSFAIIDVYNFGKIQGNHLETALLGTWQPDQGLEIILKGYKYYNRWDFHNLTLRAISVIVDQPKVFYPEMLSEMTYTSGVAAMTKITTQMLNTIKERHNFRFNYSIASRWIGSPERNSTMAVTNTLFWEEQDLSSTCARIFPKWLNWVDIYHPPTTNLQTKFYYSLIPETGCRGQYEGTGFLTLGCSHGGLGGCVLLSPGIPRTRGSWQPRLRMEKQTQARDCMPSFRVFAAGLSTRLGRRRSGCWSQTLSSQGRRTNPASDRTDEHAAVQLTTPAVWCPGCWTLRAPFPSANLEGLINSDFELVLEDIGYTRGWLDNPGFFYYSGFKNVKEDELRDKKVTKAKRTVSVLQNVNKGVELLRTGKYAFHTEPYTASQVISKTYEDKELCNLGALQMMLPAHVYIMAQKKSPYKEFFDWSLLRLLERGHVKAIRARFAGTMSACSGAQPRALALGQAAPAFLMLASFAVLSCFILVLEVLWKRVQLKNRGQ

>SlitIR87a

MTLITRTSEILENSGVVYRNGTATGAFEVLRNETADLVIGNVEVTRVLRKWFHPTVNYLQDEMTFCLPKAQQAPTWDNLVIIFQWSTWVATFLSLVIMGLVFHFFYYREHSNATKWPTNSLLMTFSMLLGWGATFEPKSPTFRILIFAWLCFSINMGISYESFLRSFLMHPRFEKQISSEADLIQSGIPLGGREIYRSYFETNNASSFYLYRKYNSTTFSEGVRRAALQRNFAVVSSRRQAVYQDQKLGKGAPLIYCFPESNNMYKYGVAILTRRWFPMLERFNNIIRSVTENGLIDKWMNELLIHTVSSEEASTIVPLSIQNLLGAFMFIGFMYACSIVIFLGEVIMGVIGKRRRVKKFKCKCSW

>SlitIR76b

MAGIELIISSICNATFCEVPYNETYQAPDSLAEKDTNFMSLMKEVNGKNIKVTTYNNTPLSSTELENGTVVGKGVAFTILNILRKKFNFTYEVVLPTKNFELGAKISDDSIIGLLNSSKVDMAVAFIPTLLPYREWVSFSIDLDEGVWVMMLKRPKESAAGSGLLAPFNDLVWYLVLAAVLTFGPCITFFTRVRSKLITDDEGVLPLKPSFWFVYSAFLKQGTNLSPEAHTTRVLFVTWWLFMILLSAFYTANLTAFLTLSKFTLAIETPKDLYQKNNRWVASAGSSVEHVVKTEGEDLYFLNAMISSGKARFLSVAGDKDFLDFVKKGAVLVKEQTVVDHLMYNDYISKKDVEESEKCTYVVAPSAFMKKQRAFAYPVGSKLKGLFDPVLTQIFQAGILDFLKRSDLPSTKICPLDLQSKDRKLRNSDLIMRYMVMVAGSATAVAVFGAEVFIKRYVSGKLNKNKKSKRKKSKTGKSLKSHDDSRPPPYDSLFGKNPKFNVETTRMKMINGREYYVFETSNGDKKLIPARAPSSFLYRSDK

>SlitIR75q.1

MKYLTFVLNIICLDYCVTFNTNTELQIIVDVAKSYDKPTSVIAKMCWETSKRSKYAPLEAKLAKMLANLDRPMNIRYLRQNETIDNDNYPNNHLLFILNRTCDDANAFLRWASANHKFRKSHRWLILGKSLIIKDETFNVSPEFDDIRISVDSEVIIIGKINNSEEVSLHTFYKLKPHTKWIIEDYGTWSFDTGFTKSTTRIESNVIRRKDFMGETLITSVAISDNRTKTDLLGLGNIFIDTPAKSSFRTIVPLFDFLNATKVVKSLILGVLINGSGMEGLVILSGRSRLVWNCNVHNERTYDNFRIFDPSYTHHTEVCLQTTALVVPEQFIPSAIFHWCLAVHWCIHCYINCHIVRQHDMGFKEIQRLNKQKIDQTCLPPTWSDITIFVLSAISQQGSSNELKGTLGRLVMFLVFLAFVFLYTSYSANIVVLLQSTSNQIRTLSDLLHSRLELGLEHAPFNKFYFSSAYTADDPIKKALVDTKIAPKGVLTNVMNIEQGVRIMQKKPFAFNMNTGXRVQNCFSNLKNHGKVSGLQEIEYIPNSNPWLCSRRLSPYGELFKVGYIRIQEHGLSDRENRLIYAKKPACTVMGGSFGSVNMVDLHPVCLVLLYGMILAFLLLGVEILVHRKQMKMRNQARVECNVKLCSKVASCEIRFWFCIAYKMCSNDAFIAKILLIFFTVNRKLLMASPPLTSVTGDPMAFNVLIDDFH

>SlitIR75q.2

MKKITFIFIAFFFSSINGKTEQANMIVDIIQAANRPSSVIGKLCWTPTKIIHLSSALVKENIQFSANADLTNDYMQFYDEEQQIVFLADLNCPDIEDYFQMNSTRTFFRAPFRWILFGDTGSVNEDNIVPEAIANVDVLIDSEVLVVRSIDDAYEMHFIYRISPNNTWNTEYYGTWDSENRFQKSNRFVEPTSLRRLDINEYEISICYVLTNNNSINHLSDGLDDHVDYEFTKVNFPTTNHLLDFLNAGRKYIFAETWGYKVNGTWNGMTGYLVRGEVEIGGSPMFFTFERVSIVDYISSPTPTRSKFVFQQPKLSYENNLFLLPFNTTVWYCTVGLVFVIYLVLLLVAKWEWKKTKHTIETREKDAGVLRANVVDVIILIFGAACQQGSPSELNGRWLGRIVMLVLFLALMFLYTSYSANIVALLQSSSSHIKTLDDLLHSRIKFGVHDTVFNRYYFSTATEPVRKAIYEKKVAPPGTTPRFMTMDEGVLQMRKGLFAFHMETGVGYKFVGKYFNEGEKCGLREIQYLQVIDPWLAVRKDTPFREMFKIGTKRIQEHGLQYRENRLMYEKRPKCSGGGSNFVSVSMVDCYPAILILSYGTIVALFFLGLEILVHKREKVLLKLKCLKRKLRLMTEVILRRNALDVEPLVVEL

>SlitIR75p

MTGISIVLFFLVTQCSVLIQSKDMENINFIKLFILNDQKPTHLIYGGLCWKKELINKLVVEMSNIGVRTSASFKPRSKYQDHAIMYLTDLDCAQSRTVLSYASSKELFQFTYRWLILVTSPQLQQSKISLLENGPVLVDSDVVLAERVGNMFKMTELHRPGPNGSMISTPRGYYNGSVVDVRAHRELYRRRRNMRGHAITMSNVIQDSNTTRLHLPREDRLKLQYDSITKACWSAAKIGFEMINATPRYIFSYRYGYKVNGQWSGMIADLYANKADMGTNCVIFRDRFDVVTYTDLVAPMRMLFIFRQPPLAYVANVFYLPFSTRVWVTIAVCTAIATVTLFLASKVEIVITKTTTQQQLDGGICDVLLLTMSAVTQQGCYIEPRRAPGRMMVFVLFTALMALYAAYSANIVVLLQAPSDSIRSLPQLANAKITLAANDVDYNHFVFNQSREPLYISIRDRVFPENGKAKLYSLADGVERIRQGLFALHSVAEPVYRQIEATFLESEKCDIATVDYLVTFDSFTPVRKGSPYLELIRVVHKQIRESGIQSAIRRRYLVSKPHCTTKMSSFSSVGLMDMRPVLILMLYGVAVSVIIVFGEIIVHKLINRYYKQKSKVQMVKTIHY

>SlitIR75d

MELISFILSYFITKDLSMMTAFICWPSEQALELQRSARVAGVRLTVVSELRHSAPMTTSGYFREAMLLDLNCPDTHFVLEKASRSRVLNKRHSWLLLHNSSAEPALVEETLYAYEILPDADVVWSSPNSLVDVYKTKPNQPLLQVQLGLSRNSSHQELLSLWGALPTAVTRRRDLRNVSLKGISVVTEPDNFKGWADLRNRQIDTFPKFTYPLMMLLAQDLHFRFDLRQVDFYGVSHNGSFDGLVGHLQRREAEVGLASLFMRHDRMQVADFFSETCVLACAFIFRQPSRSAVSNVFLAPFSAGVWGASACVAASAALLLVALRRLRQHTRASTDLQLFTLLEAVTFALGSMCQQGFHRTPPVTSVRLVMFSTLLTSLFVFTAYSAKIVAILQTPSTALQTIDDLVRSPMTIGVQDTTYKTVYFLESPEKSTQQLYRHKILPQGERAYHSVVDGIARVRTGFFAFQVEKSSGYDIIKQTFTEREKCSLSEIEAFKPPLVAVPMKKHSGYRELFASRLRWQREVGLMDRARHVWLVS

>SlitIR68a

MLRILIIFVITSTYNSRLQVESFPIIKDLHERRDLEFVLIDLLNVLTRDYEVTCIAIICDEVYLNVFGGPLFKRTASVPYVMTVVEDYEDLLSPNFVTLESLRAARKEGCNVYVILLANGLQASRLLRFGDRHRILDTRAKYIMLHDFRLFRSELHYIWRRIVNIIFVKYHKKILGVSKSRPWFELSTVPFPNPIKGVFVPRRVDIWKNENFYYKRPLFADKTSNLNGEVLNVVYLDHVPSVVVVKNNGSNKIGGVEVEILHTLAEKMNFKPKPYQAINAELHKWGQKQPNGSFSGLLGEMVNGRADVALGNLQYTPYHLELTDLSIPYTSQCWTFLTPEALTDNSWKTLILPFKLYMWIAVLLVLLITGTIFYGLAKNHMNLQEYKKLRPIQTKDDEGIDAKPGLYLFGEIINSILYTYGMLLVVSLPRLPTGWSIRLLTGWYWLHCILLVVSYRASMTAILANPAPRVTIDTLRELVDSKVTCGGWGTQSKKFFQESLDENTQKIGDKFETIDDPMKAASKVAQGVYAYYANSDFLKYISVTRKDALKGSKGNSTNTTDIAPKIDSQRNLHIMSDCVVNIPISIGFHKNSPLKPLADVYMWRVVEVGLVEKWLNDVMHPIHSLETNEDELKALMNLKKLYGAFIALAIGYTLSALCLAGELTHWHFIVKRDPNFDKYALHLYYRNKNKKDY

>SlitIR41a

MLLPTISLPLEILLNTIITQYLDSSYCVTVFSDKPLSPIISTSFIYLIPDEENLVEQIYNVSERGCSDYIVRMRDPQNFMTAFERVVHIGNVRRSDRKIIILPYNEEYNDNNDENLPSLIFSMKGSEYLANMLMVVNHNSSNSDCKEFDLITHQYVGPDDVSNLPKYLDRWDSCSQQFENNANLFPHDMTNLFGKTLRVACFTYKPYALLDIDTAIEPLGRDGVEIRIVDEFCRWVNCTVEVVREDVDQWGEIYKNESGGIGVIGSVVKDRADLGITALYSWYEEYRVMDFSVAGVRTAITCIAPAPRLLSSWEMPLMPFTWYMWLAVVFTYFICLNWDFNSTGIWFIIVSILNAFGMMIGQSQYEGKPSWKIRSVTGWLLIAGLILSSAYGAGLASTFTVPRYEPSIDTVQDIVDRKMEWGATHDAWIFSLTLSTEPLVKELVSQFRIYSFDELKRKSFTRSMAYSIEKLPAGNFAIGEYVTQEAILDMMVMLEDFYYEQCVVMMRKSSPYTEKVSQLIGRLHQSGLLLAWETQVALKHLNYKVQVEVRLSRSKNDVGTTKALNLGNVMGIFIVYAIGLMLSIATFLGELYVHHHKQKKERIHVD

>SlitIR40a

MRRQRVQRVRNVEVGVRLVLSHKRVAVLGGRETLYYDTERFGSHNFHLSEKLYTRYSAIALQIGCPYLETFNNVVMTLFEAGIVAKMTTDEYKNLPEHARRSDPVTESDKQGGEVMGESAATSSQTPQGESTKGLQPVSLRMLRGAFCLLGIGHLLAAISLAVEIQLHRRSKRRRKPEHNEHRKAQKLLVLGKSVMLFKRGCKKVCTSVFTSIDKALGSDNKDYFDKMAPDLGNLFLIVVCLLTSRVFLVHTGVLCRIFSSLQPVLYLIEQNSYFAVSIKIFTRVSENARLPVCTRGAREGNSNFVFKCFFYLSRLKVVATVIVK

>SlitIR25a

MNGEPEITSAFYFDLSLRTFLTIKSLLDSGKWPNDMKYITCDDYDGKNTPNRTLDLKTAFQEIKETPTYAPFYIPQDDPMNGRSYMEFSTDLLAITVKDGASISSHSLGSWKAGLSSNLTLTDPNNMSNYSAQLVYRIVTVEQKPFIIRDDQAPKGFKGYCIDLIEEIRAIVKFDYEISLAPDGNFGTMDENGNWDGIIKELVDKKADIGLSSLSVMAERENVVDFTVPYYDLVGITIMMKLPRTPTSLFKFLTVLENDVWLSILAAYFFTSFLMWVFDKWSPYSYQNNREKYKEDEEKREFTLKECLWFCMTSLTPQGGGEAPKNLSGRLLAATWWLFGFIIIASYTANLAAFLTVSRLDTPIESLDDLSKQYNIPSATVSMDLRLMTCFQRGGYLGGSFLKFGGSWAKRPFKGGGTAQNLRYGIIPVRDKYIKFWRAMEEAVLPLPFLGKLYRGVRDSKSFSEGFAWLGDATDVKYHVMTSCDLQSVGDEFSRKPYAIAVQQVSPLKDHFNNAILQLLNKRKLEKLKEIWWNNNPESMKCEKQDDQSDGISIQNIGGVFIVIFMGIGLACVTLGVEYWWYKWRKRPAVGDVTQVEPAKLTRNNVDKQGEGFNFRGRNLGLNFKPKF

>SlitIR21a

MAVPWFYVVFLAYHVVFGAEVIIEYYPSQSVLDMNNKVVRKREVNNTDDPNLNINGSDSYWRHFNNDTDDGDIHKRALDPVFYGHPKTREELWNERFLNETTSFDQTPSLVKLLHNITLTYLKDCTPVILYDNQVMSKESYLVQNLLKGFPTTFIHGYINDDGELVEPELIHATIECQNYILFLSDIKISAKILGKQPENKIIIIARSSQWAVQEFLASVTSRNFVNLLVVGQSFKEGDDAKLESPYILYTHKLYTDGLGASQPLVLNSWTHGKFSREVNLFPLKMTEGYAGHRFVVAAANQPPFVFRRIKSDLDGGNPRVVWDGIELRLIKLLAERNNFSIEIIEPREPNLGPGDAVAKEIVTGRADIAIAGMYLTNDRIREMDMSLAHSHDCAVFVTLMSTALPRYRAILGPFHWHVWVALTFTYLFGMFPLAFSDKHTLRHLIHNSGEIENMFWYVFGTFTNCFTFLGKNSWSKTNKITTRLLIGWYWIFTIITTSCYTGSIIAFVTLPVFPETVDTIKQLLAGFYRVGTLDRGGWEKWFLNSSDPQTNKLLRKLELVPSVEAGIRNTTKAFFWPHAFLGSKAELEYIVQANFTATKSKRAVLHISNKCFVPFGITIGFPNNSVYSAKMNLDISKMIQSGLIDKITNEVRFEMQRSPTGSLLAAGSGTINIPSAEEKGLTLEDTQGMFLLLAAGFTIAATALVSEWMGGFTRRCRFQKKSETPTSANSRDNLIITPKTDVDSEIRIIEDTERRLHFEERPSSSVSVDTLEGQVIHVTESSIDVHNTFNVDRFDSRRSSSLDLDREVREIFEKDQKRRRIFSRDMESLDENGSTVSRAAFGDSVKNDI

>BmorIR87a

MTTGNSDQIAKTAECVLKLSAKYFVERKALSGSIVIINVNSYSSTTQGLLLKTIHSSIKYSVMAKDSFYPHANASHFPEKAKNYMLILEERTELKRNIFQLNKLPSWNPLAKAVVFYQIKGNESAQRIAIEFINELREHKFFRSIIFINNGTESGVTSYTWRPYSENNCGGKCDSVYVLDRCKNNIVEQIEPQPEWFPSNMNGCPLTTYAIVSEPYVMPPIRKIPNAKFDDVYEFQKGGETNLVKTIAEFSNMTLIVRLSAIEENWGIIYANGTATGAYGVLRNDSVDIVFGNIEVTKQIRKWFHPTISYTQDEITWCLPKAGQASAWDNLVIIFQWTIWVATFTSLILMGLLFHYMYYREKNKKITKWPTNSLLMTFSMLLGWGSHFEPKTATFRILIFGWLCFSINMGISYESFLRSFLMHPRFEKQIATESDLIQSGIRFGGREIYRTYFESNDASSSYLHTEYSSTTFSEGIRRAALNRDFAVVSSRRQAEYQDQKLGKGASLIYCFPESDNLYKYSVVLLARKWFPMLERFNGIIRSVSENGLINKWNDEMFIHRVSLEGASTIVPLSIQHLLGAFMFIGFMYGTSAFIFLVEVFVGFVQRRAFLSAFFCGKKKRFSAVFKVKV

>BmorIR7d.3

MRTEPEDITLFLQHFHGSAVIVPLDYQNMKAVSELNKATGFKQTVLFAVSVEEFILFITTLNLDLIVPIRMVLVLTTQLTDLAMITKEAWKHDLAEIIIISKDENEEIRLTTYFPYKNGICGDYTPHSISNEKELFPEKFKNLHGCPIKVTLLNFLPYVGLQKVNGTITFIFGIDGSVFILLIKELNAIMDIVSSTDHGGMGVFVNGSWKGSFGDIVRREADIFAPAGIITQKRFSVAQMSHTYETLNIHWCAPPRREIYAWAKVLLPFLTNITPFLVLAFTVFVITIVLVKRSKLHGIKSNKNVFLQSFMIFLGQGVKFETKSSVINSFFVAWLWFCLIVRIAYQGDLVNGLQKKIYEPPFESVEQALQELDGYGGTELFREYYAGSPIADNYQVIKIGDLPRYIRDVIAGKRFLIATDILMHQYAKKFQILQEPLTHSPTCLFMRPGWPVSRRVDVIIIRAIEAGLVQKIIYDFHYTVRLRRHEKEEETGTRPLGMSTMFACYYGLILLWIFSFVIFLFEVLYYNWKHKIAYIKRKRNKLFKFHH

>BmorIR7d.1

FNLPLKLTILSLFLGIILLNMLRKTIFFNNIRRVCNITPPKRNSLFYAWLLFLGLPLEKFSSRKHFKIIILAWIWFSFVIRCAYQVTLVTSLKSITYNYNLRYDSDILKYPFGGMSSIRDYFIEDKDFYENWTSVDMQKAYKLLDEIMEEKTDFVLALNKDTILHHAAEHIGSKRIQVIDNCIVNSPIVLYFRKHSPMTDPIAKIMNAALECGFIQYSYQTNWKRQKHLLNSHYAYNLQPLTLDNFSGCFFLLIIGYGISILYFVLEVVCHKIDKTNQRIDLRVDQE

>BmorIR7d.2

MSPRNLSDASHFCEENSNEITTAALNIALHNFKWRILTYVFFNATFLCNLNIFLKTYNKGVVVGNGLVEPRIDGKIQQLVLFCDDIVGITLALNSLPNQFDETGKVIVICQSPISWKCSAEEAMRSFWSVKITNVVFLKKDVFVMAYTYMPVYNEQCEISDPIPLFGLKPCIINATKCGVFDKKLDNLNKCKIVVSTLIRRPFMIINNGIPEGADGDLLLLIMERLNATLEVIIPGDHNYWGKLDSNGTWSGSLGDVYYGAADISMTSAALTASIISYFKISIPYRSTNVVWISHPPKALSPALKLLHPFKPSTQIALGIIFFIVIACVLFVSSKKMWLLCCRRVRPTKKKPSLLFNTWMICIGVPIAHLPSTSTFLSLIVLWIWYCFLIRTFYQVWLINSLQGKFYLDGFEKIDEAIEAGYDIGGGIFLKEYFVDYPYIYNNWKETVSLNVTLHEISEGSNFIAATIYDLAKSLTNFEKINVHFLAEKVVVSPSVLFFNKNSPLVAPINELLQQLTESGFVEKISRNYFTHNVTNWKRQKHLLNSHYAY

>BmorIR41a

WINCTVQGVIGSVVEGRSDFGIAALYSWYEEWKAMDFSVSVVRSAVICLVPAPRVWELPFLPFKSIWIAVVITFVYASIGLTIAQ--GCKLLIVFGTIISQSQYIVSDSWRIRSVIGWLLVSSLILVSAYGAGLASTFTVPPSIDTVQDLLNSRMEWGFEELQRRSAFSLEQDMQMFYFDCVAMLHKNSPYTEKLSELIGRLHQSGLLWESQVSLNFNHVEGIFLIFITGTILSTLFFALE

>BmorIR68a

KMNFRPKGLLGEMVNGRADLALGNLQYTPYHLELIDLSIPYTSQCWTFLTPEALTWKTLLLPFLYMWIAVLLVLIT-GTIFYGLARYFGNILYTYGMLLVVSLPKLPTGWSIRFLTGWYWLYCILLVVSYRASMTAILANPVTIDTLVELAASKLTCGPNIAADKVAYYDNRNLHMVVNIISIGFHKNSPLKPLTDIYITRIVEVGLVWLNDAMMNLKKLYGAFIALAIGYFLSVMCLIGE

>BmorIR21a

KNNFSIEAVAKEIAKGRADIGVAGMYLTIDRTREMDVTFAHSQDCAVFITLMSTAYQAILGPFWHVWVALTLTYLFGMFPLAFSDKHSGNFWYVFGTFTGRNSWSKTDKITTRLLIEMVLDFTIIITSCYTGSIIAFVTLPETVDTIHQLLAGFYRVGVEAGIMNTAFLGSKVLHSFVPFVTIGFPNNSLYTAKLNNDLRRMVQSGIVIVDEVRLTLEDTQGMFLLLAAGFLIAATALISE

>BmorIR64a

MYNITYDGMVGSLQRHEADVGGSPIFFKTDRAYVVDYVAETWPSKQSFIFRHPKHHTVYSRPLNSVWYCVIAFLFVTASTVFFMLKFETLFLFAWSAICQQGMSLRRNSLALKVVVFVTFVCSITLYQYYNATVVSTLLKEITIRTLKDLLQSDLKVGPEYGMSLVAFHVDCEIHVYPPQMGAVLKKNSPYRNYFAIGIRRLWETGLMMKHIWDVSILEFSTPLFIVVFGVIASVVVLLCE

>BmorIR75d

DLRFRFNGTVGLLQRGRAELGVASMFMRSDRWRVLHFSSATVALLNAFMLRAPAQSNIFLLPLRGVWCCAAALLCGSAVLLAVLSCLLLEFVFSIGTVCQQGFYIMPKLSSIRMIMFLTLLTSLFTFTAYSAKIVAILQTPAAVRTVADLADSHMDVGVVEGVERMAFQVECGLMIFKLPVAVPLRKHSGYRELFGTRLRWQREVGLMVRAIWLVRLLDMLPALQMLAAGGLVAVVLLILE

>BmorIR75p

MLNATPRGMINDLHTSKADLGTNCVVSDVERLSVVTYTDMLAPFRVRFVFRQPPLANIFYLPFGRVWAAVAVCAMVYTAAIYWASKWDGDMLLTMSALSQQGCFIEPKRAPGRIMLFVLFTALMALYAAYSANIVVLLQAPNSITSLAQLAASKVTLALEDGVDMIAFHSICDLTVLSSFPFVPVKKDSPYLELLRVSFKQIRESGIQLNRRYQVGIVDLRPVLIMMIYGIISSCLILIME

>BmorIR75q1

FMNASHKGMMGDLAKGTVDFGGTIAFLTSQRLQVVDYLSSPVPINAKFVFREPPLNNLFLLPYANVWYCTAAFVVLLVIILYINAKWQPDTILVISAISQQGSSNELKGTLGRAVLFLLFLTFLFLYISYSANIVALLQSNKQIRTLQDLLNSNLNIGIEEGVKKLAFNMNCGLQIIESSPWMSCRKNSPFREIYKLGLFKLQEHGITENRLLFVNMVDVYPVILMFLYGLFLAFLILLVE

>BmorIR75q2

FLNAERKGLTGFLVNGDVEIGGSPMFFTAERTAVVDFISSPTPTRSKFVFQQPKLNNLFLLSFTAVWYSTLALISLIFTMLLSVTAWRPDTMLVFGATCQQGSTVELKGSLGRVVMLILFLTLMFLYTSYSANIVALLQSSSQIKTLEDLLHSRLKFGMEEGVKKMAFHMECGLKILQVIPWLAVRKNTPYKEMFKIGMKRIQEHGLQENRLLYVSMVDCYPALLVLSYGIIIAIALVIME

>BmorIR40a

KLNFRYRGTLGLIWKRQADFFLGDVTMTWERLQAVEFSFLTLADSGAFLTHAPAKTLAIIRPFWEVWPLVCATLFITGPALWIVIAAMGNCWFTVTLFLRQSSTKPSSTHKARLVTVLISLATYVIGDMYSANLTSLLARPPPIGTLPALEEAMREHGVEAGVRLVAVLGGHNFHLLYTRSAIAFQIGSPYLETINNVVMTLFEAGILMTTDEYVSLTMLRGAFCLLGIGHLLAGVTLLIE

>BmorIR76b

KFNFTYESLIGLTNTSKVDMIAAFIPRLVRFRKLVTFSRDLDEGVWMMMLRRPKEGSGLLAPFNFVWYVTLASVLCYGPCICFLTHVPLPFWFVYSAFIKQSTNLAPEANTTRVLFATWWLFIILLSAFYTANLTAFLTLSLDIETPEDLYKKNYRWVPDQEYLPIAVLVKCTYVAFMKKRAFVYPVGSKLKSLFDPTLAYILQSGIILEHKDLLTNSHLMMTYYIMCVGLASGLAVFVVE

>BmorIR93a

NKNFTIRYNNIPLYFRAVFIHQAGVNLKNNYYRCINYTIPVSTQPHTFIVARPREALLFLLPFTDTWLCLGFAVILMGPMLYIVHRLLANLWYIYGALLQQGGMYLPRADSGRLVIGTWWLVVLVIVTTYSGNLVAFLTFPAPVTTISELLKNSYTWSARGTLDRVLIFDWCDFAAFMEEVAMIVPAGSPYLPVINKEINRMHKAGLIWLSAYLVNLSDMQGSFFVLFLGNDKIVYMYIAE

>BmorIR8a

AMNFDYEGVVGDLTTGETDIAVAALTMTAEREEVIDFVAPYFEQGILIAIRKPIRLFKFMTVLTEVWLSIVAALVLTGFMIWLLEKYDFEFWFALTSFTPQGGGEAPKALSGRTLVAAYWLFVVLMLATFTANLAAFLTVETPVSSLEQLARQSINYTAETGFKQVAFIHDCNLTVFAEQYAIAVQQGSRLQEDISRALLELQKERFLLTSKYWITLESLGGVFIATLFGLGLAMITLAWE

>BmorIR25a

IVKFDYEGIIKELIEKRADIALTSLSVMAERENVVDFTVPYYDVGITIMMKLPRTLFKFLTVLNDVWLSILAAYFFTSFLMWVFDKWEFELWFCMTSLTPQGGGEAPKNLSGRLLAATWWLFGFIIIASYTANLAAFLTVSTPIESLDDLSKQYIQYAIEEAVQRVAWLGDCDLQVFSRKYAIAVQQGSPLKDQFNNAILQLLNRRRLLKENWWISIQNIGGVFIVIFMGIGLACITLGVE

**GR:**

>LstiGR63a

IVTFISVAIRIFSGVMFPGLSSDKKIFIITACILIAICSSINIAWLVYRCEQSYGQRNTIIRIADHMLVDKNISESMRRTLSEFRNLVDSRPVQFTAMDFYPLSYGLVVSSASVVTTLTIILLQGLE

>LstiGR63a.1

KRFLSQIQINNNKQMDETKVGQNKQNESEDAFDSLNFINQLLKIFCLSILSRENRRLKISYSWFKVFFTIMCIICLIIFLTYDIVKFYAYEIQHFKFNDEVLLVILVRAVLYSIDLCYVFKFGGNTNLHYFKLYEQIDTILDTDNAMIKTKVLKVTVFITSLYGIQTVVNIIWAAFYDPTESFTTVRATVGIIMIYINSLSILEMLVHVILIEYRLIKINNILQLRCSSTTNNFGALSVLVENNWLYFSKHKEITRNPQVDCNYFYDISWLNKCYLLLIEQSNFINKLFGVRILLNSVINLWDLVNNINFSIRISFRVLDLDPETTILNILSSTLNISSVAAILICLVYRCEKTYEQRRSIINVMDRILVEKYINVSMRSRLAEFRTLVYFRPIQFTAAHFYRLDYALLVTFCSAVTTYSIILLQYLQ

>LstiGR6

VDVVSCQYLYKSVADMVEKVKKSFDVVLILTLLANTTDVIIHVYLPFAKEPFKSIVGYDLTLAYVVVVQQLLILFFPALTAGMLTGQVEKLKLVLCDMLIKDKSRKKDIKR

>LstiGR7

DAGFVKIGVPTARRKKFLPTLTHIFTVAQWFGIPSYGNKFAICWAIIVLCMLTVVEGAAIWMMIRLLAGIAKHIDDGRGLTARLSGSIFYANGFLSLILSWKFMYSWKRLSFYWKRAELVDVSLAIPDEAIQRKVIVVTCFVSVCAFAEHLLSMILAIGIDSPPMDFLERYILNSHAFLITPNTYSLWS

>LstiGR5b

MGLTSRYHRLNSFVNLCVKNEKINRDKTSVTEKYVRTHQWRRIREAYVRQAALVRMVDANIGALVLLSNVNNFYFICLQLFLGLTKSQGSLVSYLYYFISLGWLLFRACSVVLAAADVHIHSRRALEYLQTCPGTGFNIEIMRLNNQLSHDFVALSGMGFFSLSRQTLLEVAGNIIKYELVLIQYDK

>LstiGR51

LRTIFKALQNTNEVVMGKGMIAYASSRCLRYTVMVIIPCYYSSVTTTQVSYMRTMLHDAMNQVNIGKVDRRRVKAFFQLTRENEFAYAIWGVIRLNMSLPLSYLSLCTTYLVIIIQFAKFID

>LstiGR45

AVYLNIVVTGLPLNLIGNLEWVSVVSFVATNLFVALFMSIRCEIFLREVVETKQLCITILSMYTDGPIREKARKMLKLAEASPPRFSVYGMWNIEGRFLLYLFSIITAVMLTELQLLLL

>LstiGR5a

MGSSRVHFTGEDKMSDSKTVGAQNKLILPDQPYHDGFLETMSKTFHWARLFGIMGRESRRWNVWAIVLLITLLVIEVAAIWKVIKALAGWAVDTAAHRSVTARLSGTLFYTTVIASQILCSRLSLNWHNLSSYWVSVERAVAINIPTDQTMRKRMLTVIITMAILATVEHLMSVVALIGFDCPPHLILRRYTARSHGFLFLRDDYSVWFAIPLIFISNIATILWNFQDALVVLICMGLTSRYRRLNNYVSKICEEEKKLANKNMKAEAVRIYSWRRIREAYVKQAALVRKLDGALGGIILLSSFGNFYFICLQLFLGITQGLSNTSTIKQIYYIVSLLWILGRFTSMVLAAADVHVHSKKALPDLHACHSRCYNVEIDRLLNQLNKDYVVLTGVGFFTIDRNIL

>LstiGR63a.2

TMDNRRRALLPSRRYSSKNMIQVSDVKSVTSDQSWFNNPLSFLLQLFSLSTNAHRDKNFNSCLSLIRMIITGVGFGVLQLFDLYYKIGHVYSGLSVSVRLTDSVQTIYDYFQYTVDLFYVYKYGRHFYQEYYKQYNTIDQILRAGSCNAIRKKITKLVILFVSIWLITSVMDFIAWVLIYGWTIPTVFSLAYNYLLLKILTNLDLTYQTMHIEVRLQVISGLMQSYYTCCDSLPGGPGEKCGDPVQNKNWLYSNFSVPPKDSLKWSAESRRHGIRWLTRCYLLLKEQSAFINQMFGVRVLLNSLSLLIDMVRFSNLAIRLVMGLQQDGNGAKVTKYIVNGVKRNMHDWEYFTAISTVCRLLVCAVILTNLVHHCELVYRQTDRIISISDHLLINKNPDPDLREAVTELRDLVQSRPIDFHMANFIRLDYSMLMSTA

>LstiGR1

MGQLKKNLGFWIPIKKNKVHVTKFQTDGKQATFQKSLRMTLIIGQMFSLIPVTGIFSNSASNVRFVLKSWKCLYSTLSFCGQIFMTVMCVHKVVHTTTSLNGNAPVIFYGTTCITMIMFFQVGRSWPSLVRHIARNEELDPNFDPGLSYKCNVTCAIVLMLALLEHILSLLSAFAGAMVCHPDKAFYEGFVTHFYPWVFNVLPYSAVLGATTQFLHFQSTFIWNFSDLFVICMSYYLTSRLEHINGKLLAAQGKYLPEIFWKTTREDYCRATQLVRRVDEVISGIVFISFANNLFFICLQLFNTLEDGIKGTGECSSRSKSTPSNLLGGYEAATYFLFSLVYLISRSVAVSLIASQVNAASTVPAPVLYDVPSPVYCVEVQRFLDQVNGEHVALSGLQFFSVTKGLLLTVAGTIVTYELVMFQFTTSQPDDVSTNTGFNTTGAFSNISSTISYFIQ

>LstiGR21a

MDEEKQMFRIYNTNQINGKQKNTNGIREEYDAKDIYGPEITDKDGALLDEHDSFYHTTKSLLVLFQIMGVMPIMRVPKDAQTTNRTTFNWISKATLWAYLVWSLECIIVVRVGKERLATFQQNTNKRFDEVIYNIIFLSILIPHFLLPVASWRHGPQVAIFKNMWTHYQLKYRKITGTPIVFPNLYILTWGLCVFSWGLSFAVILSQHYLQEDFELWHSFAYYHIIAMLDGFCSLWYINCNAFGTASRGLAMNLHKALKAEHPALKLAQYRHLWVDLSHMMQQLGRAYSNMYGIYCMVIFFTTTISLYGALSEILERGLSYKEMGLFVIVGYCMTLLYIICNEAYHATRKVGFEFQVRLLNVNLGAIDRSTQREVEMFLVAIAKNPPIMNLDGFTNINRELFAANISFMSTYLIVLMQFKLTLLRQSARKAIKTVVKAIFNTTTLGPDDEDDDVEEE

>LstiGR21b

DYEQEQRDLLSSQDGDTCEIHDQFYRDHKLLLVLFRALAVMPITRSRPGTITFSWRSSATAYAICFYIASTIVVLFVGYERILILRSIRKFDDYIYGVLFIVFLVPHFWIPFVGWGVAHQVAIYKTNWGKFQDVGIDCSAKLISRYRYLWLNLSELLQLLGNAYARTYSTYCLFMFTNITIAVYGALSEIVDHGIGFSFKEMGLFVDTVYCSTLLFIFADCSHKSTQKVADGVQETLLTIDVLAVDRPTQNEIDHFIQAIEMNPAVVSLKGYANVNRELLTSAISMIAIYLIVLLQFKISLPKDP

>LstiGR4

MGVETSNEERGDVTPAPTLRSTEPTRSVVGGAHAFILRISSFFGLAPLRFESRANGFTVSISSVMCIYSFILVSILILLTIYGLVAEINAGVKLSVRMSSRMSQVVSTCDVLVVVVTAAVGVYGAPARMRKMLKLMDRIASVDNTLSGQYSAVMERKLSAILLALLIFFSLLIVDDFCFYAMQAKKVDREWEIVMNYIGFYLLWYVVMILELQFAFTALSVRARFRALNDVLALTARSIAVPVEKARKPTPLNIFAIRVTPTDLQRSDDISLLMTSTPKKRETVIVRRSVSGESRLLVSPSEAIYGLASLHGTLCELVHRIDDSYGIPLVVILISTLLHLIVTPYFLIVEIIVSAHRVHFLVLQFLWCATHLLRMFVVVEPSHYTIMEGKRTEGLVCRLMTSGPSAGPLPSRLELFSRQLMLRSVSYSPMGIC

>BmorGR9

MPPSPDLRADEPKTPCLVGGAHAFILKISSFCGLAPLRFEPRSQEYAVTISKGKCFYSYILVTFLVICTIYGLVAEIGVGVEKSVRMSSRMSQVVSACDILVVAVTAGVGVYGAPARMRTMLSYMENIVAVDRELGRHHSAATERKLCALLLLILLSFTILLVDDFCFYAMQAGKTGRQWEIVTNYAGFYFLWYIVMVLELQFAFTALSLRARLKLFNEALNVTASQVCKPVKKPKNSQLSVYATSVRPVSCKRENVIVETIRVRDKDDAFVMMKTADGVPCLQVPPCEAVGRLSRMRCTLCEVTRHIADGYGLPLVIILMSTLLHLIVTPYFLIMEIIVSTHRLHFLVLQFLWCTTHLIRMLVVVEPCHYTIREGKRTEDILCRLMTLAPHGGVLSSRLEVLSRLLMLQNISYSPLGMCTLDRPLMVTVLGAVTTYLVILIQFQRYDS

>BmorGR13

MEDSFNRLLSIRNMIIFQNVCGFYHMCTEKLYISRIIKMYCVALAIVLSVFCFQNPDITYLSWDVVWVTFGYTLNVIICLRYNGNYFFQYWNGLHEIDIKMNLTSIDKEKVPISRAVFTVFLILRSTAFAMTIFVFGYLETGILSNTIISIYSINLTEFYRNMSNIPMILMFETFYVRIKILKEQLCSELSTVLGCNNDARQLKLILKYLRNYRSLVRHLMDTTLPFKILILVILVGSFLRSLLIGYAFVYNSDQIILLSLPVMFSTKILSEVVEIKLICTKELLKNKNEGLVLLDLDSKKPTFLTSKACGEQLQDALSFLNNRSYSYTLLQVIEFDCSLAFVFTSFCITHLIVVVQFTHVLD

>BmorGR14

MNLHKNIIPIRNNLFANKVTAIALPKTLSVLFKLIHIFFLLDLGVYEYKTFKIKCIVKFLTISGSLTISVVCFSFMVSNLSEHTFVGWYGFFISTYIFVVLFFNLSNRMTFVEFYKTLLRFDANYGIDSNEYKFNFKIIFVNILFIANRMVLSFVYCSYYPQNCIRPRYAQILFMLPWLTLDVLLTTNMFLFYATYCRIAKFPMLIKNSMNIVALRNSYKLIVDSLEKTQTSFDIVFIIALVFSVPEIMMSIYSTLLEVISKHFLEVASILSLNYVAIAQSLLLTLAPSLCAGVLPWKTNNIKIILHEKLFTEKDKASAREIELFIKYIESRPLKLRACNLVPLDFSLTIIVLNICVTYLIVIIQFTHLY

>BmorGR15

MISSSDINHKRNKVFAYNVPGIALSKTLTVLFKLLHYVLLLDVGIYEYKTFKNKCIVKFLTIATGVSVSIVYFCLIATVLRKNAFFYWFYVLFISQYMIIVFIFTLSNGMSFTDYYKMLLRFDAKYQINSNNYYFNIKIILVIIISILNRIGMAIIYCSYYTKNCYEMSFSQIIFVLPWLTRDVILIMNVFLFYVTYCRITKFPALLENTKNVGSLRNSYKLIVDSLEKTQKPFDFVFTISLVFNIPEIMLSIYFTLLQVIHSHFLEVAPTLSISYFSITHSVVLILAPSLCAGVLPWKTNTIKIVLHDKLFLEKDKNSARNIKLFIKYIEARPLKLRACNLVPLDFSLPVIVLNLCVTYLIVIVQFSHLS

>BmorGR16

MIMNLTTDRISKRNKVFAYNVPEVTLPTTLKVLFKLIQFTLSLDFGVYKYKTFKMKCVAKVLTLAGCLAASAACVSLIISNIFENQLFFGWYTLFVCQYTIVIFMFTFSNGMTFIDYKMMLLRFDAKYQIDSNVYHFNIKIVLVVVISVTSRLFLCAVYCIYSTENCIKPWYNQLLFFPWLSLDIVLIMNMFLFYATYCRLAKFPSLFENPKNVVPLRNSYKLIVDSLEKTKKSFDAVLIAALIFNIPEIMMSIYYTLFQVMNKHFQEVAPVLSLSYFTIILSVLLILAPSLCAGVLPWKTRHMRLILLEKLFAEKDKNSAREIELFIKYIEARPLQLRACNLVPLDFNLPVIVLNLCITYLIVIIQFTHLF

>BmorGR17

MGFSLGTTALSMFFFEKPVVFTIIQITMIIVKPAKYKLSDPFRPKDTSKLSESIIMYFKLFHIFLGIDLGGFRYQNRQVKYAVRLISLIQPLAIYGLCIYALLKIIANTEFLWYTISFTEYVAMSVAITLFSNEMTYCNFMINLKFIDTKLKIGDESFRIGVKLISSTILIGVTRCFTTTTYCLLGFCAKPTAAQILFQIPWLTIDLMLLQYMFIFYACYCRLVKILRILKKRNTDIEEMRRIYKTLVDVLDRARAPFDLAYLLGLLFSIPDVLYSIYESIIKVGEINTAKALSMSIIYITNIQSLALMFAPALTAGFLPSLTMKMRIILHDKLLEEQDKKTYRHIVLFIKYIETCPLKLKACQIIPLDFSFPIIILNIVVTYLIVAIQLTHFL

>BmorGR18

MRRSTKVISMVNQSDKGEIKTCSRFMKIYFFVIYILTGFNFGFYTGRGLNFLRVIQASVLLLRFIIASNCIYIAFHFRLLEAIWYSLTFSESLAIVVCFMLSRSALSCKNLFEYLYSVDQELKKSVGPSIEVKLALYTVVVSVLRLTVYVFCAIAYYETLHEGFCVELVYNTPCYCSDLYLVIHFTIFHSVYCRLKALRISMNEKFDVYKGTLIYKSLIDNLEEIKKSLDVPFFVILLNAVAIAMINILVTLEISYGQTMKFIRTAPRYLETVLLFSSAFAPVLAADMMASEAQKIKVTLNNILQRDDSLLEDDRRKVKQFAGYVSARPFRLRACRVLSLDCTLPVTVLSICVTYLIVVVQFTHLY

>BmorGR26

MNKTKIYRKKLDKNERLVCSVQPAMFARLIVGLYYDIKVSNRVKWMIKSYCISLSSFICYLIIFRDDNFSLHPKLTSVMEYITYVTFSFLTCDKYLFRYLRFNPRTDGYPIFLYLCKKFEKFFKIIICLFVSFKILGVVLMMQSWPILSTPKYIWGTLALHFLWLASHMGRLVFILVYGILFCRMRTIRIIFENRGFQNTPQNRLTPKRYILMYEAVLNSIESVDFPVKFLIFTFICCFAPKLVVSLFEIMEEMKKGELSLTTFIWFLVELSPSYLFLLLSAIALDLVSEDVQELLSITIDRRLNCKNEKERSEIQEFFQYLRNNPFNYTLWQVVSLNLRTLLVATSFSIANVIAIMQIKNSKI

>BmorGR27

MVFKYKIMTKAPKSLPVLKILMLFRLVFGNYFRLSSNRYINFLVKSYCSTFTILLSVMCGKRLKNDSPYMLSLTEYILNKILNYATSEGYIFKYCNSIKTCDKIMGFKKLPIITIDVFIAIIITVITRTAITIYFGFLFPFDKYQVVLYVGCIVFSNDLNSLTIMNVFGLLNNRMNLLRKSLEAMTVPINIIGKNEVAPKVRLVRNAFRYYSNLLDNLDSVNHCVQYSLSVTLLLKFPKAVLLCYDSIKTYFVKIDNNFAMDIVDPTEIILSIVVMSFPAMLCEMITNEVEKIKAILTKHLIQCSDNSLRFELNITLLYICHRPFKYILWRAIPLDTSVPIGIVSLIITYVIVLIQLLHFST

>BmorGR29

MYLRSKKSRFKLFSFERMIKILLMICGHYVQTDSSNVVSSIHRIFSIVITICLCPYFQFNPFFFHVIESVWYSILSQFTQYGFFFRYCSTIKTFDLLSGFKQIPLYTKRVCFFLLITLLVRLIIVLIHFSAHQTKLKTFCAFLIILSANTGHILMTIMFSILNTRMTLIQKLFANNPIPVNIVGKNQNASHIKRVRKGLICYNNLLDTLKVAEKEIQFTLTVTYLCHVPTIICYVYFVITVIYKSKFSGYNLIPMLDMILACMAVTAPALFAELTKNTVDKIKKILGSQLLRCSDESLRYELEITLEYVIQRPFSFSIWRAVSLDASLPVAMTSLCITYVIVILQLTQLRP

>BmorGR30

MYLRSKKSRFKLFSFERMIKILLMICGHYVQTDSSNVVSSIHRIFSIVITICLCPYFQFNPFFFHVIESVLYSILSQFTQYGFFFRYCSTIKTFDLLSGFKQIPLYTKRVCFFLLITLLVRLIIVLIHFSAHQTKLKTFCAFLIILSANTGHILMTIMFSILNTRMTLIQKLFANNPIPVNIVGKNQNASHIKRVRKGLICYNNLLDTLKVAEKEIQFTLTVTYLCHVPKIICYVYFVITVIYKSKFSGYNLVPLFDMILACMAVTAPAVFAELTKNTVDKIKKILGSQLLRCSDESLRYELEITLEYVIQRPFSFSIWRAVSLDASLPVAMTSLCITYVIVILQLTQLRP

>BmorGR45

MKSPEYLSKDILDEDFVRVFSFPFLVQMALGSCRVHLKARFITVPTLGQKLYTVMCIIICSLMYFNMTKLYLPLYYEHSIVYYIFVTVTGLDQLSFFANLIHLRFLNGETNTAFYIMMQRIDRNMKIDHNNIFNKTVTLANILTITLIILHYVGLVISTIILKEYSLLSLFGLLYGQLMLMVEMALCSNLIIFFFMRVRFVNAIIKNHVHPENQNQPPKLVRYFITNRITRYLAAQTHDFIVNDTDVYLKQIFEGFSMFIDIYRFQVCPLCIKLVVLTLLNFEFCLVAIQRNVLGPNHIGNYYIIVNSVMGFFTALYVSGRCELFFREIRETKRLSVAVLLQYQEGPLREKATRMLKIIEESTPQFSIYDMWQMDGYTFVKICSLVTNLIVTLLQFAYL

>BmorGR50

MAGIRTISSKVKPLELPDVSENNFADDGLKIVQPFKFFIYIQAITGINRLYLLKCNKFVLMFSYLYAIFLISFVALVYWTTEPKKNSHLVIRLFTFFEYTLLACISVFLKKKKMIKFFENLSLLDKMLKINKNVNSTCCMKQVFFWVTGSIVYNLIEFYAMEFYDNTNKGLKTIICTYAIALAHDCEQIFFFTLQRVVYLRLLVVKRHIQEYFKVDEDSSRKKPNKYEMLSNNVQLNLTALHEVYALLHNCAEKLNTVMSIPVLLMLFTSGLSTTILLKFFVRVIQLTDPSNPGSAIGVCMYLIVRCIKYTLLVVISCYYSSITATQVSLIRITIHDAINTVPLGKLQRRKVKAFYLMTKEYSFVYALAGVIKLNMSLPLSYISLCTTYLVIIIQFSKFLD

>BmorGR51

MAMGIRTILSKVKPLELPDVSENNFADDGLKIVQRFKFFIYIQVLTGINRLYLLKCNKFVMLFSYLYAIFLISFVASVYWTKEPMKNSHLVIRLFSFIEYILLICISVFLKKKKMMKFFENLSMFDQILKIDKNVNSTFCMKRVFFWVTGSIVYNLIEFYALEFYDNTSKGLMTIICTYTIALTHDCEQIFFFTLQRVVYLRLLVVKRHIQEHFKVDEDSNRKKPNKYEMLSKNVQLNLTALHEVYGLLHNCAEKLNKIMSIPVLLMLFTSGLTTTILLRILVRVIQLADPSNPGSAIGLCVYLIVRCIKYTLLVVISCYYSSITATQVSLIRITINDAINTIAFGKLQRRKVKAFYLMTKEYSFVYTLAGVIKLNMSLPLSYISLCTTYLVIIIQFSKFFD

>BmorGR53

MAHIKDENQSKQQQKEHETLNKNKLKKVVYTLKPALMLENWFGLSDFLLVNEDELVLLMQTEKFGVILSIFFIVMFAVFVDFPDTETESIMELMDEVPSMVVLSQYFIASITTSSCLSAIAIRIFETFADLDSMLLITTTQDFYNKSRYQTNKYLIILGVSHIISSTLDLLTDDEIVWCKFFVLPIYFLQKLEVLTFCKLIVMIQCRLQIINKYLTNFIEEQEKNKALVFTLAESNPKKTDKFNWIGCPSPNNMKIRDLATMYDVIGTICSLINDLFNIQIFMTLVSTFTYIVIAIWSTLYFYRAPNFTFGTLTTIIIWCITIILSVVVMSFVCERLVSVRNNTKILVNKVIMNYDLPKTMRVQAKAFMELIESWPLKIMVYDMFSVDISLMLKFISVATTYLIVIIQLSHFV

>BmorGR63

MQIGNAVIHLKSTKLTTMNTISPTTKLLKIFALNSNIEEIDLKCSTKLRITMTAFVLCSLIFYSLYYKFIYVFDYVNISIKITDCVQMVYDFCQYIVDLYFVTNYGRNISSEYFQQYKIIDKILEVVCYEIIKHRIVKLLWVFMCIWFSSSCFDFIAWFLNYGWITPLVYSVAYIFLLIKILTTLDLSAHIMNVEIRLKMIADLIHHYYMSCEDNFQAEETLCHKNWLNSKERAKYYELQFRIHALKQLSCNNNEIKLLSRCYLMLTEQVEIINRMYGFRILLNSLSLLIDMVRFTNISVRIMIGSQNLAYNCGYFPAVSSIFRLLTCGAVIINLVSHCERVYYQRTRICNVIDHMIVNKNLSRESTEALQEFRNLVQNHPIEFNMANFFQLNYSLLVSIASVVVTYTIILLQSVN

>HassGR1

MGQTSFRRNMSFWIPVKKNKVDVAKPKVKNITSFQDALRATLIIGQVFSLLPFVGVFTNVASNVKFVKTSWKCVYSLLSLIGQMFMAVLCINKLAKTTVSLNGTSPVIFYVTTCVTMMLFFQVARRWPALVQHISKAEDMDPNFDCSLTRKCNITCAVVLILALCEHILSLLSAFAGASACYSGMDTYEGFVTHFYPWVFSYLPYSIVLGVITQFLHFQSTFIWNFSDLFVICMSYYLTSRLEQVNRKLLAAQGKYLPEIFWRATREDYCRATQIVRKVDEVISGVVFISFANNLFFICLQLFNTLEDGLKGTGECTQKLKKIVVSKSGPLGGYEAAAYFLFSLVYLLSRSVAVSLIASQVNSASSVPAPVLYDVPSPVYCVEVQRFLDQVNGDKVALSGLQFFSVTRGLLLTVAGTIVTYELVMFQFNSSTPSLNMTSPTVVTHTVTTLAT

>HassGR3

MTVPIPNGFPVQINSKPKNKIIFLDVTPVSTPIKPHSPNVVAPMRNNLVAPHISNDIIYENIKPVFTLLRIMGVLPITRPSACVNQFQIASSSMLYAILVFISLVSYVLYLSLHKVQILRTAEGKFEEAVIEYLFTVYLFPMIAVPLLWYETRKIANVLNGWVDFEMVYKQLSGRTLPVKLYKKALAMAVIIPILSTTTVIVTHVTMVHFKPMQLVPYVFLEILTYMLGGYWYLLCETLSICANILAEDFQNALRHIGPAGKVAEYRALWLRLSKLSRDTGIANCYTFTFVNLYLFLIITLSIYGLLSQISEGFGIKDIGLALTAFCSISLLFFICDEAHYASHNVRTNFQKKLLMVELSWMNTDAQTEVNMFLRATEMNPSQISLGGFFNVNRTLFKSLLATMVTYLVVLLQFQISIPDESQNRDEEEEVPYNITSATTEAMTTSTTTIMTTVLTTLAKKKKKN

>HassGR4

MLWIQTHHYIGVESAKVEEVTAAPVPSESGSRPSRPTHCVVGGAHVFILRISSFFGLAPLRFESRSNGFTVTISGAMCVYSYILVTVLVICTIFGLVAEINVGVELSVRMSSRMSQVVSTCDVLVVVATAGAGVYGAPRRMRNMLKFMENIASVDTSIGGQYSLVTERKLCGIILAILIFFSILIADDFTFYALQAKKLDREWDVVTNYLGFYLLWFVVLILELQFAFTALSVRARFSAVNDALALTARQVSIPVEKPKSSSPLNIYAIRVAPVDSQRSANVSLLVDTVTGREHVVIIKRTASGEPRLVVSPCDAVRRLAAPHGTLCDVVNSIDDSHGLPLVVILISTLLHLIVTPYFLIMEIIVSTNRIHFLVLQFLWCVTHMLRMIVVVEPRHYTIAEGERTEGLVCRLMTSVPSTGVLPSRLEIFSRQLLLQSVSYAPVGMCTLHRPLIASVIGAVTTYLVILIQFQRYDN

>HassGR5

MSSKEFKQFMQQKKLLLPQQPIHDDFLDVIEKVFHWSCFYGVFVSKRFISLIWSTLILGSLVIIEALAIWKVIRVLAGVARDMSGHRSVTARLAGTIFYSISILSLVLISKLYYNWRTDIAGVWGKVERSVGVKIPVDKTLKCRMSFVAGLMTFCSFFEHALSILASVGFDCPPSLILKRYVLVSHGFIFMGQDYSEWFAMPLVIISTIATLLWNFQDQLIVLISMGLTSRYRRLNECLAKFCELEKQHMNSDQKVGAVKVYTWRKIREAYVKQAMLVRKIDVALGGIIILSCSCNFYFICLQMFLGITQGMSTDFLTGVYYVVSLAWLCIRVLSVVLAASGVNTHSKLALNHLYTYETHCYNVEVERLQDRLTKDYIALSGMGFFYLNKTILLQMAGAIITYELVLIQFDDQGSDGIALNATNI

>HarmGR1

MGQTSFRRNMSFWIPVKKNKVDVAKPKVKNITSFQDALRVTVIIGQVFSLLPFVGVFTNVASNVKFVKTSWKCVYSLLSLIGQMFMAVLCINKLAKTTVSLNGTSPVIFYVTTCVTMMLFFQVARRWPALVQHISKAEDMDPNFDCSLTRKCNITCAVVLILALCEHILSLLSAFAGASACYSGMDTYEGFVTHFYPWVFSYLPYSIVLGVITQFLHFQSTFIWNFSDLFVICMSYYLTSRLEQVNRKLLAAQGKYLPEIFWRATREDYCRATQIVRKVDEVISGVVFISFANNLFFICLQLFNTLEDGLKGTGECTPKLKKIVVSKSGPLGGHEAAAYFLFSLVYLLSRSVAVSLIASQVNSASSVPAPVLYDVPSPVYCVEVQRFLDQVNGDKVALSGLQFFSVTRGLLLTVAGTIVTYELVMFQFNSSTPTLNITSPTVVTHTITTLAT

>HarmGR4

MLWIETHHYLGVESAKVEEVTAAPVPSESGSRPSRPTHCVVGGTHAFILRISSFFGLAPLRFESRSNGFTVSISGAMCVYSYILVTVLVICTIFGLVAEINVGVELSVRMSSRTSQVVSTCDVLVVVATAGAGVYGAPRRMRNMLKFMENIASVDTSIGGQYSLVTERKLCGIILAILIFFSILIADDFTFYALQAKKLDREWDVVTNYLGFYLLWFVVLILELQFAFTALSVRARFSAVNDALALTARQVSIPVEKPKSSSPLNIYAIRVAPVDSQHSANVSLLVDTMTGREHVVIIKRTASGEPRLVVSPCDAVRRLAALHGTLCDVVNSIDDSYGLPLVVILISTLLHLIVTPYFLIMEIIVSTNRIHFLVLQFLWCVTHMLRMIVVVEPGHYTIAEGKRTEGLVCRLMTSAPSTGVLPSRLEIFSRQLMLQSVSYAPMGMCTLHRPLIASVIGAVTTYLVILIQFQRYDN

>HarmGR5

MSSKEFKQFLRQNKLLLPQQPIHDDFLGVIEKVFHWSCFYGVFGSKRFISLIWSTLILGSLVIIEVLAIWKVIRALAGVARDMSGHRSVTARLAGTIFYSISILSLVLISKLYYNWRINIAGVWAKVERSVGVKIPVDKTLKCRMSFVAGLMTFCSFFEHALSILASVGFDCPPSLILKRYVLVSHGFIFMGQDYSEWFAMPLVVISTIATLLWNFQDQLIVLISMGLTSRYRRLNECLAKFCELEKQHMDSDKKVEAVKVYTWRKMREAYVKQAMLVRKIDVALGGIIILSCSCNFYFICLQMFLGITQGMSTDFLTGLYYMVSLAWLCIRVLSVVLAASGVNTHSKLALNHLYTYETHCYNVEVERLQDQLTKDYIALSGMGFFYLNKTILLQMAGAIITYELVLIQFDDQGSDGIALNATNI

>DmelGR21a

MTFLDRTMSFWAVSRGLTPPSKVVPMLNPNQRQFLEDEVRYREKLKLMARGDAMEEVYVRKQETVDDPLE

LDKHDSFYQTTKSLLVLFQIMGVMPIHRNPPEKNLPRTGYSWGSKQVMWAIFIYSCQTTIVVLVLRERVKKFVTSPDKRFDEAIYNVIFISLLFTNFLLPVASWRHGPQVAIFKNMWTNYQYKFFKTTGSPIVFPNLYPLTWSLCVFSWLLSIAINLSQYFLQPDFRLWYTFAYYPIIAMLNCFCSLWYINCNAFGTASRALSDALQTTIRGEKPAQKLTEYRHLWVDLSHMMQQLGRAYSNMYGMYCLVIFFTTIIATYGSISEIIDHGATYKEVGLFVIVFYCMGLLYIICNEAHYASRKVGLDFQTKLLNINLTAVDAATQKEVEMLLVAINKNPPIMNLDGYANINRELITTNISFMATYLVVLLQFKITEQRRIGQQQA

>DmelGR10a

MTSPDERKSFWERHEFKFYRYGHVYALIYGQVVIDYVPQRALKRGVKVLLIAYGHLFSMLLIVVLPGYFCYHFRTLTDTLDRRLQLLFYVSFTNTAIKYATVIVTYVANTVHFEAINQRCTMQRTHLEFEFKNAPQEPKRPFEFFMYFKFCLINLMMMIQVCGIFAQYGEVGKGSVSQVRVHFAIYAFVLWNYTENMADYCYFINGSVLKYYRQFNLQLGSLRDEMDGLRPGGMLLHHCCELSDRLEELRRRCREIHDLQRESFRMHQFQLIGLMLSTLINNLTNFYTLFHMLAKQSLEEVSYPVVVGSVYATGFYIDTYIVALINEHIKLELEAVALTMRRFAEPREMDERLTREIEHLSLELLNYQPPMLCGLLHLDRRLVYLIAVTAFSYFITLVQFDLYLRKKS

>DmelGR63a

MRPSGEKVVKGHGQGNSGHSLSGMANYYRRKKGDAVFLNAKPLNSANAQAYLYGVRKYSIGLAERLDADYEAPPLDRKKSSDSTASNNPEFKPSVFYRNIDPINWFLRIIGVLPIVRHGPARAKFEMNSASFIYSVVFFVLLACYVGYVANNRIHIVRSLSGPFEEAVIAYLFLVNILPIMIIPILWYEARKIAKLFNDWDDFEVLYYQISGHSLPLKLRQKAVYIAIVLPILSVLSVVITHVTMSDLNINQVVPYCILDNLTAMLGAWWFLICEAMSITAHLLAERFQKALKHIGPAAMVADYRVLWLRLSKLTRDTGNALCYTFVFMSLYLFFIITLSIYGLMSQLSEGFGIKDIGLTITALWNIGLLFYICDEAHYASVNVRTNFQKKLLMVELNWMNSDAQTEINMFLRATEMNPSTINCGGFFDVNRTLFKGLLTTMVTYLVVLLQFQISIPTDKGDSEGANNITVVDFVMDSLDNDMSLMGASTLSTTTVGTTLPPPIMKLKGRKG

>DmelGR8a

MSGHLGRVLQFHLRLYQVLGFHGLPLPGDGNPARTRRRLMAWSLFLLISLSALVLACLFSGEEFLYRGDMFGCANDALKYVFAELGVLAIYLETLSSQRHLANFWWLHFKLGGQKTGLVSLRSEFQQFCRYLIFLYAMMAAEVAIHLGLWQFQALTQHMLLFWSTYEPLVWLTYLRNLQFVLHLELLREQLTGLEREMGLLAEYSRFASETGRSFPGFESFLRRRLVQKQRIYSHVYDMLKCFQGAFNFSILAVLLTINIRIAVDCYFMYYSIYNNVINNDYYLIVPALLEIPAFIYASQSCMVVVPRIAHQLHNIVTDSGCCSCPDLSLQIQNFSLQLLHQPIRIDCLGLTILDCSLLTRMACSVGTYMIYSIQFIPKFSNTYM

>DmelGR64d

MLRSHLSVHGLQMERSVQENTLHYTIGHVLIIARIFGVLPLAGINPNGKPENVRFRWFSPYILFFVVAFTFVIADFMLSTKIVLNDGLQLYTMGSLSFSVICIFCFGSFIKLSRRWPHIIRETALCERIFLKPCYANQEGLNFTRFLRRWALILLVAALCEHLTYVGSAAWSNYVQIRDCNLKVGFVENYFLRERQELFSVFEYRAWMVFFIEWNTMAMTFVWNFGDIFLFLMCRGLKIRFQQLHWRIRQNLGKPMAKEFWQEIRSDFLDLDSLLKLYDKELSGLILVCCAHNMYFICVQVYHSFQVKGAFMDELYFWFCLLYVISRLMNMMLAASSIPQEIKDISNTLYEVRSSPWCDELGRLSEMLRNETFALSGMGYFYVTRRLIFAMAGALMGYELVLFRQMQGAVVQKSICSRGPGSSMSIFFS

>DmelGR64b

MPQGETFHRAVSNVLFISQIYGLLPVSNVRALDVADIRFRWCSPRILYSLLIGILNLSEFGAVINYVIKVTINFHTSSTLSLYIVCLLEHLFFWRLAIQWPRIMRTWHGVEQLFLRVPYRFYGEYRIKRRIYIVFTIVMSSALVEHCLLLGNSFHLSNMERTQCKINVTYFESIYKWERPHLYMILPYHFWMLPILEWVNQTIAYPRSFTDCFIMCIGIGLAARFHQLYRRIAAVHRKVMPAVFWTEVREHYLALKRLVHLLDAAIAPLVLLAFGNNMSFICFQLFNSFKNIGVDFLVMLAFWYSLGFAVVRTLLTIFVASSINDYERKIVTALRDVPSRAWSIEVQRFSEQLGNDTTALSGSGFFYLTRSLVLAMGTTIITYELMISDVINQGSIRQKTQYCREY

>DmelGR43a

MEISQPSIGIFYISKVLALAPYATVRNSKGRVEIGRSWLFTVYSATLTVVMVFLTYRGLLFDANSEIPVRMKSATSKVVTALDVSVVVMAIVSGVYCGLFSLNDTLELNDRLNKIDNTLNAYNNFRRDRWRALGMAAVSLLAISILVGLDVGTWMRIAQDMNIAQSDTELNVHWYIPFYSLYFILTGLQVNIANTAYGLGRRFGRLNRMLSSSFLAENNATSAIKPQKVSTVKNVSVNRPAMPSALHASLTKLNGETLPSEAAAKNKGLLLKSLADSHESLGKCVHLLSNSFGIAVLFILVSCLLHLVATAYFLFLELLSKRDNGYLWVQMLWICFHFLRLLMVVEPCHLAARESRKTIQIVCEIERKVHEPILAEAVKKFWQQLLVVDADFSACGLCRVNRTILTSFASAIATYLVILIQFQRTNG

>DmelGR64c

MQQSGQKGTRNTLQHAIGPVLVIAQFFGVLPVAGVWPSCRPERVRFRWISLSLLAALILFVFSIVDCALSSKVVFDHGLKIYTIGSLSFSVICIFCFGVFLLLSRRWPYIIRRTAECEQIFLEPEYDCSYGRGYSSRLRLWGVCMLVAALCEHSTYVGSALYNNHLAIVECKLDANFWQNYFQRERQQLFLIMHFTAWWIPFIEWTTLSMTFVWNFVDIFLILICRGMQMRFQQMHWRIRQHVRQQMPNEFWQRIRCDLLDLSDLLGIYDKELSGLIVLSCAHNMYFVCVQIYHSFQSKGNYADELYFWFCLSYVIIRVLNMMFAASSIPQEAKEISYTLYEIPTEFWCVELRRLNEIFLSDHFALSGKGYFLLTRRLIFAMAATLMVYELVLINQMAGSEVQKSFCEGGVGSSKSIFS

>DmelGR64e

MARTTGDPAKRRRCMSRIKFWRRSRVGSEVVEKDTKRFKLSLIKAWLLRIRQEDYKYSGSFQEAIKPVLIIAQIFALMPVRKVSSKFAEDLTFTWFSVRSYYALVTILFFGVSSGYMVAFVTSVSFNFDSVETLVFYLSIFLISLSFFQLARKWPEIAQSWQLVEAKLPPLKLPKERRSLAQHINMITIVATTCSLVEHIMSMLSMGYYVNSCPRWPDRPIDSFLYLSFSSVFYFVDYTRFLGIVGKVVNVLSTFAWNFNDIFVMAVSVALAARFRQLNDYMMREARLPTTVDYWMQCRINFRNLCKLCEEVDDAISTITLLCFSNNLYFICGKILKSMQAKPSIWHALYFWFSLVYLLGRTLILSLYSSSINDESKRPLVIFRLVPREYWCDELKRFSEEVQMDNVALTGMKFFRLTRGVVISVAGTIVTYELILLQFNGEEKVPGCFEN

>DmelGR93a

MFSSSSAMTGKRAESWSRLLLLWLYRCARGLLVLSSSLDRDKLQLKATKQGSRNRFLHILWRCIVVMIYAGLWPMLTSAVIGKRLESYADVLALAQSMSVSILAVISFVIQARGENQFREVLNRYLALYQRICLTTRLRHLFPTKFVVFFLLKLFFTLCGCFHEIIPLFENSHFDDISQMVGTGFGIYMWLGTLCVLDACFLGFLVSGILYEHMANNIIAMLKRMEPIESQDERYRMTKYRRMQLLCDFADELDECAAIYSELYHVTNSFRRILQWQILFYIYLNFINICLMLYQYILHFLNDDEVVFVSIVMAFVKLANLVLLMMCADYTVRQSEVPKKLPLDIVCSDMDERWDKSVETFLGQLQTQRLEIKVLGFFHLNNEFILLILSAIISYLFILIQFGITGGFEASEDIKNRFD

>DmelGR61a

MSRTSDDIRKHLKVRRQKQRAILAMRWRCAQGGLEFEQLDTFYGAIRPYLCVAQFFGIMPLSNIRSRDPQDVKFKVRSIGLAVTGLFLLLGGMKTLVGANILFTEGLNAKNIVGLVFLIVGMVNWLNFVGFARSWSHIMLPWSSVDILMLFPPYKRGKRSLRSKVNVLALSVVVLAVGDHMLYYASGYCSYSMHILQCHTNHSRITFGLYLEKEFSDIMFIMPFNIFSMCYGFWLNGAFTFLWNFMDIFIVMTSIGLAQRFQQFAARVGALEGRHVPEALWYDIRRDHIRLCELASLVEASMSNIVFVSCANNVYVICNQALAIFTKLRHPINYVYFWYSLIFLLARTSLVFMTASKIHDASLLPLRSLYLVPSDGWTQEVQRFADQLTSEFVGLSGYRLFCLTRKSLFGMLATLVTYEL

MLLQIDAKSHKGLRCA

>DmelGR33a

MIQIMNWFSMVIGLIPLNRQQSETNFILDYAMMCIVPIFYVACYLLINLSHIIGLCLLDSCNSVCKLSSHLFMHLGAFLYLTITLLSLYRRKEFFQQFDARLNDIDAVIQKCQRVAEMDKVKVTAVKHSVAYHFTWLFLFCVFTFALYYDVRSLYLTFGNLAFIPFMVSSFPYLAGSIIQGEFIYHVSVISQRFEQINMLLEKINQEARHRHAPLTVFDIESEGKKERKTVTPITVMDGRTTTGFGNENKFAGEMKRQEGQQKNDDDDLDTSNDEDEDDFDYDNATIAENTGNTSEANLPDLFKLHDKILALSVITNGEFGPQCVPYMAACFVVSIFGIFLETKVNFIVGGKSRLLDYMTYLYVIWSFTTMMVAYIVLRLCCNANNHSKQSAMIVHEIMQKKPAFMLSNDLFYNKMKSFTLQFLHWEGFFQFNGVGLFALDYTFIFSTVSAATSYLIVLLQFDMTAILRNEGLMS

>DmelGR32a

MSPNTWVIEMPTQKTRSHPYPRRISPYRPPVLNRDAFSRDAPPMPARNHDHPVFEDIRTILSVLKASGLMPIYEQVSDYEVGPPTKTNEFYSFFVRGVVHALTIFNVYSLFTPISAQLFFSYRETDNVNQWIELLLCILTYTLTVFVCAHNTTSMLRIMNEILQLDEEVRRQFGANLSQNFGFLVKFLVGITACQAYIIVLKIYAVQGEITPTSYILLAFYGIQNGLTATYIVFASALLRIVYIRFHFINQLLNGYTYGQQHRRKEGGARARRQRGDVNPNVNPALMEHFPEDSLFIYRMHNKLLRIYKGINDCCNLILVSFLGYSFYTVTTNCYNLFVQITGKGMVSPNILQWCFAWLCLHVSLLALLSRSCGLTTTEANATSQILARVYAKSKEYQNIIDKFLTKSIKQEVQFTAYGFFAIDNSTLFKIFSAVTTYLVILIQFKQLEDSKVEDPVPEQT

>DmelGR64f

MKILPKLERKLRRLKKRVTRTSLFRKLDLVHESARKKAFQESCETYKNQIENEYEIRNSLPKLSRSDKEAFLSDGSFHQAVGRVLLVAEFFAMMPVKGVTGKHPSDLSFSWRNIRTCFSLLFIASSLANFGLSLFKVLNNPISFNSIKPIIFRGSVLLVLIVALNLARQWPQLMMYWHTVEKDLPQYKTQLTKWKMGHTISMVMLLGMMLSFAEHILSMVSAINYASFCNRTADPIQNYFLRTNDEIFFVTSYSTTLALWGKFQNVFSTFIWNYMDLFVMIVSIGLASKFRQLNDDLRNFKGMNMAPSYWSERRIQYRNICILCDKMDDAISLITMVSFSNNLYFICVQLLRSLNTMPSVAHAVYFYFSLIFLIGRTLAVSLYSSSVHDESRLTLRYLRCVPKESWCPEVKRFTEEVISDEVALTGMKFFHLTRKLVLSVAGTIVTYELVLIQFHEDNDLWDCDQSYYS

>DmelGR64a

MKGPNLNFRKTPSKDNGVKQVESLARPETPPPKFVEDSNLEFNVLASEKLPNYTNLDLFHRAVFPFMFLAQCVAIMPLVGIRESNPRRVRFAYKSIPMFVTLIFMIATSILFLSMFTHLLKIGITAKNFVGLVFFGCVLSAYVVFIRLAKKWPAVVRIWTRTEIPFTKPPYEIPKRNLSRRVQLAALAIIGLSLGEHALYQVSAILSYTRRIQMCANITTVPSFNNYMQTNYDYVFQLLPYSPIIAVLILLINGACTFVWNYMDLFIMMISKGLSYRFEQITTRIRKLEHEEVCESVFIQIREHYVKMCELLEFVDSAMSSLILLSCVNNLYFVCYQLLNVFNKLRWPINYIYFWYSLLYLIGRTAFVFLTAADINEESKRGLGVLRRVSSRSWCVEVERLIFQMTTQTVALSGKKFYFLTRRLLFGMAGTIVTYELVLLQFDEPNRRKGLQPLCA

>DmelGR66a

MAQAEDAVQPLLQQFQQLFFISKIAGILPQDLEKFRSRNLLEKSRNGMIYMLSTLILYVVLYNILIYSFGEEDRSLKASQSTLTFVIGLFLTYIGLIMMVSDQLTALRNQGRIGELYERIRLVDERLYKEGCVMDNSTIGRRIRIMLIMTVIFELSILVSTYVKLVDYSQWMSLLWIVSAIPTFINTLDKIWFAVSLYALKERFEAINATLEELVDTHEKHKLWLRGNQEVPPPLDSSQPPQYDSNLEYLYKELGGMDIGSIGKSSVSGSGKNKVAPVAHSMNSFGEAIDAASRKPPPPPLATNMVHESELGNAAKVEEKLNNLCQVHDEICEIGKALNELWSYPILSLMAYGFLIFTAQLYFLYCATQYQSIPSLFRSAKNPFITVIVLSYTSGKCVYLIYLSWKTSQASKRTGISLHKCGVVADDNLLYEIVNHLSLKLLNHSVDFSACGFFTLDMETLYGVSGGITSYLIILIQFNLAAQQAKEAIQTFNSLNDTAGLVGAATDMDNISSTLRDFVTTTMTPAV

>DmelGR5a

MRQLKGRNRCNRAVRHLKIQGKMWLKNLKSGLEQIRESQVRGTRKNFLHDGSFHEAVAPVLAVAQCFCLMPVCGISAPTYRGLSFNRRSWRFWYSSLYLCSTSVDLAFSIRRVAHSVLDVRSVEPIVFHVSILIASWQFLNLAQLWPGLMRHWAAVERRLPGYTCCLQRARPARRLKLVAFVLLVVSLMEHLLSIISVVYYDFCPRRSDPVESYLLGASAQLFEVFPYSNWLAWLGKIQNVLLTFGWSYMDIFLMMLGMGLSEMLARLNRSLEQQVRQPMPEAYWTWSRTLYRSIVELIREVDDAVSGIMLISFGSNLYFICLQLLKSINTMPSSAHAVYFYFSLLFLLSRSTAVLLFVSAINDQAREPLRLLRLVPLKGYHPEVFRFAAELASDQVALTGLKFFNVTRKLFLAMAGTVATYELVLIQFHEDKKTWDCSPFNLD

>DmelGR39aIA

AMSKVCRDLRIYLRLLHIMGMMCWHFDSDHCQLVATSGSERYAVVYAGCILVSTTAGFIFALLHPSRFHIAIYNQTGNFYEAVIFRSTCVVLFLVYVILYAWRHRYRDLVQHILRLNRRCASSCTNQQFLHNIILYGMLTILCFGNYLHGYTRAGLATLPLALCMLVYIFAFLVLCLLLMFFVSLKQVMTAGLIHYNQQLCQGDLISGLRGRQQILKLCGGELNECFGLLMLPIVALVLLMAPSGPFFLISTVLEGKFRPDECLIMLLTSSTWDTPWMIMLVLMLRTNGISEEANKTAKMLTKVPRTGTGLDRMIEKFLLKNLRQKPILTAYGFFALDKSTLFKLFTAIFTYMVILVQFKEMENSTKSINKF

>DmelGR39aID

DMKRNAFEELRVQLRTLKWLGVLRFTIDFNKCLVRENASEERSAWLYLIGVVGITCSLIVYSTYFPSHFIMGKHNTTGNCYALINIRSCSIVTMLIYTQLYIQRFRFVALLQSILRFNQISGSHREEGRFAFYYYTHLSLLIICMLNYAYGYWTAGVRLTTIPIYLLQYGFSYLFLGQVVVLFACIQQILLSILKYYNQVVLKNIKSSKESREFYYNFCKYNQVIWLSYTEINHCFGLLLLLVTGLILLITPSGPFYLVSTIFEGRFRQNWQFSLMSFTAILWSLPWIVLLVLAMGRNDVQKEANKTAKMLTKVPRTGTGLDRMIEKFLLKNLRQKPILTAYGFFALDKSTLFKLFTAIFTYMVILVQFKEMENSTKSINKF

>DmelGR39aIC

CMDFQPGELCAYYRLCRYLGIFCIDYNPTKKKFRLRRSVLCYIVHFALQAYLVGCISVMVTYWRRCFKSELTTTGNHFDRLVMVIALGILVVQNAWLIWLQAPHLRIVRQIEFYRRNHLANVRLLLPKRLLWLIIATNVVYMANFIKTCIFEWLTDASRLFVITSLGFPLRYLVTSFTMGTYFCMVHIVRLVLDWNQSQINAIIDESADLKMTSPNRLRLRVCLEMHDRLMLLCNDEISLVYGFIAWLSWMFASLDVTGVIYLTMVIQTKKSIVLKLITNVVWLSPTFMTCAASFMSNRVTIQANKTAKMLTKVPRTGTGLDRMIEKFLLKNLRQKPILTAYGFFALDKSTLFKLFTAIFTYMVILVQFKEMENSTKSINKF

>DmelGR39aIB

BMGTRNRKLLFFLHYQRYLGLTNLDFSKSLHIYWLHGTWSSTAIQIVVVGVFMAALLGALAESLYYMETKSQTGNTFDNAVILTTSVTQLLANLWLRSQQKSQVNLLQRLSQVVELLQFEPYAVPQFRWLYRIWLLVCLIYGAMVTHFGINWLTTMQISRVLTLIGFVYRCVLANFQFTCYTGMVVILKKLLQVQVKQLEHLVSTTTISMAGVAGCLRTHDEILLLGQRELIAVYGGVILFLFIYQVMQCILIFYISNLEGFHSSNDLVLIFCWLAPMLFYLILPLVVNDIHNQANKTAKMLTKVPRTGTGLDRMIEKFLLKNLRQKPILTAYGFFALDKSTLFKLFTAIFTYMVILVQFKEMENSTKSINKF

>DmelGR22e

MFRPSGSGYRQKWTGLTLKGALYGSWILGVFPFAYDSWTRTLRRSKWLIAYGFVLNAAFILLVVTNDTESETPLRMEVFHRNALAEQINGIHDIQSLSMVSIMLLRSFWKSGDIERTLNELEDLQHRYFRNYSLEECISFDRFVLYKGFSVVLELVSMLVLELGMSPNYSAQFFIGLGSLCLMLLAVLLGASHFHLAVVFVYRYVWIVNRELLKLVNKMAIGETVESERMDLLLYLYHRLLDLGQRLASIYDYQMVMVMVSFLIANVLGIYFFIIYSISLNKSLDFKILVFVQALVINMLDFWLNVEICELAERTGRQTSTILKLFNDIENIDEKLERSITDFALFCSHRRLRFHHCGLFYVNYEMGFRMAITSFLYLLFLIQFDYWNL

>DmelGR68a

MKIYQDIYPISKPSQIFAILPFYSGDVDDGFRFGGLGRWYGRLVALIILIGSLTLGEDVLFASKEYRLVASAQGDTEEINRTIETLLCIISYTMVVLSSVQNASRHFRTLHDIAKIDEYLLANGFRETYSCRNLTILVTSAAGGVLAVAFYYIHYRSGIGAKRQIILLLIYFLQLLYSTLLALYLRTLMMNLAQRIGFLNQKLDTFNLQDCGHMENWRELSNLIEVLCKFRYITENINCVAGVSLLFYFGFSFYTVTNQSYLAFATLTAGSLSSKTEVADTIGLSCIWVLAETITMIVICSACDGLASEVNGTAQILARIYGKSKQFQNLIDKFLTKSIKQDLQFTAYGFFSIDNSTLFKIFSAVTTYLVILIQFKQLEDSKVEDISQA

>DmelGR98d

MEANRSRLLAAARPYIQIYSIFGLTPPIQFFTRTLHKRRRGIVILGYACYLISISLMVIYECYANIVALQKDIHKFHAEDSSKVMGNTQKVLVVAMFVWNQLNILLNFRRLARIYDDIADLEIDLNNASSGFVGQRHWWRFRFRLALSVGLWIVLLVGLTPRFTLVALGPYLHWTNKVLTEIILIMLQLKCTEYCVFVLLIYELILRGRHILQQISVELEGNQSRDSVQELCVALKRNQLLAGRIWGLVNEVSLYFTLSLTLLFLYNELTILQIVNWALIKSVNPNECCQYRRVGTCLLLSINIFLSCLYSEFCIQTYNSISRVLHQMYCLSAAEDYLILKMGLREYSLQMEHLKLIFTCGGLFDINLKFFGGMVVTLFGYIIILVQFKIQFFAQSNFMQNINSTELKAYTA

>DmelGR98c

MEMEAKRSRLLTTARPYLQVLSLFGLTPPAEFFTRTLRKRRRFCWMAGYSLYLIAILLMVFYEFHANIVSLHLEIYKFHVEDFSKVMGRTQKFLIVAIATCNQLNILLNYGRLGLIYDEIANLDLGIDKSSKNFCGKSHWWSFRLRLTLSIGLWMVIIIGVIPRLTLGRAGPFFHWVNQVLTQIILIMLQLKGPEYCLFVLLVYELILRTRHVLEQLKDDLEDFDCGARIQELCVTLKQNQLLIGRIWRLVDEIGAYFRWSMTLLFLYNGLTILHVVNWAIIRSIDPNDCCQLNRLGSITFLSFNLLLTCFFSECCVKTYNSISYILHQIGCLPTAEEFQMLKMGLKEYILQMQHLKLLFTCGGLFDINIKLFGGMLVTLCGYVIIIVQFKIQDFALIGYRQNTSDT

>DmelGR97a

MRFLRRQTRRLRSIWQRSLPVRFRRGKLHTQLVTICLYATVFLNILYGVYLGRFSFRRKKFVFSKGLTIYSLFVATFFALFYIWNIYNEISTGQINLRDTIGIYCYMNVCVCLFNYVTQWEKTLQIIRFQNSVPLFKVLDSLDISAMIVWRAFIYGLLKIVFCPLITYITLILYHRRSISESQWTSVTTTKTMLPLIVSNQINNCFFGGLVLANLIFAAVNRKLHGIVKEANMLQSPVQMNLHKPYYRMRRFCELADLLDELARKYGFTASRSKNYLRFTDWSMVLSMLMNLLGITMGCYNQYLAIADHYINEEPFDLFLAIVLVVFLAVPFLELVMVARISNQTLTRRTGELLQRFDLQHADARFKQVVNAFWLQVVTINYKLMPLGLLELNTSLVNKVFSSAIGSLLILIQSDLTLRFSLK

>DmelGR94a

MDFTSDYAHRRMVKFLTIILIGFMTVFGLLANRYRAGRRERFRFSKANLAFASLWAIAFSLVYGRQIYKEYQEGQINLKDATTLYSYMNITVAVINYVSQMIISDHVAKVLSKVPFFDTLKEFRLDSRSLYISIVLALVKTVAFPLTIEVAFILQQRRQHPEMSLIWTLYRLFPLIISNFLNNCYFGAMVVVKEILYALNRRLEAQLQEVNLLQRKDQLKLYTKYYRMQRFCALADELDQLAYRYRLIYVHSGKYLTPMSLSMILSLICHLLGITVGFYSLYYAIADTLIMGKPYDGLGSLINLVFLSISLAEITLLTHLCNHLLVATRRSAVILQEMNLQHADSRYRQAVHGFTLLVTVTKYQIKPLGLYELDMRLISNVFSAVASFLLILVQADLSQRFKMQ

>DmelGR98b

MVAQKSRLLARAFPYLDIFSVFALTPPPQSFGHTPHRRLRWYLMTGYVFYATAILATVFIVSYFNIIAIDEEVLEYNVSDFTRVMGNIQKSLYSIMAIANHLNMLINYRRLGGIYKDIADLEMDMDEASQCFGGQRQRFSFRFRMALCVGVWMILMVGSMPRLTMTAMGPFVSTLLKILTEFVMIMQQLKSLEYCVFVLIIYELVLRLRRTLSQLQEEFQDCEQQDMLQALCVALKRNQLLLGRIWRLEGDVGSYFTPTMLLLFLYNGLTILHMVNWAYINKFLYDSCCQYERFLVCSTLLVNLLLPCLLSQRCINAYNCFPRILHKIRCTSADPNFAMLTRGLREYSLQMEHLKLRFTCGGLFDINLKYFGGLLVTIFGYIIILIQFKVQAIAANRYKKVVN

>DmelGR98a

MEQMSGELHAASLLYMRRLMKCLGMLPFGQNLFSKGFCYVLLFVSLGFSSYWRFSFDYEFDYDFLNDRFSSTIDLSNFVALVLGHAIIVLELLWGNCSKDVDRQLQAIHSQIKLQLGTSNSTDRVRRYCNWIYGSLIIRWLIFIVVTIYSNRALTINATYSELVFLARFSEFTLYCAVILFIYQELIVGGSNVLDELYRTRYEMWSIRRLSLQKLAKLQAIHNSLWQAIRCLECYFQLSLITLLMKFFIDTSALPYWLYLSRVEHTRVAVQHYVATVECIKLLEIVVPCYLCTRCDAMQRKFLSMFYTVTTDRRSSQLNAALRSLNLQLSQEKYKFSAGGMVDINTEMLGKFFFGMISYIVICIQFSINFRAKKMSNEQMSQNITSTSAPI

>DmelGR36a

MFDWVGLLLKVLYYYGQIIGLINFEIDWQRGRVVAAQRGILFAIAINVLICMVLLLQISKKFNLDVYFGRANQLHQYVIIVMVSLRMASGISAILNRWRQRAQLMRLVECVLRLFLKKPHVKQMSRWAILVKFSVGVVSNFLQMAISMESLDRLGFNEFVGMASDFWMSAIINMAISQHYLVILFVRAYYHLLKTEVRQAIHESQMLSEIYPRRAAFMTKCCYLADRIDNIAKLQNQLQSIVTQLNQVFGIQGIMVYGGYYIFSVATTYITYSLAINGIEELHLSVRAAALVFSWFLFYYTSAILNLFVMLKLFDDHKEMERILEERTLFTSALDVRLEQSFESIQLQLIRNPLKIEVLDIFTITRSSSAAMIGSIITNSIFLIQYDMEYF

>DmelGR92a

MFEFLHQMSAPKLSTSILRYIFRYAQFIGVIFFCLHTRKDDKTVFIRNWLKWLNVTHRIITFTRFFWVYIASISIKTNRVLQVLHGMRLVLSIPNVAVILCYHIFRGPEIIDLINQFLRLFRQVSDLFKTKTPGFGGRRELILILLNLISFAHEQTYLWFTIRKGFSWRFLIDWWCDFYLVSATNIFIHINSIGYLSLGVLYSELNKYVYTNLRIQLQKLNTSGSKQKIRRVQNRLEKCISLYREIYHTSIMFHKLFVPLLFLALIYKVLLIALIGFNVAVEFYLNSFIFWILLGKHVLDLFLVTVSVEGAVNQFLNIGMQFGNVGDLSKFQTTLDTLFLHLRLGHFRVSILGLFDVTQMQYLQFLSALLSGLAFIAQYRMQVGNG

>DmelGR85a

MYSLIEAQLLGGKLVNRVMASLRRIIQRSLGYFCALNGILDFNTDIGTGNLRRYRVLFMYRLLHNFAVISLTLKFLFDFTDHFKYIESSTLITVNFFTYFTLVFFALLSSMGSCYQWQNRILAVLKELKHQRDLSRHMGYRVPRSKQNSIDYLLFALTVLLILRLSIHLATFTLSARMGFNHPCNCFLPECMIFSMNYLLFAILAEITRCWWSLQSGLKMVLLNRQLSTVAFNLWEIERLHTRFQCLIDLTSEVCSIFRYVTLAYMARNLWSGIVAGYLLVRFVIGNGLQDVELVYLVFSFITCIQPLMLSLLVNSMTSTTGSLVEVTRDILKISHKKSVNLERSIEWLSLQLTWQHTHVTIFGVFRINRSLAFRSASLILVHVLYMVQSDYISITN

>DmelGR36c

MDLESFLLGAVYYYGLFIGLSNFEFDWNTGRVFTKKWSTLYAIALDSCIFALYIYHWTGNTNIVNAIFGRANMLHEYVVAILTGLRIVTGLFTLILRWYQRCKMMDLASKVVRMYVARPQVRRMSRWGILTKFIFGSITDGLQMAMVLSAMGSVDSQFYLGLGLQYWMFVILNMAMMQQHMIMLFVRTQFQLINTELRQVIDEAKDLLLSPRHQGVFMTKCCSLADQIENIARIQSQLQTIMNQMEEVFGIQGAMTYGGYYLSSVGTCYLAYSILKHGYENLSMTLSTVILAYSWCFFYYLDGMLNLSVMLHVQDDYWEMLQILGKRTIFVGLDVRLEEAFENLNLQLIRNPLKITVVKLYDVTRSNTMAMFGNLITHSIFLIQYDIEHF

>DmelGR36b

MVDWVVLLLKAVHIYCYLIGLSNFEFDCRTGRVFKSRRCTIYAFMANIFILITIIYNFTAHGDTNLLFQSANKLHEYVIIIMSGLKIVAGLITVLNRWLQRGQMMQLVKDVIRLYMINPQLKSMIRWGILLKAFISFAIELLQVTLSVDALDRQGTAEMMGLLVKLCVSFIMNLAISQHFLVILLIRAQYRIMNAKLRMVIEESRRLSFLQLRNGAFMTRCCYLSDQLEDIGEVQSQLQSMVGQLDEVFGMQGLMAYSEYYLSIVGTSYMSYSIYKYGPHNLKLSAKTSIIVCILITLFYLDALVNCNNMLRVLDHHKDFLGLLEERTVFASSLDIRLEESFESLQLQLARNPLKINVMGMFPITRGSTAAMCASVIVNSIFLIQFDMEFF

>DmelGR22f

MKMFQPRRGFSCHLAWFMLQTTLYASWLLGLFPFTFDSRRKQLKRSRWLLLYGFVLHSLAMCLAMSSHLASKQRRKYNAFERNPLLEKIYMQFQVTTFFTISVLLLMNVWKSNTVRKIANELLTLEGQVKDLLTLKNCPNFNCFVIKKHVAAIGQFVISIYFCLCQENSYPKILKILCCLPSVGLQLIIMHFHTEIILVYRYVWLVNETLEDSHHLSSSRIHALASLYDRLLKLSELVVACNDLQLILMLIIYLIGNTVQIFFLIVLGVSMNKRYIYLVASPQLIINFWDFWLNIVVCDLAGKCGDQTSKVLKLFTDLEHDDEELERSLNEFAWLCTHRKFRFQLCGLFSINHNMGFQMIITSFLYLVYLLQFDFMNL

>DmelGR22a

MSQPKRIHRICKGLARFTIRATLYGSWVLGLFPFTFDSRKRRLNRSKWLLAYGLVLNLTLLVLSMLPSTDDHNSVKVEVFQRNPLVKQVEELVEVISLITTLVTHLRTFSRSSELVEILNELLVLDKNHFSKLMLSECHTFNRYVIEKGLVIILEIGSSLVLYFGIPNSKIVVYEAVCIYIVQLEVLMVVMHFHLAVIYIYRYLWIINGQLLDMASRLRRGDSVDPDRIQLLLWLYSRLLDLNHRLTAIYDIQVTLFMATLFSVNIIVGHVLVICWINITRFSLLVIFLLFPQALIINFWDLWQGIAFCDLAESTGKKTSMILKLFNDMENMDQETERRVTEFTLFCSHRRLKVCHLGLLDINYEMGFRMIITNILYVVFLVQFDYMNLKFKTD

>DmelGR22c

MFASRSDLQSRLCWIILKATLYSSWFLGVFPYRFDSRNGQLKRSRFLLFYGLILNFFLLLKMVCSGGQKLGIPEAFARNSVLENTHYTTGMLAVFSCVVIHFLNFWGSTRVQDLANELLVLEYQQFASLNETKCPKFNSFVIQKWLSVIGLLLSYLSIAYGLPGNNFSVEMVLINSLVQFSFNCNIMHYYIGVLLIYRYLWLINGQLLEMVTNLKLDCSVDSSRIRKYLSLYRRLLELKGYMVATYEYHMTLVLTTGLASNFLAIYSWIVLDISMNINFIYLLIFPLFLLVNVWNLWLSIAASDLAENAGKSTQTVLKLFADLEVKDIELERSVNEFALLCGHCQFNFHVCGLFTINYKMGFQMIITSFLYLIYMIQFDFMNL

>DmelGR28b

MDIEMAKEPVNPTDTPDIEVTPGLCQPLRRRFRRFVTAKQLYECLRPVFHVTYIHGLTSFYISCDTKTGKKAIKKTIFGYINGIMHIAMFVFAYSLTIYNNCESVASYFFRSRITYFGDLMQIVSGFIGVTVIYLTAFVPNHRLERCLQKFHTMDVQLQTVGVKIMYSKVLRFSYMVLISMFLVNVLFTGGTFSVLYSSEVAPTMALHFTFLIQHTVIAIAIALFSCFTYLVEMRLVMVNKVLKNLAHQWDTRSLKAVNQKQRSLQCLDSFSMYTIVTKDPAEIIQESMEIHHLICEAAATANKYFTYQLLTIISIAFLIIVFDAYYVLETLLGKSKRESKFKTVEFVTFFSCQMILYLIAIISIVEGSNRAIKKSEKTGGIVHSLLNKTKSAEVKEKLQQFSMQLMHLKINFTAAGLFNIDRTLYFTISGALTTYLIILLQFTSNSPNNGYGNGSSCCETFNNMTNHTL

>DmelGR28a

MAFKLWERFSQADNVFQALRPLTFISLLGLAPFRLNLNPRKEVQTSKFSFFAGIVHFLFFVLCFGISVKEGDSIIGYFFQTNITRFSDGTLRLTGILAMSTIFGFAMFKRQRLVSIIQNNIVVDEIFVRLGMKLDYRRILLSSFLISLGMLLFNVIYLCVSYSLLVSATISPSFVTFTTFALPHINISLMVFKFLCTTDLARSRFSMLNEILQDILDAHIEQLSALELSPMHSVVNHRRYSHRLRNLISTPMKRYSVTSVIRLNPEYAIKQVSNIHNLLCDICQTIEEYFTYPLLGIIAISFLFILFDDFYILEAILNPKRLDVFEADEFFAFFLMQLIWYIVIIVLIVEGSSRTILHSSYTAAIVHKILNITDDPELRDRLFRLSLQLSHRKVLFTAAGLFRLDRTLIFTITGAATCYLIILIQFRFTHHMDDTSSNSTNNLHSIHLGD

>DmelGR39b

MLYSFHPYLKYFALLGLVPWSESCAQSKFVQKVYSAILIILNAVHFGISIYFPQSAELFLSLMVNVIVFVARIVCVTVIILQVMVHYDDYFRFCREMKYLGLRLQCELKIHVGRLKWQSYAKILALGIGFLVTVLPSIYVALSGSLLYFWSSLLSILIIRMQFVLVLLNVELLGHHVSLLGIRLQNVLECHLMGANCTLDGNANRLCSLEFLLALKQSHMQLHYLFTHFNDLFGWSILGTYVVLFSDSTVNIYWTQQVLVEVYEYKYLYATFSVFVPSFFNILVFCRCGEFCQRQSVLIGSYLRNLSCHPSIGRETSYKDLLMEFILQVEQNVLAINAEGFMSTDNSLLMSILAAKVTYLIVLMQFSSV

>DmelGR93b

MSGLLVMPRILRCLNVSRISAILLRSCFLYGTFFGVITFRIERKDSQLVAINRRGYLWICLVIRLLASCFYGYSYDAWSGQYEDMYLRAFFGFRLIGCLICSVIILVMQFWFGEELINLVNRFLQLFRRMQSLTNSPKNRFGDRAEFLLMFSKVFSLLFVFMAFRLMLSPWFLLTLVCDLYTSVGTGMITHLCFVGYLSIGVLYRDLNNYVDCQLRAQLRSLNGENNSFRNNPQPTRQAISNLDKCLYLYDEIHQVSRSFQQLFDLPLFLSLAQSLLAMSMVSYHAILRRQYSFNLWGLVIKLLIDVVLLTMSVHSAVNGSRLIRRLSFENFYVTDSQSYHQKLELFLGRLQHQELRVFPLGLFEVSNELTLFFLSAMVTYLVFLVQYGMQSQQI

>DmelGR58c

MNQYFLLHTYFQVSRLIGLCNLHYDSSNHRFILNHVPTVVYCVILNVVYLLVLPFALFVLTGNIYHCPDAGMFGVVYNVVALTKLLTMLFLMSSVWIQRRRLYKLGNDLMKMLHKFRFNLGNDCRNRCLCKGLLTSSRFVLLTQQLLTRDSVVNCESNSSLRQAMVPYQSAAIVYALIMILLMSYVDMTVYMVEVAGNWLLVNMTQGVREMVQDLEVLPERNGIPREMGLMQILAAWRKLWRRCRRLDALLKQFVDIFQWQVLFNLLTTYIFSIAVLFRLWIYLEFDKNFHLWKGILYAIIFLTHHVEIVMQFSIFEINRCKWLGLLEDVGNLWDINYSGRQCIKSSGTILSRKLEFSLLYMNRKLQLNPKRVRRLHIVGLFDLSNLTVHNMTRSIITNVLVLCQIAYKKYG

>DmelGR93c

MIERLKKVSLPALSAFILFCSCHYGRILGVICFDIGQRTSDDSLVVRNRHQFKWFCLSCRLISVTAVCCFCAPYVADIEDPYERLLQCFRLSASLICGICIIVVQVCYEKELLRMIISFLRLFRRVRRLSSLKRIGFGGKREFFLLLFKFICLVYELYSEICQLWHLPDSLSLFATLCEIFLEIGSLMIIHIGFVGYLSVAALYSEVNSFARIELRRQLRSLERPVGGPVGRKQLRIVEYRVDECISVYDEIERVGRTFHRLLELPVLIILLGKIFATTILSYEVIIRPELYARKIGMWGLVVKSFADVILLTLAVHEAVSSSRMMRRLSLENFPITDHKAWHMKWEMFLSRLNFFEFRVRPLGLFEVSNEVILLFLSSMITYFTYVVQYGIQTNRL

>DmelGR57a

MAVLYFFREPETVFDCAAFICILQFLMGCNGFGIRRSTFRISWASRIYSMSVAIAAFCCLFGSLSVLLAEEDIRERLAKADNLVLSISALELLMSTLVFGVTVISLQVFARRHLGIYQRLAALDARLMSDFGANLNYRKMLRKNIAVLGIVTTIYLMAINSAAVQVASGHRALFLLFALCYTIVTGGPHFTGYVHMTLAEMLGIRFRLLQQLLQPEFLNWRFPQLHVQELRIRQVVSMIQELHYLIQEINRVYALSLWAAMAHDLAMSTSELYILFGQSVGIGQQNEEENGSCYRMLGYLALVMIPPLYKLLIAPFYCDRTIYEARRCLRLVEKLDDWFPQKSSLRPLVESLMSWRIQAKIQFTSGLDVVLSRKVIGLFTSILVNYLLILIQFAMTQKMGEQIEQQKIALQEWIGF

>DmelGR59c

MVDLVKTILLIAYWYGLAVGVSNFEVDWLTGEAIATRRTTIYAAVHNASLITLLILFNLGNNSLKSEFISARYLHEYFFMLMTAVRISAVLLSLITRWYQRSRFIRIWNQILALVRDRPQVVRGRWYRRSIILKFVFCVLSDSLHTISDVSAQRKRITADLIVKLSLLATLTTIFNMIVCQYYLAMVQVIGLYKILLQDLRCLVRQAECICSIRNRRGGVYSIQCCSLADQLDLIAERHYFLKDRLDEMSDLFQIQSLSMSLVYFFSTMGSIYFSVCSILYSSTGFGSTYWGLLLIVLSTASFYMDNWLSVNIGFHIRDQQDELFRVLADRTLFYRELDNRLEAAFENFQLQLASNRHEFYVMGLFKMERGRLIAMLSSVITHTMVLVQWEIQNDES

>DmelGR59d

MADLLKLCLRIAYAYGRLTGVINFKIDLKTGQALVTRGATLISVSTHLLIFALLLYQTMRKSVVNVMWKYANSLHEYVFLVIAGFRVVCVFLELVSRWSQRRTFVRLFNSFRRLYQRNPDIIQYCRRSIVSKFFCVTMTETLHIIVTLAMMRNRLSIALALRIWAVLSLTAIINVIITQYYVATACVRGRYALLNKDLQAIVTESQSLVPNGGGVFVTKCCYLADRLERIAKSQSDLQELVENLSTAYEGEVVCLVITYYLNMLGTSYLLFSISKYGNFGNNLLVIITLCGIVYFVFYVVDCWINAFNVFYLLDAHDKMVKLLNKRTLFQPGLDHRLEMVFENFALNLVRNPLKLHMYGLFEFGRGTSFAVFNSLLTHSLLLIQYDVQNF

>DmelGR58a

MLLKFMYIYGIGCGLMPAPLKKGQFLLGYKQRWYLIYTACLHGGLLTVLPFTFPHYMYDDSYMSSNPVLKWTFNLTNITRIMAMFSGVLLMWFRRKRILNLGENLILHCLKCKTLDNRSKKYSKLRKRVRNVLFQMLLVANLSILLGALILFRIHSVQRISKTAMIVAHITQFIYVVFMMTGICVILLVLHWQSERLQIALKDLCSFLNHEERNSLTLSENKANRSLGKLAKLFKLFAENQRLVREVFRTFDLPIALLLLKMFVTNVNLVYHGVQFGNDTIETSSYTRIVGQWVVISHYWSAVLLMNVVDDVTRRSDLKMGDLLREFSHLELVKRDFHLQLELFSDHLRCHPSTYKVCGLFIFNKQTSLAYFFYVLVQVLVLVQFDLKNKVEKRN

>DmelGR58b

MLHPKLGRVMNVVYYHSVVFALMSTTLRIRSCRKCLRLEKVSRTYTIYSFFVGIFLFLNLYFMVPRIMEDGYMKYNIVLQWNFFVMLFLRAIAVVSCYGTLWLKRHKIIQLYKYSLIYWKRFGHITRAIVDKKELLDLQESLARIMIRKIILLYSAFLCSTVLQYQLLSVINPQIFLAFCARLTHFLHFLCVKMGFFGVLVLLNHQFLVIHLAINALHGRKARKKWKALRSVAAMHLKTLRLARRIFDMFDIANATVFINMFMTAINILYHAVQYSNSSIKSNGWGILFGNGLIVFNFWGTMALMEMLDSVVTSCNNTGQQLRQLSDLPKVGPKMQRELDVFTMQLRQNRLVYKICGIVELDKPACLSYIGSILSNVIILMQFDLRRQRQPINDRQYLIHLMKNKTKV

>DmelGR59e

MDSSYWENLLLTINRFLGVYPSGRVGVLRWLHTLWSLFLLMYIWTGSIVKCLEFTVEIPTIEKLLYLMEFPGNMATIAILVYYAVLNRPLAHGAELQIERIITGLKGKAKRLVYKRHGQRTLHLMATTLVFHGLCVLVDVVNYDFEFWTTWSSNSVYNLPGLMMSLGVLQYAQPVHFLWLVMDQMRMCLKELKLLQRPPQGSTKLDACYESAFAVLVDAGGGSALMIEEMRYTCNLIEQVHSQFLLRFGLYLVLNLLNSLVSICVELYLIFNFFETPLWEESVLLVYRLLWLAMHGGRIWFILSVNEQILEQKCNLCQLLNELEVCSSRLQRTINRFLLQLQRSIDQPLEACGIVTLDTRSLGGFIGVLMAIVIFLIQIGLGNKSLMGVALNRSNWVYV

>DmelGR23aIB

BMFPPTRVQASSRVVLKIFHFILVAFSLRSRRLSRLVLWLQFLGWLTWFISMWTQSVIYAQTIDCTLDCSLRHILTFFQTVSHAFIVVTSFLDGFRIKQDQLDEPIAFEDSDPWLAFTVLAMLVPTLGVEYLVCSNAPEYAFRIRIYHLKTLPSFLALQVQIISFILEVMKVNIRVRQTKLQLLILARELSCRWPQRKQKPQFSDQQAHRVKDLKRRYNDLHYLFVRINGYFGGSLLTIIIVHFAIFVSNSYWLFVDIRTRPWRIYAILLNLGFIFNVALQMAAACWHCQQSYNLGRQIGCLISKLVKPQGSKLYNDLVSEFSLQTLHQRFVVTAKDFFSLNLHLLSSMFAAVVTYLVILIQFMFAERSSTRGSG

>DmelGR59f

MRSSATKGAKLKNSPRERLSSFNPQYAERYKELYRTLFWLLLISVLANTAPITILPGCPNRFYRLVHLSWMILWYGLFVLGSYWEFVLVTTQRVSLDRYLNAIESAIYVVHIFSIMLLTWQCRNWAPKLMTNIVTSDLNRAYTIDCNRTKRFIRLQLFLVGIFACLAIFFNIWTHKFVVYRSILSINSYVMPNIISSISFAQYYLLLQGIAWRQRRLTEGLERELTHLHSPRISEVQKIRMHHANLIDFTKAVNRTFQYSILLLFVGCFLNFNLVLFLVYQGIENPSMADFTKWVCMLLWLAMHVGKVCSILHFNQSIQNEHSTCLTLLSRVSYARKDIQDTITHFIIQMRTNVRQHVVCGVINLDLKFLTTLLVASADFFIFLLQYDVTYEALSKSVQGNVTRYK

>DmelGR23aIA

CMKTLECLTRRFLEVIFSVLALVPLPPISQLGWLFLSLAIRCCWIVYFIYLLDVAISFSWVAIENVGNAVGTMLFVGNSVLGFALLLESVLKQKTHSQLEDLRVQTELQLQRLGMFGRSRHAAYLLPLIGVQFTCDLVRLATNFGETVSPVFCISLPLMWLLRYRYVQLVQHVMDLNQRSIHLRRSLLSMASGNDLWQPYGVQECLQLQTLRTTYERIFECYETFSDCYGWGMLGLHLLTSFQFVTNAYWMIMGIYDGGNVRSLIFNGATGIDFGTPIATLFWHGDSGAENNQAGPVGRTDCVRGLRSLVGLHCAGDAGSHFGSRVFGVLERARICLSYQDLSPENAAQFSGSAGADYILYPGGHEGEHKGFVVTAKDFFSLNLHLLSSMFAAVVTYLVILIQFMFAERSSTRGSG

>DmelGR2a

MDTLRALEPLHRACQVCNLWPWRLAPPPDSEGILLRRSRWLELYGWTVLIAATSFTVYGLFQESSVEEKQDSESTISSIGHTVDFIQLVGMRVAHLAALLEALWQRQAQRGFFAELGEIDRLLSKALRVDVEAMRINMRRQTSRRAVWILWGYAVSQLLILGAKLLSRGDRFPIYWISYLLPLLVCGLRYFQIFNATQLVRQRLDVLLVALQQLQLHQKGPAVDTVLEEQEDLEEAAMDRLIAVRLVYQRVWALVALLNRCYGLSMLMQVGNDFLAITSNCYWMFLNFRQSAASPFDILQIVASGVWSAPHLGNVLVLSLLCDRTAQCASRLALCLHQVSVDLRNESHNALVGTLVRYCAPLIILVPLQITQFSLQLLHQRLHFSAAGFFNVDCTLLYTIVGATTTYLIILIQFHMSESTIGSDSNGQ

>DmelGR77a

MPLPLGDPLALAVSPQLGYIRITAMPRWLQLPGMSALGILYSLTRVFGLMATANWSPRGIKRVRQSLYLRIHGCVMLIFVGCFSPFAFWCIFQRMAFLRQNRILLMIGFNRYVLLLVCAFMTLWIHCFKQAEIIGCLNRLLKCRRRLRRLMHTRKLKDSMDCLATKGHLLEVVVLLSSYLLSMAQPIQILKDDPEVRRNFMYACSLVFVSVCQAILQLSLGMYTMAILFLGHLVRHSNLLLAKILADAEHIFESSQKAGFWPNRQELYKGQQKWLALELWRLLHVHHQLLKLHRSICSLCAVQAVCFLGFVPLECTIHLFFTYFMKYSKFILRKYGRSFPLNYFAIAFLVGLFTNLLLVILPTYYSERRFNCTREIIKGGGLAFPSRITVKQLRHTMHFYGLYLKNVEHVFAVSACGLFKLNNAILFCIVGAILEYLMILIQFDKVLNK

**OBP:**

>LstiPBP1

MTYPKMWTSKILVMMVAACVMTVMVDSSQSVMTSMTKNFIKAYEACAKEYNLPESTGQELINFWKEGYTVTSREAGCAILCLSSKLDLLDPEGKLHHGNTVEFAKQHGSDDAMAHKVVEILHSCEKAAAPNEDMCLVALDVSMCFKKEVHSLNWAPDNELLFEELVGEMSKT

>LstiPBP2

MNSPLIVSISFYLINFSTSFPDEATAKQIMTIVHECEEKFATNEDHCARAMEVSRCFRDHMHRLQWAPSVDVLVGEILVEMA

>LstiPBP3

MGFSVRLLVVLVAVTIYGVNSSQDIIKQMTINFGKALDSCRKELDLPDSINADFYNFWKEGYELSNRQTGCAIMCLSSKLDLVDPEGKLHHGNTHEFAKKHGADDAMAKQLVDLIHKCESDVPDDPDPCLKVLNIAKCFKAEIHKLNWAPSMDLIVAEVLAEV

>LstiGOBP1

MTDVTLGFGQALEQCREESGLSEEKMEEFFHFWSEDFKLEARELGCALRCMSNHFNLITDSNRMHHANAEKFVKSFPNGEVLAKQLVGMLHECEKKHHDEEDNCFRVLHMAACFREACRGASLAPTMEMLLAEFIMQGEN

>LstiOBP1

MMAAMTGRAVLVAAALAALALGARAMDDEMAELAKMLHDNCGEETGVDLGLVDKVNAGADLMPDGKLKCYIKCIMETAGMMSDGEMDVEAVLALLPDDMRRKNEPSLRACGTQKGADDCDTAFLTQVCWQKANKADYFLI

>LstiOBP2

VYRIFIRFYVNMPYNPKCTFKASIRASSLLYIYGKERKKSSLYSTLTMAKGMDGRFLLLLVFIISACDAMTKQQLKNSGKMLKKSCMGKNQVTEDQIGSIEKGKFIEEKPVMCYIACIYQMTQIVSIV

>LstiOBP3

ISFQELSEEIKEIIQHVHNECVGKTGVAEEDITNCENGIFKEDKKLKCYMFCLMEEANLVEDDGSVDYDMVISIIPEQYQERAKNMIYSCNHLDTPDKDKCQRAFDVHKCSYDKDPDFYFLF

>LstiOBP4

MAKFTILCLGVLAAAISSARALTPEELTKIEGDMLVHVQDCAKKFDVDESDLKKAKEEENIDGVDPCLIGCVFKNIKLVNDKGLYDPDVAIESSKSYLSDDADKAKFAEIAKDCASVNDESVSDGEEGCERSKLLLVCFGKHKHLLMKE

>LstiOBP5

MKFIIKYVLVLAITLVCDGLVDIEKYLKICDRNSVDVNDCLLEAAQEGLAVLANGIQDLDVPSIDPYNQKDLRIEYKNNQIYAKFIAKNIYVEGLKESTVHDARLRADEDRFHLELDLTTPKINVRGQYAGEGRYNSLQIQANGEFITNMTDLVYTWKLDGVPEKN

>LstiOBP6

MIVKKHHCVFVNFFVLILLIDVSFGMTRQQLKNSGKLMKKSCMPKNDVTEEQVGEIEQGKFIEERNVMCYIACVYSMTQVVKNNKLSYDAVIKQVDMMFPPEMKDAVKASAAHCKDISKKYKDICEASYWTAKCMYDFDPKNFVFP

>LstiOBP7

MKTFIVLAVCFVVAQAFTDEQKEKLKKHKTECLSETKADEQLVSKLATGDYKAENDALKKYALCMMIKSELMTKDGKFKKDVALAKVPNPADKPQVEKVIDACLANKGNTPHQTAWNYVKCYYEKDPKHAILQ

>LstiOBP8

DSKMFCRVILLSSVYFLALTPYSINAMTEAQKEMIKQHFEQLGMECIGDNPITEQDINDLRAKKAPSGPGGPCFLACIQRKIGVMDEHGMMQNENALELAKKSIPG

>LstiOBP9

MSIVVVFLSLVPALVKCSGEGNIRLLEEEVATAMKACAVPSEDPKDGSAANQRQRRSEDYPSVDNNDNNTGQNVYSYERRVLNLTDIRDQMYILNATDYDYGGYGAGSAGEKYLLTVPRPASGRSYYGNSSDNANRTRRSEPLLKPESNQCLSQCIFANLQVVDSKGIPREAELWGKVQSSVTSQQSRAALRDQIRACFQELQSDAEDNGCSYSNKLERCLMLRFSDRLKADRSKTQANNQKT

>LstiOBP10

LVKVNNKSTFYNIYYPFRLNIDCAKICSDSVRSHDMFLDLPVRLQLNLGQDVLTDFLYYWQEDRQFTNKQVGCTVICVSKKLNLLDKAGRLSQPDAEAYVKTAGGGNSHFFYLSSTSQPTSTAGQRPPLLFLRCPHPFTYQRP

>LstiOBP11

MFGVIGLFVLMFATCRANVAVSRSDTPQVLCGLIPDKLNSCGHLPCGLIPDKLNSCGHLPTIVSAESAKKCGSSSNSCQRMTCIFQESGWMDGKSVNNAKLSEYLDHFSSEHPDWTAAIQHAKTTCLVPNLPAQGFHLNCPAYDVVTCVFRSFVWNIPPSLWSSSSDCEPVRQYAAACPVCPTDCFSPAIPVGSCNACRALPRSP

>LstiOBP12

MCHSLLCIVIFAVIVLDCNALNCRSEGGPKENELKSVYMTCLKKQDGKNSSDSHGYTEDQDWKETRGQSKFHHRSKWGSGSMGEIDDRMRDRDDRMDDRDDRTNREDRTRSRDDRMGGRNDRMGGRDDMNSRDRMGGRENIMNDRNNMMNRDEDANRYGREPLRGRHDFPQSDEYETDMTRYGYHSTTQSTRRFKRSRRTEINSGQRSQYNPNSRKPSQYEETYKDEERNSSNSSRESDNKACALHCFMEQLEMTDDNGMPDRYLVTHAITKDVKNEDLRDFLQESIEECFQILDNENTEDKCEFSKNLMMCLSEKGRANCDDWKDDLKF

>LstiOBP13

MRPFLFLCLVMAVAGNSHHAQLSQAQKEKVQQYTMQCIKQTGVKPDVLAEAKKGHFSDDEALKKFALCFFQKAGIVDSNGKLNVEAALAKIPSSVNKADAMKLLEECKKKSGKDAADTAFEVFKCYSRGTKTHILV

>LstiOBP14

MFHRVLSIILFGLFTCNVKGDFSNELQKKFVGYLGECWQTYELTPKDLEDLKLLKMPDSENVKCYFACVYKKAEMMNDKGEFWEEGVKKTSLEQYGNDDALLKKVNDFIDICKKVNDEPVTDGEKGCERAALMFKCSNEHAPEFGFI

>LstiOBP15

MVRKISALLCCLCVFGISLSDSAISADSEKRCRNPPTAPQKIERVITLCQDEIKLSILREALDVIKEEHTMPEKRRRNKREVPFTHDEKRIAGCLLQCVYRKVKAVDGYGFPTLEGLVGLYSDGVNERGYFMAVLEASRECLMRHHDHFSRTVPMDNGRNCDVSFDIFECISDRIGEYCGNSGL

>LstiOBP16

MLTKALDCTKGNFVSSKELQMMMNHQLPGTKNSDCYIACVFKKVEWLDEKGNYNIEATHKMADKEYADDATKMENAKKLFDHCKTVNDEAVTDGEAGCDRGHYLAKCLIDNAPKMGFDLSKY

>LstiOBP17

MYLKRTLVVLCTVLVLGSAAFVDNIPKCGAKDTDCHRQSFQYVIREGSKTGIPEANIAPFDPLELKQELNIPIRDIVQLHFGDGVVKGLSKCVINDFVTNVEQGKASLDITCNFTVKGHYKANSSSPVIKSLLGGESVHGDGRVKIKIVKLNLKLDFDFIVDKRNGDTYFKRKGNNIKFKYDVLGQVMFAADGLYLGDRDASELLTNMLNQNWKLVMASVGDDIMKDSMGAVEEFVRNFFENVPTKYFITDDLTPYASN

>LstiOBP18

MCYIACVYKTIQVVKNEKIDRDLVFKQVDILYPADMKAAVKSAVEQCYGVQAKYNDLCEAAYYAAKCLYETDPPNFVFP

>LstiOBP19

MFKLILSCIAVAVCMKSVNSLTPDQKAAVQAKLLTSGLHCIRDHPLNLDEIKMLRDKKLPEGENAKCFTACLFKQIGIMDDMGKLNAANAVKSAEEVFKSSDKHLEKSKQIIQECISVNDAPTSDGAKGCDRAKLAFSCLIEGADKHGLHITF

>LstiOBP20

MRFLCLCLLLQSVLYSEATFGTPYLVNSRLCQNWTCVNSKLGLPNSLPPRDQYTQILKTLLPSGAWQDVVERVLDSCYGTRPRNYVGTCPGQALLLCTVDNLIENCPEESWRKDDGCYPVTSLAGTKN

>LstiOBP21

MFKSGVYIALFACLLEMAMSLTEEHKLKLQATFETVGEKCAKENNITEEDIAAFKERKFPDGQEAACFSACVLKNIGLIDDEGQLSHDLAVENAKAVFGEGDEIKAIEEFIETCKDSVADGADACERAKLVFKCFVEHSEKFNF

>LstiOBP22

MRKFFWIIIVFVSGVKTDLLHQDRSKGATLKPISACCDIPELGDEKPLSECSNPKLPGPCNDVHCVFEKSGFLVDKNTLNKDAYRRHLRQWAENHKDWSDAIERAITDCVDKDLRQYLDYPCRAYDVFTCTGIAMLKKCPKDAWKC

>LstiOBP23

PLLFYLDQELGSVRCRDKTVMEDFIDNCLPKKASAPHEIAWNYTKCYHTQAKEPHNKKKKLDYLNIFY

>LstiOBP24

FVSNVQGEDGKNHWNIKNWTYTYDLKGKSNVYFENLFNKESFLGQTAQEMVASNGNAIIHDIGKPIITSIVTEIVHNVQRFFKAVPSEDLSLD

>LstiOBP25

MDGQLLVLKLQGNGNAHFKLRNTQFNVICEHNEKIGKDGKLHYNIKNTKYTYDLKGKVDIELENLLQGNEVLAAAAREVFTTNANIIADEIAPKFIKAVVDKIVKNVNNFFHAAAVEDIEIV

>LstiOBP26

MIQIVFLVLAFISGYSHALTEEEIKAEFTKLVMKCLKDHPVDMSELTNLQKLVVPKKNDVKCLLACAYKLDGIMNAKGLYDLDHAYKVAELTKNGDEKRLENGRKMADECVKINDIEVSDGEKGCERAGLMFKCAIENAPKFGFKL

>LstiOBP27

MLLPFVTHARISVMYAHDKLSDIVAEQCFNEMFPKTKHVEVQESDEPCLIFCVMKKLGIMSPNGAINLETYRKRVLMAHQHDQRTLVSDFGSSCVENAEATQHKQDVCKKAKVFND

>LstiOBP28

MTWVLAVALLAVFGAVQSASTGCKNCISLGKEEKAMFRAHSDACLPQSEVDPKLVEAMLNGELTDDPALKRHVYCVLLKCKVISKDGKLQKTAVLGKMANRADGKNATKVLEGCAEQHGDTPEEIAWNLFRCGYDKKAVLFEYMPTNIGNSEIDNNS

>LstiOBP29

MIKLLFIVLLSISGSSHAMTEAEIKADFIKLVMKCLKDHPVEMTELIKLQSLEVPKKPEVKCLLACAYKLDGLMTEKGLYNIEHAYKVAEVTKNGDEKRLENGKKIADICVKVNENEVSDGEKGCERAGMVFKCVVENAPKFGFKI

>LstiOBP30

LFCLFASICAVIGDHEKDSPIMAMVHKTLVITAHSCMDQINATETDLEYLRHDPPYPEKASCIIKCLLEK

>CsupGOBP1

MEAAKVIMAGLLVVGVVPSMRADMVVMKDITLGFGAALEHCREESGLTQENMEEFFDFWREDFKFEHRELGCALRCMSRYFNLITDTNRMHHENTENFIKSFPNGEKLSKVLVQVIHECEKKFDHEEDHCWRILHIGECFRDMCRSQNIAPDMEMLLAEFIMQAESDTNPVAL

>CsupGOBP2

MVCSGFYLGLVVMAAVTSVKGTAEVMSHVTAHFGKALDECREESGLSTEVLEEFKHFWSEDFEVVHRELGCALICMSNKLSLLHDDTRVHQVNMHDYVKSFPNGEVLSEMMVKLIKNCERQYDDIKDDCDRTVKVAACFKADAKKEGIAPEITMIEAVIEIY

>CsupPBP1

MLYKQMIILDEPQLNCLMVRDTMMLKLVVVMCLTMTVMVDSSQTVMKSMTKNFLKAYEVCAKEYSLKEGTAGILIGFWKDDFSTTSRDVGCAILCLSTKLDLIDPEGKLHHGKATEFAMQHGSGEEMAKKLVEILHNCEQTVTPNEDKCMRALDIAMCFKKELHTLGWAPDPELLFEELIAEMR

>CsupPBP2

MSLYMRIVVLALVYLFNGVESSQEIMKQLSLNFGKAYDSCKKELELPNEVDTDFFNFWKEDYQLTNRLTGCAIMCMSNKLDLLDPDGKMHHGNAREFAKKHGADDSMAQQLLDILHNCEKGASPGPDGDACVQVLEISKCFKVEIHKLNWAPSMDLIMAEVLADV

>CsupPBP3

MAASMKCCLFGILVCFNVMVSDVESSQELIKKMSISFLKVLQECKLELSVPEEVLQSLMTFWNQDTDLSHRELGCVILCVVSKLDLIELETYKLHPDNANEYVKKHGADDETASQIMNILRGCEIKNEAISDHCDRVREIAKCFHGHMHELKWAPNMEVIINELVATKAI

>CsupPBP4

MVQHIAIFALMILSAVSVREVEMVPEYFKSMSRSLLEVLKTCSTELEIKDGIMYEIYQLWKENYDGLSRETGCVLHCMSQKLDLFNVQGKFEHGNTKEFIMKHGADSSTATQLEEMVHICQHKVGEMADECLRVLEMAKCIRGNLTQINWNPNMEVAVEEIVAEA

>CsupOBP1

MTIVFVVLSLLPVLVRCSGDGNIRLLENEVEEALKSCTLLPDDSLKDNNARQRRSNEYTRIDFNDSTIGQNQYGHEKRNSTDMKEQMYVLNATYGNNDYEYGNAGIGNSNGEKFVSSAPRLAAGGDYNKTQMNANRTRRSEPLLNRPDTDQCLSQCIFANLQVVDSRGIPREAEFWNKVQSSVTSQQSRTALKDQTRACFQELQTEAEDNGCSYSNKLERCLMLRFADRKLTGAQQGQNRKT

>CsupOBP2

MVRKISALLCCFCVFGISLSDSAISTESEKRCRNPPTAPQKIERVITLCQDEIKLSILREALDVIKEEHTMPAEKRRNKREVPFTHDEKRIAGCLLQCVYRKVKAVDGYGFPTLEGLVGLYSDGVNERGYFMAVLEASRECLMRHHDHFSRTVPMDNGRNCDVSFDIFECISDRIGEYCGNTGL

>CsupOBP3

MIRQVSVILLAIGFQVISSQGPPPFPPNIPPQCRGPPQVTEKPHECCKIPPFFEDADFEECGFKKADDEHPHERHGPPDCSKQLCMLKKYDLAKEDNIDFEALAKFMDKWVEAHPDFKSSVDAAKERCIGKPLPGPPYICEANKIVFCVSSTLIEFCPKWEATDGCQKLKSHIEECAPLFNRKQ

>CsupOBP4

MTWLLALGLLAVIGDVHPATTGCKNCITLGKEEKAMFRAHSDACLPQSKVDPKLVEGMLSGELTDDPRLRKHVYCVLLKCKVIGKDGKLQKTAVLGKMTNRTDGRNATKVLDSCSDQSGDTPEDLAWNIFRCGYDKKAVLFEYMPTSIAATDVENN

>CsupOBP5

MFRSTVLLCSLYFLALTPYLARAATEEQKAKIKEELEKLGAECMADFPITEDDINDFKSKKIPAGDGVPCFVACMMKKMGVLDEAGMMQKETALELAKSVFHDEEELKIIGDYLHSCASVNTEAVSDGAKGCERAMLALKCMYTNAPKFGFEL

>CsupOBP6

MAKFVVLCLGLLAAALSVKALTKEELDHIKEATLMHFNECNKDFNVSEDDIKAAETQKNMDKIDACLIGCMMKRSHLLDGEGKFDTEKAIELSKSFMKSEDDQKKFAEVVAECAKVNDEPVSDGANGCERSKMVLVCLAKHKAEFVPARR

>CsupOBP7

MLTVSKISFLVLAFLYYVKADSLEDLKKEYTATLVECMKKYEISPADIVQLQEKKMPDNENAKCMVACAYKASGMMDDNGMLSVEGVKKISEKYLSDNPEKMQNAFKFADACKSVNDQQVNDGNKGCERAALIFKCSLEQAAVFNFE

>CsupOBP8

MMEGKKGFHVRFAVLVVLFFIHLSYSMTRQQMKNSGKMMKKSCMPKNDVTEEQVGEIDQGKFLEEKNVMCYIACIYQMTQVVKNNKLNYDAVIKQVDMMFPAELKEPVKAAAAHCKDVGKKYKDICEASYWTAKCMYDFDPKSFVFP

>CsupOBP9

MASFHFKVNCFLYFVLLSSYFVYSMTRQQLKNSGKLLKKACIPKTNVSEEQIRDIDKGKFIEEKNFMCYIACIYTMSQAIKNNKIQHDAMIKQVETMFPNDIKESAKFAIQQCRGIAKQHKDICEAAFWTTKCLYDVDPATFIFP

>CsupOBP10

STEHYPAHHVINMKAFIVLAVCIVAAQALTDEQKEKLKKHKSECLAETKVDEQLVNKLKAGDYKSDNEALKKYALCMLIKSELMTKEGKFKKDVALAKVANPADKPQVEKLIDTCLANKGNTPHQTAWNYVKCYHEKDPKHAIFL

>CsupOBP11

MRCCAVLFVLAFIGCIYAEQEIVHLPPEKVAQILPVAMQCVGESSVPPEVIFQYASGKSLGNDKKYQKFIHCVFTKTGYADETGHINIDKAMEVFPKGTDKEAVKKIMEECSKERGEDPPETSFKFAKCFRKKAPVRITL

>CsupOBP12

MLLIIIAKFLVLVAICEAMTMKQIKNTGKMLRKTCQPKNNAADEKIDPLNEGVFIDEKEVKCYIACIMKMANTMKNGRPNIEVAMKQADLLLPEELKEPAKEALTACRKVPDAHKDVCDAAFHLTQCVYNQNPDIFYFP

>CsupOBP14

MGFSRAVLLAAFVAGAWAMDEEMAELAKMLHDNCGEETGADLSLVDKVNAGADLMPDPKLKCYLKCIMETAGMMTEGVVDVEAVLALLPDDMRAKNEQNLRGCGTQKGADDCDTAFLTQLCWQKANKADYFLI

>CsupOBP15

MLMYHYVQMTRAQLKKTMTVAKKQCVPKIGVSEDKINKIEEGVFIEDPKVMCFIACVYKSLQVIKNDRLDRDLITRQVDILYPNDMKAPVKKAIDKCFHVQDKYSDLCEAVFYGVLCMYKVDPLNFVFP

>CsupOBP16

MFLKLLMLTVLFCAIHAMTRQQLKNSGKMLKKNCMGKNQVTEDQIGSIEKGKFIEDKNVMCYIACIYQMTQVIKNNKLNYEASLKQVDIMYPAELKESAKKSIENCKHISSKYKDICEASY

>CsupOBP17

MNCSVIAIIFAFLSLTSAELELSDEIKEIIQHVHNECVAKTGVAEEDIKNCENGIFKEDEKLKCYMFCLMEEANLADDDGVVDYEMMVSIIPEQYTDRVTKMIFACRHLDTPD

>BmorOBP1

MWKLVVVLTVNLLQGALTDVYVMKDVTLGFGQALEQCREESQLTEEKMEEFFHFWNDDFKFEHRELGCAIQCMSRHFNLLTDSSRMHHENTDKFIKSFPNGEILSQKMIDMIHTCEKKFDSEPDHCWRILRVAECFKDACNKSGLAPSMELILAEFIMESEADK

>BmorOBP2

MFSFLILVFVASVADSVIGTAEVMSHVTAHFGKTLEECREESGLSVDILDEFKHFWSDDFDVVHRELGCAIICMSNKFSLMDDDVRMHHVNMDEYIKGFPNGQVLAEKMVKLIHNCEKQFDTETDDCTRVVKVAACFKKDSRKEGIAPEVAMIEAVIEKY

>BmorOBP3

MSIQGQIALALMVYMAVGSVDASQEVMKNLSLNFGKALDECKKEMTLTDAINEDFYNFWKEGYEIKNRETGCAIMCLSTKLNMLDPEGNLHHGNAMEFAKKHGADETMAQQLIDIVHGCEKSTPANDDKCIWTLGVATCFKAEIHKLNWAPSMDVAVGEILAEV

>BmorOBP4

MGLTETVLKDFYNFWIEDYEFTDRNTGCAILCMSKKLELMDGDYNLHHGKAHEFARKHGADETMAKQLVDLIHGCSQSVATMPDECERTLKVAKCFIAEIHKLKWAPDVELLMAEVLNEVSWKS

>BmorOBP6

MARYNIVVAVLVLGVVGARGSSEAMRHIATGFIRVLDECKQELGLTDHILTDMYHFWKLDYSMMTRETGCAIICMSKKLDLIDGDGKLHHGNAQAYALKHGAATEVAAKLVEVIHGCEKLHESIDDQCSRVLEVAKCFRTGVHELHWAPKLDVIVGEVMTEI

>BmorOBP8

MYTNFILIFYFGISIYDVRASSLDDLKMVYKNVIKECVGDYPITAADLKLIKARQIPNDDIKCVFACAYKKTGMMTEEGMLSVEGIKDMSQKYLSDNPEQLRKSKEFAEACSSVWL

>BmorOBP11

MSANSFVVLAFCALAVGVNALTEEQKAEITKSSLPLIAECSKEFSVNQGDIDAAKKLGDPSGLNSCFVGCFMKKAGIINASGLFDVAATIEKSKKYLTSEEDLKAFEKLTETCAPENDKPVSDSDKGCERAKLLLDCFVANKGSFSVFSL

>BmorOBP13

MLFSKAVTPEESKAFEAFAKPLIEQCQKDFGMDKESFAQKNLDEIDECLIACVVEKFGIDLWGF

>BmorOBP15

MTKQQIKNSGKILKKACISKNDVTEDQISDIDKGKFIEDKNVMCYIACVYSMSQVVKNNKFVHDAMVKQVDMMFPTEMRDAVKASIANCRGVAKNYKDICEASFWTAKCMYEFDPANFVFA

>BmorOBP17

MTRQQLKNSGKIMKKTCMPKNDVTEEEIGQIEQGKFLEQRNVMCYIACIYTVTQVVKNNKLSYDAVIKQVDVMFPAEMRPAVKAAAENCKDISKTFKDICEASYWTAKCMYDFDPKNFVFP

>BmorOBP18

MMRKSCQPKNNVDDEKINPINDGVFIEENEVKCYIACIMKMANTMKNGKLNFEAAMKQADLLLPDEMKEPTKEAIVACRKVADSYKDVCDASFHVTKCIYNHNPSVFFFP

>BmorOBP20

MAVHIFLILASYMALAAHGQLDDEIAELAAMVRENCADESSVDLNLVEKVNAGTDLATITDGKLKCYIKCTMETAGMMSDGVVDVEAVLSLLPDSLKTKNEASLKKCDTQKGSDDCDTAYLTQICWQAANKADYFL

>BmorOBP21

MPVGTFWREREEFSCSVDTDILELEDDKYNKHKQDKVKVRVYYEALCPDSKYFFVKNLAPVTEKLSEFLDVTLVPYGKATTKEINGKYIFSCQHGEEECYANKIHSCSIEAVTNMTKAVKFTTCMITDNNDADEALQRVNDDGTVDYEMFTSLIPEEYFDRATKMIFSCKELDTPDKDKCERAFEVHKCSYEKDPDVSIVTILNVREK

>BmorOBP22

MLKVFVVVVCTLGASQLCAALYTQKVAVSFPKDKTTIVVEAMKSCIAKTGANPNVIEVISSGKVSEDEKFKEFFYCACNDIGVVNPDGHIKVKECIELFPKETQPLVEPVIKNCDKEGVNKYDTLFKYLKCFQETSPVRVTLA

>BmorOBP23

MTSKVLLSCVVLAVLATTVLAEDSRKLVSFAPEVAKKLKVLIQECLNENGLGEDAIEVIRAGEYREDEPFQNLVYCAYKKFGALDENNRIISQVAAASFPKDIDVVTVIESCGKEDGNTPVEQVFKYFKCFQKNSPVRMQLY

>BmorOBP24

MLSMVHTDNKIYSRYAMADGDANFRNMMEELFPDYPQIITSLLDCYRESLHSVATTNTFAKCIQEKTQVKLLIH

>BmorOBP25

MKSVVLICLAFAVFNCGADNVHLNEDEREKANWYTAECGVETGVSTEVINAAKIGKYSKDKAFKKFVLCFFKKSAILNSDGTLNMVVALAKLPSGVNKSEAQSVLEQCKNKTGQDAADKAFAILQCFHKGTKTHILF

>BmorOBP26

MIIILSYSQNVHLAETQKEKAKQYTSECVRESGVSTEAINAAKIGKYSKDKAFKNFVLCFFKKSAIFNSDGTLNMDVALAKLPPGVNKSEAQSVLKQCKNKTGQGAADKAFEIFRCYYKGTKTHILF

>BmorOBP27

MKSVVLICLAFAVFNCGADNVHLTETQKEKAKQYTSECVKESGVSTEVINAAKTGQYSEDKAFKKFVLCFFNKSAILNSDGTLNMDVALAKLPPGVNKSEAQSVLEQCKDKTGQDAADKAFEIFQCYYKGTKTHILF

>BmorOBP29

MTGPAAAAVLLALLAAAGQILGKEERAMFRSHSDACLAQSRVEPRLLESMMNGELIDDAALRKHVYCVLLSCKMIGKDGKLLKAAILGKLAARPAGRDVTKVLEACAEQPGASPEDVAWNIFRCGYNRKAVLFDYMPAGGASSGNTENHP

>BmorOBP30

MREKENEVRALRAFQADCAEDVQVKPDLVVNLKSGDWQTEDVSLKKWALCVLMKLGLMTAQGVFKMNEAMSKIPDMNDKIIAEKLIDDCLSLQATTPHDAAWNYIKCHHQKDPEGNFSSLNIF

>BmorOBP31

MKTFIVFVVCVVLAQALTDEQKENLKKHRADCLSETKADEQLVNKLKTGDFKTENEPLKKYALCMLIKSQLMTKDGKFKKDVALAKVPNAEDKLKVEKLIDACLANKGNSPHQTAWNYVKCYHEKDPKHALFL

>BmorOBP32

MYPKNLYKYPLRIDRNDIPCIIHCVLKKFGIISNDGFINIKNYYRRVQAIHRYDPRILISDVGETCAQNINGMNLDHDVCKKAKVFNDCTQLYAISYREPEDW

>BmorOBP33

MYAHDKLSDMIADQCLNEMYPRSKRLEIEESDEPCIIFCVLKKFGIMSPTGVINLEAYRKRVQLPEQLAQRNSINDFGSACLESAEATQHKQDVCKKAKVFNECTHLYKILLK

>BmorOBP34

MAQSCVIKVRATPKDVRAYFTNSSPVSRSGQCFATCMLEQSDIINHGKVNRDLLVHLAGLVNGKNSRVVRKLNSVSRLCLDSISGMTDRCQLASTYNDCLNENMIEFAFPLDIAEEAVRKMPFHLIQPK

>BmorOBP36

MKSKTKRARENRQTANMAVSEISRILTFLTIVSFIYIVYSFKPLTKDEHIERYNKMNEDIEPFRKNLTECARQVKASMADVEKFLKRIPQSNMEGKCFVACILKRNSLIKNNKLSQENLLEVNRAVYGDDSEVMSRLKTAILECSKIVEDIFEICEYASVFNDCMHMKMEHILDKITMERRMEALGQMSSNPDEWSEEEDEMLKLVKDEL

>BmorOBP37

MFYPFRFTLLFYGLFVIYLVRAEPEKENHFTLALKKTLFSTARSCMSHVNANETDLEYLRKDPPFPDKAACIIKCLLEKINHDQTTECELGNEVVSCIFKYAPELHFKT

>BmorOBP38

MANLVLLLTFVLMTLSMARLKSTEAPKSKTALFNDQDNMGYEELDMEEIMSACNESFRIEYAYLESLNDSGSFPDETDKTPKCYIRCVLEKTEILSENGVLNPATAALVFAGERNGKPMSDLEEMAVACADRHEKCKCEKAYNFVKCLMYMEIDKYEKKN

>BmorOBP39

MIICIVVLSLTEALDVIKEEHTMPAERKRNKREVPFTHDEKRIAGCLLQCVYRKVKAVDGFGFPTLEGLVGLYSDGVNERGYFMAVLEASRECLMKNHDKFSRTTPMGNTTAMQSDLNNNCLTL

>BmorOBP40

MGLLWLFFIFNLALVQAEFGTPYLRKSRLCHQWSCINTKLGFPESLPPREQSAVVLSRILPDGAWRNLTDHILDVCYENRPRTYTNTCPGQGLLHCLMYQMIENCPEESLRKDDVCSPVSSLSGFNYMFSQSMYEDLEEHLPVEIRPEWFLRNTNLDPLDCCDMSEFIQPSWRTQCNFRLNWDNRNRLSIDISHGAATTQTPVPTTKPKALRDFMVVPQSCDKTTCVFKKLNIVSDKGVVDVKSFIKLLDKFTNSYPVWNSAKARVITTCLRKSLIAYDGGCELNNILACTFDVLSENCPLNGNNQTCKHSSRKDTVCQISSSKYRPKHRRDPCSTIPELVNTDILTECNISALSRIEFAPETPIKIKKYGLDISKYKCKGQSVSATCLMDKMEVLNKYTFMDYFKMKDKIRKFTATQPLWTIYNDGYLSAFTNMPMYKEYCSSPKKLLNVVDAMLMTCPESRRQNTQQCRKLFTELTNSIPANKQNLTEEMVNHFHRIFLANVSSPKTGHPKRRIHLKQHKNNPLYYAILNTKEAPRVALLDIPRTSVREPLIIKPVYLRQKNQNTIATPYISDNILRSSPFWLHEQIAAAHSNSTTPVSVARIVLNSTDKIPNLSPNSNVELVTP

>BmorOBP41

MLTILFLLPIVVGIYSCLGNPKIIQPEVSEKCNKPISECDKTRCIFKESGWAKNNVIDKKKVSDYFEQFAKDNPDWSAAVQNFKTTCLSDSLKPQGVDTNCPAYDIIHCALISFIKFASPSQWSTSEQCVYPRQYAGACPVCPERCFAPSVPNGSCNACLALLRTP

>BmorOBP42

MMGYACVFVILAVLQAISAEDPPGLPPFLKDAPEKCKSPPRVKNPNECCISEPFFKEADFIECGIEKPGSERGPPDCSKQNCLLKKYNLLKNDETPDIEAIKSLLDKYIEKNPSFKSSVEKAKECLREDLPGPPQICLANRMTLCIGTVLLMECPDEKWNTTDDCKAFKDHMTECQKYFPK

>BmorOBP43

MTAWGQKQVAQAAKATLKPISACCNIPELGNPEPLAECSNPKLPGPCKDIQCVFEKSGFLTENKTLIKEAYKTHLRQWAKEHEGWSVAVEKAISDCVDKDLRQYLEFPCSAYDVFTCTGIAMLKKCPNEHWTC

>BmorOBP44

MSRLVLFFTILVVLQEFIINLYFNFITEIDSCCVKKYPKLFDSEFITECYNTQRKANDKCERDMCVARKLNLLTEEDSINKDALLRFVEEGFKTEIDLVNAIKKKCFEEDISNIGKPEMCEVAKYKICITSRMAEDCPKWDSKGICSSAQQKVENFMKMLS

>HarmPBP1

MEFHRSTMMSVRLALVVAVCLFIRVDASQDVIKNLSMNFAKPLEDCKKEMDLPDSVTTDFYNFWKEGYEFTNRQTGCAILCLSSKLELLDQELKLHHGKAQEFAKKHGADDAMAKQLVDLIHGCAQSTPDVADDPCMKTLNVAKCFKAKIHELNWAPSMELVVGEVLAEV

>HarmPBP2

MAASRWLFARAFCLVLMMGSAMSSKELLTKMTGGFTKVVDACKTELSVGDHIMQDMYNFWREEYQLVNRDLGCMIMCMTAKLDLIGDDQKMHHGKAEEFAKSHGADDALAKQLVGLIHGCETQHQAIEDHCSRALEIAKCFRTKIHELKWAPSMEVIMEEIMTAA

>HarmPBP3

MGSRHVFFALVVLAVSVRKAEPSKDAMQYITSGFVKVLEECKHELNLNEQILADLFHFWKLEYSLLGRDTGCAIICMSKKLDLLDANGRMHHGNAAEFAKKHGAGDEVASKIVTIIHECEKKHEQDGDECLRVLEVAKCFRTGIHELNWQPKVEVIVSEVLTEI

>HarmGOBP1

MCAKILNTMPGVLRALLVLAAAAPLLADINVMKDVTLGFGQALDKCREESQLTEEKMEEFFHFWRDDFKFEHRELGCAIQCMSRHFNLLTDSSRMHHDNTEKFIQSFPNGEVLARQMVELIHSCEKQFDHEDDHCWRILHVAECFKGSCVQRGIAPSMELMMTEFIMEAEAR

>HarmGOBP2

MTSKSCLLLVAMATLTASVMGTAEVMSHVTAHFGKALEECREESGLSAEVLEEFQHFWREDFEVVHRELGCAIICMSNKFSLLQDDSRMHHVNMHDYVKSFPNGHVLSEKLVELIHNCEKKYDTMTDDCDRVVKVAACFKVDAKAAGIAPEVAMIEAVMEKY

>HarmOBP1

MSKFTFFVLCVVAVSLSKVYASDEDKAKLHEALKPLVEECMKDHEVSLDDLKAAKEAKSADGVKPCFLACVYKKAEVLNDKGEFDADHALEKLKEFVSDEDVLAKVAEVGNTCKAVNDKAVSDGDAGCERAALLTACFLEHKAEILV

>HarmOBP2

MMDRKRLCLLIIAMFLAQGSDAMSRQQLKNSGKMLKKNCMNKNQVTEDQIGSIDKGKFVEDKKVMCYIACIFEMTNVVKNNKLNYDASIKQIDLMYPPDLKESAKAAVEKCKDVQKKYKDICEASYWTAKCMYDFKPEDFIFA

>HarmOBP3

MSKFTCFVLCVLAVSLGEVRSNALEKAAIRAAVYPLIVDCAKEHAVTLEQLKAAKASHSAEGINPCFQSCVYKKTGIFNDNGEYDVANAKTKLQKFVTDEDEYARIAEVGKTCASVNDKSVSDGAAGCERAALLTACFLEHRAQIII

>HarmOBP4

AVAVVLSNVNADDETRASFRQVLGPLVMECRNEFGITEDDLKKAQQERSPDALKPCFIACVFKKFGIITSAGKYDSDASISRIKDVVKNDDLFAKLKSVGEKCNSVNDASVSDGDAGCERAALLAKCFMENKSELSI

>HarmOBP5

MSKFTCLVLCVVAASLSQAYASEEEKAAFREAIKPIVEECSKEHGVSHDELKSAKDNQNADNIKPCFLGCVYKKAEVFNSKGEYDVDKALEKLKKFVSNDEAYAKFAEVGKKCASVNDKAVSDGDAGCERGALLTACFLEHKAEVPL

>HarmOBP6

MSKFTCLLLCVVAVSLSKVHATEEEKEAIRAAVRPIMQECGKEHGVTLDDLKAAKAAHSADGIKPCFQSCVYKKAGIFNDNGEYDIANAKTKLQKFVTNDEEYARIAEVGKMCASVNDKPVTDGAAGCDRAALLTACFLEHRAQIII

>HarmOBP7

MFRFGVLSFVVLLFCMESSYALSSEEELSIKEALHPFVVECAEEYGMTEEMFEEAKKKGSAEDIDPCFMSCFLKKTGFFDDSGKFDAEKSISFAKEHITSESAIKFLEAGAGECVKINDEDVSDGENGCDRAKLLFDCLTELKKKMSE

>HarmOBP7.2

MSRFGVLSFVVLVFCMENIYALSSEEELSIKEALHPFVVECAEEYGMTEEMFEEAKKKGSAEDIDPCFMSCFLKKTGFFDDAGKFDAEKSISFAKEHITSETAIKFLEAGAGECVKINDEDVSDGDKGCDRAKLLFDCLTDLKKKMSE

>HarmOBP8

MLLIEIVKFLTLVAMCEAMTMKQIRNTGKMMRKSCQPKNNVADEQIDPIAEGVFNEDKEVKCYMACIMKMANTIKNGKLNYEAAIKQADLLLPDDIKEPAKEAITACRKVADAYKDICDASFHITKCIYTQNPGIFYFP

>HarmOBP9

MCKFSVLFLYSAVMAVNIWSASCISEEDKAAIITAIAPLAQNCGSECGLDNDDFEKYKEDGSDMDPCFKACLMTQMGVLDKEGKYDGKGLHKAMEEADYPGDKDDAQKFLDELDRCFDAKGDNSGSDEEAKMKRADVLFRCMQDMKEK

>HarmOBP9.2

MCKCSVVFLYLAVMAINIWRASCLSEEDKAAIITAIAPLAQNCGSECGLDNDDFEKYKEDGSDMDPCFKACLMTQMGVLDKEGKYDGKGLHKAMEEADYPGDKDDAQKFLDELDRCFDAKGDNSGSDEEAKMKRADVLFQCMQDMKEN

>HarmOBP13

MFTGTLPLVVFLATFAYGGKEKPVFSDEIKEIIQTVHDECVAKTGVAEEDITNCENGIFKEDPKLKCYMFCLMEEASLVDDDDAVDYDMLVSLIPEEYVDRTTKMIFSCKHLDTPDKDKCQRAFEVHKCSYEKDPDLYFLF

>HarmOBP14

AFAANVSLPPKQNEKANQIATECMKESGLKPEVLAEAKKGHISDDEHLKKFTFCFFKKAGIVSEDGKLNTEVALAKLPPGVDKAEAEKLLETCKGKTGKDVTDTVFEIFKCY

>HarmOBP15

LSLQAFITAMTSTSALLSMLLSWSDIVVVVMASLGIALVLVFADCVRANVMTVLLAIGLTVITFALTQSANTKTSAMPKEAMTTTTMSDQDSSIDNNADVDVIAVMNACNESYRIEMAYLESLNESGSFIDENDKTPKCFIRCVFENVGIVSEDGMQLNPARAAVIFAGQRNGKPMDDIGDMTALCAADRQETCPCDRAYKFIRCLMSMEIERYEKS

>HarmOBP16

MFKLCVVLAFIVATCHGGTLERTSSTCGQIPRELTACLDLQPAVSPEIQEKCRRANECERLTCVFREYNLLDGAEVNKERTAAFLDNFVKQYPSWEVAIDVAKTSCLRSSGLKPQGVFLDCPAYDIIQCVFANLVKNALPSQWSSMSQCNHAREFAAACPICPDACFAPLVPIGTCNACSAARRSS

>HarmOBP17

RWWRCWARWARRAPSPWTSGSMRAWSVTLVALLGALGAARAVAMDEDMAELARMVRENCAAETGADVALVERVNAGADLMPDDKLKCYIKCTMETAGMMADGEVDIEAVLALLPPELAEHNAPSLRACGTVRGADHCDTAFRTQQCWQNANKADYFLI

>HarmOBP18

MTRQQLKNSGKLMKKSCMPKNDVTEEEVGDIEKGKFIESRNVMCYVACIYTMTQVVKNNKLSYEAVIKQVDMMFPAEMRDAVKAAATSCKDITKKSKDLCESAYWTAKCMYDYDAENFVFP

>HarmOBP19

ICRKNAASCRRYKHKARTEHEIKEWLFREGVACNKDFPITPDEMMMLKDNKLPDSTNAKCLIACIFKKTGMIDSKGMFDPDKSIAMTEKDFADNPEKLATSKKLMEACRGVNEQAVADGEKG

>HarmOBP21

MSRAQVKKTMSLVKNQCMPKNSVTEDQVGKIEEGVFLEDRNVMCYVACIYKNLQVVKNDKLDMSLITKQIDALYPPELKEPVKKAVSLCIHSQDNYNDLCEKVFHASKCLYEKDPASFIFP

>HarmOBP22

MTREQIKNSGKLIKKTCMAKNDLSEDQVKDVDKGKFIEEKPFMCYIACVYKMGQTIKGNTVNHDMMIKQVEMMFPNEMKAPMKAAIEHCRPVVKKYKDVCEVSYWTAKCIYEFDPPNFMFP

>HarmOBP23

MVSAVVLLTLLPAWVSCAGEGNIKLLEDKVAVALKSCSYPDDIPSSKESAPKDRQRRSDDSYDGSPRIDDNMKQGSRYSHERRNTNDSGDQLQVINATNDDYDGYGSGDTGEKLLTSMPRPASANNKANMRNHSRTRRSEPLLKPETDQCLSQCVFANLQVVDTRGIPREAELWNKVQSSVTSQQSRSALHDQIRACFQELQSEAEDNGCSYSNKLERCLMLRFADRKVDGKASTQKPASTEKT

>HarmOBP24

MIRSCFVFIAVLQALGVSAQEGPGGPPGDPRQHPILSKIPRKCWAPPPGIDIYRCCPIPKLYPDEIMEQCGIKRASGEGSEELEKIQPGPKVPCKEGICLMQKANLLQENNSVDYSKLRSFLDQWADTNAEFTDAILAAKKICAQDGGPAGPPVCEQDRIFFCLTSNILWNCNLRKLDGCDILQEHMDECRQYYVQDEPEE

>HarmOBP25

MRSVVLFNMFKLFVFLAFTVATCYGAQGQGVLCGPLPRRLTRCLNMAPAISGEIQDKCHESRTATECERLTCIFREYNLLDGTTVNKDRTNAFLDNYVKQYPVWTTAVQHAKAACLGDVALKPQGIDLNCPIYDTLQCIFSNLIKHAIPSQWSTTSECQGYRAFAAACPICPEDCFAAQVPIGSCNACLTLPRSA

>HarmOBP26

MFKSSVFLCCVFFFAITPYLASAMTAEQRAQIHAHFETVGMECNKGSNMITADDIASLRARKIPAGPNAPCFLACLLKHIGIMDDSGLLQKETALEMAKSVFQDPEELKQIEDYLHSCSGVNSESVSDGAAGCERAMLAYKCMTENASQFGIDV

>HarmOBP27

MWNFFIVVLALCSSVAVYALTEEELKLQFTKLIMKCNKDSEVDMQELMQLQSYVVPTKTATKCVLACAYKAASVMNAEGLYDIDHAYKVAEMMKNGDEK

>HarmOBP28

MFNLFFCVFLCGVLSLKVQASSLEDLKMKYVEMIIECSSNYPITAGDMVQLKRKIMPDNESIQCLFACVYKKAGMMDEQGFLSVEGVNEMTRKYLSDDPEKLRKSEEFTDACKSVNDIPVNDGDKGCERAALIFKCTVEKSPDFDFV

>HarmOBP29

MSKFSGLVLCAVAATFISVASGESLSESLRPVIEKCSKEHGVTDADIQAAKAKGSSDGINPCFLFCVFQNAGIFDAKGQYDAATGLKNLRQIVKDNDQYKNLEHAFNDCSKIKDKPVSDAAGCEKGVHLAVCLLEHKTSILI

>HarmOBP30

MKTFVVLAACVMLVQASGLTDEQKEKLKKHRSECLTETKVDEQLVNKLKGGDYKTESEPLKKYALCMMMKSELMTKDGKFKKDVALAKVPNAADKPTVEKLIDACLANKGNTPHQTAWNYVKCYHEKDPKH

>HassPBP1

MVLHRSTIMSVRLALVVAVWLFVRVDASQDVIKNLSMNFAKPLEDCKKEMDLPDSVTTDFYNFWKEGYEFTNRQTGCAILCLSSKLELLDQEMKLHHGKAQEFAKKHGADDAMAKQLVDLIHGCSRSTPDVTDDPCMKALNVAKCFKAKIHELNWAPSMDLVVGEVLAEV

>HassPBP2

MADSRWLFTRVFCLVLVMGSAMSSKELLTKMSEGFTKVVDACKTQLNVGDHIMQDMYNFWREEYQLVNRDLGCMIMCMVAKLDLIGDDQKMHHGKAEEFAKSHGADDVLAKQLVSLIHSCETQHQAIEDHCSRVLEIAKCFRTKIHELKWAPSMEVVMEEIMTAA

>HassPBP3

MGSRHVFFAFAVLAVSVRKAEPSKDAMQYITSGFVKVLEECKHELNLNEQILADLFHFWKLEYSLLGRDTGCAIICMSKKLDLLDANGRMHHGNAAEFAKKHGAGDEVASKIVTIIHECEKKHEQDGDECLRVLEVAKCFRTGIHELDWQPKVEVIVSEVLTEI

>HassGOBP1

GVLRALLVLAAAAPLLADINVMKDVTLGFGQALDKCREESQLTEEKMEEFFHFWSDDFKFEYRELGCAIQCMSRHFNLLTDSSRMHHDNTEKFIQSFPNGEVLARQMVELIHSCEKQFDHEDDHCWRILHVAECFKGSCVQRGIAPSMELMMAEFIMEAEAR

>HassGOBP2

MTSKSCLLLVAMATLTASVMGTAEVMSHVTAHFGKALEECREESGLSAEVLEEFQHFWREDFEVVHRELGCAIICMSNKFSLLQDDSRMHHVNMHDYIKSFPNGHVLSEKLVELIHNCEKKYDTMTDDCDRVVKVAACFKVDAKAAGIAPEVAMIEAVMEKY

>HassOBP1

MSKFTFLVLCVVAVSLSKVYASDEDKAKLHEALKPLVEECMKEHEVSLDDLKAAKEAQSADGVKPCFLACVYKKAEVLNSKGEFDADHALDKLKEFVSDEDVLAKVAEVGNTCKAVNDKAVGDGDAGCERAALLTACFLEHKAEVKPIRPLLFPWGHHHHH

>HassOBP2

MMDRKRLCFLIIAMYLAQGSDAMSRQQLKNSGKMLKKNCMSKNQVTEDQIGSIEKGKFVEDKKVMCYIACIFEMTNVIKNNKLNYDSSIKQIDLMYPPELKESAKAAAEKCKDVQKKYKDICEASYWTAKCLYDFKPEDFIFA

>HassOBP3

MSKFTCFVLCVLAVSLAEVRSNALEKAAIRAALYPLIVDCAKEHSVTLEQLKAAKAAHSAQGINPCFQSCVYKKAGIFNDNGEYDIANAKTKLQKFVTDEDEYARIAEVGKTCASVNDKSVSDGAAGCERAALLTACFLEHRAQIII

>HassOBP4

MSKLTCLVLAAVAVVFSNVNADDEPRASFRQMLGPFVMECKKEFDITEDDLKKAQQEHSPDALKPCFIACVFKKFGIITSAGKYDSDASISRIKDVVKNDDLFAKLKSVTEKCNSVNDASVSDGDAGCERAVLLAKCLIENKSELSI

>HassOBP5

MSKFTCLVLCIVAASLSQAYASEEEKAAFREAIKPIVEECSKEHGVSHDELKSAKDNQNADNIKPCFLGCVYKKAEVFNSKGEYDVDKALEKLKKFVSNDEAYAKFAEVGKKCASVNDKAVSDGDAGCERGALLTACFLEHKAEVPL

>HassOBP6

MSKFTCLLLCVVAVSLSKVHATEEEKEAIRAAVRPIMEACGKEHGVTLDDLKAAKAAHSADGIKPCFQSCVYKKAGIFNDNGEYDIANAKTKLQKFVTDEDEYARIAEVGKTCASVNDKSVSDGAAGCERAALLTACFLEHRAQIII

>HassOBP8

MLLIEIVKFLTLVAMCEAMTMKQIRNTGKMMRKSCQPKNNVADEQIDPIAEGVFNEDKEVKCYMACIMKMANTIKNGKLNYEAAIKQADLLLPDDIKEPAKEAITACRKVADAYKDICDASFHITKCIYTQNPGIFYFP

>HassOBP9.2

MCKFSVVFLYSAVMAVNIWSASCLSEEDKAAIITAIAPLAQNCGSECGLDNDDFEKYKEDGSDMDPCFKACLMTQMGVLDKEGKYDGKGLHKAMEEADYPGDKDDAQKFLDELDRCFDAKGDNSGSDEEAKMKRADVLFQCMQDMKEN

>HassOBP13

MFTGTLPLVVFLATFAYGGKEKPVFSDEIKEIIQTVHDECVAKTGVAEEDITNCENGIFKEDPKLKCYMFCLMEEASLVDDDDAVDYDMLVSLIPEEYVDRTTKMIFSCKHLDTPDKDRCQRAFEVHKCSYEKDPDLYFLF

>HassOBP14

MKSFVVFCVLVAGAFAANVSLPPKQNEKANQIATECMKESGLKPEVLAEAKKGHISDDEHLKKFTFCFFKKAGIVSEDGKLNTEVALAKLPPGVDKAEAEKLLETCKGKTGKDATDTVFEIFKCYHHGTKTHILLGF

>HassOBP15

MTRVLLAIGLTVITFALTQSANTKTSAMPKEAMTTTTMSDQDSSVDNNVDVDVIAVMNACNESYRIEMAYLESLNESGSFIDENDKTPKCFIRCVFENVGIVSEDGMQLNPARAAVIFAGQRNGKPMDDIGDMTALCAADRQETCPCDRAYKFIRCLMSMEIERYEKS

>HassOBP18

MYTHNFGTVFCILCISCFLFGCSNGMTRQQLKNSGKLMKKSCMPKNDVTEEEVGDIEKGKFIESRNVMCYVACIYTMTQVVKNNKLSYEAVIKQVDMMFPPEMRDAVKAAATSCKDITKKYKDLCEASYWTAKCMYDYDAENFVFP

>HassOBP19

MYQARTEHEIKEWLFREGVACNKDFPITPDEMMMLKDNKLPDSTNAKCLIACIFKKTGMIDSKGMFDPDKSIAMTEKDFADNPEKLATSKKLMEACRGVNEQAVADGEKGCDRAKLLFKCIIDSSSKVPK

>HassOBP22

MVYFSYVFICSFILFVTLNSSFISAMTREQIKNSGKLIKKTCMAKNDVSEDQVKDVDKGKFIEEKPFMCYIACVYKMGQTIKGNTVNHDMMIKQVEMMFPNEMKAPMKAAIEHCRPVVKKYKDVCEVSYWTAKCIYEFDPPNFMFP

>HassOBP23

MKQGSRYSHERRNTNDSGDQLQVINATNDDYDGYGSGDMGEKLLTSMPRPASANNKANMRNHSRTRRSEPLLKPETDQCLSQCVFANLQVVDTRGIPREAELWNKVQSSVTSQQSRSALHDQIRACFQELQSEAEDNGCSYSNKLERCLMLRFADRKVDGKASTQKPASTEKT

>HassOBP24

MIRSCFVLVAVLQVLGVSAQEGPGGPPGDPRQHPILSKIPRKCWAPPPGIDIYRCCPIPKLYPDEIMEQCGIKRASGDPSEEPEKPQPGPKVPCKEGICLMQNANLLQQNNSVDYTKLRSFLDQWADTNAEFTDAILAAKKICAQDGGPAGPPVCEQDRIFFCLTSNILWNCNLRKLDGCDILQEHMDECRQYYVQDESEEEKN

>HassOBP25

MFKLFVFLAFTVATCYGAQGQGVLCGPVPRRLTRCLNMAPAISGEIQDKCHQSRTAECERLTCIFREYNLLDGTTVNKDRTNAFLDNYVKQYPVWTTAVQHAKAACLGDVELKPQGIDLNCPIYDTLQCIFGSLIKHATPAQWSTTSECQGYRAFAAACPICPEDCFAAQVPIGSCNACLALPRSA

>HassOBP26

MFKSSVFLCCVFFFALTPYLASAMTAEQKAQIHAHFESVGMECNKGSNMITADDIASLRAKKIPAGPNAPCFLACLLKHIGIMDDSGLLQKETALEMAKSVFQDPEELKQIEDYLHSCSGVNAESVSDGAAGCERAMLAYKCMTENASQFGIDV

>HassOBP27

MWNFFIVVLALCSSVAVYALTEEELKLEFTKLIMKCNKDLEVDMQELVQLQSYVVPTKTATKCVLACAYKAASMNAQGLYDIDHAYKVAEMMKNGDEKRLTNAKKMADICVKVNDIKVSDGEKGCDRAALIFKCTVENAPKFGFKL

>HassOBP28

MFNLFFCVFLCGVLSLKVQASSLEDLKMKYVEMIIECSSNYPITAGDMVQLKRKIMPDNESIQCLFACVYKKAGMMDEQGFLSVEGVNEMTRKYLSDDPEKLRKSEEFTDACKSVNDIPVNDGDKGCERAALIFKCTVEKSPDFDFV

>HassOBP29

MSKFSGLVLCALAATFISVASGESLSESLKQIIEKCSKEHGVSDADIQAAKAKGSSDGINPCFLFCVFQNAGIFDAKGQYDAATGLKNMRQIVKDNDQYKNLEHAFTDCSKIKDKPVSDAAGCEKGVHLAACLLEHKTSILI

>HassOBP30

MKTFVVLAACVMLAQASGLTDEQKEKLKKHRSECLTETKVDEQLVNKLKGGDYKTESEPLKKYALCMMMKSELMTKDGKFKKDVALAKVPNAADKPTVEKLIDACLANKGNTPHQTAWNYVKCYHEKDPKHAIFL

>HassOBP31

MSKFTCIVLCVVAASLTKISHAAISEEEKEAFRAAMAPILAECSAEHGVSEKDIEAAKESGNADAIKPCFLGCLMKKTETLDAKGLFDAEKGLSELKKFIKDDEDLAKFEKIGKICTSVNDQAVS

>HassOBP32

LKVLSVFVALIVALHADDDHEKHFAMFKECAEENGLKMDGFKRGERPVGPPSNEMMCTVKCTMEKEGILSGGKILIEEFKKDPKLTKHVPADKMDAALECLKGVEVSDCSDMKKVMDCTHDMKF

>CpunGOBP2

MLPIWLYFGLVMAAVSSVKSTAEVMSHVTAHFGKALDECRDESGLSPEILEEFKHFWSEDFEVVHRELGCALICMSNKFSLLQEDTRIHHINMHDYVKSFPNGEVLSAKMVELLHNCEKQYDAITDDCDRTVKVAACFKNDCKKEGIAPEITMIEAVMERY

>CpunPBP1

MAAIFKWRLVAILVLGLAVNVRVKASQEVMKKMSATFFKLLEECKKELSVTDDMIQGLVRFWLEDSALGERELGCVIICMAEKQDLVVTEDYRMHHENAYNFAKNHGADDAMATAIVKVIHTCEEQFTSNPDHCARVMEVSKCFRDEIHRLKWAPSIELLIGEMLGEA

>CpunPBP2

MMKDMTKNFLKAYGECQQELHLTDDTARDLMFFWKEDYEVTSREAGCTILCLSKKLEIIDPEGKLHKGKTADFIKQHGSDEETAQKVIDVLHACEASAVPNEDHCIMALGVATCFKKEIHKLNWAPDTEVLLEELMAEMSER

>CpunPBP3

MGTCPKHGNMKGFFVTLLVVFMGGKEVEMSSDGMKQLTTGFLKVLGACKTELGLSDGILSDMYHLWKEEYEQVSRDSGCMFSCMSKKLDILDGDGKIHHDHTKEYVLSNGGGEDLARQLINVAHDCEKQQESLEDECDRMLEIAKCLRRNIKDIQWTPKVEVIITEIVADM

>CpunOBP8

MLLVLIAKFLMLLATCETMTMKQIRNTGKMMRKSCQPKNNATDEQLDPLNEGVFIDEKEVKCYMACIMRM

ANTMKNGEPNYDAAVKQADLLLPEEMKQPAKEALFACKKVPDDYKDPCDAAFHVTKCIFNHNPSIFFFP

>CpunOBP7

MFLGHFYISCLLLIIFDTYHVMSMTRQQMKNSGKILKKTCMPKNDVTEDEVGNIEKGKFIETKNVMCYIACIYSMGQVIKNNKIAHDAMFKQIDMVFPPEMKEPVKAAVEKCKPVAKKYKDICEAAYWTAKCICDADPENFVFA

>CpunOBP6

MFWTGLATIVTVLTVCHGKDTLELSDEIKEIIQHVHNECVGKTGVAEEDIRNCENGIFKDDKKLKCYMFCLMEEANLVDDDDNVDYDMLVSIIPEEYTDRTTKMIFSCRHLDTPDKDKCQRAFDVHKCSYGKDPEFYFLF

>CpunOBP4

MGGRDNMNRNNMDRSNGFGREQFSGRYDFPQSDEYDGDMSRYGYQSSTQPSRRFKRSKRGQNSGQRSQYNPNSRKQSDYEDSYRDEDRNSSNSSSDAESKACALHCFMEQLEMTGDDGMPDKYLVTHAITKDVKNEDLRDFLQESIEECFQILDNEHTEDKCEFSKNLMMCLSEKGRANCDDWKDDLKF

>CpunOBP5

MDGRICLLLVFLVGGSDAMTRAQLKNSAKMLKKNCMAKNSVTEDQIGNIEKGQFIEEKPVMCYIACIYQMMSIVKNNKLNYEASIKQVDMMYPNDLKESVKKSIENCKSVSDKYKDICEASYWTAKCIYEDNPKDFIFA

>CpunOBP3

MLALKCMLEQASKANSLDEKNMIRDKFESVGEECIEKHPLSDEDIAALENKMPPPGRVGACFVACVMKNVGVMDDAGMLQKETALELAREAFDDEEELESIADFLHECSSSINSVAVSDGTEGCDRAILALKCMNEHESKFGLDL

>CpunOBP2

MIRAVTLFCGLFLMALTPNVDAMTEEQRAKIREHFETVGMQCIGDNPLSEEDITALRSKKAPSDSASCFLACMMKNVGVLDDSGMLQKETALELARKVFQDEEELQIISDYLHSCSPVNSAAVSDGAKGCERAMLAYKCMIENASKFGIDV

>CpunOBP1

MVVIRPFPPSGTIGDVLLVSGGPDVYYTIFFSVHVFISRCLANKLYSMFKIKKKKKKKGQFKKKKPVMCYITCIYQMMSIVKNNKLNYKTSIKQVDMMYPNDLKESVKKSIENCKSVSDPYKDICEASYWTAQCIYEDNPKDFIFA

**CSP:**

>LstiCSP1

MKSLVLVALSLLVAVAWARPGATYTDKWDHINVDEILESQRLLRGYVDCLLDKGRCTPDGKALKETLPDALEHDCSKCTAKQKESSEKVIRHLINKQPDFWKELSTKYDPENIYQEKYKDKIEEVKSKN

>LstiCSP2

VFIKTTFSHMVIINVPANLPDAIQNDCKKCSDRQREGADQVMEYIIDHRPDDWVKLEKMYNSDGSYKKKYLDRKEAKNKSATTSGEKSEENVSKSKESQD

>LstiCSP3

MKTIVALCALMAVALARPEETYSDTWDNFNAQELVDNVRLLKNYGKCFLDQGPCTSEGQDFKKRIPEALKTDCGKCSPKQRELIRTVVKGFQAKLPEVWAELVKKHDPEGTYKDSFEAFLNSNN

>LstiCSP4

MKTFILICLSALVVAAAADKFDDLMNVDFEKLLGDEESRKQLVGCLMDELPCGEYQSYRDLIPDLIATNCGKCTPEQKKRHEEVNKFILEKYPTEYNAVVNKYRPKAE

>LstiCSP5

TIQFYVIFQSKYIEKNLRMRRFILLSVLALVALSLAEEEKEKKEEKAEEKYTDRFDDINFEEILANRRLLVPYLKCVLDKGRCTPEGKELKVHIQDAMQTACAKCTDKQKVGARRVVNHIREKEQEYWEELLSKYDPKGEYKSIYEPFLAGKE

>LstiCSP6

MNFLEEHHLQKIFIMRAIVLLSCLVMVYAADKYSSKYDNFDVETLISNDRLLKAYINCFLEKGRCTPEGADFRKALPEAVETTCAKCTEKQKNNIRKVIRAIQQKHPKQWDELVNKTDPSGKHRADFDKFIQGSS

>LstiCSP7

MKFLLLACLLVCAGVSLARRVPRYTDIYDSIDIKTIVANRRLLLPYVLCVLGEGKCTAPGRELRSHIKEALETRCAKCTEAQQRGSRLVIAHLINHEPEYWAKLTAKYDPAGKFAKMYERELKEQV

>LstiCSP8

MQIALVLVMLAACAYAAETPRPQVSDTALEDALNDKRFIQRQLKCALGEAPCDPIGKRLKTLAPLVLRGACPQCSPQETKQIQRTLSYVQRNYPQQWAKIVRQYAG

>LstiCSP9

MKFIVVLAVIVGLAMADEKYTSENDNFDVDALVANIDELKKFSGCFLDINDCDAVAADFKKDIPEAFQQACAKCTDAQKHIFKKFIAGLKEKLPHDYEAFMKKYDPDSKYYPALEKVINV

>LstiCS10

MQKLILLALVCSLGWGVVMAAPQMTDSQLDQTLADRKTMERHLKCALQEGPCDPVGRRLKTLAPLVLRGACWQCSPQETRQIRRTLAYVQRNYPWEWSKIIRQYG

>CpunCSP1

MISTKYLIVLCCVAAAVARPSDKYTDKYDNLNIQEILENKRLLKAYVDCVMGRGKCSPEGKELKEHLQEAIETGCEKCTEAQEKGAYTAIEYLIKNELDIWRELSAHFDPTGKWRKKYEDRARANGIEIPE

>CpunCSP2

MKTIILVGLTIVVAVAWARPQSTYTDKWDHINVDEILESQRLLRGYVDCLLDKGRCTPDGKALKETLPDALENDCSKCTPKQKEASDKVIRYLINKQPEYWKELSVKYDPDNIYQEKYKDKIQEVKATA

>CpunCSP4

MRSCIVFACLLVSVFAAEKYNSKYDNFDVETLISNDRLLKSYVNCFLDKGRCTPEGTDFKKTLPDAVETTCAKCTDKQKTNIKKVIKAIQTRHPRQWDELVKKNDPTGKHIVNFNKFIES

>CpunCSP5

MRAIVLLSCLVVVYAADKYSTKYDNFDVDTLISNDRLLKAYINCFLEKGRCTPEGADFRKALPEAVETTCAKCTEKQKNNIRKVIRAIQQKHPKQWEELVKKTDPSGKHRADFDKFIQSN

>CpunCSP6

MKTFVALFALVAVALARPQETYNTNYDNFDVKQLVENPRVLKNYGKCFLDQGPCTPEGSDFKKTIPEALKTECAKCTPKQRELIRTVVAAFQSKLPEVWAELVQKHDPQGTYKKSFDSFLHASN

>CpunCSP7

MKYDDFDIQPLLDNDRILTGYTKCFLDQGPCTPEAKDFKKVIPEALESSCGKCTPKQKQLIKTVIKAMMERHPDFWTELVDKYDKDKKYRENFNKFIESDDK

>CpunCSP8

MKIVILTLCLATAILAQEKYDAVDDDFDISEVLQNDRLLNSYAKCLLDKGPCTPEVKKVKDKLPEALATRCAKCTDKQKQIGKKLAQEVKNKRPELWKELVAHYDPDGKYQDAFQDYLKP

>HarmCSP2

MKVVLLTLCFALGVLAQDQYESANDNFDISEVIGNDRLLHAYANCLLNKGPCTPEVKQVKEKLPEALETRCAKCTDKQKQMGKALAQEVKKNHPDIWKQLVAMYDPQGKYQQAWKDFLQE

>HarmCSP3

MKVLLVLCLFAAAALADDKYTDKYDNINLDEILENKRLLLAYVNCVMERGKCSPEGKELKEHLQDAIETGRSKCTEAQEKGAYKVIEHLIKNELDIWRELAAKYDPKGDWRKKYEDRARANGIQIPE

>HarmCSP4

MNSLIVFCVLSLAALTIARPDGATYTDKYDNVDLDEILGNRRLMVPYIKCMLDQGKCAPDAKELKEHIKEALENECGKCTEAQKKGTRRVIGHLINHEADFWNELTAKYDPERKYTTKYEKELKEVKA

>HarmCSP5

MRTFVVVCLLGLVAVTLARPESKYTSKYDNINLDEILANQRLLVPYLKCILEEGKCTPEGKELKSHIREALEEDCAKCTENQRKGTRKVLAHLINHEEGYWNRLKAKYDPESKYTAKHEQELRELKH

>HarmCSP6

MKADCFLFVTLIAVVAADFYNSKYDSFDVQPLLENDRILLSYTKCFLDQGPCTPDAKDFKKVIPEALETTCGKCSPKQKQLIKTVIKAVISRHPDAWDQLTEKYDKDQKYKESFDKFLAEQD

>HarmCSP7

MKVFVVLSVLIAFTAAASLTPAELDLAEAFDYEALFSNDEQRKLVFDCILGKGECGDYQKMAEISRKVLESKCADCNPKQKAKYETVLKTIQTKYEPFYNELLKNVAAKKE

>HarmCSP8

MKCIYVLSFLLALAAVQAEDKYSTENDNLDIDAVVANVDTLTSFVACFVDQEPCDAVAADFKKDIQEAVTTRCAKCTDAQKHIFYKFILGLKEELPRGYEEFGRKYDPENKHFSALENAVSPA

>HarmCSP9

MNSLIVFCVLSLAALTIARPDGATYTDKYDNVDLDEILGNRRLMVPYIKCMLDQGKCAPDAKELKEHIKEALENECGKCTEAQKKGTRRVIGHLINHEADFWNELAAKYDPERKYTTKYEKELKEVEA

>HarmCSP10

MKVLVVLSCLIVAAFAADKYNAKYDNFDVDTLITNDRLLKAYINCFLDKGRCTPEGSDFKKTLPEAIETTCGKCTDKQKNNIRKVIKAIQQKHPKEWDALVKKNDPSGKHRANFDKFIQGSR

>HarmCSP11

MNSAIVLCVVALAGMVLARPDGGTYTTKYDNVDLDEILANDRLLIPYIKCLLDEGKCAPDAKELKEHIREALENGCAKCTDKQKEGTRRVIAHLIKHKNADWQKLKAKYDPEGKYTHKYEKELEEVQH

>HarmCSP12

MNSAIVLCVVALAGMVLARPDGDGDKYTSKWDNIDLDEILGNDRLLVPYIKCALDEGKCAPDAKELKEHILEALETGCDKCTDKQKEGTHRVIAHLIKYKLEEWEKLRAKYDPEGKYAKKYEKELEELKRA

>HarmCSP13

MKVLLVLCLFAAAALADDKYTDKYDNINLDEILENKRLLLAYVNCVMERGKCSPEGKELKEHLQDAIETGRSKCTEAQEKGAYKVIEHLIKNELDIWRELAAKYDPKGDWRKKYEDRARANGIQIPE

>HarmCSP14

MNSAIVLCVVALAGMVLARPDGDGDKYTSRWDDVDLDEILENDHLLIPYIKCSLDEGKCAPDAKELKEHIQEALETGCAKCTDKQKEGTRRVIAHLIKKKLQEWEKLKAKYDPEGKYAKKYEKELEEVKNA

>BmorCSP1

MKVLIVLSCVLVAVLADDKYTDKYDKINLQEILENKRLLESYMDCVLGKGKCTPEGKELKDHLQEALETGCEKCTEAQEKGAETSIDYLIKNELEIWKELTAHFDPDGKWRKKYEDRAKAKGIVIPE

>BmorCSP2

MKLLLVFLGLFLAVLAQDKYEPIDDSFDASEVLSNERLLKSYTKCLLNQGPCTAELKKIKDKIPEALETHCAKCTDKQKQMAKQLAQGIKKTHPELWDEFITFYDPQGKYQTSFKDFLES

>BmorCSP3

MNSLIAFCLFAVLAVALARPDDKYTDRYDNVNLDEVLSNSRLLQPYIKCILDKDRCAPDAKELKEHIREALETECAKCTEAQKKGTRRVIGHLINNESKSWNELTAKYDPENKFTAKYEKELREIKA

>BmorCSP4

MKVLIVLSCVLVAVLADDKYTDKYDKINLQEILENKRLLESYMDCVLGKGKCTPEGKELKDHLQEALETGCEKCTEAQEKGAETSIDYLIKNELEIWKELTAHFDPDGKWRKKYEDRAKAKGIVIPE

>BmorCSP5

MNSLIAFCLFAVLAVALARPDDKYTDRYDNVNLDEVLSNSRLLKPYIKCILDKDRCAPDAKELKEHIREALETECAKCTEAQKKGTRRVIGHLINNESKSWNELTAKYDPENKFTAKYEKELREIKA

>BmorCSP6

MKCLTIAALLFVAGLSIAEKYTDKYDNIDVDEILENRKLLVPYIKCVLDEGRCTPDGKELKAHIKDGMQTACAKCTDKQKVSARKIVKHIKQHEADYWEQMKAKYDPKDEFKEIYEGFLAGQN

>BmorCSP7

MKGFYVLCFALFAAVYCKETYSSENDDLDIEALVGNIDSLKAFIGCFLETSPCDAVSGDFKKDIPEAVAEACGKCTPAQKHLFKRFLEVVKDKLPQEYEAFKTKYDPQGKHFDALLSAVANS

>BmorCSP8

MKTILILCALVSVVVCRPEEYYSSQYDNFDVEQLVGNLRLLKNYAKCFLDQGPCTAEGTEFKKRIPEALRTKCAKCNPKQRHLIRTVVKAFQTKLPDLWEELAIKEDPKGQYKHEFTAFINAMD

>BmorCSP9

MKFVLALIALAVVVAARPNDDLFYDKKYDNFNVDEIIDNPRLLKAYTFCFNDKGKCTAEGNDFKKWIPESLQTSCGKCSEKQKYLVAKFVHAIKDKMPDEFDILRKLHDPKGEYTENLDKFLETYGH

>BmorCSP10

MKILIIVVMACVAVTWARPESTYTDKWDNINVDEILESNRLLKGYVDCLLGKGRCTPDGKALKETLPDALEHECVKCTGKQKSGADKVIRHLVNKRPDLWKELAVKYDPDNIYQARYKDKID

>BmorCSP11

MKLTSFLLVGMAMVSAEFYSSRYDDFDVKPLVENDRILQSYTNCFLDKGPCTPDAKEFKKVIPEALETTCGKCSPKQKQLIKTVIKAVIERHPEAWEELVNKYDKDRKFRPSFDKFINEDD

>BmorCSP12

MFMLFIISFIIVPVLKCCGTETSTYTTQYDEVDIKEIMGNERLLVAYIGCLLDKNPCTPEGKELKRNIPDALQSDCSKCSDKQRENADAWIEFMIDNRPEDWTKLEER

>BmorCSP13

MKLLLVFLGLFLAVLAQDKYEPIDDSFDASEVLSNERLLKSYTKCLLNQGPCTAELKKIKDKIPEALETHCAKCTDKQKQMAKQLAQGIKKTHPELWDEFITFYDPQGKYQTSFKDFLES

>BmorCSP14

MKSSLFCVLVLTVVVSSSRQQSYPRNDNININAILQNDRILLGYFKCVMDRGPCTKDGKTFKRALPEALPTACARCSNKQKAAFRTLLLAIRARSEPSFLELLDKYDPSRSNRELLYTFLATGL

>BmorCSP15

MIENFYSKCTISKSVLFLCLIFLPYALNQKYYDSRYDYYDIDHLVQNPRLLKKYLDCFLGKGPCTPIGRLFKQVMPEVITTACAKCTPTQKRFARKTFNAFRRYFPETLMELRRKFDPESKYYDAFEKVITNA

>BmorCSP16

MIEWKRFKILHFLSYLGLLVLVVVCAAQQNRPQVTDTALDEALNDKRFIQRQLKCALGEAPCDPIGKRLKTLAPLVLRGACPQCSPQETKQIQKTLSYVQRNFPQHWAKLVRQYAG

>CsupCSP1

MQRFIIFLVLGMAAMCLAEETTYTDRFDNVDVDEIIANPRLLTAYIRCVLDQGRCTAEGKELKEHVKDAMQTACKKCTDKQKDGARKVVNHIRVNEPQDWEKLVYKYDPIGEYKPIYEPFLAGENLDKNKGTEPSEDQKPDLVEQVQAPANDDKKADLVEARKTESVEFKKTEVVDKSSGLGFQEERHF

>CsupCSP2

MKLQAIILMLVYLRCTSSKEKPYSTKYDSVNLDEILSNERLTTNYINCLLDLGPCSPEAKELKKNVPDAIENDCEKCSDRQREGADQVLDFIIDHRPEDWQKLEIKYNSDGSYKKKYLARKQQQAQNKSDESVKKDGKSNQNSLDELIKSQ

>CsupCSP3

MMSFKYLIMLCLVAAAVARPEDKYTDKYDNLNLDEILENKRLLQAYVNCILDKGKCSPEGKELKEHLQEAIETGCVKCTESQEKGAYRVIEFLIKNELNIWKTMTAKFDPEGKWRKKYEDRAREHGIVVPE

>CsupCSP4

MKAIILIGLSVVVTIAWARPGNTYTDKWDHINVDEILESQRLQKGYVDCLLERGRCTPDGKALKETLPDALEHECSKCTEKQKTASDKVIRHLVNKRPDLWTELAAKYDPDNMYQQRYKDKIEEVKGKQ

>CsupCSP5

MNSLIVFCVVSLAALTIARPDGATYTDKYDNVDLDEILGNRRLMVPYIKCMLDQGKCAPDAKELKEHIKEALENECGKCTEAQKKGTRRVIGHLINHEADFWNELTAKYDPERKYTTKYEKELKEVKA

>CsupCSP6

MKSMTVFCMLAFVAVAYALPQQYTDKYDSVDLTEILSNRRLLVPYLNCILTTGKCSPDGKELRSHIKEALENYCAKCTETQKNGTRRVIGHLINNEADYWKKLVDKYDPERKYVVKYEEELKTVAA

>CsupCSP7

MKSAIVLCLVALAGIALARPEDKYTSRYDSINIPEILQNRRLLVPYVNCALEKGKCTPEGRELKSHIKEALETRCSKCTDAQIRGTRLVLGHLINHEPGYWNQLTNKYDSTGKYTKKYEQELRTQI

>CsupCSP8

MKLIVAVALLCLVAESWAASTYTDKWDNINVDEILESQRLLKAYVDCLLDRGRCTPDGKALKETLPDALENECSKCTDKQKSGSDKVIRHLVNKRPEMWKELSAKYDPNNIYQDRYKDKIEAVKGQ

>CsupCSP9

MKAVFLLCLVVVAVSARPEAQYTNKYDNVNLDEILVNKRLLVPYIKCALDQGKCSPDGRELKSHIREALENYCAKCTPVQQDGTRRVIAHLINHEPDYWRQLSVKYDRDGKFAVKYEKELRTIA

>CsupCSP10

MRSWLVCVLALAGMVCCSAQHFNRYDNFNADSIIQNDRILLAYYKCVMDKGPCTKDGKNFKRVLPETLSTACSKCSPKQKLVVRKLLLGIRVKSEPRFLELLDKYDPQRANREALYSFLVTGV

>CsupCSP11

MKTVVALCAVLAVAMARPETYNSQYDNFDAQELVSNPRLLKNYGMCFLGKGPCTSEGKDFKSTIPDALKTECSKCSPKQRVLIRTVVKGFQEKLPDIWSEIFQKYDPNGEYKEAFERFLNSSD

>CsupCSP12

MKLLIIAVAFAIPCVLAQTKSYTNKYDTVNLDQVLSNRRLLHAYIKCTLDKGKCTSEGRELKSHIAEALQNGCEKCTNAQRAGMKRVIKHLMTYEKAYWTQLVEKFDPAREYSQKYEKELSTL

>CsupCSP13

MAIFVMLAGVAQQVMCYDEKYDKMEVDKIIANDELFNSYLNCWLDKGPCDKENAAEMKTLMPEVISTACGKCTPVQKKNIKKLIQGLTTKQRAHQLKELMLKYDPKREYFANFAKFLLGPE

>CsupCSP14

MKAVILLSCLVVAAYAADKYNAKYDNFDVDTLISNERLLKAYINCFLEKGRCTPEGADFRKALPEAVETTCAKCTEKQKLNVRKVIKAIQQKHPKQWDDLVQKNDPSGKYRASFDKFIQGS

>CsupCSP15

MKFAIVLAVALVSVVAEETYSTENDNFDLGAVLADVEKLRPFSGCFLDKNPCDDLTSAFKKDIPEAVQQACAKCNDRQKQLMKQYLEGIKEKLPQDYVDFKKKYDPENKYFENLNKAVGIA

>CsupCSP16

MRTVIFLSLLLVAIVIAEDKYDSIGDNININELLENDRLLKSYVKCLLNKGPCTPEVKKVKDTLPEALATRCAKCTERQKQIGKQLAKEVKKRHPELWNEMIAFYDPEGKYQDAFQDYLKP

>CsupCSP17

MRCLVILSAVLAVALAETYSTTHDQVDVEALVTNPDSLKAITNCFVDKGDCTETTAAFKKVLPEATEQACAKCTPAQKHMLKRYLEEVKKTFPADMDVLKQKYDPEGKHIEALRAALANA

>CsupCSP18

MQISYLLVLCGCVCACVAQAQTQRPPVSDTALEDALNDKRFIQRQLKCALGEAPCDPIGKRLKTLAPLVLRGACPQCSPQETKQIQRTLSYVQRNYPQQWAKIVRQYAG

**SNMP:**

>LstiSNMP2

MLGKRSKMFFGISLGALVVSVILAAWGFPKIVSKQIQKNVQIDNSSAMFEKWRKMPMPLTFNIYVFNVTNAEDVNNGAKPKLQEIGPYSYKEYREKTILGYGDNDTVSYMLQKTFVFDQEASGGLSEDDEVTVIHFSYMAAILTVNDMMPSISGVVNGALEQFFSNLTDPFLRVKVRDLFFDGIYLNCAGNHSALGLVCGKLKTDSPPTMRPAEDGKGYYWSMFSHMNRTPSGPYEMIRGRDNVKELGHIVSYKGKRFMKNWSNDQYCGMINGSDSSIFPPIDENDVPDKLYTFEPEVCR

>LstiSNMP1

MHLQKPLKIGLGMMSAGLFGIMFGWVLFPVILKSQLKKEMALSKKTDVRGMWEKIPFALDFKVYLFNYTNPEEVQKGGIPIVKEVGPYHFDEWKEKVEVEDHEEDDTITYKKRDYFYFRPDLSGPGLTGEETIVIPHILMLSMATIVHNDKPAMLNMLGKALNGIFDEPKDIFLRVKVLDLLFRGMIVNCARTEFAPKAVCTALKKEAANGLTFEPNNQYRFSLFGLRNGTIDPHVVTVRRGIKNVMDVGKVIAVDGKPNQDVWRDKCNEYQGTDGTVFPPFLTEKDNLESFSGDLCRSFKPWYQKKTSYKGIKTNRYIANIGDFANDPDLQCYCDSPDKCPPKGVMDLMKCMKAPMYATLPHFLDCDPQLLKNVKGLSPDVNEHGIVIDFEPISGTPMVAMQRVQFNMMLLKADKLDLIKELPGTLTPIFWIEEGLSLNKTFVKMLKHQLFIP

>OnubSNMP1

MQLQKPLKIGLGMMGAGLFGIIFGWVLFPVILKSQLKKEMALSKKTDVRAMWEKIPFALDFKVYMFNYTNVEEIMKGAAPIVKEIGPFHFDEWKEKVDIEDHDEDDTITYKKRDYFYFRPDKSGPGLTGEEVVVMPHLLMLSMATIVNNDKPAMLNMLGKAFNGIFDEPKDIFMRVKVLDLLFRGIIINCARTEFAPKAVCTALKKEGATGMTFEPNNQFRFSLFGMRNGTIDPHVVTVRRGIKNVMDVGKVIAIDGKTEQDVWRDKCNEFEGTDGTVFPPFLTEKDNLESFSGDLCRSFKPWYQKKTSYRGIKTNRYVANIGDFANDPELQCYCDSPDKCPPKGLMDLMKCMKAPMYASLPHYLDSDPQLLKDVKGLSPDANEHGIEIDFEPISGTPMVAKQRVQFNIILLKADKMDLIKDLPGTMTPLFWIEEGLALNKTFVKMLKNQLFIPKRIVSVVKWLLAGVGFVGLVGSVVYQFKGKMINFALSPSSAPVTKVNPEINQQNQPKDISIIGESQNPPKVDM

>OnubSNMP2

MLGKHTKLFFGVSLVALIVSVILAAWGFPKIVSKQIQKNIQIDNSSVMFEKWRKIPMPLTFNVYVFNVTNVEDVNNGAKPRLQQIGPYAYKEYRERTVLGYGDNDTVSYTLKKTFIFDQEASGSLSEDDEVTVIHFSYMAAILTVNDMMPSITGVVNGALEQFFTNLTDPFLRVKVKDLFFDGVYVNCAGNHSALGLVCGKLKADAPQTMRPAGDGNGFYFSMFSHMNRTESGPYEMVRGRENIKELGHIISYKGKSFMKNWGNDMYCGQLNGSDASIFPPIDENNVPEKLYTFEPEVCRSLYASLVGKSSMFNMSAYYYEISSDALASKSANPGNKCYCKKNWSANHDGCLIMGILNLMPCQDAPAIASLPHFYLASEELLEYFDRGISPDKEKHNTYIYLEPVTGVVLKGLRRLQFNIELRNIPMVPQLAKVPTGLFPLLWIEGGAELPDSIIQELRQSHTLLGYVEAVRWALLAIAIVATAVSAIAVARSGLLPVWPRNANSVSFILSPHPNSDVNKVH

>CmedSNMP1

MQLQKHMKIGLGTAGAGIFGILFGWVLFPVILKSQLKKEMALSKKTDVRAMWEKIPFPLNFKVYMFNYTNPEEVQKGGIPIVKEIGPYHFDEWKEKVGIEDHEEDDTITYKKRDVFIFRPDLSGPGLTGEEIVVIPHVLMMGIATIVNKEKPTMLGMLSKAFNGIFDEPKDVFLRAKVLDLLFRGIIINCQRTEFAPKAVCTALKKEAATGLSFEANNQYRFSLFGLRNGTIDPHVVTVKRGIKNVMDVGKVIAVDGKPEQDVWKDKCNEYQGTDGTVFPPFLTEKDNLESFSGDLCRSFKPWYQKKTSYRGIKTNRYVANIGDFANDPDMQCFCDTPDTCPPKGVMDLMKCMKAPMYASLPHFLDSDPNLLKHVKGLSPDANEHGIEIDFEPISGTPMVAKQRVQFNMLLLKADKLELIQDLPNTLSPVFWIEEGLALNKTFVKMLKTQLFIPKRIVGVVKWLLVTVGVIGMIGTAVYHYKGNIAAFALKPSSATVTKVNPETQNQPKDISVIGEPQNPPKVDM

>CmedSNMP2

MLGKHTKLFFAVSLFAVVISVILATWGFPKIVKKQIQKNVQLDESSQMFEKWRKLPMPLTFNVYVFNVTNVDDVNEGARPKLQELGPYVYKEYRERTILGYGDNNTVKYMLKKTFLFDQEASGALSQDDEVVVINFSYLAAILTVQDMMPSIVGVVNGALEQFFTNLTDAFLRVKVRDLFFDGVHVNCNGNHSALGLVCGKLKTDAPPTMRPAEDGSGYYFSMFSHMNRTETGPYEMVRGRDNIKELGHIVSYKGKASMGGRWGRDPYCGMLNGSDASIFPPIDEADVPDKLYTFEPEVCRSLYASLVGKSSIFNMSAYYYEISRLALASKSANPDNKCFCKKDWSSNHDGCLLMGVLNLMPCQDAPAIASLPHFYLASEELLEYFDGGISPDKEKHNTYMYLEPVTGVVLKGIRRLQFNIELRNIPMVPQLAKVSTGLFPLLWIEEGAELPESVLDELRHSHKLLGYVEAVRWVLLCVSLAALLASGVAVLRAGLLPVWPRNNSVSFILSPHPAENKVH

>CsupSNMP1

MQLPKHLKIGAGTAAAGVFGIIFGWVLFPAILKSQLKKEMALSKKTDVRGMWEKIPFALSFKVFLFNYTNVEEIQKGGVPIVKEIGPYHFDEWKEKLEVEDHEEDDTITYKKRDVFYFRPELSGPGLTGEEIITMPHILMVSIATVVNKEKPAMLNMIGKAFNGIFDGPQHVFMNVKALDIMFRGTIINCARTEFAPKAVCTAIKKEASGLIIEPNNQFRFSLFGMRNDTIDPHVITVKRGIKNVMDVGQVVAVDGNPEQSIWRDSCNMYEGTDGTVFPPFLTENDRLESFSTDMCRSFKALYQKKTSYKGIKTNRYVVTIGDLANDPDLQCFCEAPEKCPPKGTMDLMKCMNAPMYASLPHYLDCDPEVQKKVKGLNPDVNVHGIDIDFEPISGTPMVANQRMMFSLVLQQIDKLDLFKDLPGTMTPLFWIEEGIALNKTFVKMLKNQLFVPKRIVGALRWLLVAVGVCGVIVTGIIHYKGSILGFTLPRGSATVAKVNPETNQPKDISVIGNAQSPPKVDM

>CsupSNMP2

MLAKHMKVFFLASLAALVLAVILAAWGFPRIVSKQIQKNVQLENSSVMFEKWRKLPMPLTFKIYVFNVTNAEDINSGAKPMLTEIGPYVYKEYRERTILGYGENDTVRYTLKKTFIFDAEESGPLTENDEVVVINFSYMAAILAVQEMMPSLTTVVNQALEEFFTDLKDPFMRIKVRDLFFDGIHVNCVGNHSALGLVCGQLKSDTPPTMRPTEDGTGYYFSMFSHMNRTESGPYDMVRGTEDIRELGHVVAYKGERSMSQWGDPYCGQLNGSDSSIFPPIDGGNVPQRLYIFEPEICRSMFATLVGKTTVFNMSAFHYSISSDVLAARSANPNNKCYCRKNWSANHDGCLLMGVMNLAPCQGAPAIASLPHFYLASEELLQYFASGINPDKEKHDTYLYLEPVTGVVLKGLRRFQFNIELRNIPEVPQLAKVPTGLFPLLWIEEGATLPDSVVKELQSSHKLLSYVEAARWILLVVAVIATVVSAVTLARSGVLPVCPRNSNSVSFILNPHPTVIDVNKVH

>HvirSNMP1

MQLPKELKYAAIAGGVALFGLIFGWVLFPTILKSQLKKEMALSKKTDVRKMWEKIPFALDFKVYIFNFTNAEEVQKGATPILKEIGPYHFDEWKEKVEVEDHEEDDTITYKKRDVFYFNPEMSGPGLTGEEIVVIPHIFMLGMALTVARDKPAMLNMVGKAMNGIFDDPPDIFLRVKALDILFRGMIINCARTEFAPKATCTALKKEAVSGLVLEPNNQFRFSIFGTRNNTIDPHVITVKRGIKNVMDVGQVVAVDGKLEQTIWRDTCNEYQGTDGTVFPPFVPETERIQSFSTDLCRTFKPWYQKKTSYRGIKTNRYVANIGDFANDPELNCFCPKPDSCPPKGLMDLAPCMKAPMYASMPHFLDSDPELLTKVKGLNPDVTQHGIEIDYEPITGTPMVAKQRIQFNIQLLKTDKLDLFKDLSGDIVPLFWIDEGLALNKTFVNMLKHQLFIPKRVVGVLRWWVVSFGSLGAVIGIVFHFRDHIMRLAVSGDTKVSKVTPEEPEQKDISVIGQAQEPAKVNI

>HvirSNMP2

MLGKHSKIFFGVSLIFLVIAIVLASWGFQKIVNKQIQKNVQLANDSKMFERWVKLPMPLDFKVYVFNVTNVEEVNQGGKPILQEIGPYVYKQYREKTILGYGDNDTIKYMLKKHFEFDPEASGSLTEDDELTVVHFSYLAALLTVHDMMPSLVTVVNKALEQLFPSLEDAFLRVKVRDLFFDGIYLSCDGDNSALGLVCGKIRAEMPPTMRKAEGSNGFYFSMFSHMNRSESGPYEMIRGRDNVYELGNIVSYKGQENMPMWGDKYCGQINGSDSSIFPPIKEDDVPKKIYTFEPDICRSVYADLVDKRELFNISTYYYEISETAFAAKSANPNNRCFCKKNWSANHDGCLLMGLLNLTPCQGAPAIASLPHFYLGSEELLDYFQSGVQPDKEKHNTYVYIDPVTGVVLSGVKRLQFNIEMRQINNIPQLKSVPTGLFPMLWLEEGATIPESIQQELRDSHKLLGYVEVAKWFLLTIAIISVIASAVAVARANALLSWPRNSNSVSFILGPSVTQVNKGN

>MsexSNMP1

MRLARGIKYAVIGAGVALFGVLFGWVMFPAILKSQLKKEMALSKKTDVRKMWEKIPFALDFKIYLFNYTNPEEVQKGAAPIVKEVGPYYFEEWKEKVEIEDHEEDDTITYRKMDTFYFRPELSGPGLTGEETIIMPHVFMMSMAITVYRDKPSMMNMLGKAINGIFDNPSDVFMRVNAMDILFRGVIINCDRTEFAPKAACTAIKKEGAKSLIIEPNNQLRFSLFGLKNHTVDSRVVTVKRGIKNVMDVGQVVAMDGAPQLEIWNDHCNEYQGTDGTIFPPFLTQKDRLQSYSADLCRSFKPWFQKTTYYRGIKTNHYIANMGDFANDPELNCFCETPEKCPPKGLMDLTKCVKAPMYASMPHFLDADPQMLENVKGLNPDMNEHGIQIDFEPISGTPMMAKQRVQFNMELLRVEKIEIMKELPGYIVPLLWIEGGLALNKTFVKMLKNQLFIPKRIVSVIRWWLLSFGMLAALGGVIFHFKDDIMRIAIKGDSSVTKVNPEDGEQKDVSVIGQSHEPPKINM

>MsexSNMP2

MLAKHSKLFFTGSVVFLIVAIVLASWGFPKIISTRIQKSIQLENSSMMYDKWVKLPIPLIFKVYFFNVTNAEGINEGERPILQEIGPYVYKQYRERTVLGYGPNDTIKYMLKKNFVFDPEASNGLTEDDDVTVINFPYMAALLTIQQMMPSAVAMVNRALEQFFSNLTDPFMRVKVKDLLFDGVFLNCDGDSPALSLVCAKLKADSPPTMRPAEDGVNGYYFSMFSHLNRTETGPYEMVRGTEDVFALGNIVSYKEKKSVSAWGDEYCNRINGSDASIFPPIDENNVPERLYTFEPEICRSLYASLAGKATLFNISTYYYEISSSALASKSANPDNKCYCKKDWSASHDGCLLMGVFNLMPCQGAPAIASLPHFYLASEELLEYFEDGVKPDKEKHNTYVYIDPVTGVVLKGVKRLQFNIELRNMPRVPQLQAVPTGLFPMLWIEEGAVMTPDLQQELRDAHALLSYAQLARWIILAAAIILAIIATITVARSTSLISWPRNSNSVNFIIGPMVNDKMR

>SlitSNMP1

MLLPKELKYAAIAGGVAIFGLIFGWVLFPTILKSQLKKEMALSKKTDVRQMWEKIPFPLDFKVYIFNYTNAEEVAKGAVPILKEIGPYHFDEWKEKVDVEDHEEDDTITYKRRDVFYLNPELTAPGLTGEEIVVIPHVFMLGMALTVQREKPAMLNMVGKAMNGIFDDPPDIFLRVKAMDILFRGMIINCARTEFASKATCTALKKEAVSGLVLEPNNQFRFSIFGTRNNTIDPHVITVKRGIKNVMDVGQVVAVDGQTEQTIWKDTCNEYQGTDGTVFPPFLTENDRLQSFSTDLCRSFKPWYQKKSSYRGIKTNRYVANIGNLAEDPELQCFCPQPDKCPPKGLMDLAPCIKAPMYASMPHFLDCDPALLSKVKGLNPDVNAHGIEIDFEPISGTPLVARQRIQFNIQLLKTDKLDLCKDLSGDIVPLFWIEEGLALNKTFVNMLKHQLFIPKRVVGVLRWWMVSFGSLGAVIGIVFHFRDHIMRLAVSGDSKVSKVTPEEVEEQKDISVIGPAQEPAKINI

>SlitSNMP2

MLGKHSKLIFAVSMGFLVVAVIMAAWGFQKIVDKQIQKNVQLENNSMMFDKWLKLPMPLEFKVYIFNVTNVEDVNQGEKPILNEIGPYVYKQYRERTILGYGPNDTIKYMLRKRFEFDPEASGVLTEDDEVTVINFSYLAAVLTVHDMMPSFVGMVNKALEQFFPSLEDAFLRVKVRDLFFDGIYLNCDGDNAALGLVCGKIKSDTPPTMRPAEGANGFYFSMFSHMNRTETGPYHMIRGRENVYELGNIVSYKEQKVMPMWGDKYCGQINGSDSSIFPPIKEGNVPKKLYTFEPDICRSVYVDLVGKKEIFNISAYYYEISESAFAAKSANPNNKCFCRKNWSANHDGCLLMGLLNLMPCQGAPAIASLPHFFLGSEELLEYFGSGIMPDKEKHNTYVYIDPTSGVVLSGLKRLQFNIELRQIDTVPQLKRVPTGLFPMLWLEEGATIPASIQQELRDSHKLIGYVEVARWFLLTAAIIAVVTSAVAVARANALLSWPRNSNSVSFILGPSVTQVNKGN

>SexiSNMP1

MLLPKELKYAAIAGGVALFGLIFGWVLFPTILKSQLKKEMALSKKTDVRQMWEKIPFPLDFKVYIFNYTNAEEVAKGAVPILKEIGPYHFDEWKEKVEVEDHEEDDTITYKKRDVFYFNPEMSGPGLTGEETVVIPHVFMLGMALTVHREKPAMLNMVGKAMNGIFDDPPDIFLRVKAMDILFRGMMINCARTEFAPKATCTALKKEGVSGLVLEPNNQFRFSIFGTRNNSIDPHVITVKRGIKNVMDVGQVTAVDGQTVQTIWKDHCNEYQGTDGTIFPPFLTENDRLQSFSTDLCRSFKPWFQKKSSYKGIKTNRYVANIGNLAEDPELQCFCPQPDKCPPKGLMDLAPCIKAPMYASMPHFLDCDPALLSKVKGLNPDVNAHGIEIDFEPISGTPLVARQRLQFNIQLLKTDKLDLCKDLSGDIVPLFWIEEGLALNKSFVNMLKHQLFIPKRVVGVLRWWMVSFGSLGALIGVVFHFRDHIMRLAVSGDSKVSKVTPEEGEEQKDISVIGPAQEPAKINI

>SexiSNMP2

MLGKHSKLIFAVSMGFLVVAVIMAAWGFQKIVDKQIQSNVQLENNSMMFDKWLKLPMPLDFKVYVFNVTNVEDVNRGEKPILNEIGPYVYKQYRERTILGYGPNDTIKYMLRKRFEFDPVASGDLTEDDEVTVINFSYLAALLTVHDMMPSFVGMVNKALEQFFPSLEDAFLRVKVRDLFFDGIYLSCDGDNAALGLVCGKIKSDTPPTMRPAEGANGFYFSMFSHMNRSESGPYEMVRGRENVYELGNIVSYKGQKVMPMWGDKYCGQINGSDSSIFPPIKEGNVPKKLYTFEPDICRSVYVDLVGKKEIFNISAYYYEISESAFAAKSANPNNRCFCKKNWSANHDGCLLMGLLNLMPCQGAPAIASLPHFFLGSEELLEYFGSGIKPDKEKHNTYVYIDPTSGVVLSGLKRLQFNIELRQIDTVTQLKRVPTGLFPMLWLEEGATIPASIQQELRDSHKLLGYVEIARWFLLTVAIIAVVTSAVAVARANALLSWPRNSNSVSFILGPSVTHVNKGN
