# Supplementary material for: Identification of candidate chemosensory genes by transcriptome analysis in Loxostege sticticalis Linnaeus
Source: PLoS One. 2017 Apr 19;12(4):e0174036. doi: 10.1371/journal.pone.0174036 (PMC5396883; doi:10.1371/journal.pone.0174036)
Supplement: S3 Table — (DOC) [file pone.0174036.s005.doc]

**Supplementary material 3**

**Primer used in RT-qPCRs**

| **Primer name** | **Primer sequence** |
| --- | --- |
| LstiPBP1 Forward Primer | 5' TGCAATGGCTCACAAGG 3' |
| Reverse primer | 5' GTCAGGTGCCCAGTTCAG 3' |
| LstiPBP2 Forward Primer | 5' GAATGCGAGGAGAAGTC 3' |
| Reverse primer | 5' CTAAGCCATCTCAACCAG 3' |
| LstiPBP3 Forward Primer | 5' CCAAATGTTTCAAAGCGAG 3' |
| Reverse primer | 5' CAGCCAAGACTTCAGCCAC 3' |
| LstiGOBP1 Forward Primer | 5' TCACGCTCGGCTTCGGACAG 3' |
| Reverse primer | 5' CGGTTGGAGTCGGTGATGAG 3' |
| LstiOBP1 Forward Primer | 5' GTGACTGGTGGACAAGG 3' |
| Reverse primer | 5' AGCGAGAGCTCGTTCTG 3' |
| LstiOBP2 Forward Primer | 5' GTGCGTCCAGTCTTCTATAG 3' |
| Reverse primer | 5' GTCATAGCATCACAGGCACG 3' |
| LstiOBP3 Forward Primer | 5' TATGCCTGATGGAGGAAGCG 3' |
| Reverse primer | 5' TCGACAGTATCAAGGTGGTC 3' |
| LstiOBP4 Forward Primer | 5' TGCCTTATAGGCTGCGTCTC 3' |
| Reverse primer | 5' CACAGTCCTTGGCGACATCG 3' |
| LstiOBP5 Forward Primer | 5' CGTCATAGACTCTTACAACC 3' |
| Reverse primer | 5' GTCGATTCCTGTAATCACTC 3' |
| LstiOBP6 Forward Primer | 5' GGCAAGTTCATAGAAGAGCG 3' |
| Reverse primer | 5' TAACAGCGTCTATCATCTCG 3' |
| LstiOBP7 Forward Primer | 5' TCGTGCTCGCCGTATGCTTG 3' |
| Reverse primer | 5' TGCTCGTCAGTCTTGGTATC 3' |
| LstiOBP8 Forward Primer | 5' CTGGTGGTCACTGTCTCCTG 3' |
| Reverse primer | 5' TGCATCATACCGTGTTCGTC 3' |
| LstiOBP9 Forward Primer | 5' ACAACGGCTGTTCCTATTC 3' |
| Reverse primer | 5' TGCTTGCGTCTTGCTTCTAG 3' |
| LstiOBP10 Forward Primer | 5' ACGCTGAAGCCTATGTGAGG 3' |
| Reverse primer | 5' GGTCGCTGGTAAGTGAATGG 3' |
| LstiOBP11 Forward Primer | 5' ATGTTCGGAGTGATTGGGTTAC 3' |
| Reverse primer | 5' CTATGGCATGAGTTACTTGAGC 3' |
| LstiOBP12 Forward Primer | 5' TGCGAACAGATATGGAAGTG 3' |
| Reverse primer | 5' TGAGTCGTAGAGTGGTAGCC 3' |
| LstiOBP13 Forward Primer | 5' AATTCTCATCATGCACAAC 3' |
| Reverse primer | 5' GAACTTCTATAGAGCCTCGTC 3' |
| LstiOBP14 Forward Primer | 5' ATGCTACTTCGCTTGCGTCTG 3' |
| Reverse primer | 5' CATCGTCATCTCCGTATTGC 3' |
| LstiOBP15 Forward Primer | 5' TAAGTGACAGCGGTAGG 3' |
| Reverse primer | 5' GAGAAGTGGTCGTGGTG 3' |
| LstiOBP16 Forward Primer | 5' AAGACAGTTGAATGGCTGAG 3' |
| Reverse primer | 5' CATCTGCGTATTCCTTGTCC 3' |
| LstiOBP17 Forward Primer | 5' TATGCGAGCTGTGGAAG 3' |
| Reverse primer | 5' GGCGTCAGTCATCTGAG 3' |
| LstiOBP18 Forward Primer | 5' CCGCTGACATGAAGGC 3' |
| Reverse primer | 5' TTGAGAGCGTACGTATG 3' |
| LstiOBP19 Forward Primer | 5' TATCGCTGTGGCAGTCTG 3' |
| Reverse primer | 5' TCTCATCCAGGTTGAGTG 3' |
| LstiOBP20 Forward Primer | 5' ACAGCACCAGTCAACAAC 3' |
| Reverse primer | 5' TATAGACGAGTGTAGGAG 3' |
| LstiOBP21 Forward Primer | 5' GAGACGAAATCATAGCC 3' |
| Reverse primer | 5' AATGTTCCACGAAGCAC 3' |
| LstiOBP22 Forward Primer | 5' TAACAAGGATGCCTACCG 3' |
| Reverse primer | 5' ACGTCATACGCTCTACAG 3' |
| LstiOBP23 Forward Primer | 5' GGAAGACTTCATAGACG 3' |
| Reverse primer | 5' GTGGTAGCATCTAGTG 3' |
| LstiOBP24 Forward Primer | 5' TCACAGCAGTGCCATCG 3' |
| Reverse primer | 5' TTAATCAAGGCTCAAGG 3' |
| LstiOBP25 Forward Primer | 5' GGCATAGATGGAAAG 3' |
| Reverse primer | 5' TTGGCGTATGTGGTAG 3' |
| LstiOBP26 Forward Primer | 5' CGACCACGCTTACAAG 3' |
| Reverse primer | 5' TCGTCAGCCATCTTCC 3' |
| LstiOBP27 Forward Primer | 5' CCATCTGTCACTCACG 3' |
| Reverse primer | 5' GTCCGACTCCTGAACCTC 3' |
| LstiOBP28 Forward Primer | 5' CGGCAAGGAGGAGAAG 3' |
| Reverse primer | 5' GAGTCGTCGGTCAGTC 3' |
| LstiOBP29 Forward Primer | 5' ACGGATAGAAGTTGCTGAC 3' |
| Reverse primer | 5' GACCTCGTTCTCGTTCACTG 3' |
| LstiOBP30 Forward Primer | 5' TGGATCAGATCAACGCAAC 3' |
| Reverse primer | 5' TTCTCAGGGTAGTGAGAGTC 3' |
| LstiCSP1 Forward Primer | 5' TCCTGACGCACTAGAACG 3' |
| Reverse primer | 5' TGATGAGGTGCCTGTACG 3' |
| LstiCSP2 Forward Primer | 5' GTTCCAGCAAACCTACCG 3' |
| Reverse primer | 5' AGTTTCACTCAATCGTCG 3' |
| LstiCSP3 Forward Primer | 5' CGCACTTATGGCGGTAG 3' |
| Reverse primer | 5' CGCGTTGAAGTTGTCTC 3' |
| LstiCSP4 Forward Primer | 5' AAGAAAGCAGGAAGCAG 3' |
| Reverse primer | 5' ATGAGATCGAGAATCAG 3' |
| LstiCSP5 Forward Primer | 5' GTGTCGTCAACCACATC 3' |
| Reverse primer | 5' GCAAGGATAGGCTCGTG 3' |
| LstiCSP6 Forward Primer | 5' ATAGGCACTCACAGAAG 3' |
| Reverse primer | 5' TACCAGCTCGTCTCACG 3' |
| LstiCSP7 Forward Primer | 5' TACGTGCTCTGCGTGCTG 3' |
| Reverse primer | 5' TTGATGAGGTGAGCGATG 3' |
| LstiCSP8 Forward Primer | 5' GGCTCACTGTTGCGAGTAAG 3' |
| Reverse primer | 5' CTCATCGAGAAGAGGCTAG 3' |
| LstiCSP9 Forward Primer | 5' ATGCTGTGGCCGCTGATG 3' |
| Reverse primer | 5' GTGCGTCGGTACATATGG 3' |
| LstiCSP10 Forward Primer | 5' GATGATGCGTAGTGATGG 3' |
| Reverse primer | 5' TCTTCTGCCGACTGTGTC 3' |
| LstiIR1 Forward Primer | 5' ACCAAGTGCACCTATATGTG 3' |
| Reverse primer | 5' GTTTCCAGTAACGCCGTATG 3' |
| LstiIR7d.2 Forward Primer | 5' GATAATCCTGAAGAACTCAG 3' |
| Reverse primer | 5' ACTACACATCCAACAACAAG 3' |
| LstiIR7d.3 Forward Primer | 5' AATCTCTGTGAGTCTACAG 3' |
| Reverse primerr | 5' CAACAGAAGTTCGGTAGG 3' |
| LstiIR7g Forward Primer | 5' TCTATACTCTAACCATATGCC 3' |
| Reverse primer | 5' TTCTTCTCATCCTATCATTG 3' |
| LstiIR8a Forward Primer | 5' CTAGTGTGGACTGCGACAAG 3' |
| Reverse primer | 5' GTTAGAGCGAAGGAAGAGGC 3' |
| LstiIR21a Forward Primer | 5' CCACCAATTCCTTGTC 3' |
| Reverse primer | 5' CTATCCTGTGCTGCTG 3' |
| LstiIR25a Forward Primer | 5' GGGCTGTTGTTGGCTGCTAC 3' |
| Reverse primer | 5' ATGCGAGTGTCCAGGCGAG 3' |
| LstiIR40a Forward Primer | 5' CGTTCTGCTTACTTGTGATG 3' |
| Reverse primer | 5' TCGGTGGATATGGATCTCTG 3' |
| LstiIR41a Forward Primer | 5' TGTCTGATATGCGATGCG 3' |
| Reverse primer | 5' GTCTTCTTCGCGTGGTAAG 3' |
| LstiIR64a Forward Primer | 5' TGCCAACAAGCGATGTCAGG 3' |
| Reverse primer | 5' GTCACAGCGTACACGACAG 3' |
| LstiIR68a Forward Primer | 5' TTGGCGACATAATATACAGC 3' |
| Reverse primer | 5' CCAGTGCGTAGTCTAGGTAGG 3' |
| LstiIR75d Forward Primer | 5' CTCTACGCCGACCACATCAG 3' |
| Reverse primer | 5' TCACGGAGCCAGACAACACG 3' |
| LstiIR75p Forward Primer | 5' AAGGGGAAAGGCAAGGTGC 3' |
| Reverse primer | 5' TCACGAGTATGAAGTGGTCG 3' |
| LstiIR75p.1 Forward Primer | 5' TATCTTCACTCTGCCGTTCG 3' |
| Reverse primerr | 5' CATAGTAGCCTCCCATCTGG 3' |
| LstiIR75q.2 Forward Primer | 5' ATACTGTCATACGGTTGCCTCG 3' |
| Reverse primer | 5' CTTCTGGATTATAGTAATGCTTG 3' |
| LstiIR76b Forward Primer | 5' CTGCTGTCGCCGTCTGTATTGG 3' |
| Reverse primer | 5' GCTTCTTGTGTATGGAGTCTCG 3' |
| LstiIR87a Forward Primer | 5' TTACACGCAAGACGATATGG 3' |
| Reverse primer | 5' GTAGTGATCAACCAACGCAG 3' |
| LstiIR93a Forward Primer | 5' AGGTGTCTGACGAGGAGTGGAG 3' |
| Reverse primer | 5' GCTTATAGTGGCTATGTGTTGC 3' |
| LstiGR1 Forward Primer | 5' ATCGTGTTCATCTCCTTCGC 3' |
| Reverse primer | 5' TGCCTATGATTCCATCTTCC 3' |
| LstiGR4 Forward Primer | 5' GGAGGAGTGTGAGTGAGAG 3' |
| Reverse primer | 5' CATAGCGTTCCGTGAAGAGATG 3' |
| LstiGR5a Forward Primer | 5' CCTATCTTCGTATTGTGTGTCG 3' |
| Reverse primer | 5' GCATCCTATCTCTCATCGTCTG 3' |
| LstiGR5b Forward Primer | 5' GTAGGTATCTGGTGTTATGAGG 3' |
| Reverse primer | 5' AAGTATGATATTGGCTATTGG 3' |
| LstiGR6 Forward Primer | 5' CGTATGTGGTGGTGGTTCAG 3' |
| Reverse primer | 5' TCCTTCTTGCGAGATCTGTC 3' |
| LstiGR7 Forward Primer | 5' AGGGCGTGTAGCGTCGTC 3' |
| Reverse primer | 5' TCGTGGCTTAGCTGGTTG 3' |
| LstiGR21a Forward Primer | 5' CATCGTTGTAAGGGTGCGC 3' |
| Reverse primerr | 5' ACAGGTAGGAGGAAGTCGG 3' |
| LstiGR21b Forward Primer | 5' CTACAGGTAGCTGATGGCGC 3' |
| Reverse primer | 5' CAATCTCGTTCTGGGTAGG 3' |
| LstiGR45 Forward Primer | 5' GAGACAAGGCCAGATAGAGG 3' |
| Reverse primer | 5' CTGAATAGGTACAGAAGGAAG 3' |
| LstiGR51 Forward Primer | 5' ATCATACCGTGCTACTACTC 3' |
| Reverse primer | 5' ATTCATCTTCTCTTGTCAAC 3' |
| LstiGR63a Forward Primer | 5' GCTTGGTTGGTATACCGCTGTG 3' |
| Reverse primer | 5' CCGATAGAGTTCGACGCATGG 3' |
| LstiGR63a.1 Forward Primer | 5' GGGACCTAGTCAACAACAC 3' |
| Reverse primer | 5' CAACGGTATACCAAGCAG 3' |
| LstiGR63a.2 Forward Primer | 5' ACTGTGGACCTGTTCTATGG 3' |
| Reverse primer | 5' TTGGTTATGTCTCTGCTTAGG 3' |
| LstiOrco Forward Primer | 5' CATCGCTAACAAGGATACAAAC 3' |
| Reverse primer | 5' CCAGTAGACCTGATATACATAG 3' |
| LstiPR1 Forward Primer | 5' TGACGAAGAGACCATGAGAGG 3' |
| Reverse primer | 5' GCCAACAGATGAAACATATAG 3' |
| LstiPR2 Forward Primer | 5' TCTACATTCTGCGACACAG 3' |
| Reverse primer | 5' TCACATATACAACGCTCTC 3' |
| LstiPR3 Forward Primer | 5' GTCACTCTATCCGTCATC 3' |
| Reverse primer | 5' ACTGTTGTATTCTCTTCC 3' |
| LstiPR4 Forward Primer | 5' AGGTGCTGCGTTCTTACTTGCG 3' |
| Reverse primerr | 5' CGCGGTTGCCTCTTATTCGGTC 3' |
| LstiPR5 Forward Primer | 5' GATACTTCCTCCACCTGC 3' |
| Reverse primer | 5' CATATGACTCCTCCGACG 3' |
| LstiOR1 Forward Primer | 5' TCTGTATGCTGGCTTATC 3' |
| Reverse primer | 5' CGTTCCCTCTCTTCTCTAC 3' |
| LstiOR2 Forward Primer | 5' GACTACTCCTTCGCCACC 3' |
| Reverse primer | 5' ACAACACCATCACGCTCTG 3' |
| LstiOR3 Forward Primer | 5' CACAACACTGCTCATAGAC 3' |
| Reverse primer | 5' CTGTGCCAACAGACAATAGG 3' |
| LstiOR4 Forward Primer | 5' CAGTGTCGTGGCAGAAGAGCC 3' |
| Reverse primer | 5' TGCGAATAATACATATGGCG 3' |
| LstiOR5 Forward Primer | 5' ATTTCTTCTTCAAGCGTAAGCAC 3' |
| Reverse primer | 5' GGTAATCATACCAGAGATGGCAC 3' |
| LstiOR6 Forward Primer | 5' GCGTATGCATCTGTCTTATACTTC 3' |
| Reverse primer | 5' TTCTGCCTTTATCATTATTCCTCG 3' |
| LstiOR7 Forward Primer | 5' GAAGCGATATATTCCTGTGG 3' |
| Reverse primer | 5' GCGTATCGTGGTTCTGGTCG 3' |
| LstiOR8 Forward Primer | 5' CTTCTTATCACAGCGTAGCG 3' |
| Reverse primer | 5' TTCGTCTTGTATCAGCGCAC 3' |
| LstiOR9 Forward Primer | 5' CTAGCGGTCACCACTGACTAG 3' |
| Reverse primer | 5' CAATGCGACAAGGAACAAGC 3' |
| LstiOR10 Forward Primer | 5' GCACAGACTGAAGAGGATG C 3' |
| Reverse primerr | 5' ACAGATGTTGACGGAGCTAG 3' |
| LstiOR11 Forward Primer | 5' CTTAGACCAAGAAGCAGCG 3' |
| Reverse primer | 5' GCATGAACGCGCACATAC 3' |
| LstiOR12 Forward Primer | 5' GCCGCTACTCTGAACCTGG 3' |
| Reverse primer | 5' TACGCCGCCGTTGATACTCG 3' |
| LstiOR13 Forward Primer | 5' AATCAACATTCACACAAGG 3' |
| Reverse primer | 5' GAAGTTCAGCTATCAGACC 3' |
| LstiOR14 Forward Primer | 5' TCATAGTGCTGTTAGTCTGTGG 3' |
| Reverse primer | 5' TCTTTGTGACGTATCATGTTTC 3' |
| LstiOR15 Forward Primer | 5' TATCTTCCACTTCACCACCTTCCTG 3' |
| Reverse primer | 5' ATTTCGCACAGCTGATGACTCCTAC 3' |
| LstiOR16 Forward Primer | 5' CTGGACACTGGTAACAGCAACG 3' |
| Reverse primer | 5' ATCATACGGCACACAAGAACTC 3' |
| LstiOR17 Forward Primer | 5' ACTACCTTACTGCCTCCTTG 3' |
| Reverse primer | 5' ACTACCTTACTGCCTCCTTG 3' |
| LstiOR18 Forward Primer | 5' CAGGTCCAAACAAGATAAGAG 3' |
| Reverse primer | 5' CACGATCCACAGAAGCAATAG 3' |
| LstiOR19 Forward Primer | 5' CTTGATAGACACACAAGACGC 3' |
| Reverse primer | 5' GAACCAAGTATAAGTAGGAAGC 3' |
| LstiOR20 Forward Primer | 5' CGGAGCCGTATCATAAGAATC 3' |
| Reverse primer | 5' ACCTCGCCGAAAGGAGTGTAG 3' |
| LstiOR21 Forward Primer | 5' TGGACCACAGAAAGGCACAC 3' |
| Reverse primer | 5' CAAGGCATAGCACCATAACG 3' |
| LstiOR22 Forward Primer | 5' GCTCACTGTTGCTACATCTAC 3' |
| Reverse primer | 5' ATCGTGTCTGCGATCTACTCG 3' |
| LstiOR23 Forward Primer | 5' AGCCTTCCAGTTCTCTAGTG 3' |
| Reverse primer | 5' AAGTTCTGTGTAGGTCATTC 3' |
| LstiOR24 Forward Primer | 5' AGGAGTTGATTGATCTGGTGGAC 3' |
| Reverse primer | 5' GAATGAGCTGGTAACGATGTTG 3' |
| LstiOR25 Forward Primer | 5' CATATCCGTGGCTGCTAGTCAATC 3' |
| Reverse primer | 5' CAACCGTTCCTCTCTTCCGTGCTC 3' |
| LstiOR26 Forward Primer | 5' TCATCGTCTGACGAGGCAG 3' |
| Reverse primer | 5' TCTGATAGGTGAGGCGGAC 3' |
| LstiOR27 Forward Primer | 5' CTCTATCGCTGGTCATCTC 3' |
| Reverse primer | 5' TTCTGCTTCCTTCAATCC 3' |
| LstiOR28 Forward Primer | 5' TGAGTCAGTTACGACCCG 3' |
| Reverse primerr | 5' CAGTGAAGAGCACGAGCC 3' |
| LstiOR29 Forward Primer | 5' GCACCTCTTCTCCTCAGGC 3' |
| Reverse primer | 5' AGTCATACACGCTGCCCATG 3' |
| LstiOR30 Forward Primer | 5' AACACATGCGTTAGTGGAAG 3' |
| Reverse primer | 5' AAGTAGGAGGGACACGGCGG 3' |
| LstiOR31 Forward Primer | 5' CCAGTGTCGTGGCAGAAGAGCCG 3' |
| Reverse primer | 5' TGCGACTCATACACGATGGGGAG 3' |
| LstiOR32 Forward Primer | 5' TCAGGCTTCGTATGCTATG 3' |
| Reverse primer | 5' AATCCACGAGGTGTTCTC 3' |
| LstiOR33 Forward Primer | 5' ACGACTGTCACAGCTCTG 3' |
| Reverse primer | 5' TAACCTCGCTTCTTCTCG 3' |
| LstiOR34 Forward Primer | 5' ACGATAGAGCACATGGAAGC 3' |
| Reverse primer | 5' TTAGGGTCGCAACAGAAAAG 3' |
| LstiOR35 Forward Primer | 5' AAGATTAGTTGGTTTCTGGGTC 3' |
| Reverse primer | 5' ACGGTTTGGATGATGAGGTATG 3' |
| LstiOR36 Forward Primer | 5' GATGCCCTGTACTGTGTGCC 3' |
| Reverse primer | 5' ACCTTGTATTTCGTTGCTAG 3' |
| LstiIR37 Forward Primer | 5' GTATGCGTGTGTGTGGGAGAAG 3' |
| Reverse primer | 5' TGGTGGCAAACGTTGAAATGTC 3' |
| LstiOR38 Forward Primer | 5' ACCGTGTCTTGGCGCTCTCTC 3' |
| Reverse primer | 5' AGCTTCGTGGTCGCTGTTGAG 3' |
| LstiOR39 Forward Primer | 5' GCCTACAGCAATACAACGAG 3' |
| Reverse primer | 5' TCTCTGAATAAGGGGACGAC 3' |
| LstiOR40 Forward Primer | 5' ATGATCTCTATCCTGTCTCTGG 3' |
| Reverse primer | 5' TCTTTACTCTTATCTGGTGTGC 3' |
| LstiOR41 Forward Primer | 5' TACTTGTTTGTGTGTGCTCG 3' |
| Reverse primer | 5' ACTATCTCTATCCATTCCTG 3' |
| LstiOR42 Forward Primer | 5' TGTTCTGCCTGTCTCACCGC 3' |
| Reverse primer | 5' CGTGTCTATGTCCTCGTCGG 3' |
| LstiOR43 Forward Primer | 5' GGTGGTAATAATCGGAAACGG 3' |
| Reverse primer | 5' CAGCACATCATACAGAACGCG 3' |
| LstiOR44 Forward Primer | 5' GTCACGACAATAGACGACAAGG 3' |
| Reverse primer | 5' ATATCGCCAAAGAAGCACACAC 3' |
| LstiOR45 Forward Primer | 5' TGGTGACTATCATCATCTGTCTTCG 3' |
| Reverse primer | 5' ATCTGCTGTTATATACGGCATCTGCC 3' |
| LstiOR46 Forward Primer | 5' GAAGAAGCGAACTGCCTACACGAG 3' |
| Reverse primerr | 5' GTTCACACCAGCACTCAGACACACG 3' |
| LstiOR47 Forward Primer | 5' TCTGCGACTGTGATTATCGGTTCTG 3' |
| Reverse primer | 5' TTGACGAGCATCTTATAGCCATGTG 3' |
| LstiOR48 Forward Primer | 5' ACTGGTAACAAACGAGCTGAAG 3' |
| Reverse primer | 5' CCCATAGGCGAGTAGACGACAG 3' |
| LstiSNMP1 Forward Primer | 5' ATGACCAACGCTCAGATTG 3' |
| Reverse primer | 5' GGTATAATATAAGCTCCAG 3' |
| LstiSNMP2 Forward Primer | 5' CAGGCTAGAGTCTCGTTCGTTC 3' |
| Reverse primer | 5' CAACACGCGAAATCTCACCAG 3' |
| Actin Forward Primer | 5' GGACAGCGTCAAGCATGG 3' |
| Reverse primer | 5' CGTCGATCGGAGTCCAAG 3' |
