# Supplementary material for: Identification of candidate chemosensory genes by transcriptome analysis in Loxostege sticticalis Linnaeus
Source: PLoS One. 2017 Apr 19;12(4):e0174036. doi: 10.1371/journal.pone.0174036 (PMC5396883; doi:10.1371/journal.pone.0174036)
Supplement: S4 Table — (DOCX) [file pone.0174036.s006.docx]

The accession numbers of 131 candidate chemosensory genes in *Loxostege sticticalis*

| Gene  name | Unigene  reference | Accession  numbers | Gene  name | Unigene  reference | | Accession  numbers | Gene  name |  | | Unigene  reference | | Accession  numbers |
| --- | --- | --- | --- | --- | --- | --- | --- | --- | --- | --- | --- | --- |
| LstiOrco | c57376_g0 | GFCJ01041915.1 | LstiIR1 | c53104_g0 | GFCJ01036972.1 | | LstiPBP1 |  | c59843_g0 | | GFCJ01044841.1 | |
| LstiPR1 | c52064_g0 | GFCJ01035763.1 | LstiIR7d.2 | c57698_g0 | GFCJ01042287.1 | | LstiPBP2 |  | c52747_g0 | | GFCJ01036560.1 | |
| LstiPR2 | c53597_g0 | GFCJ01037529.1 | LstiIR7d.3 | c56115_g0 | GFCJ01040435.1 | | LstiPBP3 |  | c52060_g0 | | GFCJ01035758.1 | |
| LstiPR3 | c55412_g0 | GFCJ01039621.1 | LstiIR7g | c57960_g1 | GFCJ01042596.1 | | LstiGOBP1 |  | c58964_g0 | | GFCJ01043796.1 | |
| LstiPR4 | c55184_g0 | GFCJ01039354.1 | LstiIR8a | c60034_g0 | GFCJ01045092.1 | | LstiOBP1 |  | c54427_g2 | | GFCJ01038484.1 | |
| LstiPR5 | c49318_g0 | GFCJ01032617.1 | LstiIR21a | c57834_g0 | GFCJ01042444.1 | | LstiOBP2 |  | c49708_g0 | | GFCJ01033052.1 | |
| LstiOR1 | c52219_g0 | GFCJ01035943.1 | LstiIR25a | c56710_g0 | GFCJ01041142.1 | | LstiOBP3 |  | c60039_g0 | | GFCJ01045097.1 | |
| LstiOR2 | c51480_g0 | GFCJ01035090.1 | LstiIR40a | c55259_g0 | GFCJ01039445.1 | | LstiOBP4 |  | c48974_g1 | | GFCJ01032227.1 | |
| LstiOR3 | c48813_g0 | GFCJ01032038.1 | LstiIR41a | c56539_g0 | GFCJ01040941.1 | | LstiOBP5 |  | c56490_g0 | | GFCJ01040885.1 | |
| LstiOR4 | c50161_g0 | GFCJ01033565.1 | LstiIR64a | c54099_g0 | GFCJ01038103.1 | | LstiOBP6 |  | c53701_g0 | | GFCJ01037642.1 | |
| LstiOR5 | c57796_g0 | GFCJ01042400.1 | LstiIR68a | c57364_g0 | GFCJ01041901.1 | | LstiOBP7 |  | c51868_g0 | | GFCJ01035537.1 | |
| LstiOR6 | c52421_g0 | GFCJ01036173.1 | LstiIR75d | c59316_g0 | GFCJ01044211.1 | | LstiOBP8 |  | c49392_g0 | | GFCJ01032699.1 | |
| LstiOR7 | c54915_g0 | GFCJ01039041.1 | LstiIR75p | c57651_g0 | GFCJ01042233.1 | | LstiOBP9 |  | c59888_g0 | | GFCJ01044904.1 | |
| LstiOR8 | c53013_g0 | GFCJ01036865.1 | LstiIR75p.1 | c57266_g0 | GFCJ01041785.1 | | LstiOBP10 |  | c52167_g0 | | GFCJ01035886.1 | |
| LstiOR9 | c53531_g0 | GFCJ01037455.1 | LstiIR75q.2 | c59586_g0 | GFCJ01044538.1 | | LstiOBP11 |  | c43276_g0 | | GFCJ01026060.1 | |
| LstiOR10 | c48406_g0 | GFCJ01031578.1 | LstiIR76b | c56375_g0 | GFCJ01040742.1 | | LstiOBP12 |  | c48814_g0 | | GFCJ01032039.1 | |
| LstiOR11 | c55922_g0 | GFCJ01040202.1 | LstiIR87a | c55166_g0 | GFCJ01039334.1 | | LstiOBP13 |  | c47523_g0 | | GFCJ01030602.1 | |
| LstiOR12 | c53849_g0 | GFCJ01037807.1 | LstiIR93a | c56170_g0 | GFCJ01040501.1 | | LstiOBP14 |  | c49381_g0 | | GFCJ01032685.1 | |
| LstiOR13 | c52168_g0 | GFCJ01035887.1 |  |  |  | | LstiOBP15 |  | c51405_g0 | | GFCJ01035004.1 | |
| LstiOR14 | c58276_g0 | GFCJ01042965.1 | LstiGR1 | c50908_g0 | GFCJ01034430.1 | | LstiOBP16 |  | c45457_g0 | | GFCJ01028373.1 | |
| LstiOR15 | c56008_g0 | GFCJ01040307.1 | LstiGR4 | c53093_g0 | GFCJ01036959.1 | | LstiOBP17 |  | c47838_g0 | | GFCJ01030947.1 | |
| LstiOR16 | c52751_g0 | GFCJ01036565.1 | LstiGR5a | c51915_g0 | GFCJ01035589.1 | | LstiOBP18 |  | c57098_g0 | | GFCJ01041598.1 | |
| LstiOR17 | c52003_g0 | GFCJ01035693.1 | LstiGR5b | c52834_g0 | GFCJ01036658.1 | | LstiOBP19 |  | c51039_g0 | | GFCJ01034591.1 | |
| LstiOR18 | c53294_g0 | GFCJ01037184.1 | LstiGR6 | c3705_g0 | GFCJ01020122.1 | | LstiOBP20 |  | c57179_g0 | | GFCJ01041689.1 | |
| LstiOR19 | c53715_g0 | GFCJ01037656.1 | LstiGR7 | c52834_g1 | GFCJ01036658.1 | | LstiOBP21 |  | c45607_g0 | | GFCJ01028536.1 | |
| LstiOR20 | c46193_g0 | GFCJ01029153.1 | LstiGR21a | c49914_g0 | GFCJ01033286.1 | | LstiOBP22 |  | c41600_g0 | | GFCJ01024297.1 | |
| LstiOR21 | c49860_g0 | GFCJ01033224.1 | LstiGR21b | c41631_g0 | GFCJ01024329.1 | | LstiOBP23 |  | c23316_g0 | | GFCJ01011035.1 | |
| LstiOR22 | c53072_g0 | GFCJ01036934.1 | LstiGR45 | c21748_g0 | GFCJ01009794.1 | | LstiOBP24 |  | c65807_g0 | | GFCJ01051049.1 | |
| LstiOR23 | c51775_g0 | GFCJ01035435.1 | LstiGR51 | c4938_g0 | GFCJ01032683.1 | | LstiOBP25 |  | c38508_g0 | | GFCJ01021337.1 | |
| LstiOR24 | c52154_g0 | GFCJ01035869.1 | LstiGR63a | c28880_g0 | GFCJ01014749.1 | | LstiOBP26 |  | c38320_g0 | | GFCJ01021177.1 | |
| LstiOR25 | c52246_g0 | GFCJ01035973.1 | LstiGR63a.1 | c50350_g0 | GFCJ01033788.1 | | LstiOBP27 |  | c73123_g0 | | GFCJ01058528.1 | |
| LstiOR26 | c55854_g1 | GFCJ01040125.1 | LstiGR63a.2 | c47120_g0 | GFCJ01030157.1 | | LstiOBP28 |  | c48290_g0 | | GFCJ01031449.1 | |
| LstiOR27 | c55222_g0 | GFCJ01039400.1 |  |  |  | | LstiOBP29 |  | c48395_g0 | | GFCJ01031566.1 | |
| LstiOR28 | c53069_g0 | GFCJ01036930.1 | LstiCSP1 | c52657_g0 | GFCJ01036455.1 | | LstiOBP30 |  | c86797_g0 | | GFCJ01072083.1 | |
| LstiOR29 | c52605_g0 | GFCJ01036390.1 | LstiCSP2 | c50444_g0 | GFCJ01033899.1 | |  |  |  | |  | |
| LstiOR30 | c52897_g0 | GFCJ01036731.1 | LstiCSP3 | c55235_g0 | GFCJ01039415.1 | |  |  |  | |  | |
| LstiOR31 | c50161_g1 | GFCJ01033566.1 | LstiCSP4 | c56144_g0 | GFCJ01040471.1 | |  |  |  | |  | |
| LstiOR32 | c55203_g0 | GFCJ01039378.1 | LstiCSP5 | c50283_g0 | GFCJ01033706.1 | |  |  |  | |  | |
| LstiOR33 | c50480_g0 | GFCJ01033943.1 | LstiCSP6 | c54133_g0 | GFCJ01038143.1 | |  |  |  | |  | |
| LstiOR34 | c59969_g0 | GFCJ01045010.1 | LstiCSP7 | c48206_g0 | GFCJ01031357.1 | |  |  |  | |  | |
| LstiOR35 | c50674_g0 | GFCJ01034163.1 | LstiCSP8 | c52695_g0 | GFCJ01036499.1 | |  |  |  | |  | |
| LstiOR36 | c55053_g0 | GFCJ01039203.1 | LstiCSP9 | c44870_g0 | GFCJ01027754.1 | |  |  |  | |  | |
| LstiOR37 | c49794_g0 | GFCJ01033146.1 | LstiCSP10 | c54763_g0 | GFCJ01038866.1 | |  |  |  | |  | |
| LstiOR38 | c52410_g0 | GFCJ01036161.1 |  |  |  | |  |  |  | |  | |
| LstiOR39 | c50614_g0 | GFCJ01034097.1 | LstiSNMP1 | c53448_g0 | GFCJ01037359.1 | |  |  |  | |  | |
| LstiOR40 | c49183_g0 | GFCJ01032465.1 | LstiSNMP2 | c55425_g0 | GFCJ01039635.1 | |  |  |  | |  | |
| LstiOR41 | c56510_g0 | GFCJ01040910.1 |  |  |  | |  |  |  | |  | |
| LstiOR42 | c51381_g0 | GFCJ01034977.1 |  |  |  | |  |  |  | |  | |
| LstiOR43 | c47710_g0 | GFCJ01030811.1 |  |  |  | |  |  |  | |  | |
| LstiOR44 | c51607_g0 | GFCJ01035241.1 |  |  |  | |  |  |  | |  | |
| LstiOR45 | c44707_g0 | GFCJ01027581.1 |  |  |  | |  |  |  | |  | |
| LstiOR46 | c45601_g1 | GFCJ01028530.1 |  |  |  | |  |  |  | |  | |
| LstiOR47 | c42299_g0 | GFCJ01025030.1 |  |  |  | |  |  |  | |  | |
| LstiOR48 | c9294_g0 | GFCJ01077814.1 |  |  |  | |  |  |  | |  | |
