# Supplementary material for: Identification of candidate chemosensory genes by transcriptome analysis in Loxostege sticticalis Linnaeus
Source: PLoS One. 2017 Apr 19;12(4):e0174036. doi: 10.1371/journal.pone.0174036 (PMC5396883; doi:10.1371/journal.pone.0174036)
Supplement: S5 Table — (DOC) [file pone.0174036.s007.doc]

**Protein sequences of all identified candidate olfactory genes in the *Loxostege sticticalis***

>LstiPBP1

MTYPKMWTSKILVMMVAACVMTVMVDSSQSVMTSMTKNFIKAYEACAKEYNLPESTGQELINFWKEGYTVTSREAGCAILCLSSKLDLLDPEGKLHHGNTVEFAKQHGSDDAMAHKVVEILHSCEKAAAPNEDMCLVALDVSMCFKKEVHSLNWAPDNELLFEELVGEMSKT

>LstiPBP2

MNSPLIVSISFYLINFSTSFPDEATAKQIMTIVHECEEKFATNEDHCARAMEVSRCFRDHMHRLQWAPSVDVLVGEILVEMA

>LstiPBP3

MGFSVRLLVVLVAVTIYGVNSSQDIIKQMTINFGKALDSCRKELDLPDSINADFYNFWKEGYELSNRQTGCAIMCLSSKLDLVDPEGKLHHGNTHEFAKKHGADDAMAKQLVDLIHKCESDVPDDPDPCLKVLNIAKCFKAEIHKLNWAPSMDLIVAEVLAEV

>LstiGOBP1

MTDVTLGFGQALEQCREESGLSEEKMEEFFHFWSEDFKLEARELGCALRCMSNHFNLITDSNRMHHANAEKFVKSFPNGEVLAKQLVGMLHECEKKHHDEEDNCFRVLHMAACFREACRGASLAPTMEMLLAEFIMQGEN

>LstiOBP1

MMAAMTGRAVLVAAALAALALGARAMDDEMAELAKMLHDNCGEETGVDLGLVDKVNAGADLMPDGKLKCYIKCIMETAGMMSDGEMDVEAVLALLPDDMRRKNEPSLRACGTQKGADDCDTAFLTQVCWQKANKADYFLI

>LstiOBP2

MPYNPKCTFKASIRASSLLYIYGKERKKSSLYSTLTMAKGMDGRFLLLLVFIISACDAMTKQQLKNSGKMLKKSCMGKNQVTEDQIGSIEKGKFIEEKPVMCYIACIYQMTQIVSIV

>LstiOBP3

ISFQELSEEIKEIIQHVHNECVGKTGVAEEDITNCENGIFKEDKKLKCYMFCLMEEANLVEDDGSVDYDMVISIIPEQYQERAKNMIYSCNHLDTPDKDKCQRAFDVHKCSYDKDPDFYFLF

>LstiOBP4

MAKFTILCLGVLAAAISSARALTPEELTKIEGDMLVHVQDCAKKFDVDESDLKKAKEEENIDGVDPCLIGCVFKNIKLVNDKGLYDPDVAIESSKSYLSDDADKAKFAEIAKDCASVNDESVSDGEEGCERSKLLLVCFGKHKHLLMKE

>LstiOBP5

MKFIIKYVLVLAITLVCDGLVDIEKYLKICDRNSVDVNDCLLEAAQEGLAVLANGIQDLDVPSIDPYNQKDLRIEYKNNQIYAKFIAKNIYVEGLKESTVHDARLRADEDRFHLELDLTTPKINVRGQYAGEGRYNSLQIQANGEFITNMTDLVYTWKLDGVPEKN

>LstiOBP6

MIVKKHHCVFVNFFVLILLIDVSFGMTRQQLKNSGKLMKKSCMPKNDVTEEQVGEIEQGKFIEERNVMCYIACVYSMTQVVKNNKLSYDAVIKQVDMMFPPEMKDAVKASAAHCKDISKKYKDICEASYWTAKCMYDFDPKNFVFP

>LstiOBP7

MKTFIVLAVCFVVAQAFTDEQKEKLKKHKTECLSETKADEQLVSKLATGDYKAENDALKKYALCMMIKSELMTKDGKFKKDVALAKVPNPADKPQVEKVIDACLANKGNTPHQTAWNYVKCYYEKDPKHAILQ

>LstiOBP8 DSKMFCRVILLSSVYFLALTPYSINAMTEAQKEMIKQHFEQLGMECIGDNPITEQDINDLRAKKAPSGPGGPCFLACIQRKIGVMDEHGMMQNENALELAKKSIPG

>LstiOBP9

MSIVVVFLSLVPALVKCSGEGNIRLLEEEVATAMKACAVPSEDPKDGSAANQRQRRSEDYPSVDNNDNNTGQNVYSYERRVLNLTDIRDQMYILNATDYDYGGYGAGSAGEKYLLTVPRPASGRSYYGNSSDNANRTRRSEPLLKPESNQCLSQCIFANLQVVDSKGIPREAELWGKVQSSVTSQQSRAALRDQIRACFQELQSDAEDNGCSYSNKLERCLMLRFSDRLKADRSKTQANNQKT

>LstiOBP10

LVKVNNKSTFYNIYYPFRLNIDCAKICSDSVRSHDMFLDLPVRLQLNLGQDVLTDFLYYWQEDRQFTNKQVGCTVICVSKKLNLLDKAGRLSQPDAEAYVKTAGGGNSHFFYLSSTSQPTSTAGQRPPLLFLRCPHPFTYQRP

>LstiOBP11

MFGVIGLFVLMFATCRANVAVSRSDTPQVLCGLIPDKLNSCGHLPTIVSAESAKKCGSSSNSCQRMTCIFQESGWMDGKSVNNAKLSEYLDHFSSEHPDWTAAIQHAKTTCLVPNLPAQGFHLNCPAYDVVTCVFRSFVWNIPPSLWSSSSDCEPVRQYAAACPVCPTDCFSPAIPVGSCNACRALPRSP

>LstiOBP12

MCHSLLCIVIFAVIVLDCNALNCRSEGGPKENELKSVYMTCLKKQDGKNSSDSHGYTEDQDWKETRGQSKFHHRSKWGSGSMGEIDDRMRDRDDRMDDRDDRTNREDRTRSRDDRMGGRNDRMGGRDDMNSRDRMGGRENIMNDRNNMMNRDEDANRYGREPLRGRHDFPQSDEYETDMTRYGYHSTTQSTRRFKRSRRTEINSGQRSQYNPNSRKPSQYEETYKDEERNSSNSSRESDNKACALHCFMEQLEMTDDNGMPDRYLVTHAITKDVKNEDLRDFLQESIEECFQILDNENTEDKCEFSKNLMMCLSEKGRANCDDWKDDLKF

>LstiOBP13

MRPFLFLCLVMAVAGNSHHAQLSQAQKEKVQQYTMQCIKQTGVKPDVLAEAKKGHFSDDEALKKFALCFFQKAGIVDSNGKLNVEAALAKIPSSVNKADAMKLLEECKKKSGKDAADTAFEVFKCYSRGTKTHILV

>LstiOBP

MFHRVLSIILFGLFTCNVKGDFSNELQKKFVGYLGECWQTYELTPKDLEDLKLLKMPDSENVKCYFACVYKKAEMMNDKGEFWEEGVKKTSLEQYGNDDALLKKVNDFIDICKKVNDEPVTDGEKGCERAALMFKCSNEHAPEFGFI

>LstiOBP15

MVRKISALLCCLCVFGISLSDSAISADSEKRCRNPPTAPQKIERVITLCQDEIKLSILREALDVIKEEHTMPEKRRRNKREVPFTHDEKRIAGCLLQCVYRKVKAVDGYGFPTLEGLVGLYSDGVNERGYFMAVLEASRECLMRHHDHFSRTVPMDNGRNCDVSFDIFECISDRIGEYCGNSGL

>LstiOBP16

MLTKALDCTKGNFVSSKELQMMMNHQLPGTKNSDCYIACVFKKVEWLDEKGNYNIEATHKMADKEYADDATKMENAKKLFDHCKTVNDEAVTDGEAGCDRGHYLAKCLIDNAPKMGFDLSKY

>LstiOBP17 MYLKRTLVVLCTVLVLGSAAFVDNIPKCGAKDTDCHRQSFQYVIREGSKTGIPEANIAPFDPLELKQELNIPIRDIVQLHFGDGVVKGLSKCVINDFVTNVEQGKASLDITCNFTVKGHYKANSSSPVIKSLLGGESVHGDGRVKIKIVKLNLKLDFDFIVDKRNGDTYFKRKGNNIKFKYDVLGQVMFAADGLYLGDRDASELLTNMLNQNWKLVMASVGDDIMKDSMGAVEEFVRNFFENVPTKYFITDDLTPYASN

>LstiOBP18

MCYIACVYKTIQVVKNEKIDRDLVFKQVDILYPADMKAAVKSAVEQCYGVQAKYNDLCEAAYYAAKCLYETDPPNFVFP

>LstiOBP19

MFKLILSCIAVAVCMKSVNSLTPDQKAAVQAKLLTSGLHCIRDHPLNLDEIKMLRDKKLPEGENAKCFTACLFKQIGIMDDMGKLNAANAVKSAEEVFKSSDKHLEKSKQIIQECISVNDAPTSDGAKGCDRAKLAFSCLIEGADKHGLHITF

>LstiOBP20

MRFLCLCLLLQSVLYSEATFGTPYLVNSRLCQNWTCVNSKLGLPNSLPPRDQYTQILKTLLPSGAWQDVVERVLDSCYGTRPRNYVGTCPGQALLLCTVDNLIENCPEESWRKDDGCYPVTSLAGTKN

>LstiOBP21

MFKSGVYIALFACLLEMAMSLTEEHKLKLQATFETVGEKCAKENNITEEDIAAFKERKFPDGQEAACFSACVLKNIGLIDDEGQLSHDLAVENAKAVFGEGDEIKAIEEFIETCKDSVADGADACERAKLVFKCFVEHSEKFNF

>LstiOBP22

MRKFFWIIIVFVSGVKTDLLHQDRSKGATLKPISACCDIPELGDEKPLSECSNPKLPGPCNDVHCVFEKSGFLVDKNTLNKDAYRRHLRQWAENHKDWSDAIERAITDCVDKDLRQYLDYPCRAYDVFTCTGIAMLKKCPKDAWKC

>LstiOBP23

PLLFYLDQELGSVRCRDKTVMEDFIDNCLPKKASAPHEIAWNYTKCYHTQAKEPHNKKKKLDYLNIFY

>LstiOBP24

FVSNVQGEDGKNHWNIKNWTYTYDLKGKSNVYFENLFNKESFLGQTAQEMVASNGNAIIHDIGKPIITSIVTEIVHNVQRFFKAVPSEDLSLD

>LstiOBP25

MDGQLLVLKLQGNGNAHFKLRNTQFNVICEHNEKIGKDGKLHYNIKNTKYTYDLKGKVDIELENLLQGNEVLAAAAREVFTTNANIIADEIAPKFIKAVVDKIVKNVNNFFHAAAVEDIEIV

>LstiOBP26

MIQIVFLVLAFISGYSHALTEEEIKAEFTKLVMKCLKDHPVDMSELTNLQKLVVPKKNDVKCLLACAYKLDGIMNAKGLYDLDHAYKVAELTKNGDEKRLENGRKMADECVKINDIEVSDGEKGCERAGLMFKCAIENAPKFGFKL

>LstiOBP27

MLLPFVTHARISVMYAHDKLSDIVAEQCFNEMFPKTKHVEVQESDEPCLIFCVMKKLGIMSPNGAINLETYRKRVLMAHQHDQRTLVSDFGSSCVENAEATQHKQDVCKKAKVFND

>LstiOBP28

MTWVLAVALLAVFGAVQSASTGCKNCISLGKEEKAMFRAHSDACLPQSEVDPKLVEAMLNGELTDDPALKRHVYCVLLKCKVISKDGKLQKTAVLGKMANRADGKNATKVLEGCAEQHGDTPEEIAWNLFRCGYDKKAVLFEYMPTNIGNSEIDNNS

>LstiOBP29

MIKLLFIVLLSISGSSHAMTEAEIKADFIKLVMKCLKDHPVEMTELIKLQSLEVPKKPEVKCLLACAYKLDGLMTEKGLYNIEHAYKVAEVTKNGDEKRLENGKKIADICVKVNENEVSDGEKGCERAGMVFKCVVENAPKFGFKI

>LstiOBP30

LFCLFASICAVIGDHEKDSPIMAMVHKTLVITAHSCMDQINATETDLEYLRHDPPYPEKASCIIKCLLEK

>LstiCSP1

MKSLVLVALSLLVAVAWARPGATYTDKWDHINVDEILESQRLLRGYVDCLLDKGRCTPDGKALKETLPDALEHDCSKCTAKQKESSEKVIRHLINKQPDFWKELSTKYDPENIYQEKYKDKIEEVKSKN

>LstiCSP2

VFIKTTFSHMVIINVPANLPDAIQNDCKKCSDRQREGADQVMEYIIDHRPDDWVKLEKMYNSDGSYKKKYLDRKEAKNKSATTSGEKSEENVSKSKESQD

>LstiCSP3

MKTIVALCALMAVALARPEETYSDTWDNFNAQELVDNVRLLKNYGKCFLDQGPCTSEGQDFKKRIPEALKTDCGKCSPKQRELIRTVVKGFQAKLPEVWAELVKKHDPEGTYKDSFEAFLNSNN

>LstiCSP4

MKTFILICLSALVVAAAADKFDDLMNVDFEKLLGDEESRKQLVGCLMDELPCGEYQSYRDLIPDLIATNCGKCTPEQKKRHEEVNKFILEKYPTEYNAVVNKYRPKAE

>LstiCSP5

TIQFYVIFQSKYIEKNLRMRRFILLSVLALVALSLAEEEKEKKEEKAEEKYTDRFDDINFEEILANRRLLVPYLKCVLDKGRCTPEGKELKVHIQDAMQTACAKCTDKQKVGARRVVNHIREKEQEYWEELLSKYDPKGEYKSIYEPFLAGKE

>LstiCSP6

MNFLEEHHLQKIFIMRAIVLLSCLVMVYAADKYSSKYDNFDVETLISNDRLLKAYINCFLEKGRCTPEGADFRKALPEAVETTCAKCTEKQKNNIRKVIRAIQQKHPKQWDELVNKTDPSGKHRADFDKFIQGSS

CFLDINDCDAVAADFKKDIPEAFQQACAKCTDAQKHIFKKFIAGLKEKLPHDYEAFMKKYDPDSKYYPALEKVINV

>LstiCSP7

MKFLLLACLLVCAGVSLARRVPRYTDIYDSIDIKTIVANRRLLLPYVLCVLGEGKCTAPGRELRSHIKEALETRCAKCTEAQQRGSRLVIAHLINHEPEYWAKLTAKYDPAGKFAKMYERELKEQV

>LstiCSP8

MQIALVLVMLAACAYAAETPRPQVSDTALEDALNDKRFIQRQLKCALGEAPCDPIGKRLKTLAPLVLRGACPQCSPQETKQIQRTLSYVQRNYPQQWAKIVRQYAG

>LstiCSP9 MKFIVVLAVIVGLAMADEKYTSENDNFDVDALVANIDELKKFSGCFLDINDCDAVAADFKKDIPEAFQQACAKCTDAQKHIFKKFIAGLKEKLPHDYEAFMKKYDPDSKYYPALEKVINV

>LstiCSP10

MQKLILLALVCSLGWGVVMAAPQMTDSQLDQTLADRKTMERHLKCALQEGPCDPVGRRLKTLAPLVLRGACWQCSPQETRQIRRTLAYVQRNYPWEWSKIIRQYG

>LstiSNMP1

TVLLAAPLRTSVSEWWSRSSFKMHLQKPLKIGLGMMSAGLFGIMFGWVLFPVILKSQLKKEMALSKKTDVRGMWEKIPFALDFKVYLFNYTNPEEVQKGGIPIVKEVGPYHFDEWKEKVEVEDHEEDDTITYKKRDYFYFRPDLSGPGLTGEETIVIPHILMLSMATIVHNDKPAMLNMLGKALNGIFDEPKDIFLRVKVLDLLFRGMIVNCARTEFAPKAVCTALKKEAANGLTFEPNNQYRFSLFGLRNGTIDPHVVTVRRGIKNVMDVGKVIAVDGKPNQDVWRDKCNEYQGTDGTVFPPFLTEKDNLESFSGDLCRSFKPWYQKKTSYKGIKTNRYIANIGDFANDPDLQCYCDSPDKCPPKGVMDLMKCMKAPMYATLPHFLDCDPQLLKNVKGLSPDVNEHGIVIDFEPISGTPMVAMQRVQFNMMLLKADKLDLIKELPGTLTPIFWIEEGLSLNKTFVKMLKHQLFIP

>LstiSNMP2

VLFLKVKKTFLKMLGKRSKMFFGISLGALVVSVILAAWGFPKIVSKQIQKNVQIDNSSAMFEKWRKMPMPLTFNIYVFNVTNAEDVNNGAKPKLQEIGPYSYKEYREKTILGYGDNDTVSYMLQKTFVFDQEASGGLSEDDEVTVIHFSYMAAILTVNDMMPSISGVVNGALEQFFSNLTDPFLRVKVRDLFFDGIYLNCAGNHSALGLVCGKLKTDSPPTMRPAEDGKGYYWSMFSHMNRTPSGPYEMIRGRDNVKELGHIVSYKGKRFMKNWSNDQYCGMINGSDSSIFPPIDENDVPDKLYTFEPEVCR

>LstiIR1

MWFLWLLLSFTIQQCTPQFIFSEKKDISYQIAGIFLSDAQTQMLAFNETMASAGLDQFHLSPAILQPTSKDSLAIWNELCSNRNLRPIAIVGPQDPKWDGIVRDQCALANIPHVQASWQPLDADLELNDEEEEEVSEGENEEEQKEVSFKKLSINFYPNSEEISLAYAALLKYYHWENFAVLYEDEYGLLRIQKILAEHTTNFPVIVRKLEPEMDNRNVFKELSKHQLNRIMLDCNASRILDYMKQASDLKMVNYYQHYILVTMEAYIVAEQLTHYHSNITWLSLTEYDKLKDAQHVLAPMVGKWITRQMIPPPPVTDFPNEALIMNDIANHLLRAMQTIKDKITYFKPRAPICDADAQPWRYGALFQNAILTTPSYGVTGNIAFDEKGRRFNYTLYVNEIHVNKLQTIGTWASTNGTEILEDRPDSGTLDQQQSSKHFVVISTKAKPWFYDKTPCVGEEECENDDGLKYEGFSVDLIKEIFRFLREEKFNYTFEFMDGGDKKAGTVDETTKKWTGLIGDLLDNVADLAVCDITITEERKKMVDFSVPFMSLGISILFTQKKDPELELFSFLNPYTFEVWMYTATAYCVVSVILFICAR

>LstiIR7d.2

MLDWMQKHQFDNTGRFIVTCQSERPEDCDETKAVDIFWNHKIINVVFMKQTPDEEVNGYTYYHPDENCRSSQPVKIKTLRCTKYNNTTPCLGIFPKKMKNLHWCPIIVSTFKQMPYMSITDEGVPYGADGDLLLLIAEGLNATLKVMTPRRGFGWGNLDADGVWVGSLADVFDDVANFSMTSAAVTLSRFTYFQMSTAYYTSNVVWITRPAEKKPASLKLFYPFQTLSQIALAFSFIFVGLCVWLVNSKYWPWHNSTEEASKLSSVLFYSWMICMGLPSTKLPAKKEFICLVLIWIWYCFLIRTFYQVYLINSLQGEFYSDEIDTIEDAYFANYSFGGGPALRDYFIDHPLVYDNWKNLNASDIIPTLINLTEKKFVLATNQATTESIIKEHNLIVHILPQKVINSPSVIFSKKFSPLVEPLNIILRRLLESGFTDKLYKNYATQNTWKSDNPEEPITLEHYTGCYVILVVGWVVSTLIFIIELFYTKLQKCKYYIIKMKPRLGCQII

>LstiIR7d.3

NEHEDTMVVKILFLIGVLVGCGFAEEYRIGGIFYEDAEDLKVAFALSAKMLNFTPSIREVSKRGEILEVRQQVCSLAEESVIGIIDGIGGRGSEIIQSLCDSMELPHVLIRYDFTYSSDWSLLNLYPSPIVYNKVVEQMVLRKEWKNFTIMYIRGRSLFRLHNLLQMGNDTDNYAIGVRELSGKDYRDVLINAKENGYTNFVVDCPSRHLEQLLLQAQQVGLMADEHSYIILSPDLFTLDMERYRYGGVNMTGFRLMDLKASENLWEFTEHYNAETGKTMLPEQLKTELLLVYDAIQVFAGALQKLKGVEAQPLNCENYDAWMYGSSLLNFMKTNKVEGLTRTLLFDGLGERTDVIFDVLELTSSGNQTIGTWKHNKLDIQRPFVPDAELTENTALKNKTLKVLITTTGPYGYLKHSDQKLEGNDRYEGFSIDLIEKISEILGFSYEFEVEEDNGKLVGGKWTGMPRKLMDDEADLAICDFTITALRQSAIDFSTPFMSLGIGILYKEPSKQPPEMFSFMAVFSKEVWYYMMLIQMMLGGVMIFVGRISHKEWQNPVPCIEQPEELSNQFSFANSVWLIIGSVMQQGSEIAPIAVAPRMITSIWWFFTMVMVASYVGTLVAFLTVEKNVLPFQNVEELFRHKSISYGAKKGGSTLDFFMQSKNEIYQRMYQKMTSKGWLVGNNADGVNLVENSTYAFFMESTSIEYTMERHCDLLQIGGLLDQKSYGIGMKKNSTLKVYIDRALLILKERGEIQKLKDIWWKEKRGGGKCGEKHAVEEKQLGMKNMLGAFVVLGVGCGLGVIISTLDMLWGVFKRSVKYGTTFKYELVEELKFALKFSGDVKPVKRPQKTMEGSFEALAEAEGKDEVKSLKSHQSGRSTSTHKTHHSHSSRHSSRGLSVAFARQREYS

>LstiIR7g LFIMKPKIEICLQWLLMCCCTFVHGDRTIGAIFDDGTFLMEAAFSVAVTAASEGQENPFEAKVIRTSPGDLLEAEQAMCSLLENHIFGVFGPKTKGLIDHIQSIADVLELPQILTEPVETQNRNWSAVNLYPNHIAYSQVFADIVEMKGWEEFTIIYESSEL

>LstiIR8a

KMDVPLLFLLIFLINLGCIVSELSLRFVFIIEVHDSDLAHQIGRALKVAEDQRTGVRVSDAVVQLDRENEEESYRRLCSALSKGTSLVVDLSWAPWDMAQQLCSDAGLPLVRAALGSQQLLAALDQYLESRNATDAAILMESEGEVDKTLYELLGRSNVRLWVHAGLTRDSAKALKSMRPEPSFYVIVGESGFVMDTYRRAVKEKLVRRDYRWNLVLTDYSGDTIDVAQLVLPTMILHVDQVECCRLLGLREECSCPSDLKRKQYILNALLIYITETFSKLERELPVVSTRVDCDNVQGSEMNGTRDRILRQFSEDTEMNNDTIFFWDDERSGLFLRSNFILSTYKPDAGLETVATWSANEEYKLLPGATLEPLKLFFRIGTSPAVPWTLPKIDPETGEPEVNEDGQPVYEGYCIDLIAKLAETMEFDYEIITPKSGNFGKKLPNGSWDGVVGDLMRGETDLAVAALTMTAEREEVIDFVAPYFEQTGILIAIRKPIRKTSLFKFMTVLRTEVWLSIVAALVLTGLMIWLLDKYSPYSARNNPQAYPYPCREFTLKESFWFALTSFTPQGGGEAPKALSGRTLVAAYWLFVVLMLATFTANLAAFLTVERMQTPVSSLEQLARQSRINYTVVEGSTIHQYFINMKFAEDTLYRVWKEITLNATSDQAQYRVWDYPIREQYGHILLAINASGPVPDAKTGFEQVNEHTDADFAFIHDSAEIKYEVTRNCNLTEVGEVFAEQPYAIAVQQGSRLQEHLSRALLDLQKERFLEQLASKYWNESARQACPDADESEGITLESLGGVFIATLFGLGLAMITLAWEVFYYKRKEKNKVQGFDSKIEKAAFVDTKKKLEKVGVRLRKKNKSGKVGKVDVIGKGKGVTIGDSFKPASEKMGVSYISVYPKGEYRP

>LstiIR21a

FRGGDAVSKEVEMGRADIAVAGMYLTSDRIKEMDMSFSHSQDCAVFITLMSTALPRYRAILGPFHWHVWVALTFTYLIGILPLAFSDKHTLRHLLHNSGEIENMFWYVFGTFTNCFTFVGKNSWSKTTKITTRLLIGWYWIFTIIITSCYTGSIIAFVTLPIFPETVDTIEQLIAGFYRVGTLDHGGWERWFFNSSDPKTNKLFKKLELVPNVESGIRNTTKAFFWPYAFLGSQAELEYIVQANFTVTKSKRAMLHISHECFVPFGVSMGFPTNSLYSAKLSSDLRRMFQSGIIDKIVDEVRWEMQRSSTGKLLSAGSESLKITNAKEKGLTLEDTQGMFLLLGAGFLMGASALVSEWMGGITRRCRIGKKKPSSANSKQELISTPEIENEVKVITDATESRINFDNRSCSSSAGSRDTLESQVINVTEESIEVHESLDAARWDSRRSSSVDLDREVQEIFEKDLRRRKIVTDDIDEVIDEKREPTASHGAFGDRLK

>LstiIR25a

MGKKYMRLLLKVLLLFSFVRAAIFQTTQNINVLLINEENNALAEKAFEVAKEYVRRNPSLGLAVDPVIVVGNRSDAKAFLENVCRKYNDMLSAKKTPHVVLDFTMTGVGSETIKSFTAALALPTISGSFGQAGDLRQWRSLDANQTRFLLQVMPPADILPESIRAIVTKQDITNAAIIFDELFVMDHKYKSLLQNIPTRHVITPVKSYNKDEIKTQLRSLRELDIVNFFVVGSLRTIKNVLDAADENQYFGRKTAWFALSLDKGDITCGCKDATIVYMRPTPDAKSRDRLGKIKTTYSMNGEPEITSAFYFDLSLRTFLAVKSLLDSGKWPNDMKYITCDDYDGKNTPNRTLDLKAAFQEIKETPAYAPFYIPEDDPLNGRSYMEFNTDLTAVTVKDGASIGSRVLGSWKAGLSNPLSLTDPENMSDYSAQLVYRVVTVEQEPFIIRDDEAPKGFKGYCIDLIEEIRQIVKFDYEIVVSPDGNFGTMDENGNWNGIIKELIEKRADIGLTSLSVMAERENVVDFTVPYYDLVGITIMMKLPRTATSLFKFLTVLENDVWLSILAAYFFTSFLMWVFDKWSPYSYQNNREKYKDDEEKREFTLKECLWFCMTSLTPQGGGEAPKNLSGRLLAATWWLFGFIIIASYTANLAAFLTVSRLDTPIESLDDLSKQYKIQYAPLNGSAAMTYFERMAHIEVKFYEIWKEMSLNDSLSDVERAKLAVWDYPVSDKYSKMWQAMKEAGLPNSIEEALQRVRDSKSSSEGFAWLGDATDVRYHVLTSCDLQMVGDEFSRKPYAIAVQQGSPLKDQFNNAILQLLNKRKLEKLKENWWTNNPKSMKCEKQDDQSDGISIQNIGGVFIVIFMGIGLACITLGVEYWWYKIRKRSTIGDVTQVEPAKSSRNHTDIKGEGFTFRSRNFGLSNLKPKF

>LstiIR40a

MYNLLLGESNEMKLLPFYLFLNTAHCFIDIQDIVSETMTKLPKDFAVAIKDIAEGLPAKTITVVRGESTKIRSQDIFQLLCLLSEHNIPVINLDITTKQSKDKYYSFVKKALDVSEERTSLILCEPYECENILTELTDNNLIHRTILYIFYWPYGKVSDQFLNTMKEAMRVAVLTNPRESVFRVYYNQATPDRLHHLSLVNWWSGSLYKSPVLPPAEKVYQDFKGRIFDVPVLHAPPWHFVKYNNDSTVNVTGGRDDKLLSLLSKKLNFRYRYYDPPDRSQGSSISGNGTFKGTLGLIWKRKADFFIGDVTMTWERLQAVEFSFLTLADSGAFLTHAPAKLSETLAIIRPFRWEVWPLVCATVLVTGPALWVVIAAPSLWQKRQRDQLRLLNNCCWFTTTLFLRQSSSKEPSKTHKARLVSVLVSLGATYVIGDMYSANLTSLLARPARERPIGTLPALEEAMRERGYELVVERHSSSLAILENGTGVYGRLARLMRRQRIQRVRSVEVGVRLVLTRRHVAILGGRETLYYDTERFGSHNFHLSEKLYTRYSAIALQIGCPFLETFNNVVMTLFEAGILAKMTTDEYKNLPEQSRRSEPVTESDKPNNEITGDSPSASQSGTTQGESTKALEPVSLRMLRGAFCLLGIGHLLAAIALGVEIQIHRRSKNFIKIMEPNGGKNMPRMRALKKASKCVRQGVRRVVRAVCRSIDRALGPGVQ

>LstiIR41a

MIHAVRMLPNPISFLPIEILLSTIFHQYLNSSYCLSLVSDSPLNIPIKNSFTYISPEDGELFVNQLLDVSEMGCSDYIVRMQDPKKFMAAFEKVNHLGNVRRSDRKIVFLPPEEDNKTRIDLLEVLALKESGFVANILLVLPTTELSLCSYYDLVTHKYVGPDDQVDEPYYLDRWNSCSSKFEKNANLFPHDMSNLQGKTVKVACFTYKPYALLDLDPSKEPLGRDGTEVRIVDEFCRWVNCTIRIVRDDEHEWGEVYENHTGVGVLGNVVEDRADLGITALYSWYEEFLVLDFSVSGIRTAITCVAPSPRLLASWEMPLLPFSWYMWLALFVTFIYSSLALTVAKGFSMDKVFLTTFGMMITQSQADVGASWRVRSITGWLLLTGLVLDNAYGGGLASVFTVPKYEKPINTVQDIVDRGIDWGATHDAWVFS

>LstiIR64a

MDFNYFLNFISIAEISLIIDLLKFKDIGNVVNINCDTQKSIFLHKMFNDNTIHAAYWNINSDKNEIPNSYRKTGIILNASCSNWAQAFNNISDYSIFKNQFIWFVLTEDFLSTVRSLSSYPIEIDSDVTVVHKTDGFYKLYEVYYRNYSNGVLSIREIGYWDTFLRVNSSNRNDLHGLVMRCPVVVTDKVVQETFEEYLSKPKKNQVDSLHKLKFFALLNYIRDMYNISYDIQRTNSWGYNTRNGSFDGVVGALHRKEADIGGSPLFFRADRAELIDYIAETWQSRQCFILRHPKHPGGFYTIYTRPLTARVWCCILVMLVFSAVILCLMLKTKIPPSSDDSADSSFSLALLFIWSAICQQGMSVNRSSTSVKMVVLVTFVYAVTLYQYYNATVVSTLLRESPKNIRTLEDLLQSNLKAGAENVLYAKDYFKRTTDPVALRMYHKKIAPSHQFNFYAPARGMSLVKKGGFAFHVDSVVAYRIMRKTFTEREICEAHEVLLYPPQKMGMVIRKGSPYKEHFTYGIRKIYESGLMHRLKSVWDEPKPACVRTPDSSVFSVSIIEFSTALFALVAGNVLAILVLLCEIVMHRCGTNNHIAFFTLGSVLVL

>LstiIR68a

MFKVVVLCILLANTAADISPILRSLNERKDLELVLVDLINGLSRREDITCVAFICDAVYLNVFEGYLFKRTDAAPYVMIVVEDYEDLLSPNFDTLESLRETRKDGCNVYVILLANGLQTARLLRFGDRYRILDTRAKYILLHDYRLFHSDLHYLWKRIINVIFLRYHSKINGVRKSKAWFDLSTVPFPNPIKSVFVSRRVDIWNNGRFHYNRTLFADKTSNLNNETLNVVYLDHVPSVVVTKTNDTSKVGGVEIEILNTLAEKMNFLPKLYQPINADLHKWGQKQANGSFSGLLGEMVNGQADVALGNLQYNPYHLELTDLSIPYTTQCFTFITPEASTDNSWKTLILPFKLNMWIAVLLVLLLSGTIFFGLARYYMHLQEFKKTHDKRKNITDKQKMQIEELDSDEKPAGLYLFGEIINSILYTYGMLLVVSLPRLPTGWSIRLLTGWYWLYCILLVVSYRASMTAILANPAPRVTIDTLKELVESKIACGGWGMETKKLFEDSADDIKTIGQRFEIINDPFEAANKVAKGAYAYYDNQDFLKYIRVKRKNVEMSIESEMVNGTSNLTDASSDVGIERNLHIMSDCVVNTPISIGFHKNSPLKPLADIYMRRIVEVGLVEKWLNDAMHPIKSLETNEEEIKALMNLKKLYGAFIALAIGYLISCIGLIGELIHWHLIVKRDPKFDKYALDLYYMNKNKKQ

>LstiIR75d

MDTISLIPAFFLSKNIYFLTTFLCWNSEELHKLWRLGQQQGLRVRAMAAGPATPPLPPDDLHREGVVLDLACPYADHIIQAASETRGFNYRYAWLLLHNSSFDATSLDSVLSGSVILPDADVTFASDDKLLDVYRIKADQPLLATTLGVVRNSTRRDLEQMWGVLKSTVSRRKNLNNVFLKGATIITQPQNFKGWNDLTVRHIDTFPKLMYPLLMHCAEDLNFRLNLLQVELYGDERNGSFDGLAGMLQRRDIEVGVTTLFMRHDRLNVMHFCSETLELKGAFIFRQPPQSSVNNVFLLPFSRGVWAASALVFTAAGGLLAALSRPRWLRDADPDLVQLSAAEAFTFAVGTICQQGCYVNPHAVSVRMLMFFTLLASLFTFTSYSAKIVAILQTPSDAIQTIDDLTHSPMALGVQESTYKRVYFAESDDPATQRLYRRKLLPQGERAYLSIVDGIARVRNGLFAFQVEESSGYDVISKTFTEQEKCGLKQIQAFKLPMVAVPILKHSGYRELFAARLRWQRETGIMDRERRVWMASKPRCDSDGGGFLSVRLSDVLPAVQVLIYGMLLAAIQLFAEIALHRATERIKRKNKLRGKRE

>LstiIR75p

ALSANRVAFFEPIKFSGRVMTWVFFAALMLLYAAYSANIVVLLQAPSNSIRTLAQLAASTVTLAANDVDYNHFVFSLYKDPDRVRIYKRVDPEKGKGKGQFYDINEGVERIRQGLFAFHSIVEPVYRRIEETFLEMEKCDLAEVDFMNGFDPFIPVKKDSPYLELMRVAFKQIREAGVQSALVRRVHVPKPRCASEVSAFSSVGVRDLKPVLLFMLYGIAASVAIAVIEILIFKLHRQRRFQLRR

>LstiIR75p.1

HTWGYRDKNGNWQGIVDNLIKKKADLGTLTIFTQERMQVVDYIAMVGSTAVRFVFREPPLSYISNIFTLPFTGAVWLAIVVCVLGCSVFLYITSKWEATMSMHQFQLDGSWADVIILIIGAVLQQGCTLEPRYAAGRSVTLLLFLALTILYAAYSANIVVLLRAPSSSVRTLPDLLNSPLKLGASDFEYNRYFFKKLKDPIRKSIYDKKIAPKGKKPNYYSMEEGVEKIRKGLFAFHMELNPGYRLIQETYHEDEKCDLVEIDYINEIDPWLPGQKRSPFKDLFKINFIKIRESGIQACIHHRLHVPKPKCSGTVSTFSSVGITDMYPAMLATLYGMLLAPAVLLLEIAYHRLTVLRKRKIKTRKWKSKLDHF

>LstiIR75q.2

MKVSITLVSLFFLNICNAKTDSVVLVVADVIRAMEKPSTVVATLCWQTNKKVDFYNAVTSSNDRSRVATARLVDMKYVKRDYGQDQHIVFVADLSCPNISDYFVMKKEEQYFRSPFRWILISRNENDDIVPNEISHIDLLPDSEVIVLRQVGDDSYDLHFIYKISPGSYWRTQFYGTWNHEKRFVKSNQQIVESTALQRLDLLGYEMSICYVLTDKDSINHLTDEVNDHIDTITKVNFPTTNHLLDFLNATRKFIFADTWGYRVNGSWNGMTGYLLREEVEIGGSPMFFTSERISVVDYIASPTPTRSKFVFQQPKLSYENNLFLLSFRTSVWYSSAGLIFLLLLALFAVAAWEWKKNAHDIYRKEDSGTLRASFDDVIILIFGAICQQGSPVELKGSLGRVVMLILFLALMFLYTSYSANIVALLQSSSSQIRTLDDLLHSRLKFGVHDTVFNRYYFSTATEPVRKAIYEKKVAPPGTTPRFMSMEEGVKKMRKGLFAFHMETGVGYKFVGKYFNEGEKCGLREIQYLQVIDPWLAVRKNTPYKEMFKIGMKRIQEHGLQARENRLLYEKRPKCSGRESNFVSVSMVDCYPALLILSYGCLVSVIFLVFEFLFHQKQTIIQKLTHCNRVKTSRSAFTN

>LstiIR76b

MATGIELIISSICNATFCQPVYDNPLLEKQASSSIDQYRDLIKEINGKHLKIGTYNNRPISWVERGEDGALIGRGVSFVLVDILQKRFNFTYEVVVPEKNFEIGGTKPEDSLIGLVNNSLVDMAAAFIPKLTRFHEMVRFSYDLDEGVWVMMLSRPKESAAGSGLLAPFNNAVWYLILVAVLSYGPCITLLTKLRSKLVPDGEKYIPMSPSFWFVYGAFIKQGTNLAPEANTTRVLFTTWWIFIILLSAFYTANLTAFLTLSKFTLDIESPQDLFKKNTRWVSAEGGAVQYVVSSPNEDIYYLSRMIATGRAEFRSMNSLYEFLPLVSGGAVLVEERIGIDELMYGDYQQKAREGVAEAERCTYVVAPNLFMSKLRGFAYPKNSQLAPLFDTVLTYVLQAGIVDYLEHRDLPSTKICPLDLQSKDRQLLNSDLYMTYMIMVTGLSAAVAVFIGEIMIKRYVIKDSKPKKPKRKKTKYEKNRHINSYDDTRPPPYDSIFGRSPKIKVNDTTKTKIINGREYLVIDAANGDTRLIPLRTPSAFLFRLDR

>LstiIR87a

MCTKIFLQHLFFALYVSAAINENPLLTTTGNSEQTAKTAECVLKLSAKYFVEKKALSGSIVIININSYVSTTQVLLLQTIHGGIKYSVMVKDSFYPHANASHFPEKAKNYMLILEEKSELTRNILQLNKLPTWNPLAKAIVFYQLNKTEDAEETAIEFINELRHYKLFKSIIFIYSPEEKEVVSYTWVPYSDMNCGGKCDSVYILDKCKDNVIYQLATQKEMFPSDMKGCPLVAYAIVSEPYVLPPEMKLTNTSYNDAYVFQKGGEINLVKIITQFTNMSLIMRTSELPENWGTIYWNGTATGAYEVLRNDSADLVIGNIEVTRTIRRWFHPTVSYTQDEMTWCVPKAGQASTWNNLVIIFQWSTWVATFGSLVIMGLVFHYMYYRENGKKVTKWPTNSMLMTFSMLLGWGASFEPKSATFRILIFGWLCFSVNMGISYESFLRSFLMHPRFEKQISTETDLIQSRIPLGGREIYRSYFETNNASSFYLYRKYNSTTFAEGIRRAAKDRNFAVVSSRRQAAYADQKLGKGKPLIYCFPESNNLYKYGVVLLARKWFPMIERCNTIIRSVSENGLIDKWNRELLIHIGNGEGTSEIEPLSIQHLLGAFIFVGIMYAASILVFIFEVSIGVYEKWKIRKNLQPDNRHVRFHFAK

>LstiIR93a

MRIWLLVFCIVQVSGEDFPSLITANASIAVVLDREFLGEQYQAILDELKDYIKELARVELKHGGVVVHYYSWTSISLMKGFLAVFSVASCEDTWSLFSRTEKEELLLFALTEVDCPRLPTDSAITVTNVVPGEELPQILLDMRTEMAFKWKSAVILHDDTLSRDIVSRVVQSLIMQIDKGASTSPVSVVVYKMKHEINEYLRRKEIRRVLSKLPVKHIGENFMAIVTTEVMTTMSEIARDLVMTNTHAQWLYIISETDAQNGNLSSLINALYEGENVAFIYNVTDNGPECKNGLMCYCQEMMNAFISALDAAVQDEFDVAAQVSDEEWEVIRPNKVQRRSMLLKHMQQHIATKSSCGNCSTWRALAADTWGATYRSYGDADLVAKDTTNGTMTGAIEHVDLLQVGYWRPIDALRLDDVLFPHVEHGFRGKDLPIITYHNPPWTILQVNESGSVVSYTGLIFDIVNQLAKNKNFTVKVILPSHVKHLVANDTSADMSHSQDAMLTLSAVAKGQVAIAAVAFTVLSDPPPGINYTVAVSTQTYCFMIARPRELSRALLFLLPFTTDTWLCLGLGVILMGPTLYIIHRLSPYYEAMEITRQGGLSTIHNCLWYVYGALLQQGGMYLPRADSGRLVVGTWWLVVLVVVTTYSGNLVAFLTFPKQEIPVTTIEELLKNQQIYTWSIQKGSYLELELKNSDEPKYTALLKGAELSNTGGTMESNLSSWKKQLIRIREQRHVIFDWKLRLSYLMRNEHKLTDRCDFSLSVDEFIDEQLAMVLPAGSPYLPVINKEINRMQKAGLISKWLYAYLPKRDRCWKTSSISQEVNNHTVNLSDMQGSFFVLFLGFFSASFVLFLEWFCNRRKRRSEEVIIKPYVE

>LstiGR1

MGQLKKNLGFWIPIKKNKVHVTKFQTDGKQATFQKSLRMTLIIGQMFSLIPVTGIFSNSASNVRFVLKSWKCLYSTLSFCGQIFMTVMCVHKVVHTTTSLNGNAPVIFYGTTCITMIMFFQVGRSWPSLVRHIARNEELDPNFDPGLSYKCNVTCAIVLMLALLEHILSLLSAFAGAMVCHPDKAFYEGFVTHFYPWVFNVLPYSAVLGATTQFLHFQSTFIWNFSDLFVICMSYYLTSRLEHINGKLLAAQGKYLPEIFWKTTREDYCRATQLVRRVDEVISGIVFISFANNLFFICLQLFNTLEDGIKGTGECSSRSKSTPSNLLGGYEAATYFLFSLVYLISRSVAVSLIASQVNAASTVPAPVLYDVPSPVYCVEVQRFLDQVNGEHVALSGLQFFSVTKGLLLTVAGTIVTYELVMFQFTTSQPDDVSTNTGFNTTGAFSNISSTISYFIQ

>LstiGR4

MGVETSNEERGDVTPAPTLRSTEPTRSVVGGAHAFILRISSFFGLAPLRFESRANGFTVSISSVMCIYSFILVSILILLTIYGLVAEINAGVKLSVRMSSRMSQVVSTCDVLVVVVTAAVGVYGAPARMRKMLKLMDRIASVDNTLSGQYSAVMERKLSAILLALLIFFSLLIVDDFCFYAMQAKKVDREWEIVMNYIGFYLLWYVVMILELQFAFTALSVRARFRALNDVLALTARSIAVPVEKARKPTPLNIFAIRVTPTDLQRSDDISLLMTSTPKKRETVIVRRSVSGESRLLVSPSEAIYGLASLHGTLCELVHRIDDSYGIPLVVILISTLLHLIVTPYFLIVEIIVSAHRVHFLVLQFLWCATHLLRMFVVVEPSHYTIMEGKRTEGLVCRLMTSGPSAGPLPSRLELFSRQLMLRSVSYSPMGIC

>LstiGR5a

MGSSRVHFTGEDKMSDSKTVGAQNKLILPDQPYHDGFLETMSKTFHWARLFGIMGRESRRWNVWAIVLLITLLVIEVAAIWKVIKALAGWAVDTAAHRSVTARLSGTLFYTTVIASQILCSRLSLNWHNLSSYWVSVERAVAINIPTDQTMRKRMLTVIITMAILATVEHLMSVVALIGFDCPPHLILRRYTARSHGFLFLRDDYSVWFAIPLIFISNIATILWNFQDALVVLICMGLTSRYRRLNNYVSKICEEEKKLANKNMKAEAVRIYSWRRIREAYVKQAALVRKLDGALGGIILLSSFGNFYFICLQLFLGITQGLSNTSTIKQIYYIVSLLWILGRFTSMVLAAADVHVHSKKALPDLHACHSRCYNVEIDRLLNQLNKDYVVLTGVGFFTIDRNIL

>LstiGR5b

MGLTSRYHRLNSFVNLCVKNEKINRDKTSVTEKYVRTHQWRRIREAYVRQAALVRMVDANIGALVLLSNVNNFYFICLQLFLGLTKSQGSLVSYLYYFISLGWLLFRACSVVLAAADVHIHSRRALEYLQTCPGTGFNIEIMRLNNQLSHDFVALSGMGFFSLSRQTLLEVAGNIIKYELVLIQYDK

>LstiGR6

VDVVSCQYLYKSVADMVEKVKKSFDVVLILTLLANTTDVIIHVYLPFAKEPFKSIVGYDLTLAYVVVVQQLLILFFPALTAGMLTGQVEKLKLVLCDMLIKDKSRKKDIKR

>LstiGR7

DAGFVKIGVPTARRKKFLPTLTHIFTVAQWFGIPSYGNKFAICWAIIVLCMLTVVEGAAIWMMIRLLAGIAKHIDDGRGLTARLSGSIFYANGFLSLILSWKFMYSWKRLSFYWKRAELVDVSLAIPDEAIQRKVIVVTCFVSVCAFAEHLLSMILAIGIDSPPMDFLERYILNSHAFLITPNTYSLWS

>LstiGR21a

MDEEKQMFRIYNTNQINGKQKNTNGIREEYDAKDIYGPEITDKDGALLDEHDSFYHTTKSLLVLFQIMGVMPIMRVPKDAQTTNRTTFNWISKATLWAYLVWSLECIIVVRVGKERLATFQQNTNKRFDEVIYNIIFLSILIPHFLLPVASWRHGPQVAIFKNMWTHYQLKYRKITGTPIVFPNLYILTWGLCVFSWGLSFAVILSQHYLQEDFELWHSFAYYHIIAMLDGFCSLWYINCNAFGTASRGLAMNLHKALKAEHPALKLAQYRHLWVDLSHMMQQLGRAYSNMYGIYCMVIFFTTTISLYGALSEILERGLSYKEMGLFVIVGYCMTLLYIICNEAYHATRKVGFEFQVRLLNVNLGAIDRSTQREVEMFLVAIAKNPPIMNLDGFTNINRELFAANISFMSTYLIVLMQFKLTLLRQSARKAIKTVVKAIFNTTTLGPDDEDDDVEEE

>LstiGR21b

DYEQEQRDLLSSQDGDTCEIHDQFYRDHKLLLVLFRALAVMPITRSRPGTITFSWRSSATAYAICFYIASTIVVLFVGYERILILRSIRKFDDYIYGVLFIVFLVPHFWIPFVGWGVAHQVAIYKTNWGKFQDVGIDCSAKLISRYRYLWLNLSELLQLLGNAYARTYSTYCLFMFTNITIAVYGALSEIVDHGIGFSFKEMGLFVDTVYCSTLLFIFADCSHKSTQKVADGVQETLLTIDVLAVDRPTQNEIDHFIQAIEMNPAVVSLKGYANVNRELLTSAISMIAIYLIVLLQFKISLPKDP

>LstiGR45

AVYLNIVVTGLPLNLIGNLEWVSVVSFVATNLFVALFMSIRCEIFLREVVETKQLCITILSMYTDGPIREKARKMLKLAEASPPRFSVYGMWNIEGRFLLYLFSIITAVMLTELQLLLL

>LstiGR51

LRTIFKALQNTNEVVMGKGMIAYASSRCLRYTVMVIIPCYYSSVTTTQVSYMRTMLHDAMNQVNIGKVDRRRVKAFFQLTRENEFAYAIWGVIRLNMSLPLSYLSLCTTYLVIIIQFAKFID

>LstiGR63a

IVTFISVAIRIFSGVMFPGLSSDKKIFIITACILIAICSSINIAWLVYRCEQSYGQRNTIIRIADHMLVDKNISESMRRTLSEFRNLVDSRPVQFTAMDFYPLSYGLVVSSASVVTTLTIILLQGLE

>LstiGR63a.1

KRFLSQIQINNNKQMDETKVGQNKQNESEDAFDSLNFINQLLKIFCLSILSRENRRLKISYSWFKVFFTIMCIICLIIFLTYDIVKFYAYEIQHFKFNDEVLLVILVRAVLYSIDLCYVFKFGGNTNLHYFKLYEQIDTILDTDNAMIKTKVLKVTVFITSLYGIQTVVNIIWAAFYDPTESFTTVRATVGIIMIYINSLSILEMLVHVILIEYRLIKINNILQLRCSSTTNNFGALSVLVENNWLYFSKHKEITRNPQVDCNYFYDISWLNKCYLLLIEQSNFINKLFGVRILLNSVINLWDLVNNINFSIRISFRVLDLDPETTILNILSSTLNISSVAAILICLVYRCEKTYEQRRSIINVMDRILVEKYINVSMRSRLAEFRTLVYFRPIQFTAAHFYRLDYALLVTFCSAVTTYSIILLQYLQ

>LstiGR63a.2

TMDNRRRALLPSRRYSSKNMIQVSDVKSVTSDQSWFNNPLSFLLQLFSLSTNAHRDKNFNSCLSLIRMIITGVGFGVLQLFDLYYKIGHVYSGLSVSVRLTDSVQTIYDYFQYTVDLFYVYKYGRHFYQEYYKQYNTIDQILRAGSCNAIRKKITKLVILFVSIWLITSVMDFIAWVLIYGWTIPTVFSLAYNYLLLKILTNLDLTYQTMHIEVRLQVISGLMQSYYTCCDSLPGGPGEKCGDPVQNKNWLYSNFSVPPKDSLKWSAESRRHGIRWLTRCYLLLKEQSAFINQMFGVRVLLNSLSLLIDMVRFSNLAIRLVMGLQQDGNGAKVTKYIVNGVKRNMHDWEYFTAISTVCRLLVCAVILTNLVHHCELVYRQTDRIISISDHLLINKNPDPDLREAVTELRDLVQSRPIDFHMANFIRLDYSMLMSTA

>LstiOrco

MMTKVKAQGLVSDLMPNIKLMQAAGHFLFNYHSDNSGMTTLLRKMYSSVHAFLIVINYLCLAANMAQYSDEVNELTANTITVLFFAHTVIKLLFFAVSSKSFYRTLAVWNQSNSHPLFTESDARYHQLALTKMRRLLYFICGVTVFSVMCWITITFFGESVRYIANKETNETLTEPAPRLPLKAWYPFDAMSGTMYVVAFVYQVYWLLFSMAIANLMDVMFCSWLIFACEQLQHLKAIMKPLMELSASLDTYRPNTAELFRASSTEKSEKVPDPVDMDIRGIYSTQQDFGMTLRGAGGRLQNFGQPNPNNPNGLTQKQEMLARSAIKYWVERHKHVVRLVASIGDTYGTALLFHMLVSTITLTLLAYQATKINGLNVYAFSTIGYLSYTLGQVFHFCIFGNRLIEESSSVMEAAYSCQWYDGSEEAKTFVQIVCQQCQKAMSISGAKFFTVSLDLFASVLGAVVTYFMVLVQLK

>LstiPR1

MKSYRILIRKFGREFHLEHYTHMGQIYEDMNKKINVISVYFTRFMMCQMILAMIMFNIAPMYNNITNRYIRHTENYTLEFSLLISYPGFKPLNYFATTTVYNFYLSYNCGVMLSGLDLILSLLIFQTIGHVKILRHNLENFQSPKNKVVIKLDEPHKYKFHGSCLYEVFDEEENEKIRIKLAECVEHHRQIINFTDELSELFGPFIAFNYLFHLVGCCLLLLECTGNDGGMLRFGPLTTVVFGQLIQISCMFELMGSEAEKLKDSAYMVPWESMNVSNQRTANIILHKMQYKISLKALGLAAVGVNTMTGILKTTFSYYAFLQTL

>LstiPR2

MKNKSPLTLKYIKIIRSFLRPPGGWPSEVFGEKLSLAIRFHRVTLPFHTSLIVIGGFYHLYDNVHRLSFLEFGHIIITTLLAMVTVLRSVLPNLQKFNSLLSKFINDFHLMHFTHKGEYFEKMNKMVDLISNYYTMVSTCMMYVGMLMFNIGPTFNNVRNTVFLKTENYSMEYSVYYSYPGFKPLDYVTIASIYNCYLSYNCSTLLCGFDLLLFLMIFQTIGHVYILRHNLENFPSPNNKIMLTFLGDKYRNKEGCICEKFDPEENKLVSLKLAECIEHHKIIISFTDDLSQIFGPILAFNYFFHLVSCCLLLLECSEGGLDAVIRFGPLTLIVFGQLVQMSVIFELLGLETEKLKDSVYCTPWESMSVSNQRTVCIILHKMQYKINLKALGLAAVGVSTMTGILKTTFSYYAFLQTMGE

>LstiPR3

ICLLCSNARHPTDLHYMKMIRYQLRMICSWPQKLLGEEVKSVPLRNTRFLFIEGSLVAFLGLVYIKTHHTRVTFLEMGHTYLTVFLGIVAAQRVTVSWFKSYDQTMKNFVLELHLFQHRHKSEFHEHMYQYINKICTVFVDFIHVELFMGIILFTLTPVYNNYMKGMFNRATPVGPDKFFEHSINYSLPYINQFIYDELVSYLFIAILNVLFAYDAGICFGSLDVTLSVIVFHIWGHLKILDHNLRSIPKPVNELTYTAEENKKVGGILKNIVDHHRMIMSFMTNTSDAFGPMLCLYYMFHQVSGCILLLECAELDAKSLTRYGALTVTIFQLLIQISVIVELLGSQVRVNSISQMVFKTRTNCLTNQRVRYVL

>LstiPR4

MHKLRMFLISDGSDLKDVKRVVDIKYIQVLRSYLRIISAWPAKHVGDTPTKWDRIKGNPVLVLSIINFLTGLLYLKENIGKIKFFDLGQTYITVLMNLVSVSRQLMVYQKSYTEVSRDFVTKVHLFNWKDDSEYAMEIHILVHKISHFFVMYIHGLMFIGLSMFNLTPLYNNYSNDAFTKRLHGNATLEHAVYYSLPFDYTTQIPGYIVVFTYNWFISLVCSINFCSVDTYMSLLVFHLWGHLKILIHNLEHLPKPSGLKSAANINGATQTERYNEDETQQVSERLRDLIKHHCLIRNFISIMSSAFGYVLFVYLGFHQVCGCILLLECSSLEPSALTRYGVLTVIIFQQLIQLSLIFELLGAMTEKLMNAVYNLPWECMEERNRRMVCLMLRQSQLPLRYKALNMIEVGSATMVTILKASISYFVMLQTFATKD

>LstiPR5

MDLRYMKQLRNFLHLLDCWPHRLLGEDVKPFPLRSIRVLVTEWIIILVGGVIFLRANINKRDFIELGQTYLTIFLTAFGIQRVTISLSKSYQELMNDFVLEIHLFHHRQKSKYSEYMYQHIHKICTVLVSLMYAEAIISSVLFNVTPLYKNYKKGMFSQERPSDKRFELSVYYSLPFVNQETNLFAYIVVSIFNVTLTFDCGLIYCGLDANLAIIVFHIWGHLKILDNTLRSIPTPVEMRNHIPRFDDKLSYTKEENEKVAAMLKYIIHHHRLIMGFMTKTSSAFGPTLCLYLLFHQISGCFLLLECSTMDAESLGRYAALTVIFFQLLIQICVIVELLGTQSETLKDAVYSIPWESMDTSNRK

>LstiOR1

MLVKKFKAFYNKEGFDYSKGYIDPRDFHLTFFFVQRAFQVIDEPFQPWTYVSKTITVICGIGVLTDACFSFYHAIDIFDMGMITEAGTYVLMLMYKMMTLTITKINLSSYIHLIKCMKEDFAYICTKNDKYRKAFFETHMATWQLCVKVCMFMFWLATSLVLFAIGSLFFYLATHEPGDGTHRPLVFPFWAPGIDYTTSPAYDIAFNFANIGVIACTYNYTFVLQTNIVWVRQIASKAEMIGMCISDLLEGIQPANNEEEKRHYARMINFRMREIVSQHQKMYKLLDSYAAVYKKCLMFEQFVSSPVICMLAYCSAEVCFFSGFMSLLYLKIEKRGNALTIN

>LstiOR2

MTLIKAIKGIFFKDSFDFSEPDIDLYTFHPQLRIFIAPLGIFFNNRKSLLRFLWPFINGSLSIVAIVLEMIFVYHGITVGDYSFATECFCYFVMLSVIPVLYCAVLANSQSVMVLLDKMDKDFAYICKLGAKYRDHFLQRQLLIWQLCWIWLGFLCCVAVLYTLMTLAPLTYQTLIATQDENMIRPLLFPMWLPEDDPYRTPNYEIFLFLQLDYLLIFIQSFGVYVYIQFHVLLHNFTVLELVTFDFDVIFEGLDKSVVDLPRDDLRRLTVQRVFNRRLKRVATWHDSVFKSIGTLSRVQGPVIVYQVMFSSLCICLMMYQVADKLDKGTFDILFIMLTVAGITQLWIPCYLGTLLRNKAFDVADACWNCGWHETPLGRMIRPDIIIVIMRAQHPISIKFTGLPNLSLETFSSIMSSAYSYFNMIRQSNN

>LstiOR3

MFKNFSLRFEDPEKPLYGPNFWILKKMGLILPDNRTGKALYILMHEIVAFFVFTQYIELYIIRSNLDLVLTNLRISMLSIICIVKANTFVFWQTKWKDVIDYLTEADRFERESNDPQRKIIIDKYTNYSRRVTYNYWILVFITFLTTTGSPFIHFASAVYRESIRNGTEIFPHIFSSWVPIDKYHPPGNYITVVWHISVCAYGAMIMASYDTSIMVIMVFFGGKLDVLRERCKQMLGTGEVALSDSEVAARVRELHDTHVLIMKYLRLFNSMLSPVMFIYVVMCSLMLCASAYQLTSAQNTAQKLLMAEYLIFGIAQLFIFCWHSNDVLVKSDNVMLGPYESEWWAVNVRQRKNILLLAGQLRISKVFTAGPFTDLTLSTFITILKGAYSYYTLLRD

>LstiOR4

MSLAGRSVSAHLTFLRLCGFCRLGRDGSSPLARRAHAFYCSFALAVTTVYLMQECVYAYQERNDMDKLARVMFLLLCHVTSITKQLVFYLDADRIDEMISGLDDPLYNQPVSWQKSLLTETAVSARRLLRVYSGTAVITCTLWIIFPILYYSQGLPVEFPFWTNLDHRKPVFFVILLMYSYYVTTLVGIANTTMDAFMGTVLYQCKTQLRILRMNLENLTQRASVIVKKDPNEIFDKVLERLFLECLVHYKQISE

>LstiOR5

MVSTSNAPADFFDFNLKYLFYVGLWPREDWPPTLNWLYRIYEVTLMLFAFAFLTSTGIGMYMSKDDVITFLTNMDKAIVAYNFTIKIVIFFFKRKHIRVLISEILHSGDKIDKSRQKLMMIHVVAISGMITTIIGSFQTFAQMKGEMVVDAWLPFDPRKNMWTVFIAGQILGVLFVVPVIYRAIAIQGIVCSIIMYMCDQLIELQGRLKALTYSVENESYMREEFKDIIRKHIRLMGYSKSLKSEFKEYFLVQNLAVTTELCLNALMVTIVGLEQKNHLISFMAFLIVALFNAYIFCHLGNELMDQSAGIANAAYESTWTSWPIDMQKDLLIIITVAQKSFKLSAGGLANMSMQTFAEALYNGYSIFAVLRDVVD

>LstiOR6

MKILPKGIIKKCTQTVGVNNEIDEIMRLTLFFTRIFGHHILDPNWTWNITFPYQLATLLLISYVIVGTLEIIRGTNDVKLIAEAAYTFIVIVVMQTRFYFFLSTRKHFQHLYIQMKTTLYNSILDDPEKNLKDVLKKLRMVVNWMAFFCFFPVVIYILTTLWCYFNGEKRLMSKTTSILMPMRTPYYEIGLLLHSIFMFSAAFTIGEVEIWFVMMMIFFRTACDGTEKYLSVEARHENESQHDYAIRLKNSLRKFYKSHVKEIEFLNTLNAMFKWLGMMPLISVALCICLILLLLSKGIDMTFVSNVIPVIVELFVYNWFGEEIKIKAEKWKSAILEFDWLNLLPKDKKCYCILVCYMSKEFGIKIATGTFLSLLTMSTSLKFSYQAFTVLQTMDI

>LstiOR7

MAIFRKDIPASKMNHQNFTFDKIFIITAKAMILNRSHPSIPRNWFWVFQFLVILTLSATTFLFLINSVLFYDIPARRYAEASKNSTMAIVAFTVTIKYLFMLYFQKYMQDLIDVVDRDFKLALDFEEEEKEIVIMYAKKGSKASWYWLLAASSTSSLFPLKALLKMGYSYWKGEFELIPMFDMRFPDRVDIVKEIPAVFAFYFVLCFMFSCYAGSMYIGFDPLVPIFLLHISGQLNILSKQIMRIFTENNSVDEINEKLKHVNIKLQDLYWLIENIKSKFTVLFEYNMKTTTFLLPLALFQVVEDLKRSQLNLEFISFFIATILHFYMPCFYSDNLLDQSNYLREAIYSCGWEKHSDTRARKTILLMMTRTTKPLVLSTVFYPICLDTFAEMCRQAYAIFNIMSAACA

>LstiOR8

MEVYHESGLGGTENAEDIYFNPFEKTFKFVMFCMVVGMIYPYPKMTRMWQIAAICFYLIIPNPSSSIVVYDVIEAYKEGDMDCIFRHIIVMGPFITHYLKMVLMYIYRSNAKLLLEEMNEYFEKLNSKPLSHKLIAKKWLTKSFFLEKSWAYCVLAGSFSFPIMAICKNIYSALFDEYPRRYCIQELRSPFSGSNLDSPFYEIMFINTCMASCMYYINFNGYDGFFVQLILHTALRIAVCGESLKDAFNIDDSVLRRRAVCMVIKEHIAICNFINRINVLFQHWMSIITSYMVIHFCVCVFFLSKRSGLQESQFLCAAFASVMYLFMICAVGGLIQDESEKLSDLFYECGWERISDPDCRRLLVFMIARAQKPLQVQTIAMYNVNLQLFVKVLKMAYSLFTFLQQT

>LstiOR9

MENQFKPFHETYKAVMYSMVFAMVYPNPATDKWRLLAIPLLLLTMLPMSVIALLDSLRCWNEANYLEVLRHIAMFGPFLCGILKMCFMYHRRVEAKAIIDTINEDYASYNYFPENYQTFIRAYIENTKIYHRVWYFCVLLILSAFVMTTTLYNTYEYMFRSEPKRHMIYDIRLPNKAAGVELETPYFEILYMYMLYVALIFYLNFTGYDCFMITAVNHACLRIELFCKHLDDAMEFKGEELRRRMRTAISEQCETFKLIDACQSTFNGNLGMVYLAVTTELCINLFLMTEGYEFDYKFTAFSIGTILHVFVPCRIAEKMKNVCEESSTMIYCCGWEEMYDLSVRRYIPFMLARAQYPATLKAFGILTYDMMLFASTVKTAYSLYTILKRQQT

>LstiOR10

MSEATLAEAKREIAESLTLNTFCMRRIGLSFEEPKNASSYLAQKFMLVLSVMSICYHVFSEIVYIGLTLSNSPRVEDVVPLFHTFGYGALSIAKVFVLWYKKDVFKQLIHELAGIWPMPPLDDDATAIKLKSLTALRIAHQWYFAVNVLGVWFYNLTPIIVYTYRLWQGQEVQMGYVWVSWYPFDKYKPFAHVVVYIFEIFAGQTCVWIMVGTDLLFSGMASHIGLLLRLLQRRLETLATQAQTEEDDYREILNNIKLHQRLITYCNDLEVAFSLSNLVNIVLSSVNICCVVFVIVLLEPFVAISNKLFLGSALIQIGMLCWYADDILHANADVAAAAYNSGWYRTSARCRRALLFLIQRAQKPIAFTAMGFTDISLVTYSSILTRSYSYFALLYTMYNDK

>LstiOR11

DLAITTKVCASIEINFTTMEGIYPEEFINSVLKSLSYFKKCNIDVFDSKNSLYRKFWWLFNIPSFILNYITLTMYIVKIFTEGVDPFEKIYMIPVWLVTTQEFFVCIIIIQKEKEIRTVIEHLGSIWRTKDLTEYQSNHKKTTMKQLNFGQKIFEIMSLIVAWLYMLMPLAETLFRKFILDQEAELMLPYASVYPFAVDSWATYLGVLAFQIYNMLFVIFMYLGSNLLLVSLSTGLSIQFDLLRADLINIKPTNNRENIVFEINDENVKWSACNIEEFVKFHQDVILLTQELNAVFDKIVFLSL

>LstiOR12

MTSLDKLADCLPFLVSIIIVVYFGLYRKEMYDLTKFMQREFKYRSAHGLTNMTMLNSYKTARNFGYFYTACTMFSVTMYVIPEIINRWNRQPLQSYIYMDVVRSPFFEFTFLRQCVAQMFVGLAMGQFGVFFASNAILLCGQLDLVCCSLRNARYTALLRCGIKHSVLAAAHGDIQGDELYNYIYNAAELQPSRYHYDQKMNHNILNTKTSFDIYSREFDEATCEALRDCARVSDVINAYKAKFERFASPLLVVRVVQVTMYLCMLLYAATLNLDMVTVEYLVAVMLDTFVYCFYGNQIIIQADRVSTAAYQSAWHTMGVRPRRLLLNILLANRRPVAVRAGYFLPMDLHTFLVIIKTSFSYYTLLVNVNEK

>LstiOR13

YTFRKVTRTIVDGYLVCDALTLKGERFTKNLLKTLKDVKKRALIFWVVIIGNGVIYFVKPILLPGRHLMEDQFILLGLEPTFESPNYEIGFVLMSCGVICTVYLPANITAFLIVLTGYTEATMLALGEELINLWADAQRFYRNNHVEIDITDENALVNLTDDAEKNRIMNRYIKQRLEEIIKIHTRNINLITQIEKVFRGAIAVEFLLLITGLIAELLGGLENTYIEMPFALMQVGMDCLTGQRMMDACVKFEESVYDCKWENFNVSNMKTVLLMLQNSQKTMVLSAGGMATLSFS

>LstiOR14

MINFKSLWTRLTHTKALEKSSGKLETRFFETVYRVSYLTGISAADDDIPYMIYSSTVKLLIVLLVCGEIWYAFTETSSLDEIAASINTTVIQFITMYRYRNMIRHKDVYKKLAMSMESPFFDISTQERRNLVDYWVKKNERYLKLLLFLGNCTLAAWFLYPLVDDLEYNMFIGIRLPFQYRSLIRYTFAYLVVVMAFAYISHFVMVNDLIMQAHLLHLVCQFAVLSDCFENILADCEKKFKGADRERLIANKRFREAYRVRLGDMVNQHQSILSHVMDLRRTLSGPMLGQLAASGTLICFIGYQLTTTGADNVTKCLMSLFFLGYNLFEFYIICRWCEEITVQSQKIGEAAYCSNWECGLADIPGVKSCLVLVIARANKPLVLTAGGMYNLSLLSYTSLVKTSYSALTVLLRFRQN

>LstiOR15

MLSQKLISFLERLEDQSHPLLGPNVKCLYIFGLWQTVKTRKRNFIYNIFHFTTFLFVMTQFFDLYKQLDDFNKALNNLSMTFIGVISCAKCYSYVLCQRQWQKLAADISAEELAAMEDGDETVMIKMKEYKLYSRVITYLFWVLVTMTDTALIVTPLIKYLTTPMYRADIREGIEEYPQIMSCWFPFDYMSMPGYMFSTMIQIIMSIQGSGVIAASDANAITIMTFMKGQMQILRQKCIKIFESNSYEPKEILKRIKECHRHHTFLIQRSEDFDKLLSPVMFIYVLICSMAICCSVVQFFSSGATAAQKLWVIQYTSAQIAQLFLFCWHGNEVFVESKDVDQGVYESDWWKADVRLRKQVLLLAGKLNRPILYTAGPFSRLTIPTFISIIKGSYSFFTLFAQMQEET

>LstiOR16

MGGSFQSHYSFCFLIMLRRFVSSLEHEDNPLLSPTLWGLQKWGMWQPKNGPSPKISNAIHFAAILFVISQYVELWLIRSDLNLALRNLSVTMLSTVCVVKASTFVAWQKYWRNVIENVSKLEKRQLSKKDKMTNTIIDEYTKYSRRVTYFYWTLVTATVLTVIVAPLVGFLSSAETRQRIRNGYDSYPEIMSSWVPFDRSRGLGYWVSVLEHILICFYGGGIVATYDSNAVVLMTFFAGQLKLLSVNCSRLFEGEEEMTYEEEIKKIRECHYHHLLLIKYSKILNGLLSPVMFLYVIICSLMICASAIQITTDGTTTMQRIWIAEYLLALIAQLFLYCWHSNEVLVMSNEVDEGVYSSAWWSRSIRVRRCVLLLAGQLRRSVVFTAGPFTEMTLPTFVAILKASYSYYTLLVNKDD

>LstiOR17

PLESFFDKMTHIFLTRQKKALTLLGHWFPTKKYRIPYLIYRCFLLFIQWSFLLFNIIYMRQVWGDLEETSEGSYLLFTHATLSLKSTIFLMSKNRWSNILNFMESDIFAAQTSVHEKILSVDAMKMWSVYAFFITSATFNCIEWAVVPLLDNHGERVFPFKIWMPADPTKSPEYHIGYVYQVMAIYINAATFLTMDYLTASLIMFAATQLGIIEEKIKQIPATPLSATLEEKNKLIKQNNEILNECIQHHQAVIRFVRLVENMFNVNVFFQMSGTVAIICVISFRMTIEPPNSIHFFSNDY

>LstiOR18

MTTTKADTKNRVYSHTDYDDSYKIITKNILSLVGIRIAQKDSSFARLCWNILYWAEFANLFIALVLDGYTACDVIRSSALKEENIVFMMLPCMGYLVIALLKSYKTVYQRGIYENLVSELRSMWPQGSVTEEEHIIIDKALKELNIVVKGYYWCNLGLCFSVIGPPYVDLVRRAFGEDVPRTLPYFYWVPYDEFQPVAYELTLALHAWQTMLTLWFMQAGDLLFCAFLSHITTQFDLLCLRIQRLFHVPVDQQLIAEYPLGKQSKKPSDNETFSPLNESETRSKQEKELKKIIVRHNDLIRLSNDVENLFSFALLINFFNSSIVICFCGFCCVMVEKWNVFMYKTFLATSLSQTWLLCWHGQKLLESSARVADALYNSGWYIASNPIKKSILIMIHRSQKNVCVTTYGFSVISLSSYTTIIKTAWSYFTLLLNTYNQ

>LstiOR19

MDETLKVFHRVLSFAGIAIYAKEKWNSNLWLSFQIFNFLIGTFCFIFTTGFVVSNYSDLLIFIQAACIWTTGVIMTMSLGVCLIFRQKFRMFLCEMVFKDEVLEMPLIRYVLRLESGKKLFELKQMVKDSQEQLFRVTGVLLKCYVTSVWLVATLYLCSPIYEMFSKGDKSLRLLAFDMWFPWSLENLKVYIASFIFHAYAGYLCCVAYPGLQLTIILLIGQVIRQLKILTFIMLNLNDLVLEITKEKDIRWQTYCTAVLSQCVDHYIKLKSFSNRLNVICRPFYLTLILVAIMLVCMCSVKIAVSDKLSPDTIKYYVHEFCFILVVLMFCLLGQQVDNECEQLERAITENWYIFDKKHKTHVKIFKMALTQRMHIFIFGTITLSLPTFTWFIKTGMSFFTLVMSVLEEGNYE

>LstiOR20

MTNTRTIDKIGIPTLNFLNFLEDPRYPSVGPHLRLLGLTGLWHPNLKSRITRFKQYLFFVTIAFFLSQYVKCLIKFDPIYLKLVLQYAPFHLGIVKSCFFQKDHKKWETLIDYISAVEREEISDGKRQSNEIISEYIKRGRKVTYFFWGLAVVSNISIFTEPYQKNQINVNGTSVYLCVFDGYTPFGEVPPGYYASMFIQTVLGHIVSAYVVGWDTLVCTIMIFFAGQLKISRMNCTNVIDTGNADINHKNIVKCHNFHTTLVMNQKLFNSLISRPMFVYLIVISVNLGVCIIEIVQQQNDLTTLISSCVFVVACLIQLLLFYWHSNVVSQESTVVSYGTFESNWVGLDQRTQKEVYLLGLTTSTRLVFKAGPFNEMSLTTFVAILRLSYSLYTLLDNTM

>LstiOR21

YTRRSCYGSYVFLPGIGEVVYLLKRRENIGDVAEGLYLFLSEMYTYFKVAVFWLNKDKVINLLGYLSCEEFKPVEAEHREIIRKSIKAARFVMTYYSTMCVGAVSVGIIMPLTENFDILPTNVEYPHFDVYKSPAYETLYIHHIYYKPATCIIDGVMDTILAAFVASAIGQIEILAFNLRHFDLVAERRRKRAVAGNKPSATWTKERHIRAVLKECIVHHNSIIKYVSMIESAFSLASALQFMLSVMVLCLVGIQFLSIENPSRHPMQILWMAIYLTCMLIEVFILCWFGDELIWKSTELRQAAFDGPWLETNHKTMVFIVIFLERCKRPLRVTAGKIFTLSLDTYTVLINWSYKAFAVVSNMKK

>LstiOR22

SFIRYSSFFLSQSYRDNVKEGNEDYLQVVSSWVPFNKSTIQGYLAASIWQSYASIYGGGWITSFDSNAMVIMVFFRAELEMLKIDCANIFGTEWNPVSDKVAFARLKDCHRRHVELVKYSRLFDACLSPIMLLYMFVCSVMLCVTAYQITSETSAMQRFLTTEYLVFGVAQLFIYCWHSNDVYFASLRLSEGPYESTWWCRHVSHRKNLFILTAQFSRVVVFSAGPFTKLTVATFISILKGAYSYYTLLSKSQTK

>LstiOR23

MAESFKVKTSRMDIIRHKVLVWAGIYKLHTKKYYLGVCHDVYRVFVIVMLILVNIQHIIYIYLRAIRGEDVPWDIILLVITMIDLIIKVVTINIHSKQIDEIHDLIKAPMFDPTCTEDETILKKTEKQINMLLKIVYIDVTVLNITWDIYKIGQRMTNKSAAIESYFPFNTNPWPGYLLAFLYEWWIIIIWLGYGLLSLDCSIAIYYTRAATQLKIVNYHLEHMFDNVKVQSQKRFQYKDLVDRSLNLKFIHFVQRYQNIHRLINTVNIAFSEGTAFQFFSATVGIGFCLYKMTYTELFSVEFQLAFGLVLIYQMQNFMYCYFGNKVESESDRVCTSMYFSDWPSASPRFRRQMLIAMARWARPITPRVSIVPVTMATFEATVRLSYTLYTVLKSRSMTMN

>LstiOR24

EDDDFDEKTITEVNTVKIFNYEQTDEEKEIVKESIKFLNFVVRLQYYICGVVIFAFPLMPVTSMAYDYYMTGTTEYKYPYLVKYFFDVYNMKMWPAVYFHHVGSTAIVGAAVFGSDSLFYTVCIYIQMHFRTLCLRCERIVTSSAKETRENLAKAVKRHQELIDLVDQVEILYSKSTLFNIVTSSFLICLSGFIITVLEDISVVVTFATFLFMNLSQISLLCYFGDMLMRSSTEVSSAVYNSLWYETDERTKKSMLVILMRAQKPCKLTACNFADLNLTAFTTILSRSWSYFALLKTMYK

>LstiOR25

MDSNKLSHSFYKITYVWKLLGMWSGKSSSKNLRIYSWLFVTLYYIMYNFFYTLSLVCAPRAVDTIGVGVYYFTTLCGLAEIVMILRNRQKIISVFETMDCKEFQGNNRQTNEYLRQFKITFSRYFKVYAAYCFISSHTFLMFLPLFNYFFQHKELEMPIWEYYFLTNATRNKYFFYLYTYQSMGMIATIFNHIVYNVFLFGILSVAVSQSKVLNWNIANIRLADEDILKSTEEKERLYLNKLYNCLKHYEIILKYCEDVQDLTSFLISLNYGLSVFTLCFSMYMFLLPTNSNTLVYMGFYLSAILIKNFVPSYLGSELTNESNNLRFAVYSCDWVPRSKNFKTCLMIFVERARRPLLIKGLKVVPLSLATFTSIVKTAYSFFTLLRGAQDQLI

>LstiOR26

MGIFKGFKRIFFKENFEFSSPDVDLDNFHPQLRRLIVPLGIFFNNQDSFLRFLNPVLNSLMIFIAVVMEMICVIHGIQTLDYSFFTECFCYLVMLLYVPVLYFSVLGNKDSMLEILHQMEGDFKFICNLGDKHRDHFLKRQLLIWQFFLIWMATISSVAFLIFLRTLIPLTYQSLIATHDEHTIRPSLFPMWLPKDDPYRTPNYEIFMFFQMYFVCVYVQSFGVNVYIQFHMLIHNYTILELVIIDFEMIFEDLDEDVVYLSRYHPRRVLIQRILNKRIQRIVAWHDSVFTSFDNLSSIQGPVICYQVLFTPIVYCLMMFQIADKLEGGQLDIYFAGLLSVFTFQLWMPCYIGSLLRNKGFDVGEACYNSSWNTTPLSRMIRNDIVIVMSRAQQPLSMQFFCLPDLSLETFSSIMSSAYSYFNMLRQYNR

>LstiOR27

MATFNSEDLFLNRAKFVMKFLGVWIPPVDESLPRKFLKLFMLMLQYLFLIFQTIHIVQIWGDLAAVSQPSYLLFTQACLCMKITVFHVNVDKLRELLKQMASDTFMPQSIVHEKILKAQAARIKKYLLAFMIGSQGVCSMWYLHPLFEGTGVRKFPFDMWMPVSPEDSPQYEIGYAFQLLTICMSAYMYFGVDSVALSLVIFACAQIEIIKDKLLSIAPVQYGLKEKERKIMNEKNHKILVECIVQHQAVVTFTQLVEDTYHLYLLFQLTGDVGVTCMCALRILVEEVRSVPFASIFLYVIVMLIQLFICCWSGHELTATSEDLHTVLHKCSWYEQDLKFKRDLRFAMMRMNRPLVLRAGHYISMSRQTFVAVLRMSYSYFAVLNQANKKDQ

>LstiOR28

MWENLRKFGLGHCDLPTMVWNVAFMLRGFTLNIDSRFTGRIPKIFYITTIIIAFCYLYSYFFSMLWFVFWRCIETGDVTAAMIVFPLGITSEIGIAKFIYTCVYRKKVRQLLQQYLEYDSQIPQGSRLSRHLLQALRNVKRRALIYWIFIVSNGTLYILQPLVMPGRVPMEEVFVLYGLEPELETPNYEITYVLCTFGSVCTCYLTSNVAAFLIIVSGYVESQLLALSEEILNVWDDAELEYKVIDNADEEEFENKEKYDAINESVKTRLKDIVKGHTTNINLLLQVEDIYRGTFKYARALHWTRVEIETAGFEVGKANYENSWLLLWKMQSKNVGKENVNKILYVFISFSNALDYSALLSPISY

>LstiOR29

MHLFPSKKIHKGLRFAMNVIILLFMMSEWAAFLTQSNLTEKQATDRLMFGFSHPTLFSYVLAVEYHQERITNMLHKLAMVLKEEYNDKDIERKMVKKAMSNAAGFACLFMMALIFYGYDGIMQVIRGDGTFTTVITFWPDVTEVSYAASITRVATYFIWCVFMTRVCAVYCLVIPTTVCLSHQFKNLQSYFYSLEDIFDEDLEQTELEKKYEEAFKVGIKMHSKTLECTKDYQTAYNVIVSNQVLTTVGVLVLLMSQMVDSERTLENVLSIVTTGSAMLISTGFFMWNAGDVTVEAAELATAVYCSGWAHCQHSAPRVRRLLVITMMQAQKPVVIWALGIVELSYQSYVSIVKSSYSVFSVLY

>LstiOR30 LLLTMKNYEILKKYCKKMFLVGSGNFWYESGIVGDDSSWYYKIYSRSLFSIYGFMTILEIMAAIFGDFPGDEKRDSVTFAVSHTIVMLKIFSVVSNKGLVKTMNQNMVKICEAYEEPTLMAAKYRIVRINVLAYFSVVYGSAACFVCEGIRKLNAGSHFVTVVTYYPSFEDDSIFGITFRIFTTVILFLMMMTMIVSVDSFTMAYLIMYKYKFITLRHYFEGLTEEFHKMNSVNPRLAADKLTNGLVEGIIMHKEILRMAKDIDQAFGTVIALQLLQSSGSAVSLLLQIALSDQLTFVASMKIIFFVVALFFLLGLFLCNAGEITYQASLLSDAIFFLRLARQQMAAASAAQSQATGAAGGYAVAAAARHEGLQDD

>LstiOR31

TCRLGKMSLAGSSVAPHLALLRRVGFCHLQGAAGGGSARSGPKRLHSYYCLFAFGVTSGYVLQQAIYAFQERSDLDKLSRVLFVMLCHCTCVAKQAVFHADARRIDRIITGLNQTLFNQPIESHRSQLRGTALSAARLLRVYYSTAVATCVLWIIFPVVYKLRGHRIEFPFWTFVDYNQPVMFVVVLFHSFYATNLVAVGNTTMDAFIATVLFQCKTQLRILRMNFETLP

>LstiOR32

MLYNGMGTCSLLRHVLLLQPKRKSVWLLLHSNIAAKMAGPTTYSTFSAVTPHFNALARVGYFKMVMKNPSPTQLMLHNCYRWLIWFSILSYNIQQLIRVIQTRHSTDEMVDTLFILLTTFNTLGKHVAFNARVWRIDRIIKVINGSIFAAKNPKHVDIMKLNEKAMARLLYFFQGMVLTGCVMHATYPMINRALGQDEISSCFSSESSGSLSTQIATWYLSISLTIQAYGNSTMDCTISGFYAMAKVQLQVLRYNLEHLVDSEDEQEDIDTNDVNIGKLRYKDNTVIQSRLVHCVKHQLQIKWFVKEVESIFCEAMTVQFLIMAFVICMTVYKIVGLTVSSAEFWMVFVYLNCMLAQLFIYCYFGTQVKYESEFVAQSAYCGAWTRLSPAFRRQLGILMQCARPIVPCAAKIVPVSLETYIAVLRASYTLFTILDKQ

>LstiOR33

MKWLVKNTFTALRISLTCLTITGFYTRRREVNFVLSYCFPFLTFMFMTGISIMAQFVDLIIIWGDVALMTGTSFLLLTNVVLGLKVFNVVWKREKIRAVIEESDGQLQAVDTNWGKDILKSCERQSTIFFYIYMFFPYLTIMGWATGHEKGELPTRAWYPYDTTTSPGYEITSLHQVVAVCIGAAVNVSVDTVVTALLAQCCCRLKLLSACLKMLGDGMLTNNQGMFKQDQEVAIKANIRSCIQQHQAVLEAADLLQEHFSSPILAQFTVSMVIICVTAYQLAFESSNTIALLAMACYFTCMTLQVFLYCYQGHELSVESGNVGAAVYESPWYKMSLPLRRDLLMLMMRSQRLAKLTAGGFTTLSLNTFMAIIKTSYTLFTVLQQTED

>LstiOR34

MMVKKIFSFVRRSPLQETRDLKVNDLVAMITKRIQNAGLNYRDENLKVHWLAIASIICFIITYGLQVVALVNAKDDIDRLFECLSVMSFCGMGILKLLSLYRNHKHWKMLLNKITELEKEQVINEGTSNEEYESDNEDDTTYFPDYIATYTKQFQTLSDILSRIYGSTAIIYILSPYAEFALLKFTGSDVSSYPHILPGWTPFDSSFFGYLATIAIELVSAIYCVCVHVAFDLTSIGIMIFICGQFSLISDYSKNIGGNGAMCFLSKRRDDRAHGRIIRCHKIHVQLINTCDELSKLLQNILGVYFSVATLTLCSVAVRLNSELSSMELVSLLQYMCATLTQLYLFCHFGDNVLHQSAVGMGQGPFGAAYWCLSPRIRKELAILGMGMMIPRSFKAGPFISVDLPSFIQVVRTAYSYFAVIRK

>LstiOR35

MIVKNVNTSVSISLTTLRLVGFWVPEHFEGNKKLLYDCYGIFSFMFLLGTYLIIQTVDMYMIWGDLPLMTGVAFVLFTNLAQTTKIVFMVRRRRQVHAIIKEADRELRAVDSNEARAIVKSCNKETIFLQVVFNCLTLVTMVGWATSAEKNKLPLRAWYPYDITRSPAYELTYMHQIGALCVAAFLNVCKDTLVTSLIAQCRCRLRLLGLSLRTLCKDLRTTEQNHLSADQEDIVRARLAKCVKQHQSALEAALQIQRSFSEQTFAQFNVSLVIICVTAFQLVSQTGNLVRLMSMGTYLLNMMFQVFLYCYQGNQLSEESAQIAGSAYECPWYLMSTPLRRSLLIVMTRTRRIAKITAGGFTTLTLASFMAIIKASYSLFTLLQQVEEKD

>LstiOR36

MDIPAFEDLFKEIKINLWLFGIPFNCSRIRLRFYLMLIAIVLMIIGESCFLVSRYSPENLLELTQLAPCLCQGLLSALKILPIAAKKEKIFELTKCLDRLYSTILMDAHKKAVVQREMTLVKILMKYFFILNAILISVYNFSTLLFMFYSYVVRSEVEFMLPYAVIVPFSTETWVTWFIVYIYSISCGFICVLYFTTVDALYCVLTSHICKNFAIISNEIQGINASNVGNLKDLVKNHQYVLKLSEDLEEIFRLPNLFNVLVGSLEICALGFNLTMGSITEKPKSFLFLSSVLLQILFMSVFGENLIRESRKIGDAAFCSKWYDIDMSSKKTILIIMTRCRKQQQLTAYKFSVISYGSFTKIISTSWSYFTILKTVYKPPE

>LstiOR37

MDLFNYIKRVFRDAKSRLQENSYESLLSLVNFVPSVAGFSIRGNTIFVPFWILHLSLLFYIYGVGCAVYQIKYAEDARDFIKSFVNVSLIVLIANNSHWFLQKRSLLKTALKEISESDVMATANESFRQKHERSVQKIKRILFIFYGFNLLNATFVYLPHRADVLNSYSMTPCFGMEPLTSSPNREICMTLLCIQEITIMVVVLNYQALLLLLIAYTALMYTLLADEIMTLNNFDRETYYNNPTVKLILPDLVKRHAILLSIIDKLKALYSGSIGVNFGSNAVCISLFFYLPLQEWLQFMPVLVYCFLVFFLYCFLCQRLTNAAELFEMSVYACGWENFELKEKKAIYFMLRQAQKPVEILAADIIPVNISTFATTLQAMFKFVTVVKV

>LstiOR38

MNLKMNFLKRYTEEDLVNIEEHNFGPFHKVYQWLTFTLTLGLLFPNPASERFRLTFIIVLLVTIQPLAVMIFIDMYKCWQERDIFNIIRHSTIIGPFLGAFFKMFLMYWKRVQTKSIVDQINSDHEAFNHLPRKQQDIAFLYIKAGVRNVERIWAPLVSVAIMMFPGMAVILTLYSYTFNDIPKKYMIHELNPPFSTDPEDMSRSPYFEVLFVYETGAAIICVLNYTAYDGLFGVATNHACMKMSLCCVKLNDAFACEDQEEMYKGVLAFIEEQQKMYKFVDLIQEIFNIWLFAILMSTMIQIGSLLFHISAGYGFDLRYTLFSFTSVVHIFLPCKHAATLKSMSTEMATMIYISGWERSRDRRVLRMIPFMLARA

>LstiOR39

MNEQLSYKSLAPHVKYLRAAGLFRLSPDSPKRHIFFHTIYLRWLIAFFSVYTVQQILKIYEVRDDVNKVMDTMFLFITNTDCIYKAAVLQKKPEKIEELLNTMKGPIFNLGVPEHRPILLATVRKALLLVHMFNRLSLITCFLWALHPTIMHMQGNPIEFAVWLPFDANQDPQFYIAVVYVWIQTSWLAYSNTTMDIFIAFLLEQCRTQVSILRLDLESVVQKSKEEAARTSSPYSEILERRFGRILIHHNEIVNSADKIQDIFGGAVFYQFVIGGWILCCSAYRIVNTQPASVEFVSMLMYTTCIVVEIFVYCFFGNELFYESNKLMDSAYAVDWLEIPVKQRRSLIIFMERVKRPICPTAGSMIPLSNSTFVSILRSAYSCYAFLRNSEH

>LstiOR40

MENQNPQERSAPIQYVRGSRAFRQFKNPPQPHMCIQDTIKDTTEKLFINVLGWQKIANPKQYNDPIPLYGGMQVPQGCGPNSNRPPLLVFAVMVNPDILKANGKNAANPNSNRPPLLVFAVMVNPDILKANGKNAANPTDREALVSLLCDFVEAMNPGLLLARSPVILKDRDLAGELKDVWLAVQNKREREKGLSQDVMYKVYDIDGIGNEDSNEEDKMNSKNCKQNDCDSLNRSSNDKKNVIKSSKQILMNAGQKSEFDSGMNNCQINQNPSREYRCSTTKADTTYCTPVYGQIVSSRENHNQINDIQQKFSSSETTKPSSSFRKDWNPVHGKTTEGWDEFSKRNVSSISNEGDVKKQRYIKNEKGNKEMSNGKSQYDFFPVFDNKAVDEVSDSNEKSGTPDKSKEQELGNEDSSKIILDAVQKLVLQPTDNKICDNKTSALSSISS

>LstiOR41

MPEKSYGTVKSNLREELNYINSMGSKIFLYPFSGRSKLVDICYLFVCFLVVVTATQLLTALLVTDLKEWIEIVNVAPNLGVVLMTLLKYTKVHNNQHVYKKIFKHFSDDLWDVVFDSYDHKKIVIRYTAIAKYATRFLFYYSVPLVVFVDSFPRIIMYLENEIIGNENPQYLYPFDGWYPFDKVNWYYTAYLWESFMTFIVVCVYAFSNMIHASYTSFICMELEILGVSIKDLITPDDVTNITNHLKVQEIHSHIKRKLKTIIRRHQFLAQLASELNIVLGDMMLLNYIFGSVFITLTIFTATVVDNMYKSLRYFFMFCSLIVEIFFNCMIGQVLSNHSEQLTDAIYSADWPFADNETKVMLLILMRRTQKPFEYTANGYLAMNLNSFSGVCSMSYQLFNLIRTAYSK

>LstiOR42

MNTTLGTPVKTNKSAGFFLKVCQLCYLFGFPNCWMESLKFSKTFTKIYDPFSKLTNVTIYLFILAEWGSMFTQNNLTEKQRSDRIMFCLSHPVLCSYRVILAYHREKLQELMYNLCLVLKEKVNDEEIEKGMVRKALAYTSALIGLCSTSLFLYGADGFNQMMRSEATFTTVITAWPLVEDTSISASAARFFLYFMWWVFMSRVFGAFAMLISLIVALEHQYKNLGKYFRNLSGIFEQDLSQAQKEKEYDQSVKYGIKLHAKTLRCTRLAQDSFSSIFGAQILLNTYVLVLLMFQMVSSERTLANVLAVIATGIAMLLSTGFLMWTGGDITVEAAILPTDMYCSGWHNCRSATGTRKLLALAMLQGQKPVMIKGLGFITISYPAYLSIVKSAYSVFSVLY

>LstiOR43

MWENLHKFGLSLEYCNLSTMLWNVGFLLRPLTLNVDSRHKDRIPISSYVFTITIASCYFYVYLFNMLWFVFIKCRATGDLITAMLVLSLGISSEIGPCKLFSMLFYKETIRTIVEGYLICDAQTLKSDRFSRNLLKTLRDVKKRVLIFWVVIIGNGLFYIIKPIVLPGRHLTEDLLIIYGLEPMYETPNYQIAFFMMCCGTTCCCYLPANIGAFLIILVGYTEATMLALSEELLNLWTDAQSYYTNNHEEIETTVDSAMVTPNDAEANRIMNTYIKQSLENIVKIHTKNIGLIQQVEHVFRGAIAVEFVLVICAIISELLGGLENTYLEMPLTFMIVGMDCLIGQKMMDACDTFESAVYDCKWENFNVANMKTVLMMLQNSQKTMVLSAGGMATLSFSCLMSVLQSTYSAYTTLRSTM

>LstiOR44

MIEESPFDKSLQKIQFAFRSTGLNLGTDGRKRNFKQNCVYLFNFLWLNTDIIGALSWLLEGIISGKNFTELTYVAPCLTLSILGDIKAFCLLLNERKVHNLIDNLRNLEAKSKNFENSEWDNIMQPEIKLFNIIIKVLNVLNCLMIVVFDVSPLILIAVKYFTTGELELLLPFLDVYPFDSFNLRYWPFAYIHQIWSECIVLLEICATDYFFFACCTHIKIQFKLLQHQFQEIISAKSVSAMDSEDPIVVRAKFQELVKWHQEIISCANKLEKIYSFSTLLNFCTSSLVICLTGFNVTTIDDKAFVITFIIFLSMSLLQVFFLCFFGDILMRSSMDVTDAVYNSRWYLSDVATGRNVLLVQTRAQTPCKLTAAGFADVNLNAYMTILSTAWSYFALLQTIYGSRS

>LstiOR45

YFFFTCCTHIRIQFKLLQHQFQEIIANRSISAVVSMNQMSIRAQFKDLIKWHQNIISCANMLEEIYSKSTLFNFLASSLMICLTGFNVTTVDDKAIVVTFIIFLSMSMMQVYFLCFFGDLLMCSSAAVADAVYNSRFYLGDVVMGKIVLLVQTRAQKPCKLTAAGFADVNLKAYMRILSTSWSYFALLQTIYSSRC

>LstiOR46

HLQENIKTLQQETKMKFVIKNTFKASQISLTYLGFTGFWTRRSEVNFVLNYCYCFVTFMFMTGISIMAQFVDLIIIWGDVALMTGTAFLLLTNVVLALKVLNMVCRREEIRAIVEETDGQLQAVNTDWGKEIVKSCDRHLTVLISIYTCLSYLTIMGWATGHEEGELPTRAWYPYDTTTSPGYEITSWQQVVGVCLGAGVNISLDTVVVSLMAQCC

>LstiOR47

FDPSQSWFIYCLVYPFEMYCMFRFIYAYLGAEFIMEALCSHLVTEFRLLREDLMLIKPVPNKRSSEGIDEIGEFVKKHQKLTLLSKQLDDIYNKVNFIVLLFATVIIGFFAFAVKVSHGYKMLVNSLAVYGMLLPVFIMCYYSQLLAVESAGIAVSAYNSPWYKGGTHHQKSIYFIIKRAQLPCYLTSLKYSPITLKTFSKVLSTTWSYFSLVTRVYEHGNEG

>LstiOR48

VTPYNCIANISGGKINMQLLTMQLLWCSIHFVSLIVMVEPCHITQREMGRTNFLVSQLMLQNTDELVTNELNVFGRYLYLNDVVYSPMGICVLSRSLVASILASVTTYLVIMMQFQATENIVYHG
